# Gene Sets Net Correlations Analysis (GSNCA): a multivariate differential coexpression test for gene sets

**DOI:** 10.1093/bioinformatics/btt687

**Published:** 2013-11-30

**Authors:** Yasir Rahmatallah, Frank Emmert-Streib, Galina Glazko

**Affiliations:** ^1^Division of Biomedical Informatics, University of Arkansas for Medical Sciences, Little Rock, AR 72205, USA and ^2^Computational Biology and Machine Learning Laboratory, Center for Cancer Research and Cell Biology, School of Medicine, Dentistry and Biomedical Sciences, Queen’s University Belfast, Belfast BT9 7BL, UK

## Abstract

**Motivation:** To date, gene set analysis approaches primarily focus on identifying differentially expressed gene sets (pathways). Methods for identifying differentially coexpressed pathways also exist but are mostly based on aggregated pairwise correlations or other pairwise measures of coexpression. Instead, we propose Gene Sets Net Correlations Analysis (GSNCA), a multivariate differential coexpression test that accounts for the complete correlation structure between genes.

**Results:** In GSNCA, weight factors are assigned to genes in proportion to the genes’ cross-correlations (intergene correlations). The problem of finding the weight vectors is formulated as an eigenvector problem with a unique solution. GSNCA tests the null hypothesis that for a gene set there is no difference in the weight vectors of the genes between two conditions. In simulation studies and the analyses of experimental data, we demonstrate that GSNCA captures changes in the structure of genes’ cross-correlations rather than differences in the averaged pairwise correlations. Thus, GSNCA infers differences in coexpression networks, however, bypassing method-dependent steps of network inference. As an additional result from GSNCA, we define hub genes as genes with the largest weights and show that these genes correspond frequently to major and specific pathway regulators, as well as to genes that are most affected by the biological difference between two conditions. In summary, GSNCA is a new approach for the analysis of differentially coexpressed pathways that also evaluates the importance of the genes in the pathways, thus providing unique information that may result in the generation of novel biological hypotheses.

**Availability and implementation:** Implementation of the GSNCA test in R is available upon request from the authors.

**Contact:** YRahmatallah@uams.edu

**Supplementary information:** Supplementary data are available at *Bioinformatics* online.

## 1 INTRODUCTION

Large-scale biological research, including genetic linkage/association studies, copy number variation, microarray and RNA-Seq expression experiments, typically compare two or more different phenotypes to infer a unique genetic background, associated with a particular phenotype. A decade ago, the methods for such analyses were dominated by univariate two-sample statistical tests, which frequently fell short from a statistical and a biological perspective because of two reasons. First, small changes in expression cannot be captured for a single gene using two-sample tests (e.g. *t*-statistic) with the correction for multiple testing (Mootha *et al.*, 2003). Second, genes do not work in isolation but interact with each other collectively; as a consequence, statistical tests need to account for a multivariate nature of expression changes (Emmert-Streib and Glazko, 2011; Glazko and Emmert-Streib, 2009). These shortcomings catalyzed the appearance of conceptually new methodologies for the analysis of genomic data. Instead of considering a single gene as an expression unit, new methodologies started to operate with gene sets (corresponding, e.g. to biological pathways), considering a gene set as the unit of expression. The first test of this kind was the gene set enrichment analysis (Mootha *et al.*, 2003). To date many methodologies for testing the differential expression of gene sets (molecular pathways, biological processes) have been suggested and are collectively named gene set analysis (GSA) approaches (Ackermann and Strimmer, 2009; Dinu *et al.*, 2009; Emmert-Streib and Glazko, 2011; Huang da *et al.*, 2009). GSA approaches can be either *competitive* or *self-contained*. Competitive approaches compare a gene set against its complement that contains all genes except genes in the set, and self-contained approaches compare whether a gene set is differentially expressed between two phenotypes (Goeman and Buhlmann, 2007; Tian *et al.*, 2005). Unfortunately, some competitive GSA approaches are influenced by the genomic coverage and the filtering of the data and can increase their power by the addition of unrelated data and even noise (Tripathi *et al.*, 2013). Owing to these problems, we focus in this article on self-contained methods only. Self-contained approaches, depending on the statistics used for the testing, test different null hypotheses (Emmert-Streib and Glazko, 2011; Glazko and Emmert-Streib, 2009; Rahmatallah *et al.*, 2012). The possibility to formulate different statistical hypotheses enables the formulation and exploration of different biological hypotheses. However, for GSA approaches, testing hypotheses other than the equality of the mean expression vectors remains underexplored. We recently suggested to extend a univariate analysis of differential gene variability (Ho *et al.*, 2008) to a multivariate case of gene sets (Rahmatallah *et al.*, 2012) with a multivariate non-parametric ‘radial’ Kolmogorov–Smirnov test, sensitive to alternatives that have similar mean vectors but are different in their scale (Friedman and Rafsky, 1979). We found that for several tumor types, the pathways, detected exclusively by the radial Kolmogorov–Smirnov test, were mostly tumor-specific, whereas the pathways with differences in the mean expression vectors were detected simultaneously in different tumor types (Rahmatallah *et al.*, 2012). The main focus of this article is to develop a novel multivariate differential coexpression analysis approach for gene sets.

The first approach for testing the differential coexpression of gene pairs, tested the equality of pairwise correlations to identify gene pairs with correlated expression patterns in one phenotype but not the other (Dawson and Kendziorski, 2012; Fukushima, 2013; Yu *et al.*, 2011). Its extension to the general multivariate case, involving gene sets with >2 genes, depends on the biological context. When there are no a priori defined gene sets available, the simplest way of differential coexpression analysis, implemented in the R package CoXpress, is to find clusters of coexpressed genes in one condition and check whether these clusters show no correlation in another condition (Watson, 2006). Another approach, e.g. implemented in the R package DiffCoEx, constructs adjacency matrices of all genes under different conditions, transforms adjacency matrices into a matrix of adjacency differences and uses a topological overlap measure to infer clusters of differentially coexpressed genes (Tesson *et al.*, 2010). When there are a priori defined gene sets available, the differential coexpression of gene sets can be found by using the gene sets coexpression analysis (GSCA) (Choi and Kendziorski, 2009). In this approach, the Euclidian distance between two correlation vectors (constructed from diagonal matrices of pairwise correlations for different conditions) is calculated and the significance of the difference is estimated using permutation test. The differentially coexpressed gene sets (dCoxS) method is similar to GSCA in its overall strategy. First, the gene’s pairwise coexpressions are characterized separately for two conditions, and second, the similarities of these characteristics are estimated (Cho *et al.*, 2009). The dCoxS uses relative entropy matrices in place of correlation matrices, as used by GSCA, and the correlation coefficient between the upper-diagonal elements of these matrices as a measure of their similarity. The new property of dCoxS is that the coexpression of two different pathways can also be estimated (Cho *et al.*, 2009). There are also other approaches for the differential coexpression analysis of gene sets (Emmert-Streib, 2007; Freudenberg *et al.*, 2010; Yu and Bai, 2011); the common aspect of all these approaches is that they account for changes in aggregate measures of pairwise correlations only.

In this article, we present a novel approach that assesses multivariate changes in the gene coexpression network between two conditions. Importantly, we do not infer ‘gene coexpression networks’ explicitly, but, instead, we estimate net correlation changes by introducing for each gene a weight factor that characterizes its cross-correlations in the coexpression networks. Weight vectors in both conditions are found as eigenvectors of correlation matrices with zero diagonal elements. The Gene Sets Net Correlations Analysis (GSNCA) tests the hypothesis that for a gene set there is no difference in the gene weight vectors between two conditions.

Furthermore, we suggest a new graphical visualization to present the full coexpression network that highlights the most highly correlated genes, using the union of the first and second minimum spanning trees (MST2). We show that genes in the center of MST2 have large weights, and we demonstrate that hub genes—genes with the largest weight in the pathways—correspond in real data frequently to pathway regulators. In previous studies, MST was mainly used for cluster analysis in gene expression studies. For instance, Xu *et al.* (2001) suggested gene expression data clustering based on MST, which rigorously converts a multidimensional clustering problem to a tree partitioning problem. Prom-On *et al.* (2011) presented a method to improve the biological relevance in the inference of functional modules from the gene expression data by enhancing the structure of a weighted gene coexpression network using MST. However, to our knowledge, no attempt has been made so far to present the full coexpression network that highlights the most highly correlated genes via MST2 structure.

We choose to compare GSNCA with the GSCA (Choi and Kendziorski, 2009), as the idea behind GSCA—comparing pairwise measures of the genes coexpression between two conditions—is frequently used in other approaches (e.g. dCoxS). The conceptual differences between our approach and GSCA are illustrated in simulations as well as in the application to two gene expression data sets.

## 2 METHODS

In the following, we are considering two biological conditions with different outcomes, with *n*_1_ samples of expression measurements of *p* genes (that form a gene set) for the first, and *n*_2_ samples of measurement of the same *p* genes for the second conditions. Let *R_l_* with elements *r_ij_* denote a *p* × *p* gene correlation matrix (*l* = 1, 2) for a given condition. Let *N_l_* denote a completely connected coexpression network (*l* = 1, 2), with *p* nodes (genes) and *p*(*p*-1)/2 edges, where the weight of an edge between any two nodes *i* and *j* is given by 1- *|r_ij_*| (correlation distance).

The organization of this section is as follows: the GSNCA and the GSCA, we use for comparison, are explained in Section 2.1. The MST approach for the visualization of a backbone of coexpression network is given in Section 2.2, and the simulation setup is outlined in Section 2.3. Section 2.4 presents the biological datasets we use to demonstrate the performance of GSNCA and GSCA. All computations in this work were implemented using the R (version 2.15.3) computing language.

### 2.1 Gene sets net correlations analysis

To quantitatively characterize the importance of gene *i* in a correlation network, we introduce a weight (*w_i_*) and set *w_i_* to be proportional to a gene’s cross-correlation with all the other genes. Then, the objective is to find a weight vector *w*, which achieves equality between a gene weight and the sum of its weighted cross-correlations for all genes simultaneously. Thus, genes with high cross-correlations will have high weights that may indicate their regulatory importance. This problem can be formulated as a system of linear equations
(1)
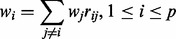

or equivalently in matrix form
(2)
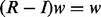



This is an eigenvector problem that has a unique solution when the eigenvalue 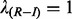, *w* > 0. Because the matrix 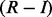 is not guaranteed to have eigenvalue 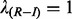, we introduce a multiplicative factor, *γ,* which ensures a proper scaling for eigenvalues and solves the following problem
(3)
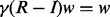



Because the matrix *R* is non-negative, irreducible, symmetric and has unity diagonal elements, its eigenvalues are real and from the Perron–Frobenius theorem for non-negative matrices (Meyer, 2001); it follows that *R* has a largest eigenvalue 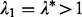, with a multiplicity of 1 and the other (*p*-1) eigenvalues all satisfy 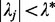 for *j* ≠ 1. This eigenvalue corresponds to a unique positive eigenvector v* such that 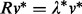.

**Proposition**

For a non-negative irreducible correlation matrix *R*, solving 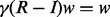 as an eigenvector problem for *w* > 0 has the unique solution *w* = v*, where v* is the positive eigenvector corresponding to the largest real eigenvalue of *R* (λ*). This solution is achievable if the following condition is met
(4)
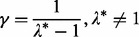

**Proof of Proposition**

We solve 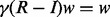 for *w* > 0 as an eigenvector problem where the unique solution is the eigenvector of matrix 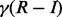 corresponding to 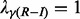. Setting 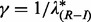 where 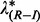 is the largest eigenvalue of the matrix 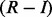 guarantees that the largest eigenvalue of matrix 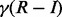 will be 1, and consequently the corresponding eigenvector is the unique solution. Because the matrices *R* and 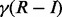 have the same eigenvectors, the unique solution is *w* = v*, where v* is the positive eigenvector corresponding to the largest eigenvalue of *R*. Because the eigenvalues of the matrix 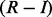 are exactly 1 less than the eigenvalues of matrix *R*, 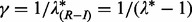, whereas 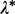 is the largest eigenvalue of *R*.

As a test statistic, 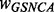, we use the *L*_1_ norm between the scaled weight vectors *w*^(1)^ and *w*^(2)^ (each vector is multiplied by its norm to scale the weight factor values around one) between two conditions,
(5)
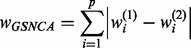



**Fig. 1.**
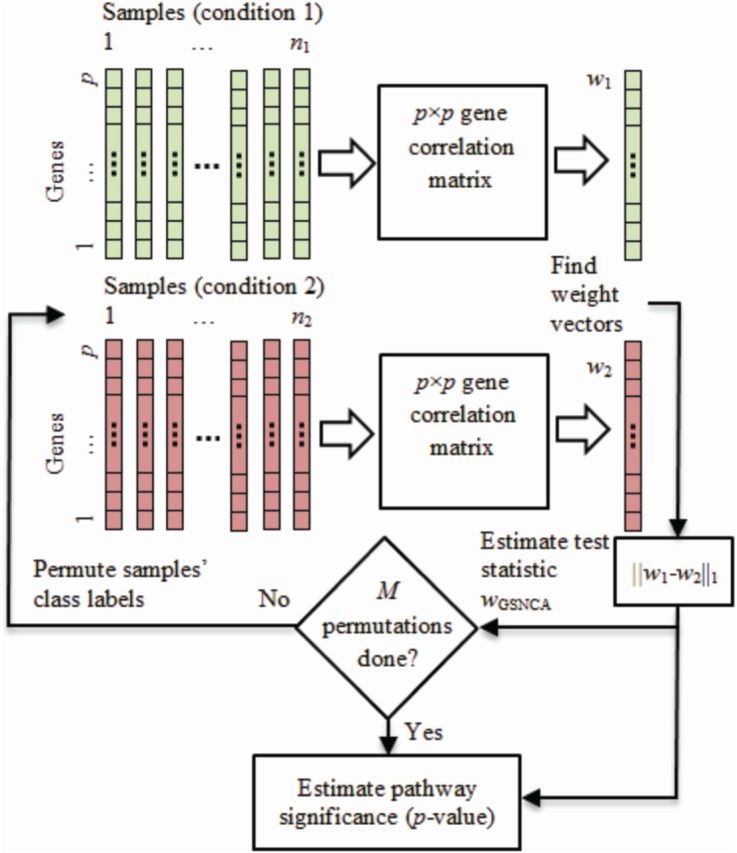
Schematic diagram of GSNCA. Shown are expression samples from a single set of *p* genes in two biological conditions

We use this test statistic to test the hypothesis *H*_0_: 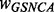 = 0 against the alternative *H*_1_: 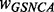 ≠ 0. The *P*-values for the test statistic are obtained by comparing the observed value of the test statistic to its null distribution, which is estimated using a permutation approach. We call this test GSNCA. The GSNCA test is illustrated in Figure 1. We found that the introduced weights are somewhat similar to the eigenvector centralities, defined for binary (adjacency) matrices.

The performance of GSNCA is compared with the performance of GSCA (Choi and Kendziorski, 2009). Briefly, GSCA works as follows. For all *p*(*p*-1)/2 gene pairs, GSCA calculates correlations in the two biological conditions. The Euclidean distance, adjusted for the size of a gene set is used as a test statistic,
(6)
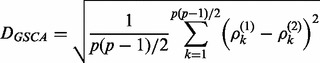

Here, *k* indexes the gene pairs within the gene set, and 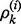 denotes the correlation of gene pair *k* in condition *i*. We would like to note that in this context, the Euclidian distance is similar to the graph edit distance, frequently used by methods aiming to detect the differential correlation between pathways (Emmert-Streib, 2007). GSCA tests the hypothesis *H*_0_: 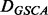 = 0 against the alternative *H*_1_: 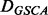 ≠ 0.

### 2.2 Minimum spanning trees

For a graph *G*(*V*,*E*) where *V* is the set of vertices and *E* is the set of edges, the first MST is defined as the acyclic subset *T*_1_ ⊆ *E* that connects all vertices in *V* and whose total length 
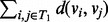
 is minimal. The second MST is defined as the MST of the reduced graph G(V, E-T1). The union of the first and second MST (denoted by MST2), constructed from using correlation distances, gives the minimal set of essential links (interactions) among genes, which we interpret as a network of functional interactions. Each vertex in the MST2 has a minimum degree of 2 if all the *p*(*p*-1)/2 pairwise correlations between genes are considered. A gene that is highly correlated with all the other genes tends to occupy a central position and has a relatively high degree in the MST2 because the shortest paths connecting the vertices of the first and second MSTs tend to pass through this gene. In contrast, a gene with low intergene correlations most likely occupies a non-central position in the MST2 and has a degree of 2. The weight factors, inferred from GSNCA, correlate to some extent with genes centralities in the MST2: genes with large weights are placed near the center of the MST2, and genes with small weights are placed on the periphery (see Section 3.2 for examples). Adopting network terminology, a gene with the largest weight is a hub gene, coexpressed with all the other genes in a pathway. In Section 3, we illustrate a coexpression analysis of gene sets with MST2 and discuss the interpretation of hub genes. The MST2 for selected pathways of biological datasets are provided in Supplementary Materials S1 and S2.

### 2.3 Simulation setup

To evaluate the performance of GSNCA and GSCA in a fully controlled setting, we designed simulation experiments that mimic real expression data as close as possible. In a real biological setting, not all genes in a gene set are coexpressed, and intergene correlations vary in strength. Therefore, we introduced two parameters: *γ*, the percentage of genes, truly coexpressed in a gene set (detection call), and *r*, the strength of intergene correlation. It is important to understand how exactly these parameters influence the power of different tests.

We simulated two samples of equal size, *N*/2 (*N* = 40) from *p*-dimensional normal distributions *N*(0,Σ_1_) and *N*(0,Σ_2_), representing two biological conditions with different outcome. We test the null hypothesis *H*_0_: 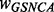 = 0, where 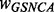 is found from Equation (5). Two cases were considered: the number of genes in a gene set (pathway) is relatively small (*p* = 20) and relatively large (*p* = 100 and *p* = 200). To ensure that Σ_1_ and Σ_2_ are positive definite, two different scenarios were studied.

First, Σ_1_ was set to *I_p_*_×_*_p_* and Σ_2_ was selected such that its elements are

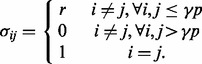



**Fig. 2.**
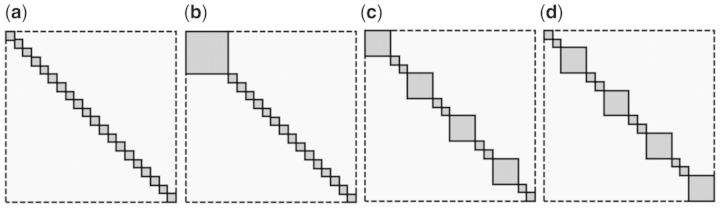
(**a** and **b**) The correlation matrices for the first simulation setup in two conditions with *p* = 20 and γ = 0.25. (**c** and **d**) The correlation matrices for the second simulation setup in two conditions with *p* = 20, *β* = 0.25 and γ = 0.6. Dark and light colors represent high and low correlation values

For the *γ* parameter, the proportion of genes truly coexpressed in a gene set, we consider *γ*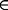 {0.25, 0.5, 0.75, 1}, and for the parameter *r*, controlling the strength of the intergene correlations we consider *r*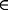 {0.1, 0.2, … , 0.9}. Figure 2a and b illustrate this setup for *p* = 20 and *γ* = 0.25 where both correlation matrices under the alternative hypothesis are shown. Dark and light colors represent high and low correlations, respectively. This design presents a gene set with low intergene correlations in condition 1 (Fig. 2a) and one group of highly coexpressed genes in condition 2 (Fig. 2b). The purpose of the design is to demonstrate a fundamental difference between GSCA and GSNCA. The power of GSCA is expected to increase as *r*, as well as the size of the highly coexpressed group of genes in condition 2 increase. Instead, the power of GSNCA is expected to increase as the difference in intergene correlations between two conditions increases. If all genes (*γ* = 1) are highly coexpressed for condition 2, the coexpression matrices in the two conditions are 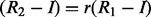. The eigenvectors for both matrices are the same and GSNCA does not detect changes regardless of the value of *r*. The maximum change in the coexpression structure between condition 1 and 2 using this design occurs when half of the genes (*γ* = 0.5) are highly coexpressed in condition 2. That is, GSNCA should have the highest power when *γ* = 0.5.

Second, for both Σ_1_ and Σ_2_ we form diagonal blocks of equal size *βp*, where *β* is the ratio of block size to gene set size (*p*). Then, for each block separately the first scenario is reproduced. Hence, each block will have *γβp* genes with intergene correlation specified by *r*, whereas all the other genes in the block have zero correlations. The locations of the *γβp* coexpressed genes inside each block are assigned differently for Σ_1_ and Σ_2_ under alternative hypothesis. Although for Σ_1_ these genes occupy the upper-left corner of the block, for Σ_2_ they occupy the lower-right corner. Figure 2c and d illustrates this setup for *p* = 20, *β* = 0.25 and *γ* = 0.6 where both correlation matrices under the alternative hypothesis are shown. Dark and light colors represent high and low correlations, respectively. Depending on *γ*, the two alternate coexpressed gene groups in Σ_1_ and Σ_2_ may have a few common genes (when *γ* > 0.5) or may be exclusive (when *γ* ≤ 0.5). Figure 2c and d shows four common genes between highly coexpressed gene groups. All intergene correlations outside the blocks are set to zero or a small value. This design presents a gene set with low intergene correlations except for selected groups of highly coexpressed genes. The membership of the genes in these groups is changing between the two conditions with the possibility of having a few common members between the two conditions (when *γ* > 0.5). Because the intergene correlation and the structure of the coexpression matrix *R* vary between the two conditions, both GSCA and GSNCA should detect changes.

### 2.4 Biological data

We illustrate the GSNCA approach using the NCI-60 cell lines (p53) and acute lymphoblastic leukemia (ALL) datasets. The p53 dataset comprises 50 samples of NCI-60 cell lines differentiated based on the status of the TP53 gene: 17 cell lines carrying normal (wild type, WT) TP53 gene and 33 cell lines carrying mutated TP53 (MUT) (Olivier *et al.*, 2002; Subramanian *et al.*, 2005). For this dataset, probe level intensities were quantile normalized and transformed to the log scale. The ALL dataset consists of microarrays from 128 different individuals with acute lymphoblastic leukemia (ALL). There are 95 samples with B-cell ALL (Chiaretti *et al.*, 2004) and 33 with T-cell ALL (Chiaretti *et al.*, 2005). Tumors carrying the BCR/ABL mutation (37 samples) were compared to those with no cytogenetic abnormalities (42 samples). To normalize samples, the robust multiarray analysis procedure (Irizarry *et al.*, 2003) was used.

The microarray platforms for the p53 and ALL datasets are, respectively, hgu133plus2 and hgu95av2 with Affymetrix gene identifiers. Genes without mapping to Entrez and Symbol identifiers were discarded. Probes with duplicate identities were assessed and the probe with the largest absolute value of *t*-statistic between two conditions was selected as a gene match. Gene sets were taken from the C2 pathways set of the molecular signature database (MSigDB) (Liberzon *et al.*, 2011; Subramanian *et al.*, 2005; Wu and Smyth, 2012) where a total of 3272 pathways are present. Pathways with <15 or >500 genes were discarded and the resulted dataset comprised 8806 genes and 2360 pathways to analyze.

## 3 RESULTS

### 3.1 Simulation study

#### 3.1.1 Type I error rate

Table 1 presents the estimates of the attained significant levels for the GSCA and GSNCA tests (1000 independent simulations were used). As can be seen, the estimates of Type I error rate when Σ_1_ = Σ_2_ = *I* under different parameter settings for both tests are similar and rather conservative.

**Table 1.** Type I error rate for GSNCA and GSCA; *α* = 0.05

GSNCA GSCA	*p* = 20	*p* = 60	*p* = 100
*n*_1_ = *n*_2_ = 10	0.054	0.051	0.050
	0.046	0.048	0.046
*n*_1_ = *n*_2_ = 20	0.050	0.051	0.050
	0.047	0.048	0.048
*n*_1_ = *n*_2_ = 30	0.049	0.051	0.047
	0.048	0.051	0.049

#### 3.1.2 The power of tests to detect changes in correlation structure

**Fig. 3.**
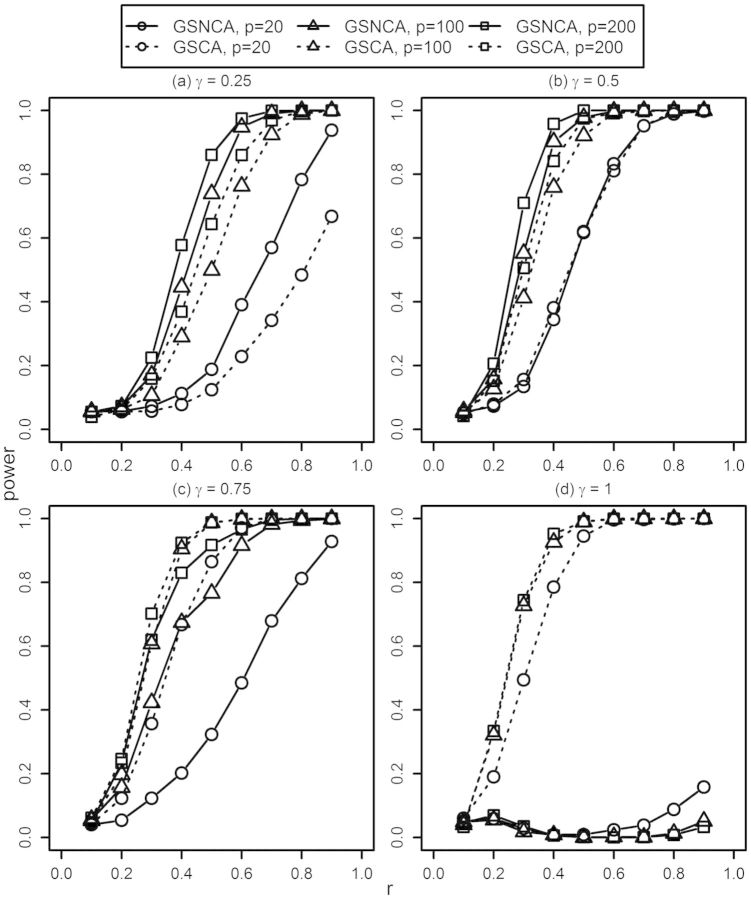
The power curves of GSNCA and GSCA for the first simulation setup when the alternative hypothesis is true (*N* = 40)

Figure 3 presents power estimates under the first simulation scenario (Section 2.3) for different parameter settings. For each parameter setting, 1000 independent simulations were used and the average (mean) power is shown.

First, consider the case when 25% of genes in a gene set are coexpressed (*γ* = 0.25). This is highly plausible for real expression data, as not many genes in a gene set are highly coexpressed (Montaner *et al.*, 2009; Tripathi and Emmert-Streib, 2012). The GSNCA shows higher power than GSCA for all settings (*p* = 20, 100, 200). Second, consider the case when 50% of genes in a gene set are coexpressed (*γ* = 0.5). Both tests show similar power when the size of gene set is relatively small (*p* = 20). However, when the size of gene set is relatively large (*p* = 100 and *p* = 200) the GSNCA outperforms the GSCA. Third, consider the case when 75% of genes in a gene set are coexpressed (*γ* = 0.75). GSCA outperforms GSNCA when the size of gene set is relatively small (*p* = 20). However, their performance becomes similar when the number of genes increases (*p* = 100, *p* = 200). Fourth, consider the case when 100% of genes in a gene set are coexpressed (*γ* = 1). This case illustrates a clear-cut difference in performance between GSNCA and GSCA. GSNCA has the highest power when *γ* = 0.5 (see Section 2.3 for detail).

**Fig. 4.**
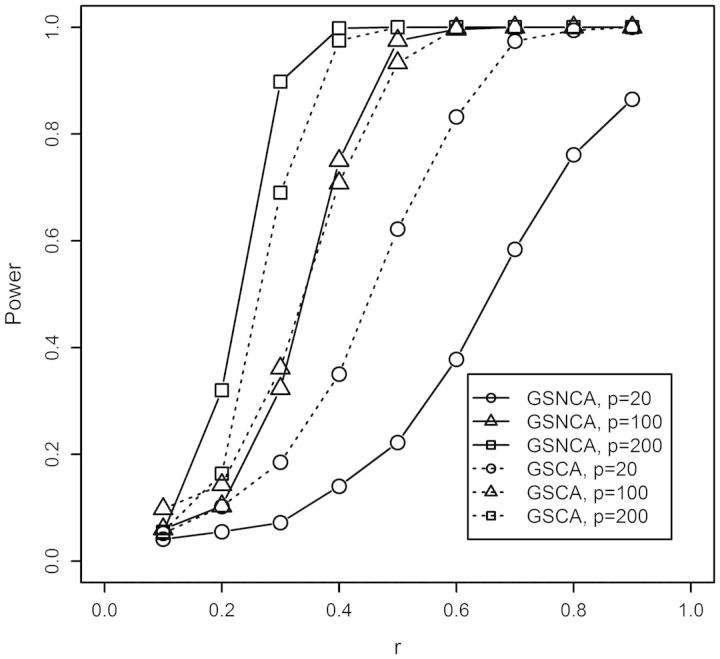
The power curves of GSNCA and GSCA for the second simulation setup when the alternative hypothesis is true (*N* = 40)

Figure 4 presents power estimates under the second simulation scenario (see Section 2.3) for different parameter settings. For all simulations, we set *β* = 0.25 and used *γ* = {0.6, 0.4, 0.5} for *p* = {20, 100, 200}, respectively. These simulation parameters result in 3, 10 and 25 truly coexpressed genes for *p* = 20, 100 and 200, respectively. The results show that GSCA outperforms GSNCA when the size of the gene set is relatively small (*p* = 20). When *p* is 100, an opposite trend is observed, and when *p* is further increased to 200, GSNCA outperforms GSCA. It is also worth noting that when the two alternate coexpressed gene groups in Σ_1_ and Σ_2_ are exclusive, the detection power of GSNCA increases as all genes in both of these groups will show high net coexpression change between two conditions. Common genes between these groups will have the same net coexpression between the two conditions.

To summarize the simulation results, GSNCA outperforms GSCA when the size of gene set is relatively large and when changes in coexpression non-uniformly affect pathway members. GSCA performs the best when all genes in a pathway are differentially coexpressed.

### 3.2 P53 dataset

To study the tests performance, we categorized pathways into three groups: detected exclusively by GSNCA, exclusively by GSCA and by both. The number of pathways detected exclusively by GSNCA, GSCA and both were, respectively, 130, 55 and 15. A complete list of these pathways is provided in Supplementary Table S1.

Pathways found by GSCA and GSNCA approaches fall into four major categories: (i) tumorigenesis, (ii) monogenic changes in tumors, (iii) signaling pathways and (iv) changes in metabolism. In turn, every category can be additionally subdivided into two more specific categories: (i) tumor signatures and comparative analysis of tumor signatures, (ii) fusions and single gene targeting, (iii) response to anticancer treatment and general system response and (iv) cellular and nucleic acid metabolisms (Supplementary Table S2). GSCA approach finds more pathways, related to metabolism, whereas GSNCA preferentially detects signaling pathways—response to anticancer treatment and general system response. The biological context of differences between pathways, found exclusively by GSNCA and GSCA reflects the difference in null hypotheses, tested by these approaches. GSCA tests the hypothesis that the averaged difference among all pairwise correlations is equal to zero, whereas GSNCA tests that the difference between two weight vectors, corresponding to genes net correlations, is equal to zero.

Cancer agents act on molecular targets related to p53 that are frequently hub genes (see later in the text). Mutation in p53 causes changes in targets interactions with the rest of the pathway and consequently changes in their weights, whereas overall average correlation for a pathway may remain the same. Several aspects of cellular metabolism are also affected by changes in p53 status: p53 has been shown to regulate TP53-induced glycolysis, synthesis of cytochrome *c* oxidase and damage-regulated autophagy (Jones and Thompson, 2009; Vousden and Ryan, 2009). Thus, most metabolic networks should be affected by mutated p53 indirectly, through reduced nutrient or energy levels, corresponding to changes in average correlations between two conditions.

**Fig. 5.**
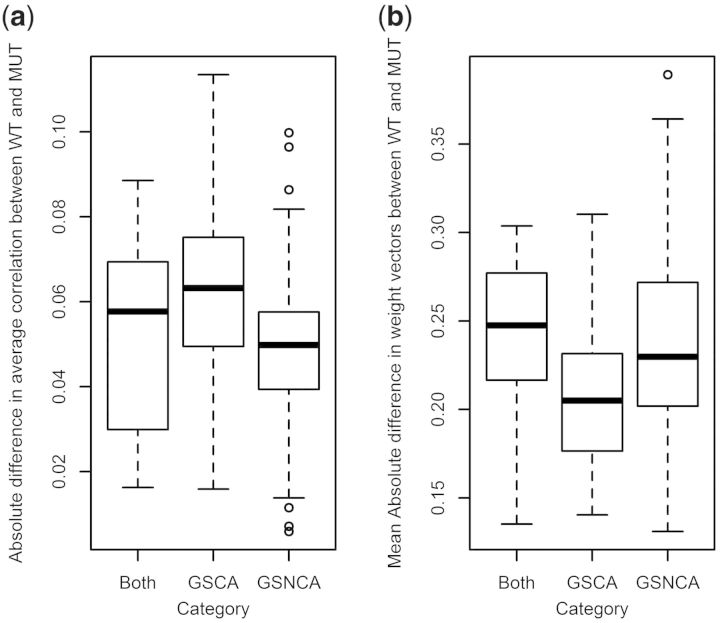
(**a**) The difference in average correlations and (**b**) the average difference in weight factors between the two phenotypes detected by different approaches for p53 dataset

To illustrate the difference between GSCA and GSNCA approaches quantitatively, for each set of pathways, detected exclusively by GSNCA, exclusively by GSCA and by both, we found (i) the average difference in weight factors between two phenotypes, WT and MUT (average 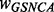) and (ii) the difference in average correlations between two phenotypes (Fig. 5). Pathways detected exclusively by GSNCA or by both tests show higher differences in weight factors than pathways detected exclusively by GSCA, whereas pathways detected exclusively by GSCA show higher difference in average correlations (Fig. 5). This observation is in agreement with our qualitative analysis of biological differences between pathways, exclusively detected by different approaches.

#### 3.2.1 Hub genes

GSNCA identifies hub genes—genes with the largest weights in each pathway. Hub genes provide useful biological information beyond the test result that a pathway is differentially coexpressed between two conditions. In what follows, we discuss several examples of hubs functional roles in pathways identified using GSNCA approach. MST2 of all significant pathways for p53 data and hub genes with corresponding weights are provided in Supplementary Material S1).

**Fig. 6.**
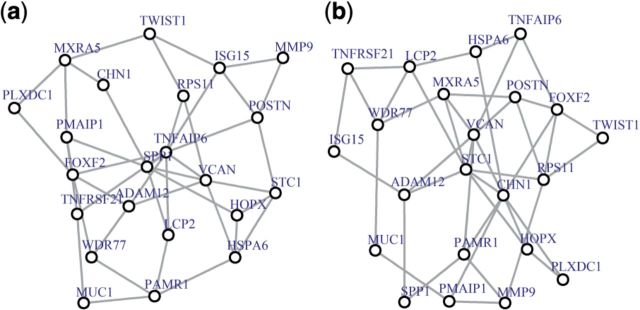
MST2s of LU_TUMOR_VASCULATURE_UP coexpression network. (**a**) MST2 for p53 WT, the hub gene is TNFAIP6 and (**b**) MST2 for p53 MUT, the hub gene is VCAN

***Major regulator*.** LU_TUMOR_VASCULATURE_UP (Fig. 6) pathway comprises genes overexpressed in ovarian cancer endothelium (Lu *et al.*, 2007). In the original study, TNFAIP6 (tumor necrosis factor, α-induced protein 6) identified in our analysis as hub gene (Fig. 6a) was 29.1-fold overexpressed in tumor endothelium, and was suggested to be specific for ovarian cancer vasculature (Lu *et al.*, 2007). It indicates that TNFAIP6 can be an important regulator of ovarian cancer, and its property of being a hub enhances the original observation. When p53 is mutated (Fig. 6b) hub gene is VCAN, containing p53 binding site. Its expression is highly correlated with p53 dosage (Yoon *et al.*, 2002). Thus, both hub genes provide adequate information about the underlying biological processes. Interestingly, in this example TNFAIP6 has the highest degree and betweenness centralities, whereas VCAN does not (data not shown).

Another interesting example is YAO_HOXA10_TARGETS_ VIA_PROGESTERONE_DN pathway (Yao *et al.*, 2003). The authors show that Hoxa-10 mediates proliferation of uterine stromal cells in response to progesterone, and the pathway consists of Hoxa-10 downregulated targets. GSNCA identifies Hoxa-10 as hub gene for those targets, in agreement with experimental evidence (Yao *et al.*, 2003).

**Fig. 7.**
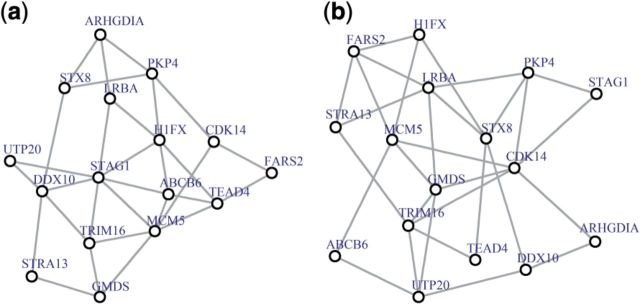
MST2s of GAJATE_RESPONSE_TO_TRABECTEDIN_DN coexpression network. (**a**) MST2 for p53 WT, the hub gene is STAG1 and (**b**) MST2 for p53 MUT, the hub gene is CDK14

***Specific regulator.*** Trabectedin (ET-743) induces a delay in S phase and an arrest in G2/M phase in human cancer cells (Gajate *et al.*, 2002). GAJATE_RESPONSE_TO_TRABECTEDIN_DN pathway (Fig. 7) presents genes, downregulated in response to ET-743. For p53 wild type data, hub gene is STAG1 (stromal antigen 1, Fig. 7a) that encodes a component of cohesin, a multisubunit protein complex that provides sister chromatid cohesion and has a specific function in cell division. When p53 is mutated (Fig 7b), hub gene is CDK14 (cell division protein kinase 14) that controls overall cell cycle progression and cell proliferation. In this example, hub genes in both conditions also have highest degree and betweenness centralities (data not shown). R package igraph (version 0.6.5) was used for network visualization.

***The p53 target***. p53 is a major tumor suppressor protein, and 44.4% of all pathways, found by GSNCA are related to tumorigenesis (Supplementary Table S2). It is logical to assume that p53 and its targets (611 genes, www.genecards.org) should be enriched in these pathways. The p53 targets frequently occupy hub positions in the case of p53 WT (hypergeometric test *P* = 1.611 × 10^−^^5^).

This demonstrates that the property of being hub correlates with supposed biological function. It should be noted that hub genes in pathways detected exclusively by GSCA showed no significant enrichment (*P* = 0.095) in p53 targets.

Overall, the analysis of hub genes provides biologically relevant information about their role in the underlying processes: it highlights genes, major and specific pathways regulators and also genes that are affected by global difference between two conditions, in this case by mutation in p53 gene. Thus, hub genes can help identify new biomarkers of tumor progression, metastasis and other markers of major phenotypic changes.

### 3.3 ALL dataset

**Fig. 8.**
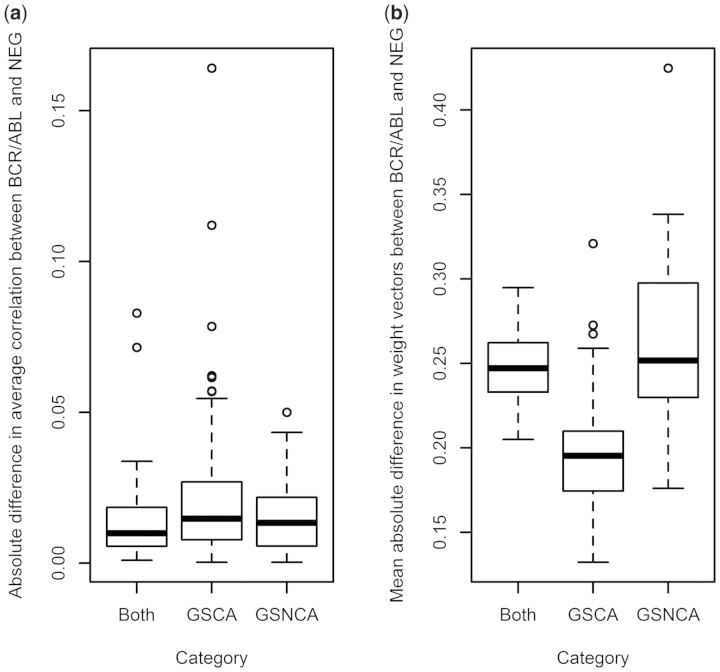
(**a**) The difference in average correlations and (**b**) the average difference in weight factors between the two phenotypes detected by different approaches for ALL dataset

For the ALL dataset, the number of pathways detected exclusively by GSNCA, GSCA and both were, respectively, 59, 162 and 27. Pathways detected exclusively by GSNCA or by both tests again show higher differences in the weight factors than the pathways detected exclusively by GSCA; differences in the average correlations among the three groups of pathways are less pronounced than in the case of p53 data (Fig. 8). A complete list of these pathways with their corresponding GSNCA and GSCA *P*-values is provided in Supplementary Table S3. MST2 of all significant pathways for ALL data and hub genes with corresponding weights are provided in Supplementary Material S2.

Next, we selected BCR/ABL-related genes (350 genes, www.genecards.org) and examined the KEGG_CHRONIC_ MYELOID_LEUKEMIA pathway, known to be specifically associated with the BCR/ABL mutation. This pathway has 28 BCR/ABL-related genes (out of 70 genes), resulting in significant enrichment (hypergeometric test *P* = 3.585 × 10^−^^21^, Supplementary Table S3). KEGG_CHRONIC_MYELOID_LEUKEMIA was detected exclusively by GSNCA with high significance (*P* = 0.005). Although GSCA detects pathways with significant differences in correlations, it failed to detect this pathway (*P* = 0.219).

From the analysis of both datasets, we conclude that changes in net correlations, overlooked by tests measuring average correlation changes, are important and point toward pathways that are crucially involved in phenotypic changes between two conditions.

## 4 DISCUSSION

In this article, we proposed a new multivariate statistical test, GSNCA that detects significant changes in the coexpression structure between two different biological conditions. This represents a major improvement over earlier approaches that compare averaged pairwise correlations, or other pairwise measures of coexpression, because our approach is able to detect changes previous approaches would miss. This strength of GSNCA stems from including all cross-correlations of a single gene. In this way, GSNCA is accounting for the multivariate structure of the data.

The analyses of the p53 and the ALL datasets confirmed that the principal difference between GSNCA and GSCA is in the ability of the former test to detect pathways with changes in the net correlation structure. For the p53 data set, GSNCA preferentially detects signaling pathways—response to anticancer treatment and general system response, whereas GSCA finds more pathways related to metabolism. Anticancer treatments frequently involve cancer agents that act on molecular targets such as p53 and p53-related genes. In agreement, GSNCA preferentially identifies pathways with p53-related hub genes (see later in the text) in one phenotype, but not the other, reflecting net correlation changes caused by differences in the p53 status. In turn, GSCA preferentially identifies pathways with averaged correlation changes, as we would expect for metabolic pathways affected by p53 status through homeostatic regulation of energy and amino acids metabolisms. Pathways detected exclusively by GSNCA show higher differences in weight factors than pathways detected exclusively by GSCA, whereas pathways detected exclusively by GSCA show higher differences in the average correlations (Fig. 5). For the ALL dataset the difference between pathways, identified by GSCA and GSNCA is explained similarly (Fig. 8).

GSNCA has an interesting property that we discuss in more detail here. The accurate reconstruction of gene networks from experimental data is considered a major goal of systems biology (Stolovitzky *et al.*, 2007). Depending on the biological context of the problem, there are many approaches available (Emmert-Streib *et al.*, 2012), and the most commonly used methods are based on correlation measures (Zhang and Horvath, 2005), information-theoretic approaches (Faith *et al.*, 2007; Margolin *et al.*, 2006; Meyer *et al.*, 2007) and probabilistic graphical models (Friedman, 2004; Friedman *et al.*, 2000). Our approach does not infer coexpression networks but uses the structure of the full coexpression network encoded in its correlation matrix to approximately identify changes in coexpression networks between two conditions. Thus, GSNCA actually avoids the problem of network inference and gets directly to the question that usually motivates the network inference—what are the differences in coexpression networks. Because the network inference step can be computational intense and method-dependent, this can be a useful property when the research question is the difference between coexpression networks.

Furthermore, we introduced a new way to visualize coexpression networks with all correlations present, using the union of the first and second MST2. MST2 is constructed using correlation distance and by construction, genes in the center of the MST2 have large weights. The analysis of the p53 data suggests that genes with large weights—hub genes—have interesting biological properties. The hubs frequently correspond to pathway regulators, and in many cases a functional difference between hub genes in two conditions reflects the global change underlying the different phenotypes. Actually it is expected, as hub genes with large weights may have high degree and betweenness centralities that are considered to be frequent indicators of genes importance (Gu *et al.*, 2012). Interestingly, the degree and betweenness centralities were the highest for hub genes for both conditions in one example (Fig. 7) but correlated with high weight of hub gene in just one condition in another example (Fig. 6). In practice, it means that the suggested weights sometimes correlate with the centrality measures, but generally characterize node importance differently. Thus, hub genes identified by GSNCA can be interesting candidates for further biological studies because, depending on the study, they may represent regulators of tumor progression, drug targets or critical pathway switches.

In sum, we presented a novel approach that characterizes differences in coexpression networks, without requiring the network inference step. In general, GSCNA should be a valuable addition to GSA approaches because (i) it identifies differentially coexpressed pathways that are overlooked otherwise, (ii) eigenvectors are computed efficiently and (iii) it provides information about the importance of genes in pathways that may result in new biological hypotheses.

*Funding*: Arkansas Biosciences Institute, the major research component of the Arkansas Tobacco Settlement Proceeds Act of 2000 (in part) and the Translational Research Institute (TRI) at the University of Arkansas for Medical Sciences (grant UL1TR000039). EPSRC (EP/H048871/1) (to F.E.S.).

*Conflict of Interest*: none declared.

## Supplementary Material

Supplementary Data
