# Supplementary material for: Gene Sets Net Correlations Analysis (GSNCA): a multivariate differential coexpression test for gene sets
Source: Bioinformatics. 2013 Nov 30;30(3):360–8. doi: 10.1093/bioinformatics/btt687 (PMC4023302; doi:10.1093/bioinformatics/btt687)
Supplement: Supplementary Data [file supp_30_3_360__index.html]

Gene Sets Net Correlations Analysis (GSNCA): a multivariate differential coexpression test for gene sets — Gene Sets Net Correlations Analysis (GSNCA): a multivariate differential coexpression test for gene sets — Supplementary Data 

# Gene Sets Net Correlations Analysis (GSNCA): a multivariate differential coexpression test for gene sets

## Supplementary Data

files

**Files in this Data Supplement:**

- Supplementary Data - pdf file
- Supplementary Data - pdf file
- Supplementary Data - pdf file
- Supplementary Data - txt file
- Supplementary Data - txt file
