# Supplementary material for: Gene Sets Net Correlations Analysis (GSNCA): a multivariate differential coexpression test for gene sets
Source: Bioinformatics. 2013 Nov 30;30(3):360–8. doi: 10.1093/bioinformatics/btt687 (PMC4023302; doi:10.1093/bioinformatics/btt687)
Supplement: Supplementary Data [file supp_btt687_SupplementaryTable_2.pdf]

Supplementary Table 2. Functional differences between pathways found by GSNCA and GSCA methods.

Tumorogenesis				Monogenic changes in tumors				Signaling pathways				Metabolism			
Tumor signatures		Comparative analysis		Fusions		Gene targets		Cancer treatment		System response		Cellular		Nucleic acids	
GSCA	GSNCA	GSCA	GSNCA	GSCA	GSNCA	GSCA	GSNCA	GSCA	GSNCA	GSCA	GSNCA	GSCA	GSNCA	GSCA	GSNCA
% of pathways found in each category by GSCA/GSNCA															
19.1	23.8	8.5	5.5	4.2	1.5	19.1	23.0	0.0	11.1[1)]	17.0	29.3	21.2[2)]	5.5	10.6	0

[1)] Green color indicates categories with the most pronounced differences between pathways (%), found with GSNCA approach as compared to GSCA.

[2)] Red color indicates categories with the most pronounced differences between pathways (%), found with GSCA approach as compared to GSNCA.

GSCA

Tumor signatures

WONG_ENDMETRIUM_CANCER
BEIER_GLIOMA_STEM_CELL_UP
BOYAULT_LIVER_CANCER_SUBCLASS_G3_DN
WOO_LIVER_CANCER_RECURRENCE_DN
CAIRO_HEPATOBLASTOMA_POOR_SURVIVAL
ZHAN_MULTIPLE_MYELOMA_LB_UP
SATO_SILENCED_BY_METHYLATION_IN_PANCREATIC_CANCER_2
SU_TESTIS
KEGG_COLORECTAL_CANCER

Comparative analysis

CAVARD_LIVER_CANCER_MALIGNANT_VS_BENIGN
WATTEL_AUTONOMOUS_THYROID_ADENOMA_UP
RICKMAN_HEAD_AND_NECK_CANCER_A
ABRAHAM_ALPC_VS_MULTIPLE_MYELOMA_DN

Monogenic changes in tumors

Fusions

SILIGAN_BOUND_BY_EWS_FLT1_FUSION
ROSS_AML_WITH_PML_RARA_FUSION

Gene targets

DACOSTA_UV_RESPONSE_VIA_ERCC3_TTD_UP
ROZANOV_MMP14_TARGETS_SUBSET
KENNY_CTNNB1_TARGETS_UP
SHEPARD_BMYB_TARGETS
KYNG_DNA_DAMAGE_BY_4NQO_OR_UV
YOKOE_CANCER_TESTIS_ANTIGENS
FIRESTEIN_CTNNB1_PATHWAY
CROONQUIST_NRAS_VS_STROMAL_STIMULATION_UP
CUI_TCF21_TARGETS_UP

Signaling

System response

RUGO_STRESS_RESPONSE_SUBSET_G
DORN_ADENOVIRUS_INFECTION_12HR_DN
SASSON_RESPONSE_TO_FORSKOLIN_DN
REACTOME_SIGNALING_BY_BMP
ST_FAS_SIGNALING_PATHWAY
REACTOME_MTOR_SIGNALLING
SIG_REGULATION_OF_THE_ACTIN_CYTOSKELETON_BY_RHO_GTPASES
BAELDE_DIABETIC_NEPHROPATHY_DN

Metabolism

Cellular

KEGG_PHENYLALANINE_METABOLISM
KEGG_SELENOAMINO_ACID_METABOLISM
KEGG_AMINO_SUGAR_AND_NUCLEOTIDE_SUGAR_METABOLISM
KEGG_SPHINGOLIPID_METABOLISM
KEGG_DRUG_METABOLISM_OTHER_ENZYMES
B BIOCARTA_NGF_PATHWAY

BIOCARTA_ERK_PATHWAY
BIOCARTA_NGF_PATHWAY
BIOCARTA_CDC42RAC_PATHWAY
BIOCARTA_ACTINY_PATHWAY
REACTOME_GAP_JUNCTION_TRAFFICKING
Nucleic acids
BIOCARTA_CELLCYCLE_PATHWAY
REACTOME_GENERIC_TRANSCRIPTION_PATHWAY
REACTOME_RNA_POLYMERASE_III_TRANSCRIPTION
REACTOME_RNA_POLYMERASE_III_TRANSCRIPTION_INITIATION
REACTOME_RNA_POLYMERASE_III_TRANSCRIPTION_INITIATION_FROM_TYPE_3_PROMOTER

GSNCA
Cancer treatment
WATANABE_RECTAL_CANCER_RADIOTHERAPY_RESPONSIVE_UP
ZHONG_RESPONSE_TO_AZACITIDINE_AND_TSA_UP
KAN_RESPONSE_TO_ARSENIC_TRIOXIDE
RASHI_RESPONSE_TO_IONIZING_RADIATION_2
MASRI_RESISTANCE_TO_TAMOXIFEN_AND_AROMATASE_INHIBITORS_UP
HONMA_DOCETAXEL_RESISTANCE
DAZARD_UV_RESPONSE_CLUSTER_G24
GAJATE_RESPONSE_TO_TRABECTEDIN_DN
GAJATE_RESPONSE_TO_TRABECTEDIN_UP
MARTINEZ_RESPONSE_TO_TRABECTEDIN_DN
GENTILE_UV_LOW_DOSE_DN
GENTILE_UV_LOW_DOSE_UP
MURAKAMI_UV_RESPONSE_6HR_DN
BIOCARTA_GLEEVEC_PATHWAY
Tumor signatures
LU_TUMOR_VASCULATURE_UP
LIU_PROSTATE_CANCER_UP
SENGUPTA_NASOPHARYNGEAL_CARCINOMA_DN

WAMUNYOKOLI_OVARIAN_CANCER_LMP_DN
DELYS_THYROID_CANCER_DN
WANG_BARRETTS_ESOPHAGUS_AND_ESOPHAGUS_CANCER_DN
WANG_BARRETTS_ESOPHAGUS_UP
MCBRYAN_PUBERTAL_BREAST_3_4WK_UP
MCBRYAN_PUBERTAL_BREAST_4_5WK_UP
HWANG_PROSTATE_CANCER_MARKERS
LINDGREN_BLADDER_CANCER_HIGH_RECURRENCE
KORKOLA_YOLK_SAC_TUMOR
WANG_HCP_PROSTATE_CANCER
PASQUALUCCI_LYMPHOMA_BY_GC_STAGE_UP
LEE_LIVER_CANCER_E2F1_UP
HOFMANN_CELL_LYMPHOMA_DN
KONDO_PROSTATE_CANCER_HCP_WITH_H3K27ME3
YAMASHITA_METHYLATED_IN_PROSTATE_CANCER
ENGELMANN_CANCER_PROGENITORS_UP
SMID_BREAST_CANCER_RELAPSE_IN_BONE_DN
SMID_BREAST_CANCER_ERBB2_UP
GRADE_COLON_AND_RECTAL_CANCER_DN
SHARMA_PILOCYTIC_ASTROCYTOMA_LOCATION_UP
LEE_EARLY_T_LYMPHOCYTE_DN
VALK_AML_CLUSTER_8
BOYAULT_LIVER_CANCER_SUBCLASS_G1_DN
BOYAULT_LIVER_CANCER_SUBCLASS_G2
CHIANG_LIVER_CANCER_SUBCLASS_CTNNB1_DN
RAMASWAMY_METASTASIS_DN
KEGG_RENAL_CELL_CARCINOMA

Comparative analysis

TURASHVILI_BREAST_DUCTAL_CARCINOMA_VS_DUCTAL_NORMAL_DN
TURASHVILI_BREAST_LOBULAR_CARCINOMA_VS_DUCTAL_NORMAL_UP

CHARAFE_BREAST_CANCER_LUMINAL_VS_BASAL_DN
CHARAFE_BREAST_CANCER_LUMINAL_VS_MESENCHYMAL_DN
OUELLET_CULTURED_OVARIAN_CANCER_INVASIVE_VS_LMP_DN
LIEN_BREAST_CARCINOMA_METAPLASTIC_VS_DUCTAL_UP
IZADPANAH_STEM_CELL_ADIPOSE_VS_BONE_DN

Monogenic changes in tumors

Fusions

NIKOLSKY_BREAST_CANCER_16P13_AMPLICON
FAELT_B_CLL_WITH_VH_REARRANGEMENTS_DN

Gene targets

CHEMNITZ_RESPONSE_TO_PROSTAGLANDIN_E2_DN
HOOI_ST7_TARGETS_UP
HORIUCHI_WTAP_TARGETS_UP
SENESE_HDAC1_AND_HDAC2_TARGETS_UP
SENESE_HDAC1_AND_HDAC2_TARGETS_DN
SENESE_HDAC3_TARGETS_DN
BERENJENO_TRANSFORMED_BY_RHOA_DN
TANG_SENESCENCE_TP53_TARGETS_UP
RODRIGUES_NTN1_AND_DCC_TARGETS
FURUKAWA_DUSP6_TARGETS_PCI35_UP
WILLIAMS_ESR1_TARGETS_UP
NUYTTEN_NIPP1_TARGETS_UP
JEON_SMAD6_TARGETS_UP
ONDER_CDH1_TARGETS_2_DN
ONDER_CDH1_TARGETS_3_DN
CERVERA_SDHB_TARGETS_1_UP
DORSAM_HOXA9_TARGETS_DN
YAO_HOXA10_TARGETS_VIA_PROGESTERONE_DN
DORSEY_GAB2_TARGETS
MCCABE_BOUND_BY_HOXC6

BRUECKNER_TARGETS_OF_MIRLET7A3_DN
GU_PDEF_TARGETS_UP
WANG_SMARCE1_TARGETS_UP
MCCLUNG_DELTA_FOSB_TARGETS_8WK
XU_GH1_EXOGENOUS_TARGETS_DN
WU_HBX_TARGETS_1_DN
WU_HBX_TARGETS_1_UP
WU_HBX_TARGETS_2_DN
PAL_PRMT5_TARGETS_DN

System response

GAZDA_DIAMOND_BLACKFAN_ANEMIA_PROGENITOR_DN
GAZDA_DIAMOND_BLACKFAN_ANEMIA_ERYTHROID_UP
FULCHER_INFLAMMATORY_RESPONSE_LECTIN_VS_LPS_UP
DOANE_RESPONSE_TO_ANDROGEN_UP
SMIRNOV_CIRCULATING_ENDOTHELIOCYTES_IN_CANCER_UP
HOEBEKE_LYMPHOID_STEM_CELL_UP
HU_ANGIOGENESIS_DN
DUTTA_APOPTOSIS_VIA_NFKB
SCHEIDEREIT_IKK_INTERACTING_PROTEINS
SCHLESINGER_METHYLATED_DE_NOVO_IN_CANCER
WU_CELL_MIGRATION
ONDER_CDH1_SIGNALING_VIA_CTNNB1
UEDA_CENTRAL_CLOCK
WILLERT_WNT_SIGNALING
BREDEMEYER_RAG_SIGNALING_NOT_VIA_ATM_UP
CAIRO_LIVER_DEVELOPMENT_DN
BROWNE_HCMV_INFECTION_30MIN_UP
BROWNE_HCMV_INFECTION_4HR_UP
KAAB_HEART_ATRIUM_VS_VENTRICLE_UP
BROWNE_HCMV_INFECTION_24HR_DN

WEIGEL_OXIDATIVE_STRESS_BY_HNE_AND_TBH
SATO_SILENCED_EPIGENETICALLY_IN_PANCREATIC_CANCER
KEGG_MAPK_SIGNALING_PATHWAY
BIOCARTA_CERAMIDE_PATHWAY
BIOCARTA_CTCF_PATHWAY
REACTOME_CELL_CELL_ADHESION_SYSTEMS
REACTOME_CYCLIN_A1_ASSOCIATED_EVENTS_DURING_G2_M_TRANSITION
ST_JNK_MAPK_PATHWAY
REACTOME_CELL_JUNCTION_ORGANIZATION
BIOCARTA_TEL_PATHWAY
REACTOME_CYCLIN_A1_ASSOCIATED_EVENTS_DURING_G2_M_TRANSITION
REACTOME_INTRINSIC_PATHWAY_FOR_APOPTOSIS
REACTOME_MICRORNA_BIOGENESIS
REACTOME_PHASE_II_CONJUGATION
REACTOME_TOLL_LIKE_RECEPTOR_3_CASCADE
REACTOME_TRAF6_MEDIATED_INDUCTION_OF_THE_ANTIVIRAL_CYTOKINE_IFN_ALPHA_BETA_CASCADE
REACTOME_UNFOLDED_PROTEIN_RESPONSE
REACTOME_MAP_KINASES_ACTIVATION_IN_TLR_CASCADE

General metabolism

MOOTHA_MITOCHONDRIA
KEGG_CITRATE_CYCLE_TCA_CYCLE
KEGG_PEROXISOME
BIOCARTA_MITOCHONDRIA_PATHWAY
REACTOME_LYSOSOME_VESICLE_BIOGENESIS
REACTOME_MEMBRANE_TRAFFICKING
