# Supplementary material for: Gene Sets Net Correlations Analysis (GSNCA): a multivariate differential coexpression test for gene sets
Source: Bioinformatics. 2013 Nov 30;30(3):360–8. doi: 10.1093/bioinformatics/btt687 (PMC4023302; doi:10.1093/bioinformatics/btt687)

Pathway: VECCHI_GASTRIC_CANCER_ADVANCED_VS_EARLY_UP

There are 107 genes in this pathway. This pathway was detected by Both

WT p53
Hub Gene (WT): AXL
Weight Factor: 1.471
Hub Gene (MUT): LOX
Weight Factor: 1.296

MUT p53
Hub Gene (MUT): LOX
Weight Factor: 1.541
Hub Gene (WT): AXL
Weight Factor: 1.321

MST2 of the coexpression network for WT p53

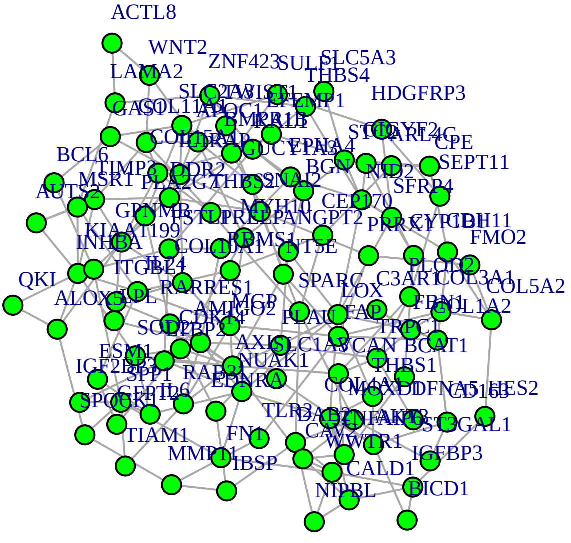

MST2 of the coexpression network for MUT p53

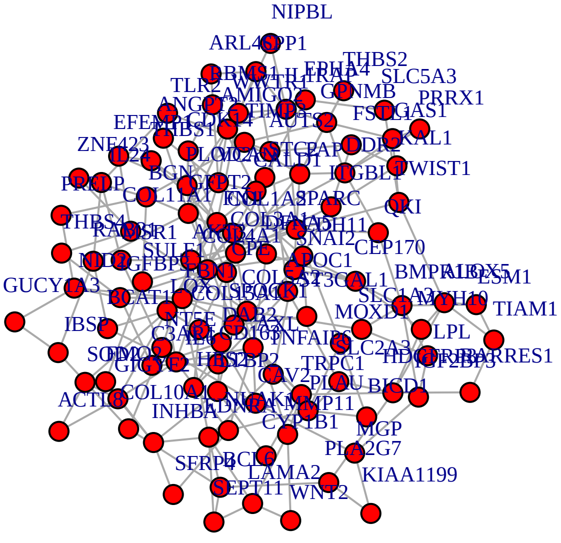

Pathway: PETRETTO_CARDIAC_HYPERTROPHY

There are 29 genes in this pathway. This pathway was detected by Both

WT p53
Hub Gene (WT): TM4SF1
Weight Factor: 1.343
Hub Gene (MUT): FBN1
Weight Factor: 1.039

MUT p53
Hub Gene (MUT): FBN1
Weight Factor: 1.411
Hub Gene (WT): TM4SF1
Weight Factor: 0.91

MST2 of the coexpression network for WT p53

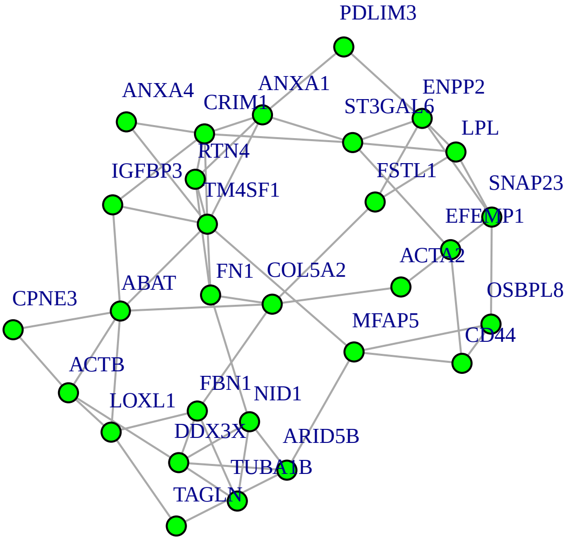

MST2 of the coexpression network for MUT p53

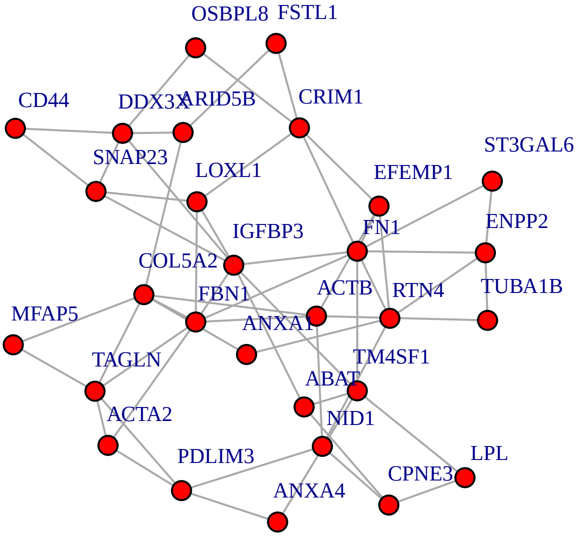

Pathway: SHEPARD_BMYB_MORPHOLINO_UP

There are 107 genes in this pathway. This pathway was detected by Both

WT p53
Hub Gene (WT): MBD2
Weight Factor: 1.391
Hub Gene (MUT): MMP2
Weight Factor: 0.848

MUT p53
Hub Gene (MUT): MMP2
Weight Factor: 1.482
Hub Gene (WT): MBD2
Weight Factor: 0.914

MST2 of the coexpression network for WT p53

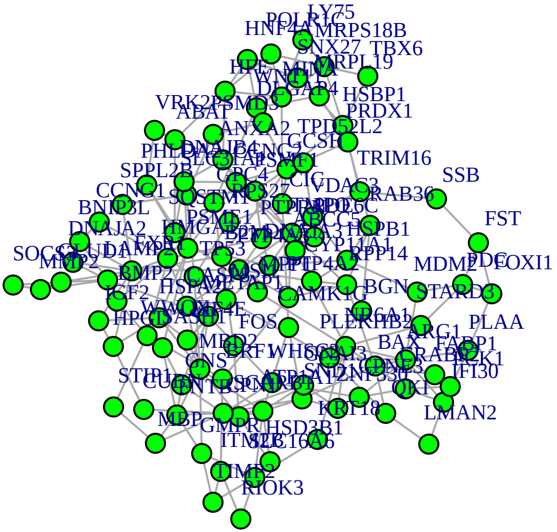

MST2 of the coexpression network for MUT p53

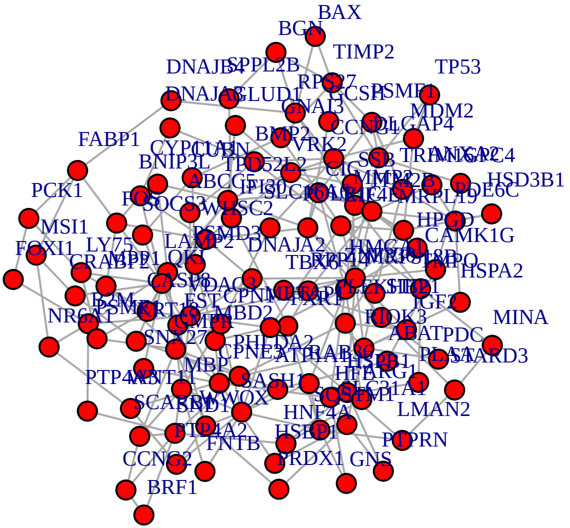

Pathway: KANG_IMMORTALIZED_BY_TERT_UP

There are 78 genes in this pathway. This pathway was detected by Both

WT p53
Hub Gene (WT): BDNF
Weight Factor: 1.332
Hub Gene (MUT): COL6A2
Weight Factor: 1.037

MUT p53
Hub Gene (MUT): COL6A2
Weight Factor: 1.528
Hub Gene (WT): BDNF
Weight Factor: 1.193

MST2 of the coexpression network for WT p53

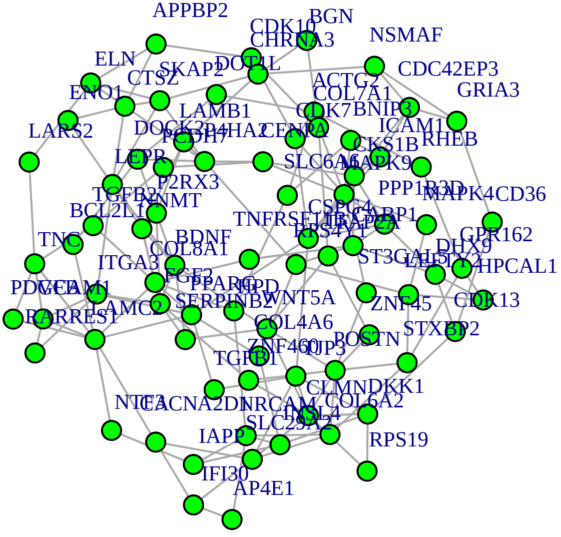

MST2 of the coexpression network for MUT p53

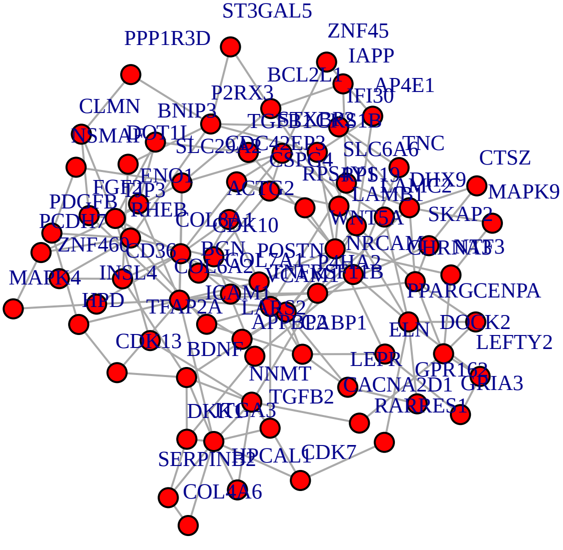

Pathway: CONRAD_STEM_CELL

There are 24 genes in this pathway. This pathway was detected by Both

WT p53
Hub Gene (WT): TTLL4
Weight Factor: 1.309
Hub Gene (MUT): KLF5
Weight Factor: 0.959

MUT p53
Hub Gene (MUT): KLF5
Weight Factor: 1.341
Hub Gene (WT): TTLL4
Weight Factor: 1.084

MST2 of the coexpression network for WT p53

MST2 of the coexpression network for MUT p53

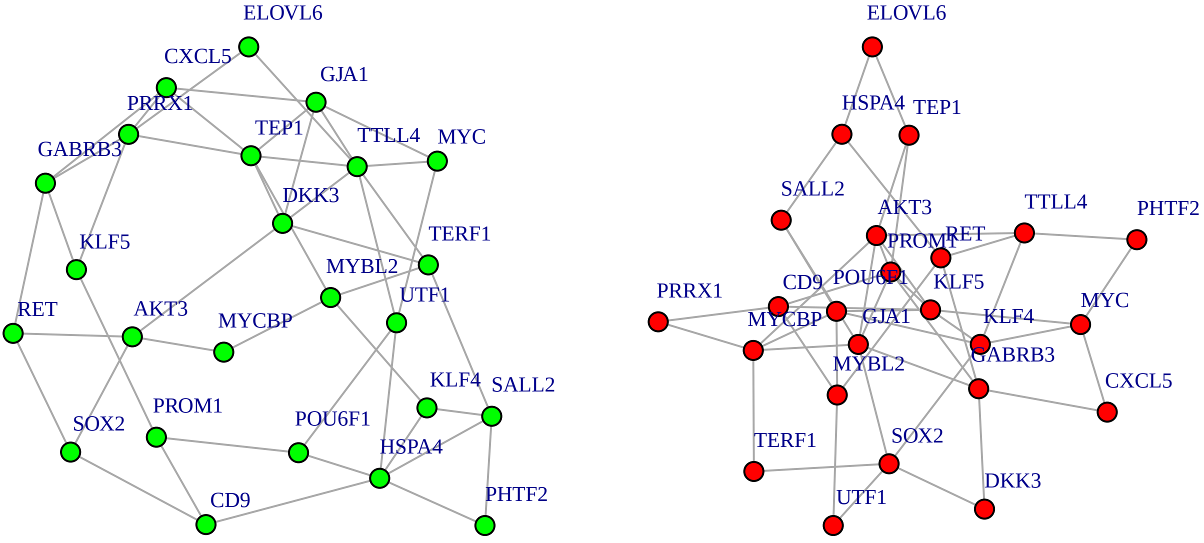

Pathway: BOYAULT_LIVER_CANCER_SUBCLASS_G6_DN

There are 15 genes in this pathway. This pathway was detected by Both

WT p53
Hub Gene (WT): RFTN1
Weight Factor: 1.32
Hub Gene (MUT): CDH1
Weight Factor: 1.175

MUT p53
Hub Gene (MUT): CDH1
Weight Factor: 1.485
Hub Gene (WT): RFTN1
Weight Factor: 1.225

MST2 of the coexpression network for WT p53

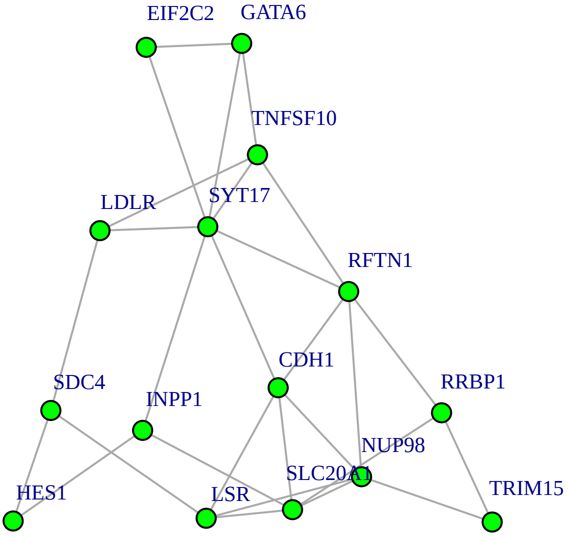

MST2 of the coexpression network for MUT p53

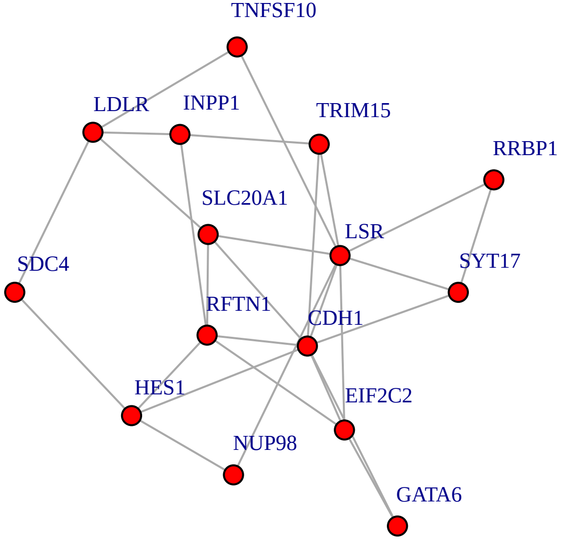

Pathway: ZHAN_MULTIPLE_MYELOMA_CD1_DN

There are 24 genes in this pathway. This pathway was detected by Both

WT p53
Hub Gene (WT): PRKD3
Weight Factor: 1.346
Hub Gene (MUT): SPINT1
Weight Factor: 0.78

MUT p53
Hub Gene (MUT): SPINT1
Weight Factor: 1.434
Hub Gene (WT): PRKD3
Weight Factor: 0.787

MST2 of the coexpression network for WT p53

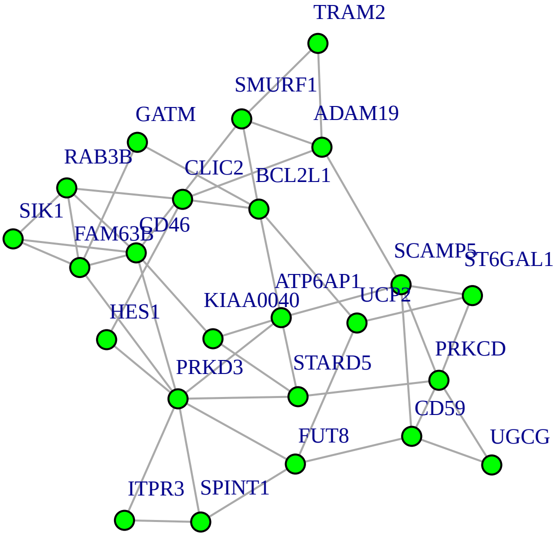

MST2 of the coexpression network for MUT p53

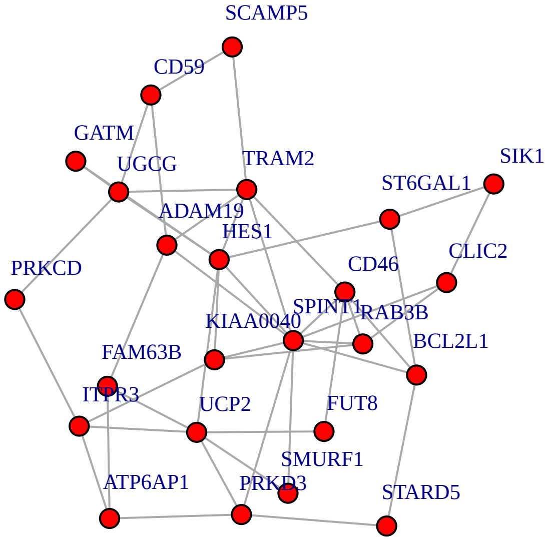

Pathway: SASSON_RESPONSE_TO_GONADOTROPHINS_DN

There are 68 genes in this pathway. This pathway was detected by Both

WT p53
Hub Gene (WT): ACP5
Weight Factor: 1.351
Hub Gene (MUT): AXL
Weight Factor: 1.214

MUT p53
Hub Gene (MUT): AXL
Weight Factor: 1.473
Hub Gene (WT): ACP5
Weight Factor: 0.783

MST2 of the coexpression network for WT p53

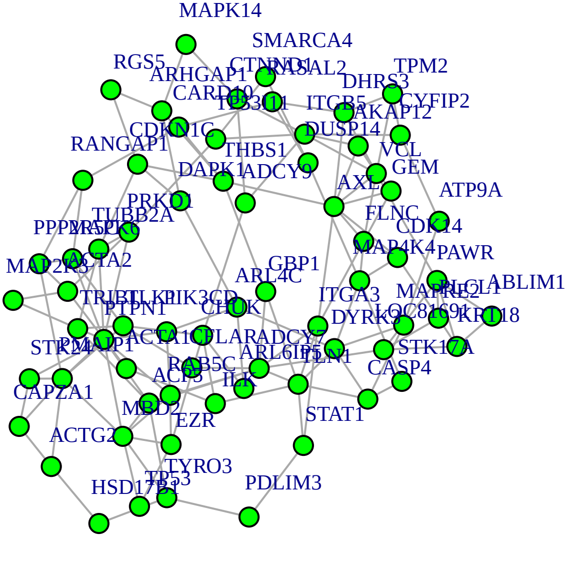

MST2 of the coexpression network for MUT p53

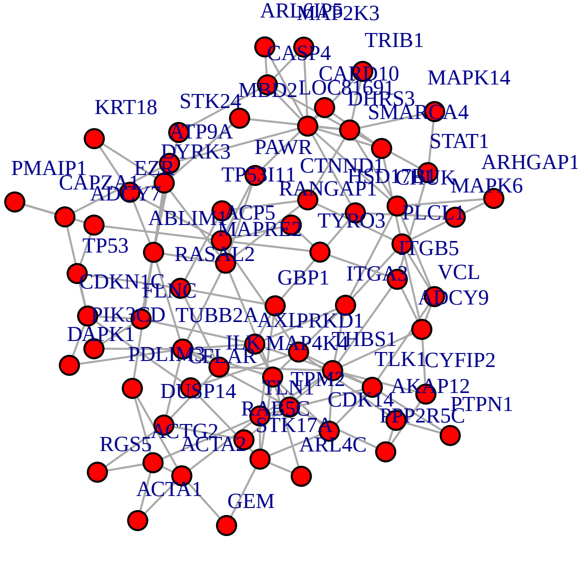

Pathway: SATO_SILENCED_BY_METHYLATION_IN_PANCREATIC_CANCER_1

There are 305 genes in this pathway. This pathway was detected by Both

WT p53
Hub Gene (WT): CITED1
Weight Factor: 1.371
Hub Gene (MUT): SPARC
Weight Factor: 1.175

MUT p53
Hub Gene (MUT): SPARC
Weight Factor: 1.47
Hub Gene (WT): CITED1
Weight Factor: 0.967

MST2 of the coexpression network for WT p53

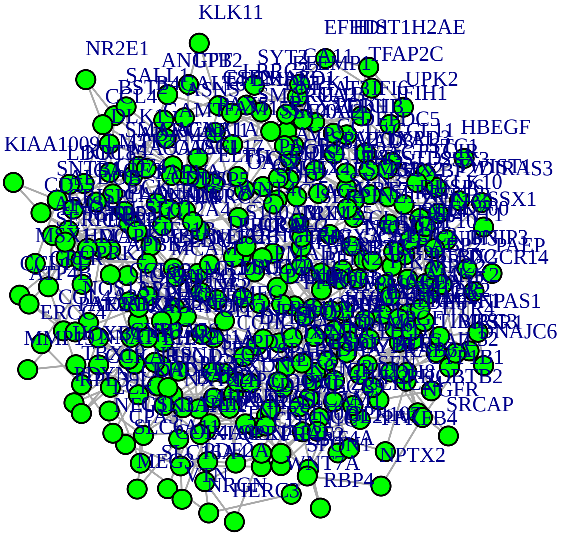

MST2 of the coexpression network for MUT p53

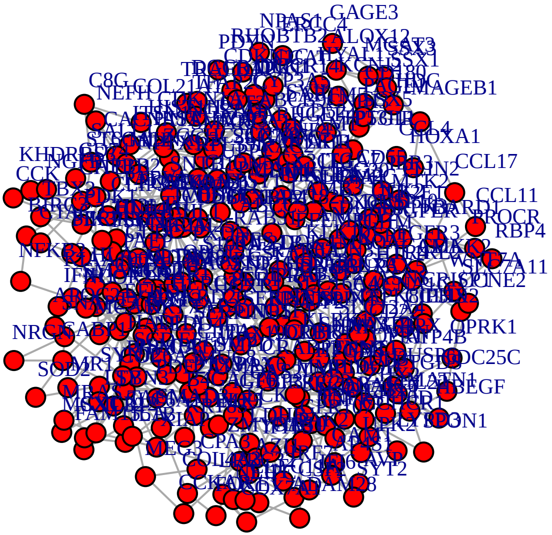

Pathway: NATSUME_RESPONSE_TO_INTERFERON_BETA_UP

There are 62 genes in this pathway. This pathway was detected by Both

WT p53
Hub Gene (WT): IRF1
Weight Factor: 1.455
Hub Gene (MUT): FOSL2
Weight Factor: 0.938

MUT p53
Hub Gene (MUT): FOSL2
Weight Factor: 1.504
Hub Gene (WT): IRF1
Weight Factor: 1.093

MST2 of the coexpression network for WT p53

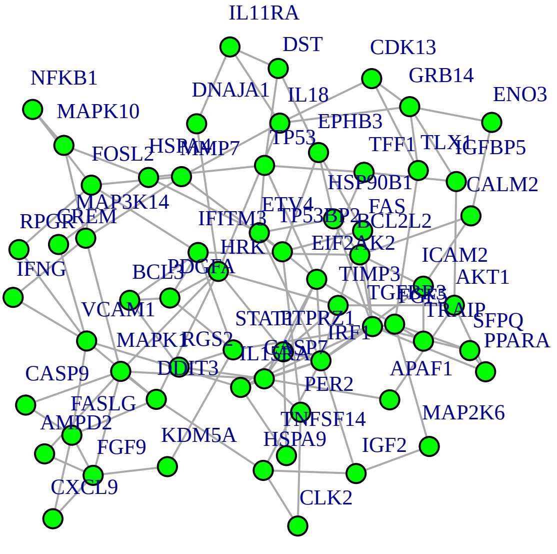

MST2 of the coexpression network for MUT p53

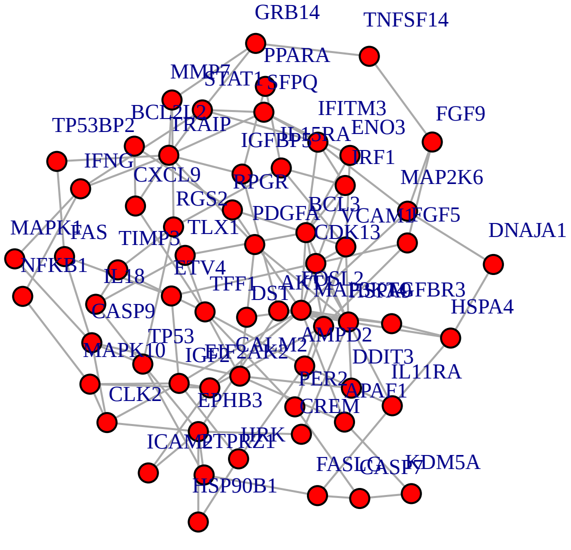

Pathway: KEGG_EPITHELIAL_CELL_SIGNALING_IN_HELICOBACTER_PYLORI_INFECTION

There are 58 genes in this pathway. This pathway was detected by Both

WT p53
Hub Gene (WT): ATP6V1E1
Weight Factor: 1.379
Hub Gene (MUT): ATP6V1A
Weight Factor: 0.872

MUT p53
Hub Gene (MUT): ATP6V1A
Weight Factor: 1.44
Hub Gene (WT): ATP6V1E1
Weight Factor: 0.966

MST2 of the coexpression network for WT p53

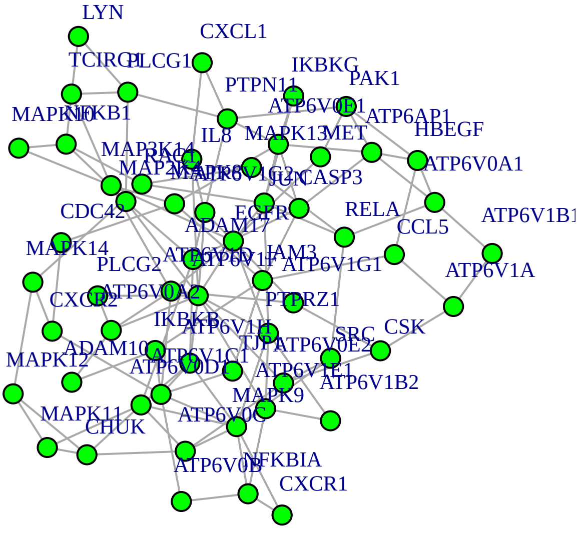

MST2 of the coexpression network for MUT p53

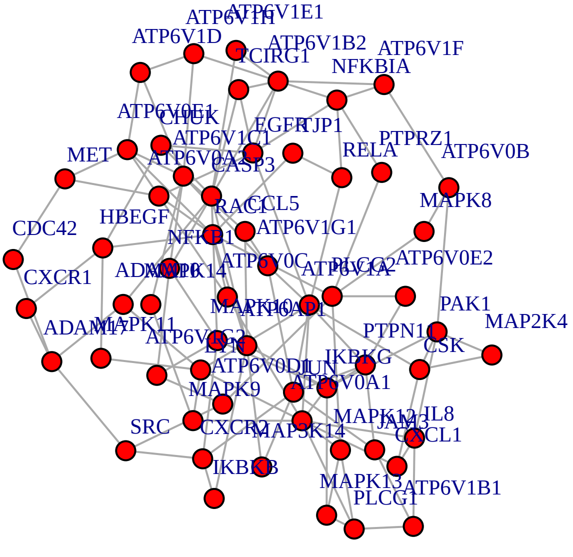

Pathway: BIOCARTA_PGC1A_PATHWAY

There are 20 genes in this pathway. This pathway was detected by Both

WT p53
Hub Gene (WT): MEF2D
Weight Factor: 1.235
Hub Gene (MUT): CAMK2A
Weight Factor: 0.793

MUT p53
Hub Gene (MUT): CAMK2A
Weight Factor: 1.291
Hub Gene (WT): MEF2D
Weight Factor: 0.817

MST2 of the coexpression network for WT p53

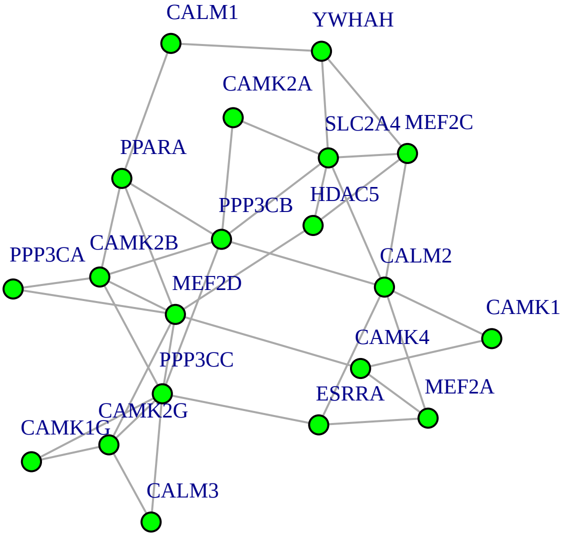

MST2 of the coexpression network for MUT p53

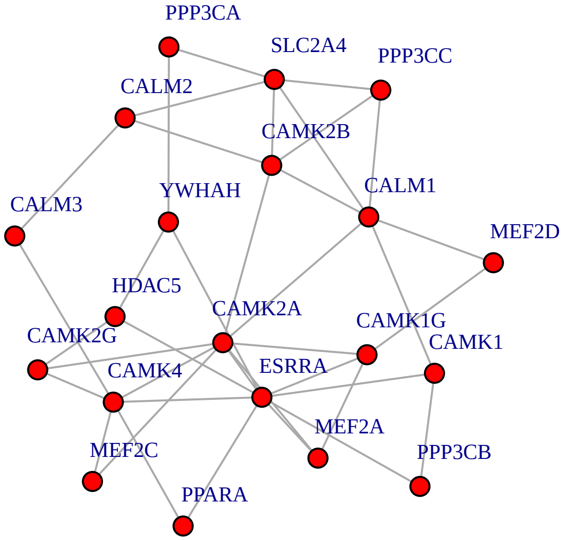

Pathway: LU_TUMOR_VASCULATURE_UP

There are 22 genes in this pathway. This pathway was detected by GSNCA

WT p53
Hub Gene (WT): TNFAIP6
Weight Factor: 1.362
Hub Gene (MUT): VCAN
Weight Factor: 1.165

MUT p53
Hub Gene (MUT): VCAN
Weight Factor: 1.465
Hub Gene (WT): TNFAIP6
Weight Factor: 0.979

MST2 of the coexpression network for WT p53

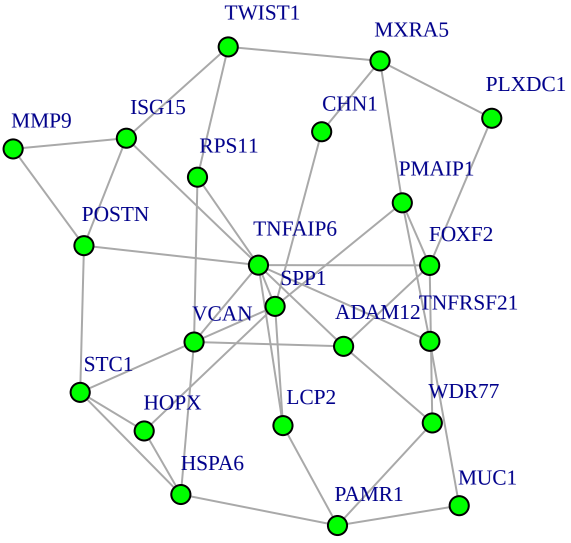

MST2 of the coexpression network for MUT p53

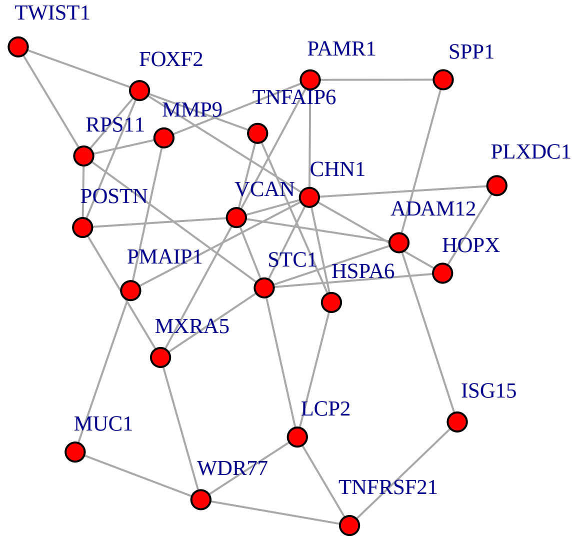

Pathway: WATANABE_RECTAL_CANCER_RADIOTHERAPY_RESPONSIVE_UP

There are 100 genes in this pathway. This pathway was detected by GSNCA

WT p53
Hub Gene (WT): RRM1
Weight Factor: 1.436
Hub Gene (MUT): CDH1
Weight Factor: 0.88

MUT p53
Hub Gene (MUT): CDH1
Weight Factor: 1.431
Hub Gene (WT): RRM1
Weight Factor: 1.023

MST2 of the coexpression network for WT p53

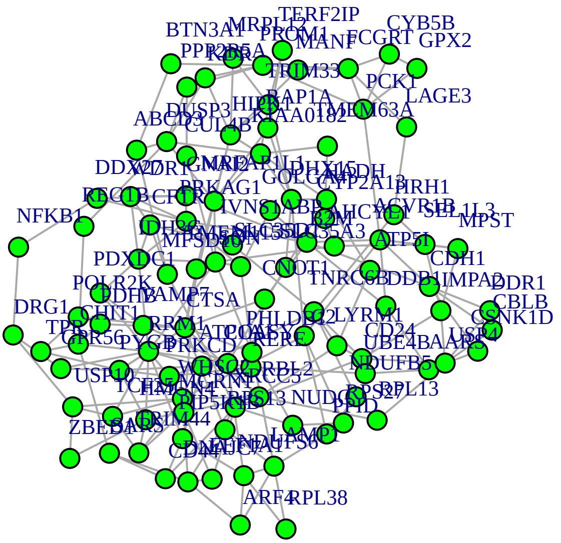

MST2 of the coexpression network for MUT p53

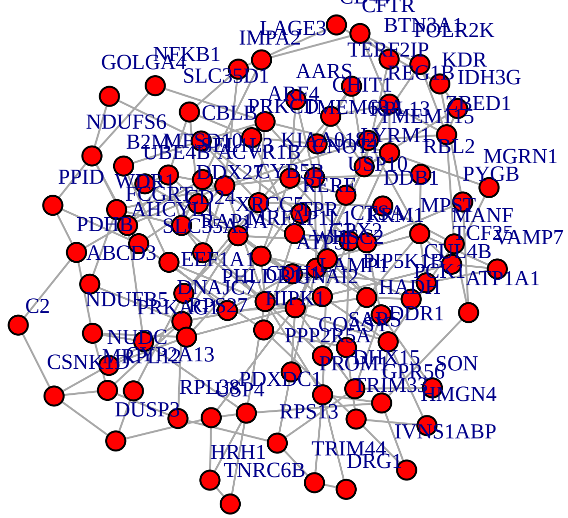

Pathway: LIU_PROSTATE_CANCER_UP

There are 56 genes in this pathway. This pathway was detected by GSNCA

WT p53
Hub Gene (WT): GJB1
Weight Factor: 1.318
Hub Gene (MUT): EPCAM
Weight Factor: 1.017

MUT p53
Hub Gene (MUT): EPCAM
Weight Factor: 1.579
Hub Gene (WT): GJB1
Weight Factor: 0.821

MST2 of the coexpression network for WT p53

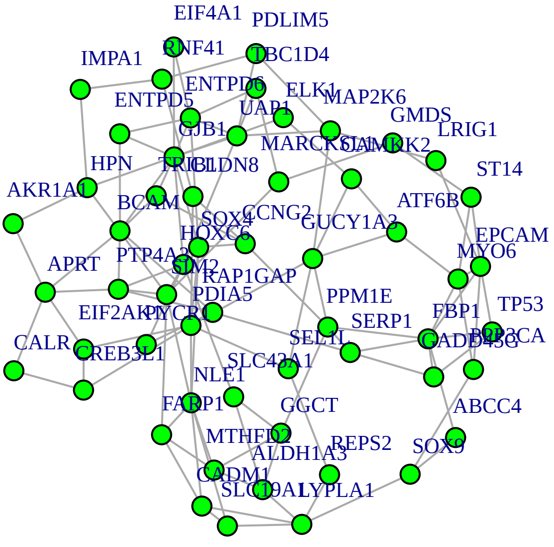

MST2 of the coexpression network for MUT p53

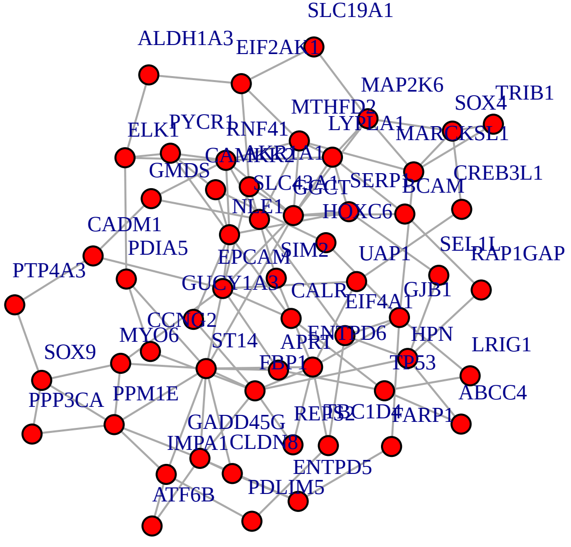

Pathway: SENGUPTA_NASOPHARYNGEAL_CARCINOMA_DN

There are 120 genes in this pathway. This pathway was detected by GSNCA

WT p53
Hub Gene (WT): ALDH3B1
Weight Factor: 1.392
Hub Gene (MUT): TJP3
Weight Factor: 1.197

MUT p53
Hub Gene (MUT): TJP3
Weight Factor: 1.649
Hub Gene (WT): ALDH3B1
Weight Factor: 1.193

MST2 of the coexpression network for WT p53

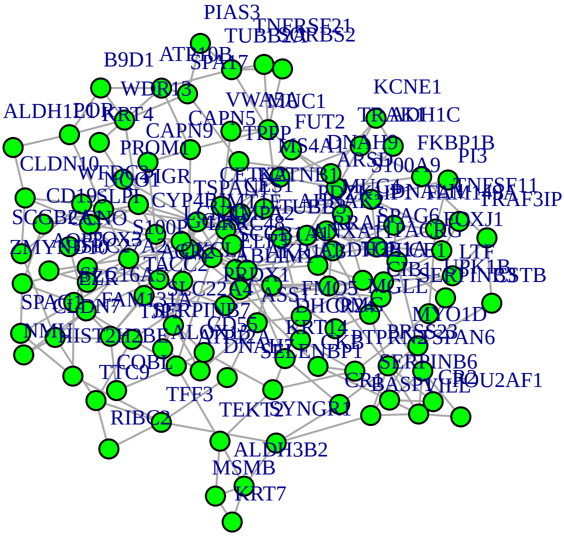

MST2 of the coexpression network for MUT p53

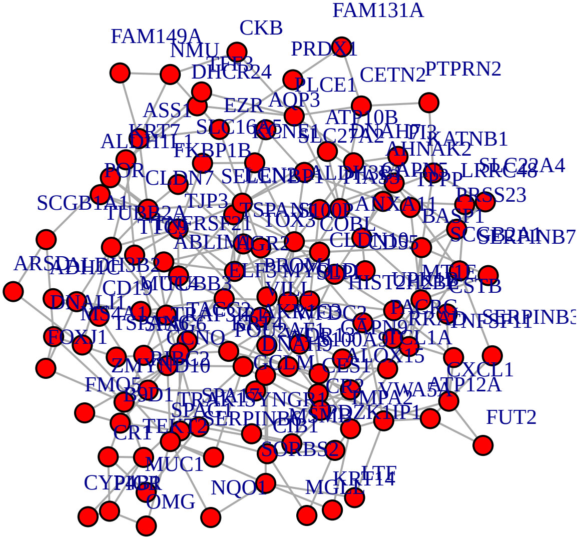

Pathway: GAZDA_DIAMOND_BLACKFAN_ANEMIA_ERYTHROID_UP

There are 18 genes in this pathway. This pathway was detected by GSNCA

WT p53
Hub Gene (WT): DPY19L1
Weight Factor: 1.376
Hub Gene (MUT): SEPHS2
Weight Factor: 0.771

MUT p53
Hub Gene (MUT): SEPHS2
Weight Factor: 1.51
Hub Gene (WT): DPY19L1
Weight Factor: 0.75

MST2 of the coexpression network for WT p53

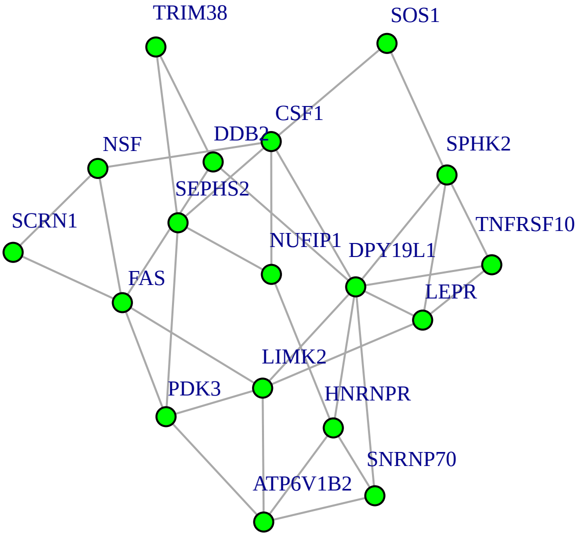

MST2 of the coexpression network for MUT p53

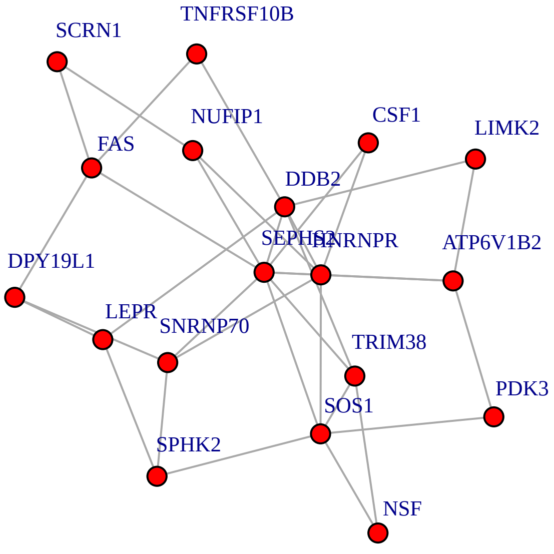

Pathway: GAZDA_DIAMOND_BLACKFAN_ANEMIA_PROGENITOR_DN

There are 47 genes in this pathway. This pathway was detected by GSNCA

WT p53		MUT p53	
**Hub Gene (WT):**	**NFYB**	**Hub Gene (MUT):**	**LYPLA1**
**Weight Factor:**	**1.347**	**Weight Factor:**	**1.412**
**Hub Gene (MUT):**	**LYPLA1**	**Hub Gene (WT):**	**NFYB**
**Weight Factor:**	**0.809**	**Weight Factor:**	**0.856**

MST2 of the coexpression network for WT p53

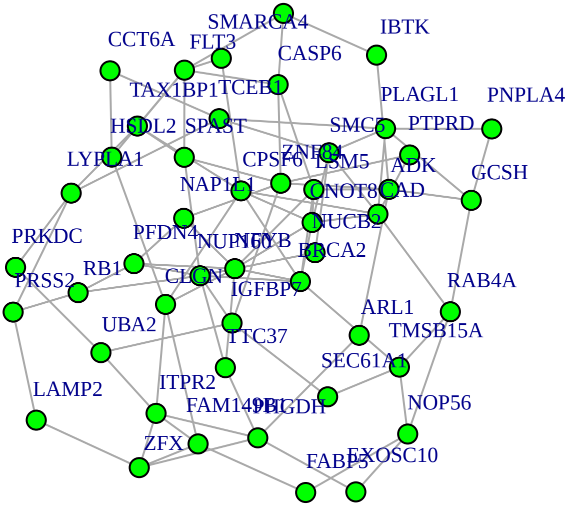

MST2 of the coexpression network for MUT p53

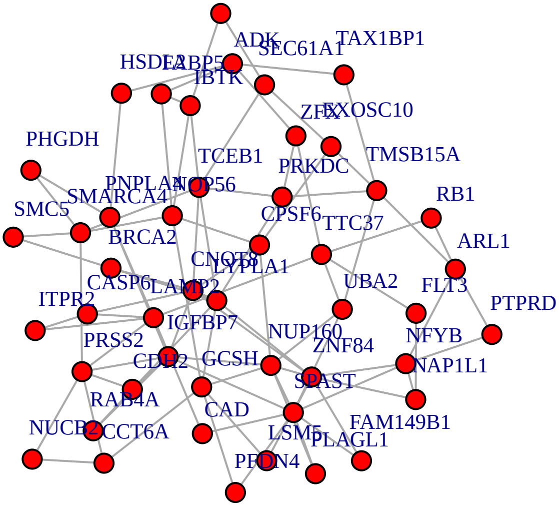

Pathway: CHEMNITZ_RESPONSE_TO_PROSTAGLANDIN_E2_DN

There are 188 genes in this pathway. This pathway was detected by GSNCA

WT p53
Hub Gene (WT): PPL
Weight Factor: 1.319
Hub Gene (MUT): CLDN7
Weight Factor: 1.124

MUT p53
Hub Gene (MUT): CLDN7
Weight Factor: 1.624
Hub Gene (WT): PPL
Weight Factor: 1.442

MST2 of the coexpression network for WT p53

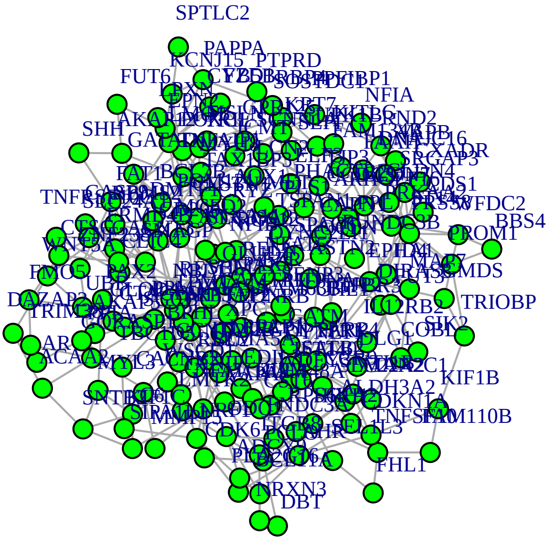

MST2 of the coexpression network for MUT p53

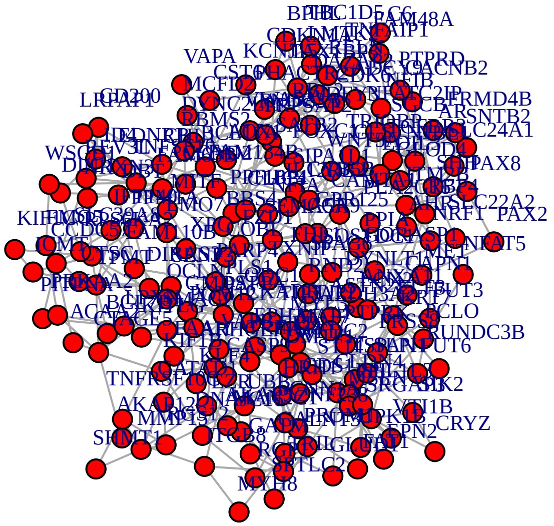

Pathway: ZHONG_RESPONSE_TO_AZACITIDINE_AND_TSA_UP

There are 138 genes in this pathway. This pathway was detected by GSNCA

WT p53
Hub Gene (WT): DGKZ
Weight Factor: 1.343
Hub Gene (MUT): PTRF
Weight Factor: 1.101

MUT p53
Hub Gene (MUT): PTRF
Weight Factor: 1.512
Hub Gene (WT): DGKZ
Weight Factor: 1.08

MST2 of the coexpression network for WT p53

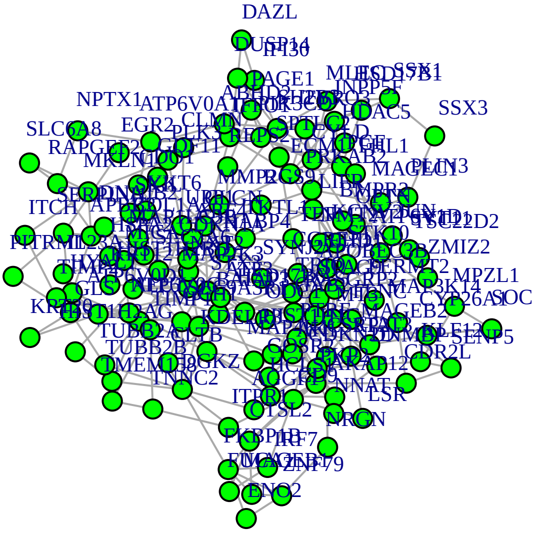

MST2 of the coexpression network for MUT p53

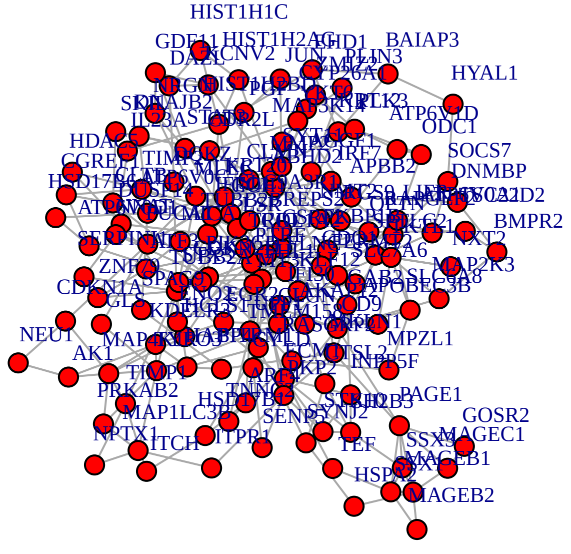

Pathway: TURASHVILI_BREAST_DUCTAL_CARCINOMA_VS_DUCTAL_NORMAL_DN

There are 125 genes in this pathway. This pathway was detected by GSNCA

WT p53	MUT p53
**Hub Gene (WT): PER1**	**Hub Gene (MUT): MYLK**
**Weight Factor: 1.401**	**Weight Factor: 1.513**
**Hub Gene (MUT): MYLK**	**Hub Gene (WT): PER1**
**Weight Factor: 1.092**	**Weight Factor: 0.963**

MST2 of the coexpression network for WT p53

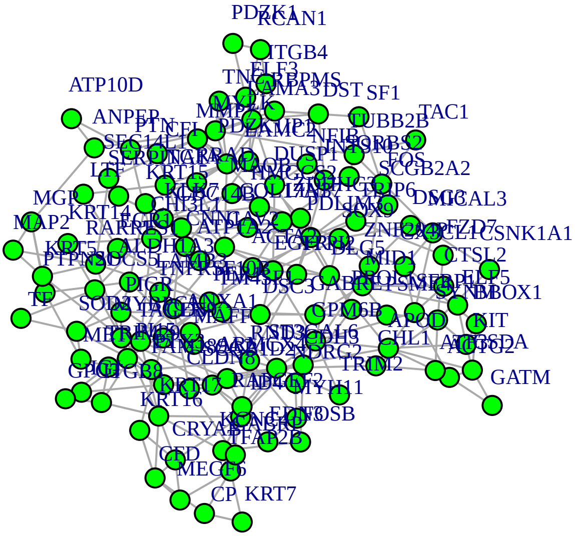

MST2 of the coexpression network for MUT p53

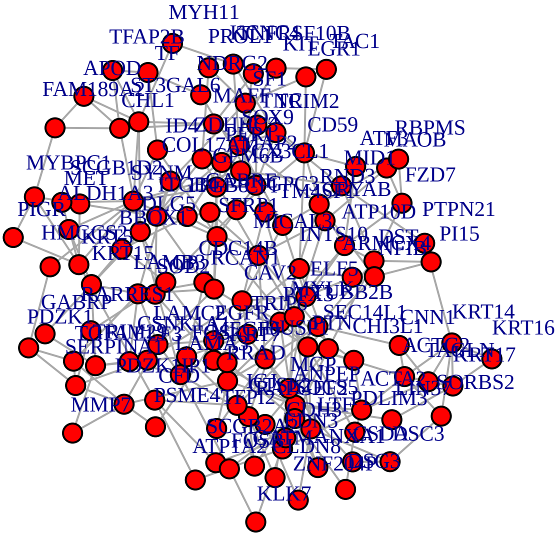

Pathway: TURASHVILI_BREAST_LOBULAR_CARCINOMA_VS_DUCTAL_NORMAL_UP

There are 48 genes in this pathway. This pathway was detected by GSNCA

WT p53
Hub Gene (WT): LRRC15
Weight Factor: 1.364
Hub Gene (MUT): LOX
Weight Factor: 1.226

MUT p53
Hub Gene (MUT): LOX
Weight Factor: 1.555
Hub Gene (WT): LRRC15
Weight Factor: 0.922

MST2 of the coexpression network for WT p53

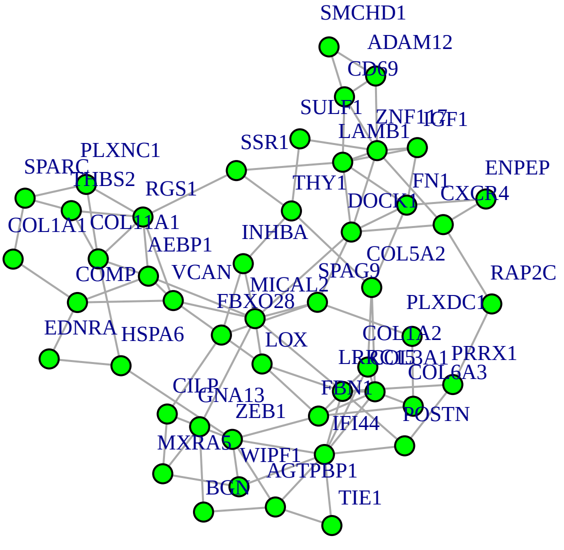

MST2 of the coexpression network for MUT p53

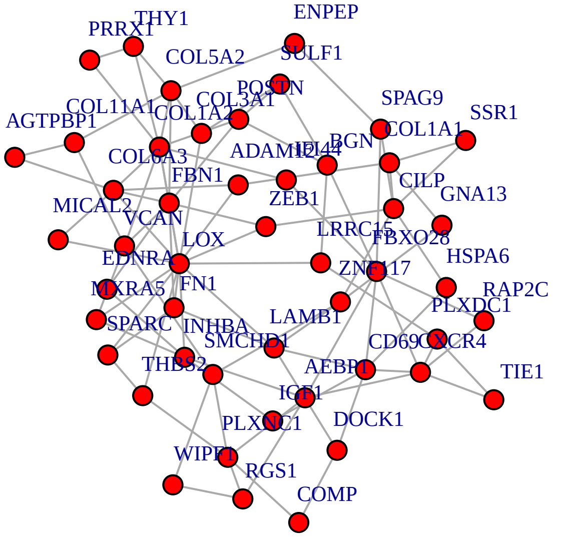

Pathway: TURASHVILI_BREAST_LOBULAR_CARCINOMA_VS_LOBULAR_NORMAL_UP

There are 59 genes in this pathway. This pathway was detected by GSNCA

WT p53
Hub Gene (WT): EGFR
Weight Factor: 1.398
Hub Gene (MUT): CDH1
Weight Factor: 0.873

MUT p53
Hub Gene (MUT): CDH1
Weight Factor: 1.532
Hub Gene (WT): EGFR
Weight Factor: 1.022

MST2 of the coexpression network for WT p53

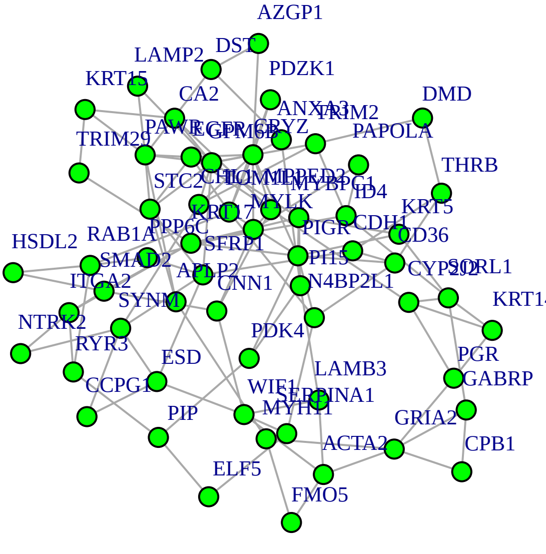

MST2 of the coexpression network for MUT p53

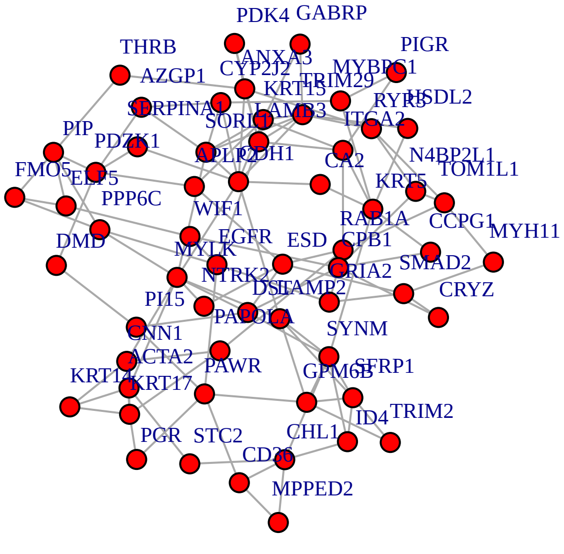

Pathway: FULCHER_INFLAMMATORY_RESPONSE_LECTIN_VS_LPS_UP

There are 346 genes in this pathway. This pathway was detected by GSNCA

WT p53		MUT p53	
**Hub Gene (WT):**	**CRIM1**	**Hub Gene (MUT):**	**FN1**
**Weight Factor:**	**1.305**	**Weight Factor:**	**1.614**
**Hub Gene (MUT):**	**FN1**	**Hub Gene (WT):**	**CRIM1**
**Weight Factor:**	**1.037**	**Weight Factor:**	**1.391**

MST2 of the coexpression network for WT p53

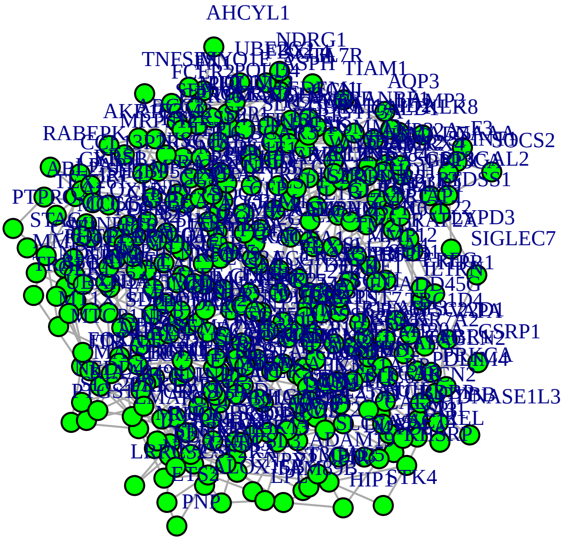

MST2 of the coexpression network for MUT p53

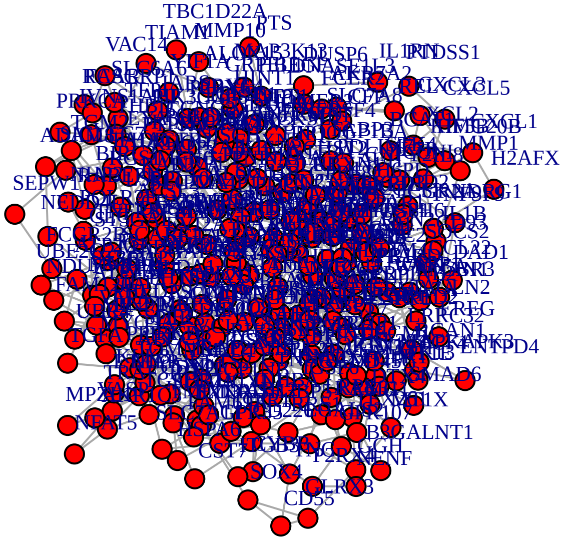

Pathway: HOOI_ST7_TARGETS_UP

There are 50 genes in this pathway. This pathway was detected by GSNCA

WT p53
Hub Gene (WT): FLNC
Weight Factor: 1.222
Hub Gene (MUT): SERPINE1
Weight Factor: 1.208

MUT p53
Hub Gene (MUT): SERPINE1
Weight Factor: 1.509
Hub Gene (WT): FLNC
Weight Factor: 1.172

MST2 of the coexpression network for WT p53

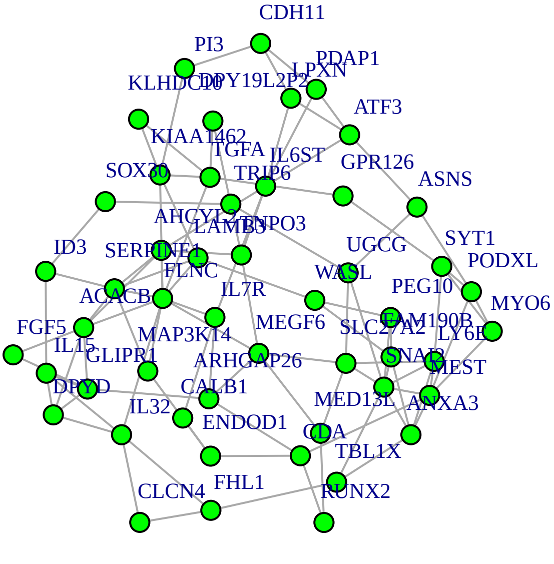

MST2 of the coexpression network for MUT p53

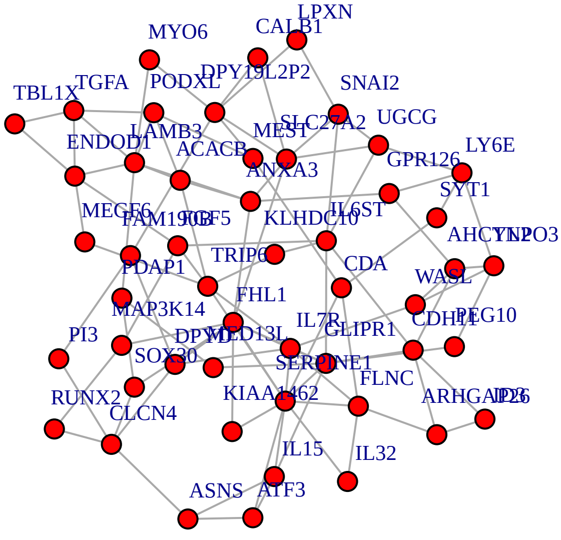

Pathway: CHARAFE_BREAST_CANCER_LUMINAL_VS_BASAL_DN

There are 309 genes in this pathway. This pathway was detected by GSNCA

WT p53
Hub Gene (WT): EGFR
Weight Factor: 1.384
Hub Gene (MUT): CAV1
Weight Factor: 1.344

MUT p53
Hub Gene (MUT): CAV1
Weight Factor: 1.607
Hub Gene (WT): EGFR
Weight Factor: 1.048

MST2 of the coexpression network for WT p53

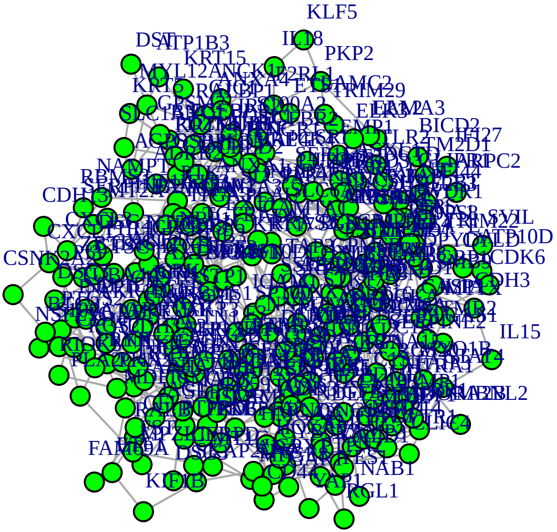

MST2 of the coexpression network for MUT p53

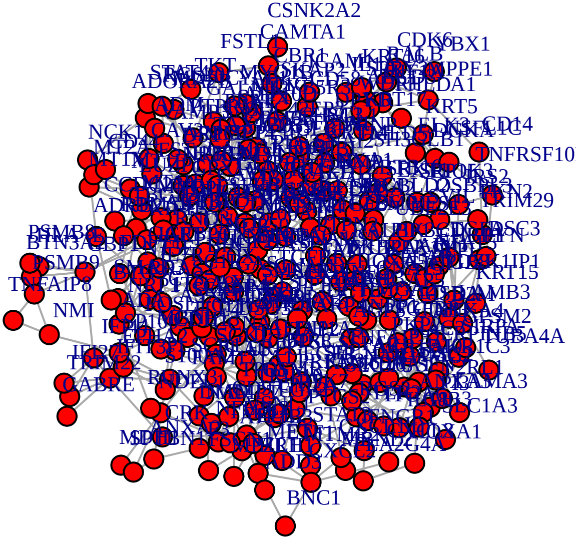

Pathway: CHARAFE_BREAST_CANCER_LUMINAL_VS_MESENCHYMAL_DN

There are 310 genes in this pathway. This pathway was detected by GSNCA

WT p53
Hub Gene (WT): CAV1
Weight Factor: 1.447
Hub Gene (MUT): LOXL2
Weight Factor: 1.426

MUT p53
Hub Gene (MUT): LOXL2
Weight Factor: 1.573
Hub Gene (WT): CAV1
Weight Factor: 1.533

MST2 of the coexpression network for WT p53

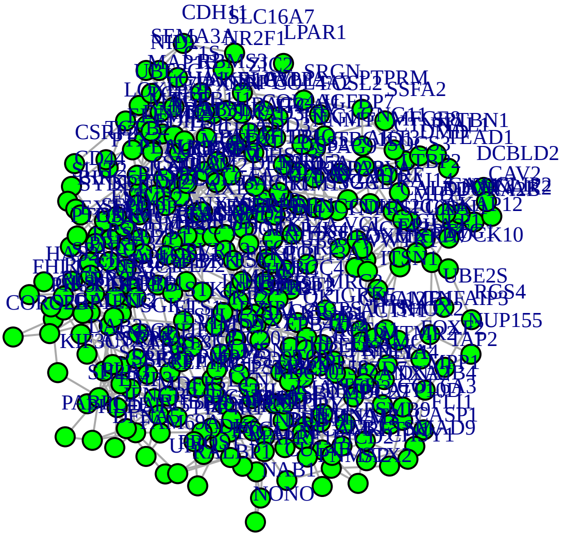

MST2 of the coexpression network for MUT p53

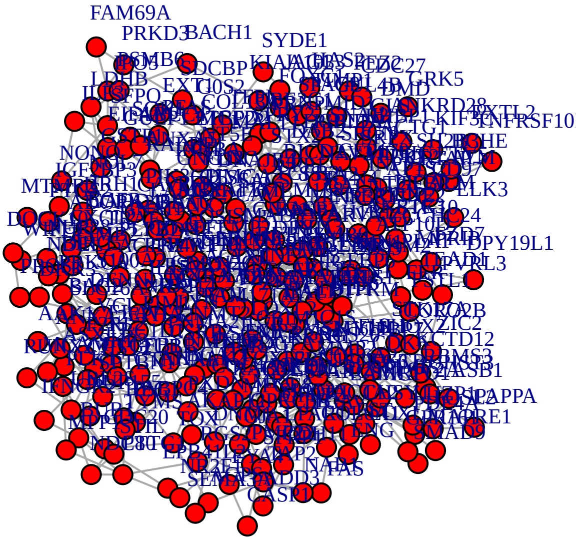

Pathway: DOANE_RESPONSE_TO_ANDROGEN_UP

There are 125 genes in this pathway. This pathway was detected by GSNCA

WT p53
Hub Gene (WT): GM2A
Weight Factor: 1.35
Hub Gene (MUT): SORD
Weight Factor: 0.942

MUT p53
Hub Gene (MUT): SORD
Weight Factor: 1.48
Hub Gene (WT): GM2A
Weight Factor: 0.905

MST2 of the coexpression network for WT p53

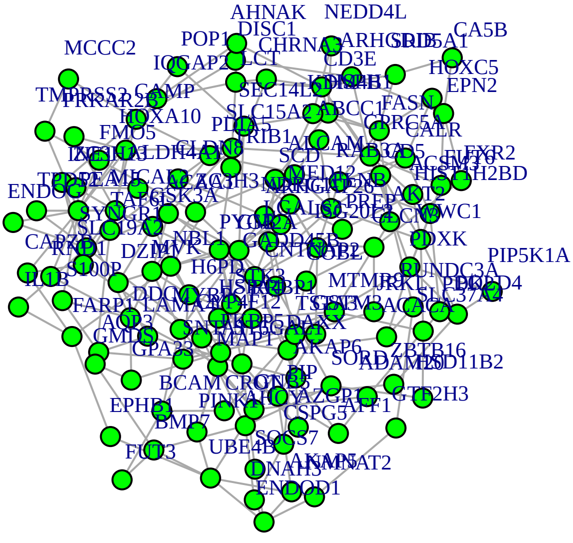

MST2 of the coexpression network for MUT p53

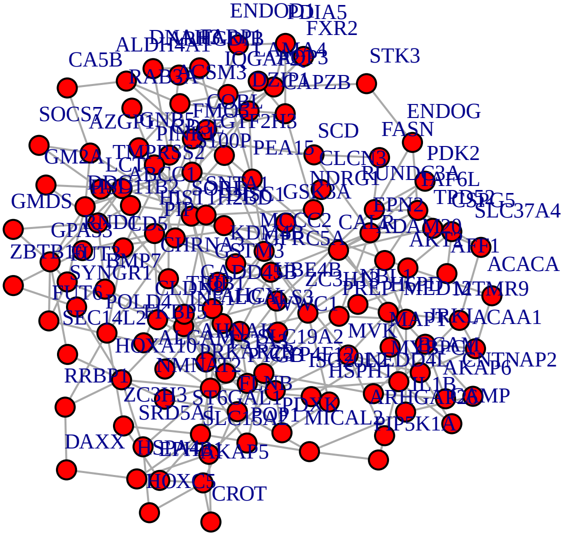

Pathway: HORIUCHI_WTAP_TARGETS_UP

There are 190 genes in this pathway. This pathway was detected by GSNCA

WT p53
Hub Gene (WT): ACP5
Weight Factor: 1.395
Hub Gene (MUT): COL4A1
Weight Factor: 1.07

MUT p53
Hub Gene (MUT): COL4A1
Weight Factor: 1.615
Hub Gene (WT): ACP5
Weight Factor: 0.926

MST2 of the coexpression network for WT p53

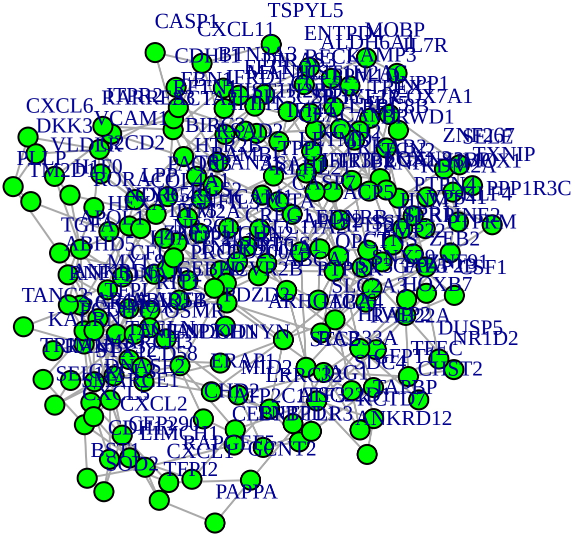

MST2 of the coexpression network for MUT p53

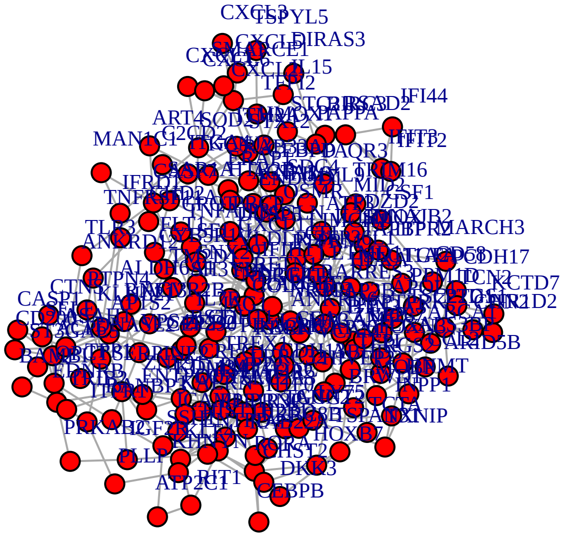

Pathway: SMIRNOV_CIRCULATING_ENDOTHELIOCYTES_IN_CANCER_UP

There are 141 genes in this pathway. This pathway was detected by GSNCA

WT p53
Hub Gene (WT): TGFBI
Weight Factor: 1.247
Hub Gene (MUT): QSOX1
Weight Factor: 1.065

MUT p53
Hub Gene (MUT): QSOX1
Weight Factor: 1.564
Hub Gene (WT): TGFBI
Weight Factor: 1.382

MST2 of the coexpression network for WT p53

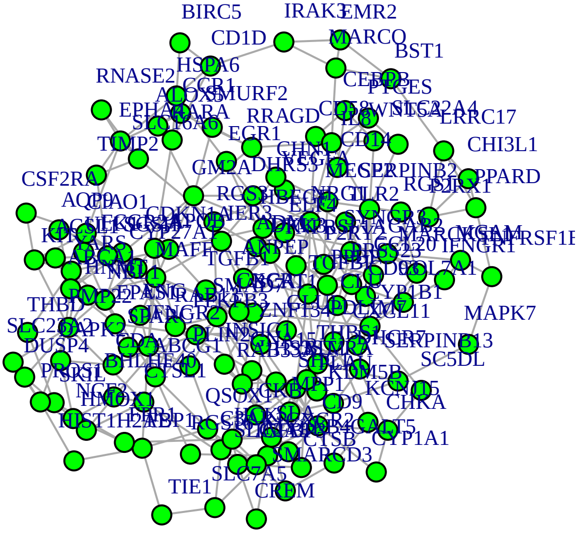

MST2 of the coexpression network for MUT p53

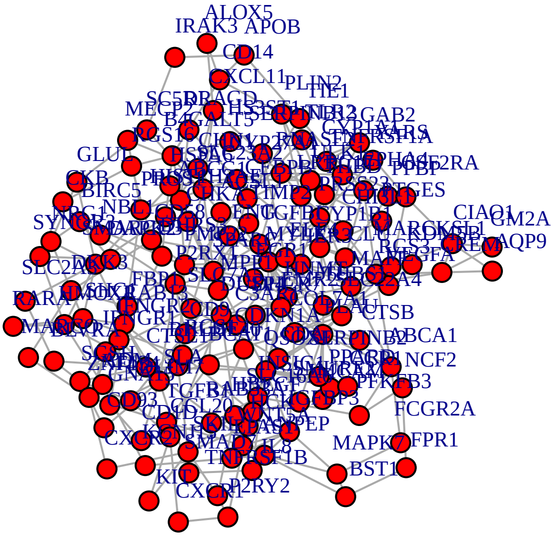

Pathway: HOEBEKE_LYMPHOID_STEM_CELL_UP

There are 76 genes in this pathway. This pathway was detected by GSNCA

WT p53
Hub Gene (WT): RBM39
Weight Factor: 1.375
Hub Gene (MUT): HNRNPA1
Weight Factor: 1.333

MUT p53
Hub Gene (MUT): HNRNPA1
Weight Factor: 1.584
Hub Gene (WT): RBM39
Weight Factor: 1.114

MST2 of the coexpression network for WT p53

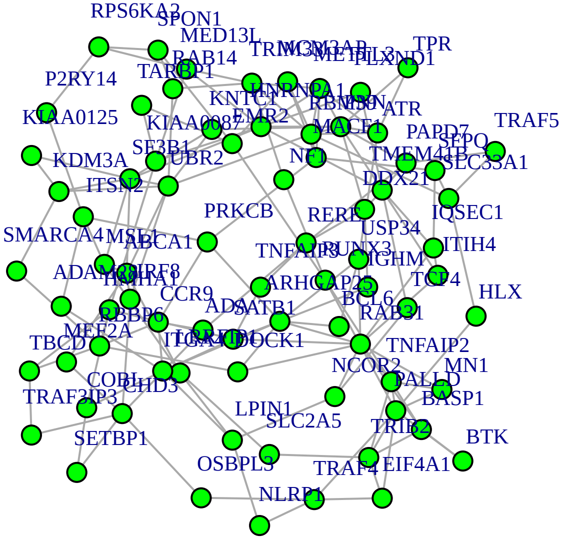

MST2 of the coexpression network for MUT p53

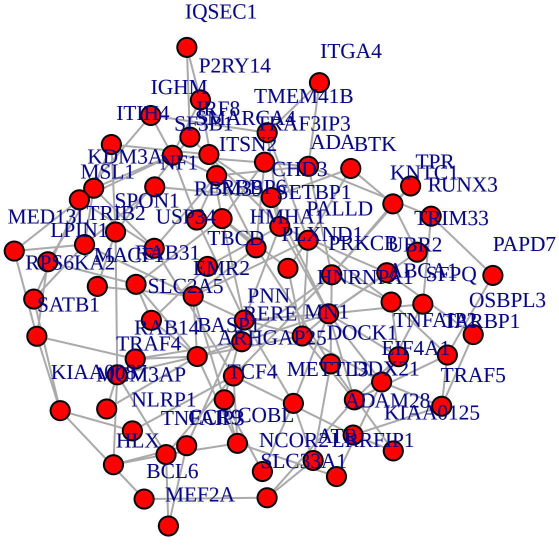

Pathway: SENESE_HDAC1_AND_HDAC2_TARGETS_UP

There are 157 genes in this pathway. This pathway was detected by GSNCA

WT p53
Hub Gene (WT): RRAGD
Weight Factor: 1.366
Hub Gene (MUT): FN1
Weight Factor: 1.004

MUT p53
Hub Gene (MUT): FN1
Weight Factor: 1.574
Hub Gene (WT): RRAGD
Weight Factor: 0.846

MST2 of the coexpression network for WT p53

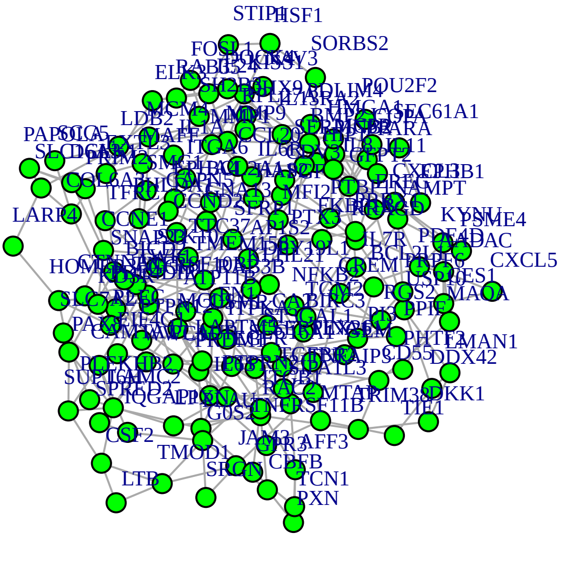

MST2 of the coexpression network for MUT p53

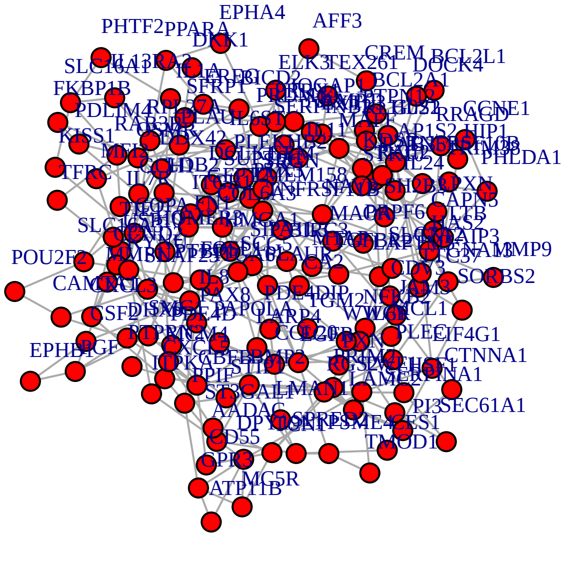

Pathway: SENESE_HDAC1_AND_HDAC2_TARGETS_DN

There are 134 genes in this pathway. This pathway was detected by GSNCA

WT p53
Hub Gene (WT): ALDH3B1
Weight Factor: 1.395
Hub Gene (MUT): MYLK
Weight Factor: 1.315

MUT p53
Hub Gene (MUT): MYLK
Weight Factor: 1.615
Hub Gene (WT): ALDH3B1
Weight Factor: 0.848

MST2 of the coexpression network for WT p53

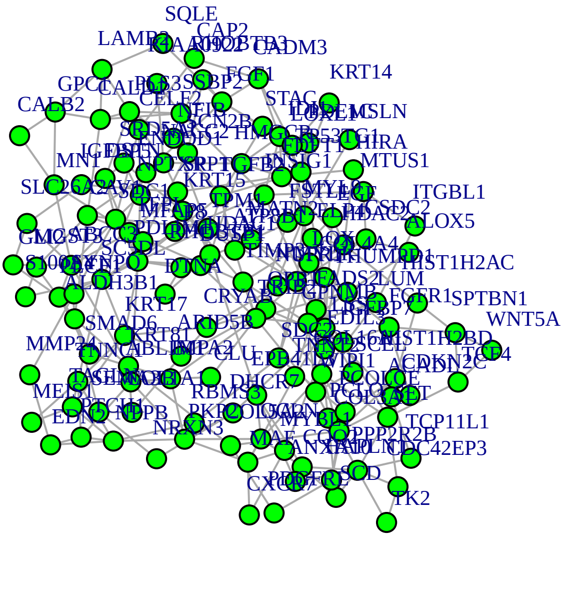

MST2 of the coexpression network for MUT p53

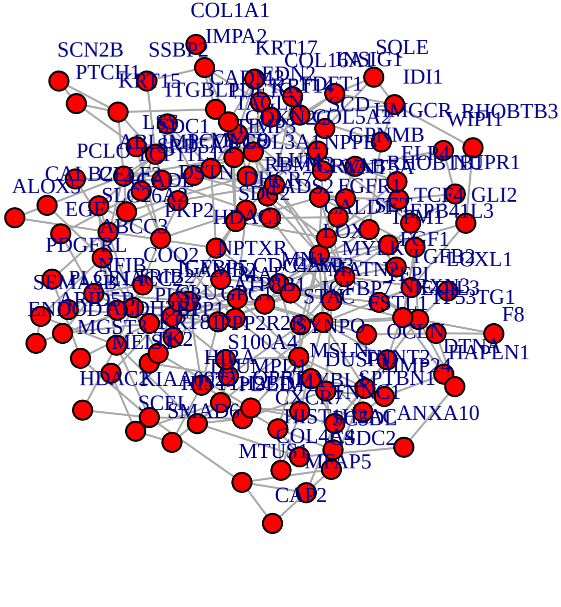

Pathway: SENESE_HDAC3_TARGETS_DN

There are 282 genes in this pathway. This pathway was detected by GSNCA

WT p53
Hub Gene (WT): CYTH3
Weight Factor: 1.416
Hub Gene (MUT): TGFB1I1
Weight Factor: 1

MUT p53
Hub Gene (MUT): TGFB1I1
Weight Factor: 1.583
Hub Gene (WT): CYTH3
Weight Factor: 0.845

MST2 of the coexpression network for WT p53

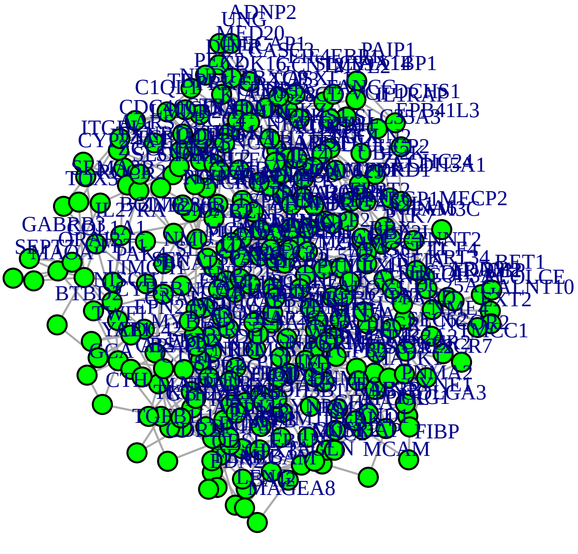

MST2 of the coexpression network for MUT p53

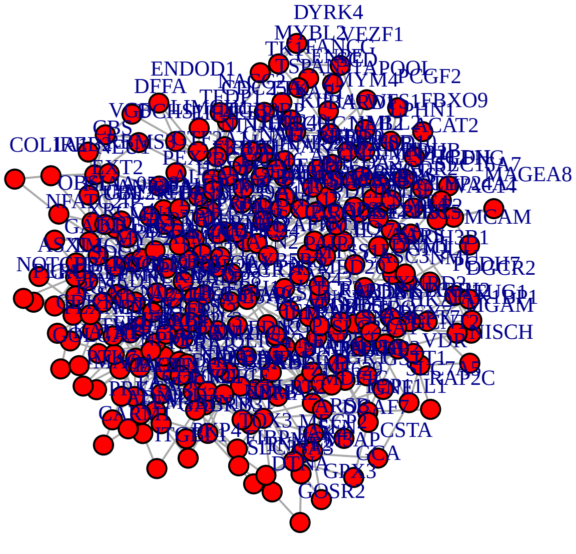

Pathway: WAMUNYOKOLI_OVARIAN_CANCER_LMP_DN

There are 115 genes in this pathway. This pathway was detected by GSNCA

WT p53
Hub Gene (WT): TIMP3
Weight Factor: 1.307
Hub Gene (MUT): CALD1
Weight Factor: 1.158

MUT p53
Hub Gene (MUT): CALD1
Weight Factor: 1.599
Hub Gene (WT): TIMP3
Weight Factor: 1.076

MST2 of the coexpression network for WT p53

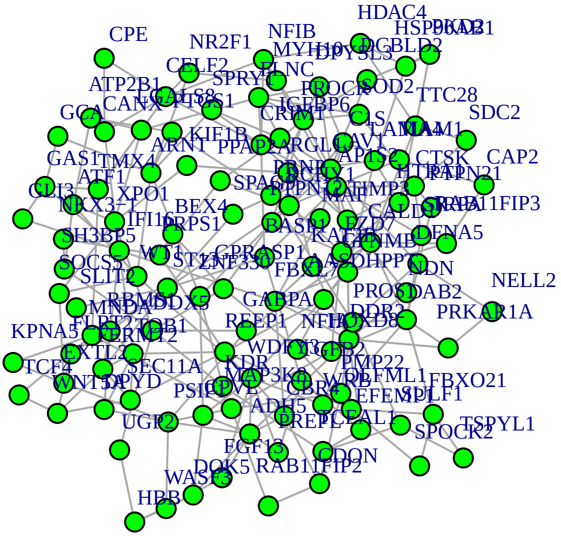

MST2 of the coexpression network for MUT p53

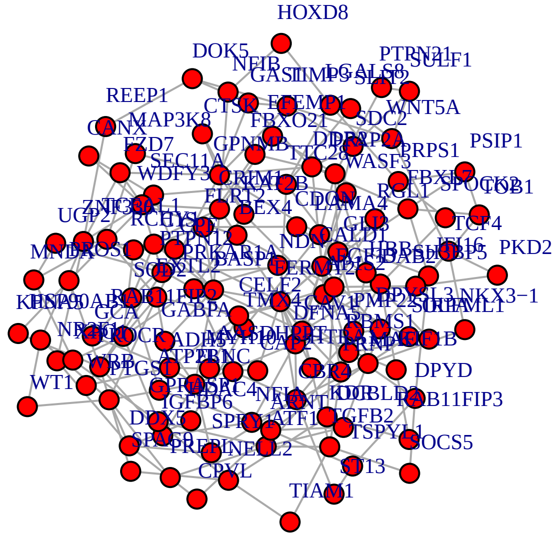

Pathway: DELYS_THYROID_CANCER_DN

There are 184 genes in this pathway. This pathway was detected by GSNCA

WT p53
Hub Gene (WT): ALDH1A2
Weight Factor: 1.222
Hub Gene (MUT): CYR61
Weight Factor: 1.207

MUT p53
Hub Gene (MUT): CYR61
Weight Factor: 1.502
Hub Gene (WT): ALDH1A2
Weight Factor: 1.144

MST2 of the coexpression network for WT p53

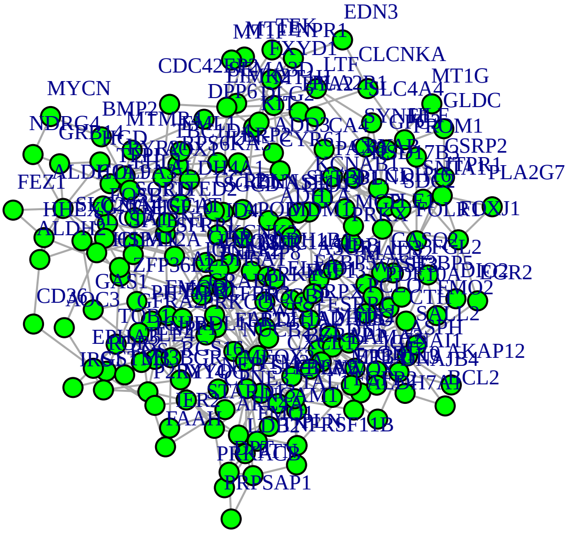

MST2 of the coexpression network for MUT p53

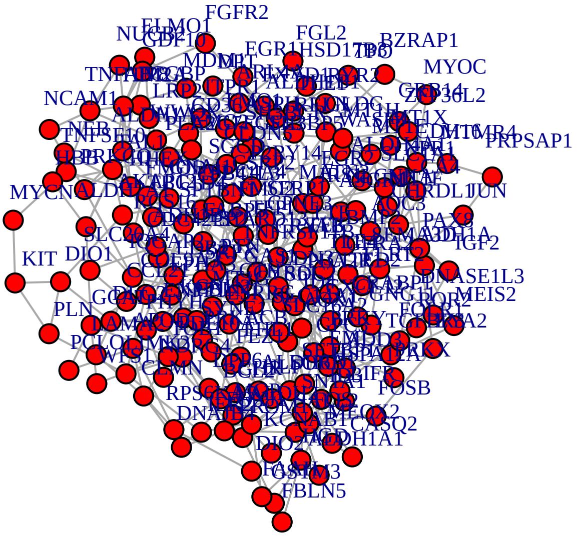

Pathway: BERENJENO_TRANSFORMED_BY_RHOA_REVERSIBLY_DN

There are 22 genes in this pathway. This pathway was detected by GSNCA

WT p53
Hub Gene (WT): SERPINE1
Weight Factor: 1.449
Hub Gene (MUT): CYR61
Weight Factor: 1.384

MUT p53
Hub Gene (MUT): CYR61
Weight Factor: 1.526
Hub Gene (WT): SERPINE1
Weight Factor: 1.201

MST2 of the coexpression network for WT p53

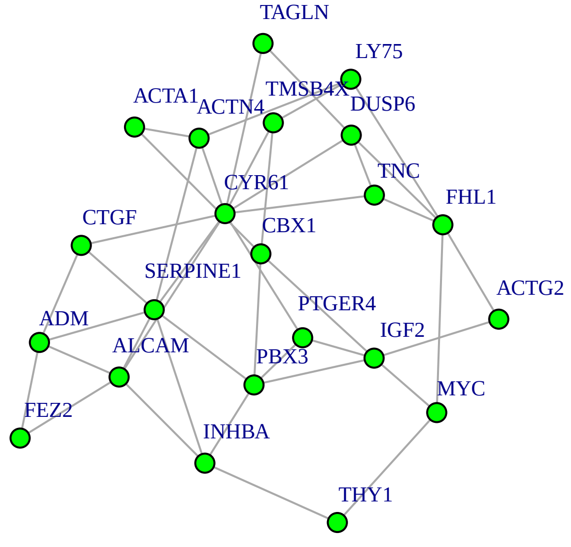

MST2 of the coexpression network for MUT p53

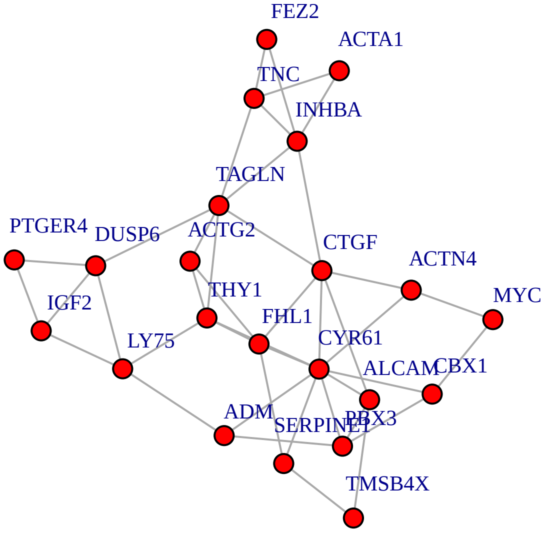

Pathway: BERENJENO_TRANSFORMED_BY_RHOA_DN

There are 290 genes in this pathway. This pathway was detected by GSNCA

WT p53
Hub Gene (WT): CTSA
Weight Factor: 1.397
Hub Gene (MUT): CAV1
Weight Factor: 1.185

MUT p53
Hub Gene (MUT): CAV1
Weight Factor: 1.576
Hub Gene (WT): CTSA
Weight Factor: 1.149

MST2 of the coexpression network for WT p53

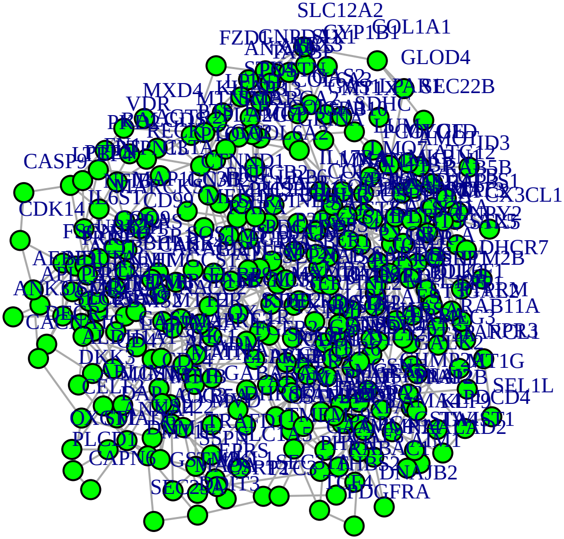

MST2 of the coexpression network for MUT p53

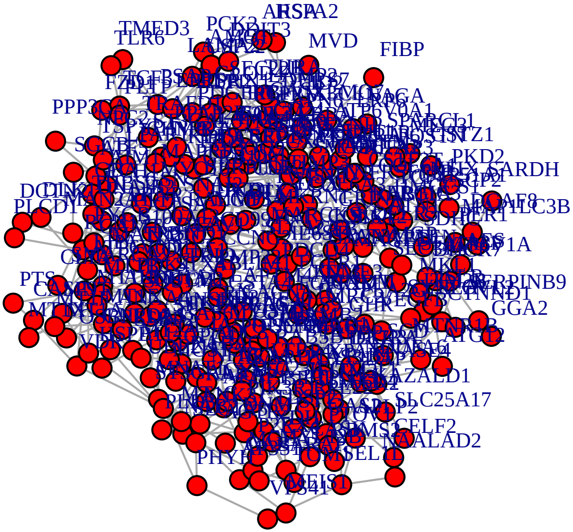

Pathway: WANG_BARRETTS_ESOPHAGUS_AND_ESOPHAGUS_CANCER_DN

There are 30 genes in this pathway. This pathway was detected by GSNCA

WT p53
Hub Gene (WT): CBR3
Weight Factor: 1.505
Hub Gene (MUT): PPL
Weight Factor: 1.139

MUT p53
Hub Gene (MUT): PPL
Weight Factor: 1.419
Hub Gene (WT): CBR3
Weight Factor: 1.145

MST2 of the coexpression network for WT p53

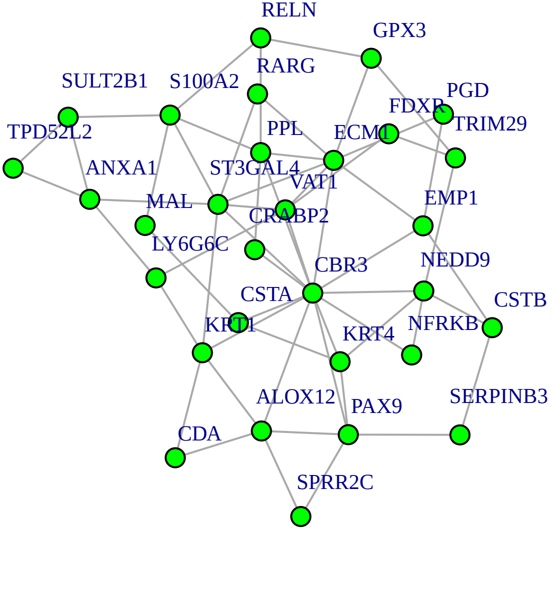

MST2 of the coexpression network for MUT p53

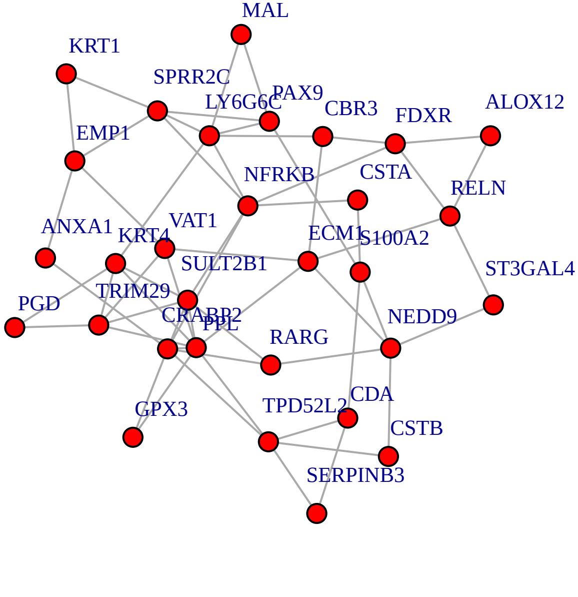

Pathway: TANG_SENESCENCE_TP53_TARGETS_UP

There are 19 genes in this pathway. This pathway was detected by GSNCA

WT p53
Hub Gene (WT): ALDH3A2
Weight Factor: 1.215
Hub Gene (MUT): LCAT
Weight Factor: 0.799

MUT p53
Hub Gene (MUT): LCAT
Weight Factor: 1.506
Hub Gene (WT): ALDH3A2
Weight Factor: 0.686

MST2 of the coexpression network for WT p53

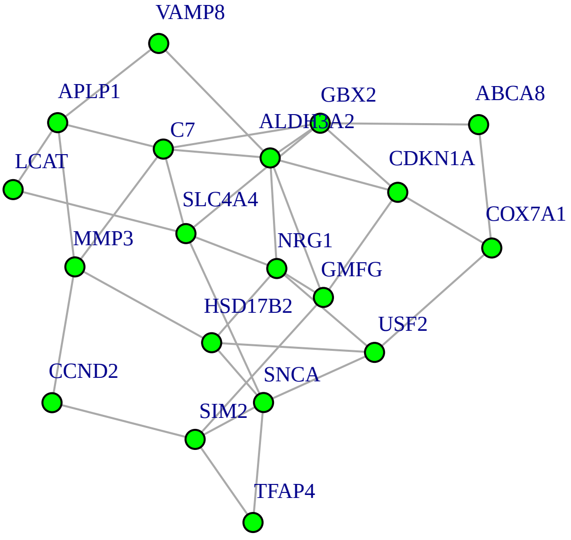

MST2 of the coexpression network for MUT p53

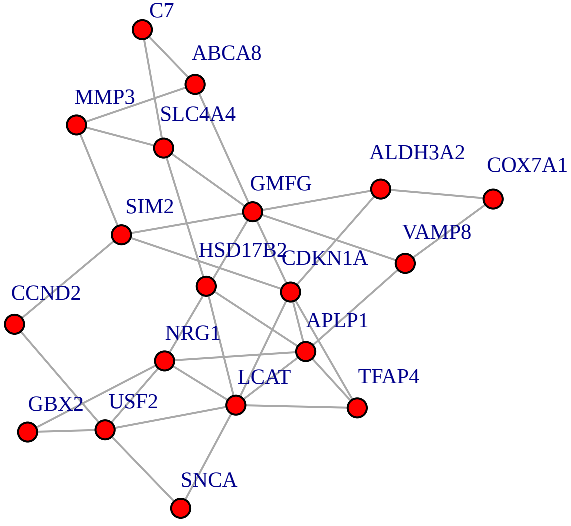

Pathway: WANG_BARRETTS_ESOPHAGUS_UP

There are 43 genes in this pathway. This pathway was detected by GSNCA

WT p53
Hub Gene (WT): ITGA3
Weight Factor: 1.398
Hub Gene (MUT): S100P
Weight Factor: 0.991

MUT p53
Hub Gene (MUT): S100P
Weight Factor: 1.525
Hub Gene (WT): ITGA3
Weight Factor: 1.015

MST2 of the coexpression network for WT p53

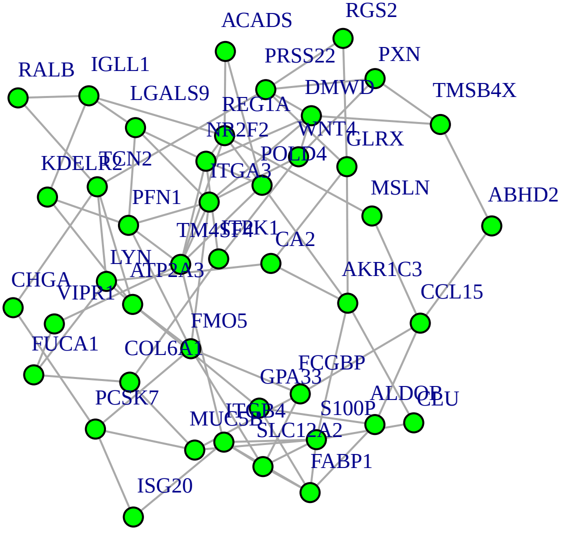

MST2 of the coexpression network for MUT p53

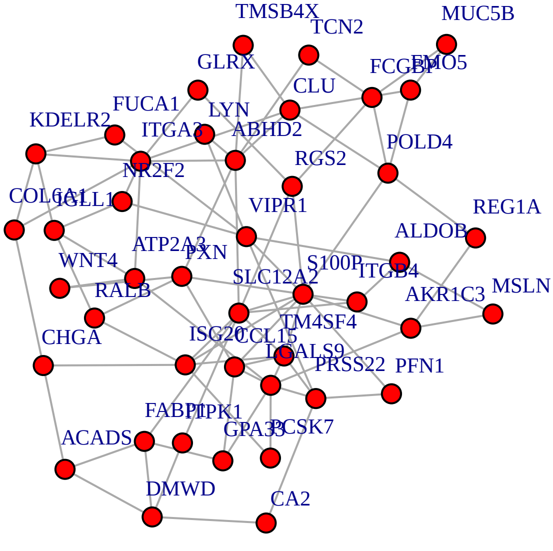

Pathway: MCBRYAN_PUBERTAL_BREAST_3_4WK_UP

There are 142 genes in this pathway. This pathway was detected by GSNCA

WT p53		MUT p53	
**Hub Gene (WT):**	**CLDN3**	**Hub Gene (MUT):**	**EPCAM**
**Weight Factor:**	**1.315**	**Weight Factor:**	**1.692**
**Hub Gene (MUT):**	**EPCAM**	**Hub Gene (WT):**	**CLDN3**
**Weight Factor:**	**1.211**	**Weight Factor:**	**1.563**

MST2 of the coexpression network for WT p53

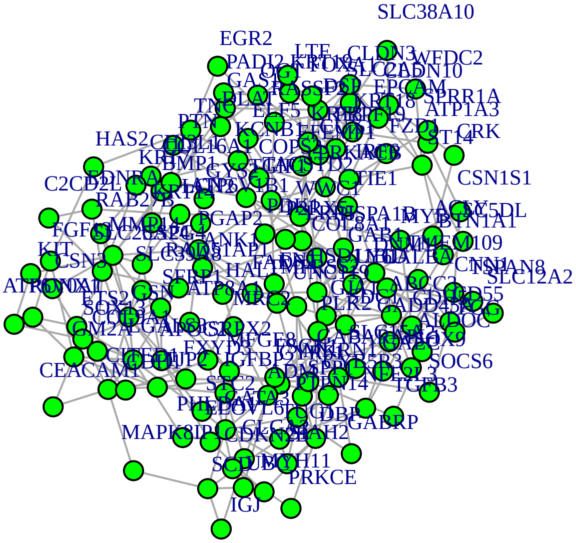

MST2 of the coexpression network for MUT p53

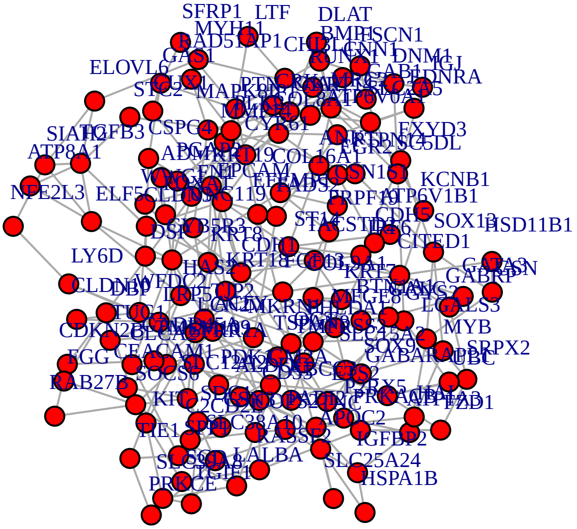

Pathway: MCBRYAN_PUBERTAL_BREAST_4_5WK_UP

There are 171 genes in this pathway. This pathway was detected by GSNCA

WT p53
Hub Gene (WT): CD24
Weight Factor: 1.401
Hub Gene (MUT): EPCAM
Weight Factor: 1.318

MUT p53
Hub Gene (MUT): EPCAM
Weight Factor: 1.673
Hub Gene (WT): CD24
Weight Factor: 1.025

MST2 of the coexpression network for WT p53

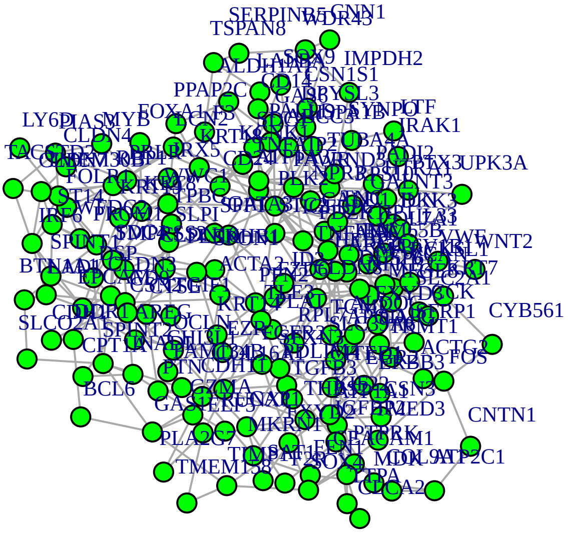

MST2 of the coexpression network for MUT p53

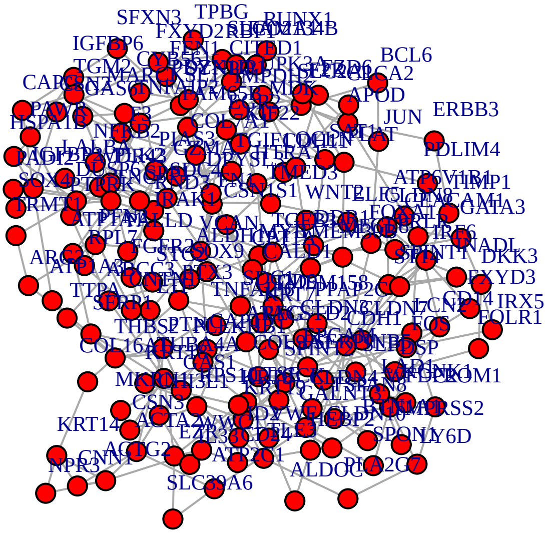

Pathway: OUELLET_CULTURED_OVARIAN_CANCER_INVASIVE_VS_LMP_DN

There are 32 genes in this pathway. This pathway was detected by GSNCA

WT p53
Hub Gene (WT): LIMK2
Weight Factor: 1.293
Hub Gene (MUT): FLNA
Weight Factor: 0.917

MUT p53
Hub Gene (MUT): FLNA
Weight Factor: 1.476
Hub Gene (WT): LIMK2
Weight Factor: 0.672

MST2 of the coexpression network for WT p53

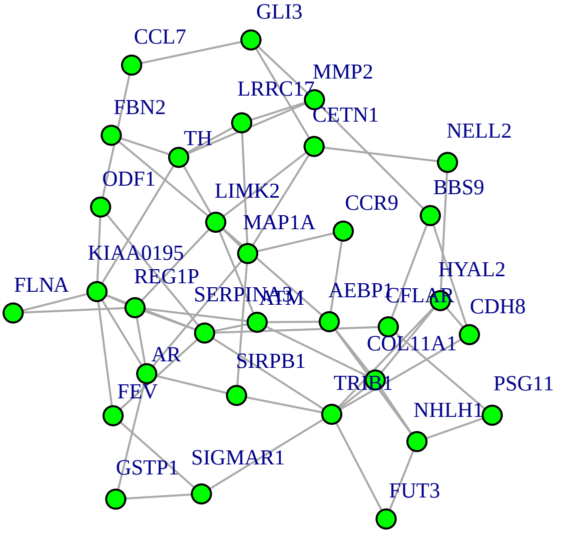

MST2 of the coexpression network for MUT p53

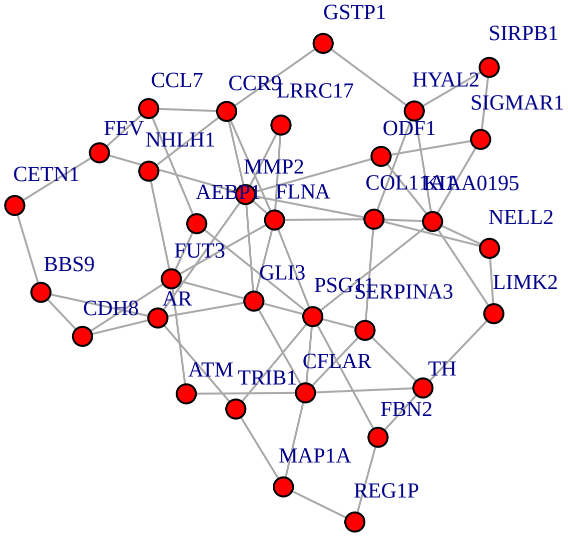

Pathway: KAN_RESPONSE_TO_ARSENIC_TRIOXIDE

There are 103 genes in this pathway. This pathway was detected by GSNCA

WT p53
Hub Gene (WT): SERPINE1
Weight Factor: 1.339
Hub Gene (MUT): GFPT2
Weight Factor: 1.025

MUT p53
Hub Gene (MUT): GFPT2
Weight Factor: 1.557
Hub Gene (WT): SERPINE1
Weight Factor: 1.471

MST2 of the coexpression network for WT p53

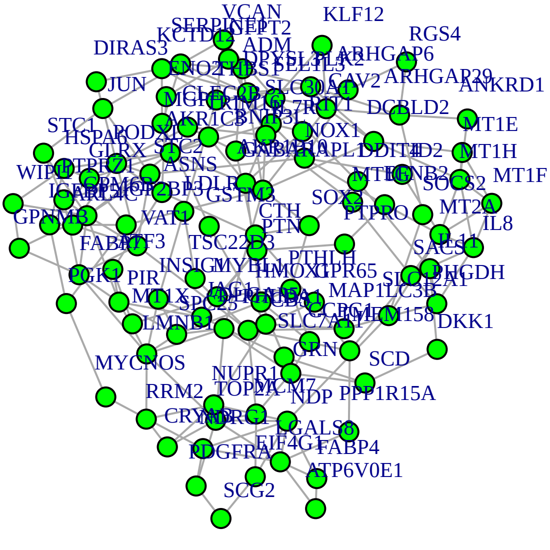

MST2 of the coexpression network for MUT p53

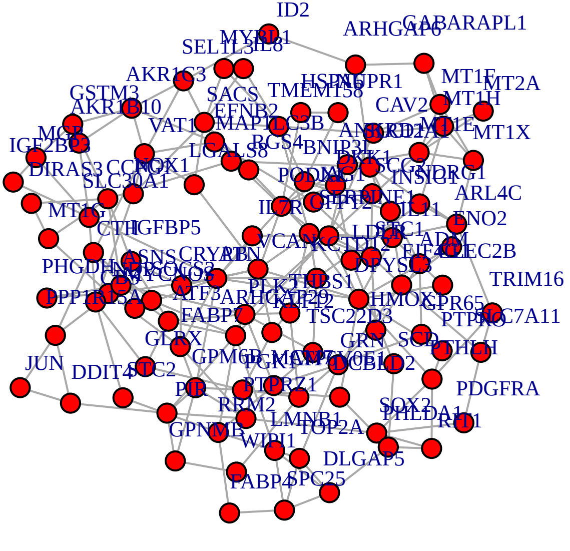

Pathway: HWANG_PROSTATE_CANCER_MARKERS

There are 25 genes in this pathway. This pathway was detected by GSNCA

WT p53
Hub Gene (WT): CTNNB1
Weight Factor: 1.47
Hub Gene (MUT): FLNA
Weight Factor: 0.886

MUT p53
Hub Gene (MUT): FLNA
Weight Factor: 1.524
Hub Gene (WT): CTNNB1
Weight Factor: 0.81

MST2 of the coexpression network for WT p53

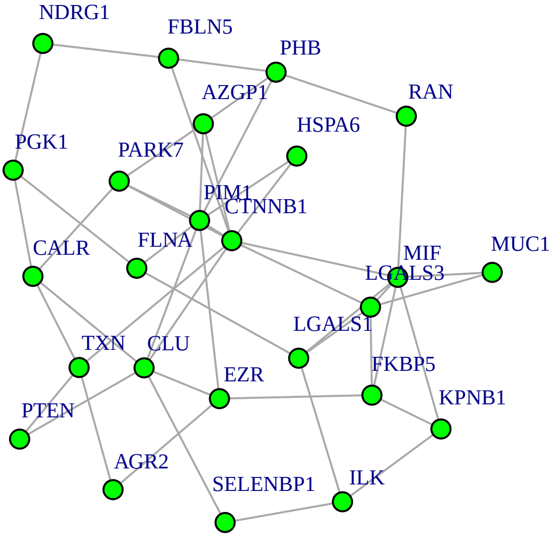

MST2 of the coexpression network for MUT p53

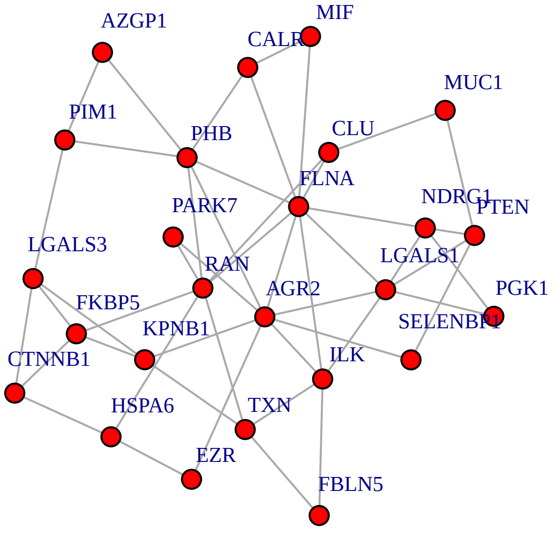

Pathway: HU_ANGIOGENESIS_DN

There are 30 genes in this pathway. This pathway was detected by GSNCA

WT p53
Hub Gene (WT): HMGN4
Weight Factor: 1.399
Hub Gene (MUT): MCM6
Weight Factor: 1.11

MUT p53
Hub Gene (MUT): MCM6
Weight Factor: 1.347
Hub Gene (WT): HMGN4
Weight Factor: 0.869

MST2 of the coexpression network for WT p53

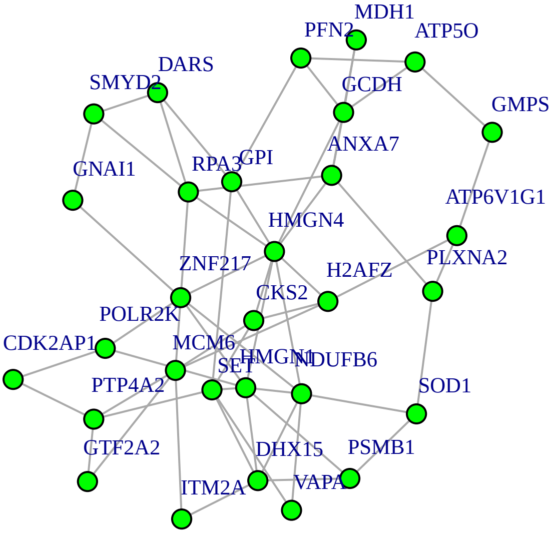

MST2 of the coexpression network for MUT p53

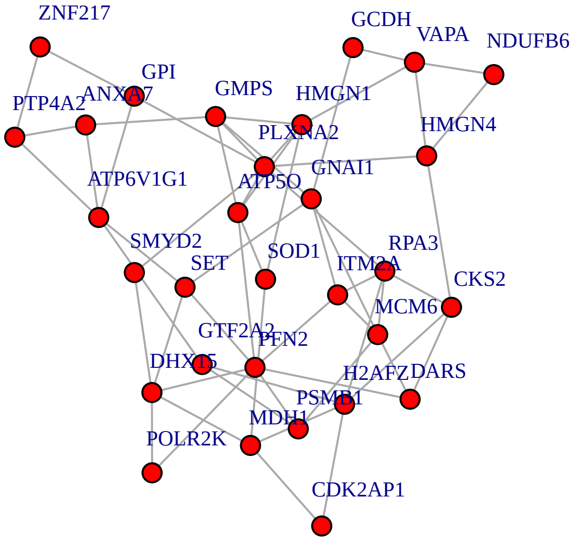

Pathway: RODRIGUES_NTN1_AND_DCC_TARGETS

There are 26 genes in this pathway. This pathway was detected by GSNCA

WT p53
Hub Gene (WT): ETS2
Weight Factor: 1.341
Hub Gene (MUT): MT1F
Weight Factor: 0.953

MUT p53
Hub Gene (MUT): MT1F
Weight Factor: 1.395
Hub Gene (WT): ETS2
Weight Factor: 0.895

MST2 of the coexpression network for WT p53

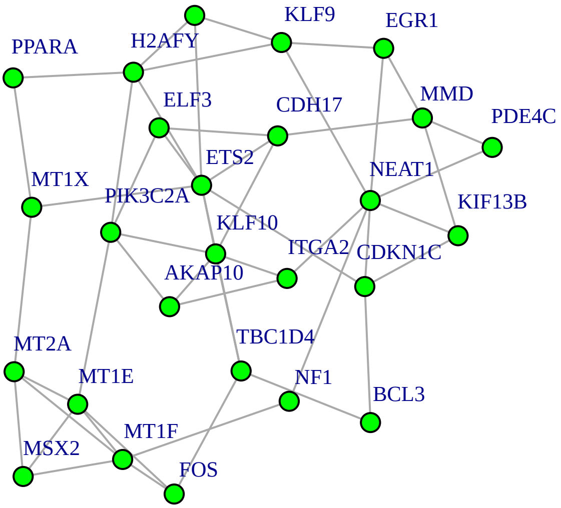

MST2 of the coexpression network for MUT p53

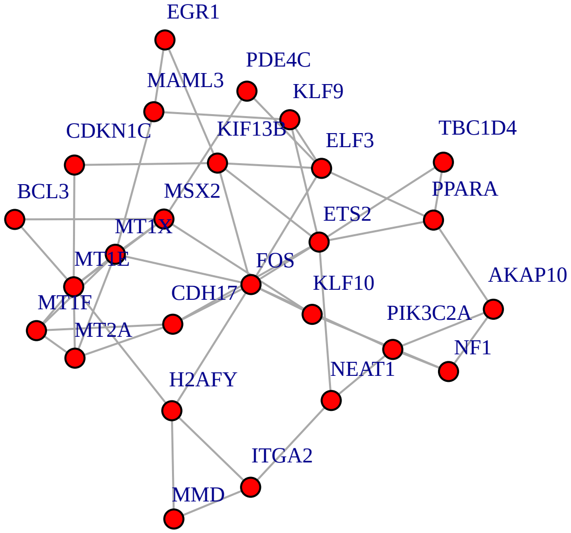

Pathway: DUTTA_APOPTOSIS_VIA_NFKB

There are 29 genes in this pathway. This pathway was detected by GSNCA

WT p53
Hub Gene (WT): BIRC3
Weight Factor: 1.382
Hub Gene (MUT): TNFAIP3
Weight Factor: 0.703

MUT p53
Hub Gene (MUT): TNFAIP3
Weight Factor: 1.272
Hub Gene (WT): BIRC3
Weight Factor: 1.112

MST2 of the coexpression network for WT p53

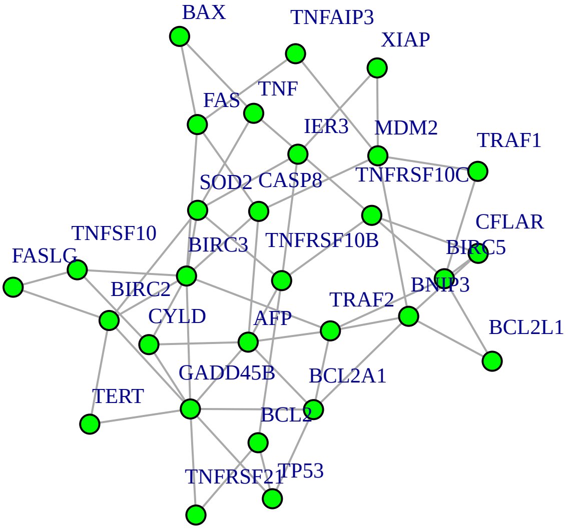

MST2 of the coexpression network for MUT p53

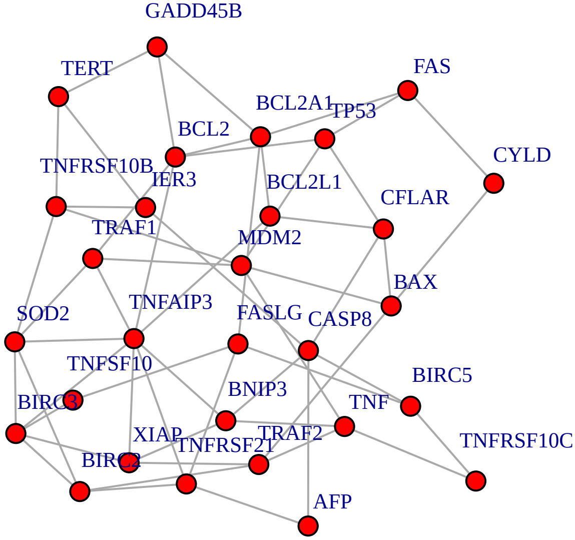

Pathway: RASHI_RESPONSE_TO_IONIZING_RADIATION_2

There are 104 genes in this pathway. This pathway was detected by GSNCA

WT p53
Hub Gene (WT): CAPN3
Weight Factor: 1.524
Hub Gene (MUT): RBMX
Weight Factor: 1.16

MUT p53
Hub Gene (MUT): RBMX
Weight Factor: 1.443
Hub Gene (WT): CAPN3
Weight Factor: 0.979

MST2 of the coexpression network for WT p53

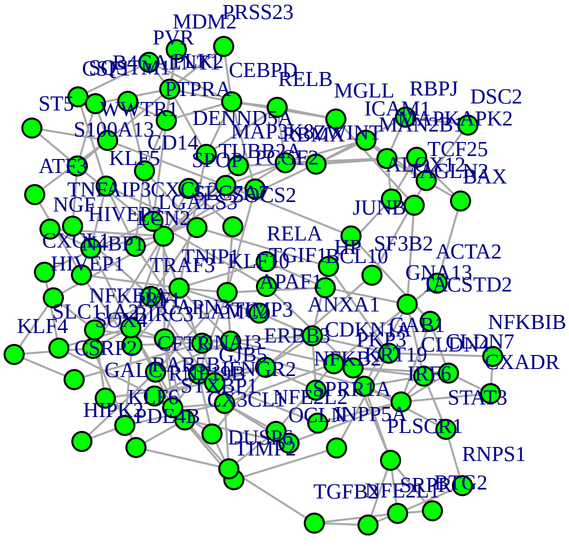

MST2 of the coexpression network for MUT p53

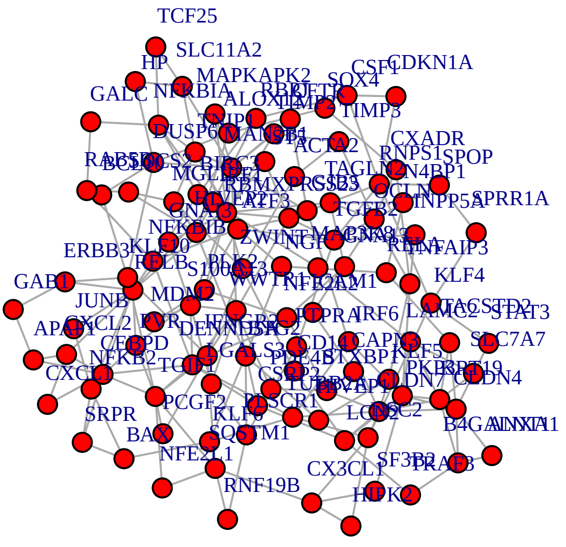

Pathway: LINDGREN_BLADDER_CANCER_HIGH_RECURRENCE

There are 34 genes in this pathway. This pathway was detected by GSNCA

WT p53	MUT p53
**Hub Gene (WT): MICAL2**	**Hub Gene (MUT): SPARC**
**Weight Factor: 1.339**	**Weight Factor: 1.475**
**Hub Gene (MUT): SPARC**	**Hub Gene (WT): MICAL2**
**Weight Factor: 1.18**	**Weight Factor: 0.881**

MST2 of the coexpression network for WT p53

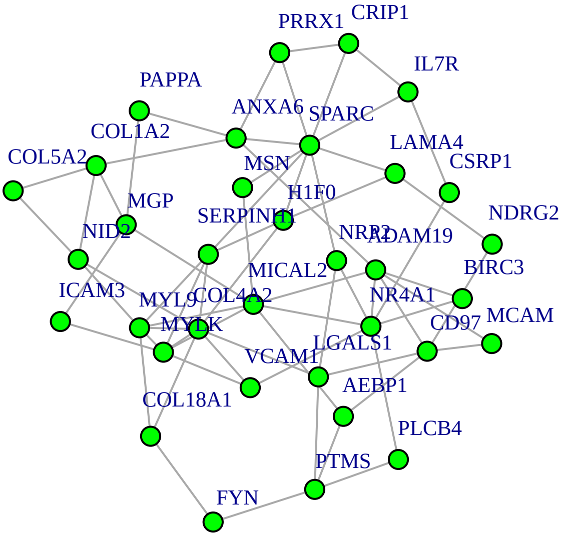

MST2 of the coexpression network for MUT p53

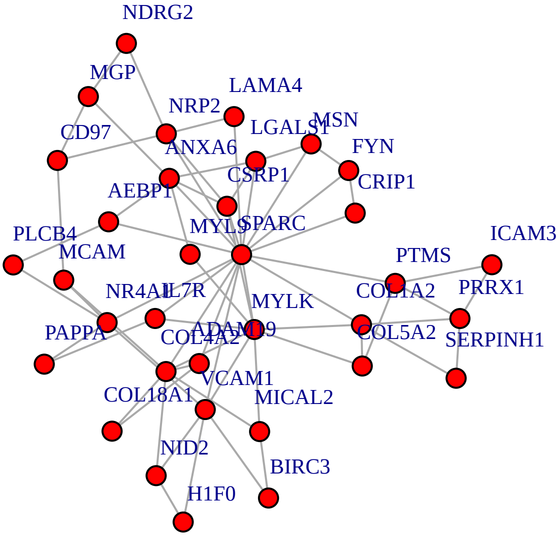

Pathway: SCHEIDEREIT_IKK_INTERACTING_PROTEINS

There are 22 genes in this pathway. This pathway was detected by GSNCA

WT p53
Hub Gene (WT): CTNNB1
Weight Factor: 1.41
Hub Gene (MUT): CASP8
Weight Factor: 0.969

MUT p53
Hub Gene (MUT): CASP8
Weight Factor: 1.284
Hub Gene (WT): CTNNB1
Weight Factor: 0.741

MST2 of the coexpression network for WT p53

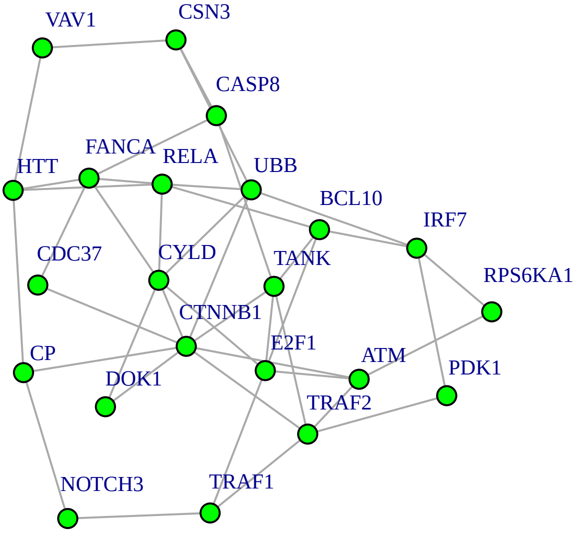

MST2 of the coexpression network for MUT p53

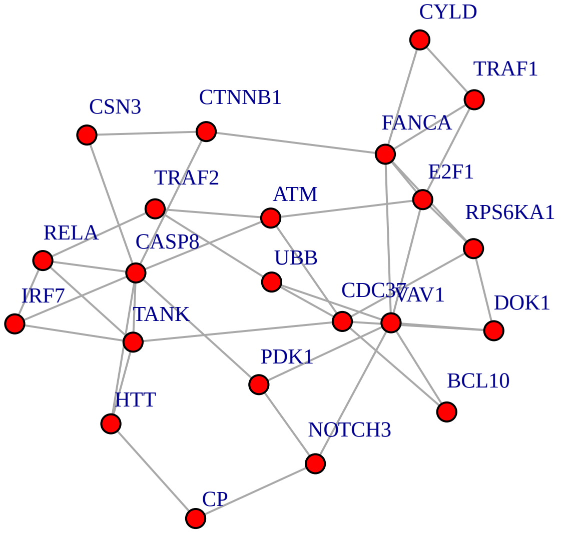

Pathway: KORKOLA_YOLK_SAC_TUMOR

There are 37 genes in this pathway. This pathway was detected by GSNCA

WT p53
Hub Gene (WT): CCKBR
Weight Factor: 1.259
Hub Gene (MUT): C1S
Weight Factor: 1.155

MUT p53
Hub Gene (MUT): C1S
Weight Factor: 1.502
Hub Gene (WT): CCKBR
Weight Factor: 0.794

MST2 of the coexpression network for WT p53

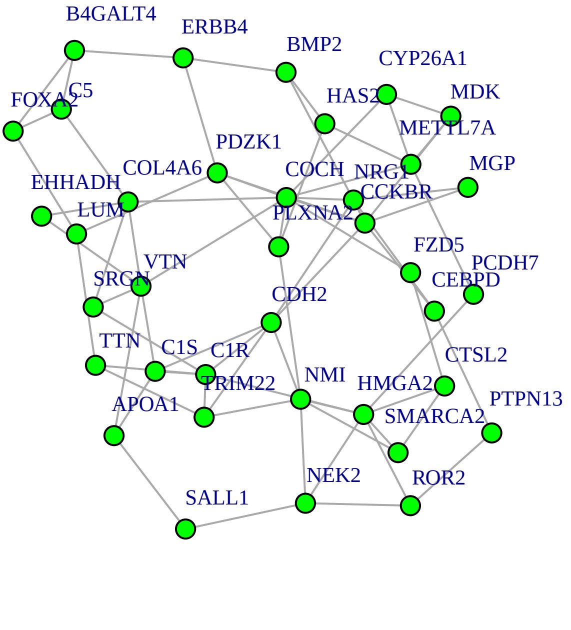

MST2 of the coexpression network for MUT p53

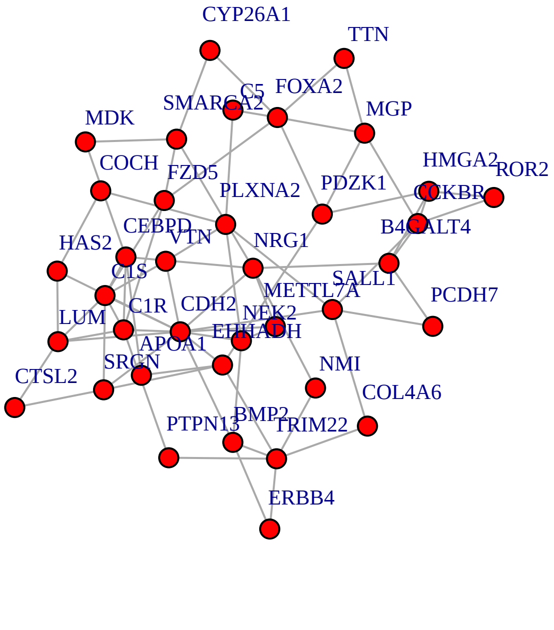

Pathway: LIEN_BREAST_CARCINOMA_METAPLASTIC_VS_DUCTAL_UP

There are 57 genes in this pathway. This pathway was detected by GSNCA

WT p53
Hub Gene (WT): S100B
Weight Factor: 1.321
Hub Gene (MUT): TMEM158
Weight Factor: 0.857

MUT p53
Hub Gene (MUT): TMEM158
Weight Factor: 1.517
Hub Gene (WT): S100B
Weight Factor: 0.815

MST2 of the coexpression network for WT p53

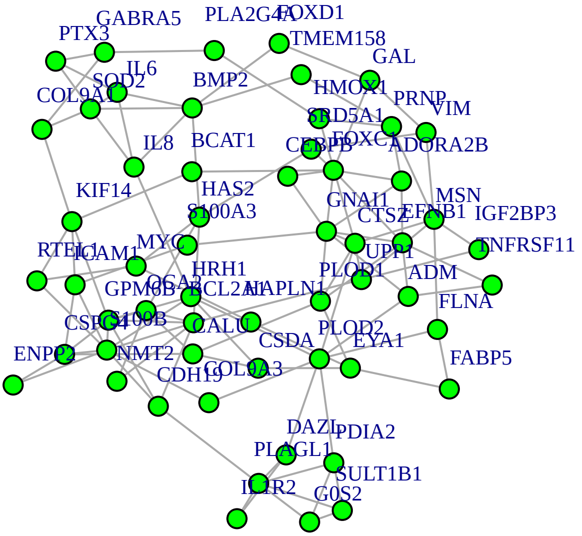

MST2 of the coexpression network for MUT p53

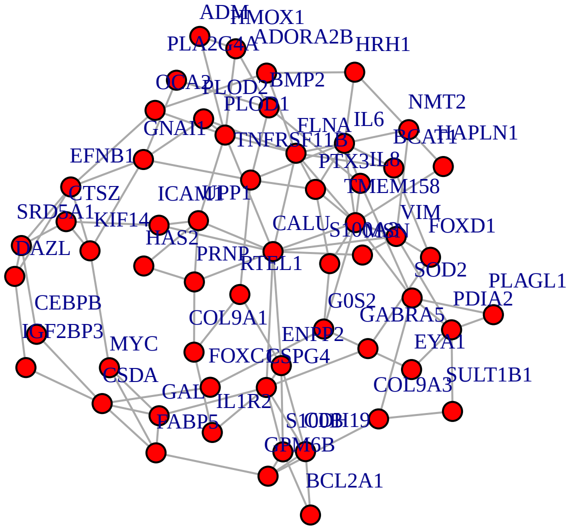

Pathway: MOHANKUMAR_TLX1_TARGETS_DN

There are 106 genes in this pathway. This pathway was detected by GSNCA

WT p53
Hub Gene (WT): ST6GALNAC2
Weight Factor: 1.45
Hub Gene (MUT): S100P
Weight Factor: 0.907

MUT p53
Hub Gene (MUT): S100P
Weight Factor: 1.478
Hub Gene (WT): ST6GALNAC2
Weight Factor: 0.955

MST2 of the coexpression network for WT p53

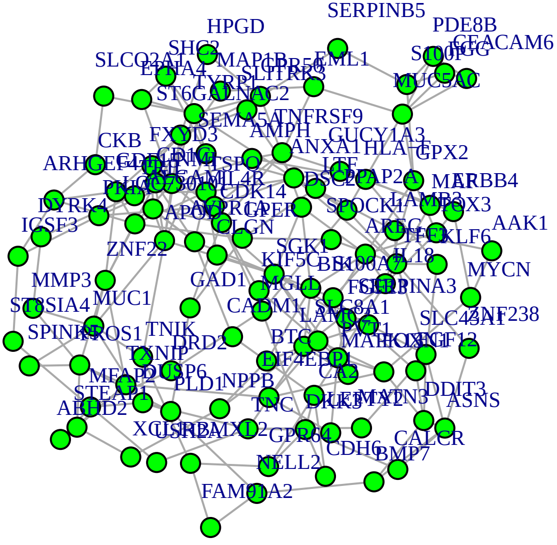

MST2 of the coexpression network for MUT p53

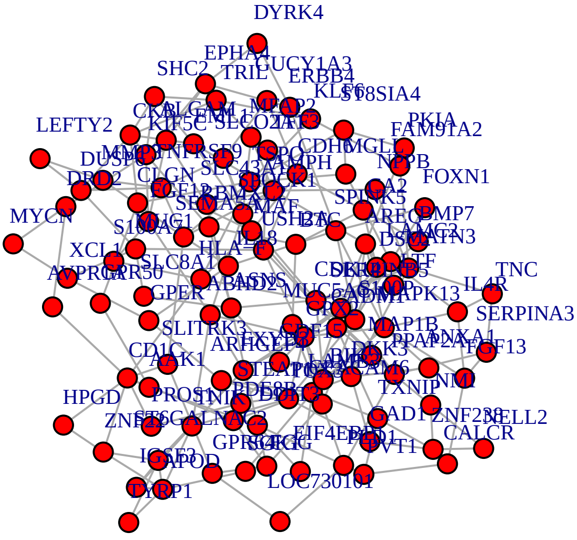

Pathway: WANG_HCP_PROSTATE_CANCER

There are 66 genes in this pathway. This pathway was detected by GSNCA

WT p53
Hub Gene (WT): CTSA
Weight Factor: 1.32
Hub Gene (MUT): FN1
Weight Factor: 1.025

MUT p53
Hub Gene (MUT): FN1
Weight Factor: 1.525
Hub Gene (WT): CTSA
Weight Factor: 1.098

MST2 of the coexpression network for WT p53

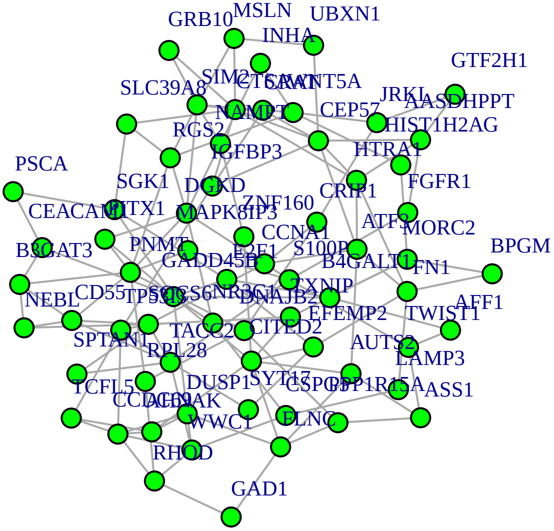

MST2 of the coexpression network for MUT p53

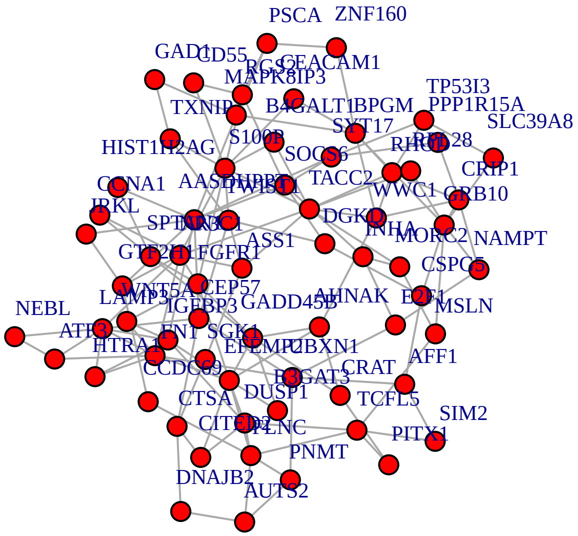

Pathway: FURUKAWA_DUSP6_TARGETS_PCI35_UP

There are 35 genes in this pathway. This pathway was detected by GSNCA

WT p53
Hub Gene (WT): ST6GALNAC2
Weight Factor: 1.415
Hub Gene (MUT): PSMB9
Weight Factor: 1.141

MUT p53
Hub Gene (MUT): PSMB9
Weight Factor: 1.379
Hub Gene (WT): ST6GALNAC2
Weight Factor: 0.853

MST2 of the coexpression network for WT p53

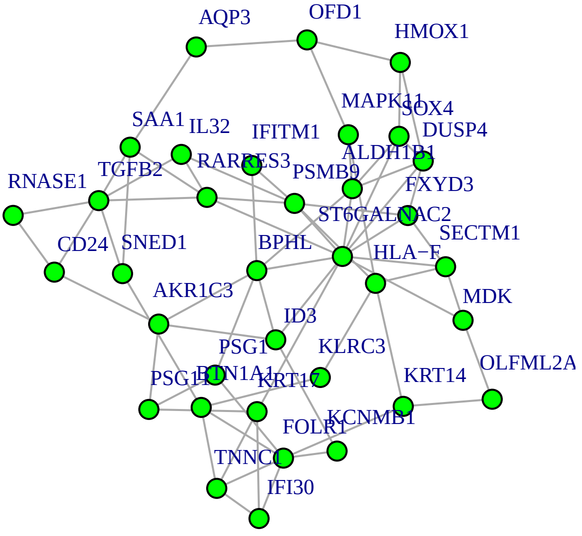

MST2 of the coexpression network for MUT p53

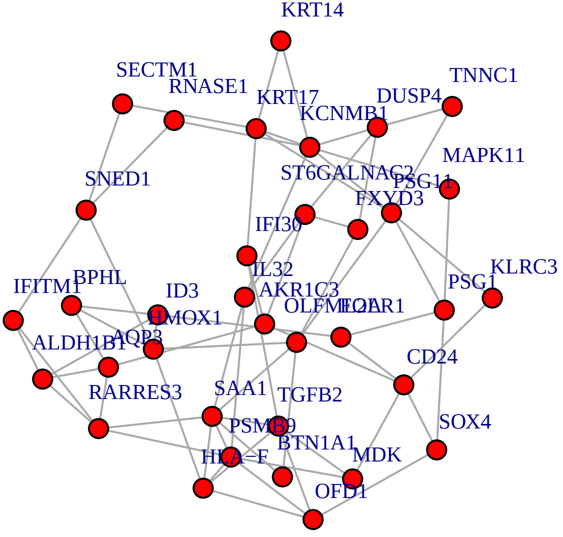

Pathway: SCHLESINGER_METHYLATED_DE_NOVO_IN_CANCER

There are 69 genes in this pathway. This pathway was detected by GSNCA

WT p53
Hub Gene (WT): GATA4
Weight Factor: 1.254
Hub Gene (MUT): MT3
Weight Factor: 1.122

MUT p53
Hub Gene (MUT): MT3
Weight Factor: 1.46
Hub Gene (WT): GATA4
Weight Factor: 0.748

MST2 of the coexpression network for WT p53

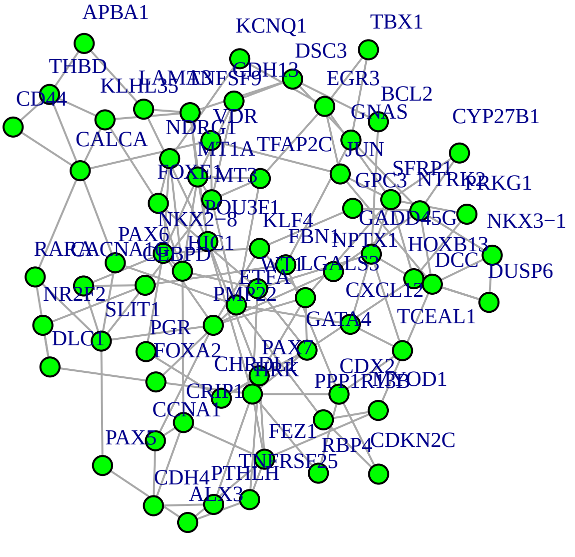

MST2 of the coexpression network for MUT p53

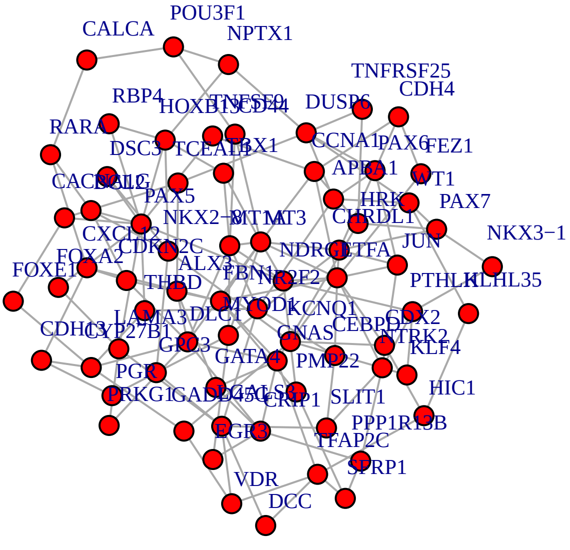

Pathway: WILLIAMS_ESR1_TARGETS_UP

There are 18 genes in this pathway. This pathway was detected by GSNCA

WT p53
Hub Gene (WT): TFF1
Weight Factor: 1.325
Hub Gene (MUT): CCNA2
Weight Factor: 0.978

MUT p53
Hub Gene (MUT): CCNA2
Weight Factor: 1.432
Hub Gene (WT): TFF1
Weight Factor: 0.963

MST2 of the coexpression network for WT p53

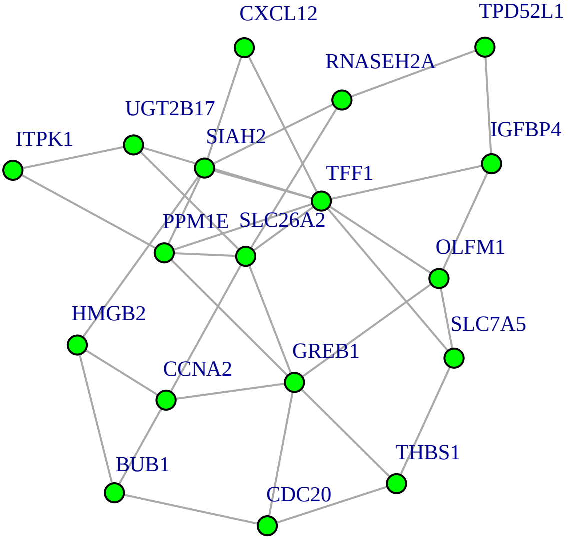

MST2 of the coexpression network for MUT p53

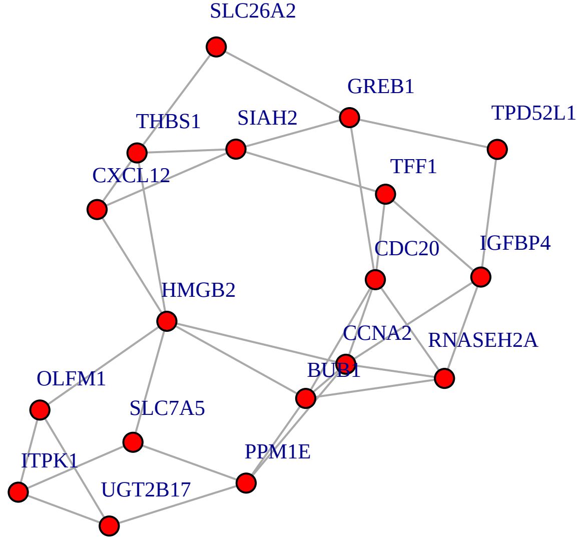

Pathway: NUYTTEN_NIPP1_TARGETS_UP

There are 359 genes in this pathway. This pathway was detected by GSNCA

WT p53
Hub Gene (WT): ST3GAL6
Weight Factor: 1.324
Hub Gene (MUT): CBR4
Weight Factor: 1.144

MUT p53
Hub Gene (MUT): CBR4
Weight Factor: 1.576
Hub Gene (WT): ST3GAL6
Weight Factor: 0.828

MST2 of the coexpression network for WT p53

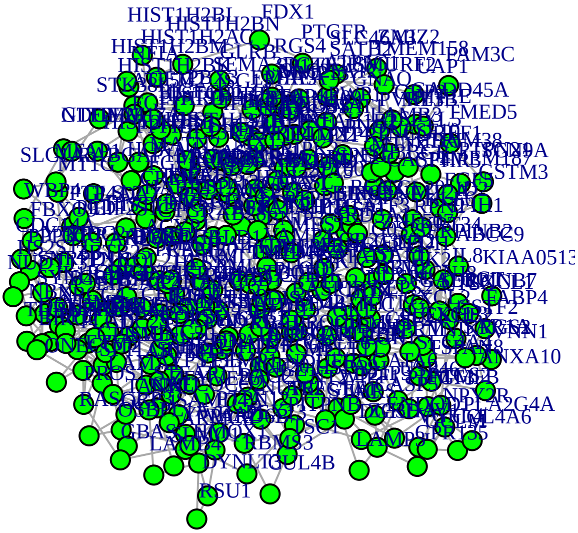

MST2 of the coexpression network for MUT p53

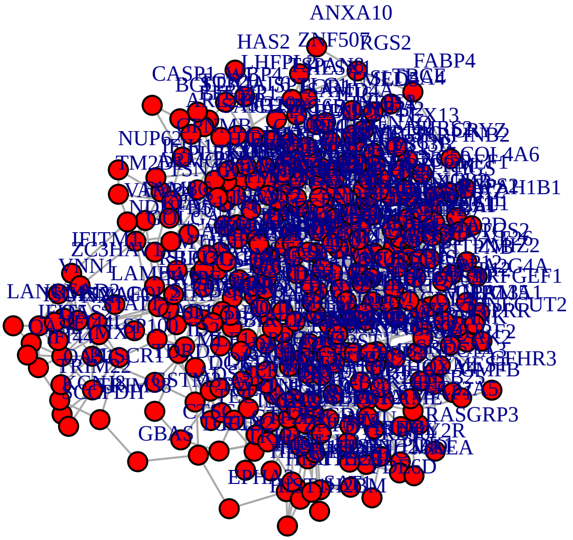

Pathway: WU_CELL_MIGRATION

There are 155 genes in this pathway. This pathway was detected by GSNCA

WT p53
Hub Gene (WT): TNFAIP2
Weight Factor: 1.367
Hub Gene (MUT): EPCAM
Weight Factor: 1.103

MUT p53
Hub Gene (MUT): EPCAM
Weight Factor: 1.57
Hub Gene (WT): TNFAIP2
Weight Factor: 0.704

MST2 of the coexpression network for WT p53 | **MST2 of the coexpression network for MUT p53**

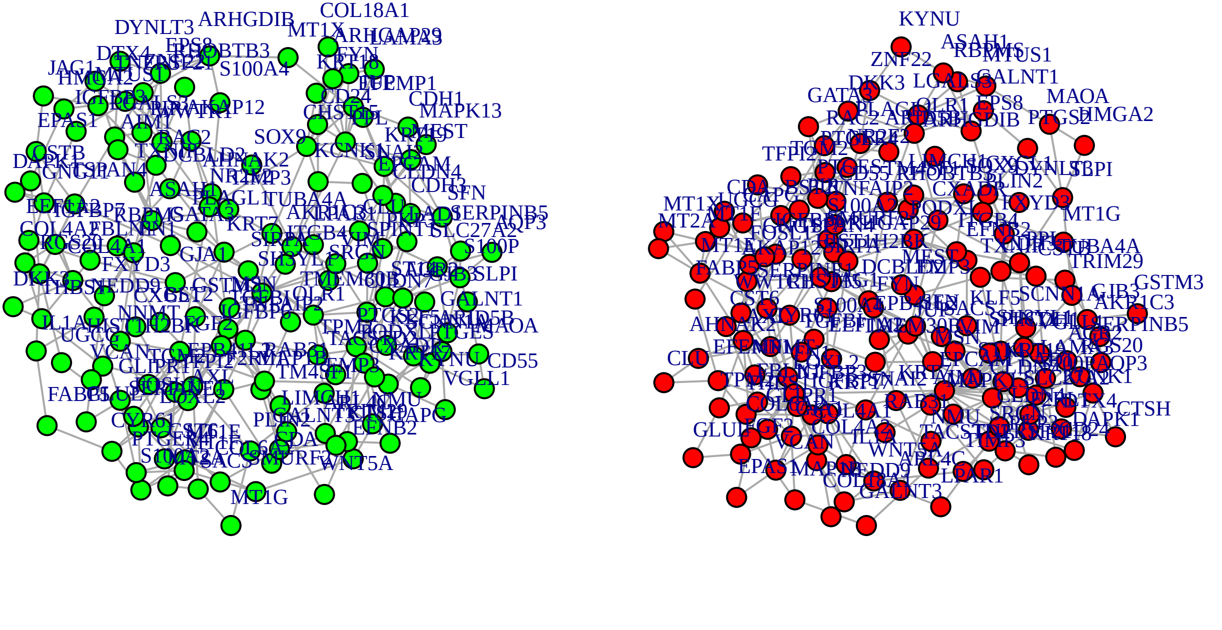

Pathway: PASQUALUCCI_LYMPHOMA_BY_GC_STAGE_UP

There are 163 genes in this pathway. This pathway was detected by GSNCA

WT p53
Hub Gene (WT): ITGB5
Weight Factor: 1.337
Hub Gene (MUT): EPCAM
Weight Factor: 0.979

MUT p53
Hub Gene (MUT): EPCAM
Weight Factor: 1.519
Hub Gene (WT): ITGB5
Weight Factor: 1.265

MST2 of the coexpression network for WT p53

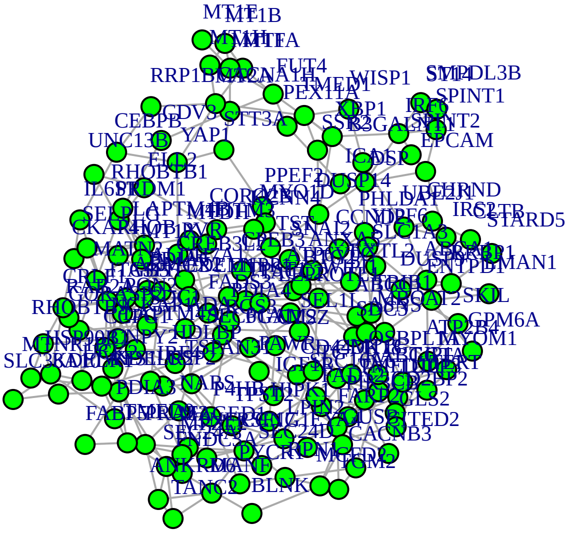

MST2 of the coexpression network for MUT p53

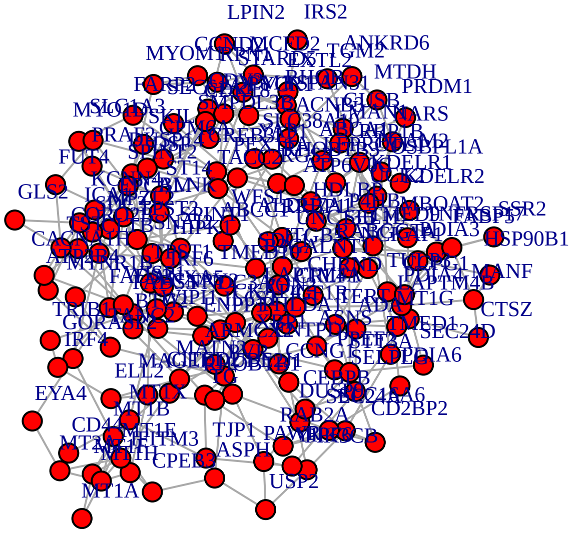

Pathway: JEON_SMAD6_TARGETS_UP

There are 21 genes in this pathway. This pathway was detected by GSNCA

WT p53
Hub Gene (WT): SERPINE1
Weight Factor: 1.354
Hub Gene (MUT): PTX3
Weight Factor: 0.925

MUT p53
Hub Gene (MUT): PTX3
Weight Factor: 1.395
Hub Gene (WT): SERPINE1
Weight Factor: 1.36

MST2 of the coexpression network for WT p53

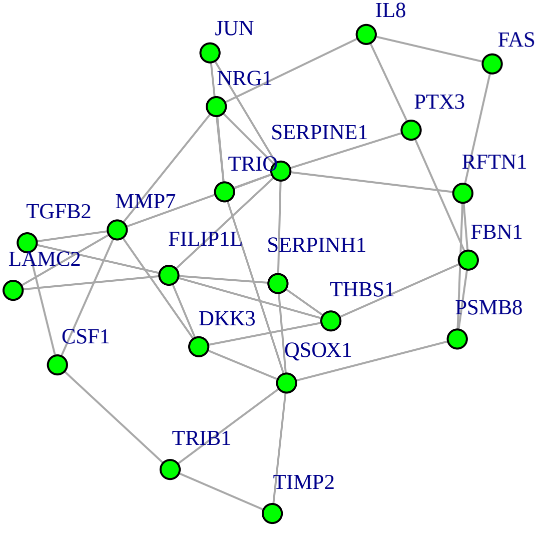

MST2 of the coexpression network for MUT p53

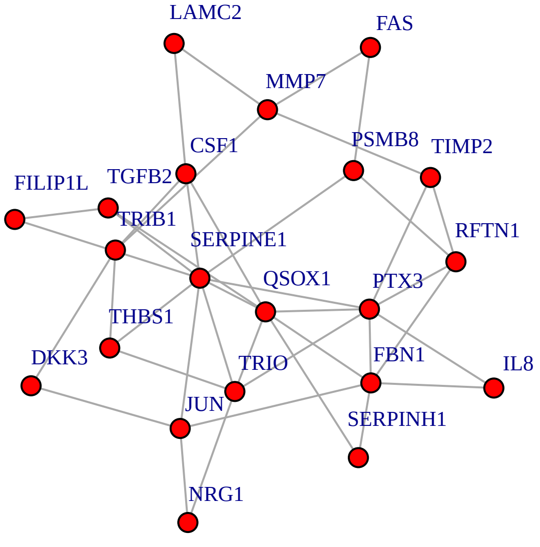

Pathway: NIKOLSKY_BREAST_CANCER_16P13_AMPLICON

There are 43 genes in this pathway. This pathway was detected by GSNCA

WT p53	
**Hub Gene (WT):**	**IFT140**
**Weight Factor:**	**1.288**
**Hub Gene (MUT):**	**MRPL28**
**Weight Factor:**	**1.188**

MUT p53	
**Hub Gene (MUT):**	**MRPL28**
**Weight Factor:**	**1.461**
**Hub Gene (WT):**	**IFT140**
**Weight Factor:**	**0.933**

MST2 of the coexpression network for WT p53

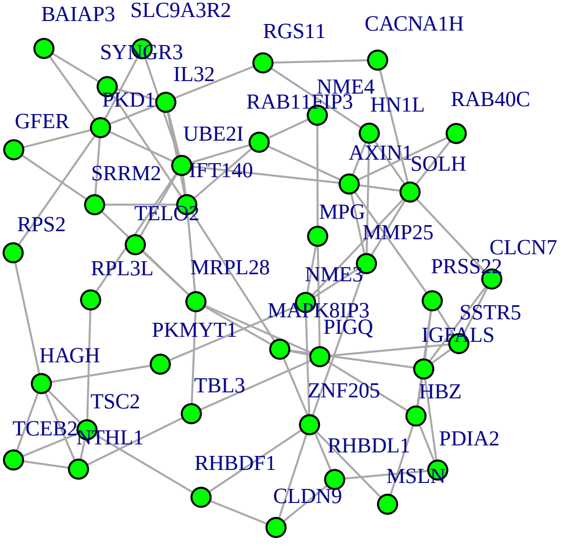

MST2 of the coexpression network for MUT p53

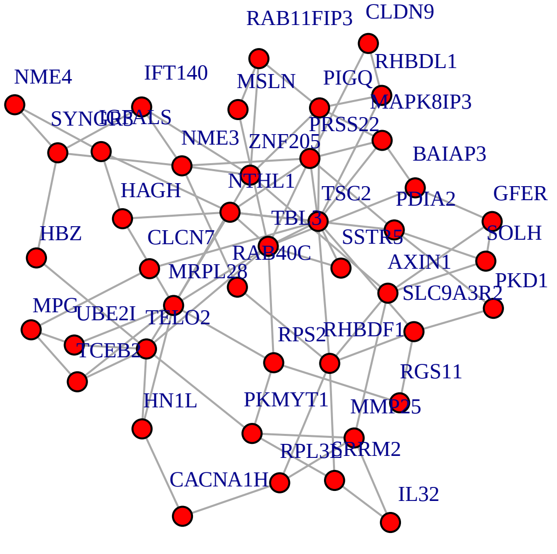

Pathway: ONDER_CDH1_TARGETS_2_DN

There are 343 genes in this pathway. This pathway was detected by GSNCA

WT p53
Hub Gene (WT): PPL
Weight Factor: 1.404
Hub Gene (MUT): EPCAM
Weight Factor: 1.338

MUT p53
Hub Gene (MUT): EPCAM
Weight Factor: 1.728
Hub Gene (WT): PPL
Weight Factor: 1.496

MST2 of the coexpression network for WT p53

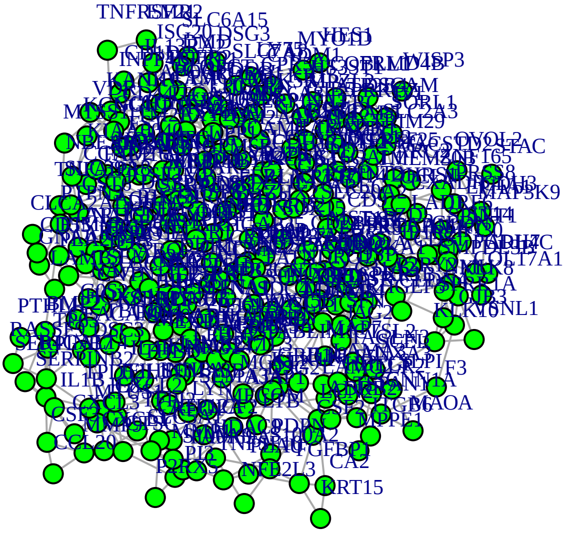

MST2 of the coexpression network for MUT p53

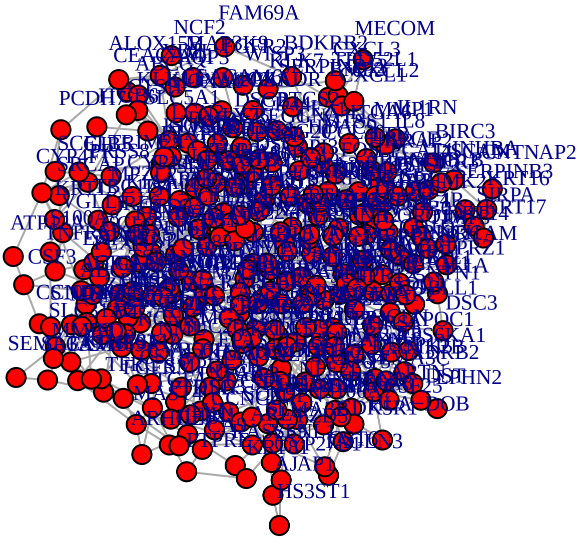

Pathway: ONDER_CDH1_TARGETS_3_DN

There are 42 genes in this pathway. This pathway was detected by GSNCA

WT p53
Hub Gene (WT): IL8
Weight Factor: 1.492
Hub Gene (MUT): LCN2
Weight Factor: 1.198

MUT p53
Hub Gene (MUT): LCN2
Weight Factor: 1.575
Hub Gene (WT): IL8
Weight Factor: 1.02

MST2 of the coexpression network for WT p53

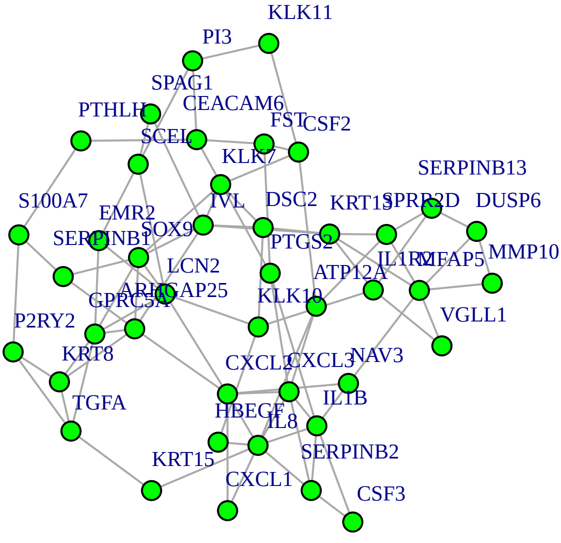

MST2 of the coexpression network for MUT p53

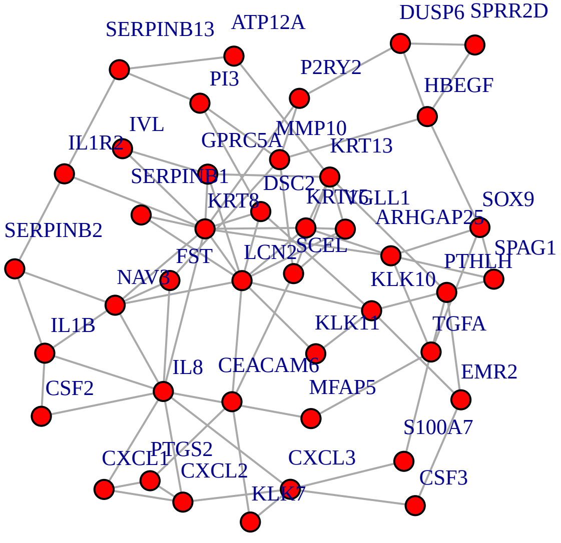

Pathway: ONDER_CDH1_SIGNALING_VIA_CTNNB1

There are 70 genes in this pathway. This pathway was detected by GSNCA

WT p53
Hub Gene (WT): MICAL2
Weight Factor: 1.502
Hub Gene (MUT): FBN1
Weight Factor: 1.149

MUT p53
Hub Gene (MUT): FBN1
Weight Factor: 1.541
Hub Gene (WT): MICAL2
Weight Factor: 1.039

MST2 of the coexpression network for WT p53

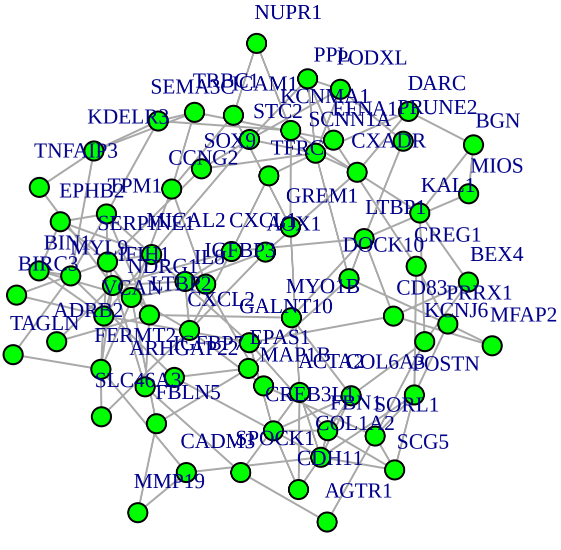

MST2 of the coexpression network for MUT p53

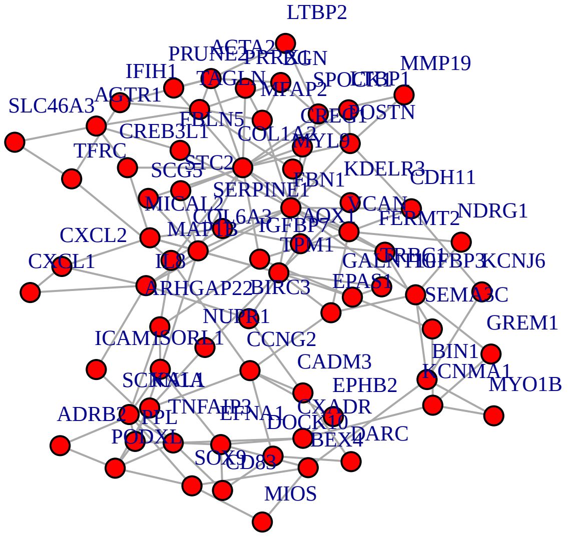

Pathway: CERVERA_SDHB_TARGETS_1_UP

There are 60 genes in this pathway. This pathway was detected by GSNCA

WT p53
Hub Gene (WT): ST6GALNAC2
Weight Factor: 1.292
Hub Gene (MUT): LOXL2
Weight Factor: 1.217

MUT p53
Hub Gene (MUT): LOXL2
Weight Factor: 1.5
Hub Gene (WT): ST6GALNAC2
Weight Factor: 0.914

MST2 of the coexpression network for WT p53

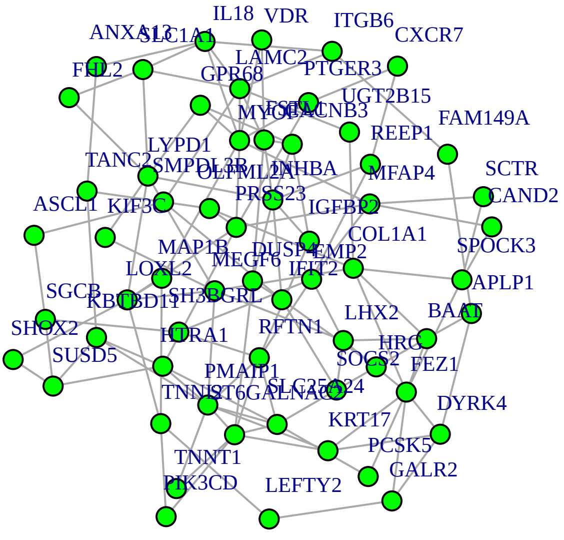

MST2 of the coexpression network for MUT p53

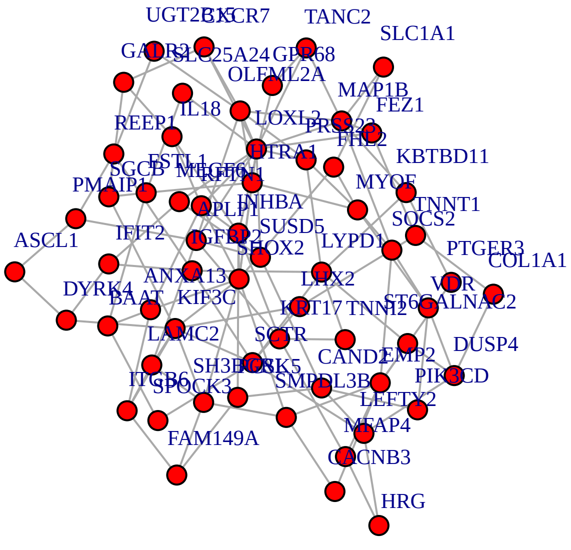

Pathway: UEDA_CENTRAL_CLOCK

There are 67 genes in this pathway. This pathway was detected by GSNCA

WT p53
Hub Gene (WT): GADD45B
Weight Factor: 1.427
Hub Gene (MUT): HNRNPM
Weight Factor: 1.16

MUT p53
Hub Gene (MUT): HNRNPM
Weight Factor: 1.308
Hub Gene (WT): GADD45B
Weight Factor: 0.892

MST2 of the coexpression network for WT p53

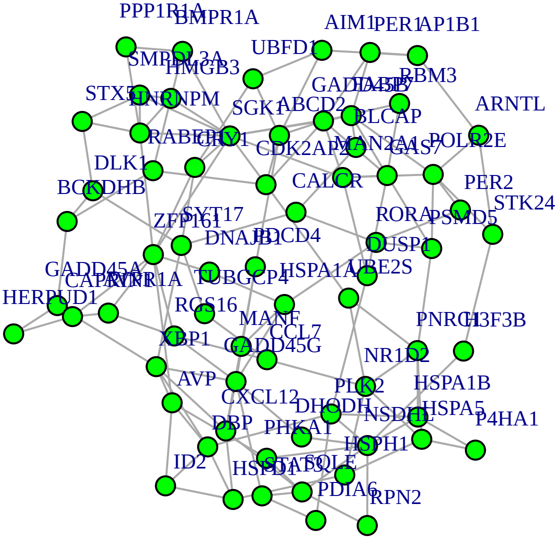

MST2 of the coexpression network for MUT p53

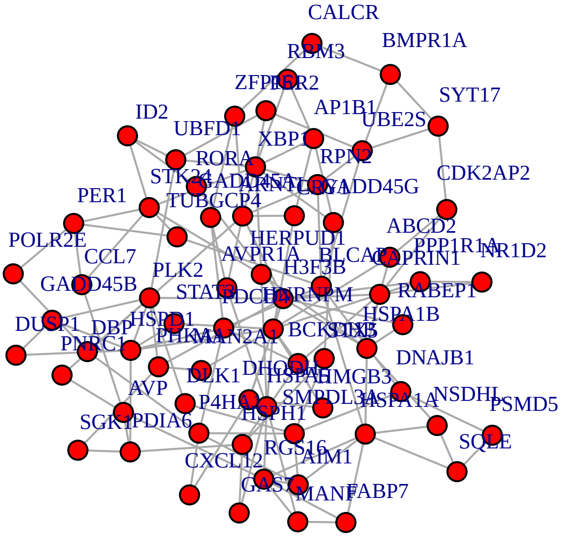

Pathway: WILLERT_WNT_SIGNALING

There are 19 genes in this pathway. This pathway was detected by GSNCA

WT p53
Hub Gene (WT): SRP19
Weight Factor: 1.377
Hub Gene (MUT): SNRPA1
Weight Factor: 1.311

MUT p53
Hub Gene (MUT): SNRPA1
Weight Factor: 1.366
Hub Gene (WT): SRP19
Weight Factor: 0.708

MST2 of the coexpression network for WT p53

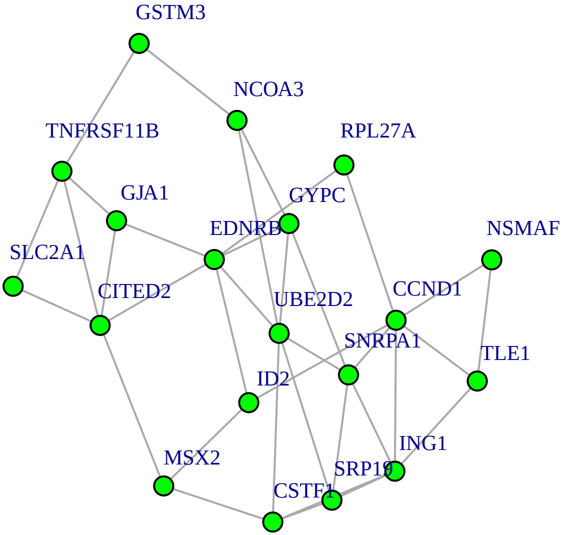

MST2 of the coexpression network for MUT p53

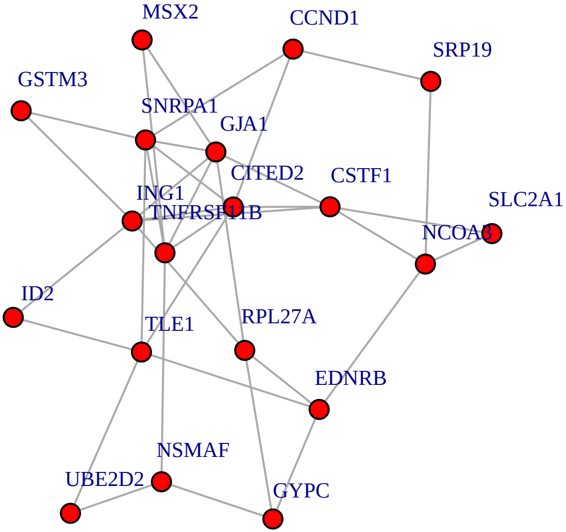

Pathway: DORSAM_HOXA9_TARGETS_DN

There are 27 genes in this pathway. This pathway was detected by GSNCA

WT p53
Hub Gene (WT): DCT
Weight Factor: 1.344
Hub Gene (MUT): MAN2A2
Weight Factor: 0.733

MUT p53
Hub Gene (MUT): MAN2A2
Weight Factor: 1.302
Hub Gene (WT): DCT
Weight Factor: 1.182

MST2 of the coexpression network for WT p53

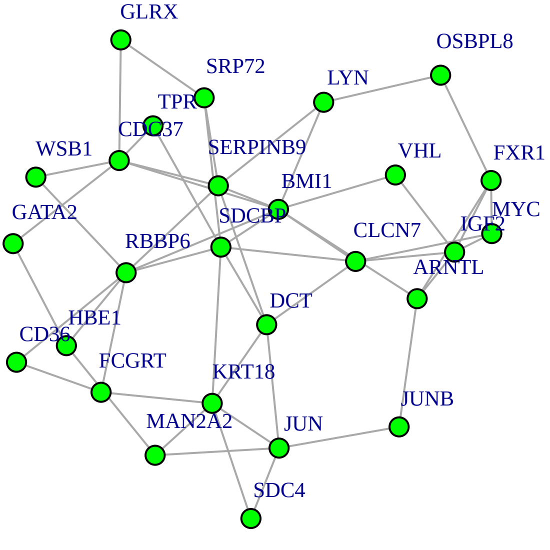

MST2 of the coexpression network for MUT p53

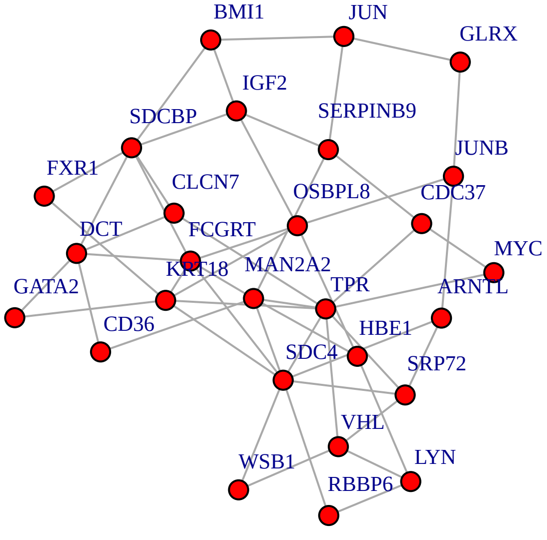

Pathway: LEE_LIVER_CANCER_E2F1_UP

There are 49 genes in this pathway. This pathway was detected by GSNCA

WT p53
Hub Gene (WT): ETS2
Weight Factor: 1.279
Hub Gene (MUT): SPARC
Weight Factor: 1.057

MUT p53
Hub Gene (MUT): SPARC
Weight Factor: 1.531
Hub Gene (WT): ETS2
Weight Factor: 0.99

MST2 of the coexpression network for WT p53

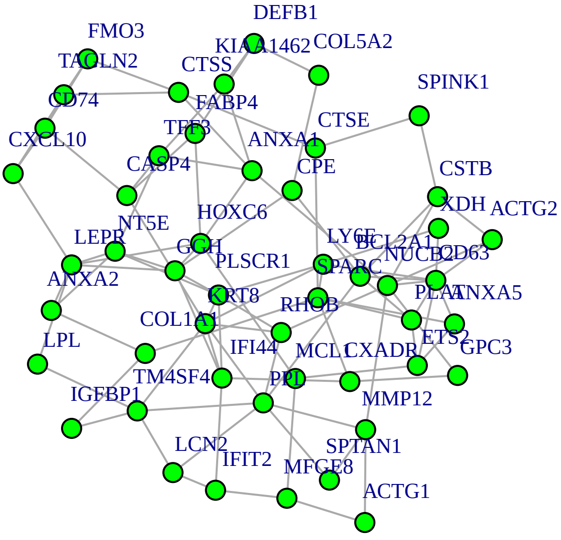

MST2 of the coexpression network for MUT p53

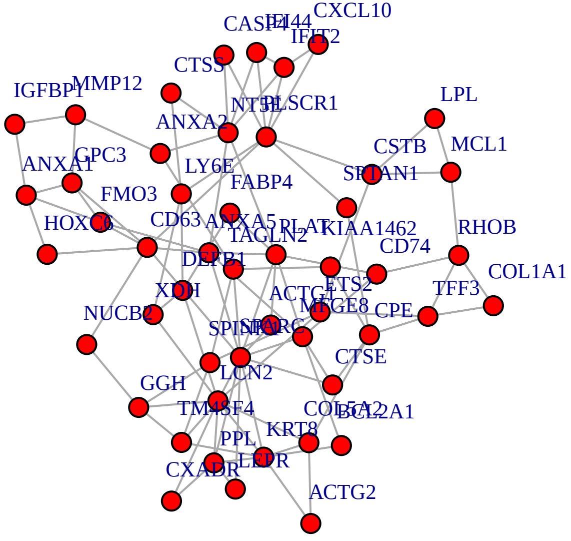

Pathway: HOFMANN_CELL_LYMPHOMA_DN

There are 33 genes in this pathway. This pathway was detected by GSNCA

WT p53
Hub Gene (WT): PRKG1
Weight Factor: 1.296
Hub Gene (MUT): TNFRSF1A
Weight Factor: 0.701

MUT p53
Hub Gene (MUT): TNFRSF1A
Weight Factor: 1.261
Hub Gene (WT): PRKG1
Weight Factor: 0.868

MST2 of the coexpression network for WT p53

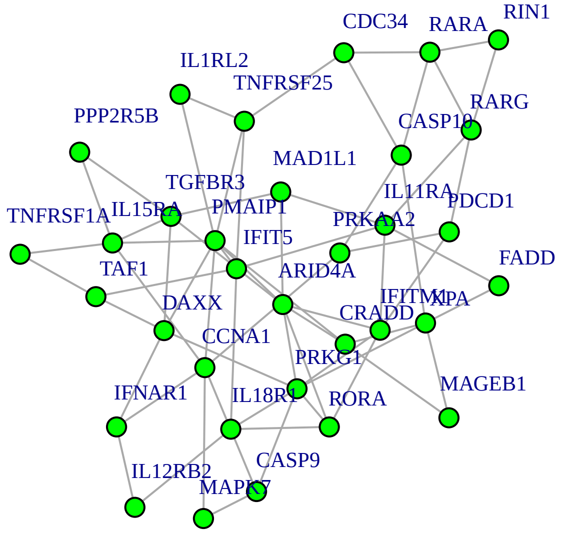

MST2 of the coexpression network for MUT p53

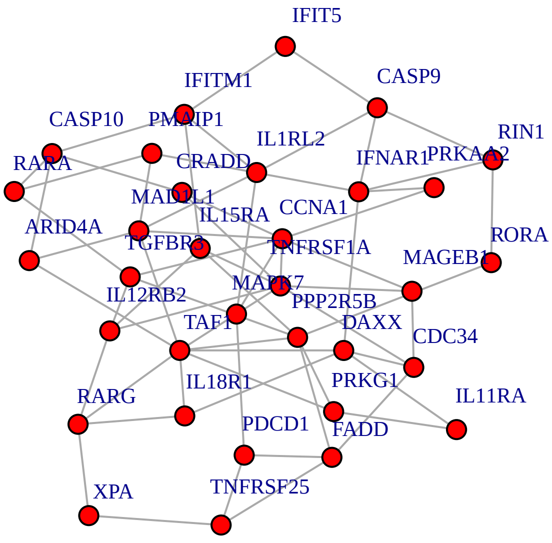

Pathway: FAELT_B_CLL_WITH_VH_REARRANGEMENTS_DN

There are 46 genes in this pathway. This pathway was detected by GSNCA

WT p53
Hub Gene (WT): PBX3
Weight Factor: 1.278
Hub Gene (MUT): MYLK
Weight Factor: 0.886

MUT p53
Hub Gene (MUT): MYLK
Weight Factor: 1.461
Hub Gene (WT): PBX3
Weight Factor: 0.959

MST2 of the coexpression network for WT p53

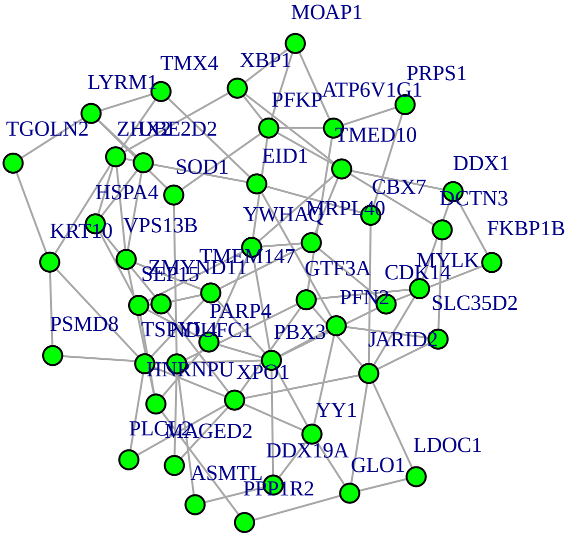

MST2 of the coexpression network for MUT p53

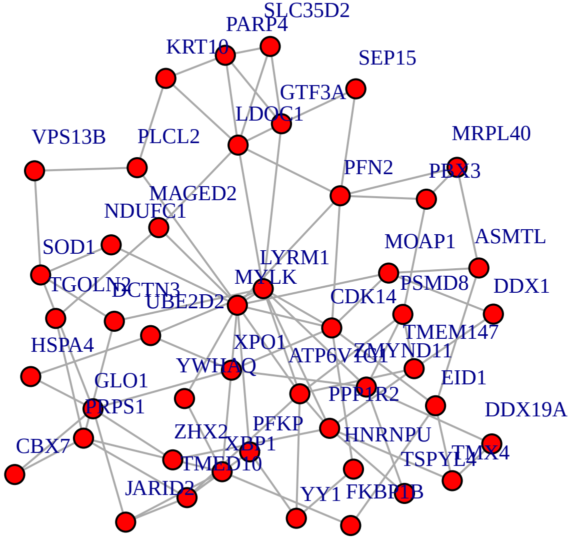

Pathway: YAO_HOXA10_TARGETS_VIA_PROGESTERONE_DN

There are 16 genes in this pathway. This pathway was detected by GSNCA

WT p53
Hub Gene (WT): HOXA10
Weight Factor: 1.4
Hub Gene (MUT): GJA1
Weight Factor: 1.115

MUT p53
Hub Gene (MUT): GJA1
Weight Factor: 1.407
Hub Gene (WT): HOXA10
Weight Factor: 0.61

MST2 of the coexpression network for WT p53

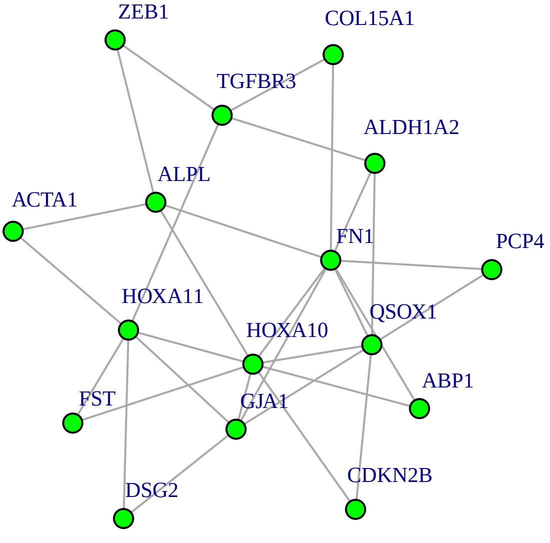

MST2 of the coexpression network for MUT p53

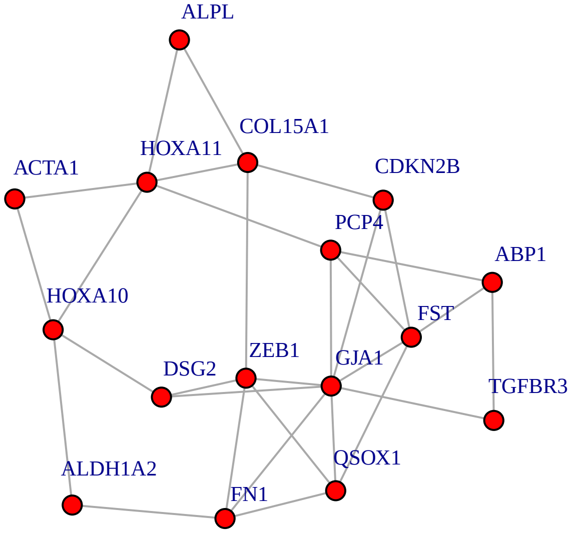

Pathway: DORSEY_GAB2_TARGETS

There are 21 genes in this pathway. This pathway was detected by GSNCA

WT p53
Hub Gene (WT): AKAP12
Weight Factor: 1.289
Hub Gene (MUT): GNG11
Weight Factor: 0.804

MUT p53
Hub Gene (MUT): GNG11
Weight Factor: 1.393
Hub Gene (WT): AKAP12
Weight Factor: 0.501

MST2 of the coexpression network for WT p53

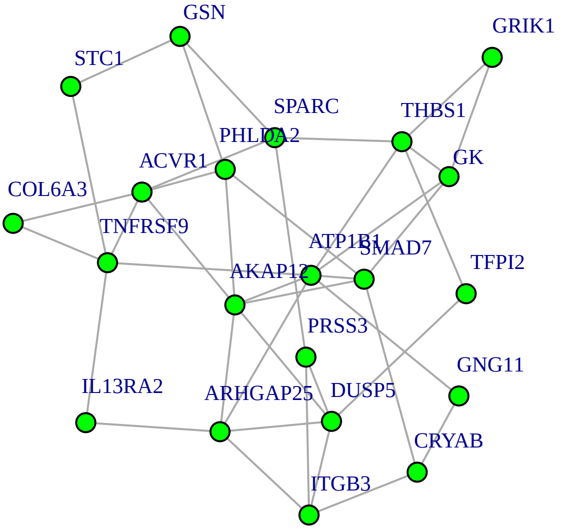

MST2 of the coexpression network for MUT p53

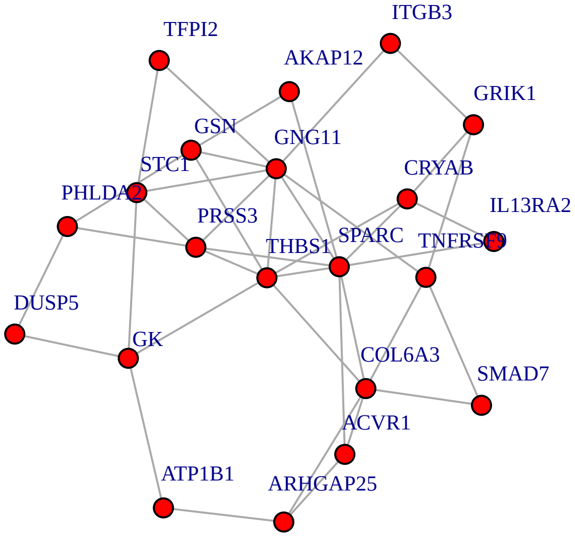

Pathway: KONDO_PROSTATE_CANCER_HCP_WITH_H3K27ME3

There are 54 genes in this pathway. This pathway was detected by GSNCA

WT p53	MUT p53
**Hub Gene (WT): EN2**	**Hub Gene (MUT): FBN1**
**Weight Factor: 1.331**	**Weight Factor: 1.464**
**Hub Gene (MUT): FBN1**	**Hub Gene (WT): EN2**
**Weight Factor: 0.78**	**Weight Factor: 0.882**

MST2 of the coexpression network for WT p53

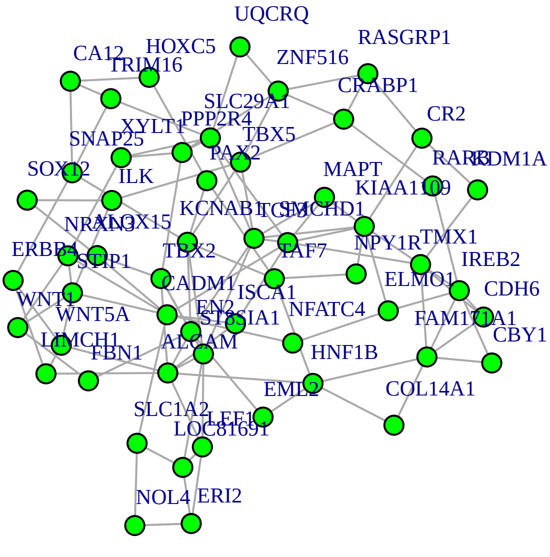

MST2 of the coexpression network for MUT p53

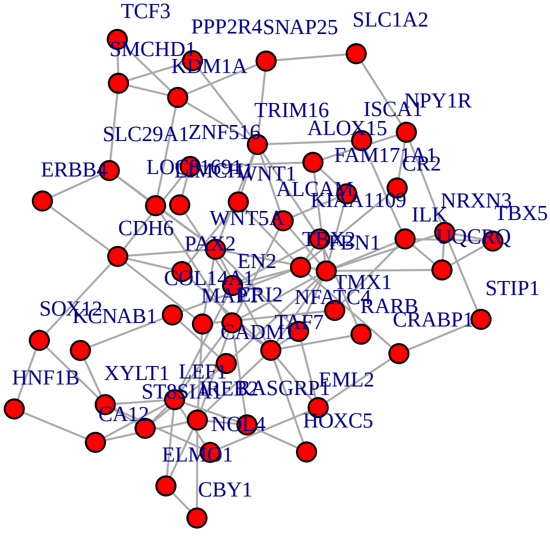

Pathway: MCCABE_BOUND_BY_HOXC6

There are 206 genes in this pathway. This pathway was detected by GSNCA

WT p53
Hub Gene (WT): CYTH3
Weight Factor: 1.309
Hub Gene (MUT): NDUFS3
Weight Factor: 1.086

MUT p53
Hub Gene (MUT): NDUFS3
Weight Factor: 1.461
Hub Gene (WT): CYTH3
Weight Factor: 0.966

MST2 of the coexpression network for WT p53

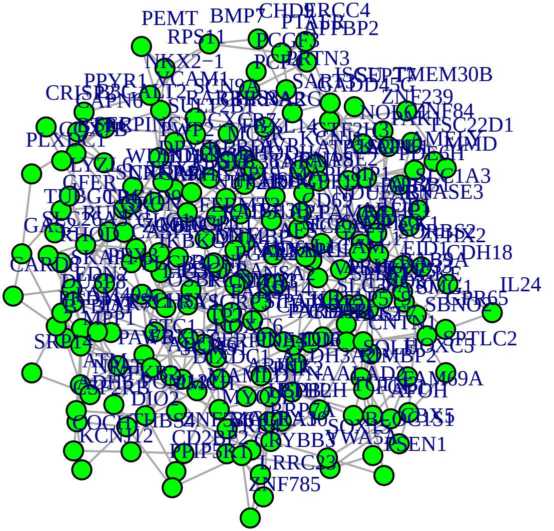

MST2 of the coexpression network for MUT p53

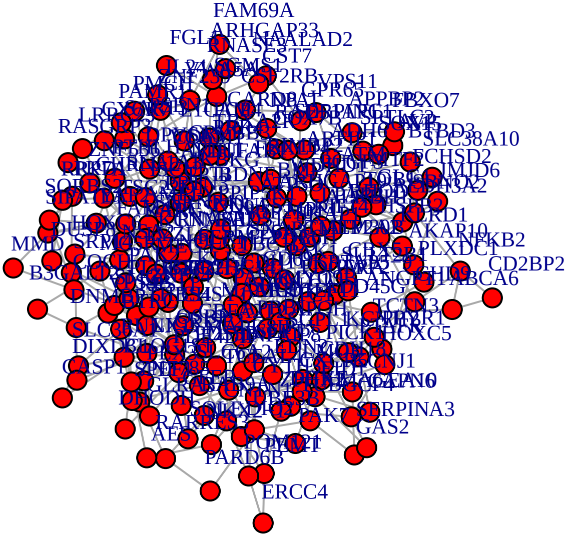

Pathway: YAMASHITA_METHYLATED_IN_PROSTATE_CANCER

There are 41 genes in this pathway. This pathway was detected by GSNCA

WT p53
Hub Gene (WT): TFPI
Weight Factor: 1.448
Hub Gene (MUT): SERPINE1
Weight Factor: 1.254

MUT p53
Hub Gene (MUT): SERPINE1
Weight Factor: 1.473
Hub Gene (WT): TFPI
Weight Factor: 1.133

MST2 of the coexpression network for WT p53

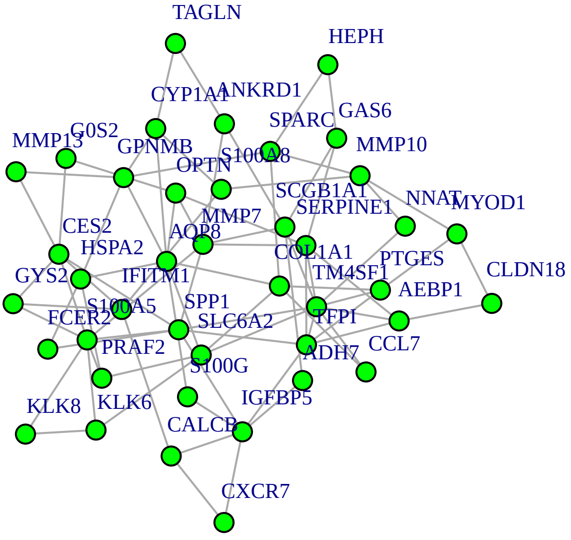

MST2 of the coexpression network for MUT p53

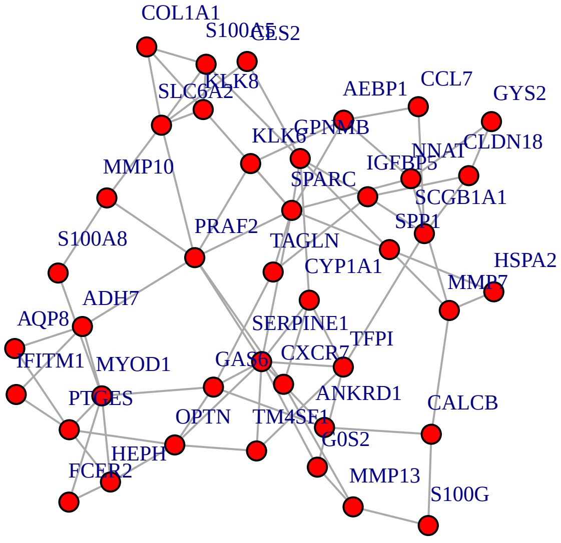

Pathway: ENGELMANN_CANCER_PROGENITORS_UP

There are 36 genes in this pathway. This pathway was detected by GSNCA

WT p53
Hub Gene (WT): CTSZ
Weight Factor: 1.216
Hub Gene (MUT): ANXA9
Weight Factor: 1.166

MUT p53
Hub Gene (MUT): ANXA9
Weight Factor: 1.435
Hub Gene (WT): CTSZ
Weight Factor: 0.717

MST2 of the coexpression network for WT p53

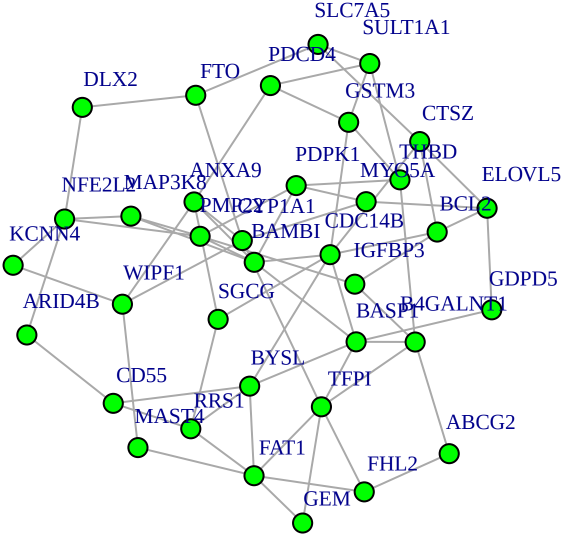

MST2 of the coexpression network for MUT p53

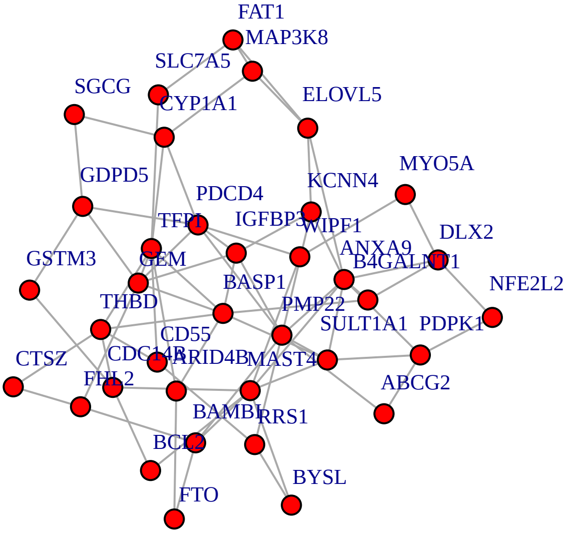

Pathway: MASRI_RESISTANCE_TO_TAMOXIFEN_AND_AROMATASE_INHIBITORS_UP

There are 18 genes in this pathway. This pathway was detected by GSNCA

WT p53
Hub Gene (WT): TFF1
Weight Factor: 1.402
Hub Gene (MUT): AGR2
Weight Factor: 0.804

MUT p53
Hub Gene (MUT): AGR2
Weight Factor: 1.345
Hub Gene (WT): TFF1
Weight Factor: 0.989

MST2 of the coexpression network for WT p53

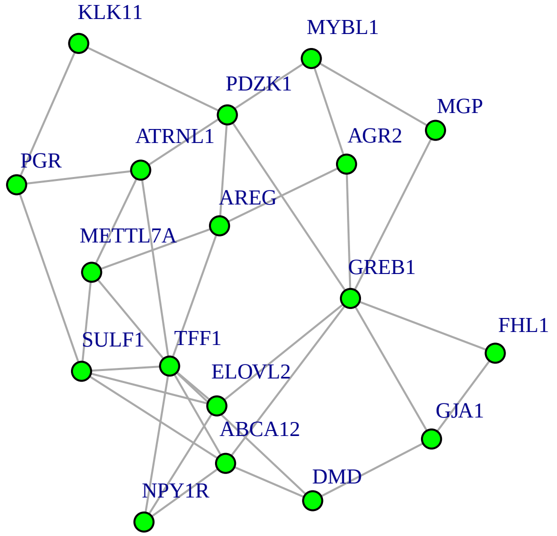

MST2 of the coexpression network for MUT p53

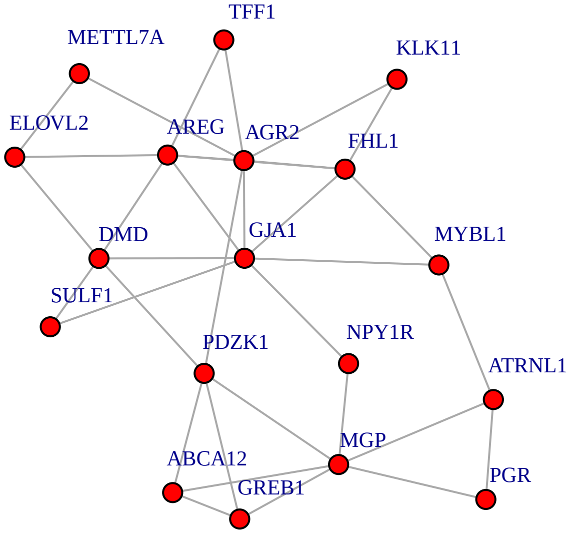

Pathway: SMID_BREAST_CANCER_RELAPSE_IN_BONE_DN

There are 248 genes in this pathway. This pathway was detected by GSNCA

WT p53
Hub Gene (WT): GPM6B
Weight Factor: 1.456
Hub Gene (MUT): FLNA
Weight Factor: 1.058

MUT p53
Hub Gene (MUT): FLNA
Weight Factor: 1.586
Hub Gene (WT): GPM6B
Weight Factor: 1.225

MST2 of the coexpression network for WT p53

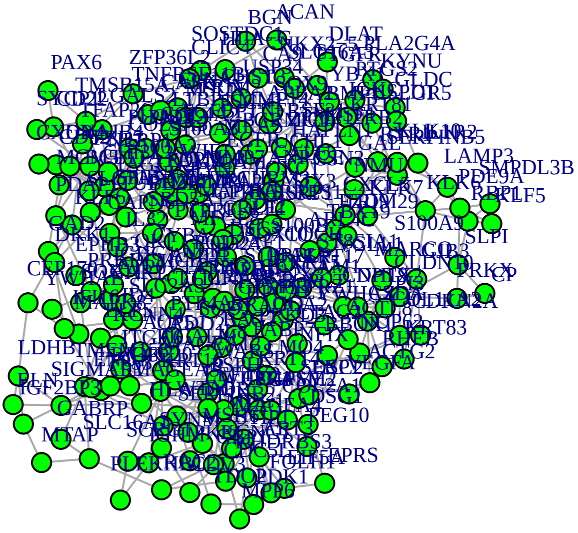

MST2 of the coexpression network for MUT p53

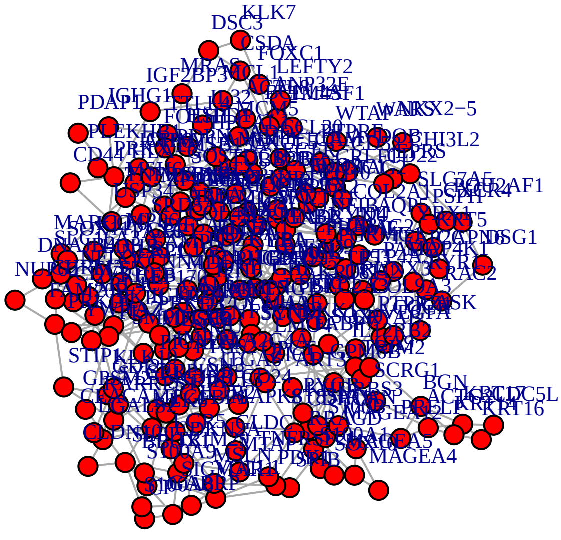

Pathway: SMID_BREAST_CANCER_ERBB2_UP

There are 110 genes in this pathway. This pathway was detected by GSNCA

WT p53
Hub Gene (WT): KIAA0182
Weight Factor: 1.278
Hub Gene (MUT): SCNN1A
Weight Factor: 0.944

MUT p53
Hub Gene (MUT): SCNN1A
Weight Factor: 1.55
Hub Gene (WT): KIAA0182
Weight Factor: 1.16

MST2 of the coexpression network for WT p53

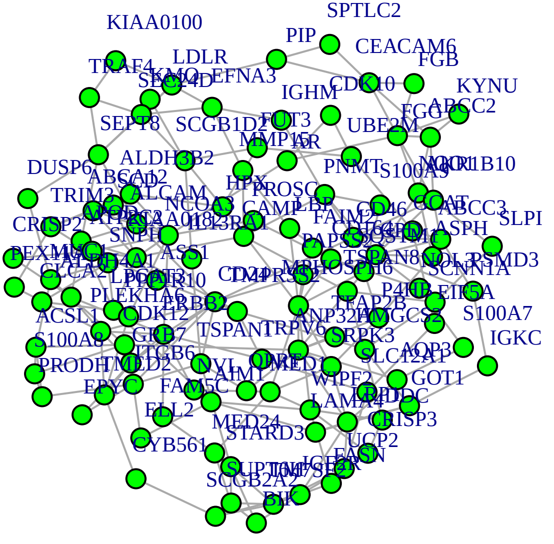

MST2 of the coexpression network for MUT p53

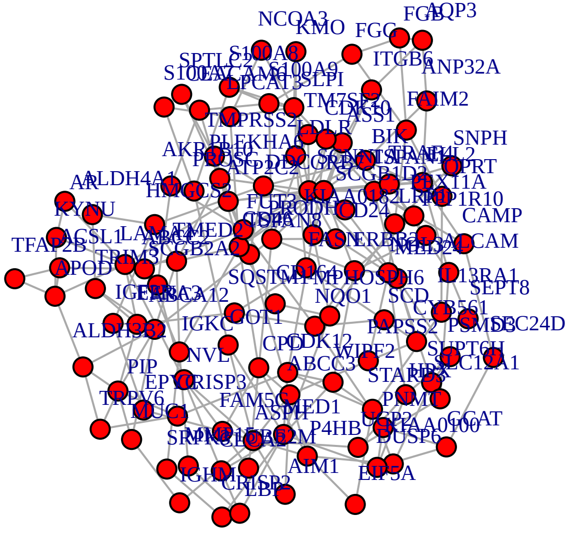

Pathway: IZADPANAH_STEM_CELL_ADIPOSE_VS_BONE_DN

There are 73 genes in this pathway. This pathway was detected by GSNCA

WT p53
Hub Gene (WT): LAMB1
Weight Factor: 1.44
Hub Gene (MUT): LOXL2
Weight Factor: 1.275

MUT p53
Hub Gene (MUT): LOXL2
Weight Factor: 1.55
Hub Gene (WT): LAMB1
Weight Factor: 1.412

MST2 of the coexpression network for WT p53

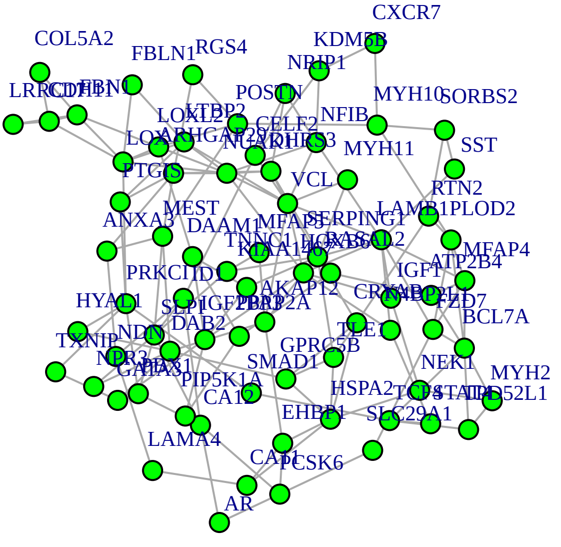

MST2 of the coexpression network for MUT p53

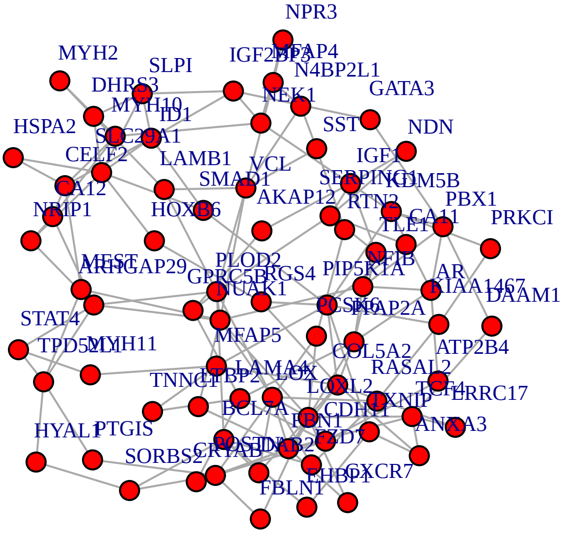

Pathway: HONMA_DOCETAXEL_RESISTANCE

There are 20 genes in this pathway. This pathway was detected by GSNCA

WT p53
Hub Gene (WT): COX7C
Weight Factor: 1.27
Hub Gene (MUT): CFL1
Weight Factor: 0.83

MUT p53
Hub Gene (MUT): CFL1
Weight Factor: 1.341
Hub Gene (WT): COX7C
Weight Factor: 1.269

MST2 of the coexpression network for WT p53

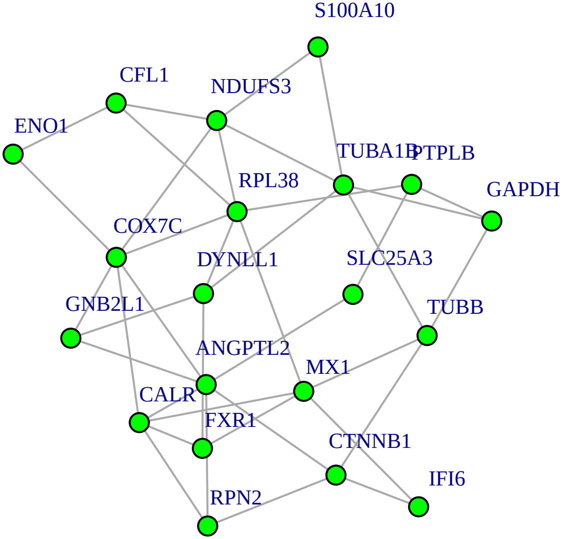

MST2 of the coexpression network for MUT p53

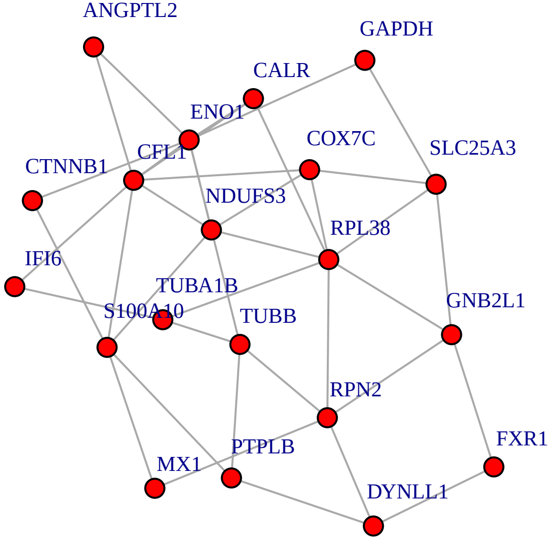

Pathway: GRADE_COLON_AND_RECTAL_CANCER_DN

There are 54 genes in this pathway. This pathway was detected by GSNCA

WT p53
Hub Gene (WT): MITF
Weight Factor: 1.435
Hub Gene (MUT): GPC1
Weight Factor: 0.723

MUT p53
Hub Gene (MUT): GPC1
Weight Factor: 1.453
Hub Gene (WT): MITF
Weight Factor: 0.985

MST2 of the coexpression network for WT p53

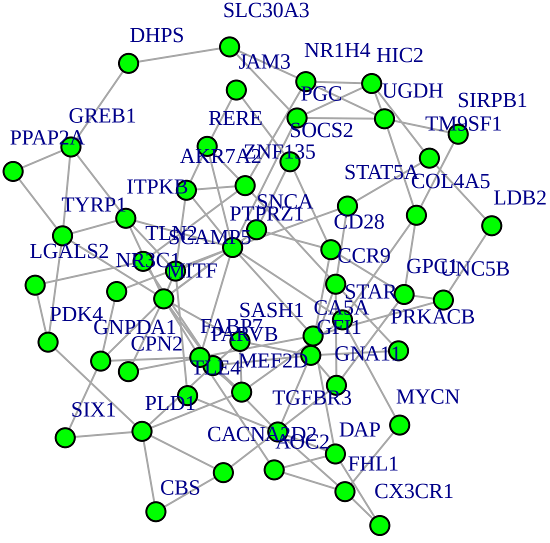

MST2 of the coexpression network for MUT p53

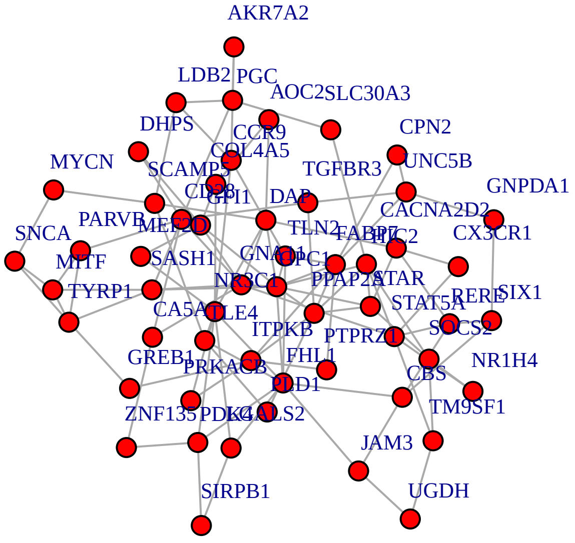

Pathway: SHARMA_PILOCYTIC_ASTROCYTOMA_LOCATION_UP

There are 17 genes in this pathway. This pathway was detected by GSNCA

WT p53
Hub Gene (WT): GPR98
Weight Factor: 1.29
Hub Gene (MUT): MSR1
Weight Factor: 0.533

MUT p53
Hub Gene (MUT): MSR1
Weight Factor: 1.365
Hub Gene (WT): GPR98
Weight Factor: 0.656

MST2 of the coexpression network for WT p53

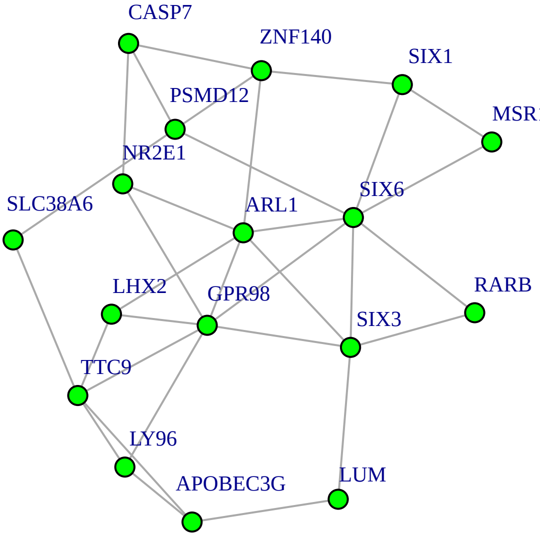

MST2 of the coexpression network for MUT p53

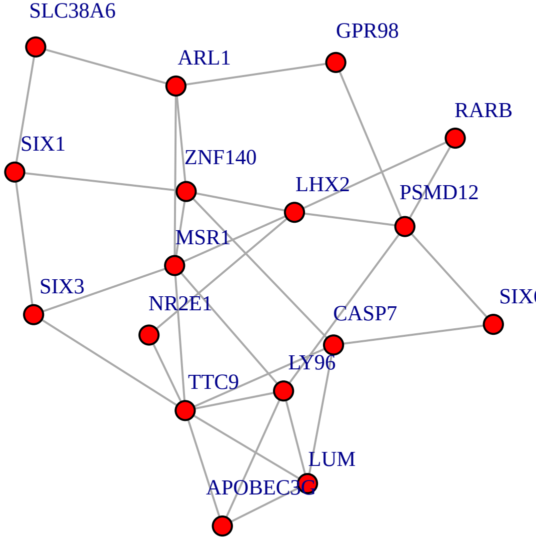

Pathway: BRUECKNER_TARGETS_OF_MIRLET7A3_DN

There are 54 genes in this pathway. This pathway was detected by GSNCA

WT p53
Hub Gene (WT): RIN2
Weight Factor: 1.382
Hub Gene (MUT): FN1
Weight Factor: 1.185

MUT p53
Hub Gene (MUT): FN1
Weight Factor: 1.439
Hub Gene (WT): RIN2
Weight Factor: 1.081

MST2 of the coexpression network for WT p53

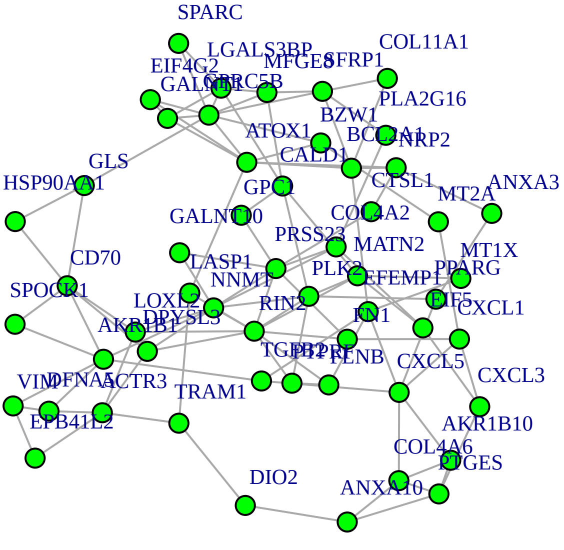

MST2 of the coexpression network for MUT p53

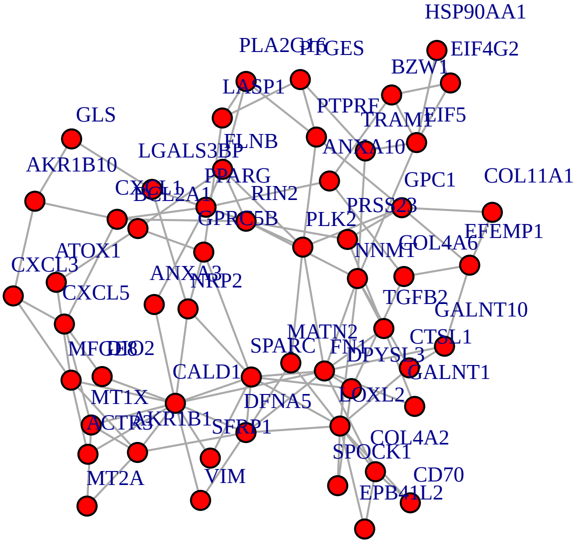

Pathway: BREDEMEYER_RAG_SIGNALING_NOT_VIA_ATM_UP

There are 28 genes in this pathway. This pathway was detected by GSNCA

WT p53		MUT p53	
**Hub Gene (WT):**	**RCAN1**	**Hub Gene (MUT):**	**RAB32**
**Weight Factor:**	**1.197**	**Weight Factor:**	**1.395**
**Hub Gene (MUT):**	**RAB32**	**Hub Gene (WT):**	**RCAN1**
**Weight Factor:**	**0.973**	**Weight Factor:**	**1.097**

MST2 of the coexpression network for WT p53

MST2 of the coexpression network for MUT p53

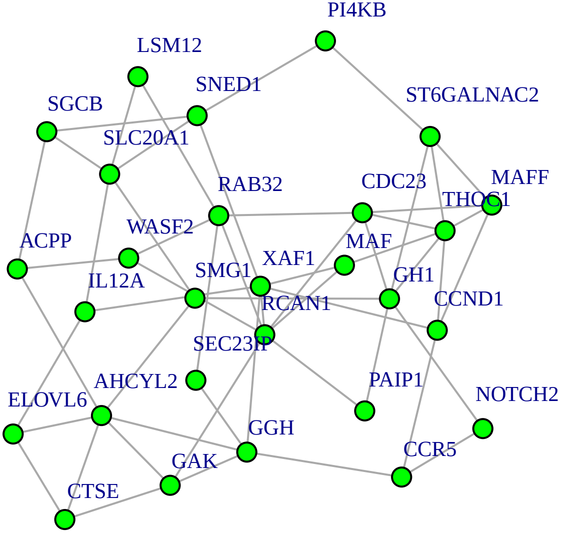

Pathway: GU_PDEF_TARGETS_UP

There are 65 genes in this pathway. This pathway was detected by GSNCA

WT p53
Hub Gene (WT): TGFBI
Weight Factor: 1.383
Hub Gene (MUT): COL4A1
Weight Factor: 1.379

MUT p53
Hub Gene (MUT): COL4A1
Weight Factor: 1.535
Hub Gene (WT): TGFBI
Weight Factor: 1.219

MST2 of the coexpression network for WT p53

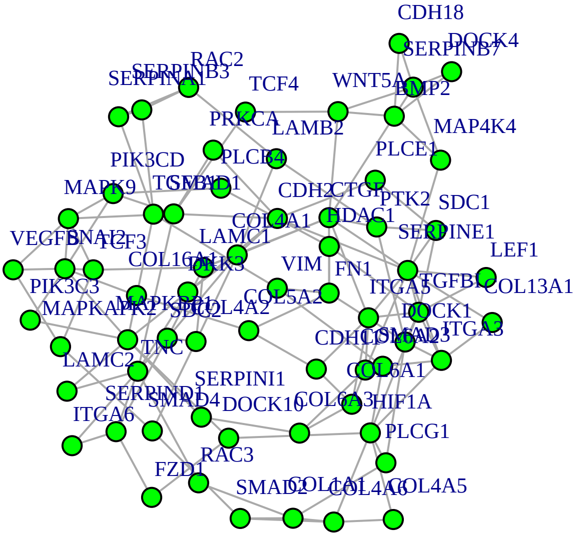

MST2 of the coexpression network for MUT p53

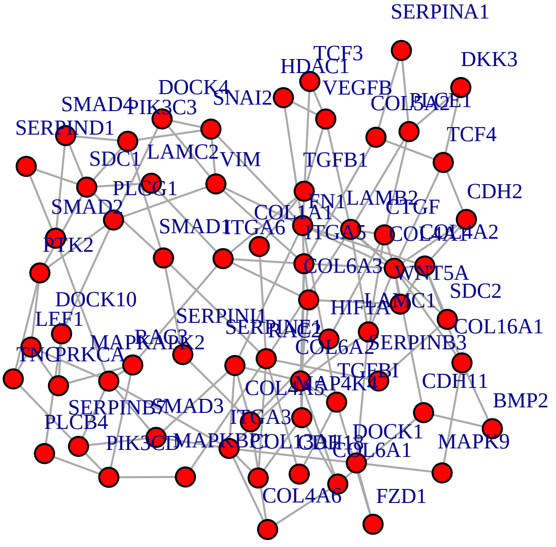

Pathway: LEE_EARLY_T_LYMPHOCYTE_DN

There are 32 genes in this pathway. This pathway was detected by GSNCA

WT p53
Hub Gene (WT): IL4R
Weight Factor: 1.357
Hub Gene (MUT): HLA−F
Weight Factor: 1.111

MUT p53
Hub Gene (MUT): HLA−F
Weight Factor: 1.523
Hub Gene (WT): IL4R
Weight Factor: 0.831

MST2 of the coexpression network for WT p53

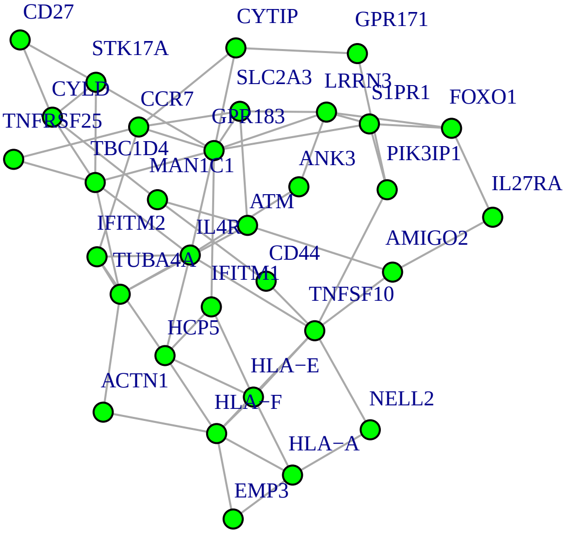

MST2 of the coexpression network for MUT p53

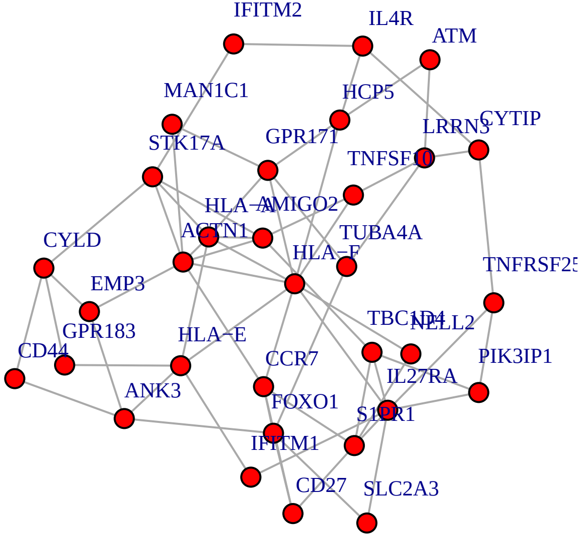

Pathway: VALK_AML_CLUSTER_8

There are 20 genes in this pathway. This pathway was detected by GSNCA

WT p53	
Hub Gene (WT):	TRAK2
Weight Factor:	1.324
Hub Gene (MUT):	DCAF11
Weight Factor:	1.119

MUT p53	
Hub Gene (MUT):	DCAF11
Weight Factor:	1.393
Hub Gene (WT):	TRAK2
Weight Factor:	1.113

MST2 of the coexpression network for WT p53

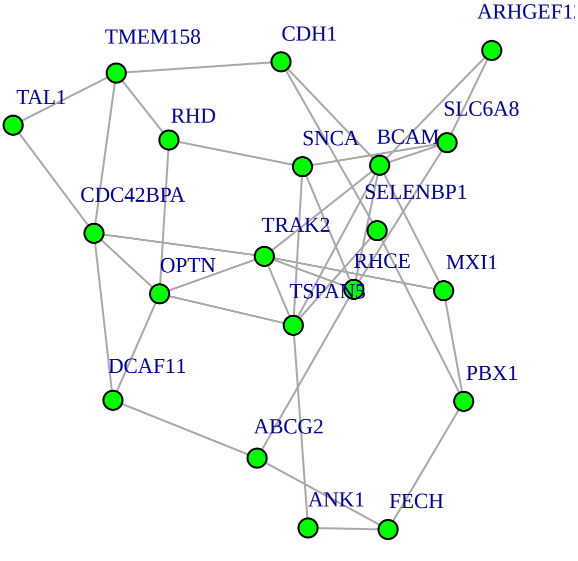

MST2 of the coexpression network for MUT p53

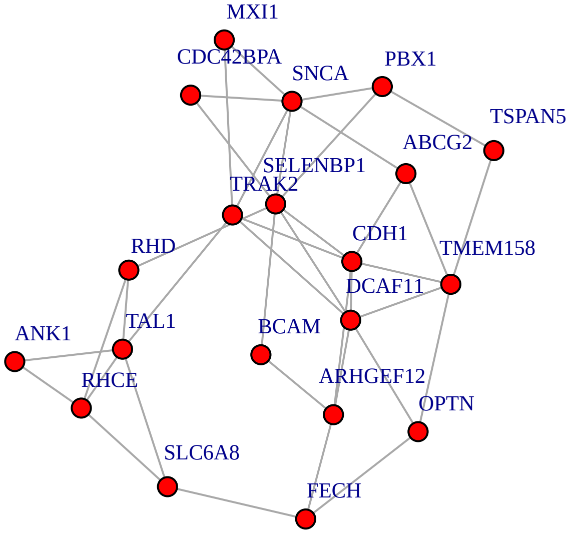

Pathway: BOYAULT_LIVER_CANCER_SUBCLASS_G1_DN

There are 35 genes in this pathway. This pathway was detected by GSNCA

WT p53
Hub Gene (WT): SDHB
Weight Factor: 1.36
Hub Gene (MUT): GBE1
Weight Factor: 0.801

MUT p53
Hub Gene (MUT): GBE1
Weight Factor: 1.426
Hub Gene (WT): SDHB
Weight Factor: 1.014

MST2 of the coexpression network for WT p53

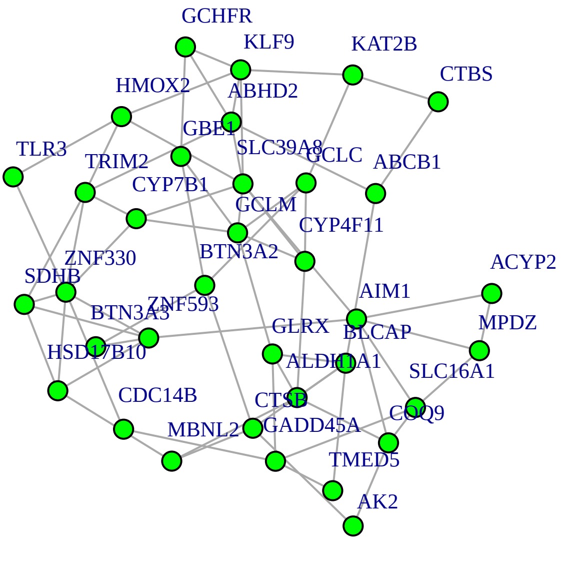

MST2 of the coexpression network for MUT p53

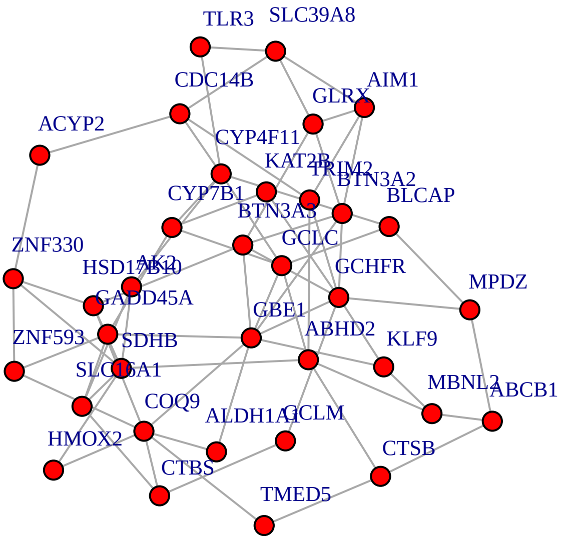

Pathway: BOYAULT_LIVER_CANCER_SUBCLASS_G2

There are 18 genes in this pathway. This pathway was detected by GSNCA

WT p53
Hub Gene (WT): AMIGO2
Weight Factor: 1.318
Hub Gene (MUT): ITGA5
Weight Factor: 1.159

MUT p53
Hub Gene (MUT): ITGA5
Weight Factor: 1.471
Hub Gene (WT): AMIGO2
Weight Factor: 0.982

MST2 of the coexpression network for WT p53

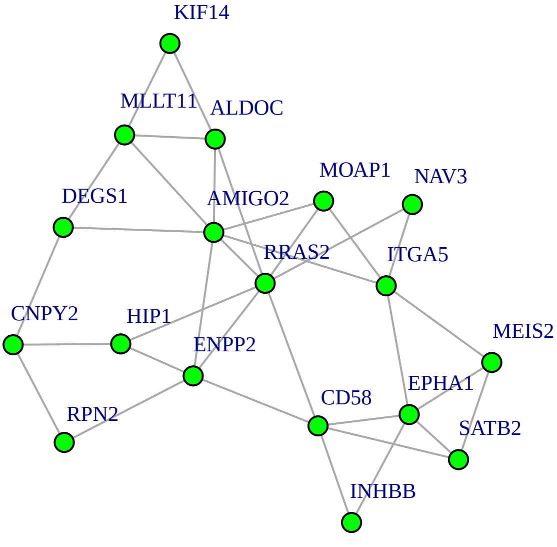

MST2 of the coexpression network for MUT p53

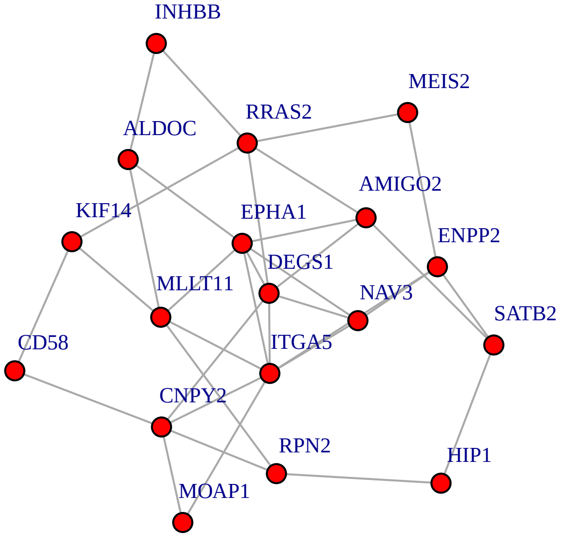

Pathway: CHIANG_LIVER_CANCER_SUBCLASS_CTNNB1_DN

There are 104 genes in this pathway. This pathway was detected by GSNCA

WT p53
Hub Gene (WT): ACP5
Weight Factor: 1.396
Hub Gene (MUT): GCH1
Weight Factor: 1.202

MUT p53
Hub Gene (MUT): GCH1
Weight Factor: 1.497
Hub Gene (WT): ACP5
Weight Factor: 0.825

MST2 of the coexpression network for WT p53

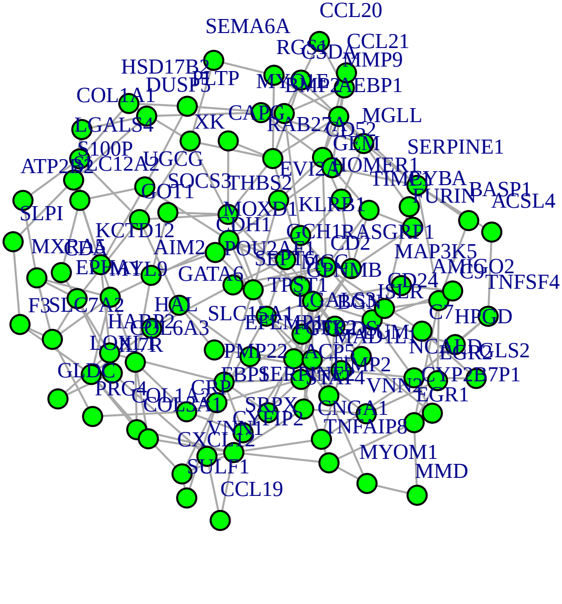

MST2 of the coexpression network for MUT p53

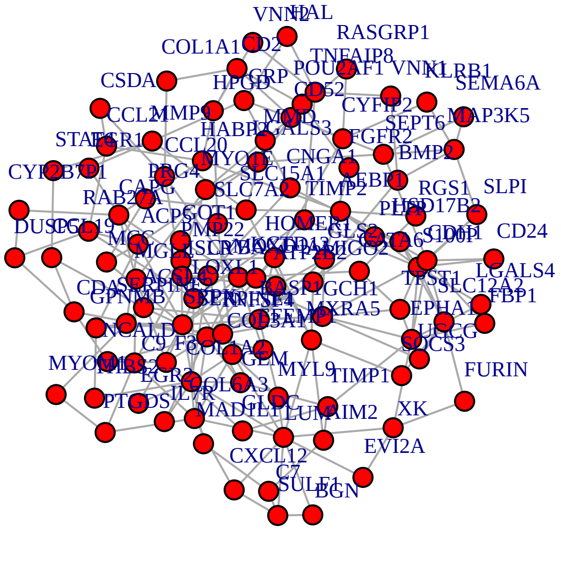

Pathway: CAIRO_LIVER_DEVELOPMENT_DN

There are 176 genes in this pathway. This pathway was detected by GSNCA

WT p53
Hub Gene (WT): GRIK5
Weight Factor: 1.367
Hub Gene (MUT): CDH1
Weight Factor: 0.989

MUT p53
Hub Gene (MUT): CDH1
Weight Factor: 1.512
Hub Gene (WT): GRIK5
Weight Factor: 0.89

MST2 of the coexpression network for WT p53

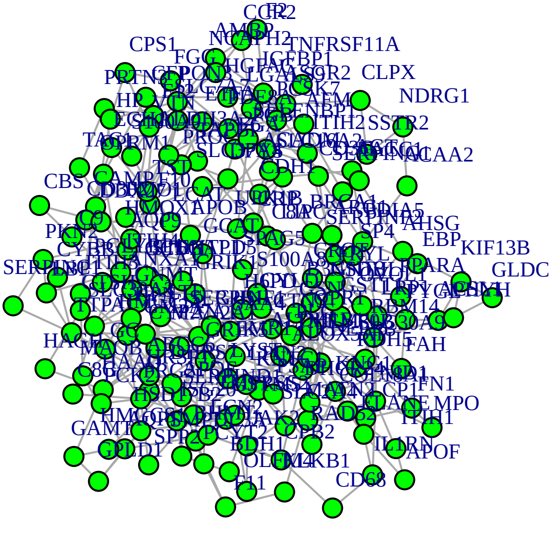

MST2 of the coexpression network for MUT p53

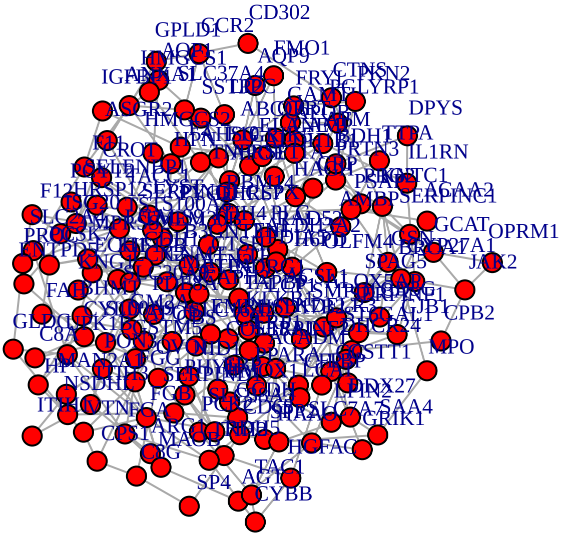

Pathway: DAZARD_UV_RESPONSE_CLUSTER_G24

There are 17 genes in this pathway. This pathway was detected by GSNCA

WT p53
Hub Gene (WT): ECM1
Weight Factor: 1.412
Hub Gene (MUT): CLDN4
Weight Factor: 1.021

MUT p53
Hub Gene (MUT): CLDN4
Weight Factor: 1.494
Hub Gene (WT): ECM1
Weight Factor: 0.965

MST2 of the coexpression network for WT p53

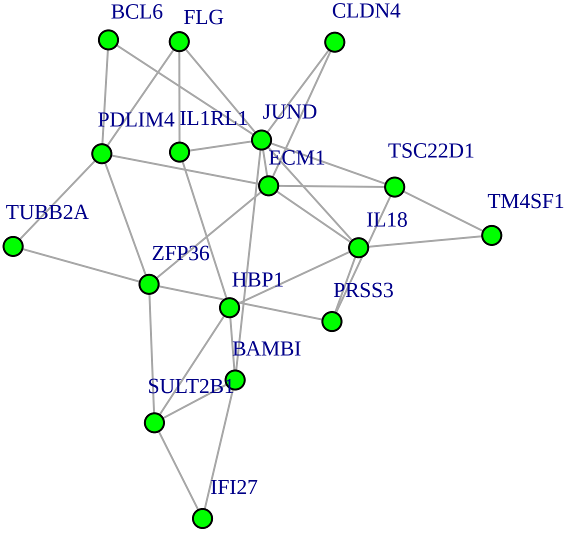

MST2 of the coexpression network for MUT p53

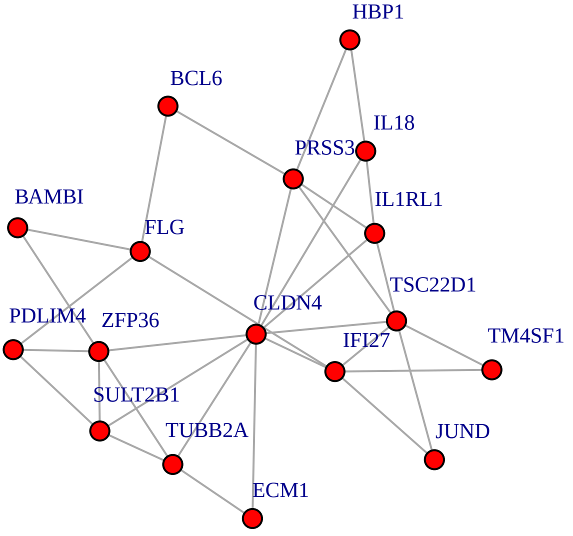

Pathway: BROWNE_HCMV_INFECTION_30MIN_UP

There are 51 genes in this pathway. This pathway was detected by GSNCA

WT p53
Hub Gene (WT): CAMK2G
Weight Factor: 1.274
Hub Gene (MUT): COL6A2
Weight Factor: 1.179

MUT p53
Hub Gene (MUT): COL6A2
Weight Factor: 1.516
Hub Gene (WT): CAMK2G
Weight Factor: 0.947

MST2 of the coexpression network for WT p53

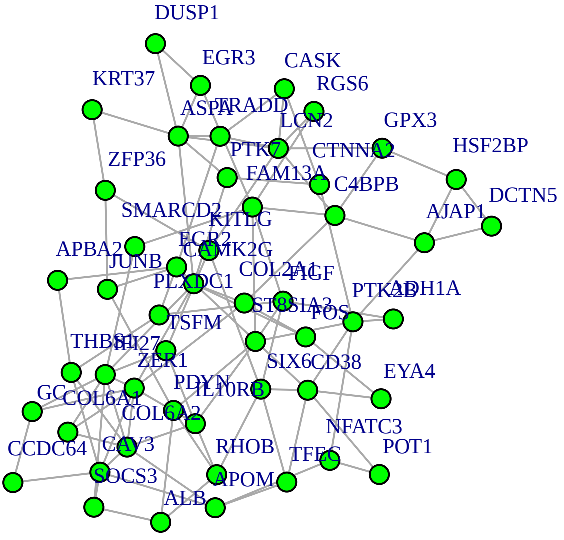

MST2 of the coexpression network for MUT p53

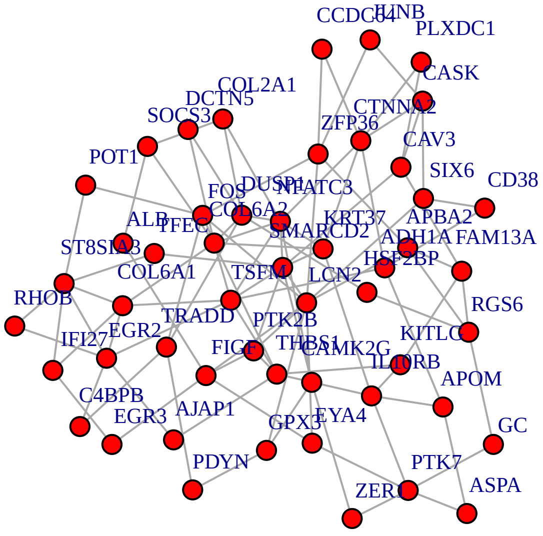

Pathway: BROWNE_HCMV_INFECTION_1HR_DN

There are 209 genes in this pathway. This pathway was detected by GSNCA

WT p53
Hub Gene (WT): YTHDC1
Weight Factor: 1.395
Hub Gene (MUT): DDX23
Weight Factor: 1.262

MUT p53
Hub Gene (MUT): DDX23
Weight Factor: 1.437
Hub Gene (WT): YTHDC1
Weight Factor: 1.214

MST2 of the coexpression network for WT p53

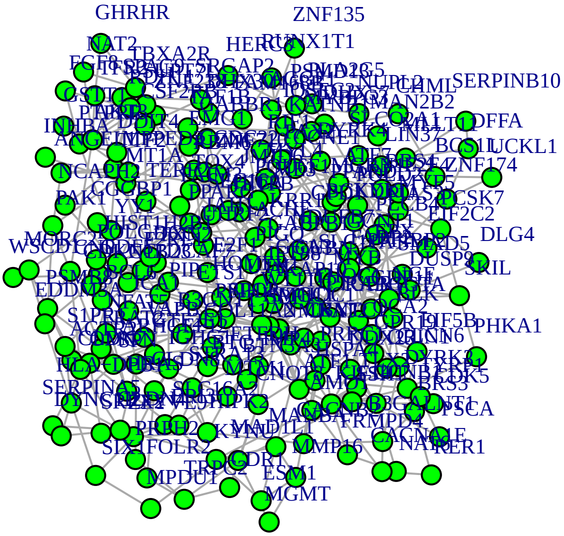

MST2 of the coexpression network for MUT p53

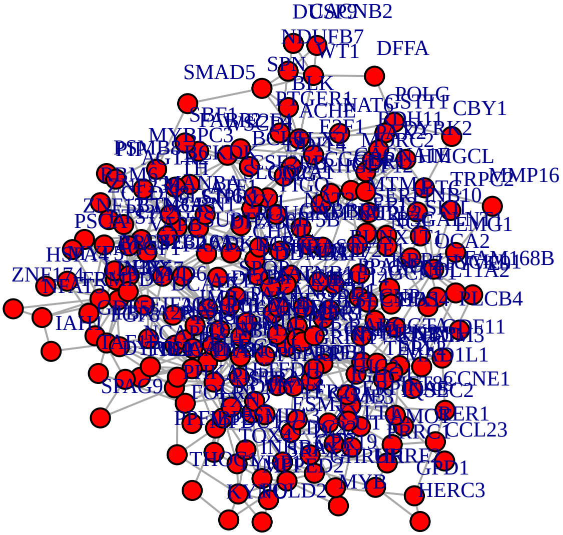

Pathway: BROWNE_HCMV_INFECTION_4HR_UP

There are 51 genes in this pathway. This pathway was detected by GSNCA

WT p53
Hub Gene (WT): GPRC5B
Weight Factor: 1.257
Hub Gene (MUT): IFIT2
Weight Factor: 1.137

MUT p53
Hub Gene (MUT): IFIT2
Weight Factor: 1.372
Hub Gene (WT): GPRC5B
Weight Factor: 1.033

MST2 of the coexpression network for WT p53

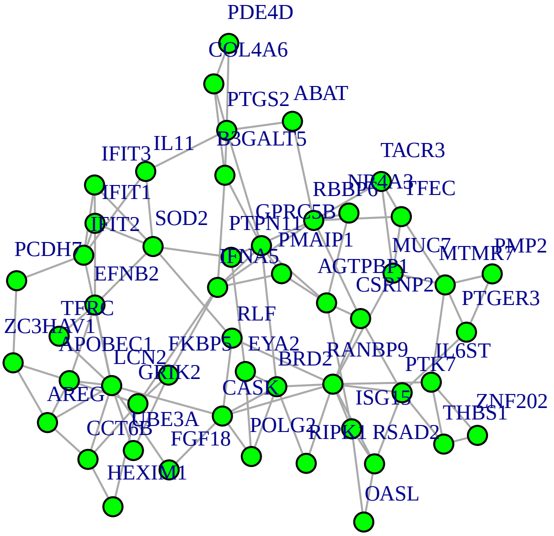

MST2 of the coexpression network for MUT p53

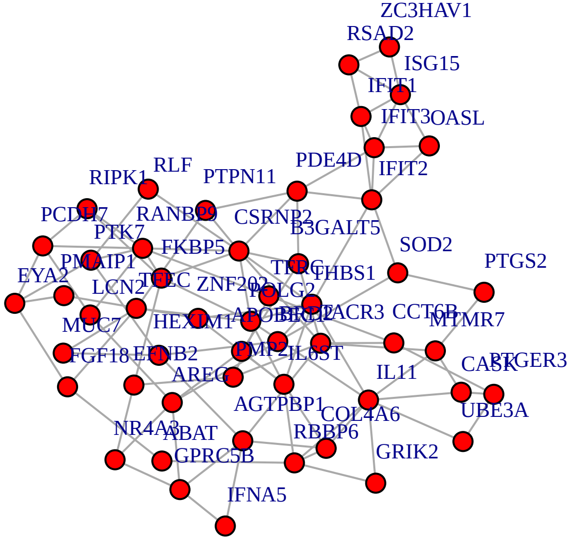

Pathway: KAAB_HEART_ATRIUM_VS_VENTRICLE_UP

There are 242 genes in this pathway. This pathway was detected by GSNCA

WT p53
Hub Gene (WT): PLP1
Weight Factor: 1.374
Hub Gene (MUT): MYLK
Weight Factor: 1.169

MUT p53
Hub Gene (MUT): MYLK
Weight Factor: 1.582
Hub Gene (WT): PLP1
Weight Factor: 0.883

MST2 of the coexpression network for WT p53

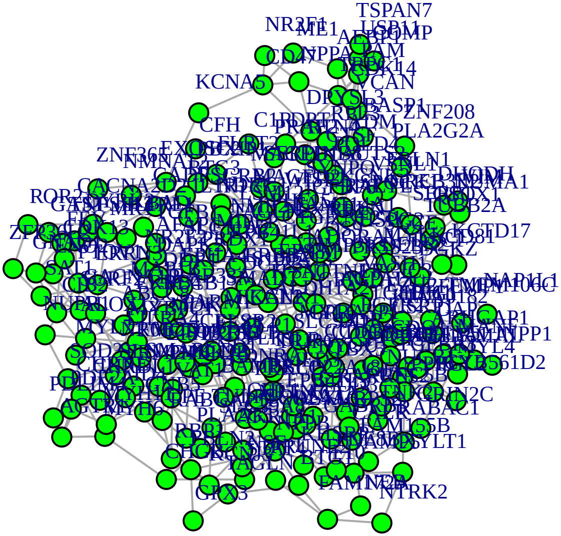

MST2 of the coexpression network for MUT p53

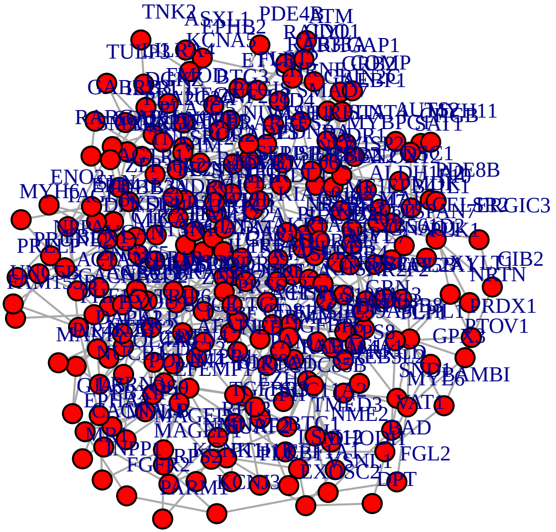

Pathway: WANG_SMARCE1_TARGETS_UP

There are 136 genes in this pathway. This pathway was detected by GSNCA

**WT p53**	**MUT p53**
**Hub Gene (WT): ITGB5**	**Hub Gene (MUT): FN1**
**Weight Factor: 1.334**	**Weight Factor: 1.576**
**Hub Gene (MUT): FN1**	**Hub Gene (WT): ITGB5**
**Weight Factor: 1.059**	**Weight Factor: 1.362**

MST2 of the coexpression network for WT p53

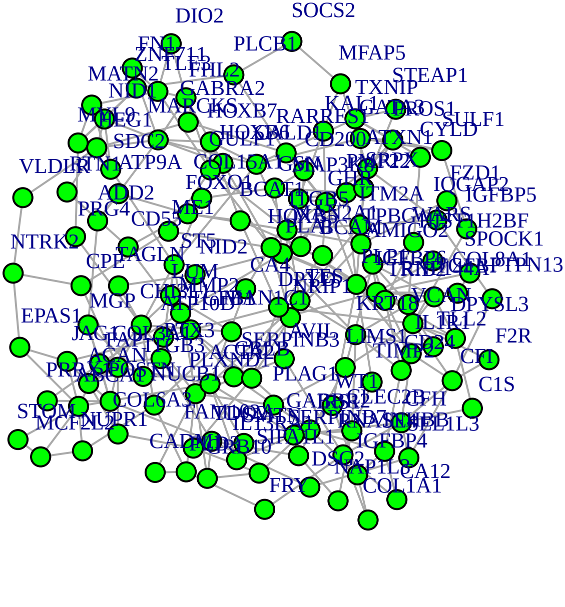

MST2 of the coexpression network for MUT p53

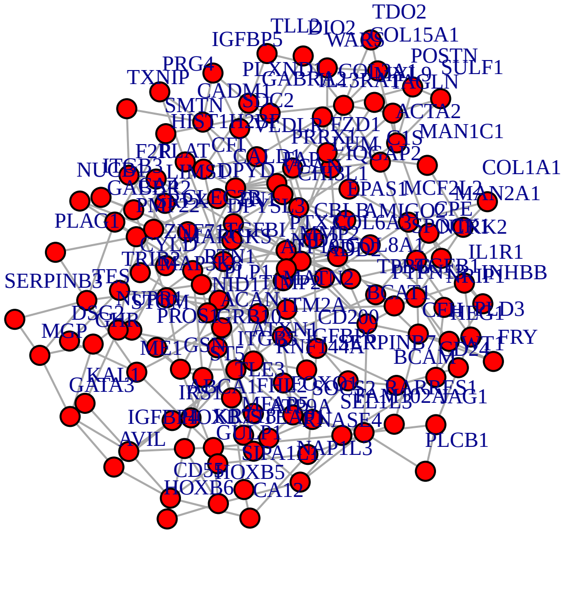

Pathway: BROWNE_HCMV_INFECTION_24HR_DN

There are 142 genes in this pathway. This pathway was detected by GSNCA

WT p53
Hub Gene (WT): CAV1
Weight Factor: 1.396
Hub Gene (MUT): FBN1
Weight Factor: 0.892

MUT p53
Hub Gene (MUT): FBN1
Weight Factor: 1.615
Hub Gene (WT): CAV1
Weight Factor: 1.606

MST2 of the coexpression network for WT p53

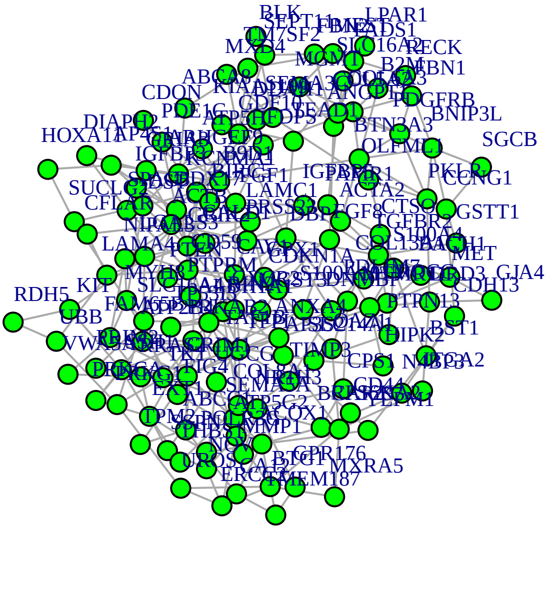

MST2 of the coexpression network for MUT p53

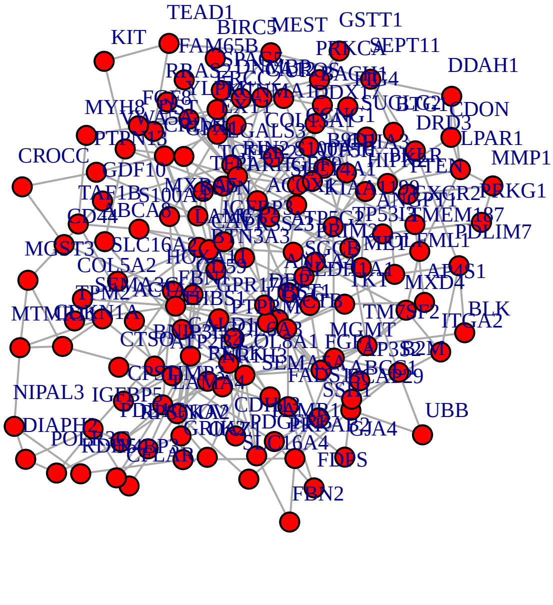

Pathway: MCCLUNG_DELTA_FOSB_TARGETS_8WK

There are 35 genes in this pathway. This pathway was detected by GSNCA

**WT p53**	**MUT p53**
**Hub Gene (WT): NDST1**	**Hub Gene (MUT): ARF3**
**Weight Factor: 1.273**	**Weight Factor: 1.43**
**Hub Gene (MUT): ARF3**	**Hub Gene (WT): NDST1**
**Weight Factor: 0.86**	**Weight Factor: 1.193**

MST2 of the coexpression network for WT p53

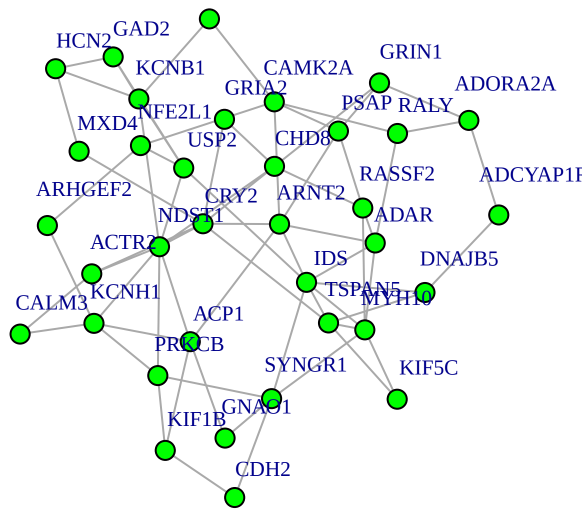

MST2 of the coexpression network for MUT p53

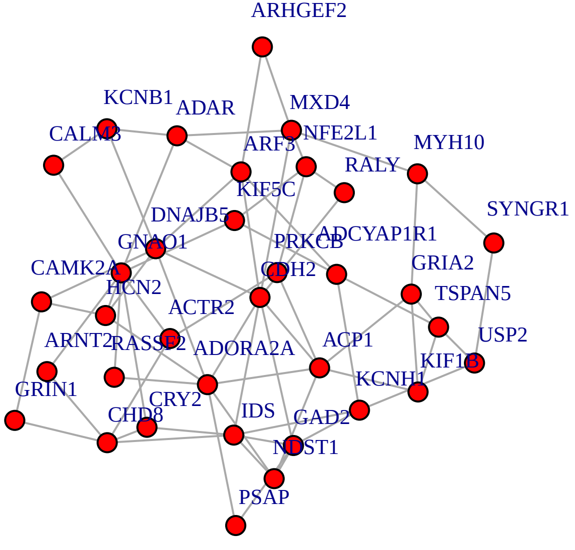

Pathway: GAJATE_RESPONSE_TO_TRABECTEDIN_DN

There are 16 genes in this pathway. This pathway was detected by GSNCA

WT p53
Hub Gene (WT): STAG1
Weight Factor: 1.392
Hub Gene (MUT): CDK14
Weight Factor: 0.718

MUT p53
Hub Gene (MUT): CDK14
Weight Factor: 1.416
Hub Gene (WT): STAG1
Weight Factor: 0.745

MST2 of the coexpression network for WT p53

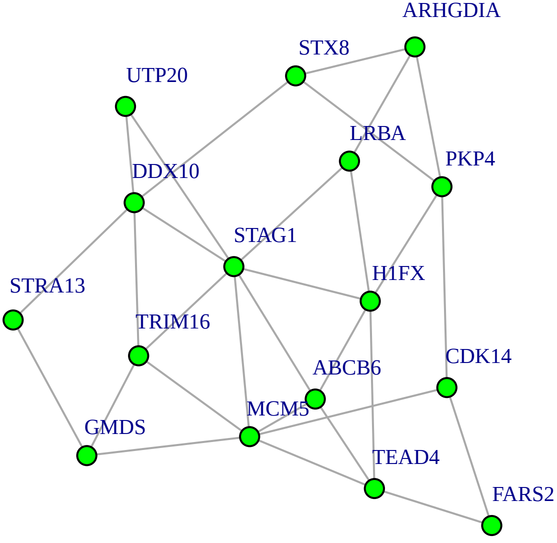

MST2 of the coexpression network for MUT p53

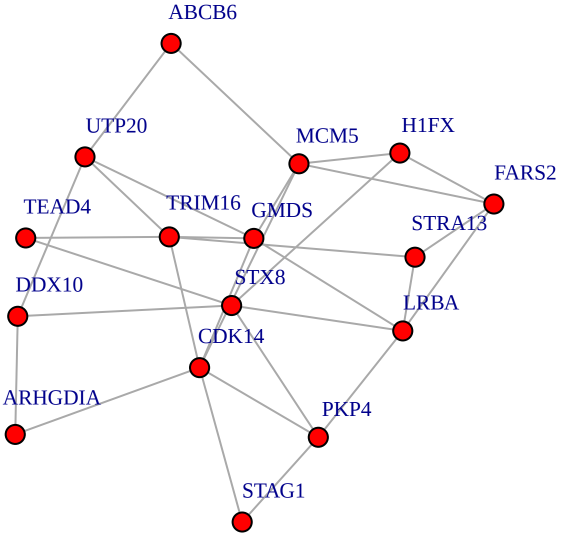

Pathway: GAJATE_RESPONSE_TO_TRABECTEDIN_UP

There are 52 genes in this pathway. This pathway was detected by GSNCA

WT p53
Hub Gene (WT): GPNMB
Weight Factor: 1.23
Hub Gene (MUT): IGFBP7
Weight Factor: 0.897

MUT p53
Hub Gene (MUT): IGFBP7
Weight Factor: 1.472
Hub Gene (WT): GPNMB
Weight Factor: 0.983

MST2 of the coexpression network for WT p53

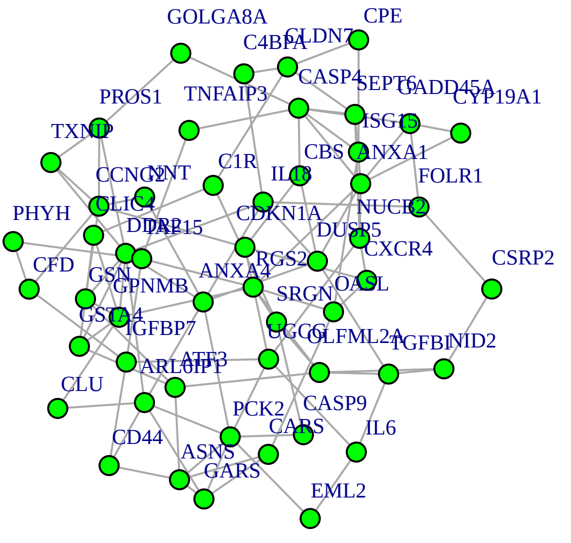

MST2 of the coexpression network for MUT p53

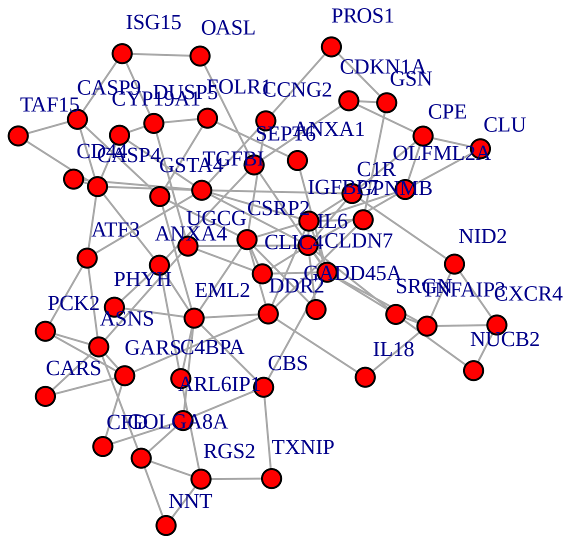

Pathway: MARTINEZ_RESPONSE_TO_TRABECTEDIN_DN

There are 172 genes in this pathway. This pathway was detected by GSNCA

WT p53
Hub Gene (WT): BUB3
Weight Factor: 1.508
Hub Gene (MUT): DCK
Weight Factor: 1.321

MUT p53
Hub Gene (MUT): DCK
Weight Factor: 1.475
Hub Gene (WT): BUB3
Weight Factor: 1.288

MST2 of the coexpression network for WT p53

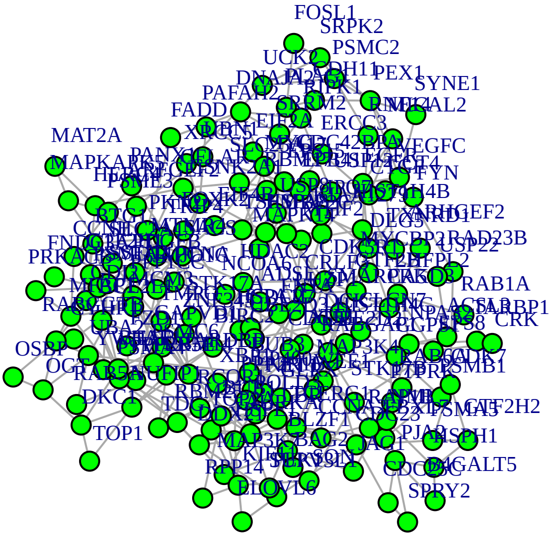

MST2 of the coexpression network for MUT p53

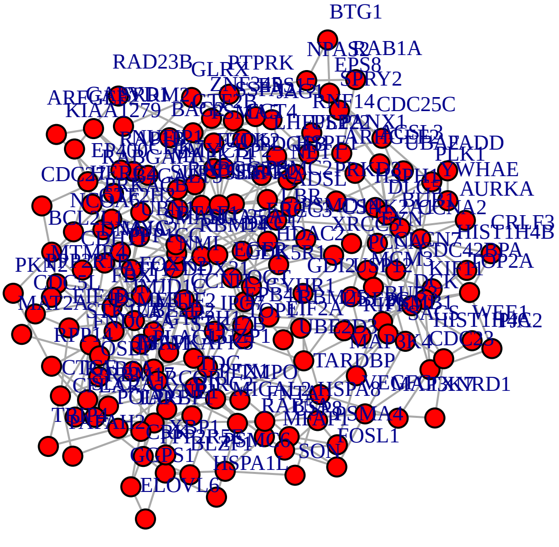

Pathway: XU_GH1_EXOGENOUS_TARGETS_DN

There are 68 genes in this pathway. This pathway was detected by GSNCA

WT p53
Hub Gene (WT): MITF
Weight Factor: 1.431
Hub Gene (MUT): NDUFS3
Weight Factor: 0.903

MUT p53
Hub Gene (MUT): NDUFS3
Weight Factor: 1.359
Hub Gene (WT): MITF
Weight Factor: 1.12

MST2 of the coexpression network for WT p53

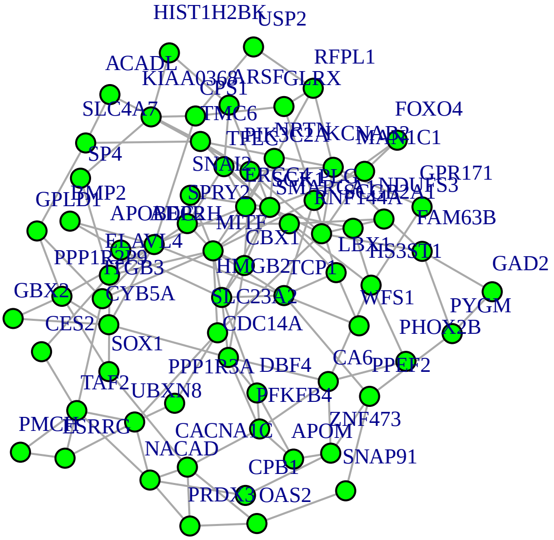

MST2 of the coexpression network for MUT p53

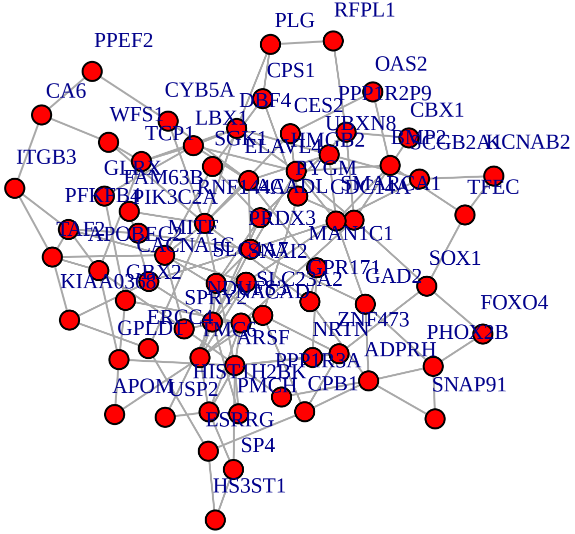

Pathway: WU_HBX_TARGETS_1_DN

There are 21 genes in this pathway. This pathway was detected by GSNCA

WT p53
Hub Gene (WT): NCOA3
Weight Factor: 1.272
Hub Gene (MUT): RYBP
Weight Factor: 0.68

MUT p53
Hub Gene (MUT): RYBP
Weight Factor: 1.381
Hub Gene (WT): NCOA3
Weight Factor: 1.184

MST2 of the coexpression network for WT p53

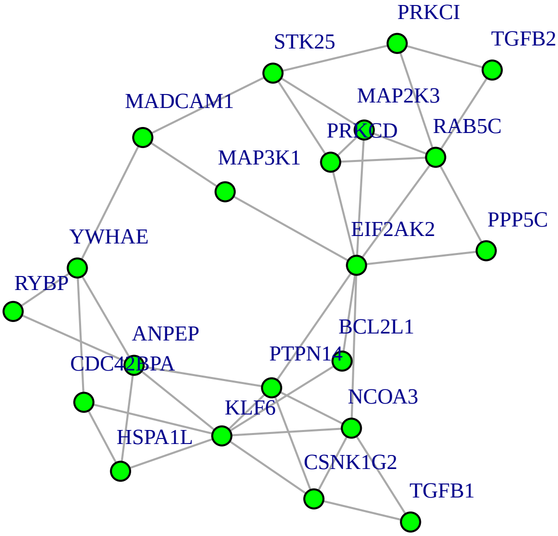

MST2 of the coexpression network for MUT p53

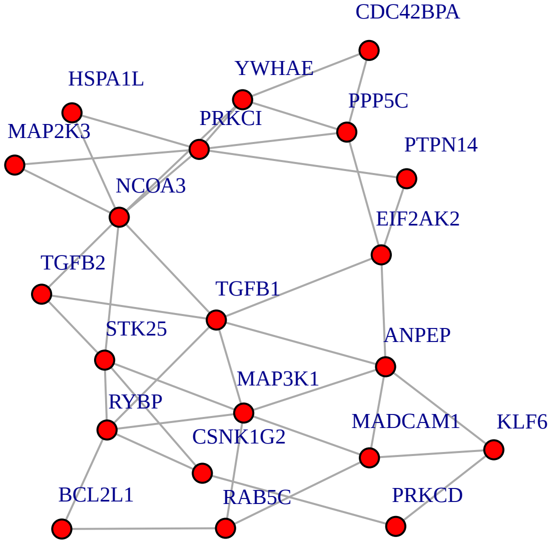

Pathway: WU_HBX_TARGETS_1_UP

There are 15 genes in this pathway. This pathway was detected by GSNCA

WT p53
Hub Gene (WT): GLRX
Weight Factor: 1.231
Hub Gene (MUT): ACP2
Weight Factor: 1.065

MUT p53
Hub Gene (MUT): ACP2
Weight Factor: 1.33
Hub Gene (WT): GLRX
Weight Factor: 0.588

MST2 of the coexpression network for WT p53

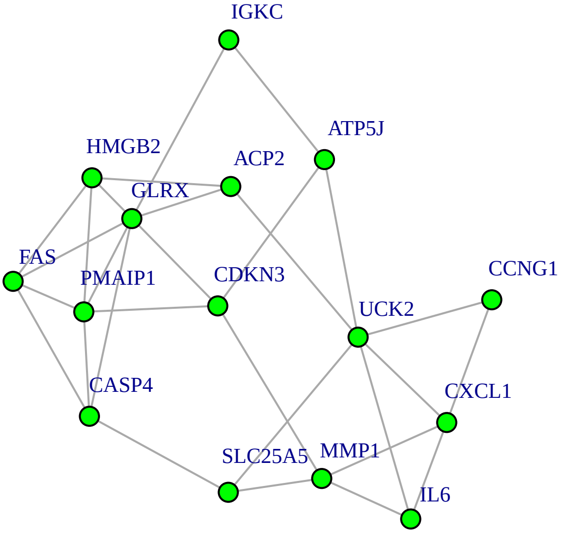

MST2 of the coexpression network for MUT p53

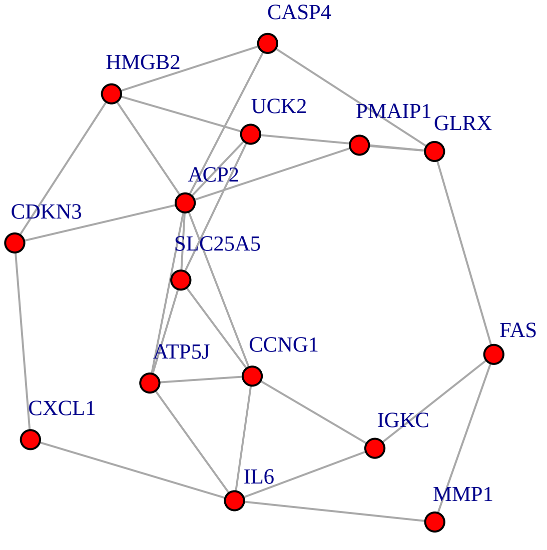

Pathway: WU_HBX_TARGETS_2_DN

There are 15 genes in this pathway. This pathway was detected by GSNCA

WT p53
Hub Gene (WT): EIF2AK2
Weight Factor: 1.403
Hub Gene (MUT): MAP3K1
Weight Factor: 0.824

MUT p53
Hub Gene (MUT): MAP3K1
Weight Factor: 1.392
Hub Gene (WT): EIF2AK2
Weight Factor: 0.855

MST2 of the coexpression network for WT p53

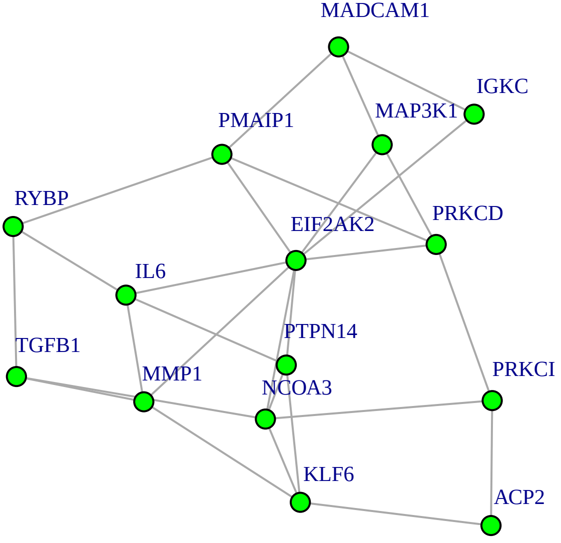

MST2 of the coexpression network for MUT p53

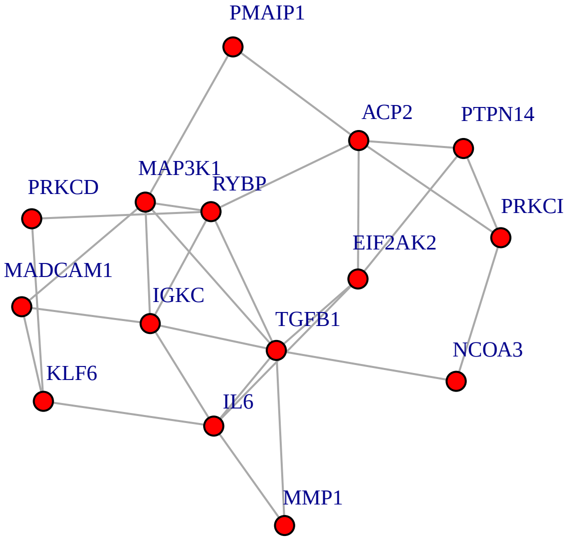

Pathway: RAMASWAMY_METASTASIS_DN

There are 60 genes in this pathway. This pathway was detected by GSNCA

WT p53
Hub Gene (WT): CPN1
Weight Factor: 1.396
Hub Gene (MUT): MYLK
Weight Factor: 1.073

MUT p53
Hub Gene (MUT): MYLK
Weight Factor: 1.639
Hub Gene (WT): CPN1
Weight Factor: 0.796

MST2 of the coexpression network for WT p53

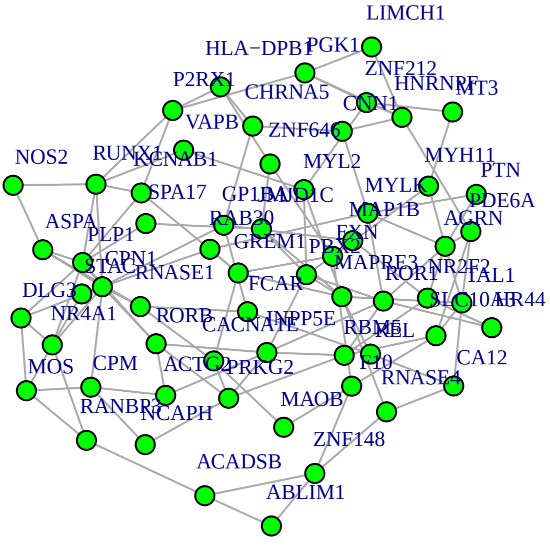

MST2 of the coexpression network for MUT p53

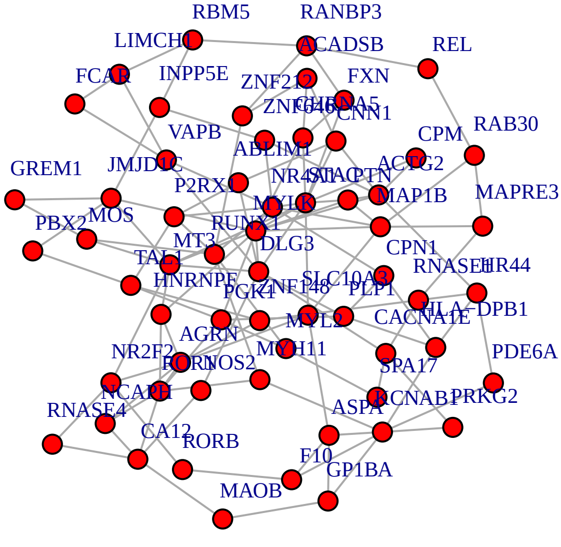

Pathway: WEIGEL_OXIDATIVE_STRESS_BY_HNE_AND_TBH

There are 58 genes in this pathway. This pathway was detected by GSNCA

WT p53
Hub Gene (WT): S100A1
Weight Factor: 1.314
Hub Gene (MUT): IL6ST
Weight Factor: 1.113

MUT p53
Hub Gene (MUT): IL6ST
Weight Factor: 1.438
Hub Gene (WT): S100A1
Weight Factor: 0.79

MST2 of the coexpression network for WT p53

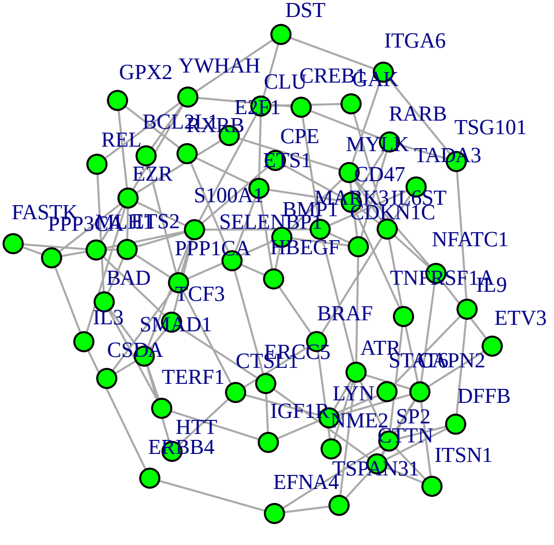

MST2 of the coexpression network for MUT p53

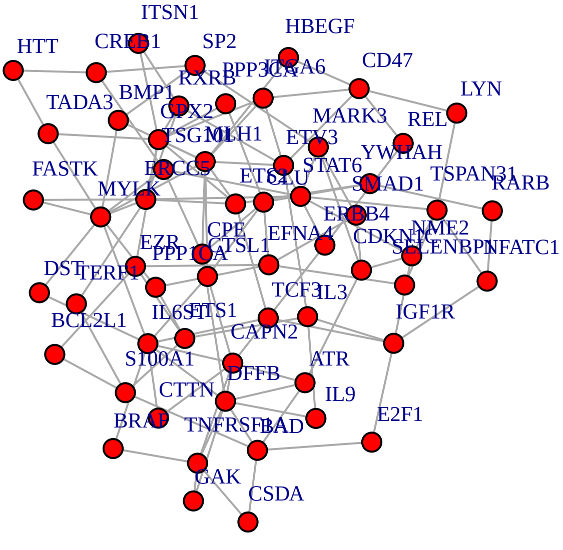

Pathway: PAL_PRMT5_TARGETS_DN

There are 25 genes in this pathway. This pathway was detected by GSNCA

WT p53
Hub Gene (WT): GATA4
Weight Factor: 1.307
Hub Gene (MUT): LCN2
Weight Factor: 0.939

MUT p53
Hub Gene (MUT): LCN2
Weight Factor: 1.44
Hub Gene (WT): GATA4
Weight Factor: 0.724

MST2 of the coexpression network for WT p53

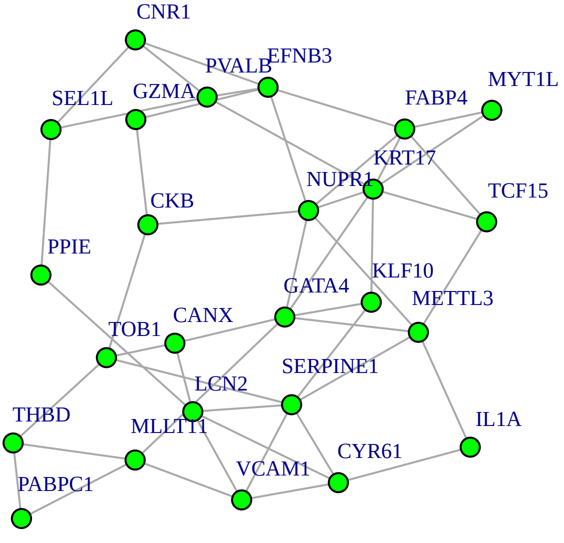

MST2 of the coexpression network for MUT p53

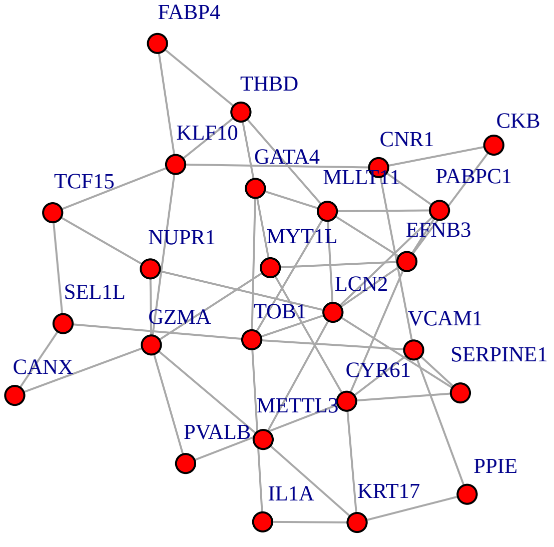

Pathway: SATO_SILENCED_EPIGENETICALLY_IN_PANCREATIC_CANCER

There are 38 genes in this pathway. This pathway was detected by GSNCA

WT p53
Hub Gene (WT): AURKC
Weight Factor: 1.33
Hub Gene (MUT): S100P
Weight Factor: 1.076

MUT p53
Hub Gene (MUT): S100P
Weight Factor: 1.423
Hub Gene (WT): AURKC
Weight Factor: 0.749

MST2 of the coexpression network for WT p53

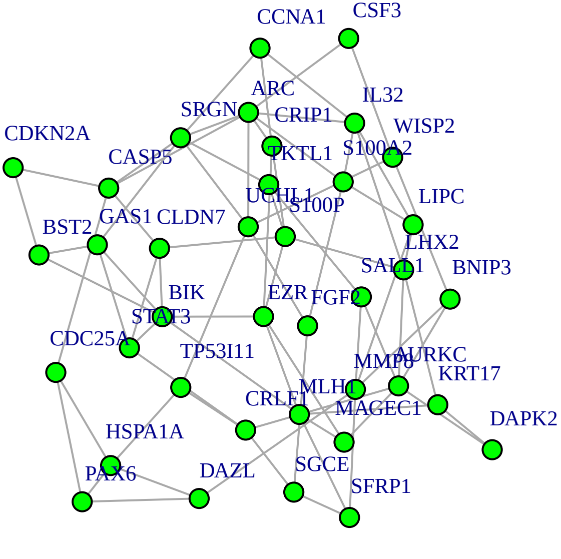

MST2 of the coexpression network for MUT p53

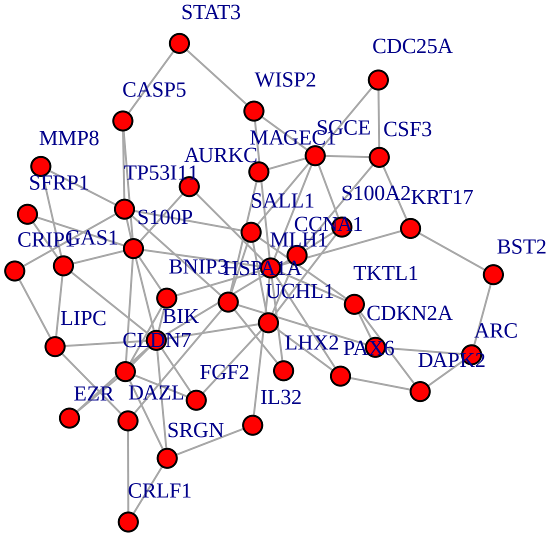

Pathway: MURAKAMI_UV_RESPONSE_6HR_DN

There are 20 genes in this pathway. This pathway was detected by GSNCA

WT p53
Hub Gene (WT): KRT13
Weight Factor: 1.274
Hub Gene (MUT): IL6
Weight Factor: 0.892

MUT p53
Hub Gene (MUT): IL6
Weight Factor: 1.436
Hub Gene (WT): KRT13
Weight Factor: 0.945

MST2 of the coexpression network for WT p53

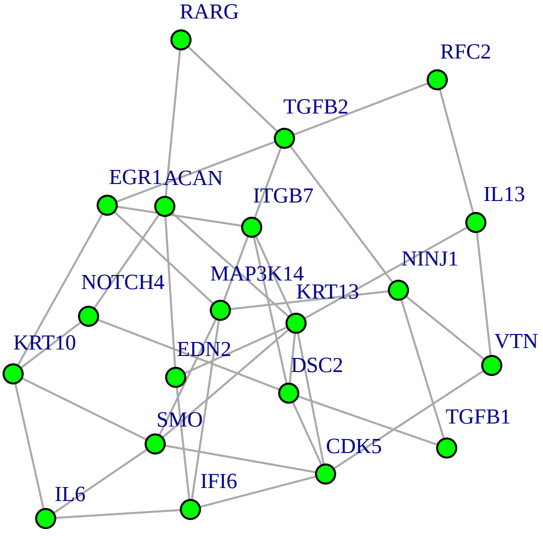

MST2 of the coexpression network for MUT p53

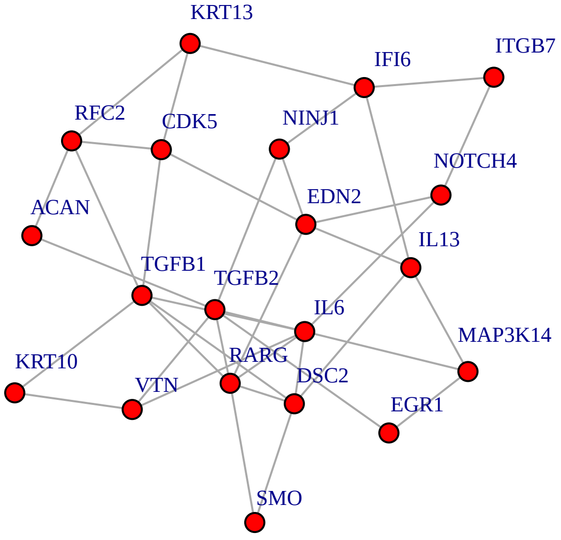

Pathway: GENTILE_UV_LOW_DOSE_DN

There are 18 genes in this pathway. This pathway was detected by GSNCA

WT p53
Hub Gene (WT): GDF15
Weight Factor: 1.422
Hub Gene (MUT): FDXR
Weight Factor: 1.05

MUT p53
Hub Gene (MUT): FDXR
Weight Factor: 1.357
Hub Gene (WT): GDF15
Weight Factor: 0.736

MST2 of the coexpression network for WT p53

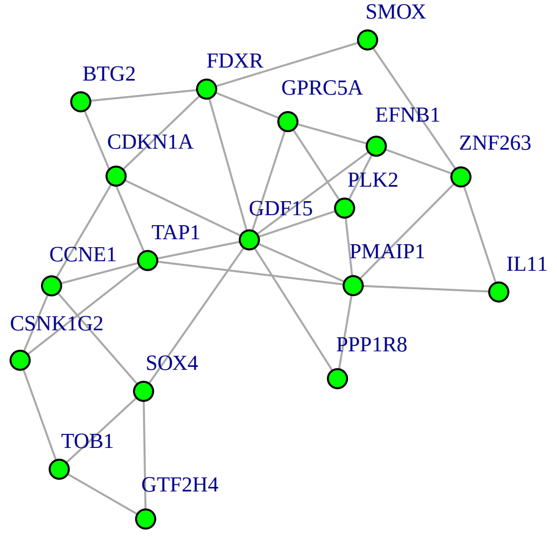

MST2 of the coexpression network for MUT p53

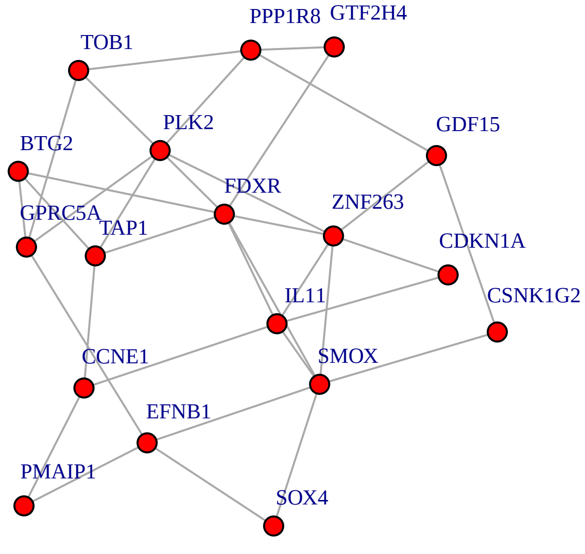

Pathway: GENTILE_UV_LOW_DOSE_UP

There are 18 genes in this pathway. This pathway was detected by GSNCA

WT p53
Hub Gene (WT): GDF15
Weight Factor: 1.422
Hub Gene (MUT): FDXR
Weight Factor: 1.05

MUT p53
Hub Gene (MUT): FDXR
Weight Factor: 1.357
Hub Gene (WT): GDF15
Weight Factor: 0.736

MST2 of the coexpression network for WT p53

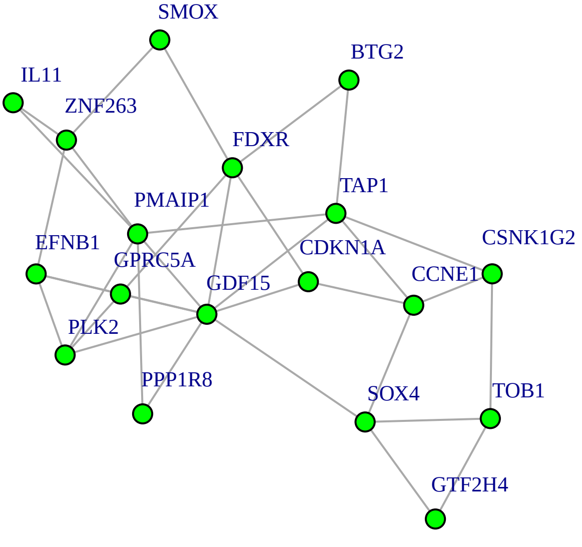

MST2 of the coexpression network for MUT p53

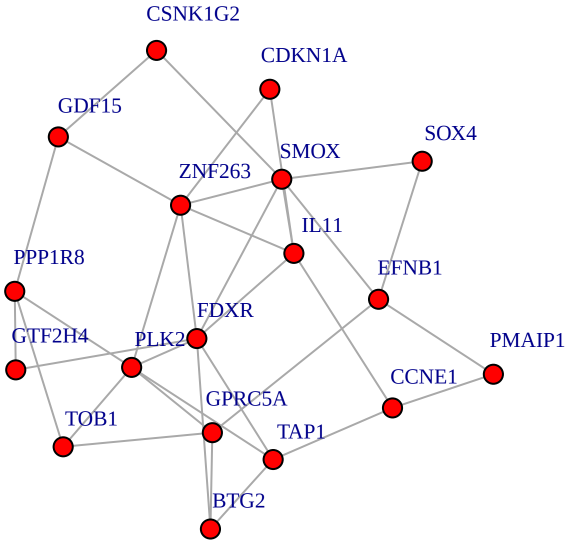

Pathway: KAAB_HEART_ATRIUM_VS_VENTRICLE_DN

There are 256 genes in this pathway. This pathway was detected by GSNCA

WT p53
Hub Gene (WT): LARP4B
Weight Factor: 1.325
Hub Gene (MUT): NDUFS3
Weight Factor: 1.163

MUT p53
Hub Gene (MUT): NDUFS3
Weight Factor: 1.529
Hub Gene (WT): LARP4B
Weight Factor: 1.098

MST2 of the coexpression network for WT p53

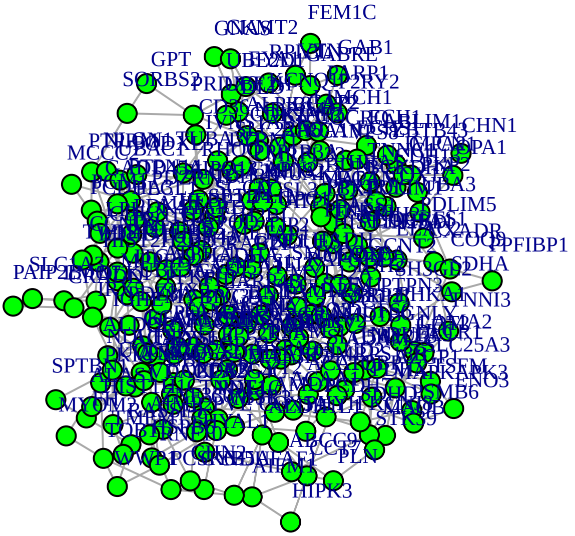

MST2 of the coexpression network for MUT p53

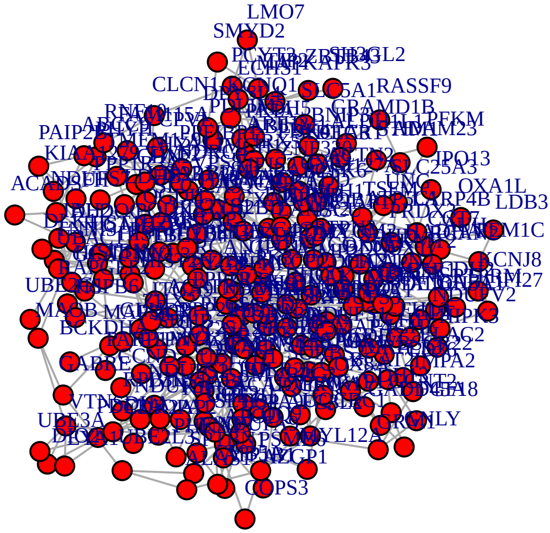

Pathway: MOOTHA_MITOCHONDRIA

There are 329 genes in this pathway. This pathway was detected by GSNCA

WT p53
Hub Gene (WT): SDHB
Weight Factor: 1.262
Hub Gene (MUT): NDUFAB1
Weight Factor: 1.167

MUT p53
Hub Gene (MUT): NDUFAB1
Weight Factor: 1.663
Hub Gene (WT): SDHB
Weight Factor: 1.251

MST2 of the coexpression network for WT p53

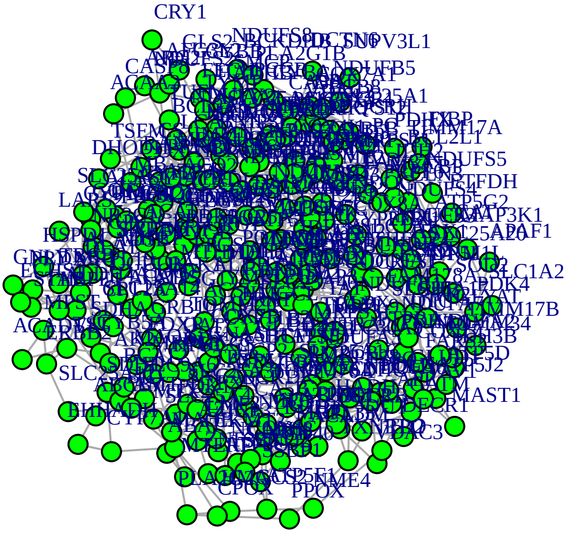

MST2 of the coexpression network for MUT p53

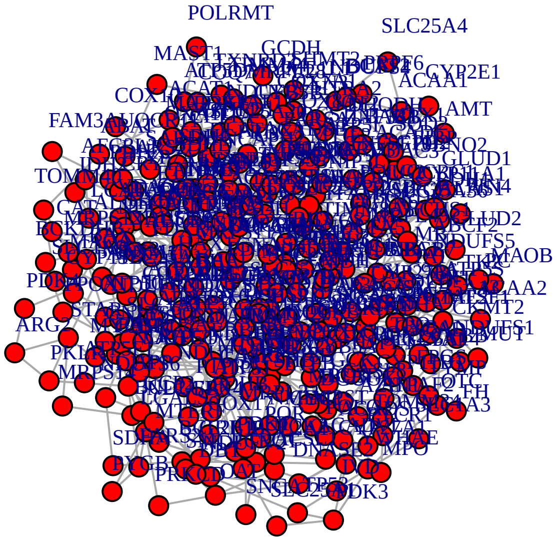

Pathway: KEGG_CITRATE_CYCLE_TCA_CYCLE

There are 28 genes in this pathway. This pathway was detected by GSNCA

WT p53
Hub Gene (WT): ACO2
Weight Factor: 1.431
Hub Gene (MUT): SUCLA2
Weight Factor: 0.77

MUT p53
Hub Gene (MUT): SUCLA2
Weight Factor: 1.366
Hub Gene (WT): ACO2
Weight Factor: 0.981

MST2 of the coexpression network for WT p53

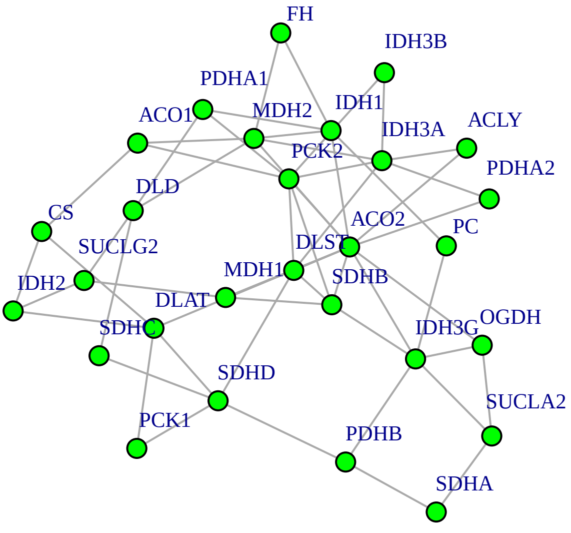

MST2 of the coexpression network for MUT p53

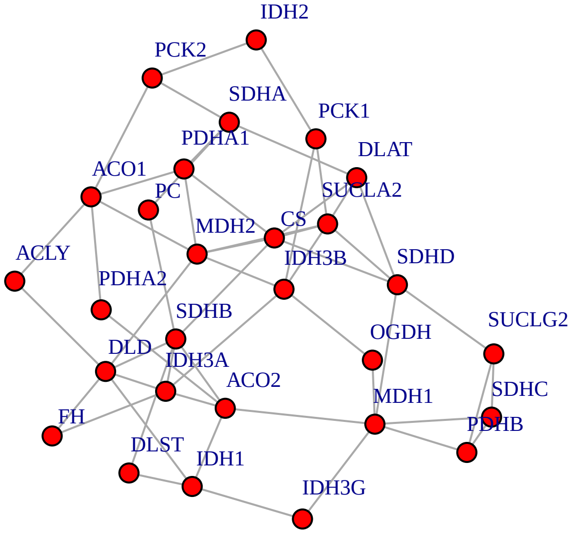

Pathway: KEGG_MAPK_SIGNALING_PATHWAY

There are 220 genes in this pathway. This pathway was detected by GSNCA

WT p53
Hub Gene (WT): GADD45B
Weight Factor: 1.385
Hub Gene (MUT): FLNA
Weight Factor: 1.107

MUT p53
Hub Gene (MUT): FLNA
Weight Factor: 1.522
Hub Gene (WT): GADD45B
Weight Factor: 1.1

MST2 of the coexpression network for WT p53

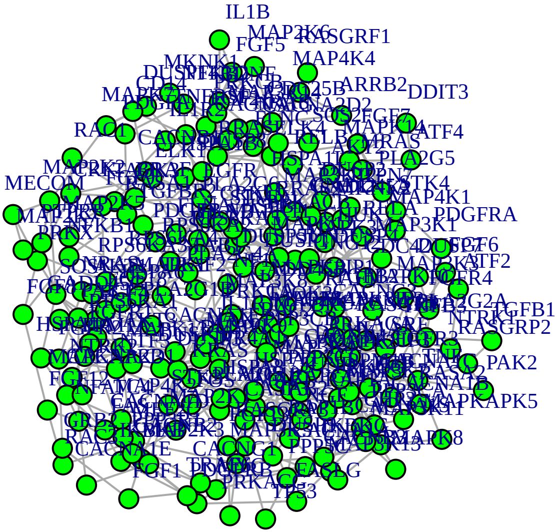

MST2 of the coexpression network for MUT p53

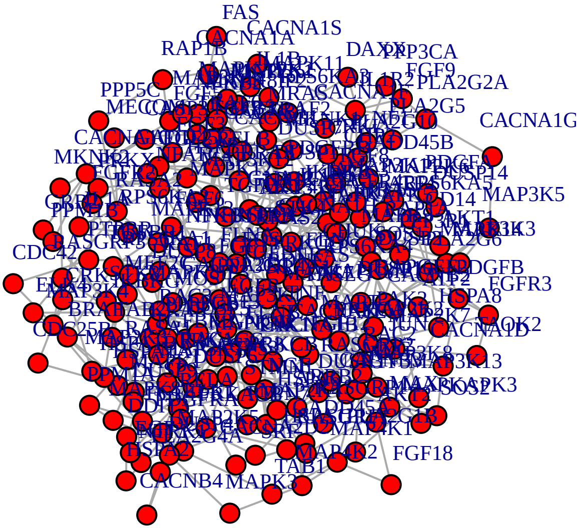

Pathway: KEGG_PEROXISOME

There are 55 genes in this pathway. This pathway was detected by GSNCA

WT p53
Hub Gene (WT): MVK
Weight Factor: 1.382
Hub Gene (MUT): ACOX1
Weight Factor: 0.953

MUT p53
Hub Gene (MUT): ACOX1
Weight Factor: 1.471
Hub Gene (WT): MVK
Weight Factor: 1.016

MST2 of the coexpression network for WT p53

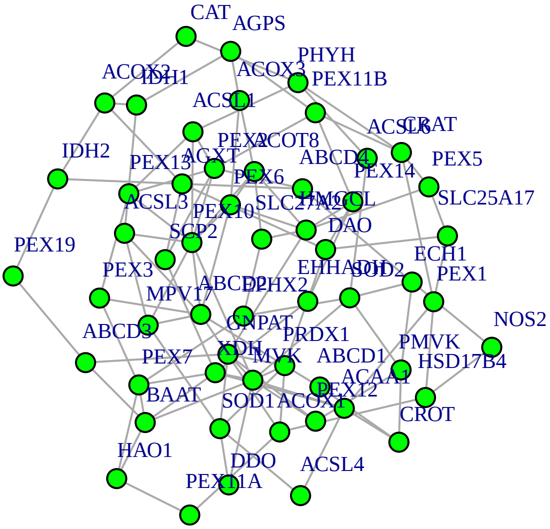

MST2 of the coexpression network for MUT p53

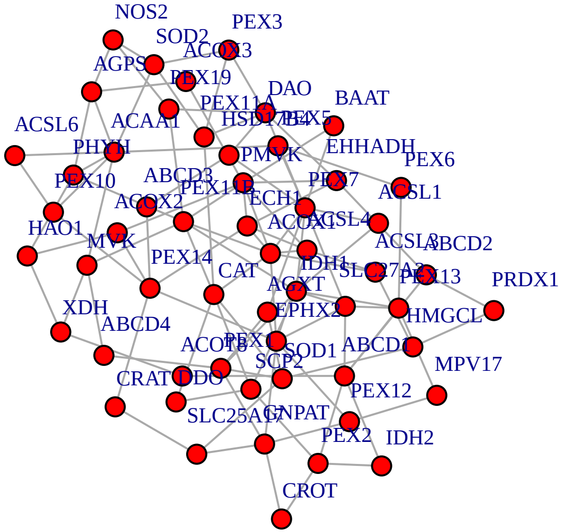

Pathway: KEGG_RENAL_CELL_CARCINOMA

There are 63 genes in this pathway. This pathway was detected by GSNCA

WT p53
Hub Gene (WT): MAPK1
Weight Factor: 1.254
Hub Gene (MUT): AKT3
Weight Factor: 0.992

MUT p53
Hub Gene (MUT): AKT3
Weight Factor: 1.375
Hub Gene (WT): MAPK1
Weight Factor: 0.846

MST2 of the coexpression network for WT p53

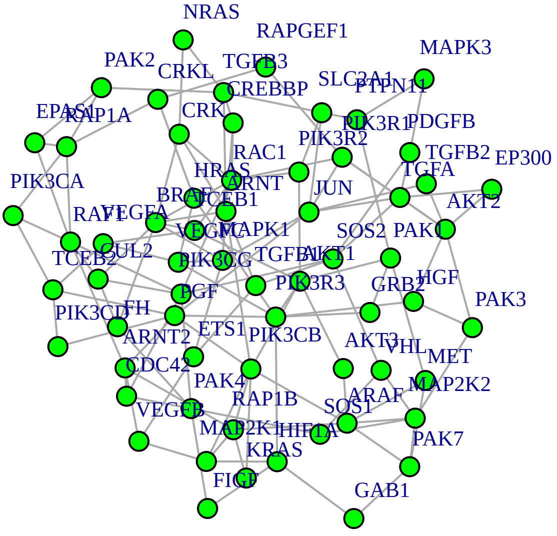

MST2 of the coexpression network for MUT p53

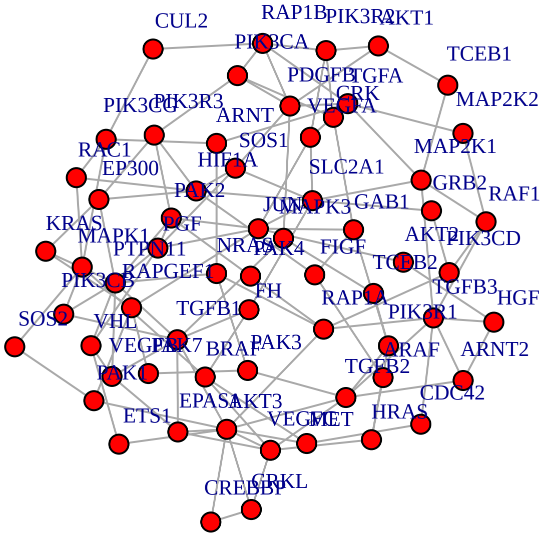

Pathway: BIOCARTA_CERAMIDE_PATHWAY

There are 22 genes in this pathway. This pathway was detected by GSNCA

WT p53
Hub Gene (WT): MAP2K4
Weight Factor: 1.335
Hub Gene (MUT): BAD
Weight Factor: 0.937

MUT p53
Hub Gene (MUT): BAD
Weight Factor: 1.394
Hub Gene (WT): MAP2K4
Weight Factor: 0.686

MST2 of the coexpression network for WT p53

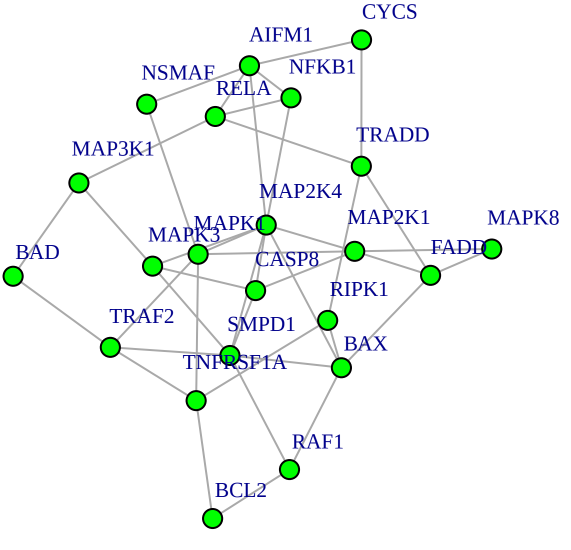

MST2 of the coexpression network for MUT p53

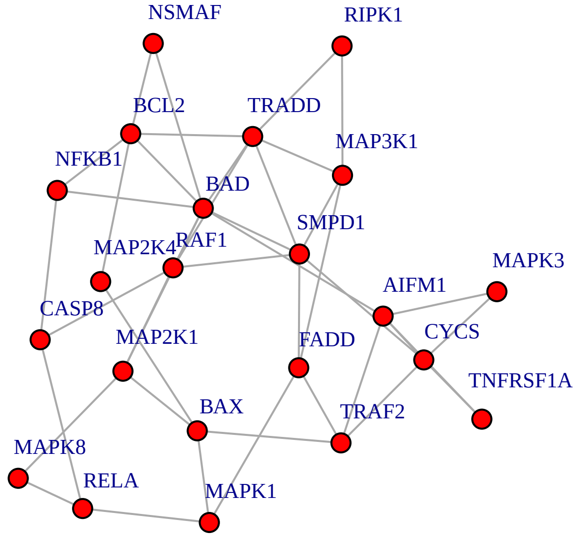

Pathway: BIOCARTA_CTCF_PATHWAY

There are 23 genes in this pathway. This pathway was detected by GSNCA

WT p53
Hub Gene (WT): MTOR
Weight Factor: 1.442
Hub Gene (MUT): TGFB2
Weight Factor: 1.03

MUT p53
Hub Gene (MUT): TGFB2
Weight Factor: 1.324
Hub Gene (WT): MTOR
Weight Factor: 0.959

MST2 of the coexpression network for WT p53

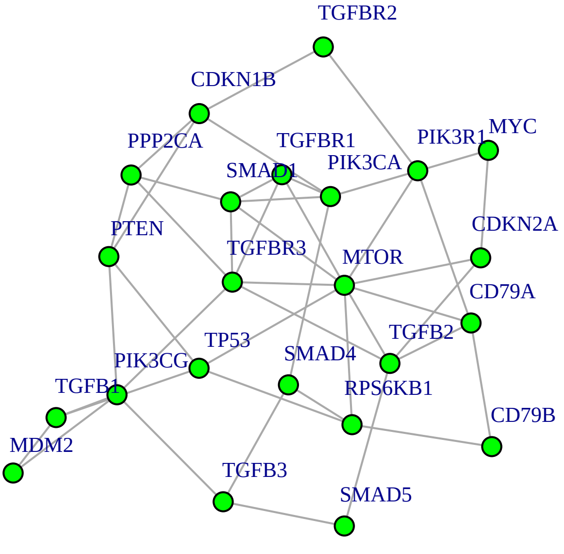

MST2 of the coexpression network for MUT p53

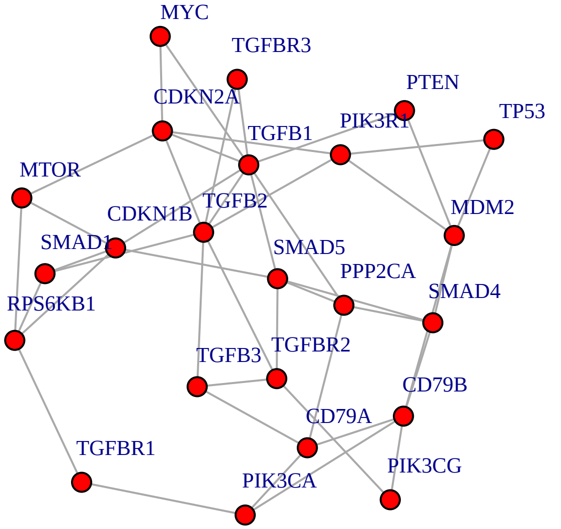

Pathway: BIOCARTA_GLEEVEC_PATHWAY

There are 23 genes in this pathway. This pathway was detected by GSNCA

WT p53
Hub Gene (WT): MAP2K4
Weight Factor: 1.238
Hub Gene (MUT): BAD
Weight Factor: 0.852

MUT p53
Hub Gene (MUT): BAD
Weight Factor: 1.275
Hub Gene (WT): MAP2K4
Weight Factor: 0.904

MST2 of the coexpression network for WT p53

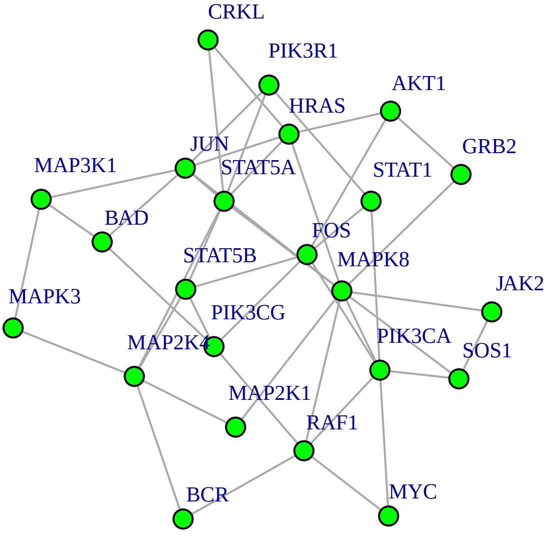

MST2 of the coexpression network for MUT p53

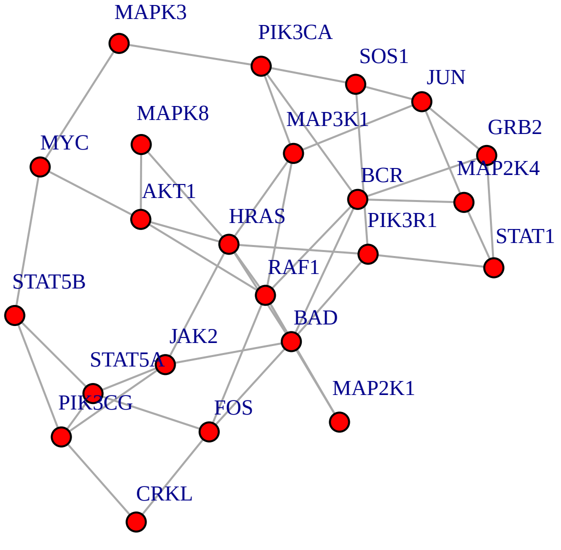

Pathway: BIOCARTA_MITOCHONDRIA_PATHWAY

There are 20 genes in this pathway. This pathway was detected by GSNCA

WT p53
Hub Gene (WT): CASP7
Weight Factor: 1.296
Hub Gene (MUT): DFFB
Weight Factor: 0.65

MUT p53
Hub Gene (MUT): DFFB
Weight Factor: 1.389
Hub Gene (WT): CASP7
Weight Factor: 0.874

MST2 of the coexpression network for WT p53

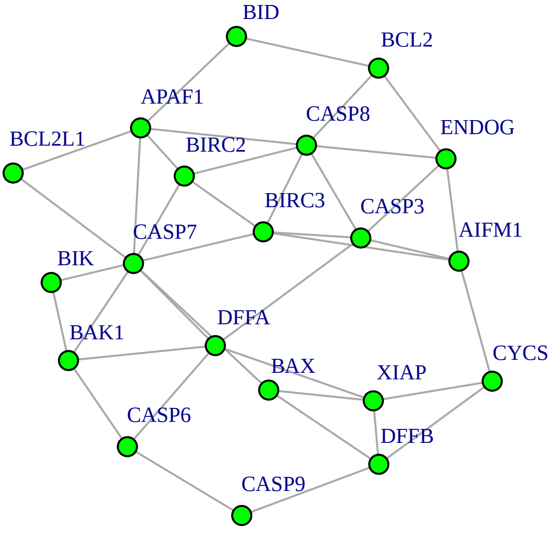

MST2 of the coexpression network for MUT p53

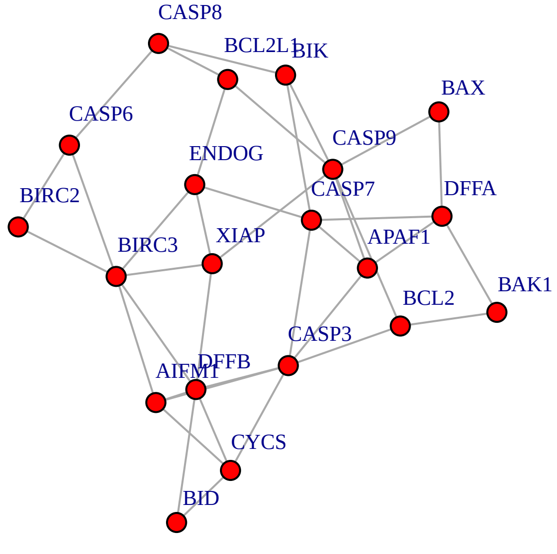

Pathway: BIOCARTA_TEL_PATHWAY

There are 18 genes in this pathway. This pathway was detected by GSNCA

WT p53	
Hub Gene (WT):	TERF1
Weight Factor:	1.438
Hub Gene (MUT):	XRCC6
Weight Factor:	0.651

MUT p53	
Hub Gene (MUT):	XRCC6
Weight Factor:	1.278
Hub Gene (WT):	TERF1
Weight Factor:	0.535

MST2 of the coexpression network for WT p53

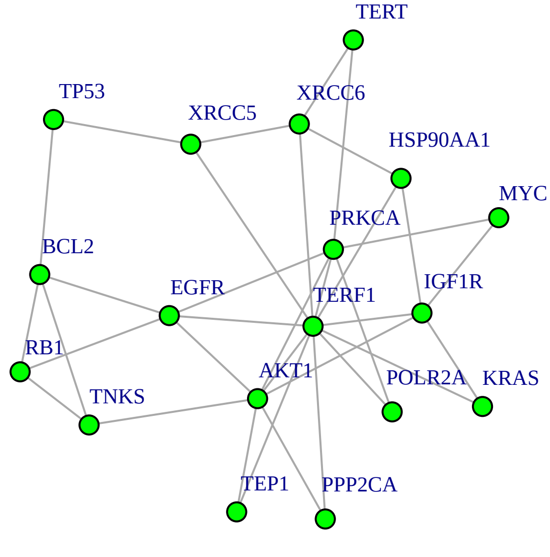

MST2 of the coexpression network for MUT p53

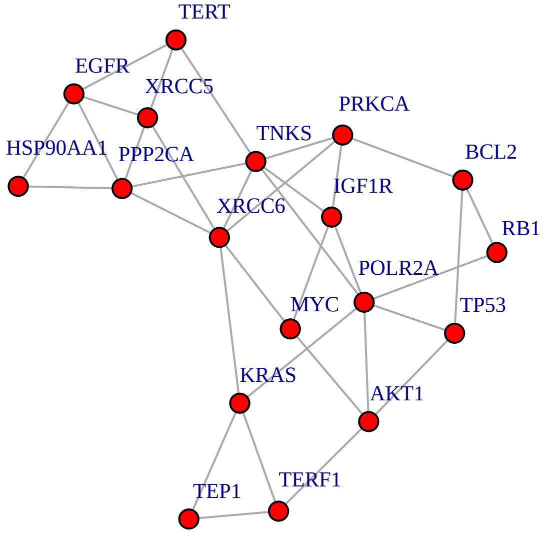

Pathway: REACTOME_CELL_CELL_ADHESION_SYSTEMS

There are 37 genes in this pathway. This pathway was detected by GSNCA

WT p53
Hub Gene (WT): CLDN3
Weight Factor: 1.322
Hub Gene (MUT): CLDN3
Weight Factor: 1.322

MUT p53
Hub Gene (MUT): CLDN3
Weight Factor: 1.574
Hub Gene (WT): CLDN3
Weight Factor: 1.574

MST2 of the coexpression network for WT p53

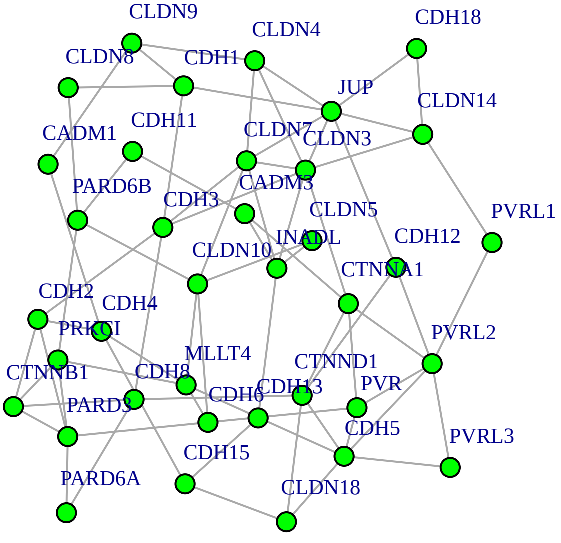

MST2 of the coexpression network for MUT p53

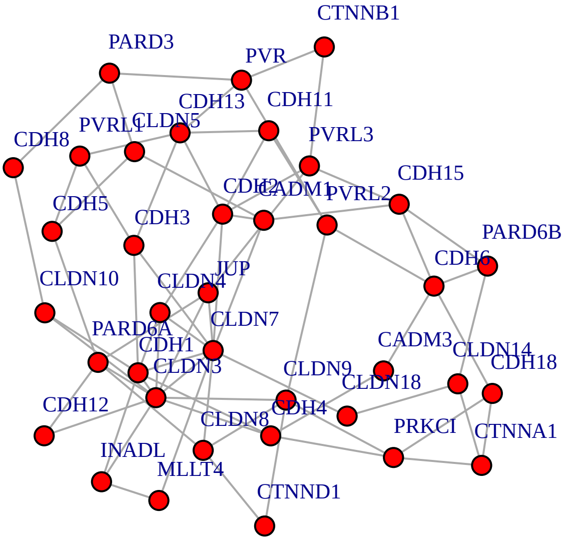

Pathway: REACTOME_CYCLIN_A1_ASSOCIATED_EVENTS_DURING_G2_M_TRANSITION

There are 15 genes in this pathway. This pathway was detected by GSNCA

WT p53
Hub Gene (WT): CDK1
Weight Factor: 1.278
Hub Gene (MUT): CCNB1
Weight Factor: 1.235

MUT p53
Hub Gene (MUT): CCNB1
Weight Factor: 1.271
Hub Gene (WT): CDK1
Weight Factor: 1.209

MST2 of the coexpression network for WT p53

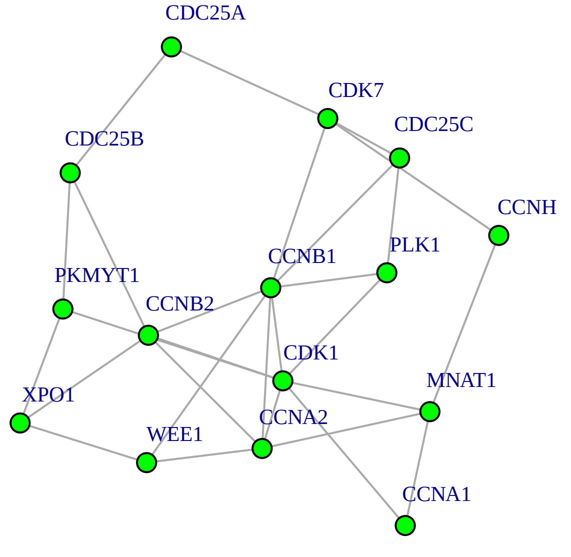

MST2 of the coexpression network for MUT p53

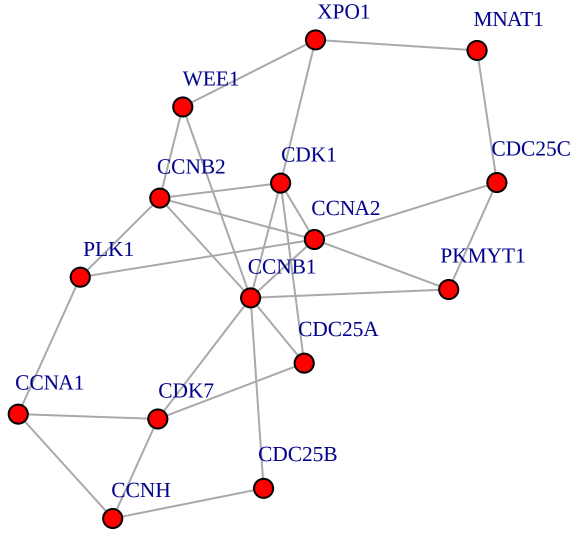

Pathway: REACTOME_INTRINSIC_PATHWAY_FOR_APOPTOSIS

There are 26 genes in this pathway. This pathway was detected by GSNCA

WT p53
Hub Gene (WT): CASP7
Weight Factor: 1.417
Hub Gene (MUT): CYCS
Weight Factor: 0.748

MUT p53
Hub Gene (MUT): CYCS
Weight Factor: 1.346
Hub Gene (WT): CASP7
Weight Factor: 0.822

MST2 of the coexpression network for WT p53

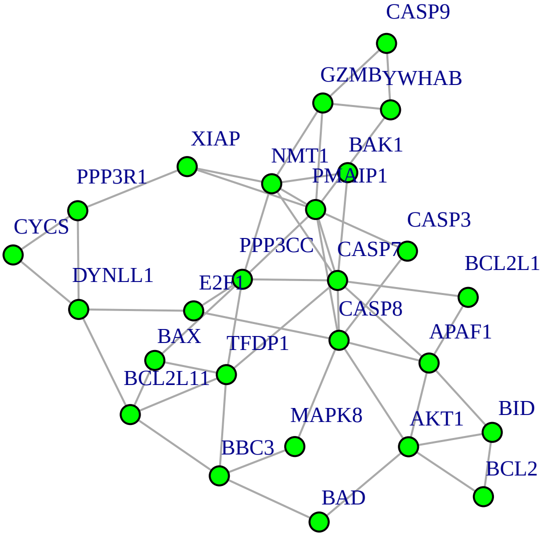

MST2 of the coexpression network for MUT p53

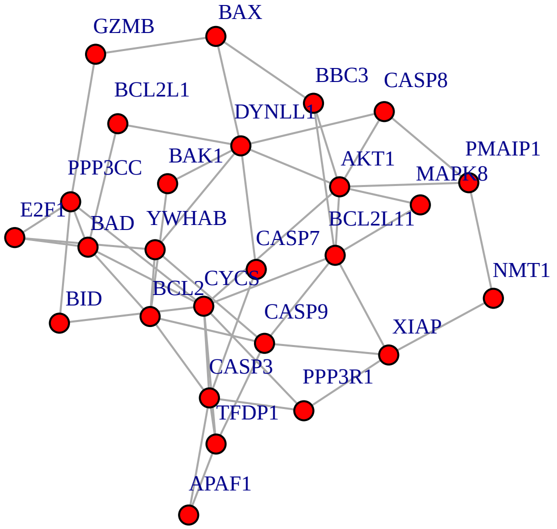

Pathway: REACTOME_LYSOSOME_VESICLE_BIOGENESIS

There are 19 genes in this pathway. This pathway was detected by GSNCA

WT p53
Hub Gene (WT): GNS
Weight Factor: 1.412
Hub Gene (MUT): VAMP7
Weight Factor: 0.9

MUT p53
Hub Gene (MUT): VAMP7
Weight Factor: 1.27
Hub Gene (WT): GNS
Weight Factor: 0.935

MST2 of the coexpression network for WT p53

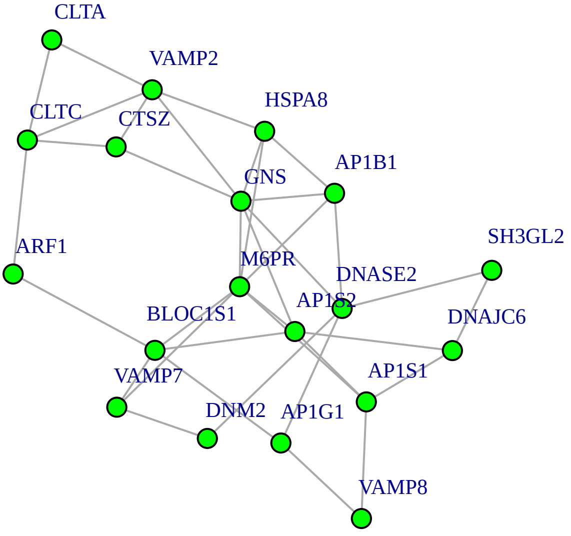

MST2 of the coexpression network for MUT p53

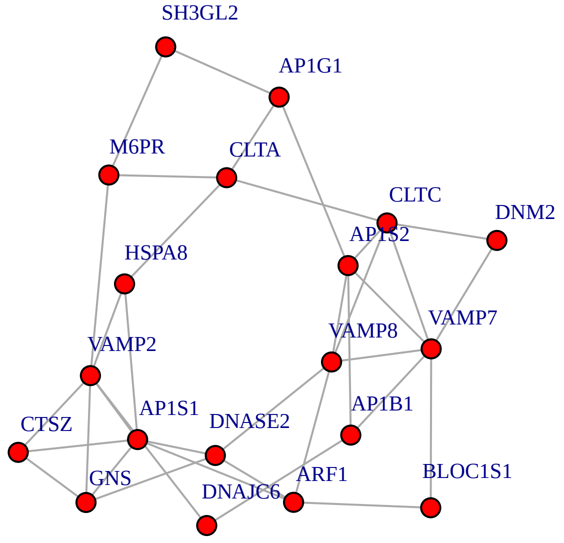

Pathway: REACTOME_MEMBRANE_TRAFFICKING

There are 58 genes in this pathway. This pathway was detected by GSNCA

WT p53
Hub Gene (WT): SEC24B
Weight Factor: 1.329
Hub Gene (MUT): COPA
Weight Factor: 1.101

MUT p53
Hub Gene (MUT): COPA
Weight Factor: 1.308
Hub Gene (WT): SEC24B
Weight Factor: 1.045

MST2 of the coexpression network for WT p53

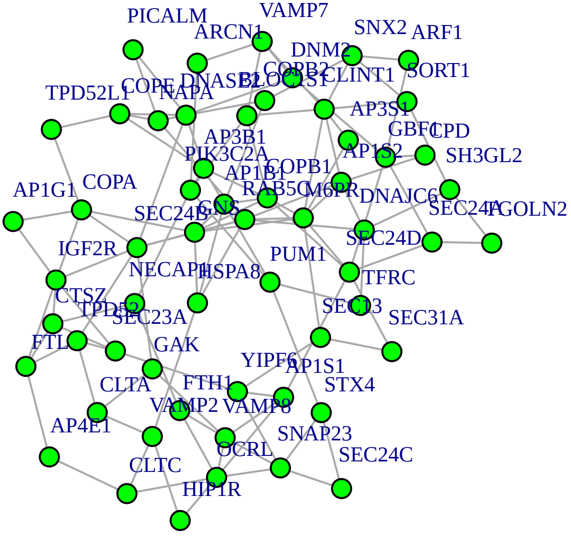

MST2 of the coexpression network for MUT p53

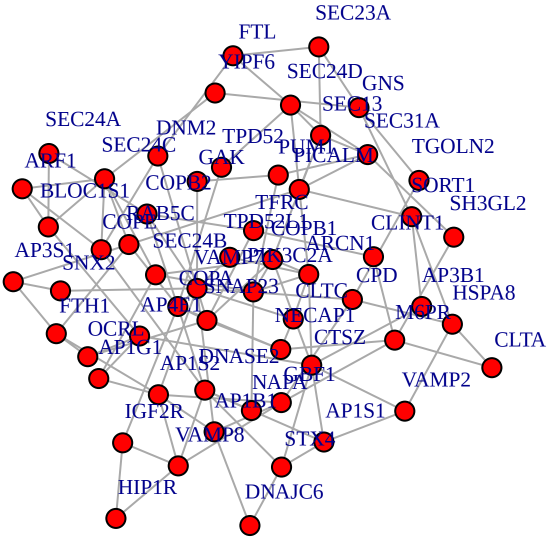

Pathway: REACTOME_MICRORNA_BIOGENESIS

There are 15 genes in this pathway. This pathway was detected by GSNCA

WT p53
Hub Gene (WT): DICER1
Weight Factor: 1.276
Hub Gene (MUT): RAN
Weight Factor: 0.602

MUT p53
Hub Gene (MUT): RAN
Weight Factor: 1.399
Hub Gene (WT): DICER1
Weight Factor: 0.458

MST2 of the coexpression network for WT p53

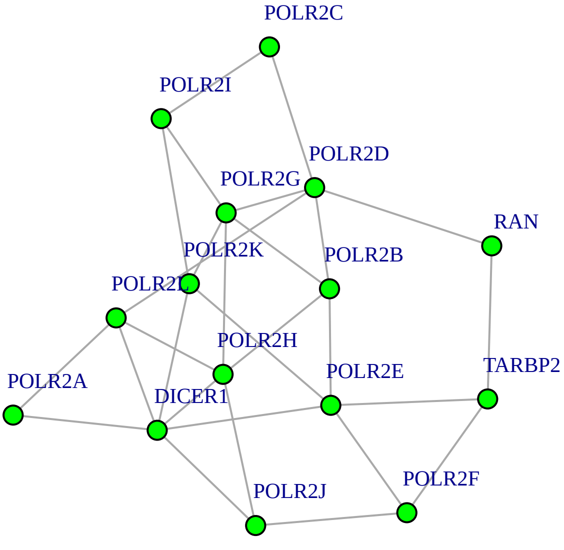

MST2 of the coexpression network for MUT p53

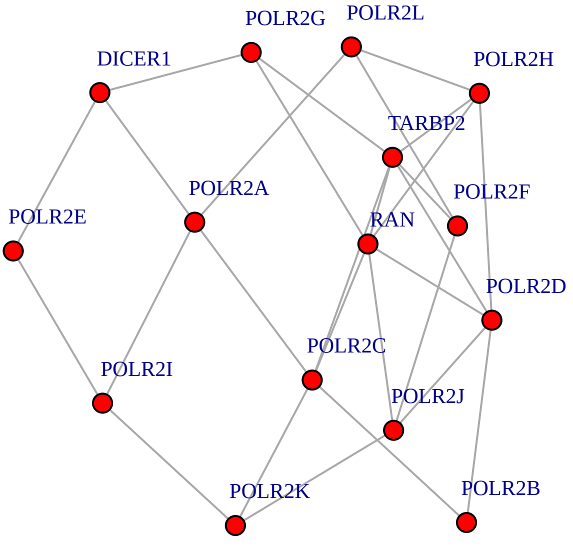

Pathway: REACTOME_PHASE_II_CONJUGATION

There are 35 genes in this pathway. This pathway was detected by GSNCA

WT p53
Hub Gene (WT): NAT1
Weight Factor: 1.26
Hub Gene (MUT): SULT1A1
Weight Factor: 0.862

MUT p53
Hub Gene (MUT): SULT1A1
Weight Factor: 1.337
Hub Gene (WT): NAT1
Weight Factor: 1.202

MST2 of the coexpression network for WT p53

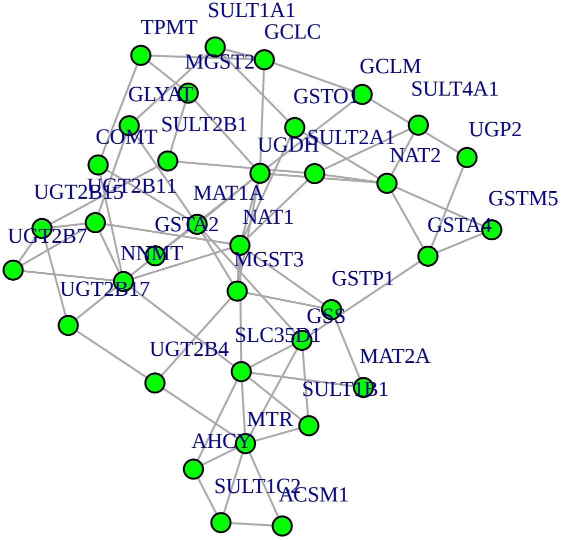

MST2 of the coexpression network for MUT p53

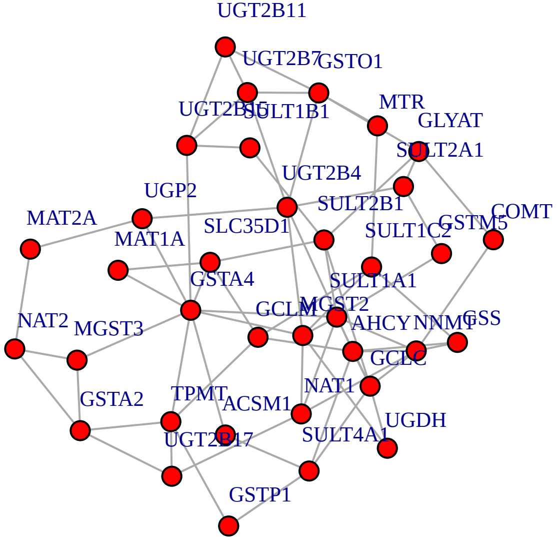

Pathway: REACTOME_TOLL_LIKE_RECEPTOR_3_CASCADE

There are 56 genes in this pathway. This pathway was detected by GSNCA

WT p53		MUT p53	
**Hub Gene (WT):**	**MAPK9**	**Hub Gene (MUT):**	**DUSP6**
**Weight Factor:**	**1.414**	**Weight Factor:**	**1.388**
**Hub Gene (MUT):**	**DUSP6**	**Hub Gene (WT):**	**MAPK9**
**Weight Factor:**	**0.942**	**Weight Factor:**	**1.078**

MST2 of the coexpression network for WT p53

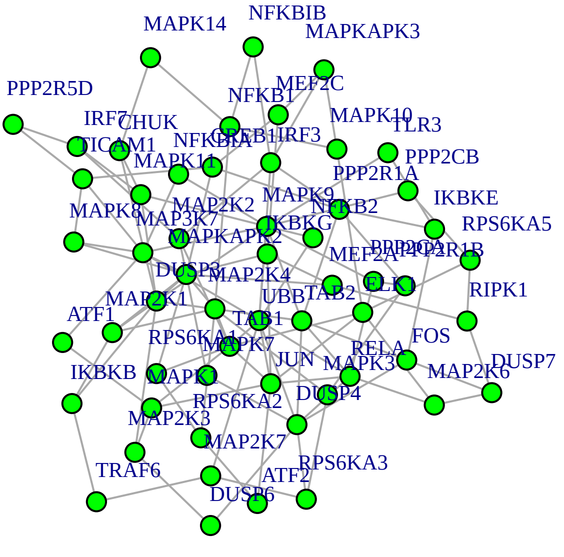

MST2 of the coexpression network for MUT p53

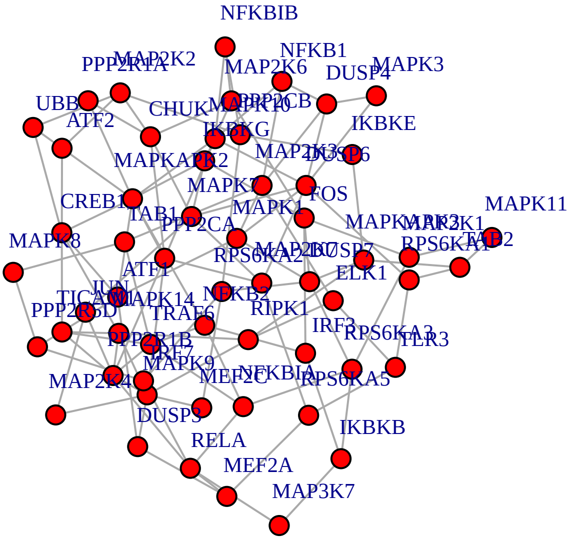

REACTOME_TRAF6_MEDIATED_INDUCTION_OF_THE_ANTIVIRAL_CYTOKINE_IFN_ALPHA_BETA_

There are 52 genes in this pathway. This pathway was detected by GSNCA

WT p53
Hub Gene (WT): MAPK9
Weight Factor: 1.443
Hub Gene (MUT): DUSP6
Weight Factor: 0.914

MUT p53
Hub Gene (MUT): DUSP6
Weight Factor: 1.39
Hub Gene (WT): MAPK9
Weight Factor: 1.06

MST2 of the coexpression network for WT p53

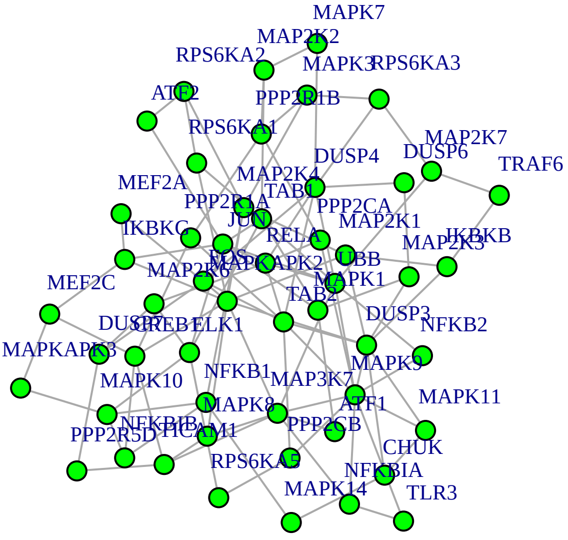

MST2 of the coexpression network for MUT p53

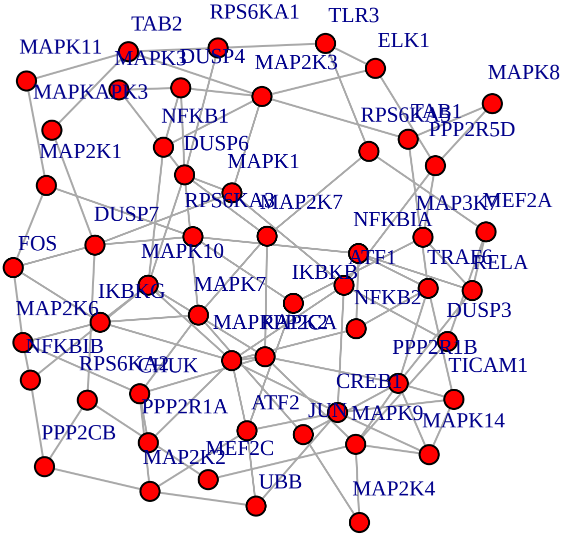

Pathway: REACTOME_UNFOLDED_PROTEIN_RESPONSE

There are 17 genes in this pathway. This pathway was detected by GSNCA

WT p53
Hub Gene (WT): NFYA
Weight Factor: 1.238
Hub Gene (MUT): HERPUD1
Weight Factor: 0.69

MUT p53
Hub Gene (MUT): HERPUD1
Weight Factor: 1.362
Hub Gene (WT): NFYA
Weight Factor: 0.761

MST2 of the coexpression network for WT p53

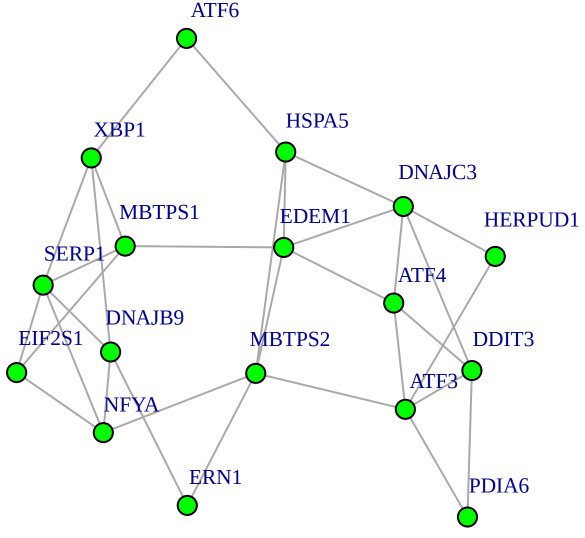

MST2 of the coexpression network for MUT p53

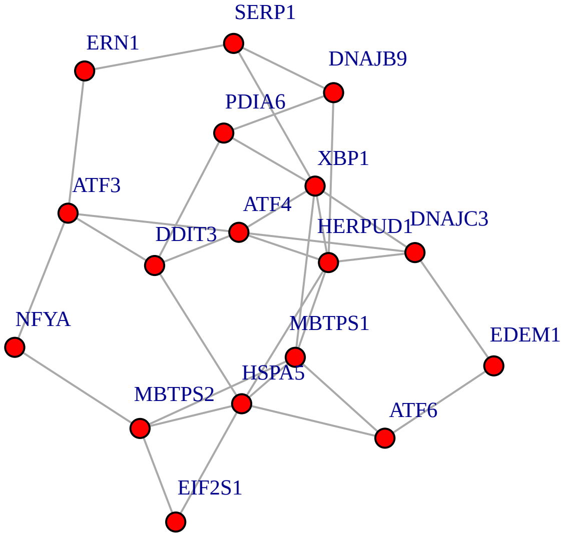

Pathway: REACTOME_MAP_KINASES_ACTIVATION_IN_TLR_CASCADE

There are 43 genes in this pathway. This pathway was detected by GSNCA

WT p53
Hub Gene (WT): MAPK9
Weight Factor: 1.405
Hub Gene (MUT): DUSP6
Weight Factor: 0.933

MUT p53
Hub Gene (MUT): DUSP6
Weight Factor: 1.431
Hub Gene (WT): MAPK9
Weight Factor: 1.139

MST2 of the coexpression network for WT p53

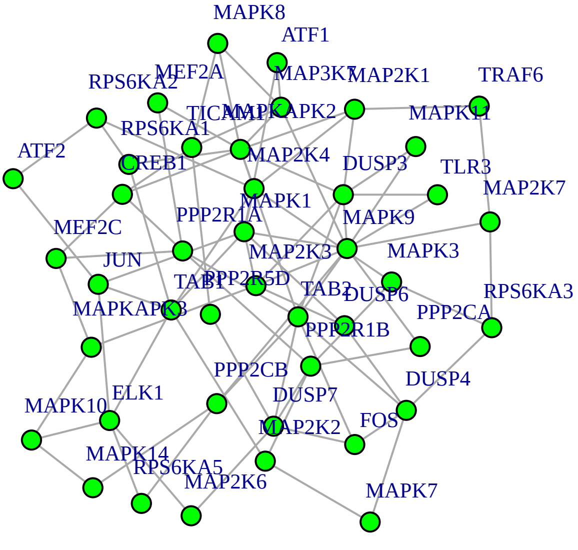

MST2 of the coexpression network for MUT p53

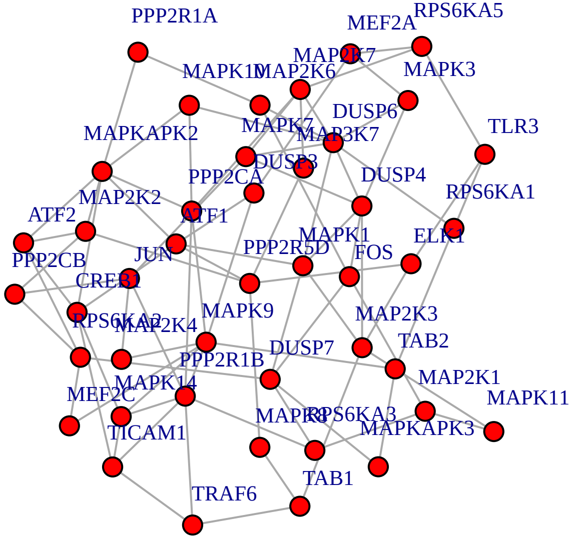

Pathway: REACTOME_CELL_JUNCTION_ORGANIZATION

There are 60 genes in this pathway. This pathway was detected by GSNCA

WT p53
Hub Gene (WT): ITGB1
Weight Factor: 1.401
Hub Gene (MUT): CLDN7
Weight Factor: 0.879

MUT p53
Hub Gene (MUT): CLDN7
Weight Factor: 1.508
Hub Gene (WT): ITGB1
Weight Factor: 1.178

MST2 of the coexpression network for WT p53

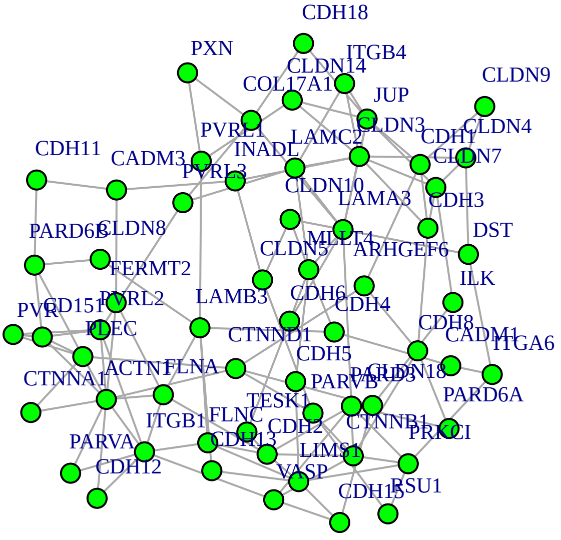

MST2 of the coexpression network for MUT p53

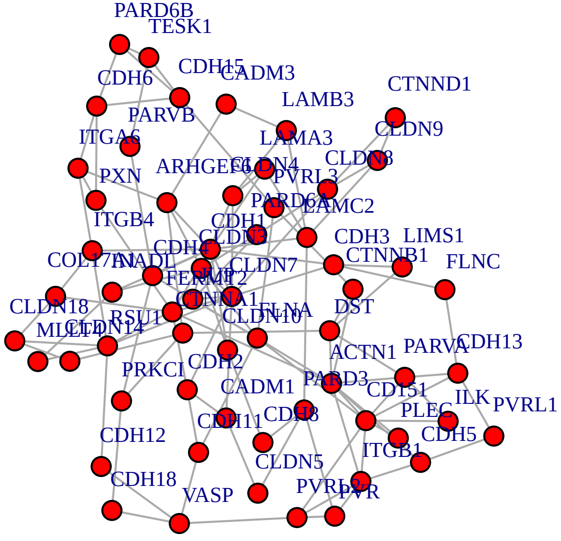

Pathway: ST_JNK_MAPK_PATHWAY

There are 36 genes in this pathway. This pathway was detected by GSNCA

WT p53
Hub Gene (WT): MAPK9
Weight Factor: 1.467
Hub Gene (MUT): PAPPA
Weight Factor: 0.652

MUT p53
Hub Gene (MUT): PAPPA
Weight Factor: 1.338
Hub Gene (WT): MAPK9
Weight Factor: 0.985

MST2 of the coexpression network for WT p53

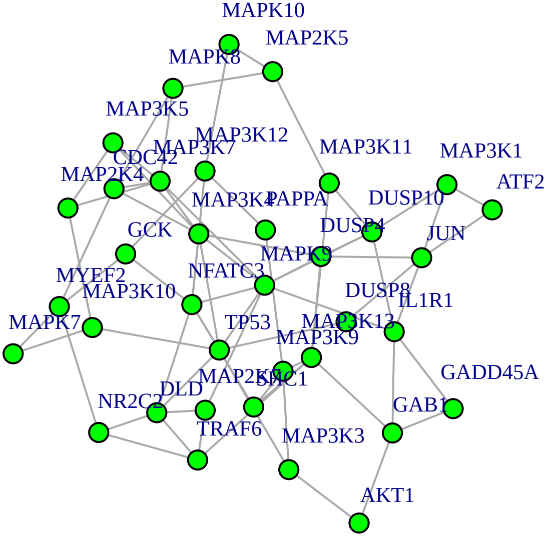

MST2 of the coexpression network for MUT p53

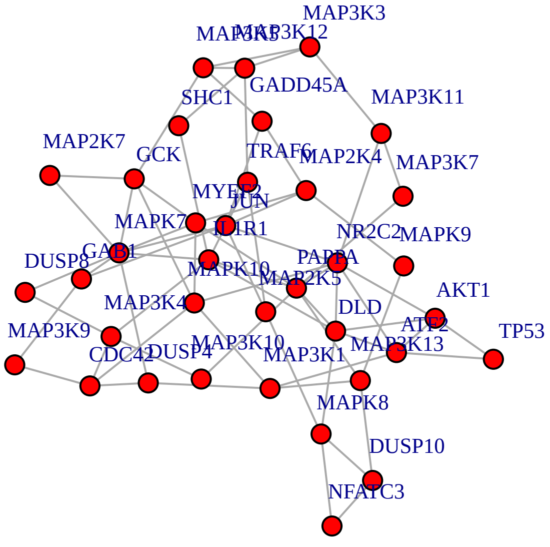

Pathway: NAKAMURA_TUMOR_ZONE_PERIPHERAL_VS_CENTRAL_UP

There are 170 genes in this pathway. This pathway was detected by GSCA

WT p53
Hub Gene (WT): CAV1
Weight Factor: 1.359
Hub Gene (MUT): PEA15
Weight Factor: 1.207

MUT p53
Hub Gene (MUT): PEA15
Weight Factor: 1.531
Hub Gene (WT): CAV1
Weight Factor: 1.339

MST2 of the coexpression network for WT p53

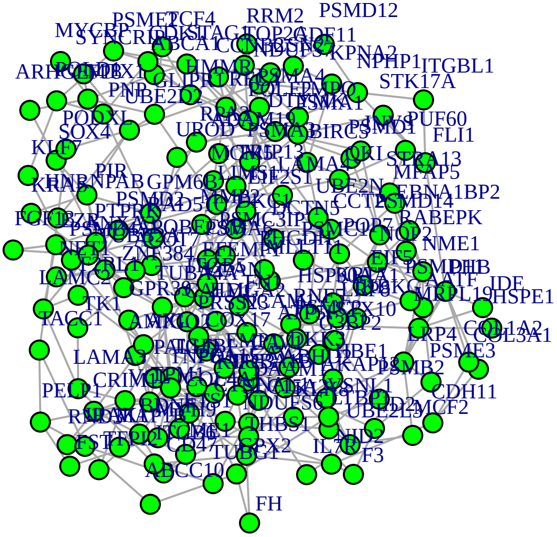

MST2 of the coexpression network for MUT p53

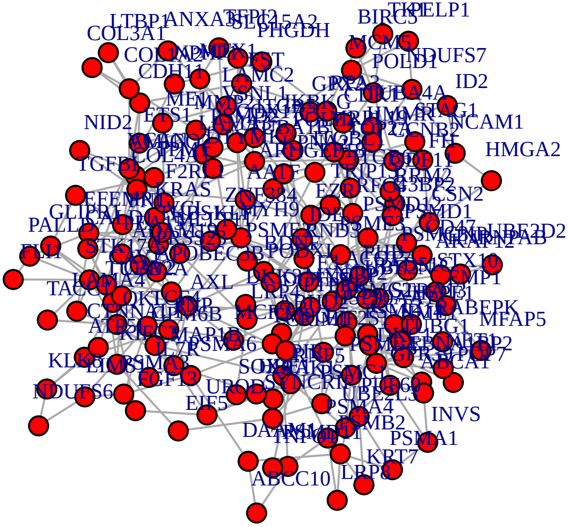

Pathway: TURASHVILI_BREAST_DUCTAL_CARCINOMA_VS_LOBULAR_NORMAL_DN

There are 47 genes in this pathway. This pathway was detected by GSCA

WT p53
Hub Gene (WT): GPM6B
Weight Factor: 1.342
Hub Gene (MUT): PTN
Weight Factor: 0.855

MUT p53
Hub Gene (MUT): PTN
Weight Factor: 1.56
Hub Gene (WT): GPM6B
Weight Factor: 0.903

MST2 of the coexpression network for WT p53

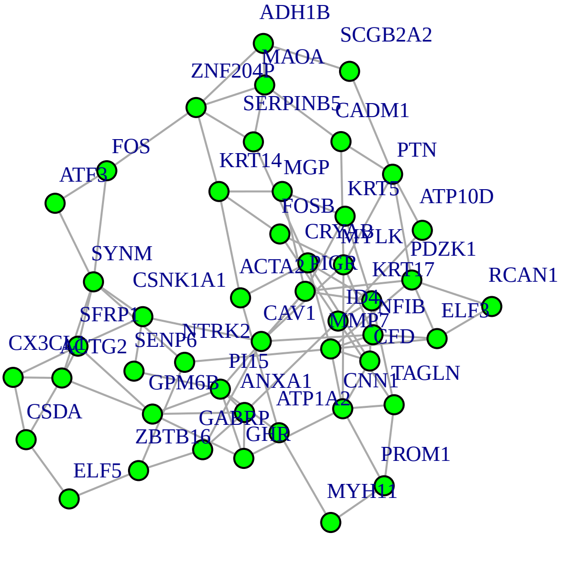

MST2 of the coexpression network for MUT p53

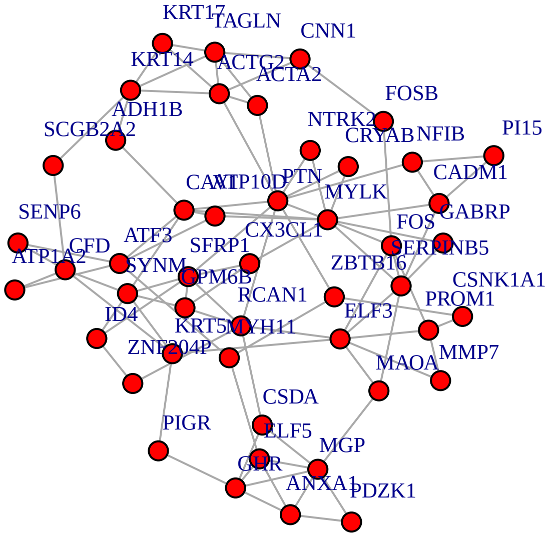

Pathway: CHARAFE_BREAST_CANCER_BASAL_VS_MESENCHYMAL_DN

There are 36 genes in this pathway. This pathway was detected by GSCA

WT p53
Hub Gene (WT): LOXL2
Weight Factor: 1.437
Hub Gene (MUT): LOXL2
Weight Factor: 1.437

MUT p53
Hub Gene (MUT): LOXL2
Weight Factor: 1.437
Hub Gene (WT): LOXL2
Weight Factor: 1.437

MST2 of the coexpression network for WT p53

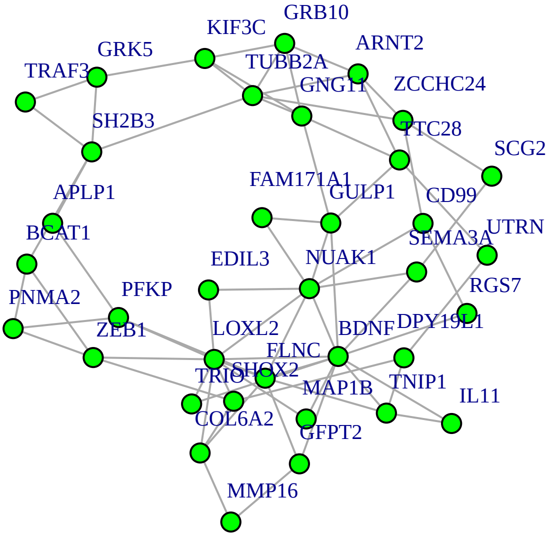

MST2 of the coexpression network for MUT p53

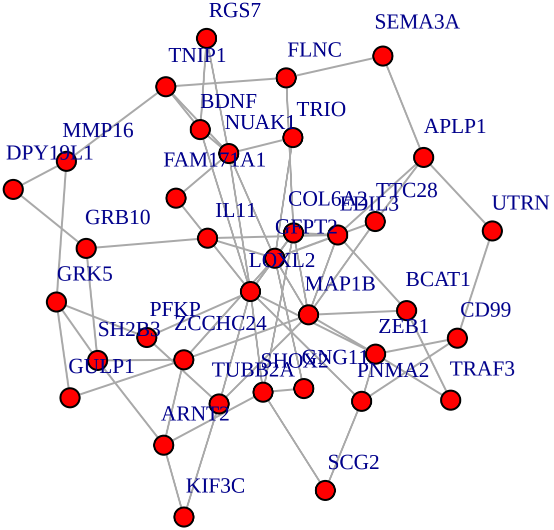

Pathway: NEWMAN_ERCC6_TARGETS_DN

There are 24 genes in this pathway. This pathway was detected by GSCA

WT p53
Hub Gene (WT): CDKN1A
Weight Factor: 1.239
Hub Gene (MUT): PSG9
Weight Factor: 0.996

MUT p53
Hub Gene (MUT): PSG9
Weight Factor: 1.447
Hub Gene (WT): CDKN1A
Weight Factor: 0.702

MST2 of the coexpression network for WT p53

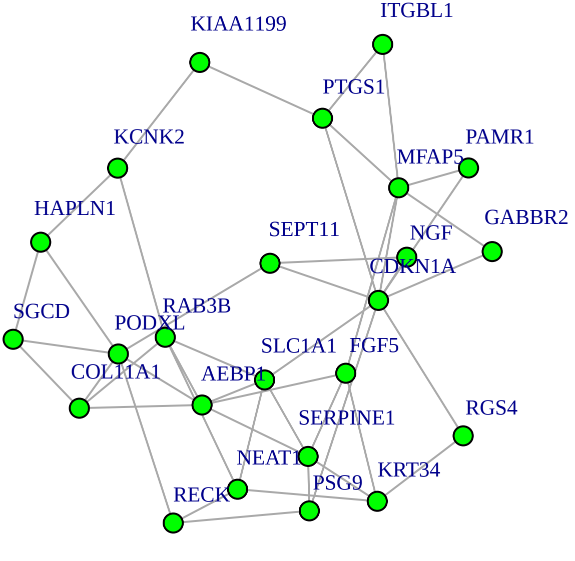

MST2 of the coexpression network for MUT p53

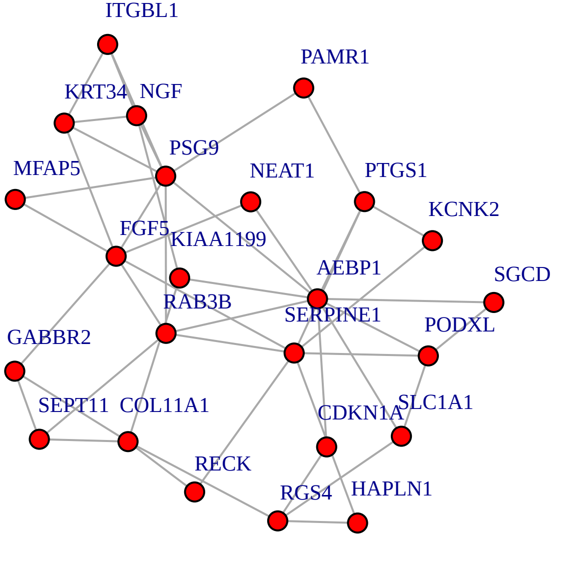

Pathway: GARGALOVIC_RESPONSE_TO_OXIDIZED_PHOSPHOLIPIDS_YELLOW_UP

There are 19 genes in this pathway. This pathway was detected by GSCA

WT p53
Hub Gene (WT): SLC7A1
Weight Factor: 1.211
Hub Gene (MUT): PPP3R1
Weight Factor: 1.211

MUT p53
Hub Gene (MUT): PPP3R1
Weight Factor: 1.213
Hub Gene (WT): SLC7A1
Weight Factor: 0.932

MST2 of the coexpression network for WT p53

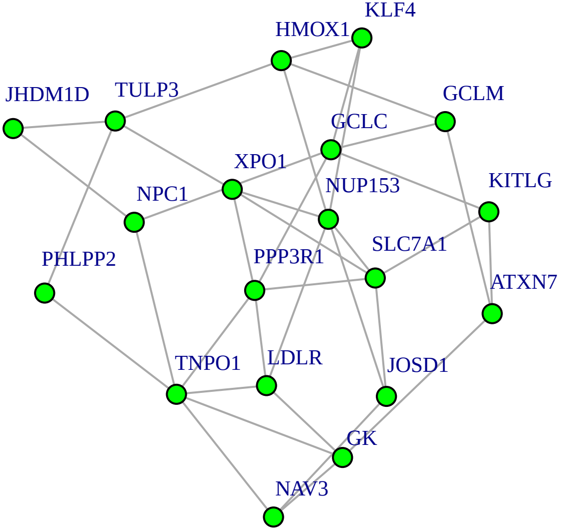

MST2 of the coexpression network for MUT p53

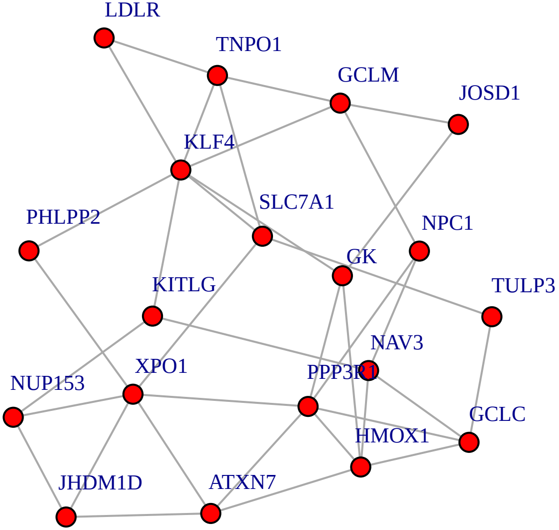

Pathway: BERENJENO_TRANSFORMED_BY_RHOA_FOREVER_UP

There are 18 genes in this pathway. This pathway was detected by GSCA

WT p53
Hub Gene (WT): MT1A
Weight Factor: 1.386
Hub Gene (MUT): MT1B
Weight Factor: 1.354

MUT p53
Hub Gene (MUT): MT1B
Weight Factor: 1.331
Hub Gene (WT): MT1A
Weight Factor: 1.319

MST2 of the coexpression network for WT p53

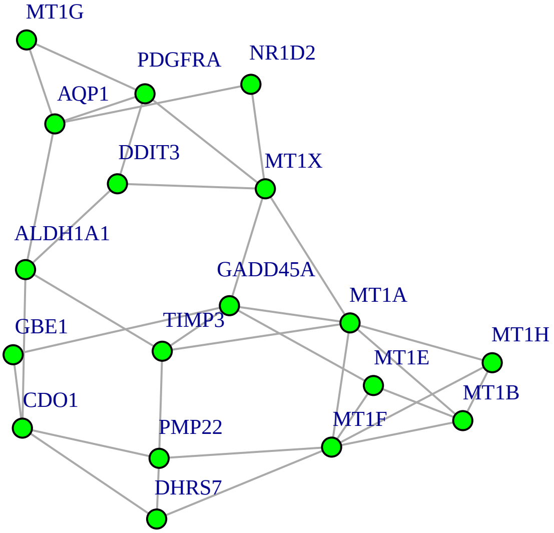

MST2 of the coexpression network for MUT p53

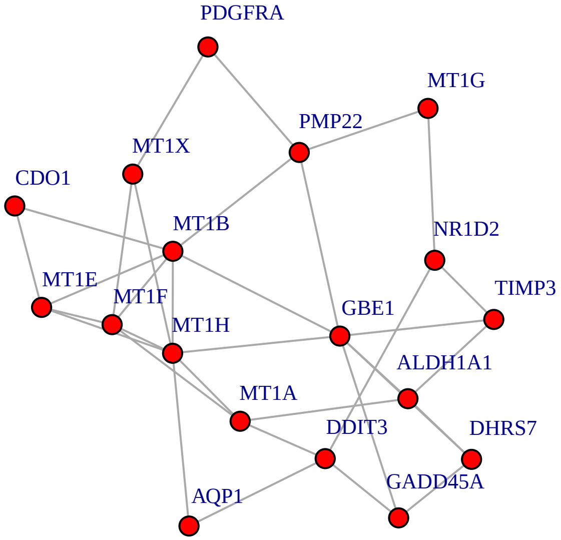

Pathway: BEGUM_TARGETS_OF_PAX3_FOXO1_FUSION_UP

There are 41 genes in this pathway. This pathway was detected by GSCA

WT p53
Hub Gene (WT): SH3GLB1
Weight Factor: 1.297
Hub Gene (MUT): SEMA3C
Weight Factor: 0.896

MUT p53
Hub Gene (MUT): SEMA3C
Weight Factor: 1.416
Hub Gene (WT): SH3GLB1
Weight Factor: 1.046

MST2 of the coexpression network for WT p53

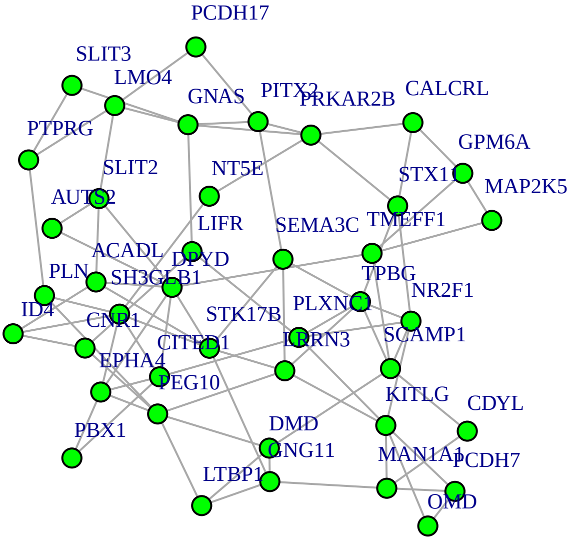

MST2 of the coexpression network for MUT p53

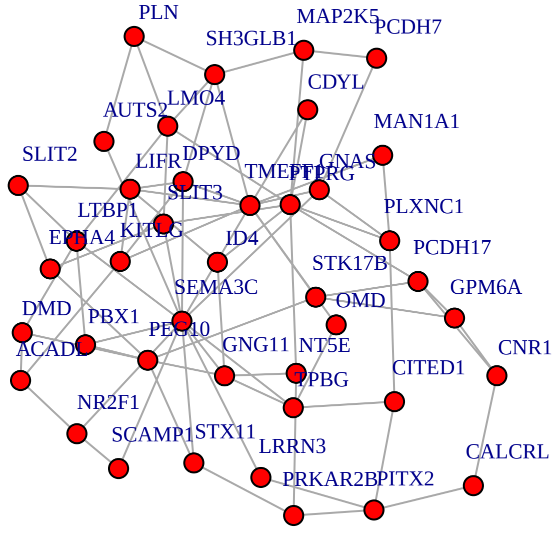

Pathway: MARKS_HDAC_TARGETS_UP

There are 19 genes in this pathway. This pathway was detected by GSCA

WT p53
Hub Gene (WT): DFFA
Weight Factor: 1.338
Hub Gene (MUT): TXN
Weight Factor: 1.078

MUT p53
Hub Gene (MUT): TXN
Weight Factor: 1.284
Hub Gene (WT): DFFA
Weight Factor: 1.038

MST2 of the coexpression network for WT p53

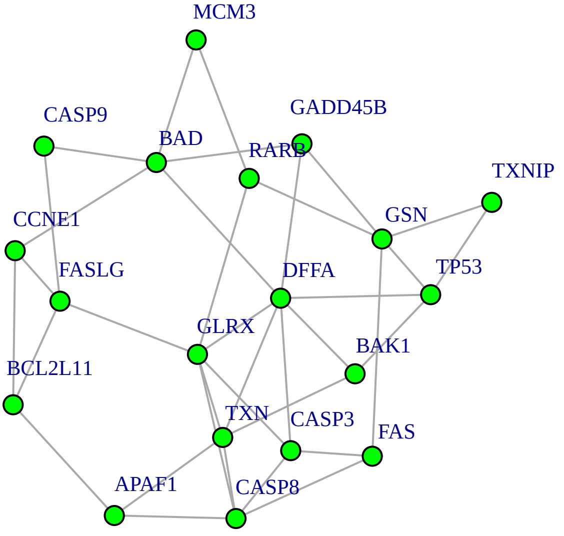

MST2 of the coexpression network for MUT p53

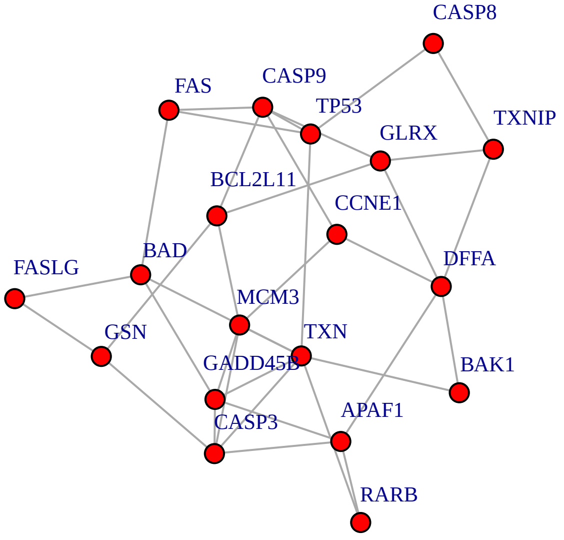

Pathway: WONG_ENDMETRIUM_CANCER_UP

There are 15 genes in this pathway. This pathway was detected by GSCA

WT p53
Hub Gene (WT): HP
Weight Factor: 1.309
Hub Gene (MUT): CCL20
Weight Factor: 0.936

MUT p53
Hub Gene (MUT): CCL20
Weight Factor: 1.189
Hub Gene (WT): HP
Weight Factor: 1.148

MST2 of the coexpression network for WT p53

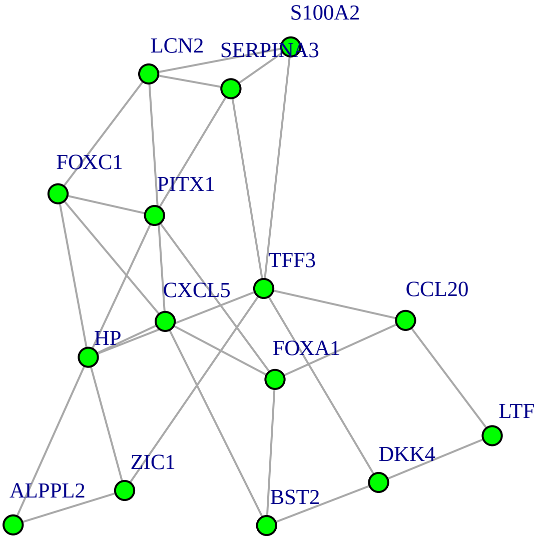

MST2 of the coexpression network for MUT p53

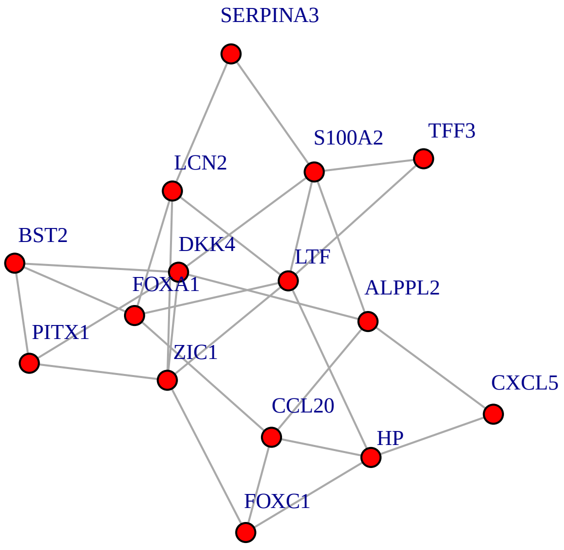

Pathway: DACOSTA_UV_RESPONSE_VIA_ERCC3_TTD_UP

There are 45 genes in this pathway. This pathway was detected by GSCA

WT p53
Hub Gene (WT): AES
Weight Factor: 1.375
Hub Gene (MUT): CDK16
Weight Factor: 1.236

MUT p53
Hub Gene (MUT): CDK16
Weight Factor: 1.322
Hub Gene (WT): AES
Weight Factor: 0.968

MST2 of the coexpression network for WT p53

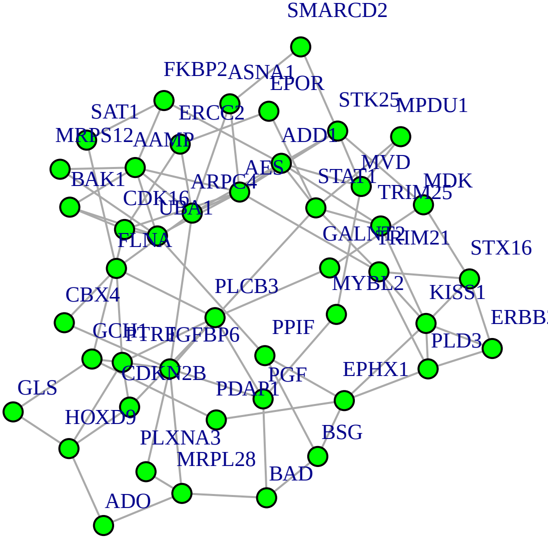

MST2 of the coexpression network for MUT p53

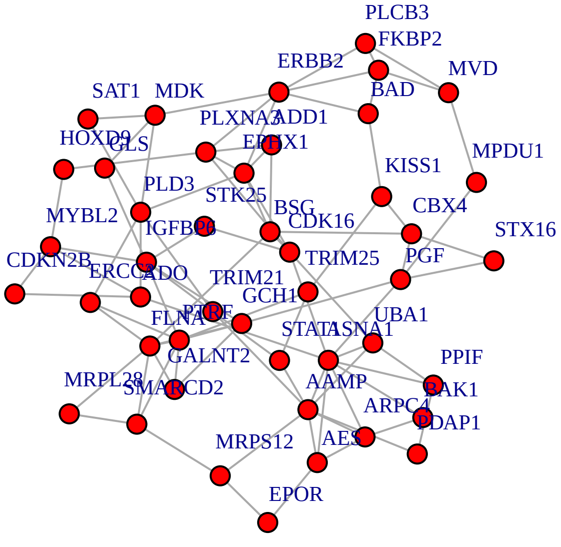

Pathway: CAVARD_LIVER_CANCER_MALIGNANT_VS_BENIGN

There are 21 genes in this pathway. This pathway was detected by GSCA

WT p53
Hub Gene (WT): PRDX1
Weight Factor: 1.218
Hub Gene (MUT): FGA
Weight Factor: 1.12

MUT p53
Hub Gene (MUT): FGA
Weight Factor: 1.211
Hub Gene (WT): PRDX1
Weight Factor: 0.665

MST2 of the coexpression network for WT p53

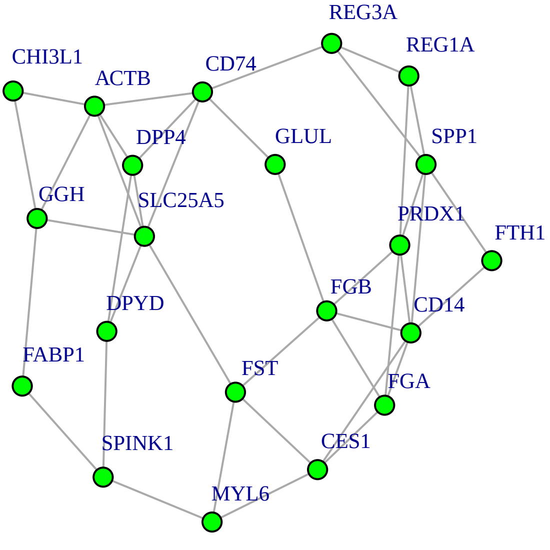

MST2 of the coexpression network for MUT p53

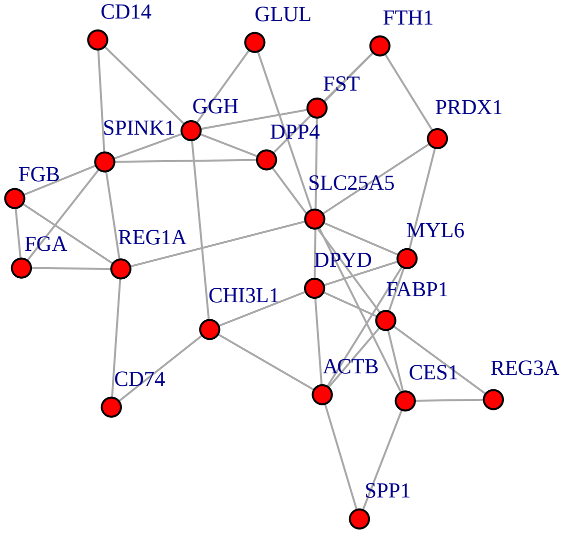

Pathway: SILIGAN_BOUND_BY_EWS_FLT1_FUSION

There are 26 genes in this pathway. This pathway was detected by GSCA

WT p53
Hub Gene (WT): ADCY1
Weight Factor: 1.264
Hub Gene (MUT): LTF
Weight Factor: 0.792

MUT p53
Hub Gene (MUT): LTF
Weight Factor: 1.259
Hub Gene (WT): ADCY1
Weight Factor: 0.914

MST2 of the coexpression network for WT p53

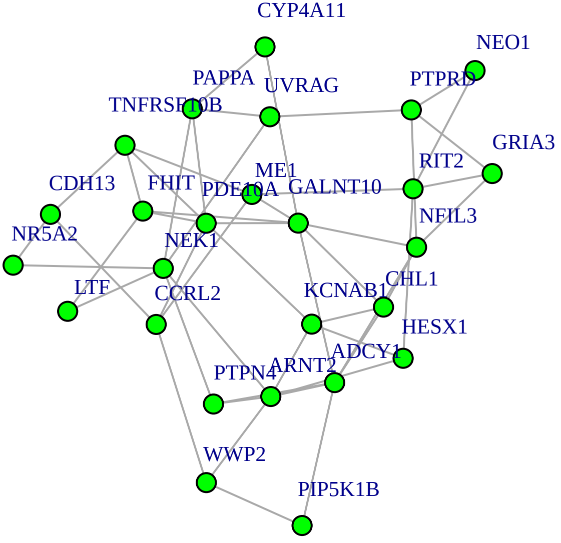

MST2 of the coexpression network for MUT p53

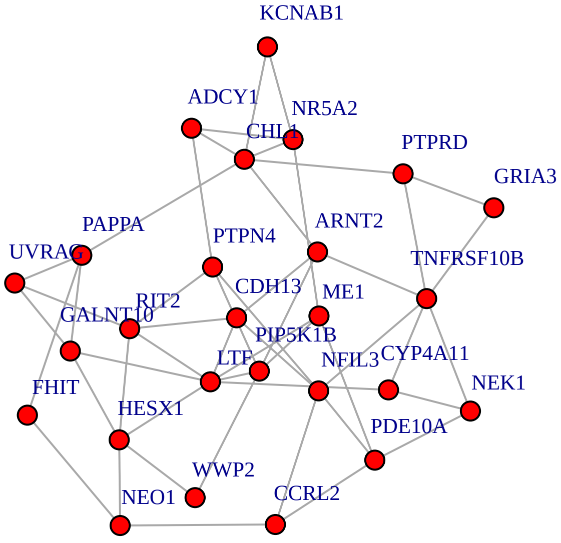

Pathway: RUGO_STRESS_RESPONSE_SUBSET_G

There are 31 genes in this pathway. This pathway was detected by GSCA

WT p53
Hub Gene (WT): PTPRN2
Weight Factor: 1.309
Hub Gene (MUT): IL1R1
Weight Factor: 1.068

MUT p53
Hub Gene (MUT): IL1R1
Weight Factor: 1.409
Hub Gene (WT): PTPRN2
Weight Factor: 0.79

MST2 of the coexpression network for WT p53

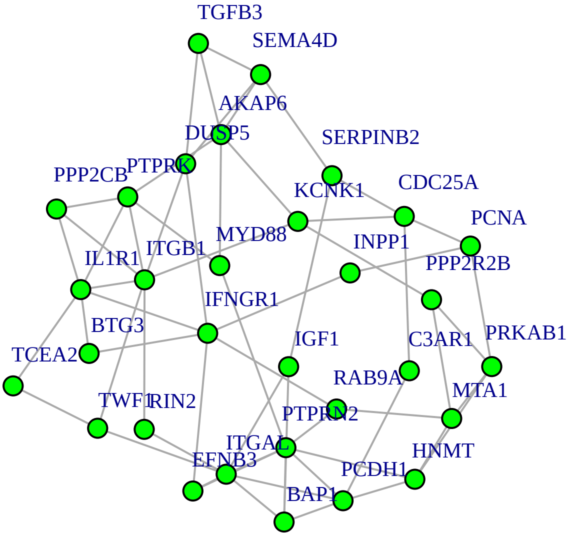

MST2 of the coexpression network for MUT p53

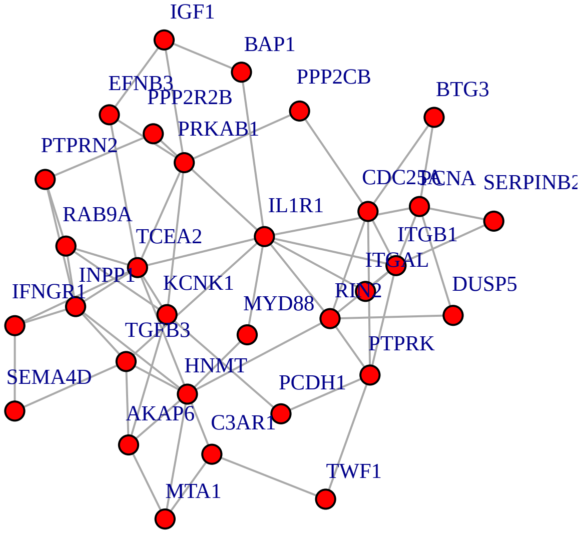

Pathway: WATTEL_AUTONOMOUS_THYROID_ADENOMA_UP

There are 18 genes in this pathway. This pathway was detected by GSCA

WT p53
Hub Gene (WT): DUSP9
Weight Factor: 1.285
Hub Gene (MUT): COL17A1
Weight Factor: 0.965

MUT p53
Hub Gene (MUT): COL17A1
Weight Factor: 1.31
Hub Gene (WT): DUSP9
Weight Factor: 1.03

MST2 of the coexpression network for WT p53

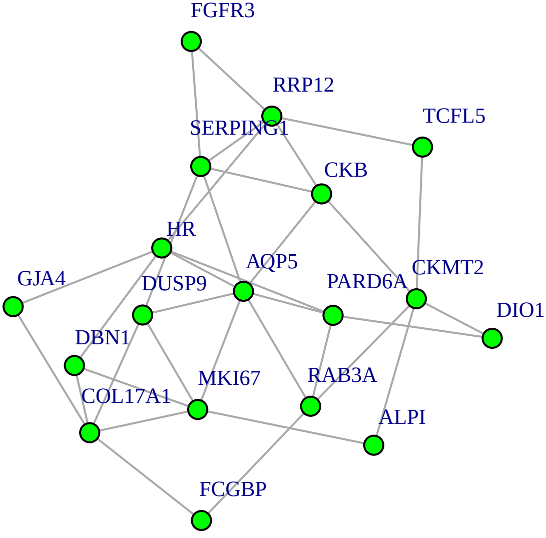

MST2 of the coexpression network for MUT p53

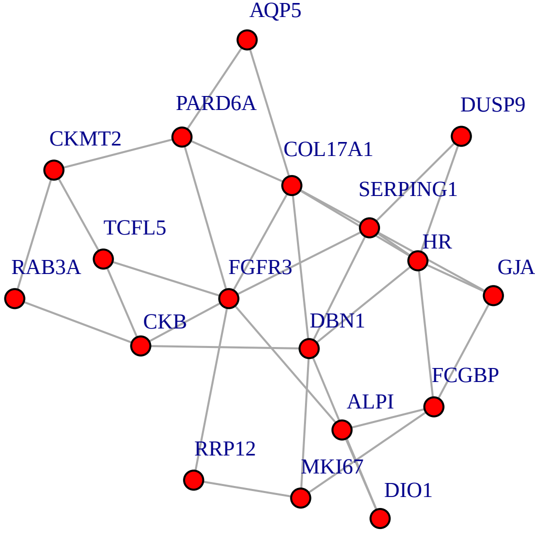

Pathway: RICKMAN_HEAD_AND_NECK_CANCER_A

There are 55 genes in this pathway. This pathway was detected by GSCA

WT p53
Hub Gene (WT): HOXD13
Weight Factor: 1.401
Hub Gene (MUT): MOXD1
Weight Factor: 0.91

MUT p53
Hub Gene (MUT): MOXD1
Weight Factor: 1.444
Hub Gene (WT): HOXD13
Weight Factor: 0.937

MST2 of the coexpression network for WT p53

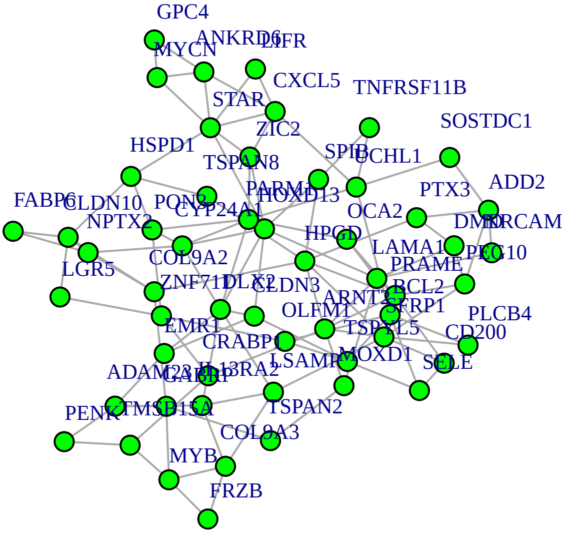

MST2 of the coexpression network for MUT p53

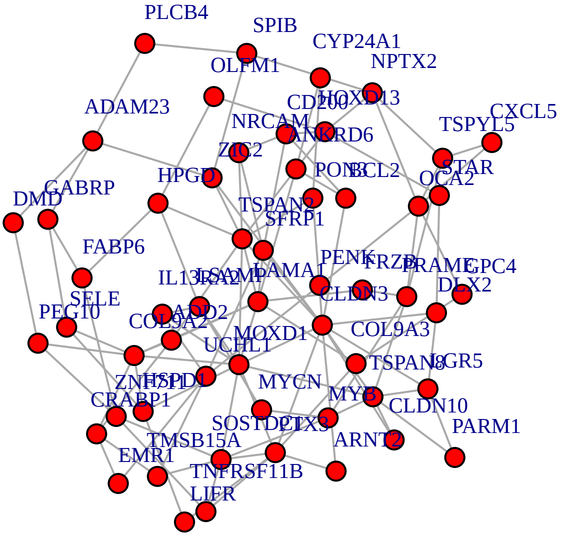

Pathway: ROZANOV_MMP14_TARGETS_SUBSET

There are 25 genes in this pathway. This pathway was detected by GSCA

WT p53
Hub Gene (WT): COL4A1
Weight Factor: 1.457
Hub Gene (MUT): COL4A1
Weight Factor: 1.457

MUT p53
Hub Gene (MUT): COL4A1
Weight Factor: 1.341
Hub Gene (WT): COL4A1
Weight Factor: 1.341

MST2 of the coexpression network for WT p53

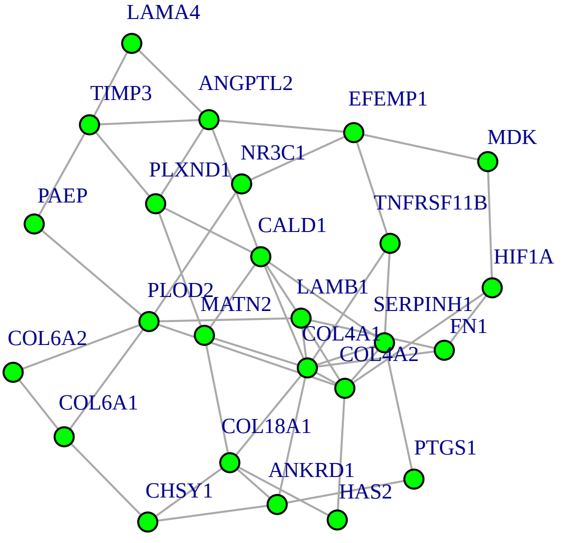

MST2 of the coexpression network for MUT p53

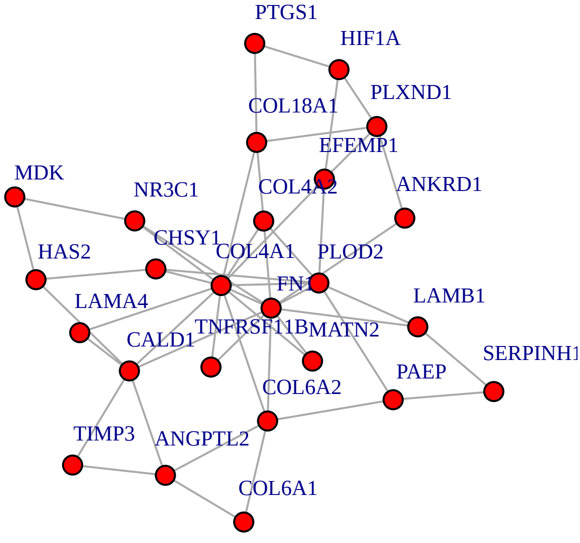

Pathway: KENNY_CTNNB1_TARGETS_UP

There are 37 genes in this pathway. This pathway was detected by GSCA

WT p53
Hub Gene (WT): RPA2
Weight Factor: 1.354
Hub Gene (MUT): PLK4
Weight Factor: 1.209

MUT p53
Hub Gene (MUT): PLK4
Weight Factor: 1.385
Hub Gene (WT): RPA2
Weight Factor: 1.154

MST2 of the coexpression network for WT p53

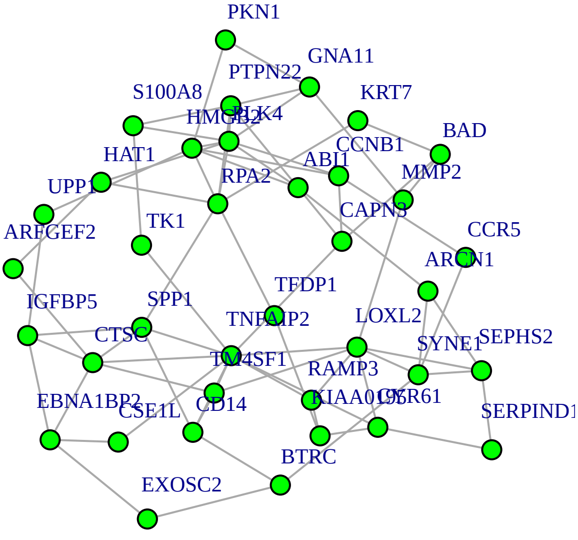

MST2 of the coexpression network for MUT p53

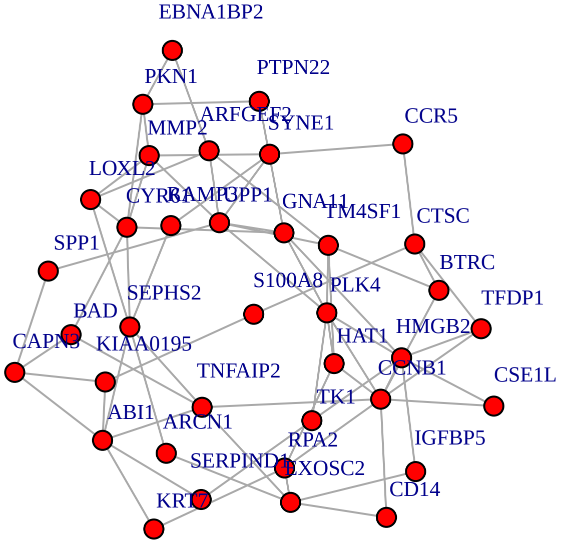

Pathway: ROSS_AML_WITH_PML_RARA_FUSION

There are 63 genes in this pathway. This pathway was detected by GSCA

WT p53
Hub Gene (WT): MXRA7
Weight Factor: 1.285
Hub Gene (MUT): MXRA7
Weight Factor: 1.285

MUT p53
Hub Gene (MUT): MXRA7
Weight Factor: 1.377
Hub Gene (WT): MXRA7
Weight Factor: 1.377

MST2 of the coexpression network for WT p53

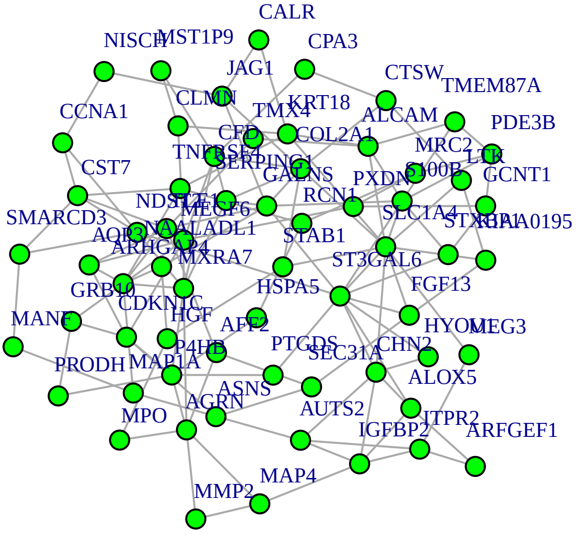

MST2 of the coexpression network for MUT p53

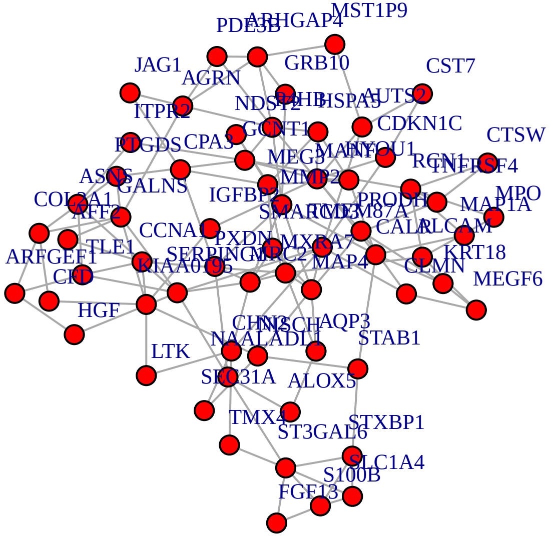

Pathway: SHEPARD_BMYB_TARGETS

There are 42 genes in this pathway. This pathway was detected by GSCA

WT p53
Hub Gene (WT): KIF14
Weight Factor: 1.414
Hub Gene (MUT): TTK
Weight Factor: 1.349

MUT p53
Hub Gene (MUT): TTK
Weight Factor: 1.502
Hub Gene (WT): KIF14
Weight Factor: 1.35

MST2 of the coexpression network for WT p53

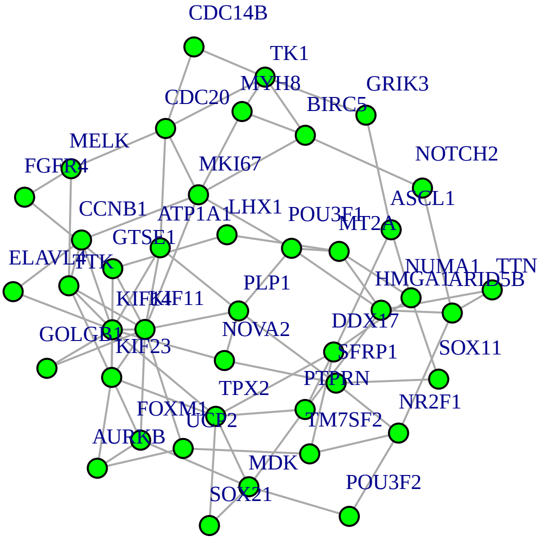

MST2 of the coexpression network for MUT p53

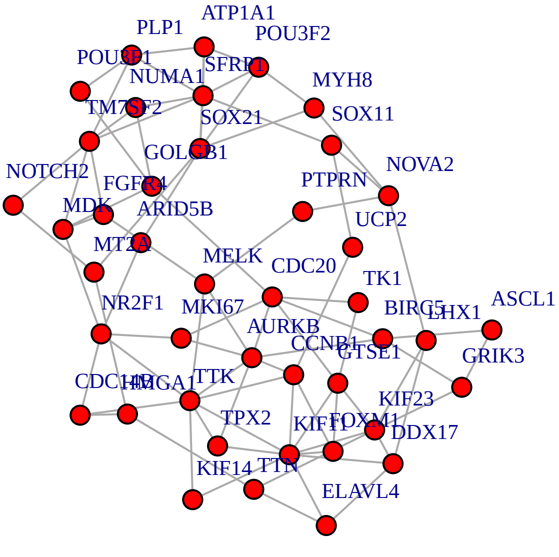

Pathway: ABRAHAM_ALPC_VS_MULTIPLE_MYELOMA_DN

There are 18 genes in this pathway. This pathway was detected by GSCA

WT p53
Hub Gene (WT): CDK5
Weight Factor: 1.334
Hub Gene (MUT): CDK4
Weight Factor: 0.97

MUT p53
Hub Gene (MUT): CDK4
Weight Factor: 1.344
Hub Gene (WT): CDK5
Weight Factor: 1.083

MST2 of the coexpression network for WT p53

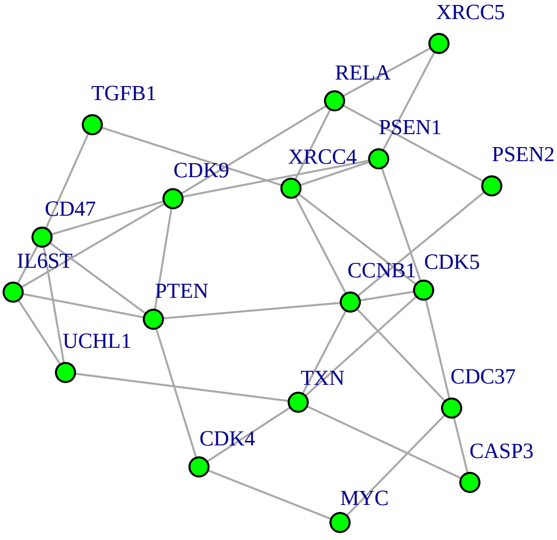

MST2 of the coexpression network for MUT p53

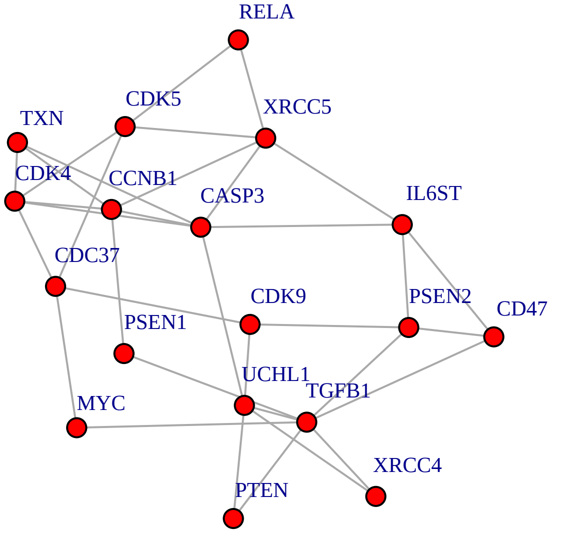

Pathway: KYNG_DNA_DAMAGE_BY_4NQO_OR_UV

There are 31 genes in this pathway. This pathway was detected by GSCA

WT p53
Hub Gene (WT): PTPRN2
Weight Factor: 1.309
Hub Gene (MUT): IL1R1
Weight Factor: 1.068

MUT p53
Hub Gene (MUT): IL1R1
Weight Factor: 1.409
Hub Gene (WT): PTPRN2
Weight Factor: 0.79

MST2 of the coexpression network for WT p53

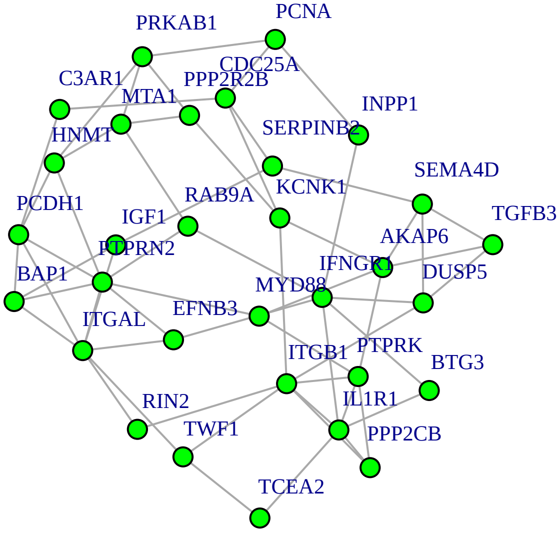

MST2 of the coexpression network for MUT p53

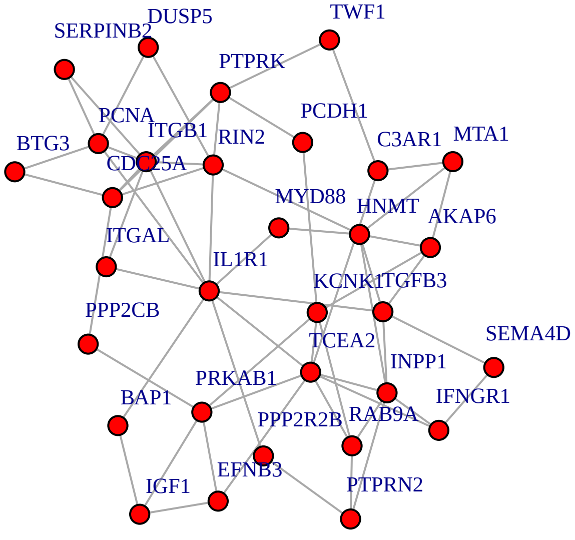

Pathway: YOKOE_CANCER_TESTIS_ANTIGENS

There are 16 genes in this pathway. This pathway was detected by GSCA

WT p53
Hub Gene (WT): GREB1
Weight Factor: 1.255
Hub Gene (MUT): DPEP1
Weight Factor: 0.869

MUT p53
Hub Gene (MUT): DPEP1
Weight Factor: 1.229
Hub Gene (WT): GREB1
Weight Factor: 1.018

MST2 of the coexpression network for WT p53

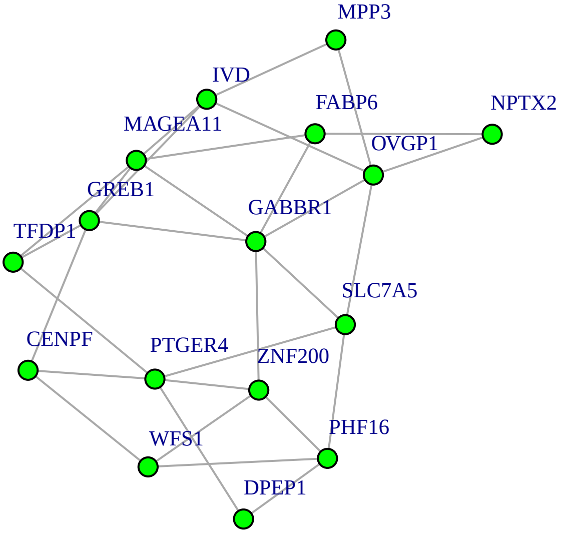

MST2 of the coexpression network for MUT p53

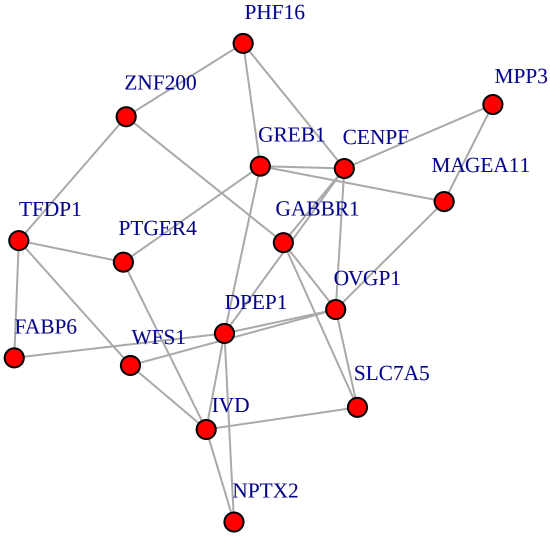

Pathway: FIRESTEIN_CTNNB1_PATHWAY

There are 24 genes in this pathway. This pathway was detected by GSCA

WT p53
Hub Gene (WT): LTK
Weight Factor: 1.331
Hub Gene (MUT): PLK4
Weight Factor: 1.314

MUT p53
Hub Gene (MUT): PLK4
Weight Factor: 1.297
Hub Gene (WT): LTK
Weight Factor: 0.688

MST2 of the coexpression network for WT p53

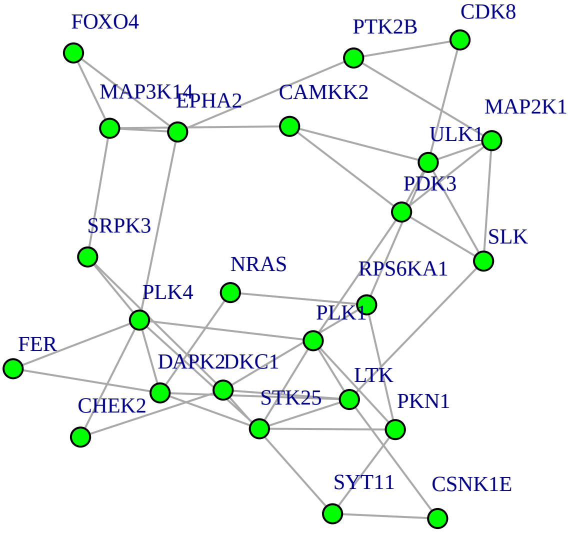

MST2 of the coexpression network for MUT p53

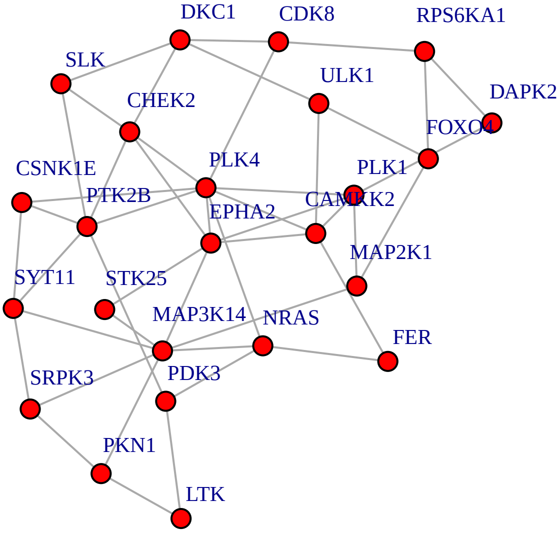

Pathway: BEIER_GLIOMA_STEM_CELL_UP

There are 17 genes in this pathway. This pathway was detected by GSCA

WT p53
Hub Gene (WT): SERPING1
Weight Factor: 1.301
Hub Gene (MUT): FGFR3
Weight Factor: 0.934

MUT p53
Hub Gene (MUT): FGFR3
Weight Factor: 1.235
Hub Gene (WT): SERPING1
Weight Factor: 1.005

MST2 of the coexpression network for WT p53

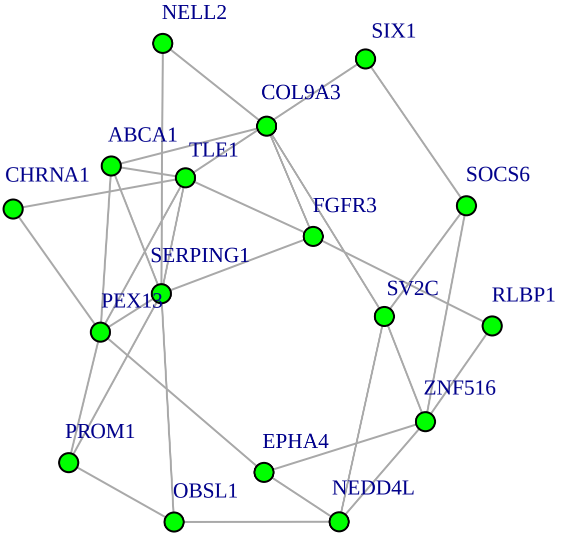

MST2 of the coexpression network for MUT p53

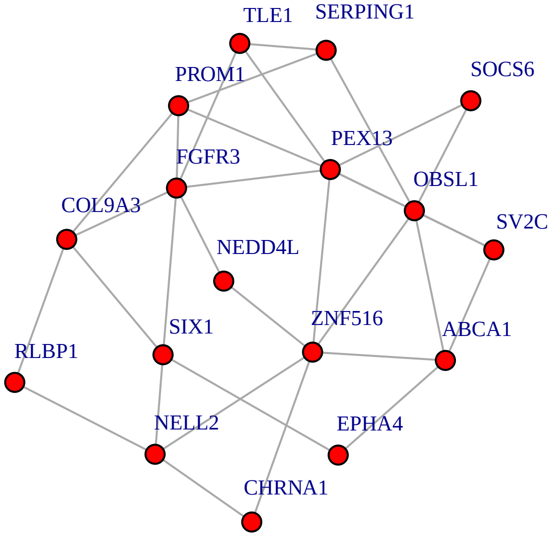

Pathway: CROONQUIST_NRAS_VS_STROMAL_STIMULATION_UP

There are 34 genes in this pathway. This pathway was detected by GSCA

WT p53
Hub Gene (WT): DUSP4
Weight Factor: 1.359
Hub Gene (MUT): SYNM
Weight Factor: 0.915

MUT p53
Hub Gene (MUT): SYNM
Weight Factor: 1.441
Hub Gene (WT): DUSP4
Weight Factor: 1.198

MST2 of the coexpression network for WT p53

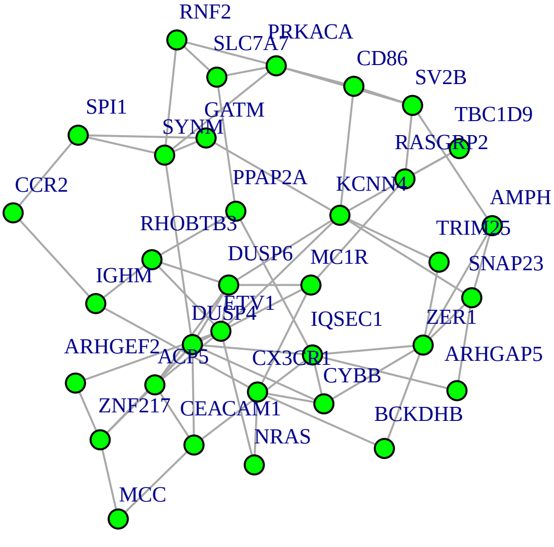

MST2 of the coexpression network for MUT p53

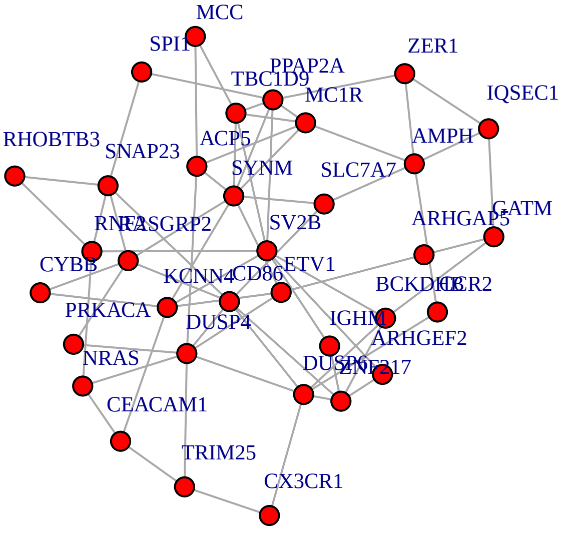

Pathway: BOYAULT_LIVER_CANCER_SUBCLASS_G3_DN

There are 42 genes in this pathway. This pathway was detected by GSCA

WT p53
Hub Gene (WT): SERPINF1
Weight Factor: 1.369
Hub Gene (MUT): ALDH5A1
Weight Factor: 0.828

MUT p53
Hub Gene (MUT): ALDH5A1
Weight Factor: 1.25
Hub Gene (WT): SERPINF1
Weight Factor: 1.004

MST2 of the coexpression network for WT p53

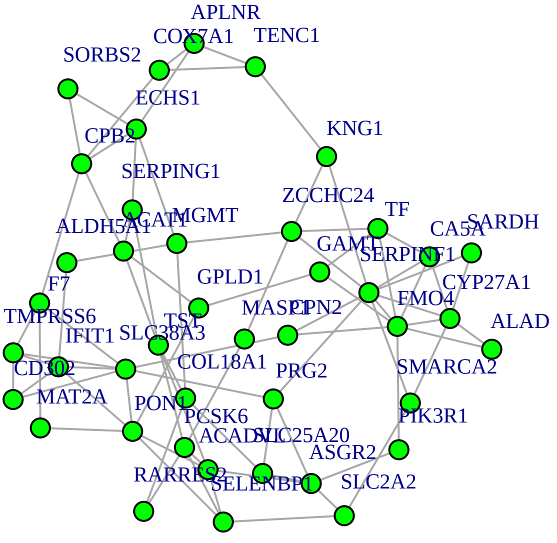

MST2 of the coexpression network for MUT p53

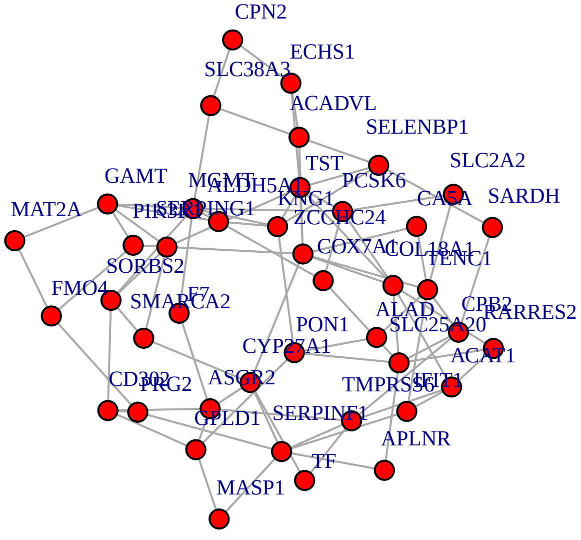

Pathway: WOO_LIVER_CANCER_RECURRENCE_DN

There are 64 genes in this pathway. This pathway was detected by GSCA

WT p53
Hub Gene (WT): CYP4F12
Weight Factor: 1.375
Hub Gene (MUT): CYP2J2
Weight Factor: 0.839

MUT p53
Hub Gene (MUT): CYP2J2
Weight Factor: 1.3
Hub Gene (WT): CYP4F12
Weight Factor: 0.958

MST2 of the coexpression network for WT p53

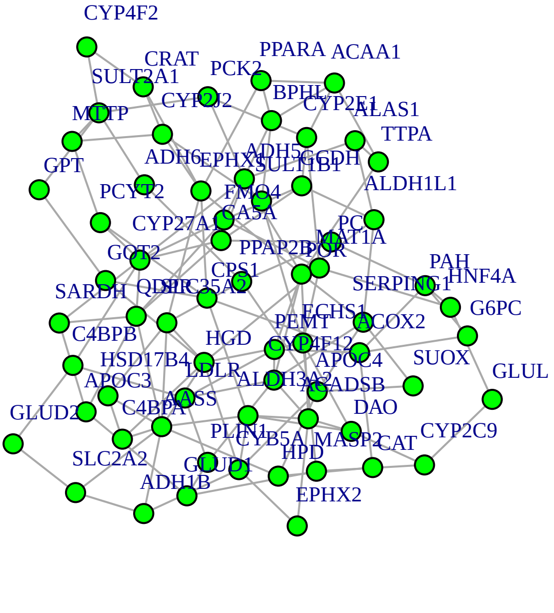

MST2 of the coexpression network for MUT p53

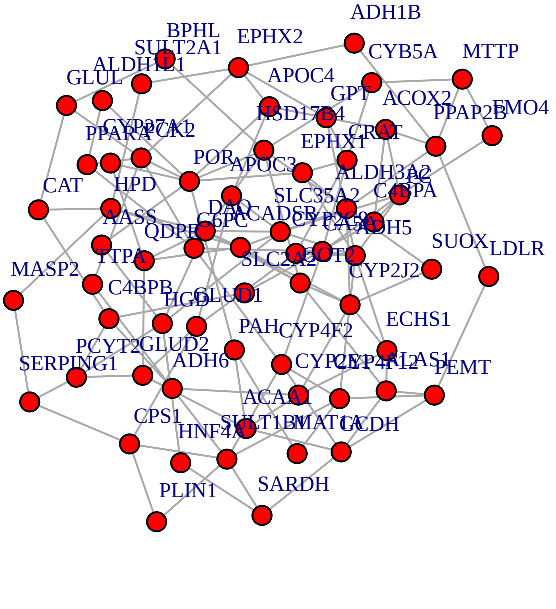

Pathway: CAIRO_HEPATOBLASTOMA_POOR_SURVIVAL

There are 16 genes in this pathway. This pathway was detected by GSCA

WT p53
Hub Gene (WT): DUSP9
Weight Factor: 1.265
Hub Gene (MUT): NLE1
Weight Factor: 1.057

MUT p53
Hub Gene (MUT): NLE1
Weight Factor: 1.463
Hub Gene (WT): DUSP9
Weight Factor: 1.041

MST2 of the coexpression network for WT p53

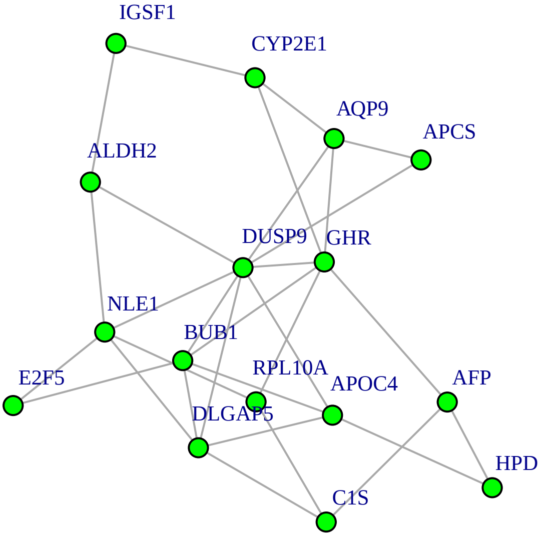

MST2 of the coexpression network for MUT p53

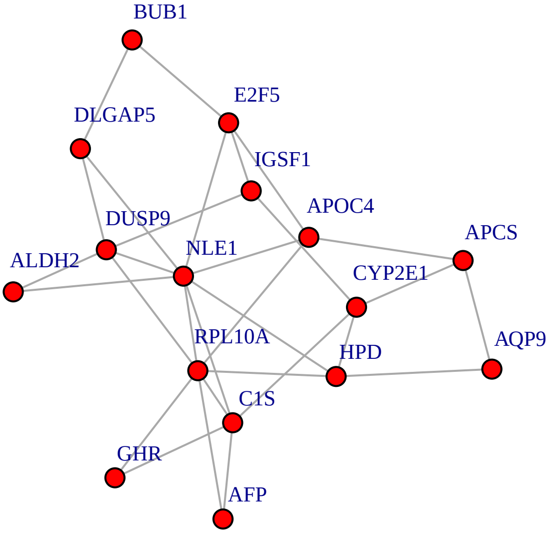

Pathway: DORN_ADENOVIRUS_INFECTION_12HR_DN

There are 24 genes in this pathway. This pathway was detected by GSCA

WT p53
Hub Gene (WT): HIST1H4B
Weight Factor: 1.283
Hub Gene (MUT): CCND1
Weight Factor: 1.128

MUT p53
Hub Gene (MUT): CCND1
Weight Factor: 1.266
Hub Gene (WT): HIST1H4B
Weight Factor: 0.935

MST2 of the coexpression network for WT p53

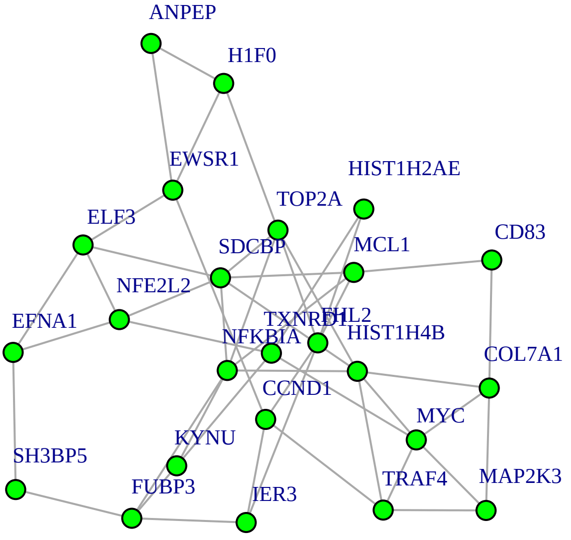

MST2 of the coexpression network for MUT p53

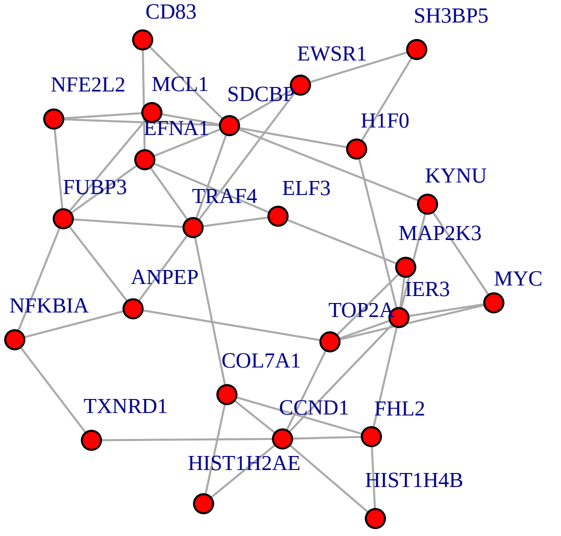

Pathway: SASSON_RESPONSE_TO_FORSKOLIN_DN

There are 66 genes in this pathway. This pathway was detected by GSCA

WT p53
Hub Gene (WT): AXL
Weight Factor: 1.268
Hub Gene (MUT): AXL
Weight Factor: 1.268

MUT p53
Hub Gene (MUT): AXL
Weight Factor: 1.487
Hub Gene (WT): AXL
Weight Factor: 1.487

MST2 of the coexpression network for WT p53

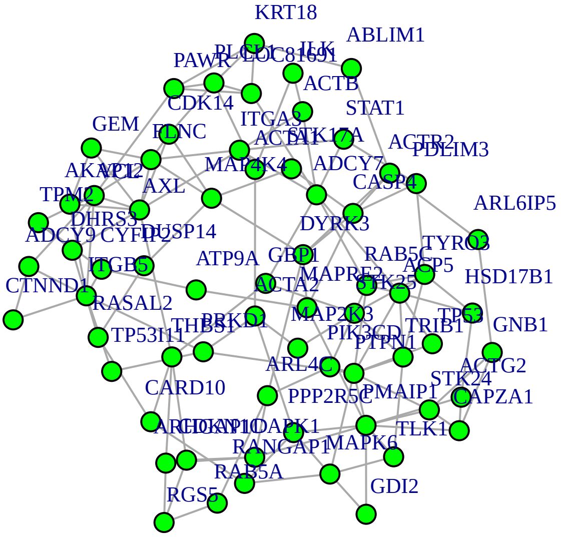

MST2 of the coexpression network for MUT p53

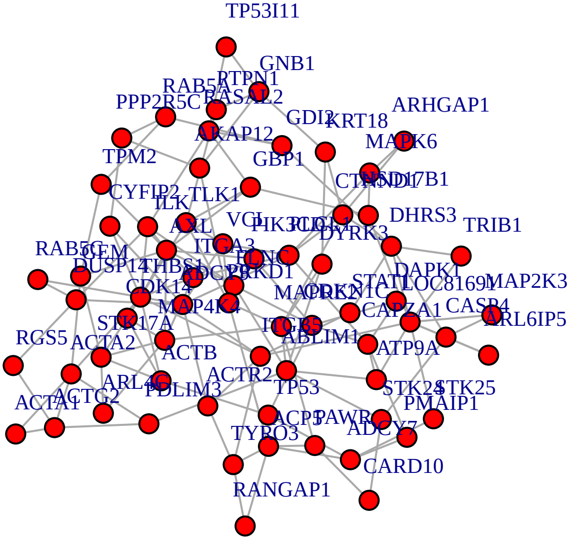

Pathway: ZHAN_MULTIPLE_MYELOMA_LB_UP

There are 17 genes in this pathway. This pathway was detected by GSCA

WT p53
Hub Gene (WT): MBD2
Weight Factor: 1.438
Hub Gene (MUT): PLS1
Weight Factor: 1.043

MUT p53
Hub Gene (MUT): PLS1
Weight Factor: 1.299
Hub Gene (WT): MBD2
Weight Factor: 1.184

MST2 of the coexpression network for WT p53

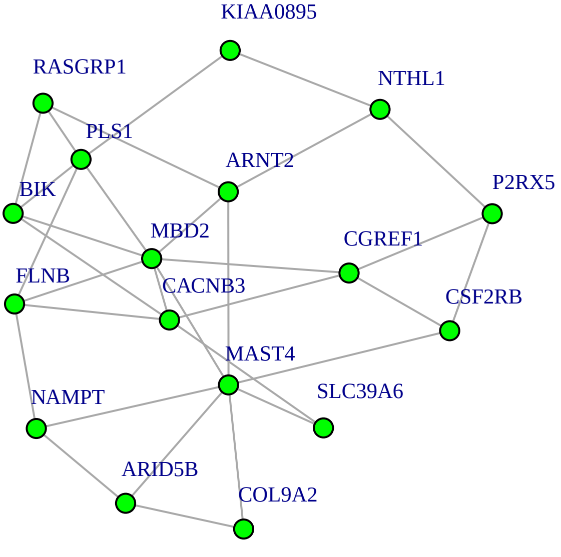

MST2 of the coexpression network for MUT p53

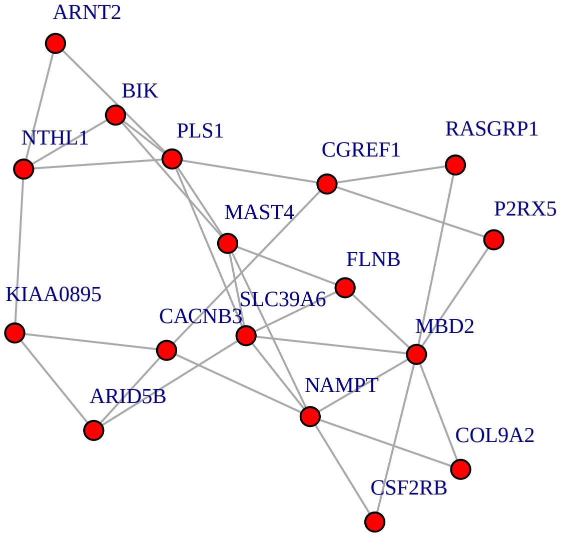

Pathway: SATO_SILENCED_BY_METHYLATION_IN_PANCREATIC_CANCER_2

There are 38 genes in this pathway. This pathway was detected by GSCA

WT p53
Hub Gene (WT): TJP2
Weight Factor: 1.256
Hub Gene (MUT): BNIP3
Weight Factor: 1.066

MUT p53
Hub Gene (MUT): BNIP3
Weight Factor: 1.369
Hub Gene (WT): TJP2
Weight Factor: 1.254

MST2 of the coexpression network for WT p53

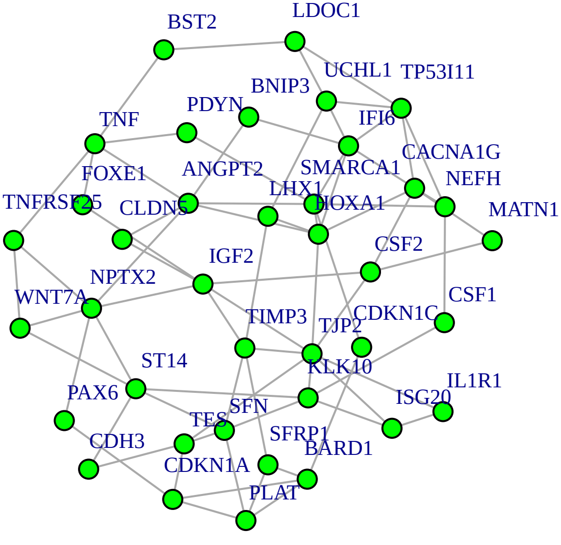

MST2 of the coexpression network for MUT p53

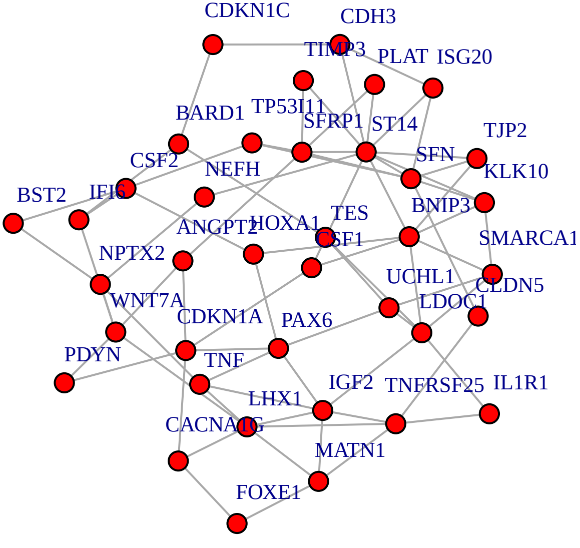

Pathway: BAELDE_DIABETIC_NEPHROPATHY_DN

There are 35 genes in this pathway. This pathway was detected by GSCA

WT p53
Hub Gene (WT): ST3GAL6
Weight Factor: 1.26
Hub Gene (MUT): CDS1
Weight Factor: 1.068

MUT p53
Hub Gene (MUT): CDS1
Weight Factor: 1.301
Hub Gene (WT): ST3GAL6
Weight Factor: 1.238

MST2 of the coexpression network for WT p53

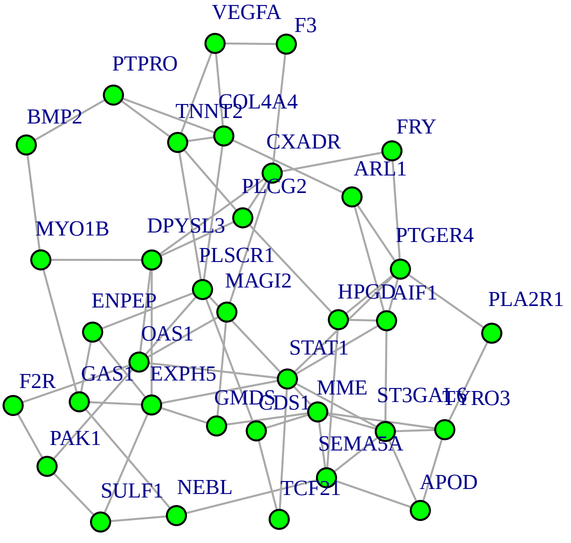

MST2 of the coexpression network for MUT p53

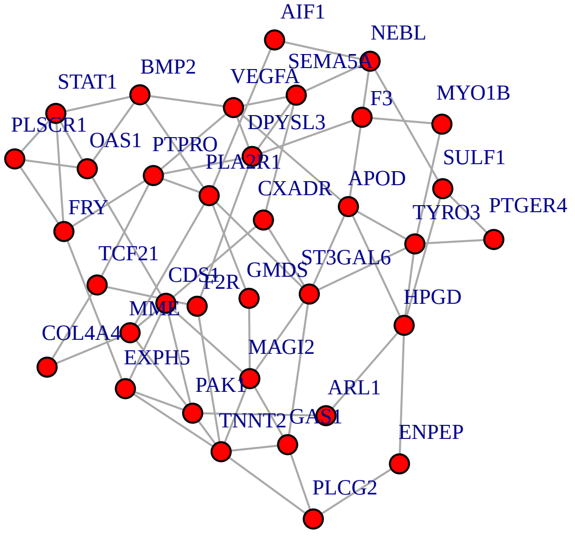

Pathway: SU_TESTIS

There are 75 genes in this pathway. This pathway was detected by GSCA

WT p53
Hub Gene (WT): RFC4
Weight Factor: 1.472
Hub Gene (MUT): RFC4
Weight Factor: 1.472

MUT p53
Hub Gene (MUT): RFC4
Weight Factor: 1.565
Hub Gene (WT): RFC4
Weight Factor: 1.565

MST2 of the coexpression network for WT p53

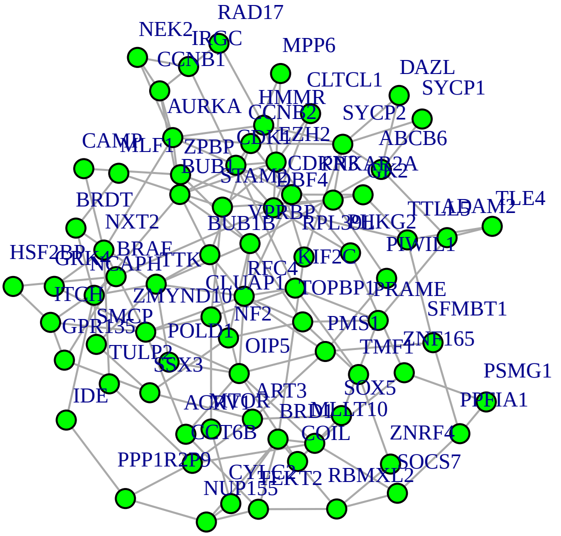

MST2 of the coexpression network for MUT p53

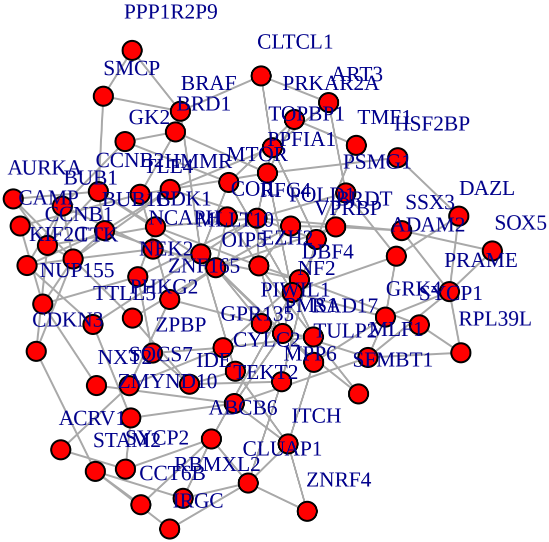

Pathway: CUI_TCF21_TARGETS_UP

There are 29 genes in this pathway. This pathway was detected by GSCA

WT p53
Hub Gene (WT): ISLR
Weight Factor: 1.427
Hub Gene (MUT): COL6A2
Weight Factor: 1.097

MUT p53
Hub Gene (MUT): COL6A2
Weight Factor: 1.455
Hub Gene (WT): ISLR
Weight Factor: 0.721

MST2 of the coexpression network for WT p53

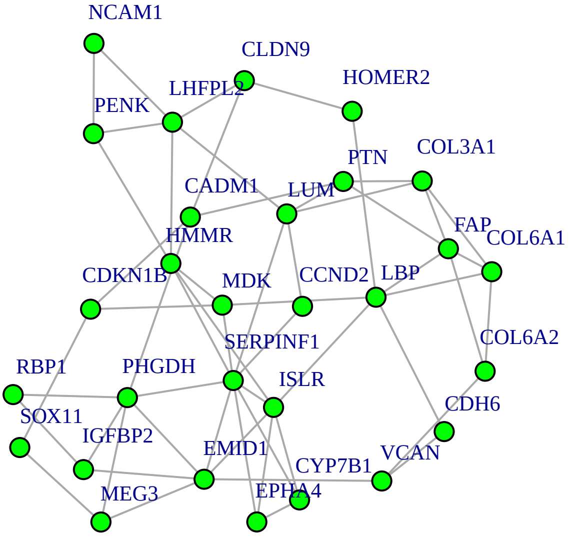

MST2 of the coexpression network for MUT p53

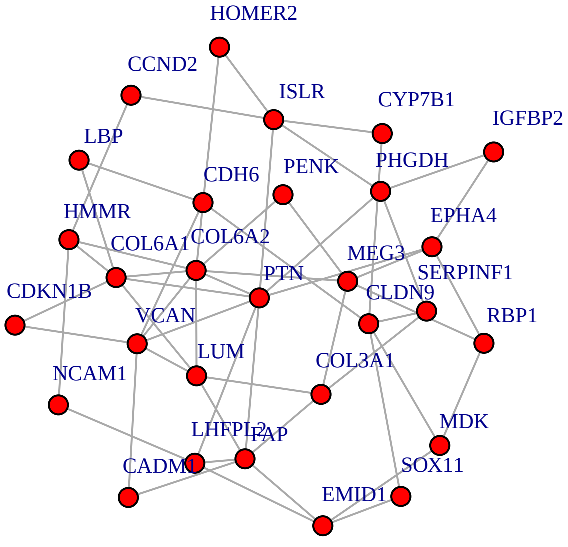

Pathway: KEGG_PHENYLALANINE_METABOLISM

There are 17 genes in this pathway. This pathway was detected by GSCA

WT p53
Hub Gene (WT): ALDH3A1
Weight Factor: 1.387
Hub Gene (MUT): GOT1
Weight Factor: 1.065

MUT p53
Hub Gene (MUT): GOT1
Weight Factor: 1.367
Hub Gene (WT): ALDH3A1
Weight Factor: 1.169

MST2 of the coexpression network for WT p53

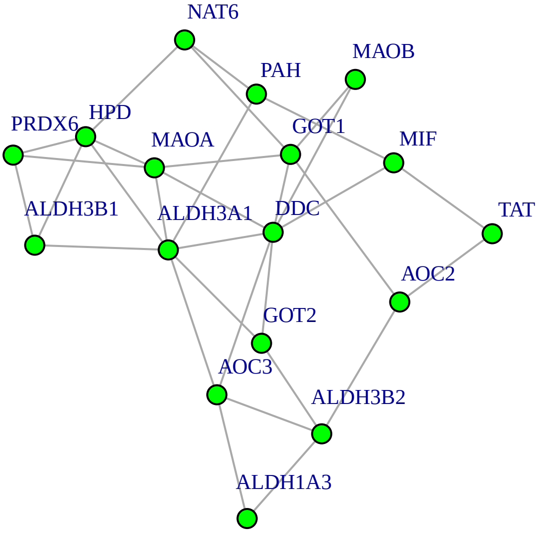

MST2 of the coexpression network for MUT p53

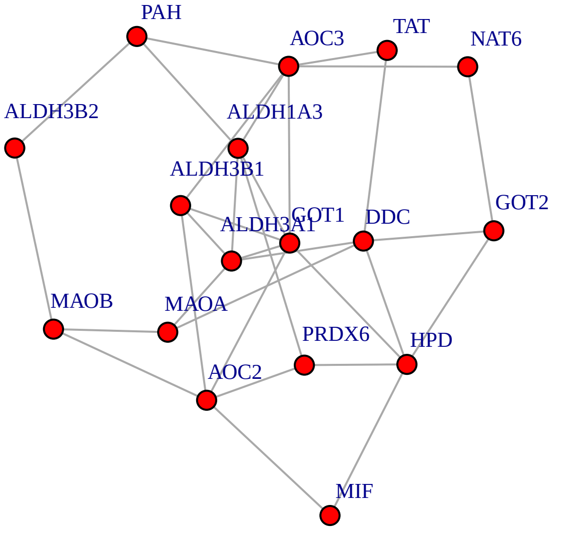

Pathway: KEGG_SELENOAMINO_ACID_METABOLISM

There are 15 genes in this pathway. This pathway was detected by GSCA

WT p53
Hub Gene (WT): PAPSS1
Weight Factor: 1.361
Hub Gene (MUT): AHCY
Weight Factor: 0.941

MUT p53
Hub Gene (MUT): AHCY
Weight Factor: 1.337
Hub Gene (WT): PAPSS1
Weight Factor: 0.857

MST2 of the coexpression network for WT p53

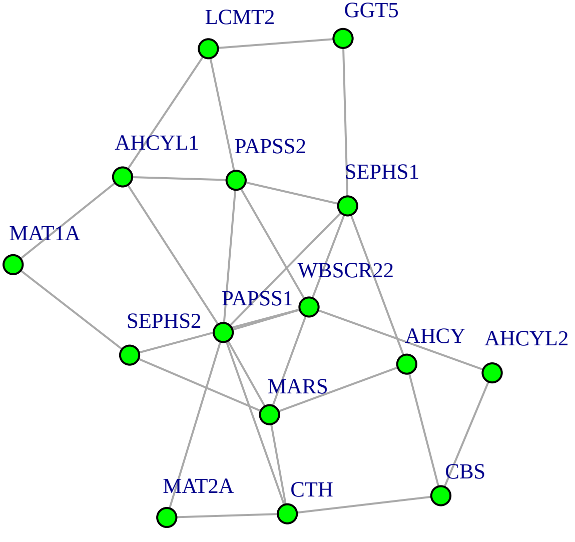

MST2 of the coexpression network for MUT p53

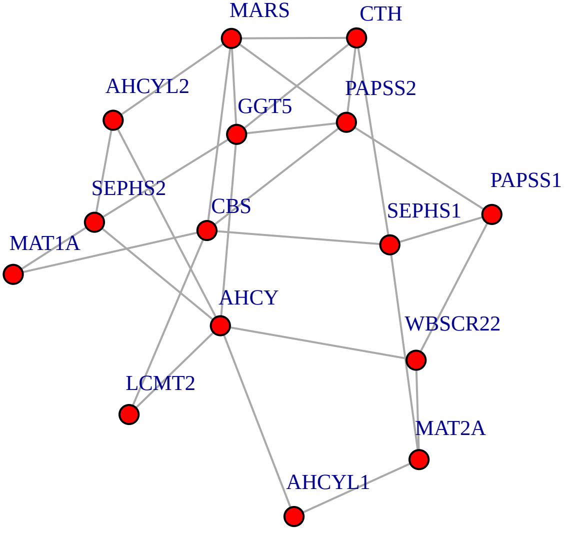

Pathway: KEGG_AMINO_SUGAR_AND_NUCLEOTIDE_SUGAR_METABOLISM

There are 28 genes in this pathway. This pathway was detected by GSCA

WT p53
Hub Gene (WT): PGM1
Weight Factor: 1.379
Hub Gene (MUT): GFPT1
Weight Factor: 0.715

MUT p53
Hub Gene (MUT): GFPT1
Weight Factor: 1.421
Hub Gene (WT): PGM1
Weight Factor: 1.199

MST2 of the coexpression network for WT p53

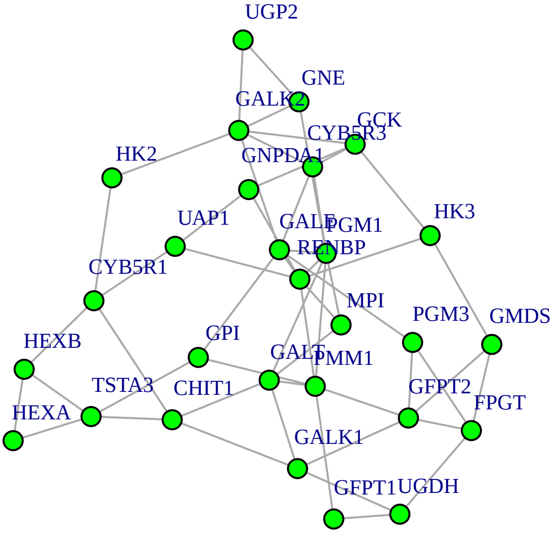

MST2 of the coexpression network for MUT p53

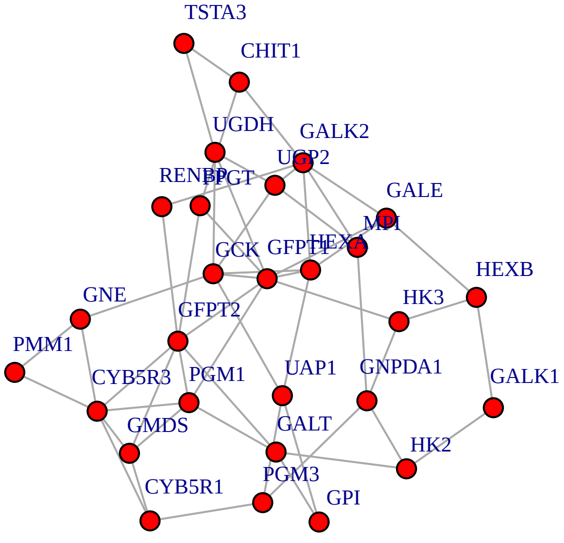

Pathway: KEGG_SPHINGOLIPID_METABOLISM

There are 24 genes in this pathway. This pathway was detected by GSCA

WT p53
Hub Gene (WT): GBA
Weight Factor: 1.366
Hub Gene (MUT): DEGS1
Weight Factor: 0.615

MUT p53
Hub Gene (MUT): DEGS1
Weight Factor: 1.435
Hub Gene (WT): GBA
Weight Factor: 1.057

MST2 of the coexpression network for WT p53

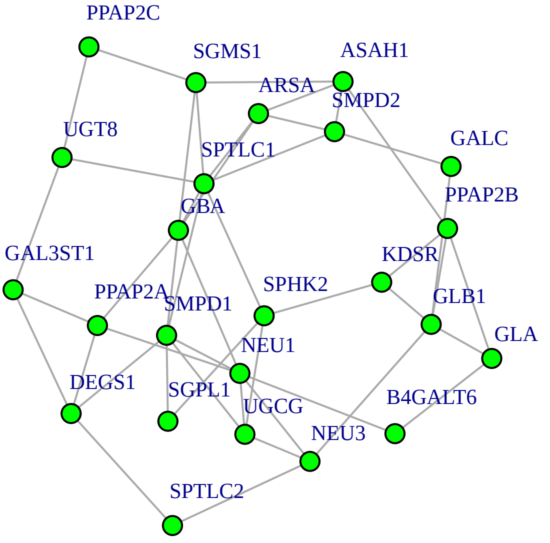

MST2 of the coexpression network for MUT p53

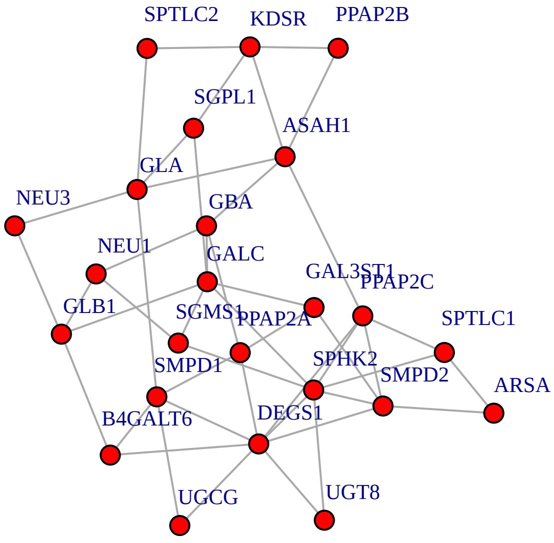

Pathway: KEGG_DRUG_METABOLISM_OTHER_ENZYMES

There are 33 genes in this pathway. This pathway was detected by GSCA

WT p53
Hub Gene (WT): CYP3A5
Weight Factor: 1.248
Hub Gene (MUT): XDH
Weight Factor: 0.967

MUT p53
Hub Gene (MUT): XDH
Weight Factor: 1.337
Hub Gene (WT): CYP3A5
Weight Factor: 1.102

MST2 of the coexpression network for WT p53

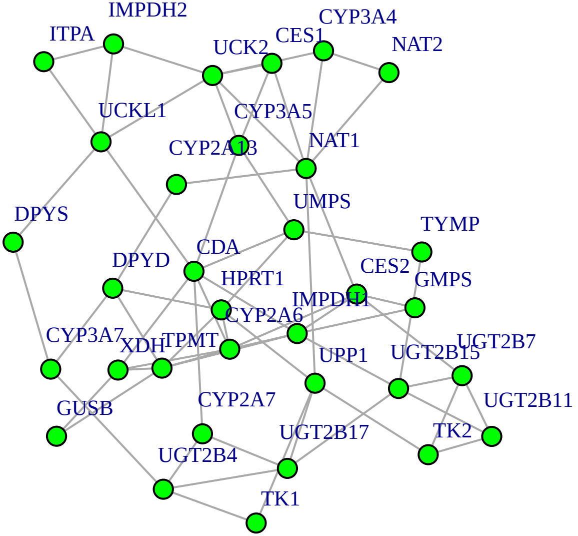

MST2 of the coexpression network for MUT p53

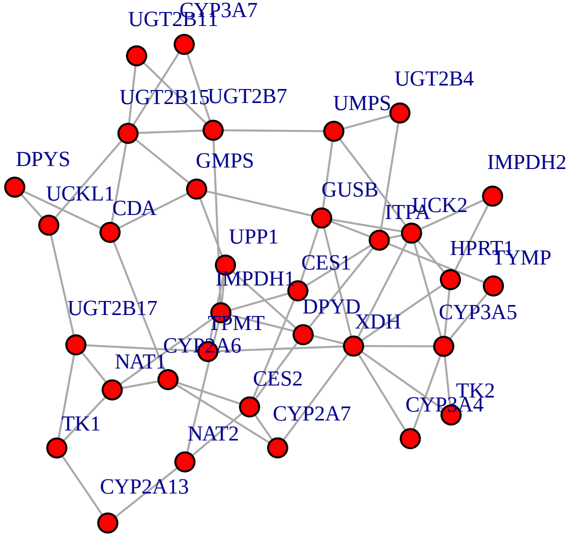

Pathway: KEGG_COLORECTAL_CANCER

There are 58 genes in this pathway. This pathway was detected by GSCA

WT p53	MUT p53
**Hub Gene (WT): PIK3CB**	**Hub Gene (MUT): MLH1**
**Weight Factor: 1.397**	**Weight Factor: 1.428**
**Hub Gene (MUT): MLH1**	**Hub Gene (WT): PIK3CB**
**Weight Factor: 1.338**	**Weight Factor: 0.817**

MST2 of the coexpression network for WT p53

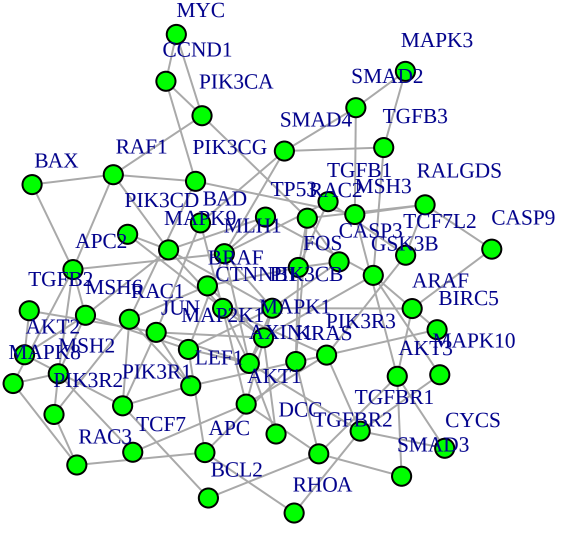

MST2 of the coexpression network for MUT p53

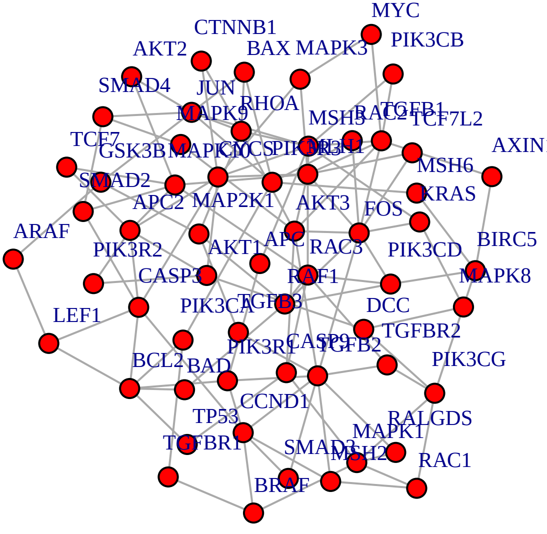

Pathway: BIOCARTA_CELLCYCLE_PATHWAY

There are 23 genes in this pathway. This pathway was detected by GSCA

WT p53
Hub Gene (WT): CDK1
Weight Factor: 1.308
Hub Gene (MUT): CDK1
Weight Factor: 1.308

MUT p53
Hub Gene (MUT): CDK1
Weight Factor: 1.37
Hub Gene (WT): CDK1
Weight Factor: 1.37

MST2 of the coexpression network for WT p53

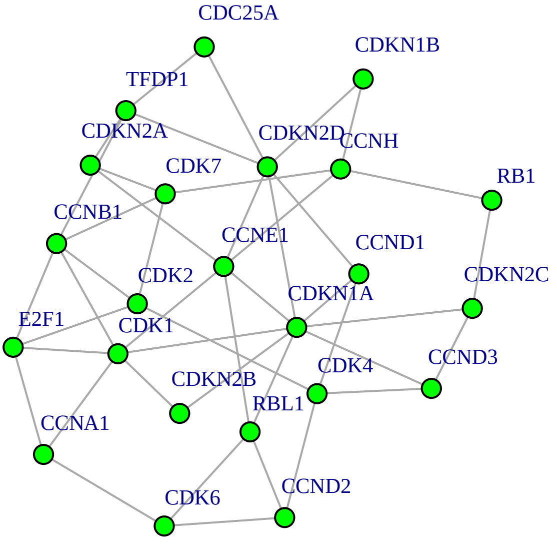

MST2 of the coexpression network for MUT p53

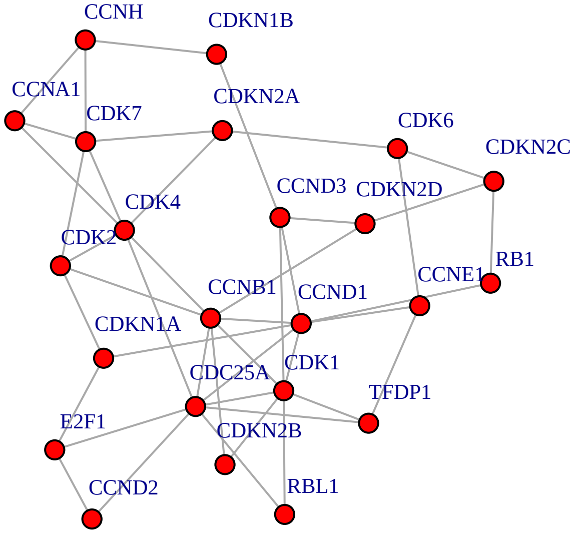

Pathway: BIOCARTA_ERK_PATHWAY

There are 28 genes in this pathway. This pathway was detected by GSCA

WT p53
Hub Gene (WT): HRAS
Weight Factor: 1.243
Hub Gene (MUT): SHC1
Weight Factor: 1.095

MUT p53
Hub Gene (MUT): SHC1
Weight Factor: 1.36
Hub Gene (WT): HRAS
Weight Factor: 1.133

MST2 of the coexpression network for WT p53

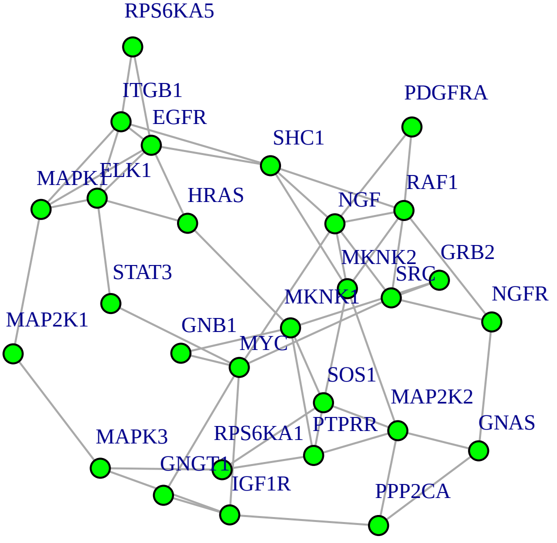

MST2 of the coexpression network for MUT p53

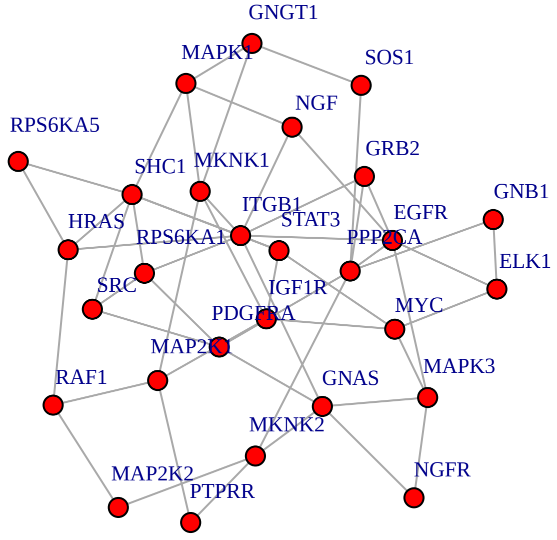

Pathway: BIOCARTA_NGF_PATHWAY

There are 18 genes in this pathway. This pathway was detected by GSCA

WT p53
Hub Gene (WT): PIK3CG
Weight Factor: 1.245
Hub Gene (MUT): HRAS
Weight Factor: 1.084

MUT p53
Hub Gene (MUT): HRAS
Weight Factor: 1.289
Hub Gene (WT): PIK3CG
Weight Factor: 0.881

MST2 of the coexpression network for WT p53

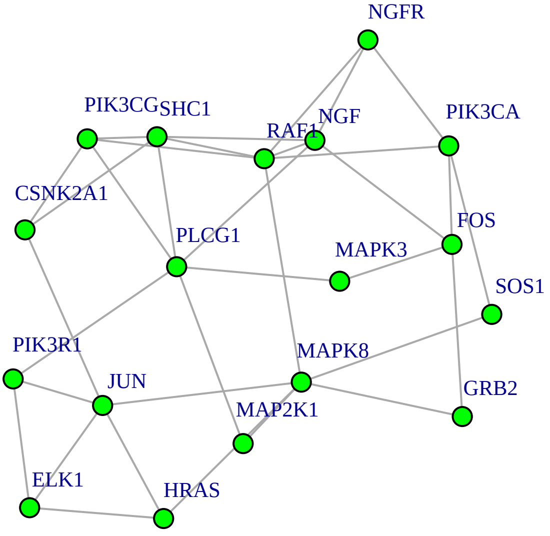

MST2 of the coexpression network for MUT p53

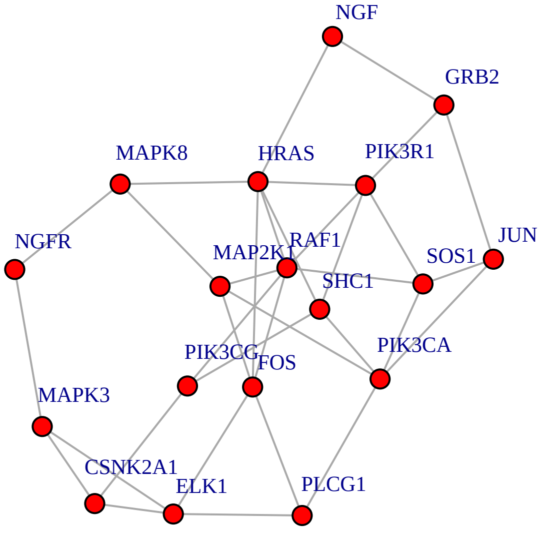

Pathway: BIOCARTA_CDC42RAC_PATHWAY

There are 15 genes in this pathway. This pathway was detected by GSCA

WT p53
Hub Gene (WT): ARPC2
Weight Factor: 1.331
Hub Gene (MUT): ARPC2
Weight Factor: 1.331

MUT p53
Hub Gene (MUT): ARPC2
Weight Factor: 1.329
Hub Gene (WT): ARPC2
Weight Factor: 1.329

MST2 of the coexpression network for WT p53

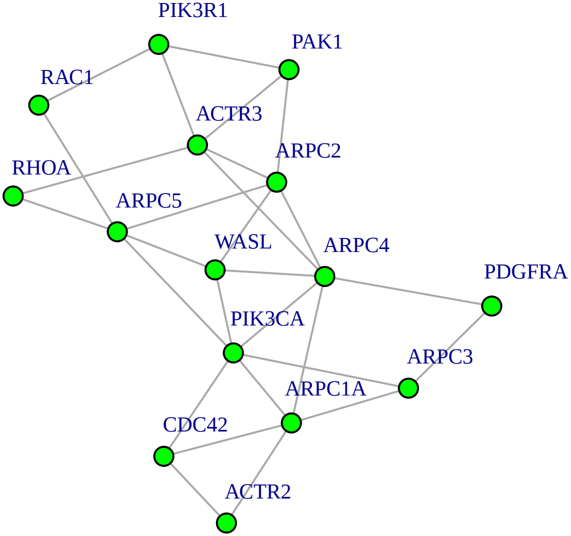

MST2 of the coexpression network for MUT p53

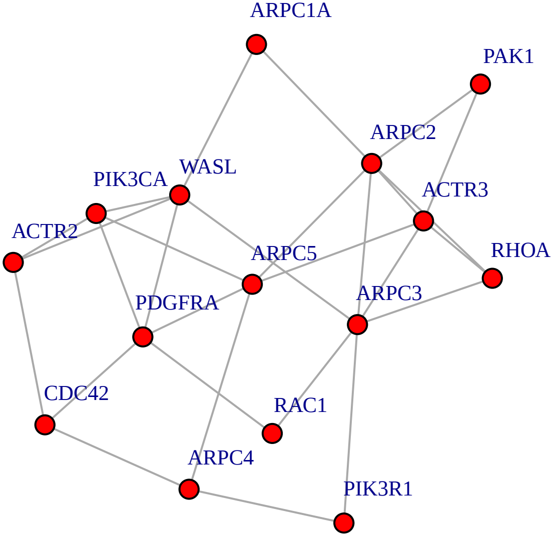

Pathway: BIOCARTA_ACTINY_PATHWAY

There are 19 genes in this pathway. This pathway was detected by GSCA

WT p53
Hub Gene (WT): ARPC4
Weight Factor: 1.334
Hub Gene (MUT): ARPC2
Weight Factor: 1.3

MUT p53
Hub Gene (MUT): ARPC2
Weight Factor: 1.336
Hub Gene (WT): ARPC4
Weight Factor: 0.823

MST2 of the coexpression network for WT p53

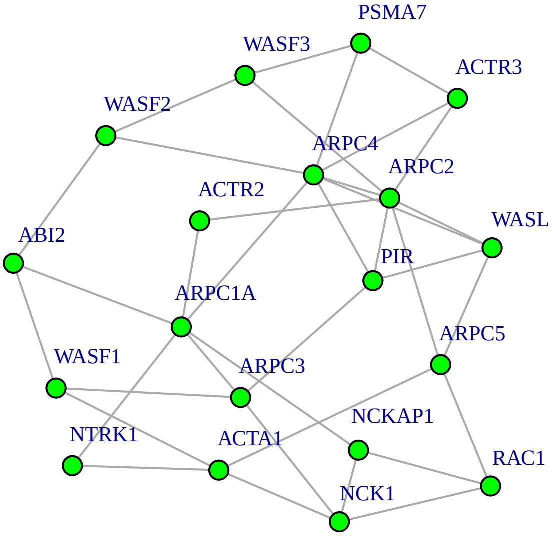

MST2 of the coexpression network for MUT p53

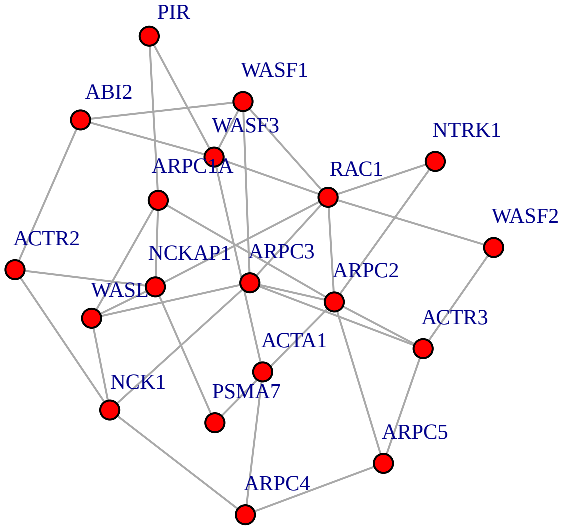

Pathway: REACTOME_GENERIC_TRANSCRIPTION_PATHWAY

There are 22 genes in this pathway. This pathway was detected by GSCA

WT p53
Hub Gene (WT): MAML1
Weight Factor: 1.349
Hub Gene (MUT): MED13
Weight Factor: 1.167

MUT p53
Hub Gene (MUT): MED13
Weight Factor: 1.326
Hub Gene (WT): MAML1
Weight Factor: 1.088

MST2 of the coexpression network for WT p53

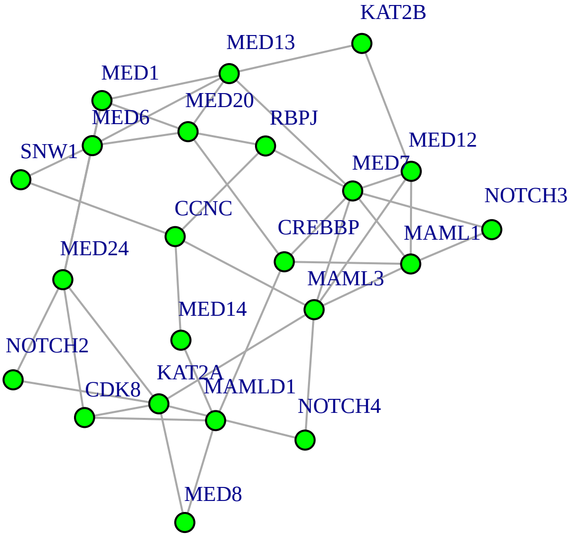

MST2 of the coexpression network for MUT p53

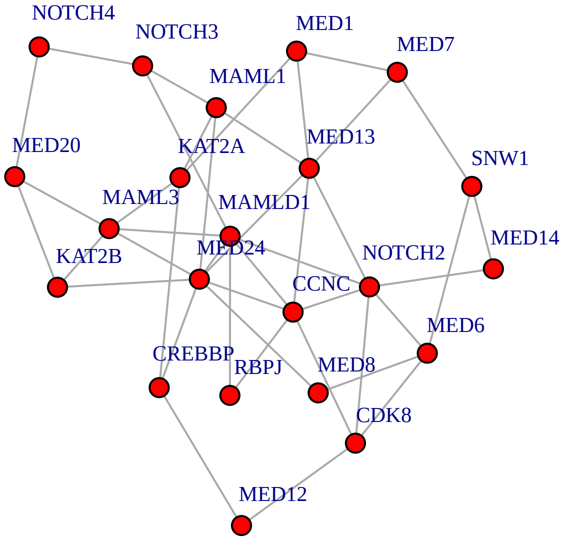

Pathway: REACTOME_MTOR_SIGNALLING

There are 18 genes in this pathway. This pathway was detected by GSCA

WT p53
Hub Gene (WT): EIF4G1
Weight Factor: 1.274
Hub Gene (MUT): AKT2
Weight Factor: 0.597

MUT p53
Hub Gene (MUT): AKT2
Weight Factor: 1.397
Hub Gene (WT): EIF4G1
Weight Factor: 0.769

MST2 of the coexpression network for WT p53

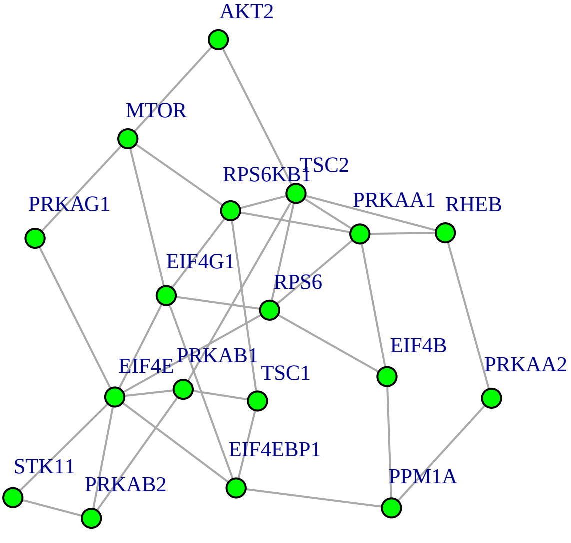

MST2 of the coexpression network for MUT p53

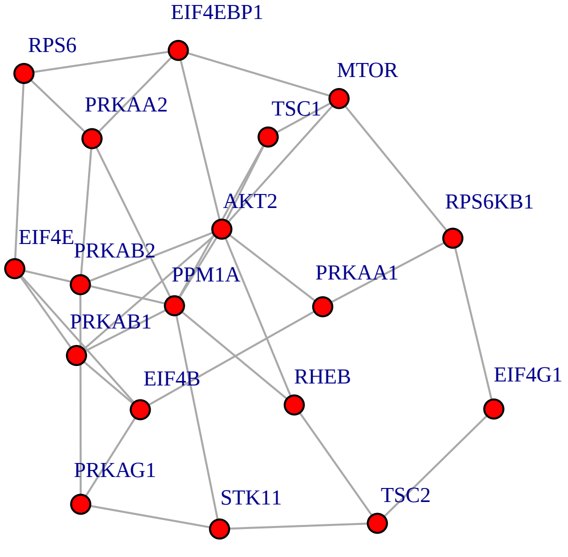

Pathway: REACTOME_RNA_POLYMERASE_III_TRANSCRIPTION

There are 24 genes in this pathway. This pathway was detected by GSCA

WT p53
Hub Gene (WT): SNAPC2
Weight Factor: 1.214
Hub Gene (MUT): ZNF143
Weight Factor: 1.113

MUT p53
Hub Gene (MUT): ZNF143
Weight Factor: 1.305
Hub Gene (WT): SNAPC2
Weight Factor: 0.974

MST2 of the coexpression network for WT p53

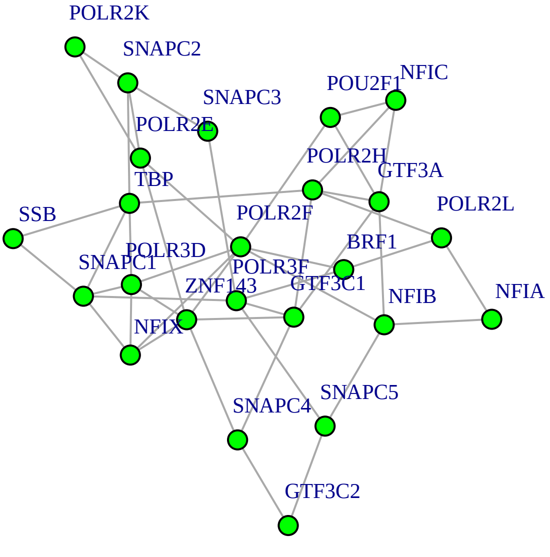

MST2 of the coexpression network for MUT p53

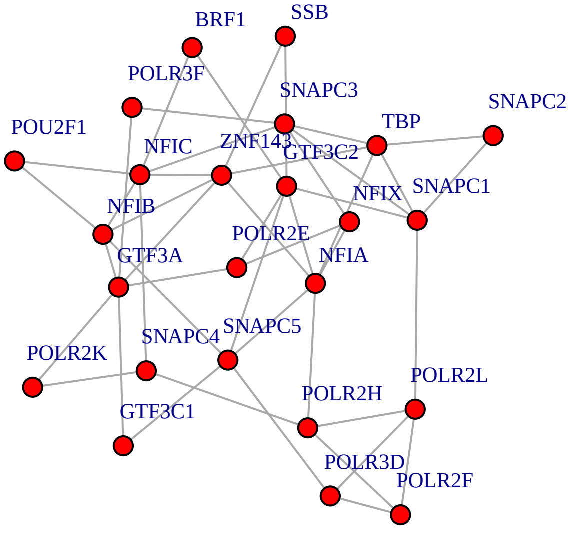

Pathway: REACTOME_RNA_POLYMERASE_III_TRANSCRIPTION_INITIATION

There are 19 genes in this pathway. This pathway was detected by GSCA

WT p53
Hub Gene (WT): TBP
Weight Factor: 1.17
Hub Gene (MUT): TBP
Weight Factor: 1.17

MUT p53
Hub Gene (MUT): TBP
Weight Factor: 1.284
Hub Gene (WT): TBP
Weight Factor: 1.284

MST2 of the coexpression network for WT p53

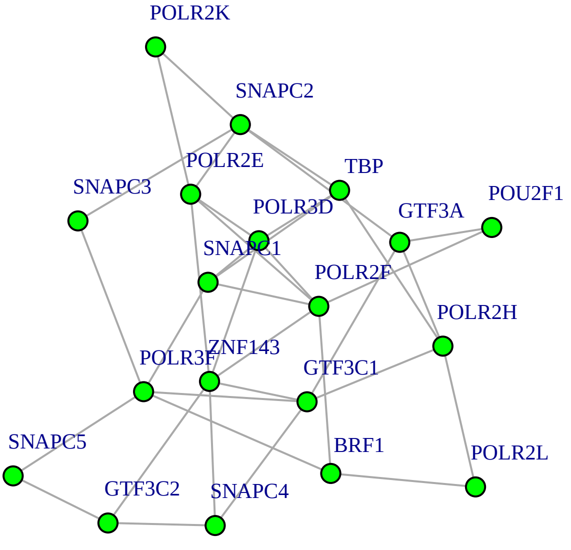

MST2 of the coexpression network for MUT p53

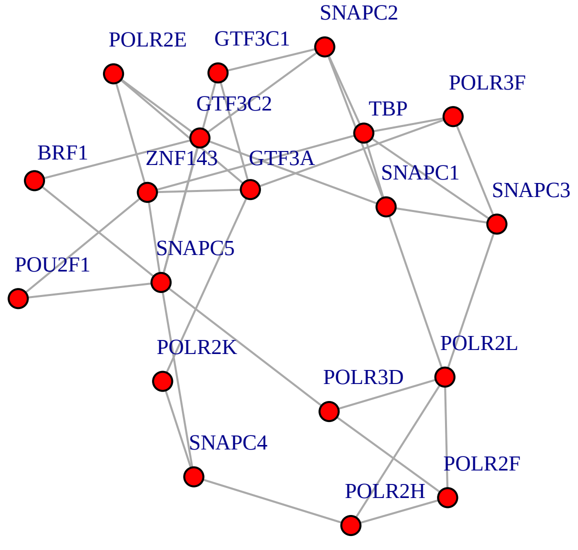

way: REACTOME_RNA_POLYMERASE_III_TRANSCRIPTION_INITIATION_FROM_TYPE_3_PROMO

There are 15 genes in this pathway. This pathway was detected by GSCA

WT p53
Hub Gene (WT): TBP
Weight Factor: 1.213
Hub Gene (MUT): TBP
Weight Factor: 1.213

MUT p53
Hub Gene (MUT): TBP
Weight Factor: 1.347
Hub Gene (WT): TBP
Weight Factor: 1.347

MST2 of the coexpression network for WT p53

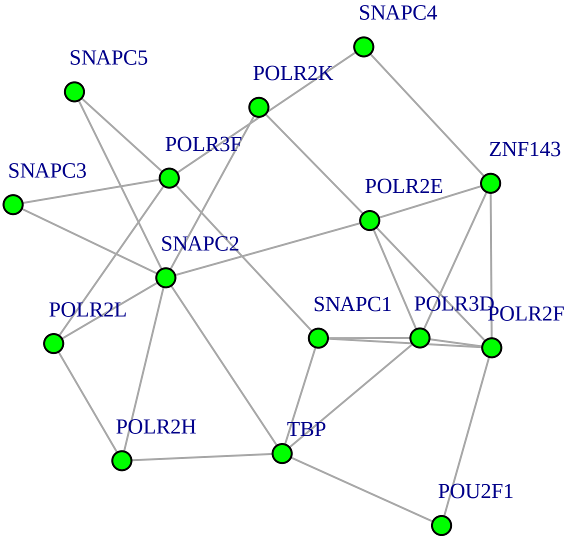

MST2 of the coexpression network for MUT p53

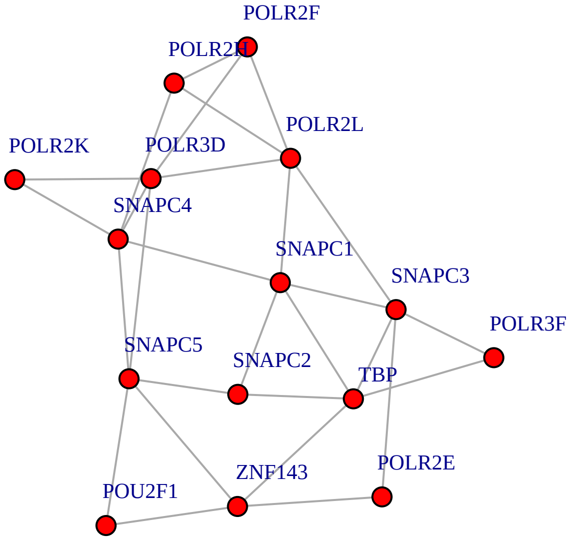

Pathway: REACTOME_SIGNALING_BY_BMP

There are 20 genes in this pathway. This pathway was detected by GSCA

WT p53
Hub Gene (WT): ZFYVE16
Weight Factor: 1.263
Hub Gene (MUT): UBE2D3
Weight Factor: 1.123

MUT p53
Hub Gene (MUT): UBE2D3
Weight Factor: 1.388
Hub Gene (WT): ZFYVE16
Weight Factor: 0.892

MST2 of the coexpression network for WT p53

MST2 of the coexpression network for MUT p53

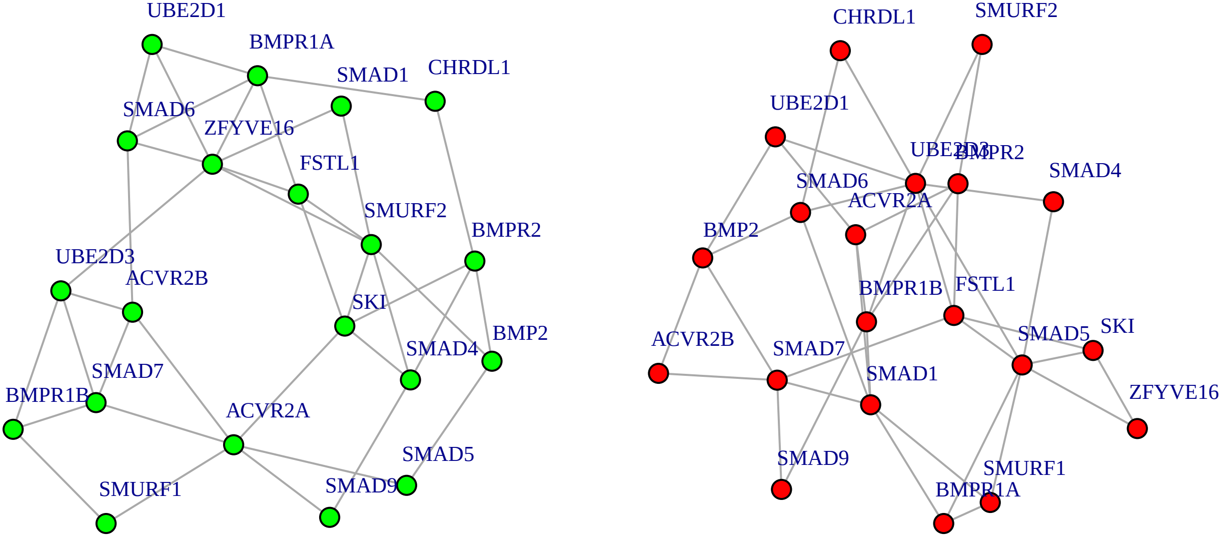

Pathway: REACTOME_GAP_JUNCTION_TRAFFICKING

There are 18 genes in this pathway. This pathway was detected by GSCA

WT p53
Hub Gene (WT): DNM1
Weight Factor: 1.209
Hub Gene (MUT): GJA1
Weight Factor: 1.208

MUT p53
Hub Gene (MUT): GJA1
Weight Factor: 1.409
Hub Gene (WT): DNM1
Weight Factor: 0.792

MST2 of the coexpression network for WT p53

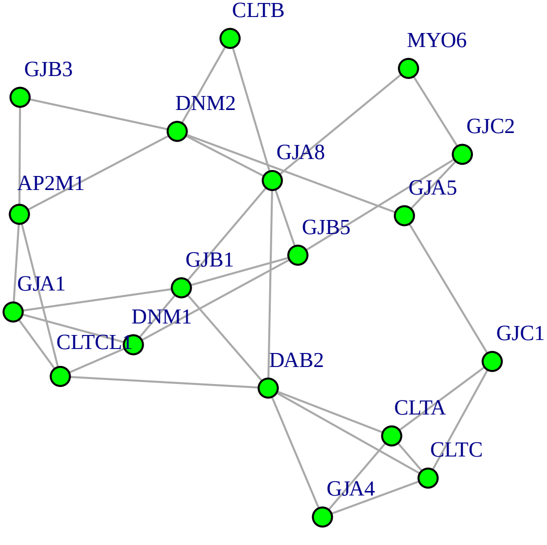

MST2 of the coexpression network for MUT p53

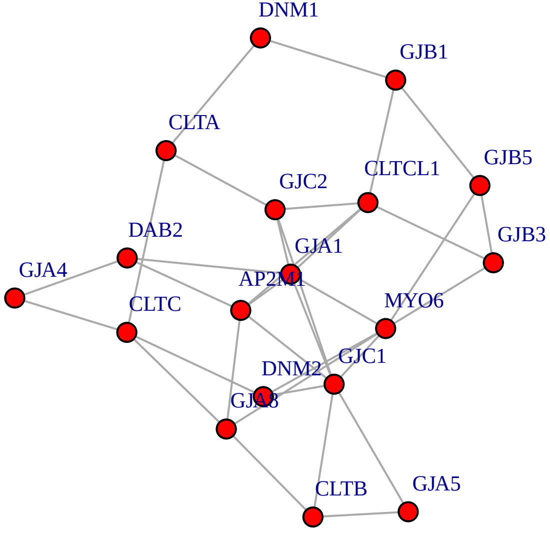

Pathway: SIG_REGULATION_OF_THE_ACTIN_CYTOSKELETON_BY_RHO_GTPASES

There are 31 genes in this pathway. This pathway was detected by GSCA

WT p53
Hub Gene (WT): PFN1
Weight Factor: 1.274
Hub Gene (MUT): MYLK
Weight Factor: 1.26

MUT p53
Hub Gene (MUT): MYLK
Weight Factor: 1.637
Hub Gene (WT): PFN1
Weight Factor: 1.008

MST2 of the coexpression network for WT p53

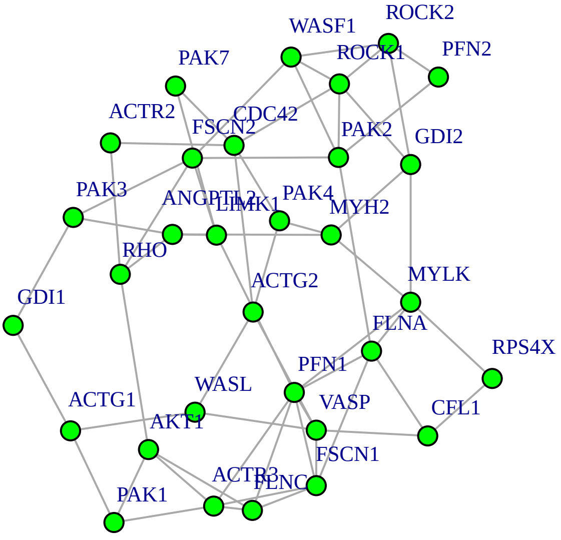

MST2 of the coexpression network for MUT p53

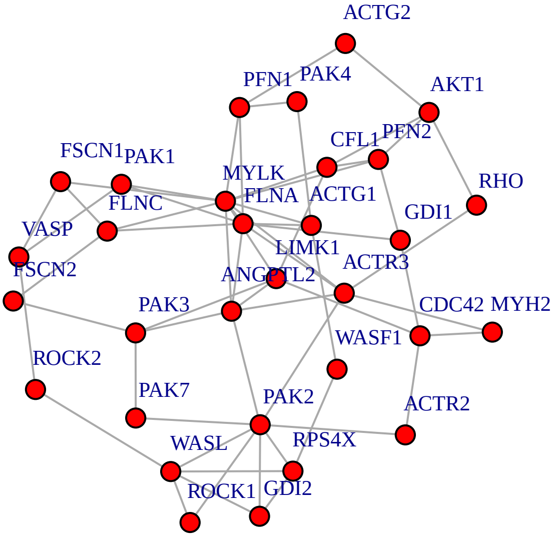

Pathway: ST_FAS_SIGNALING_PATHWAY

There are 51 genes in this pathway. This pathway was detected by GSCA

WT p53
Hub Gene (WT): MAP2K4
Weight Factor: 1.365
Hub Gene (MUT): PFN2
Weight Factor: 0.787

MUT p53
Hub Gene (MUT): PFN2
Weight Factor: 1.339
Hub Gene (WT): MAP2K4
Weight Factor: 0.856

MST2 of the coexpression network for WT p53

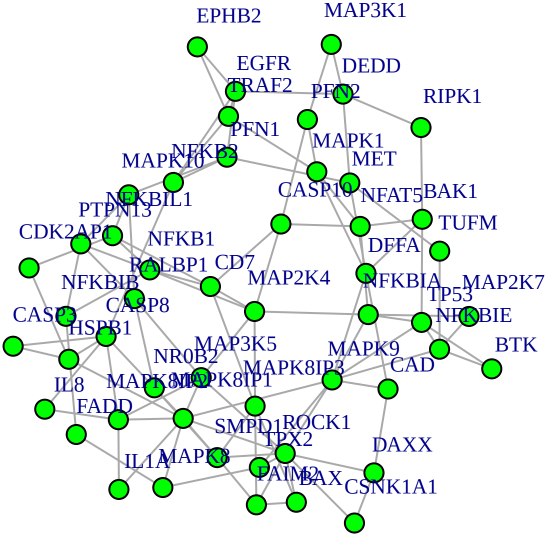

MST2 of the coexpression network for MUT p53

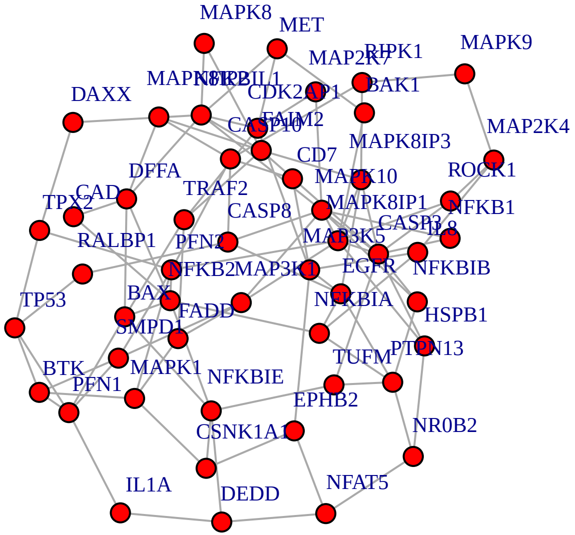

Supplement: Supplementary Data [file supp_btt687_Supplementary_Document_1.pdf]
