# Supplementary material for: Gene Sets Net Correlations Analysis (GSNCA): a multivariate differential coexpression test for gene sets
Source: Bioinformatics. 2013 Nov 30;30(3):360–8. doi: 10.1093/bioinformatics/btt687 (PMC4023302; doi:10.1093/bioinformatics/btt687)

Pathway: FRASOR_TAMOXIFEN_RESPONSE_UP

There are 43 genes in this pathway. This pathway was detected by Both

BCR/ABL ALL
Major Gene (BCR/ABL): KLC1
Weight Factor: 1.253
Major Gene (NEG): WNT4
Weight Factor: 1.21

NEG ALL
Major Gene (NEG): WNT4
Weight Factor: 1.389
Major Gene (BCR/ABL): KLC1
Weight Factor: 1.126

MST2 of the coexpression network for BCR/ABL ALL

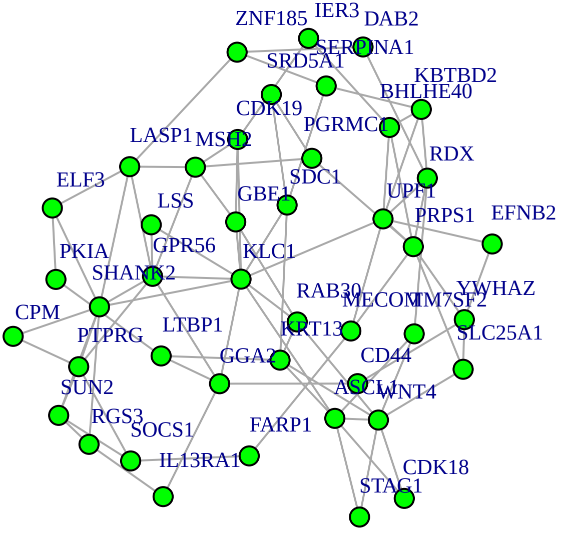

MST2 of the coexpression network for NEG ALL

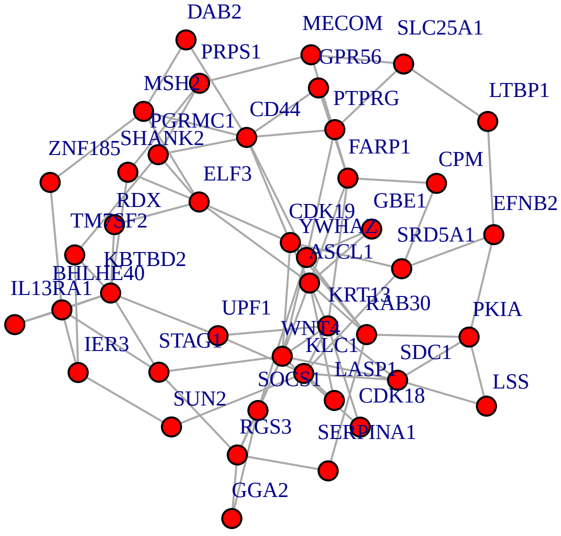

Pathway: CHANDRAN_METASTASIS_TOP50_UP

There are 15 genes in this pathway. This pathway was detected by Both

BCR/ABL ALL
Major Gene (BCR/ABL): EIF1AX
Weight Factor: 1.375
Major Gene (NEG): HSP90AA1
Weight Factor: 1.218

MST2 of the coexpression network for BCR/ABL ALL

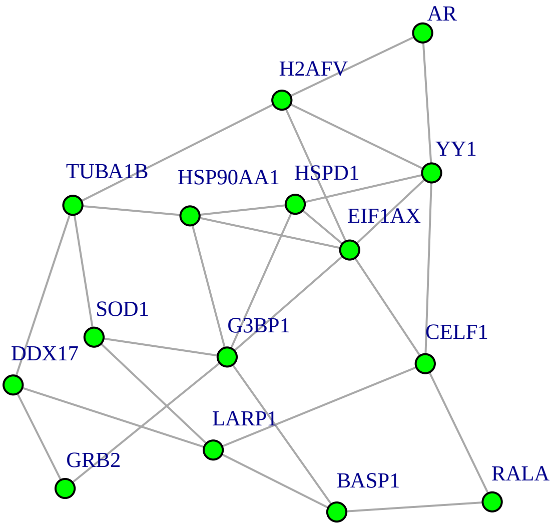

NEG ALL
Major Gene (NEG): HSP90AA1
Weight Factor: 1.312
Major Gene (BCR/ABL): EIF1AX
Weight Factor: 1.192

MST2 of the coexpression network for NEG ALL

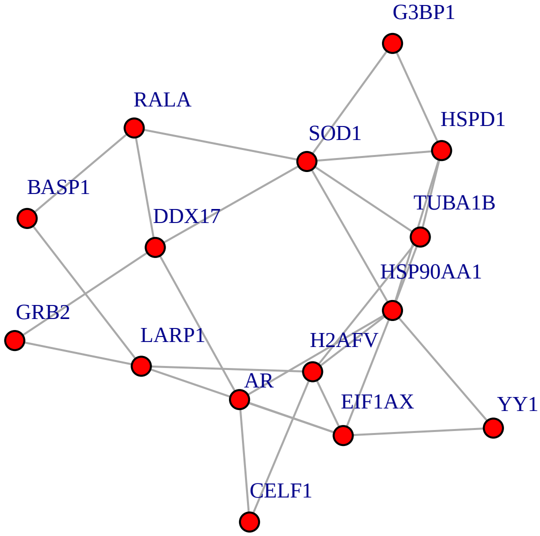

Pathway: ROY_WOUND_BLOOD_VESSEL_UP

There are 46 genes in this pathway. This pathway was detected by Both

BCR/ABL ALL
Major Gene (BCR/ABL): ANGPTL2
Weight Factor: 1.39
Major Gene (NEG): SLCO2A1
Weight Factor: 1.273

NEG ALL
Major Gene (NEG): SLCO2A1
Weight Factor: 1.449
Major Gene (BCR/ABL): ANGPTL2
Weight Factor: 1.202

MST2 of the coexpression network for BCR/ABL ALL

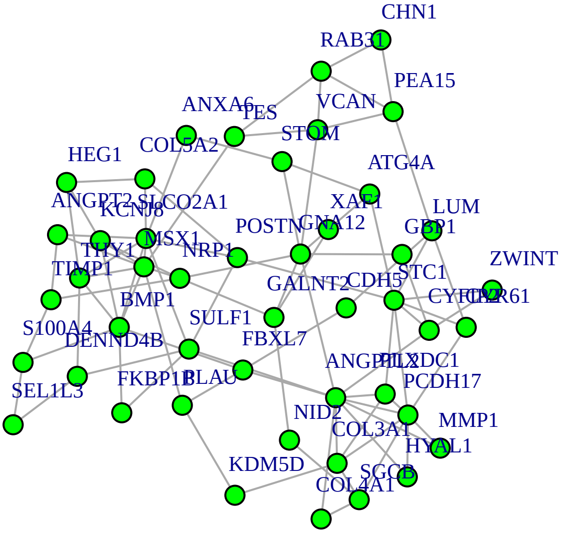

MST2 of the coexpression network for NEG ALL

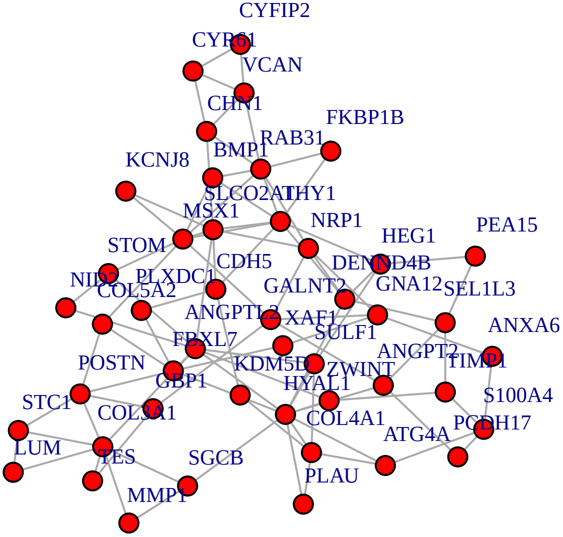

Pathway: BILBAN_B_CLL_LPL_DN

There are 31 genes in this pathway. This pathway was detected by Both

BCR/ABL ALL
Major Gene (BCR/ABL): RGS2
Weight Factor: 1.451
Major Gene (NEG): JUNB
Weight Factor: 0.952

NEG ALL
Major Gene (NEG): JUNB
Weight Factor: 1.398
Major Gene (BCR/ABL): RGS2
Weight Factor: 0.874

MST2 of the coexpression network for BCR/ABL ALL

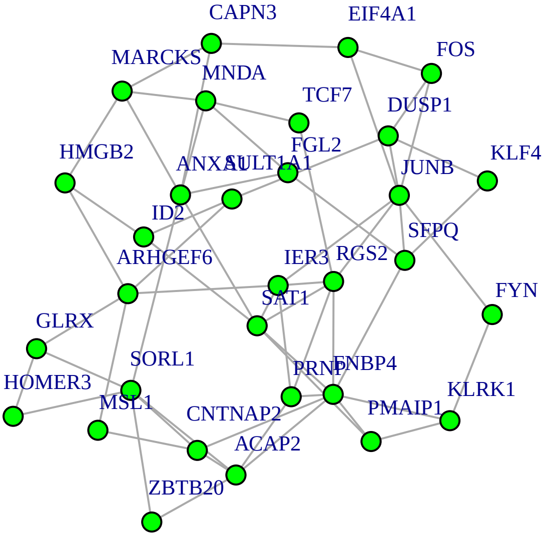

MST2 of the coexpression network for NEG ALL

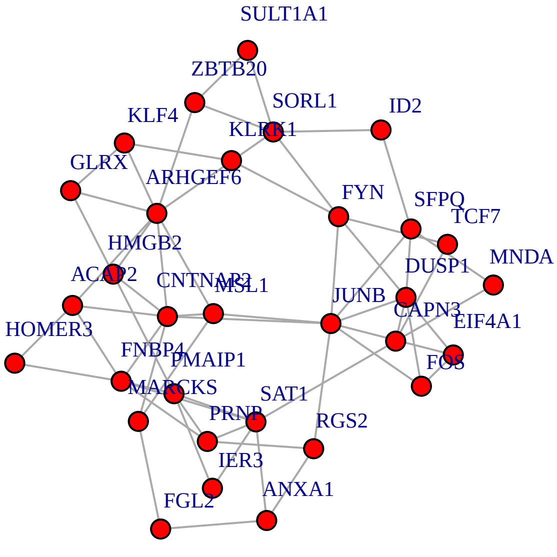

Pathway: FARMER_BREAST_CANCER_CLUSTER_1

There are 35 genes in this pathway. This pathway was detected by Both

BCR/ABL ALL
Major Gene (BCR/ABL): CD3D
Weight Factor: 1.471
Major Gene (NEG): IFI44L
Weight Factor: 1.186

NEG ALL
Major Gene (NEG): IFI44L
Weight Factor: 1.472
Major Gene (BCR/ABL): CD3D
Weight Factor: 0.77

MST2 of the coexpression network for BCR/ABL ALL

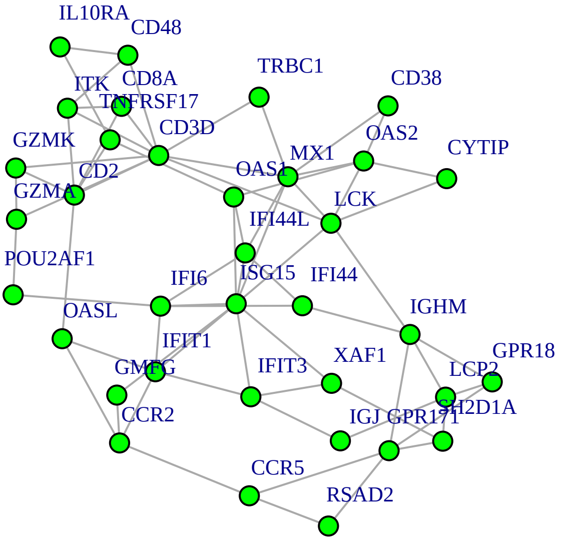

MST2 of the coexpression network for NEG ALL

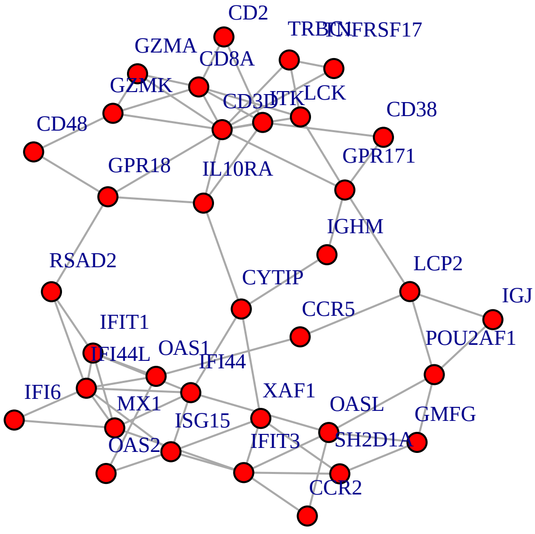

Pathway: EINAV_INTERFERON_SIGNATURE_IN_CANCER

There are 27 genes in this pathway. This pathway was detected by Both

BCR/ABL ALL
Major Gene (BCR/ABL): ISG15
Weight Factor: 1.383
Major Gene (NEG): MX1
Weight Factor: 1.335

NEG ALL
Major Gene (NEG): MX1
Weight Factor: 1.3
Major Gene (BCR/ABL): ISG15
Weight Factor: 1.282

MST2 of the coexpression network for BCR/ABL ALL

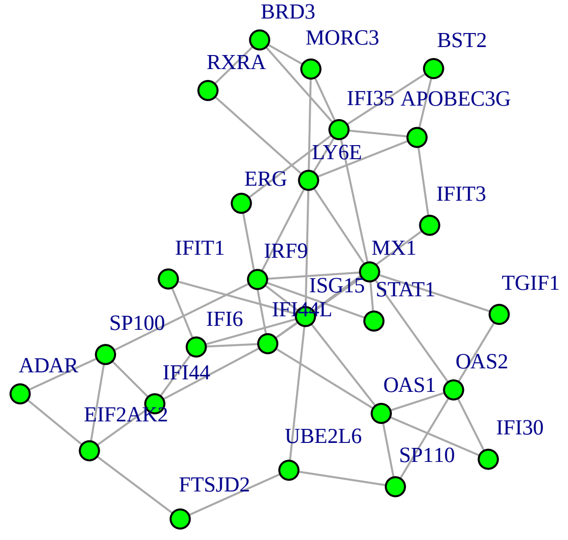

MST2 of the coexpression network for NEG ALL

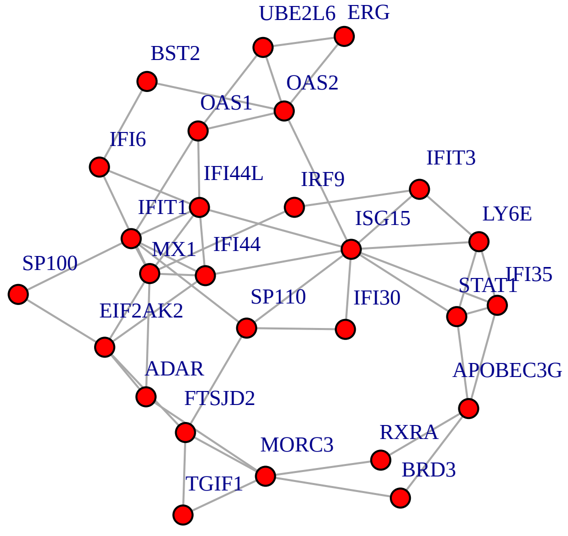

Pathway: LIANG_HEMATOPOIESIS_STEM_CELL_NUMBER_LARGE_VS_TINY_UP

There are 27 genes in this pathway. This pathway was detected by Both

BCR/ABL ALL
Major Gene (BCR/ABL): MANF
Weight Factor: 1.404
Major Gene (NEG): GCAT
Weight Factor: 0.662

NEG ALL
Major Gene (NEG): GCAT
Weight Factor: 1.235
Major Gene (BCR/ABL): MANF
Weight Factor: 1.119

MST2 of the coexpression network for BCR/ABL ALL

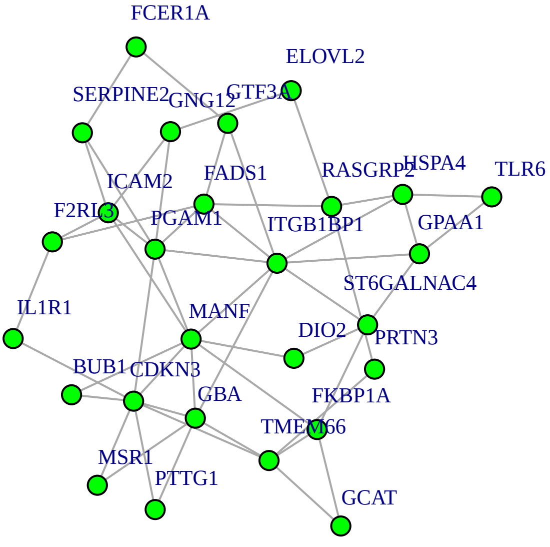

MST2 of the coexpression network for NEG ALL

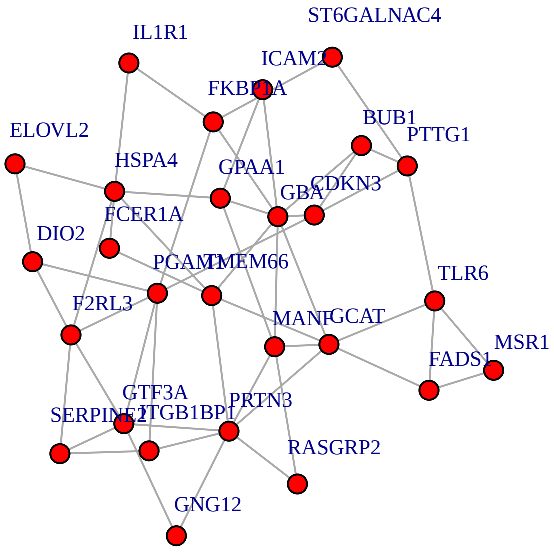

Pathway: MORI_SMALL_PRE_BII_LYMPHOCYTE_DN

There are 47 genes in this pathway. This pathway was detected by Both

BCR/ABL ALL
Major Gene (BCR/ABL): SMC4
Weight Factor: 1.28
Major Gene (NEG): XPO1
Weight Factor: 0.985

NEG ALL
Major Gene (NEG): XPO1
Weight Factor: 1.467
Major Gene (BCR/ABL): SMC4
Weight Factor: 1.351

MST2 of the coexpression network for BCR/ABL ALL

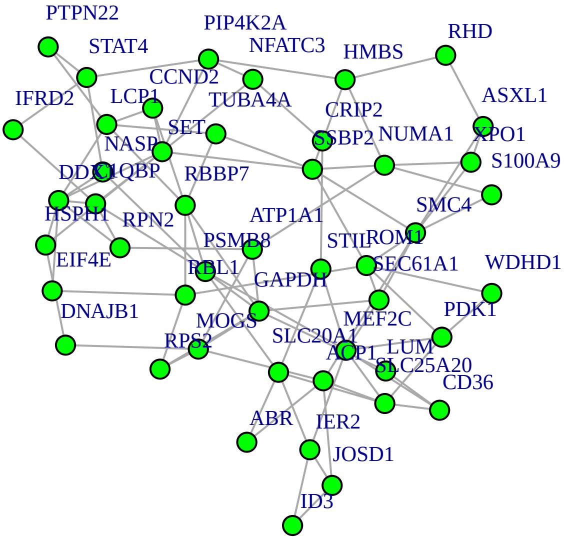

MST2 of the coexpression network for NEG ALL

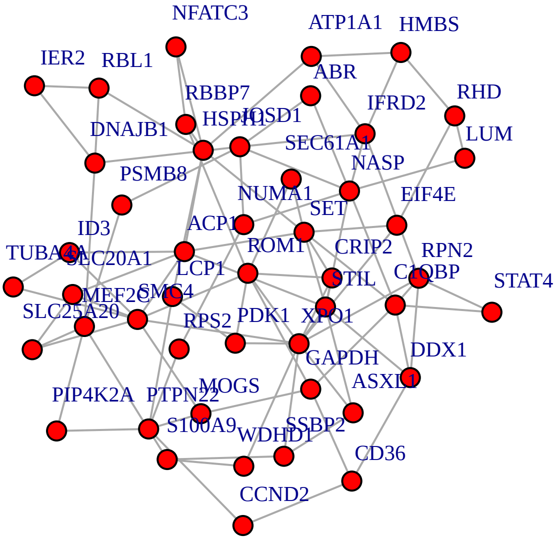

Pathway: MORI_MATURE_B_LYMPHOCYTE_DN

There are 45 genes in this pathway. This pathway was detected by Both

BCR/ABL ALL
Major Gene (BCR/ABL): CDKN3
Weight Factor: 1.481
Major Gene (NEG): CKS1B
Weight Factor: 1.097

NEG ALL
Major Gene (NEG): CKS1B
Weight Factor: 1.389
Major Gene (BCR/ABL): CDKN3
Weight Factor: 1.378

MST2 of the coexpression network for BCR/ABL ALL

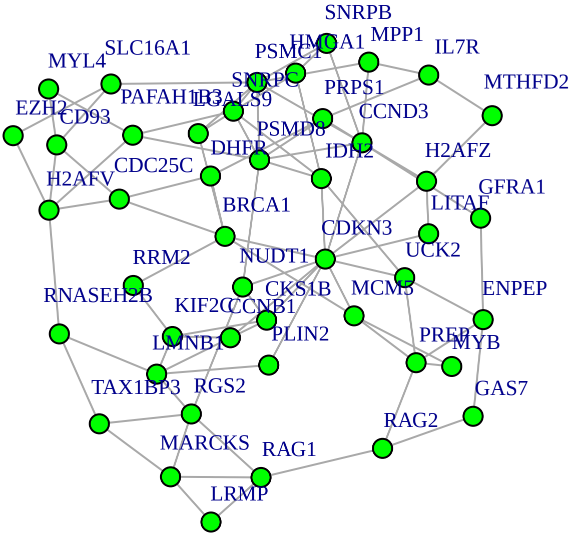

MST2 of the coexpression network for NEG ALL

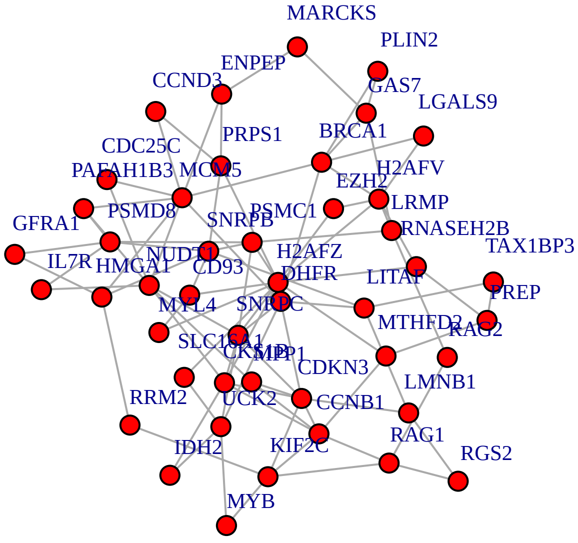

Pathway: YAGI_AML_RELAPSE_PROGNOSIS

There are 35 genes in this pathway. This pathway was detected by Both

BCR/ABL ALL
Major Gene (BCR/ABL): ECE2
Weight Factor: 1.39
Major Gene (NEG): TUG1
Weight Factor: 1.336

NEG ALL
Major Gene (NEG): TUG1
Weight Factor: 1.475
Major Gene (BCR/ABL): ECE2
Weight Factor: 1.416

MST2 of the coexpression network for BCR/ABL ALL

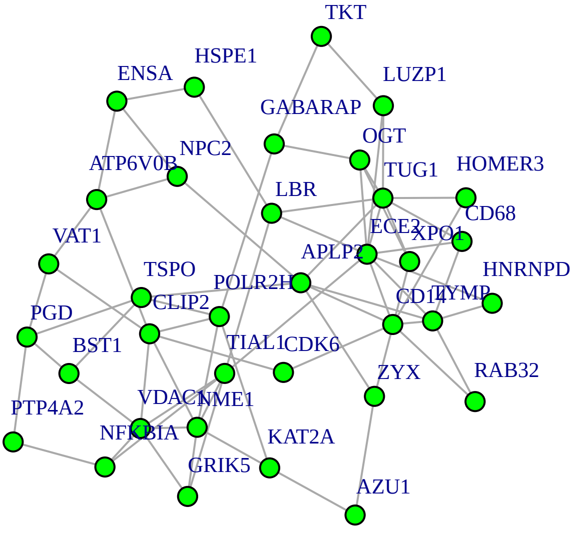

MST2 of the coexpression network for NEG ALL

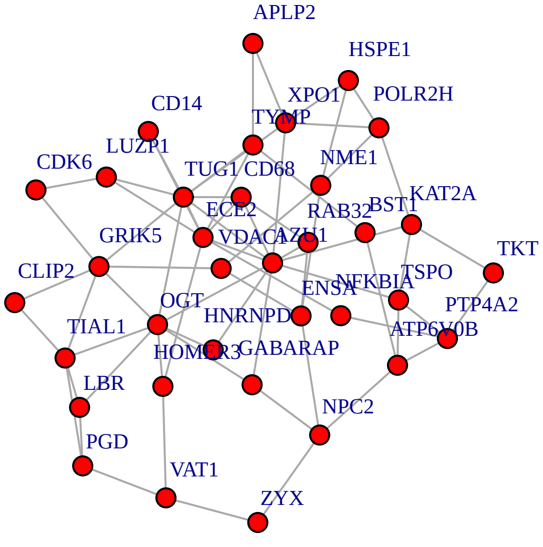

Pathway: HALMOS_CEBPA_TARGETS_UP

There are 41 genes in this pathway. This pathway was detected by Both

BCR/ABL ALL
Major Gene (BCR/ABL): IL8
Weight Factor: 1.393
Major Gene (NEG): FOXA2
Weight Factor: 1.023

NEG ALL
Major Gene (NEG): FOXA2
Weight Factor: 1.532
Major Gene (BCR/ABL): IL8
Weight Factor: 0.9

MST2 of the coexpression network for BCR/ABL ALL

MST2 of the coexpression network for NEG ALL

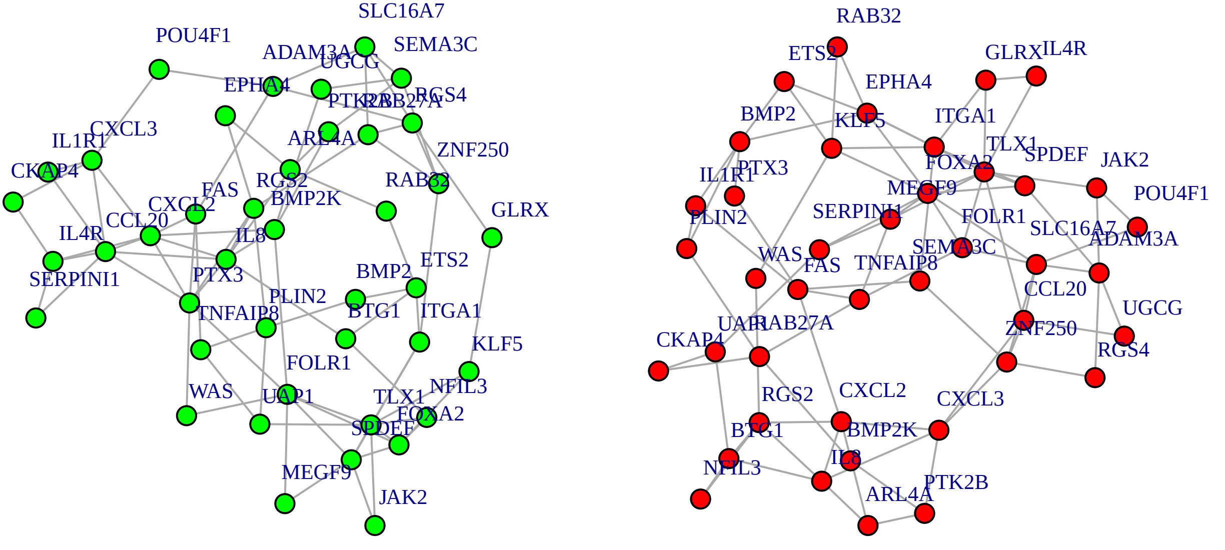

Pathway: KIM_LRRC3B_TARGETS

There are 26 genes in this pathway. This pathway was detected by Both

BCR/ABL ALL
Major Gene (BCR/ABL): IFI27
Weight Factor: 1.316
Major Gene (NEG): ISG15
Weight Factor: 1.31

NEG ALL
Major Gene (NEG): ISG15
Weight Factor: 1.426
Major Gene (BCR/ABL): IFI27
Weight Factor: 0.787

MST2 of the coexpression network for BCR/ABL ALL

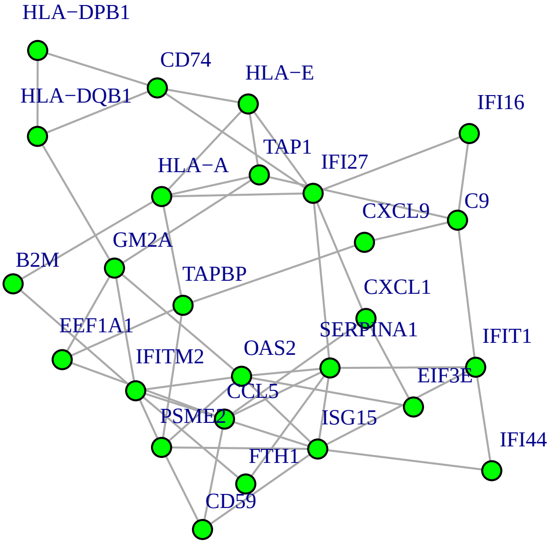

MST2 of the coexpression network for NEG ALL

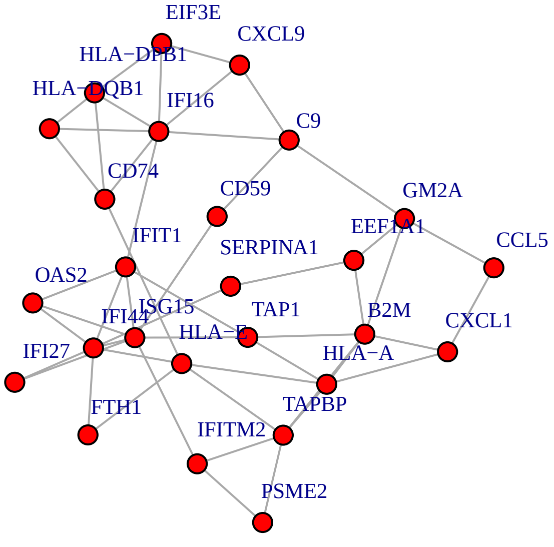

Pathway: AMUNDSON_POOR_SURVIVAL_AFTER_GAMMA_RADIATION_8G

There are 71 genes in this pathway. This pathway was detected by Both

BCR/ABL ALL
Major Gene (BCR/ABL): IL2RG
Weight Factor: 1.532
Major Gene (NEG): SYDE1
Weight Factor: 1.047

NEG ALL
Major Gene (NEG): SYDE1
Weight Factor: 1.526
Major Gene (BCR/ABL): IL2RG
Weight Factor: 0.988

MST2 of the coexpression network for BCR/ABL ALL

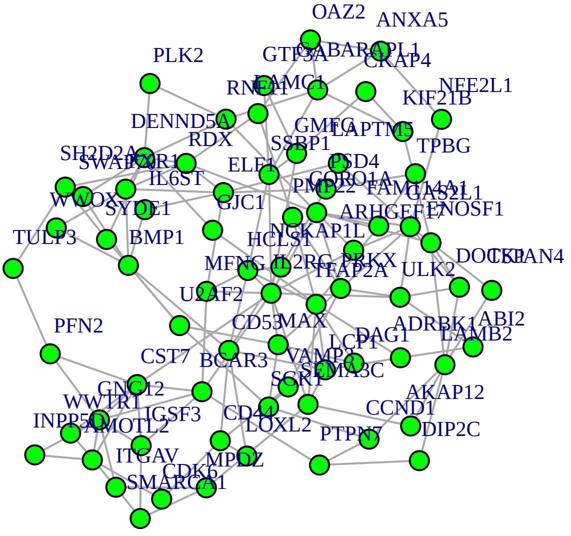

MST2 of the coexpression network for NEG ALL

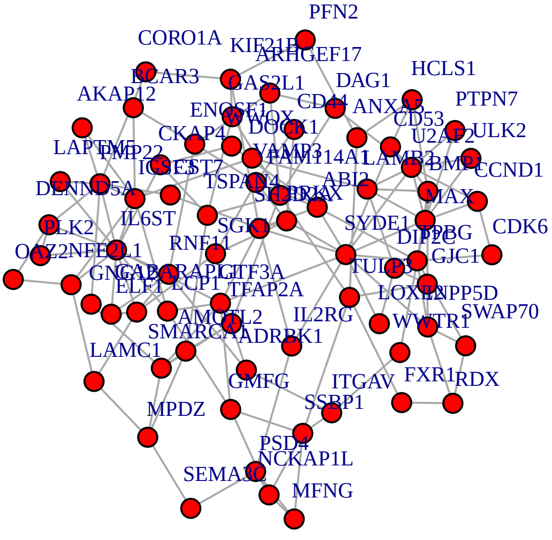

Pathway: CHUNG_BLISTER_CYTOTOXICITY_UP

There are 75 genes in this pathway. This pathway was detected by Both

BCR/ABL ALL
Major Gene (BCR/ABL): ATOX1
Weight Factor: 1.435
Major Gene (NEG): SOD1
Weight Factor: 1.429

NEG ALL
Major Gene (NEG): SOD1
Weight Factor: 1.392
Major Gene (BCR/ABL): ATOX1
Weight Factor: 1.176

MST2 of the coexpression network for BCR/ABL ALL

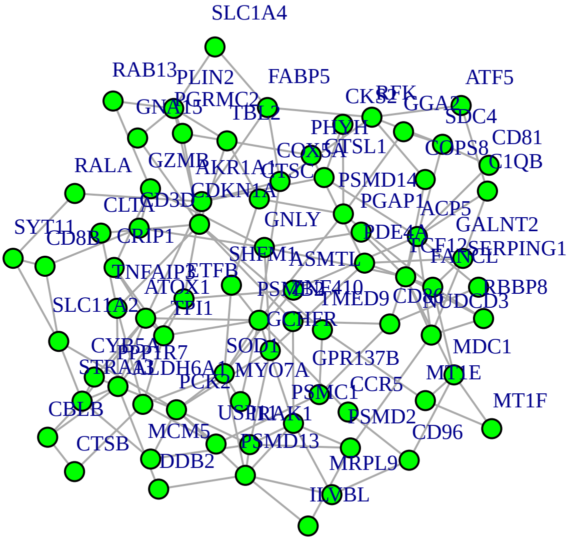

MST2 of the coexpression network for NEG ALL

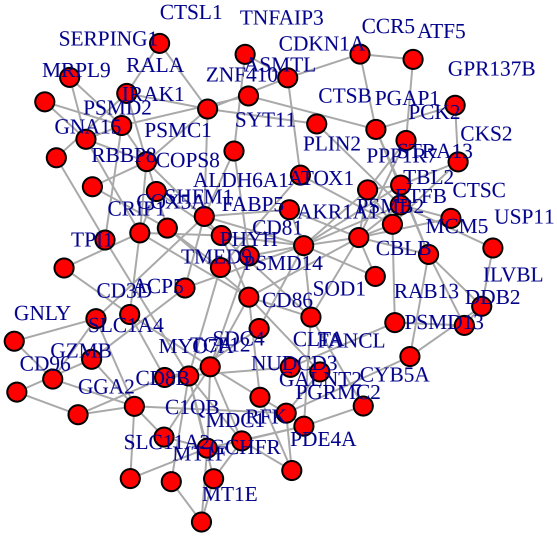

Pathway: BHATI_G2M_ARREST_BY_2METHOXYESTRADIOL_UP

There are 72 genes in this pathway. This pathway was detected by Both

BCR/ABL ALL
Major Gene (BCR/ABL): LRP3
Weight Factor: 1.418
Major Gene (NEG): PHIP
Weight Factor: 1.279

NEG ALL
Major Gene (NEG): PHIP
Weight Factor: 1.482
Major Gene (BCR/ABL): LRP3
Weight Factor: 1.321

MST2 of the coexpression network for BCR/ABL ALL

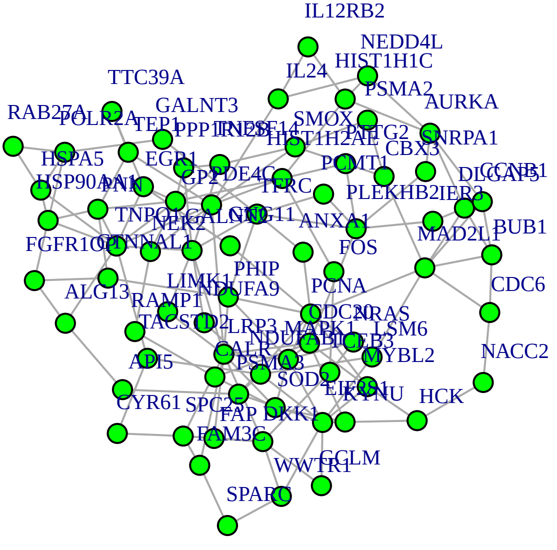

MST2 of the coexpression network for NEG ALL

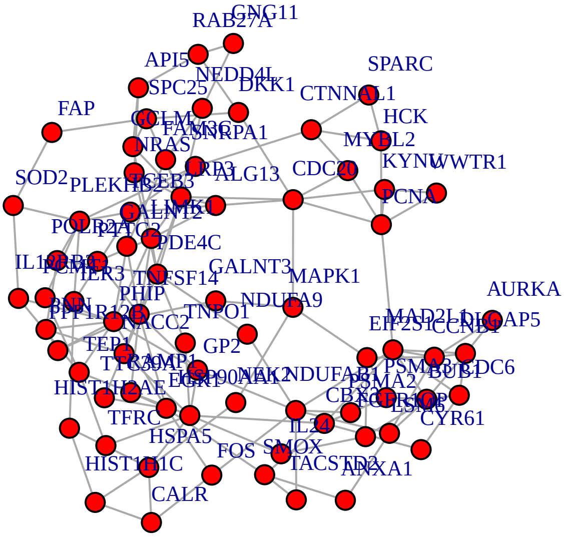

Pathway: PARK_APL_PATHOGENESIS_DN

There are 37 genes in this pathway. This pathway was detected by Both

BCR/ABL ALL
Major Gene (BCR/ABL): SPI1
Weight Factor: 1.388
Major Gene (NEG): PRTN3
Weight Factor: 1.013

NEG ALL
Major Gene (NEG): PRTN3
Weight Factor: 1.46
Major Gene (BCR/ABL): SPI1
Weight Factor: 1.432

MST2 of the coexpression network for BCR/ABL ALL

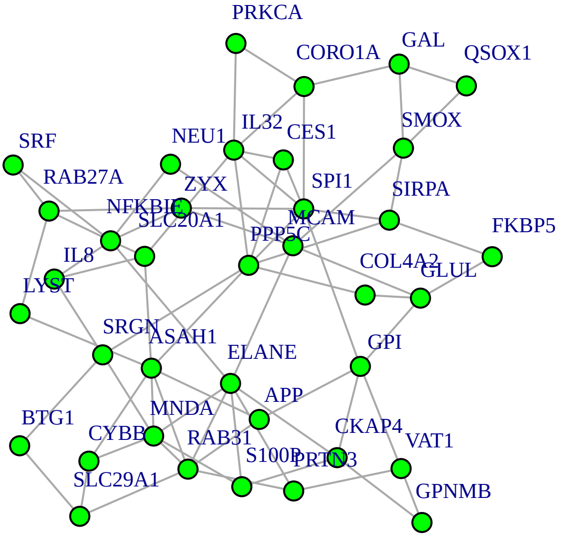

MST2 of the coexpression network for NEG ALL

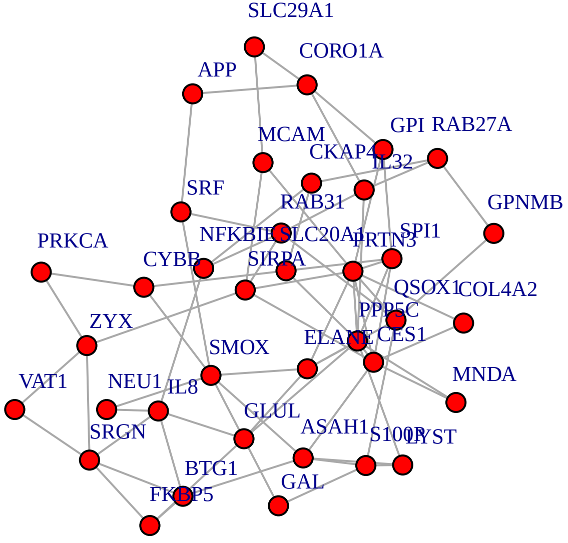

Pathway: HOFFMANN_SMALL_PRE_BII_TO_IMMATURE_B_LYMPHOCYTE_UP

There are 32 genes in this pathway. This pathway was detected by Both

BCR/ABL ALL
Major Gene (BCR/ABL): NCF4
Weight Factor: 1.459
Major Gene (NEG): XPO1
Weight Factor: 0.718

NEG ALL
Major Gene (NEG): XPO1
Weight Factor: 1.428
Major Gene (BCR/ABL): NCF4
Weight Factor: 1.064

MST2 of the coexpression network for BCR/ABL ALL

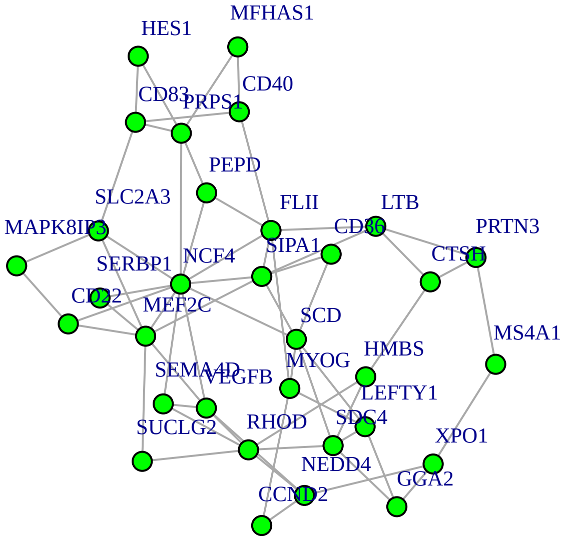

MST2 of the coexpression network for NEG ALL

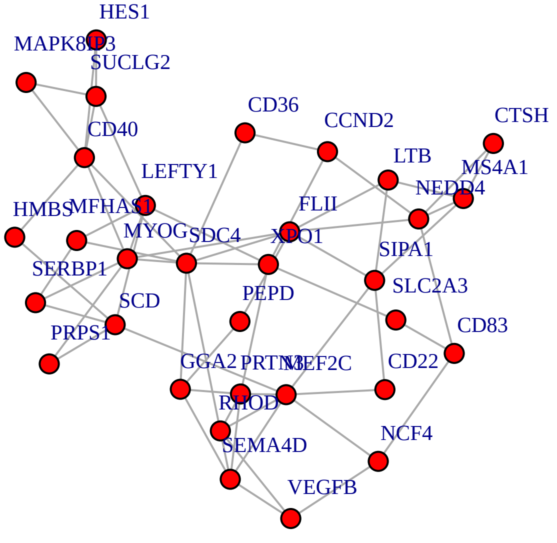

Pathway: VALK_AML_WITH_FLT3_ITD

There are 31 genes in this pathway. This pathway was detected by Both

BCR/ABL ALL
Major Gene (BCR/ABL): IL1RAP
Weight Factor: 1.335
Major Gene (NEG): HOXB3
Weight Factor: 1.118

NEG ALL
Major Gene (NEG): HOXB3
Weight Factor: 1.364
Major Gene (BCR/ABL): IL1RAP
Weight Factor: 0.745

MST2 of the coexpression network for BCR/ABL ALL

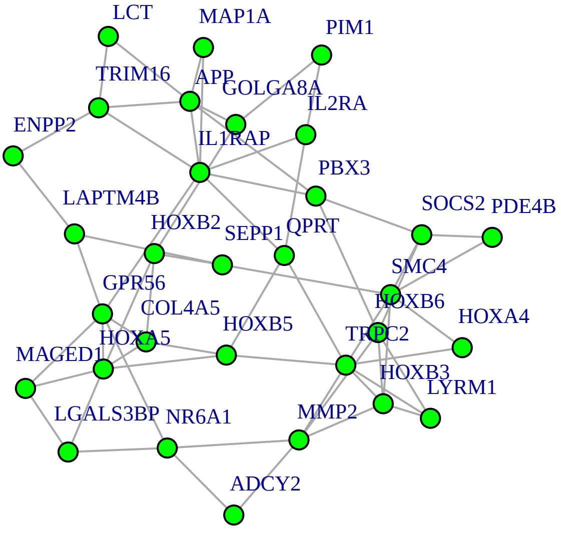

MST2 of the coexpression network for NEG ALL

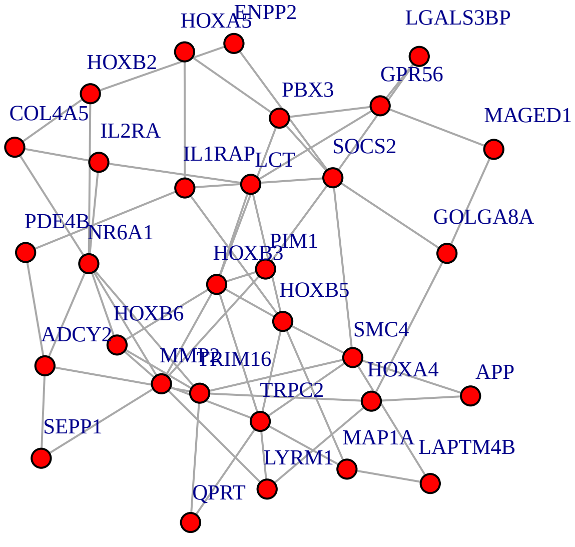

Pathway: HAHTOLA_CTCL_CUTANEOUS

There are 22 genes in this pathway. This pathway was detected by Both

BCR/ABL ALL
Major Gene (BCR/ABL): PSMB3
Weight Factor: 1.326
Major Gene (NEG): MMP9
Weight Factor: 0.979

NEG ALL
Major Gene (NEG): MMP9
Weight Factor: 1.478
Major Gene (BCR/ABL): PSMB3
Weight Factor: 0.986

MST2 of the coexpression network for BCR/ABL ALL

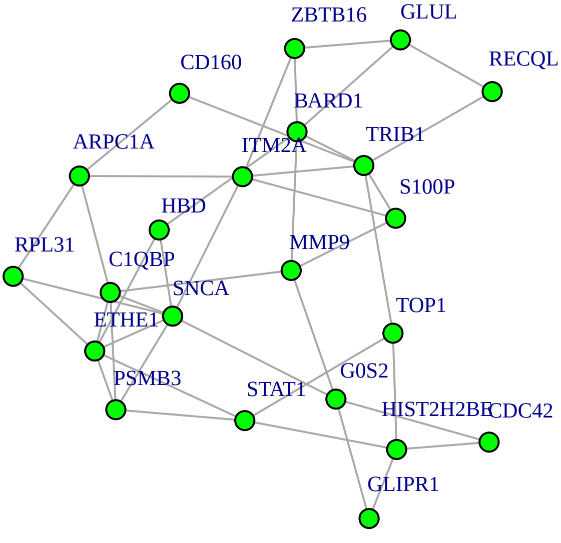

MST2 of the coexpression network for NEG ALL

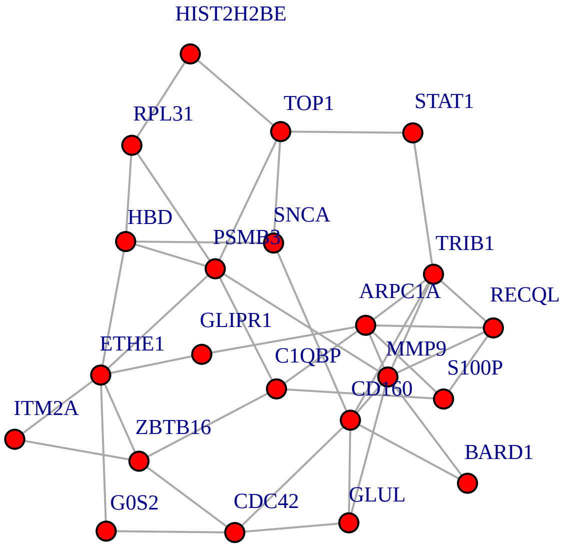

Pathway: ZHAN_MULTIPLE_MYELOMA_LB_DN

There are 28 genes in this pathway. This pathway was detected by Both

BCR/ABL ALL
Major Gene (BCR/ABL): IFI35
Weight Factor: 1.298
Major Gene (NEG): XAF1
Weight Factor: 1.259

NEG ALL
Major Gene (NEG): XAF1
Weight Factor: 1.494
Major Gene (BCR/ABL): IFI35
Weight Factor: 1.338

MST2 of the coexpression network for BCR/ABL ALL

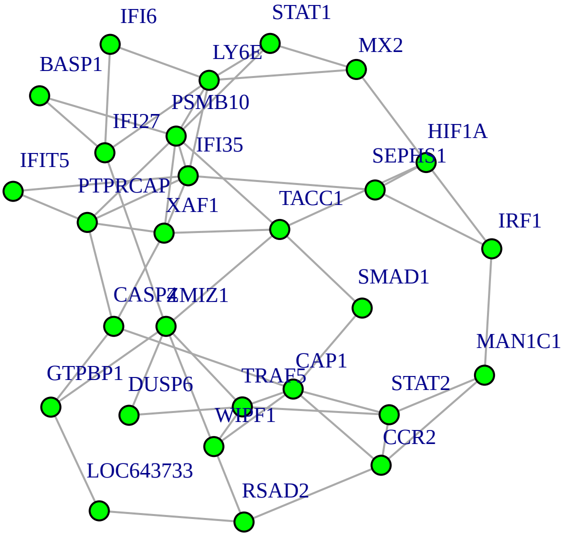

MST2 of the coexpression network for NEG ALL

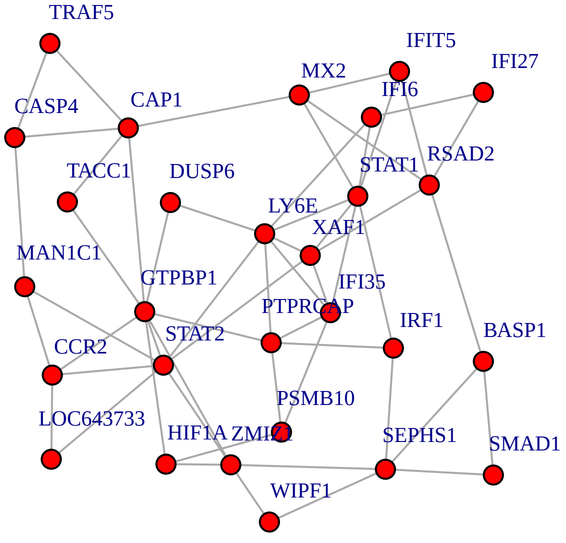

Pathway: ZHU_CMV_24_HR_DN

There are 54 genes in this pathway. This pathway was detected by Both

BCR/ABL ALL
Major Gene (BCR/ABL): PYCR1
Weight Factor: 1.337
Major Gene (NEG): COL6A2
Weight Factor: 1.225

NEG ALL
Major Gene (NEG): COL6A2
Weight Factor: 1.654
Major Gene (BCR/ABL): PYCR1
Weight Factor: 1.317

MST2 of the coexpression network for BCR/ABL ALL

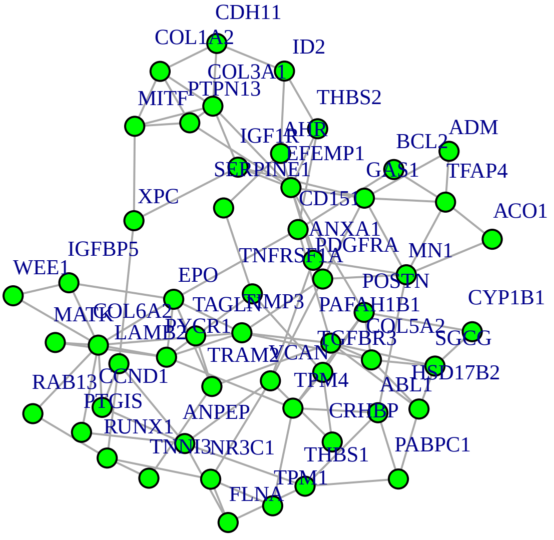

MST2 of the coexpression network for NEG ALL

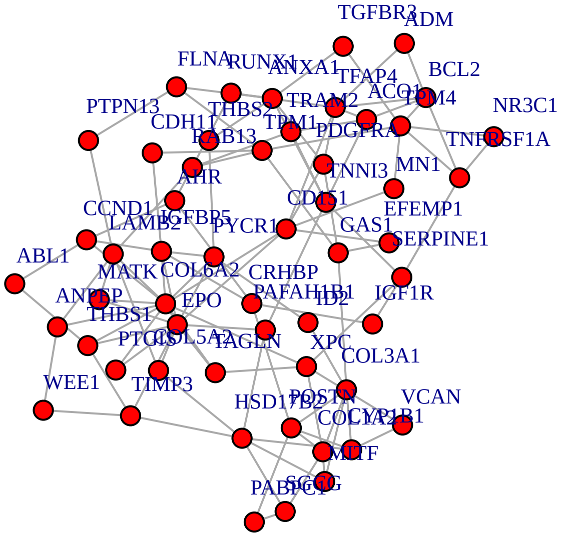

Pathway: ZHU_CMV_ALL_DN

There are 72 genes in this pathway. This pathway was detected by Both

BCR/ABL ALL
Major Gene (BCR/ABL): TIMP3
Weight Factor: 1.335
Major Gene (NEG): COL6A2
Weight Factor: 1.145

NEG ALL
Major Gene (NEG): COL6A2
Weight Factor: 1.642
Major Gene (BCR/ABL): TIMP3
Weight Factor: 1.336

MST2 of the coexpression network for BCR/ABL ALL

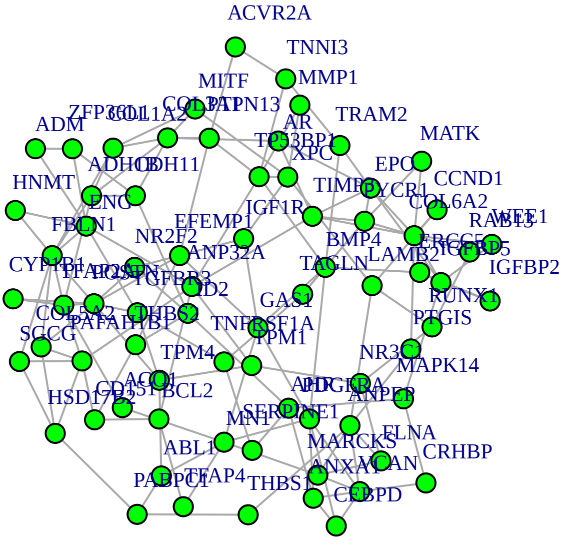

MST2 of the coexpression network for NEG ALL

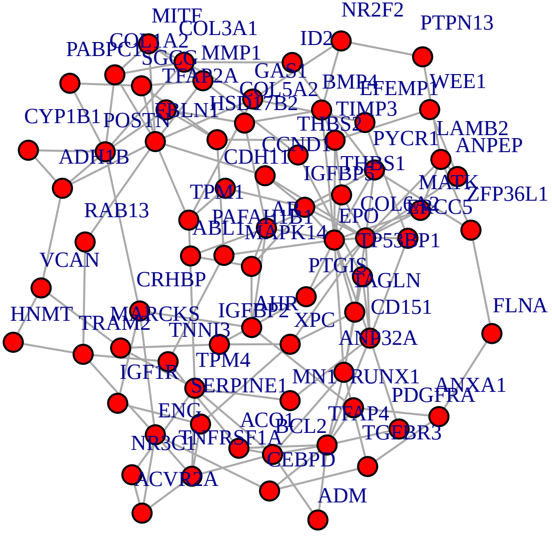

Pathway: LIANG_SILENCED_BY_METHYLATION_2

There are 31 genes in this pathway. This pathway was detected by Both

BCR/ABL ALL
Major Gene (BCR/ABL): OAS1
Weight Factor: 1.425
Major Gene (NEG): IRF7
Weight Factor: 0.911

NEG ALL
Major Gene (NEG): IRF7
Weight Factor: 1.489
Major Gene (BCR/ABL): OAS1
Weight Factor: 1.418

MST2 of the coexpression network for BCR/ABL ALL

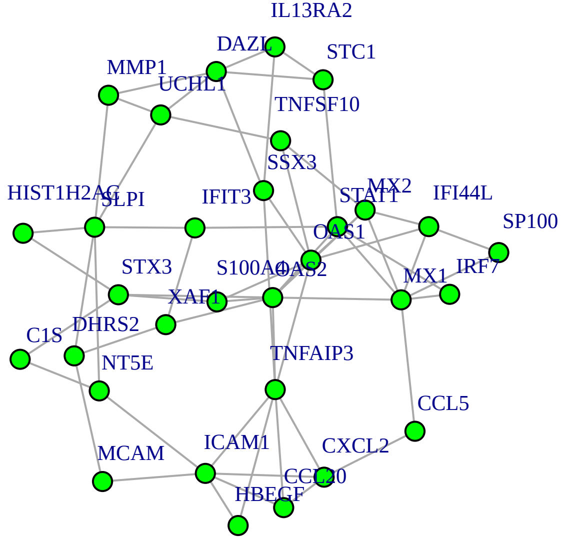

MST2 of the coexpression network for NEG ALL

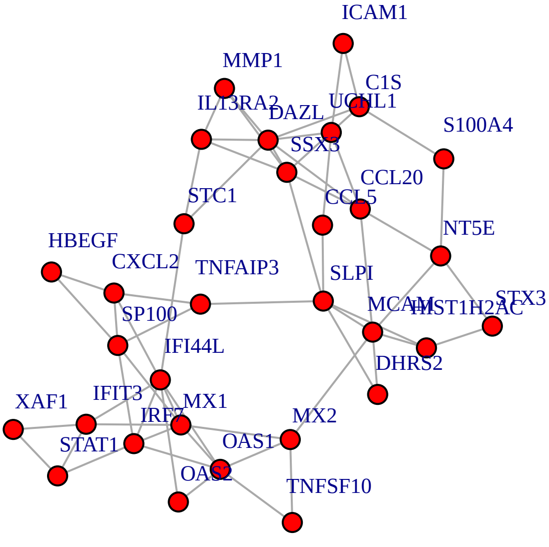

Pathway: BROWNE_INTERFERON_RESPONSIVE_GENES

There are 67 genes in this pathway. This pathway was detected by Both

BCR/ABL ALL
Major Gene (BCR/ABL): ISG15
Weight Factor: 1.34
Major Gene (NEG): ISG15
Weight Factor: 1.34

NEG ALL
Major Gene (NEG): ISG15
Weight Factor: 1.469
Major Gene (BCR/ABL): ISG15
Weight Factor: 1.469

MST2 of the coexpression network for BCR/ABL ALL

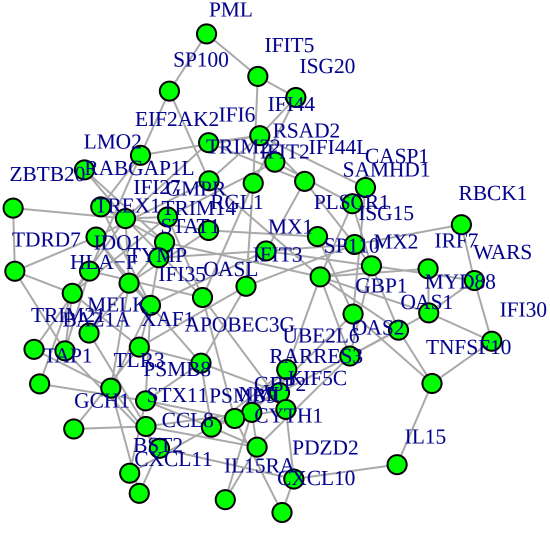

MST2 of the coexpression network for NEG ALL

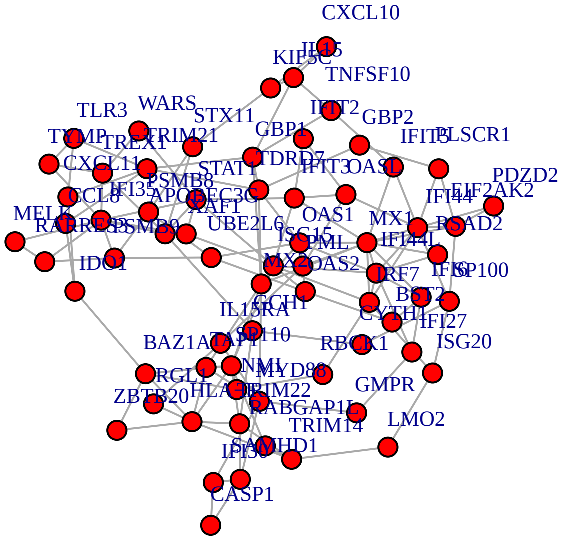

Pathway: KRASNOSELSKAYA_ILF3_TARGETS_UP

There are 29 genes in this pathway. This pathway was detected by Both

BCR/ABL ALL
Major Gene (BCR/ABL): IFI35
Weight Factor: 1.379
Major Gene (NEG): ISG15
Weight Factor: 1.216

NEG ALL
Major Gene (NEG): ISG15
Weight Factor: 1.432
Major Gene (BCR/ABL): IFI35
Weight Factor: 1.026

MST2 of the coexpression network for BCR/ABL ALL

MST2 of the coexpression network for NEG ALL

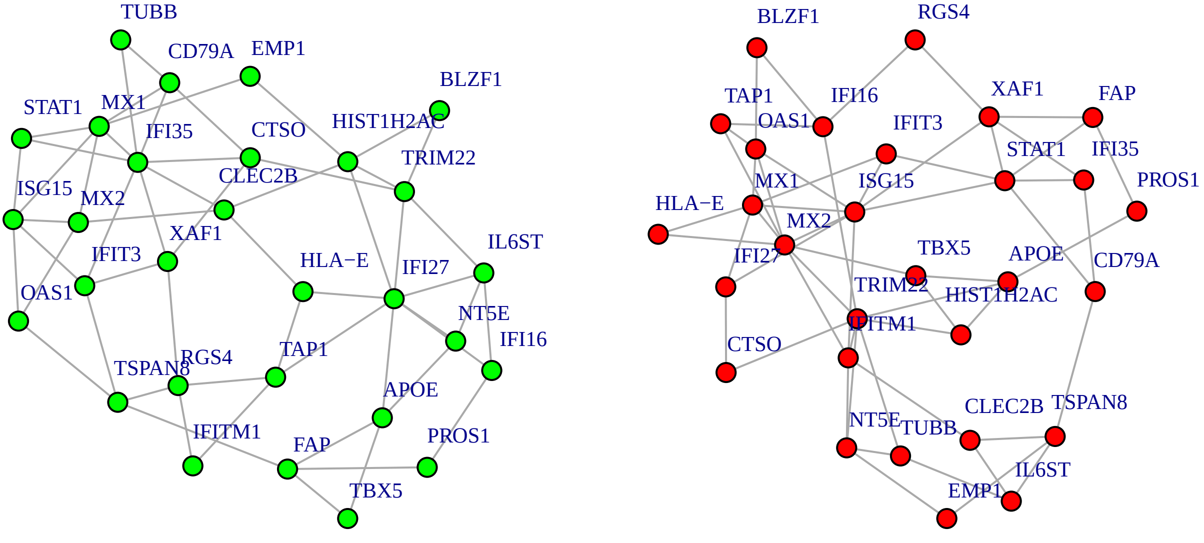

Pathway: GENTILE_UV_RESPONSE_CLUSTER_D4

There are 48 genes in this pathway. This pathway was detected by Both

BCR/ABL ALL
Major Gene (BCR/ABL): PUM1
Weight Factor: 1.528
Major Gene (NEG): PUM1
Weight Factor: 1.528

NEG ALL
Major Gene (NEG): PUM1
Weight Factor: 1.422
Major Gene (BCR/ABL): PUM1
Weight Factor: 1.422

MST2 of the coexpression network for BCR/ABL ALL

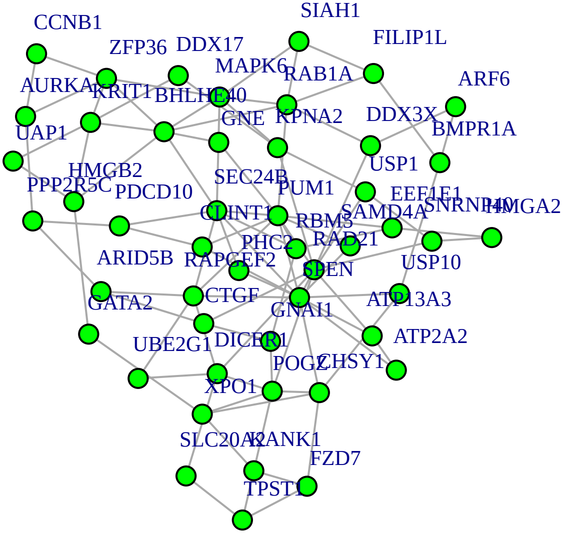

MST2 of the coexpression network for NEG ALL

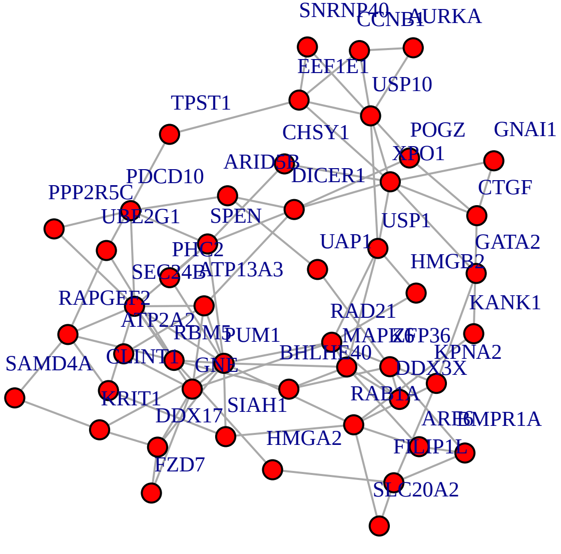

Pathway: KEGG_BLADDER_CANCER

There are 42 genes in this pathway. This pathway was detected by Both

BCR/ABL ALL
Major Gene (BCR/ABL): VEGFB
Weight Factor: 1.495
Major Gene (NEG): TYMP
Weight Factor: 1.16

NEG ALL
Major Gene (NEG): TYMP
Weight Factor: 1.491
Major Gene (BCR/ABL): VEGFB
Weight Factor: 1.348

MST2 of the coexpression network for BCR/ABL ALL

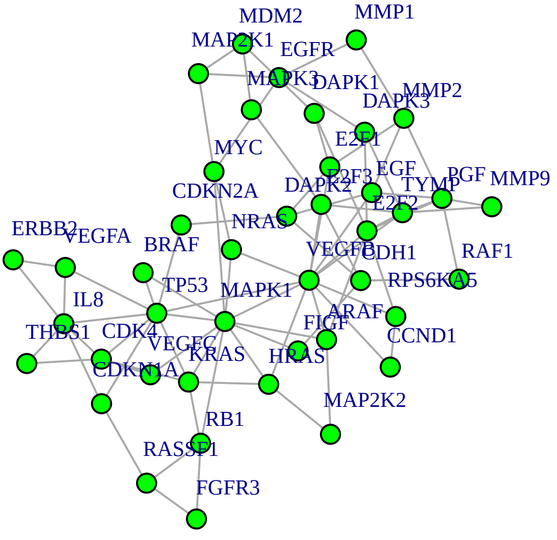

MST2 of the coexpression network for NEG ALL

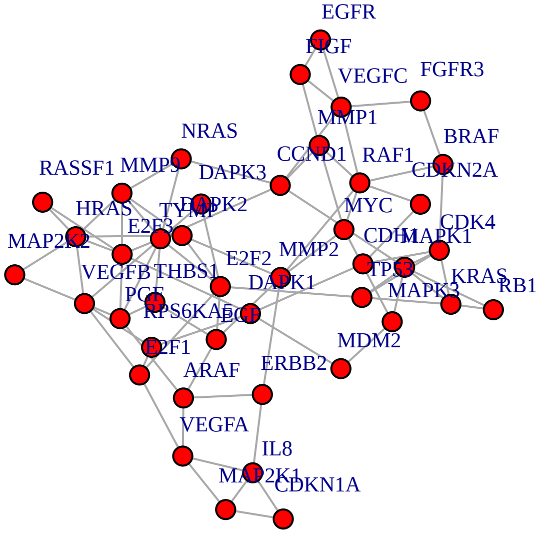

Pathway: GARGALOVIC_RESPONSE_TO_OXIDIZED_PHOSPHOLIPIDS_GREY_DN

There are 40 genes in this pathway. This pathway was detected by GSNCA

BCR/ABL ALL
Major Gene (BCR/ABL): CROT
Weight Factor: 1.42
Major Gene (NEG): GRAP
Weight Factor: 1.077

NEG ALL
Major Gene (NEG): GRAP
Weight Factor: 1.411
Major Gene (BCR/ABL): CROT
Weight Factor: 1.32

MST2 of the coexpression network for BCR/ABL ALL

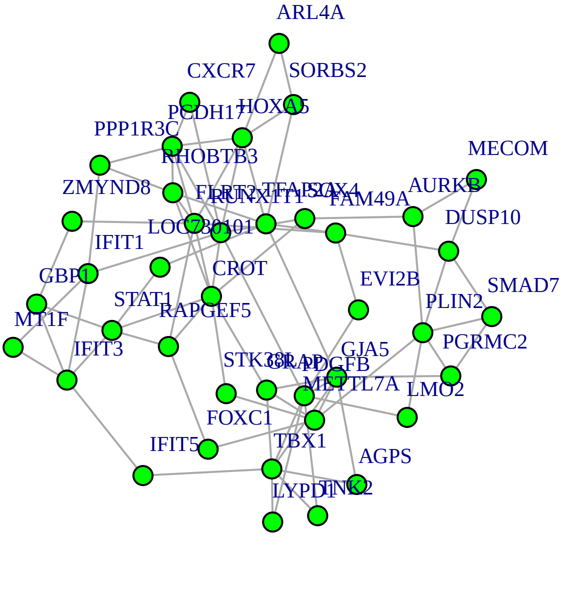

MST2 of the coexpression network for NEG ALL

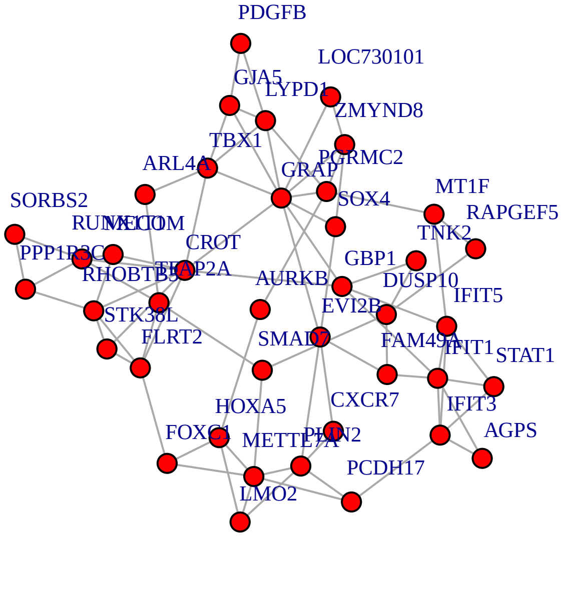

Pathway: GRAHAM_CML_QUIESCENT_VS_CML_DIVIDING_UP

There are 22 genes in this pathway. This pathway was detected by GSNCA

BCR/ABL ALL
Major Gene (BCR/ABL): CXCL3
Weight Factor: 1.419
Major Gene (NEG): CXCL6
Weight Factor: 0.744

NEG ALL
Major Gene (NEG): CXCL6
Weight Factor: 1.475
Major Gene (BCR/ABL): CXCL3
Weight Factor: 1.297

MST2 of the coexpression network for BCR/ABL ALL

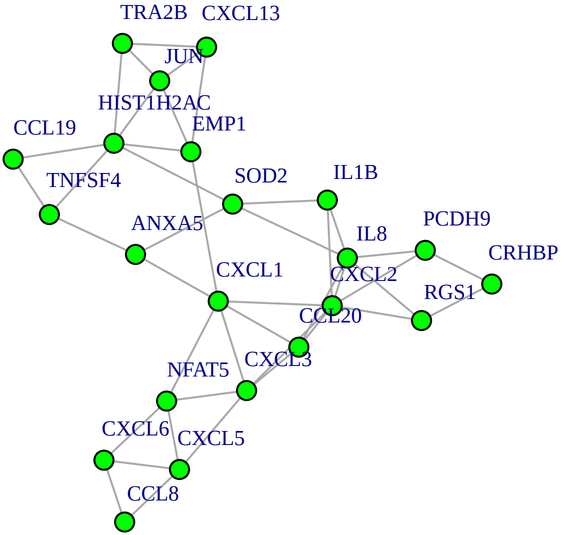

MST2 of the coexpression network for NEG ALL

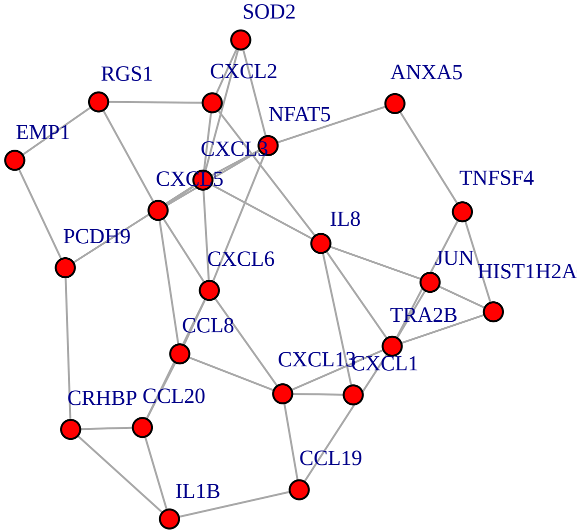

Pathway: ZIRN_TRETINOIN_RESPONSE_WT1_UP

There are 15 genes in this pathway. This pathway was detected by GSNCA

BCR/ABL ALL
Major Gene (BCR/ABL): ZNF133
Weight Factor: 1.407
Major Gene (NEG): PDGFRA
Weight Factor: 1.02

NEG ALL
Major Gene (NEG): PDGFRA
Weight Factor: 1.286
Major Gene (BCR/ABL): ZNF133
Weight Factor: 0.885

MST2 of the coexpression network for BCR/ABL ALL

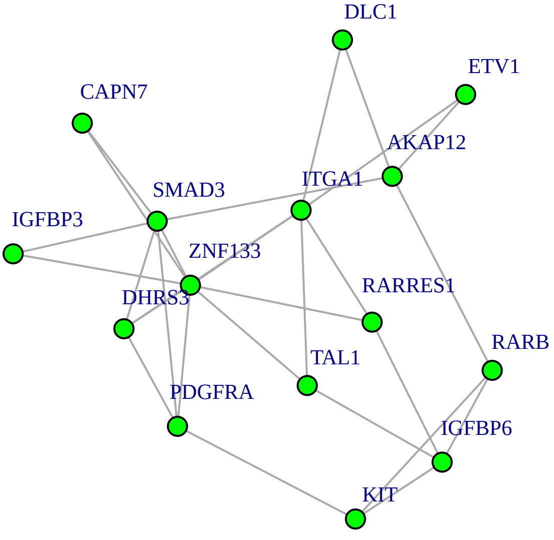

MST2 of the coexpression network for NEG ALL

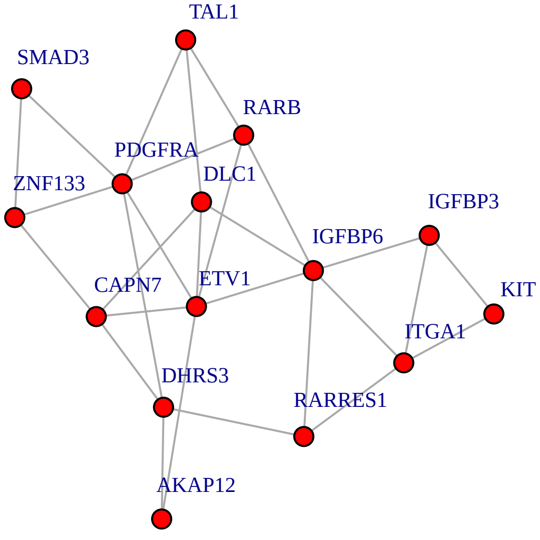

Pathway: MAHADEVAN_RESPONSE_TO_MP470_UP

There are 18 genes in this pathway. This pathway was detected by GSNCA

BCR/ABL ALL
Major Gene (BCR/ABL): CXCL3
Weight Factor: 1.294
Major Gene (NEG): IFI44L
Weight Factor: 0.794

NEG ALL
Major Gene (NEG): IFI44L
Weight Factor: 1.413
Major Gene (BCR/ABL): CXCL3
Weight Factor: 1.127

MST2 of the coexpression network for BCR/ABL ALL

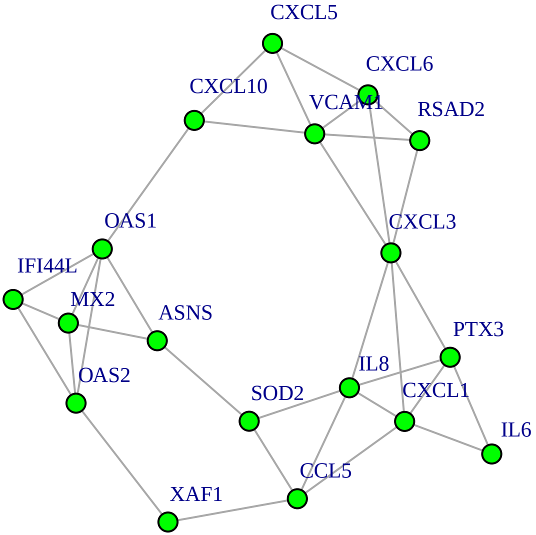

MST2 of the coexpression network for NEG ALL

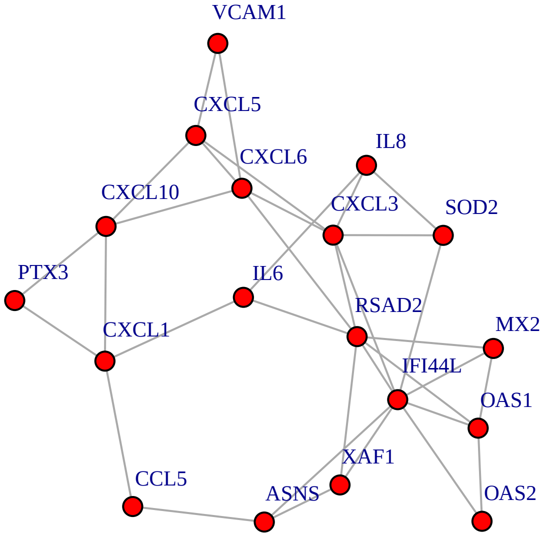

Pathway: APPIERTO_RESPONSE_TO_FENRETINIDE_UP

There are 23 genes in this pathway. This pathway was detected by GSNCA

BCR/ABL ALL
Major Gene (BCR/ABL): HSPA8
Weight Factor: 1.429
Major Gene (NEG): CCL13
Weight Factor: 0.833

NEG ALL
Major Gene (NEG): CCL13
Weight Factor: 1.458
Major Gene (BCR/ABL): HSPA8
Weight Factor: 1.114

MST2 of the coexpression network for BCR/ABL ALL

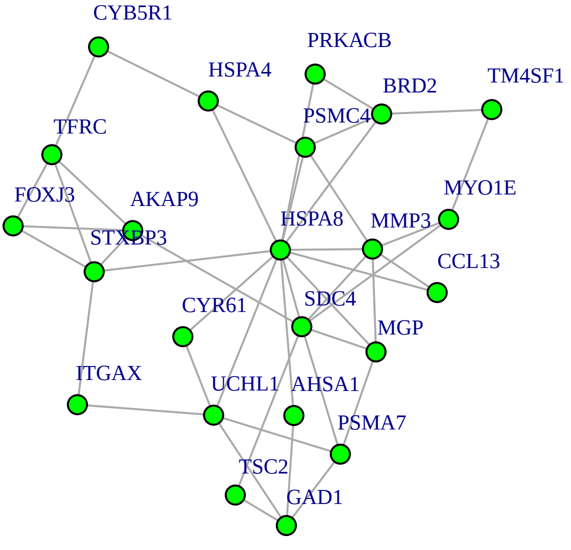

MST2 of the coexpression network for NEG ALL

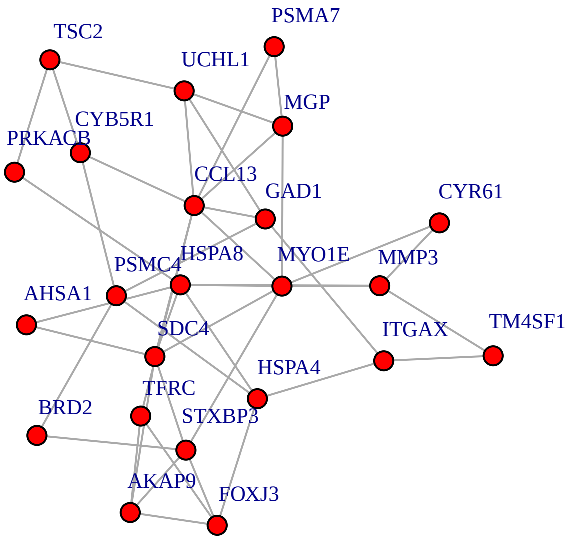

Pathway: SCHLOSSER_MYC_TARGETS_AND_SERUM_RESPONSE_DN

There are 46 genes in this pathway. This pathway was detected by GSNCA

BCR/ABL ALL
Major Gene (BCR/ABL): EBNA1BP2
Weight Factor: 1.463
Major Gene (NEG): NCL
Weight Factor: 1.19

NEG ALL
Major Gene (NEG): NCL
Weight Factor: 1.408
Major Gene (BCR/ABL): EBNA1BP2
Weight Factor: 1.324

MST2 of the coexpression network for BCR/ABL ALL

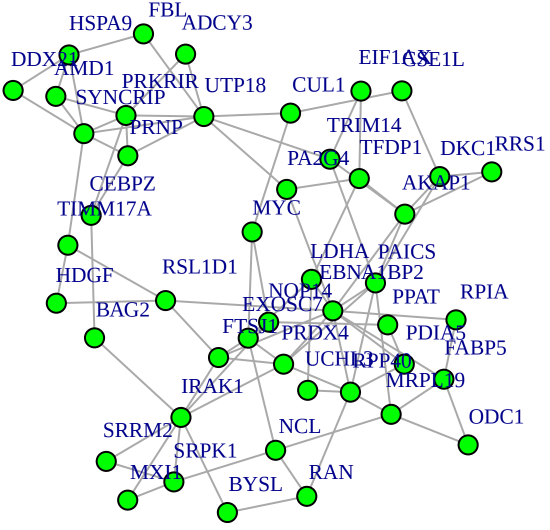

MST2 of the coexpression network for NEG ALL

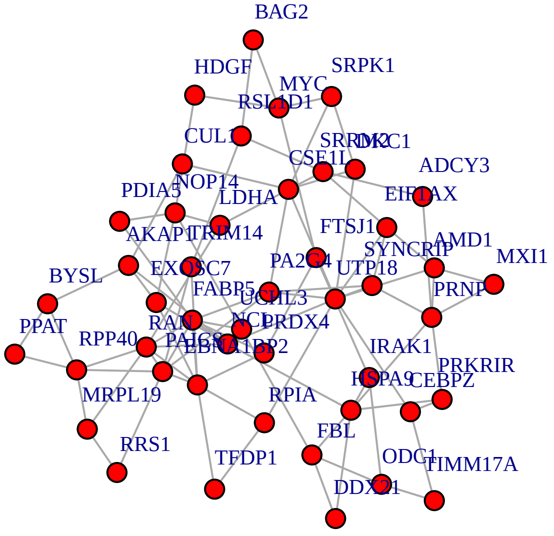

Pathway: DAUER_STAT3_TARGETS_UP

There are 36 genes in this pathway. This pathway was detected by GSNCA

BCR/ABL ALL
Major Gene (BCR/ABL): LBP
Weight Factor: 1.262
Major Gene (NEG): LBP
Weight Factor: 1.262

NEG ALL
Major Gene (NEG): LBP
Weight Factor: 1.539
Major Gene (BCR/ABL): LBP
Weight Factor: 1.539

MST2 of the coexpression network for BCR/ABL ALL

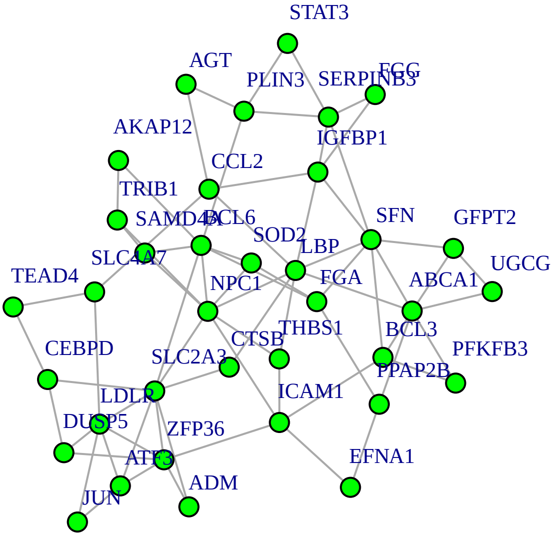

MST2 of the coexpression network for NEG ALL

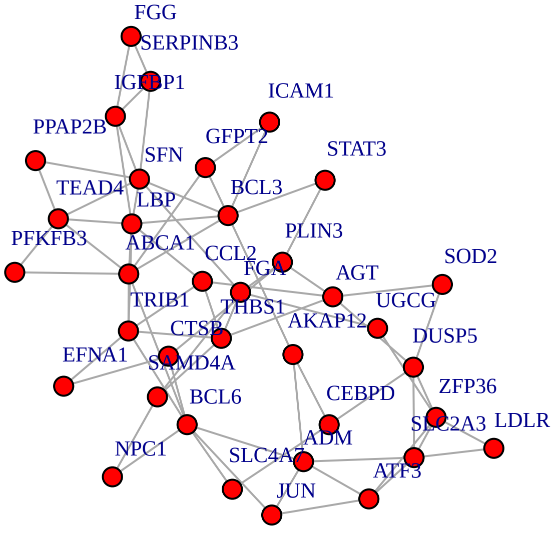

Pathway: SIMBULAN_UV_RESPONSE_NORMAL_DN

There are 29 genes in this pathway. This pathway was detected by GSNCA

BCR/ABL ALL
Major Gene (BCR/ABL): CCNF
Weight Factor: 1.377
Major Gene (NEG): ITGB4
Weight Factor: 1.076

NEG ALL
Major Gene (NEG): ITGB4
Weight Factor: 1.421
Major Gene (BCR/ABL): CCNF
Weight Factor: 1.398

MST2 of the coexpression network for BCR/ABL ALL

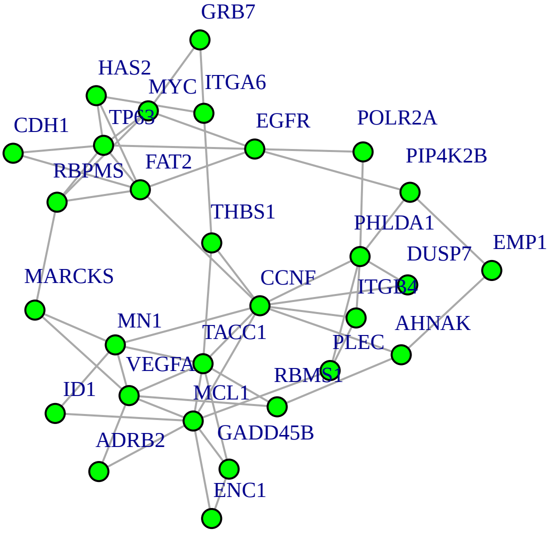

MST2 of the coexpression network for NEG ALL

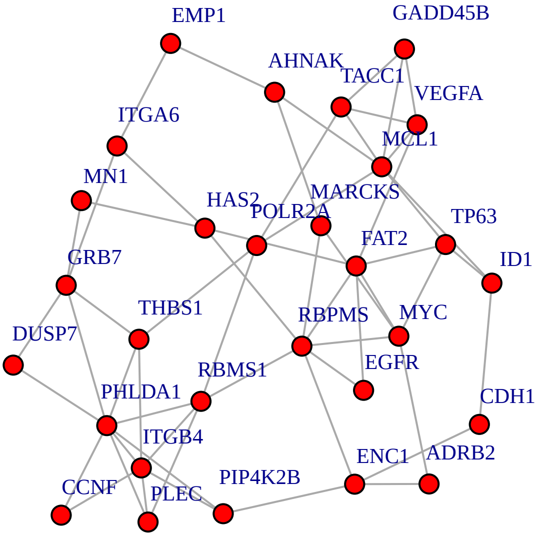

Pathway: BERTUCCI_INVASIVE_CARCINOMA_DUCTAL_VS_LOBULAR_UP

There are 18 genes in this pathway. This pathway was detected by GSNCA

BCR/ABL ALL
Major Gene (BCR/ABL): HELZ
Weight Factor: 1.272
Major Gene (NEG): GRB7
Weight Factor: 0.715

NEG ALL
Major Gene (NEG): GRB7
Weight Factor: 1.415
Major Gene (BCR/ABL): HELZ
Weight Factor: 1.051

MST2 of the coexpression network for BCR/ABL ALL

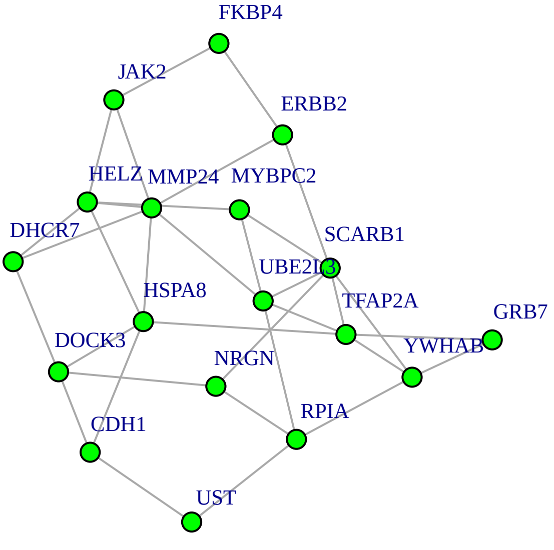

MST2 of the coexpression network for NEG ALL

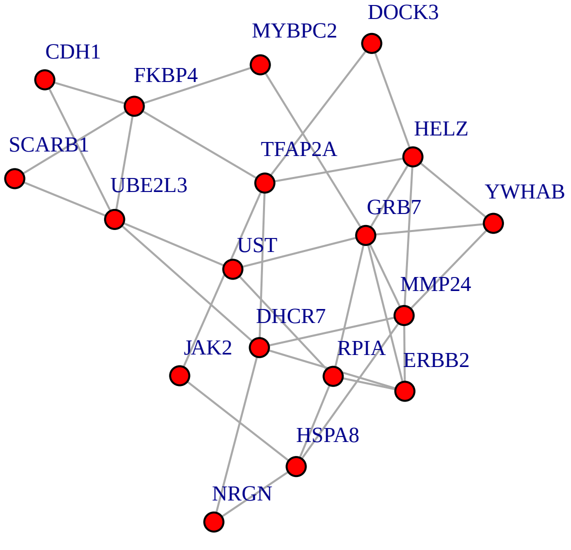

Pathway: RADAEVA_RESPONSE_TO_IFNA1_UP

There are 31 genes in this pathway. This pathway was detected by GSNCA

BCR/ABL ALL
Major Gene (BCR/ABL): IFI35
Weight Factor: 1.492
Major Gene (NEG): IRF7
Weight Factor: 0.846

NEG ALL
Major Gene (NEG): IRF7
Weight Factor: 1.321
Major Gene (BCR/ABL): IFI35
Weight Factor: 1.19

MST2 of the coexpression network for BCR/ABL ALL

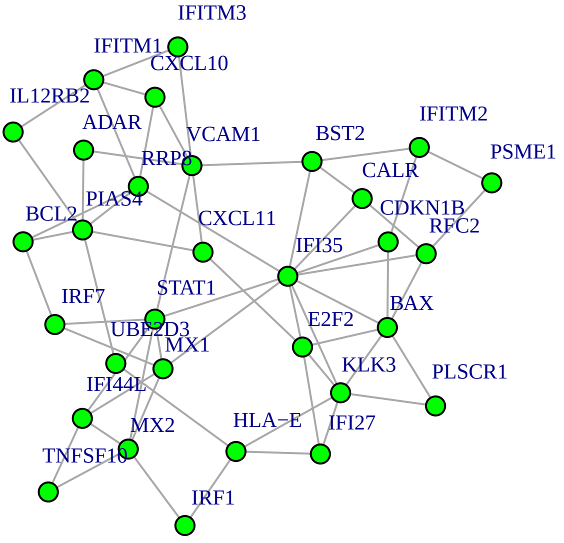

MST2 of the coexpression network for NEG ALL

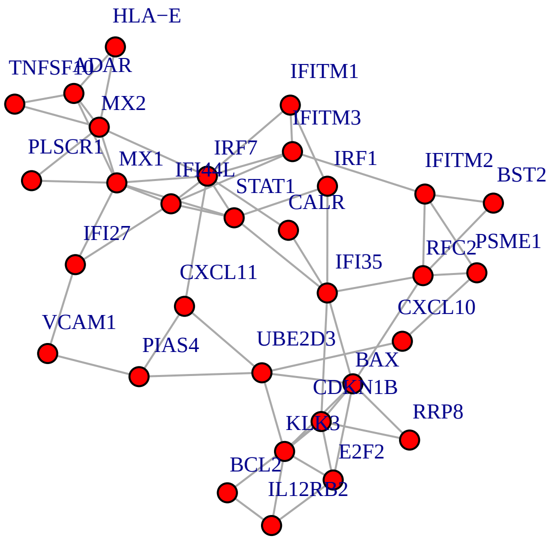

Pathway: MOOTHA_VOXPHOS

There are 68 genes in this pathway. This pathway was detected by GSNCA

BCR/ABL ALL
Major Gene (BCR/ABL): UQCRQ
Weight Factor: 1.361
Major Gene (NEG): NDUFA2
Weight Factor: 1.338

NEG ALL
Major Gene (NEG): NDUFA2
Weight Factor: 1.356
Major Gene (BCR/ABL): UQCRQ
Weight Factor: 1.299

MST2 of the coexpression network for BCR/ABL ALL

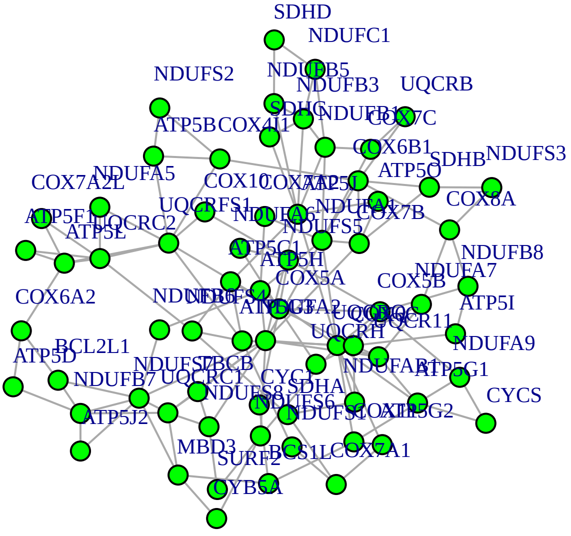

MST2 of the coexpression network for NEG ALL

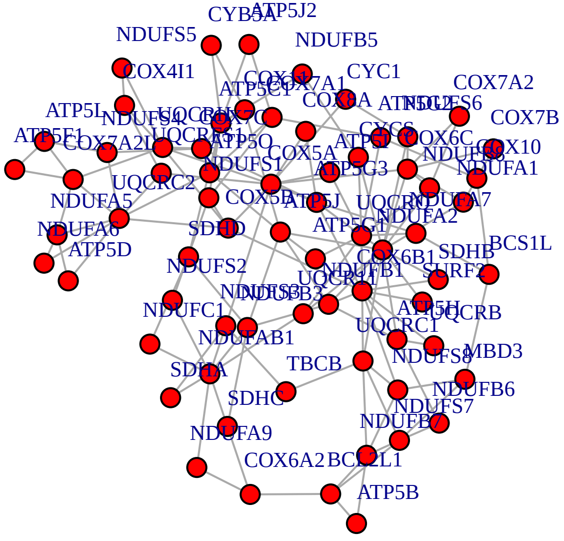

Pathway: CROMER_METASTASIS_UP

There are 47 genes in this pathway. This pathway was detected by GSNCA

BCR/ABL ALL
Major Gene (BCR/ABL): ZFC3H1
Weight Factor: 1.436
Major Gene (NEG): XPO1
Weight Factor: 1.02

NEG ALL
Major Gene (NEG): XPO1
Weight Factor: 1.532
Major Gene (BCR/ABL): ZFC3H1
Weight Factor: 1.253

MST2 of the coexpression network for BCR/ABL ALL

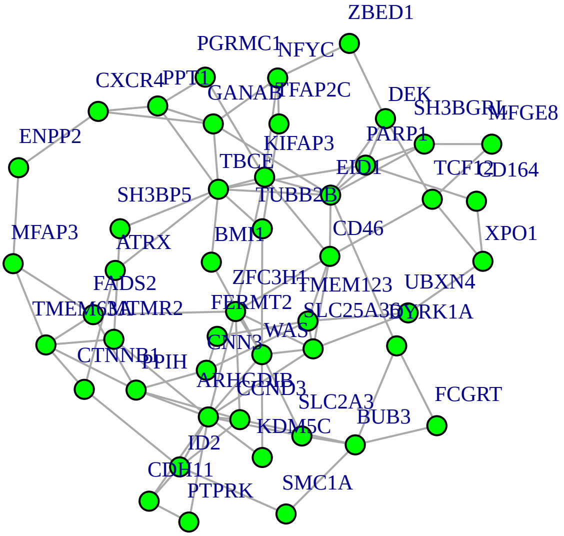

MST2 of the coexpression network for NEG ALL

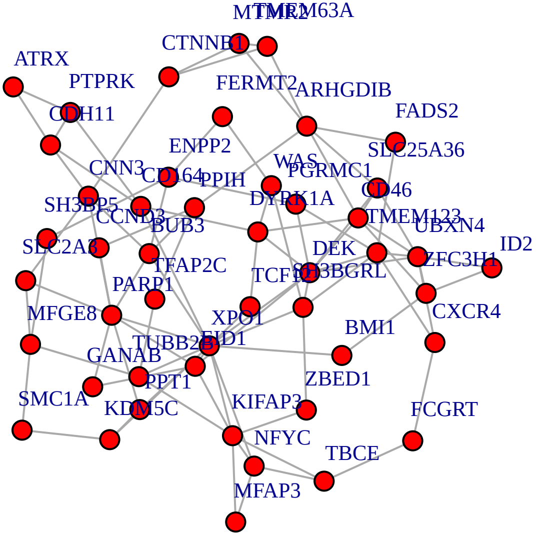

Pathway: NAKAMURA_METASTASIS_MODEL_DN

There are 25 genes in this pathway. This pathway was detected by GSNCA

BCR/ABL ALL
Major Gene (BCR/ABL): FGFBP1
Weight Factor: 1.274
Major Gene (NEG): RUNX2
Weight Factor: 1.035

NEG ALL
Major Gene (NEG): RUNX2
Weight Factor: 1.453
Major Gene (BCR/ABL): FGFBP1
Weight Factor: 1.38

MST2 of the coexpression network for BCR/ABL ALL

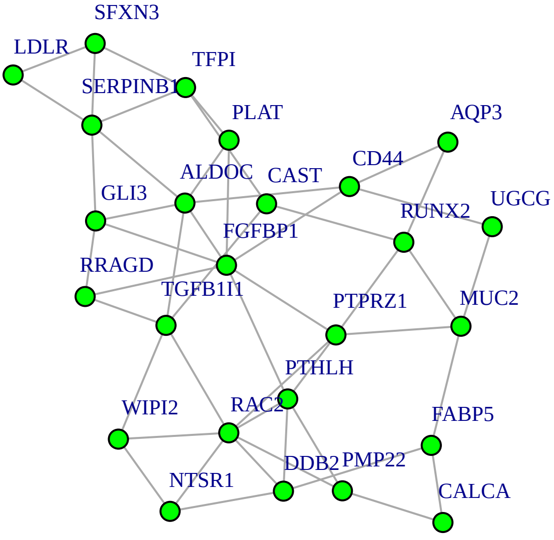

MST2 of the coexpression network for NEG ALL

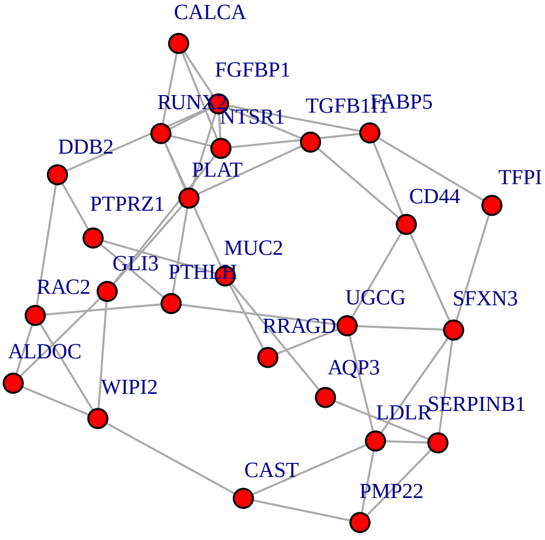

Pathway: LU_TUMOR_ANGIOGENESIS_UP

There are 22 genes in this pathway. This pathway was detected by GSNCA

BCR/ABL ALL
Major Gene (BCR/ABL): JAG1
Weight Factor: 1.417
Major Gene (NEG): VAV2
Weight Factor: 1.318

NEG ALL
Major Gene (NEG): VAV2
Weight Factor: 1.449
Major Gene (BCR/ABL): JAG1
Weight Factor: 1.117

MST2 of the coexpression network for BCR/ABL ALL

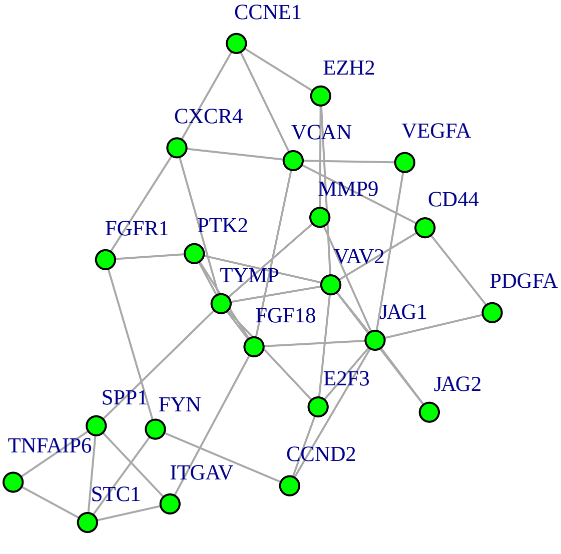

MST2 of the coexpression network for NEG ALL

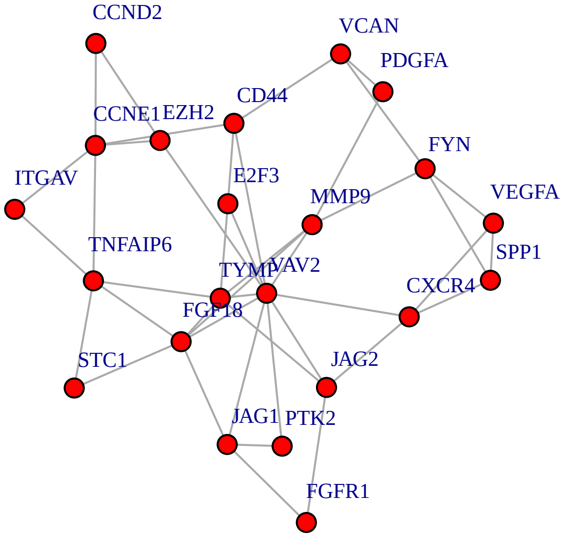

Pathway: CADWELL_ATG16L1_TARGETS_UP

There are 56 genes in this pathway. This pathway was detected by GSNCA

BCR/ABL ALL
Major Gene (BCR/ABL): ADIPOQ
Weight Factor: 1.395
Major Gene (NEG): DBH
Weight Factor: 0.979

NEG ALL
Major Gene (NEG): DBH
Weight Factor: 1.434
Major Gene (BCR/ABL): ADIPOQ
Weight Factor: 0.813

MST2 of the coexpression network for BCR/ABL ALL

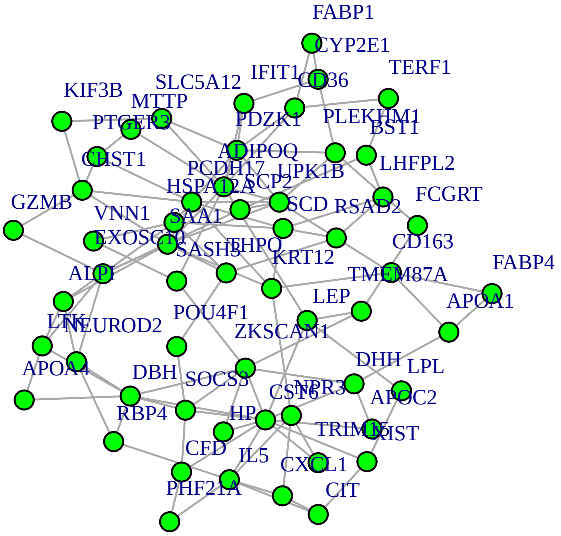

MST2 of the coexpression network for NEG ALL

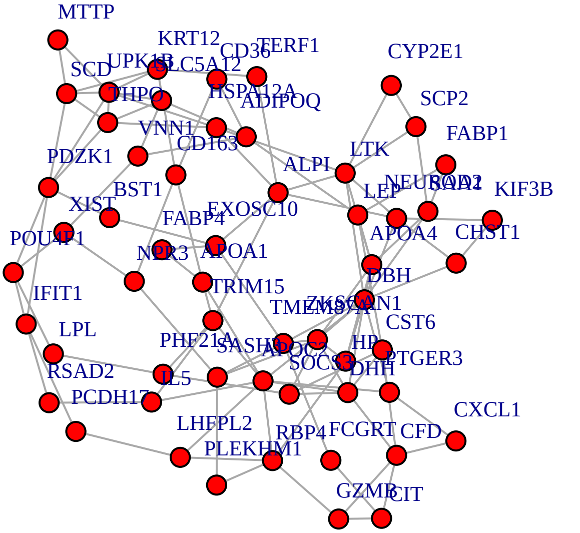

Pathway: GU_PDEF_TARGETS_DN

There are 24 genes in this pathway. This pathway was detected by GSNCA

BCR/ABL ALL
Major Gene (BCR/ABL): PARVA
Weight Factor: 1.484
Major Gene (NEG): ITGB4
Weight Factor: 1.042

NEG ALL
Major Gene (NEG): ITGB4
Weight Factor: 1.377
Major Gene (BCR/ABL): PARVA
Weight Factor: 1.24

MST2 of the coexpression network for BCR/ABL ALL

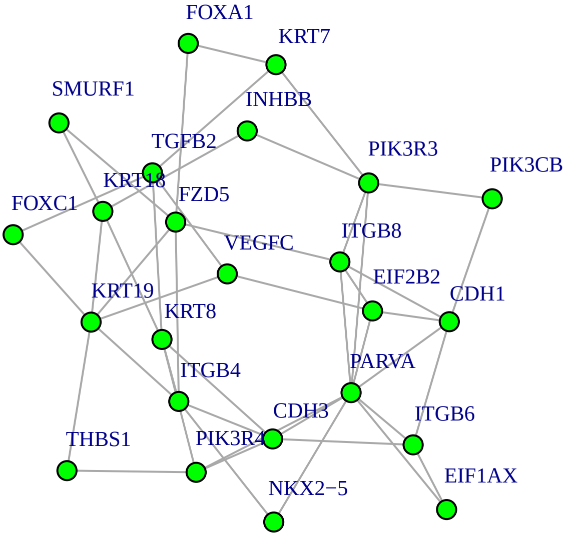

MST2 of the coexpression network for NEG ALL

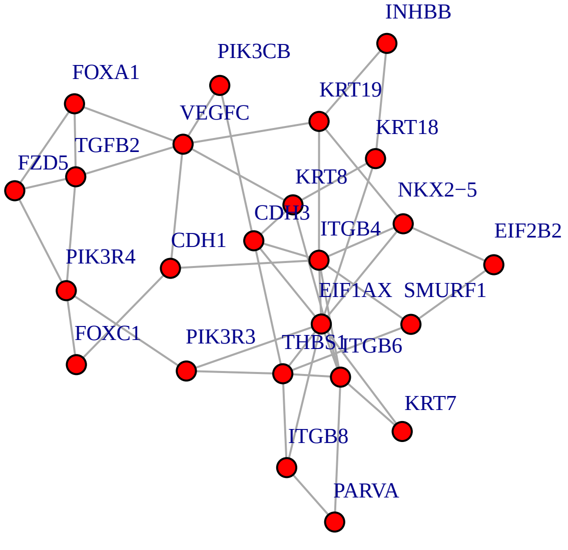

Pathway: MILI_PSEUDOPODIA

There are 29 genes in this pathway. This pathway was detected by GSNCA

BCR/ABL ALL
Major Gene (BCR/ABL): CYB5R3
Weight Factor: 1.327
Major Gene (NEG): SEPT7
Weight Factor: 1.27

NEG ALL
Major Gene (NEG): SEPT7
Weight Factor: 1.43
Major Gene (BCR/ABL): CYB5R3
Weight Factor: 1.361

MST2 of the coexpression network for BCR/ABL ALL

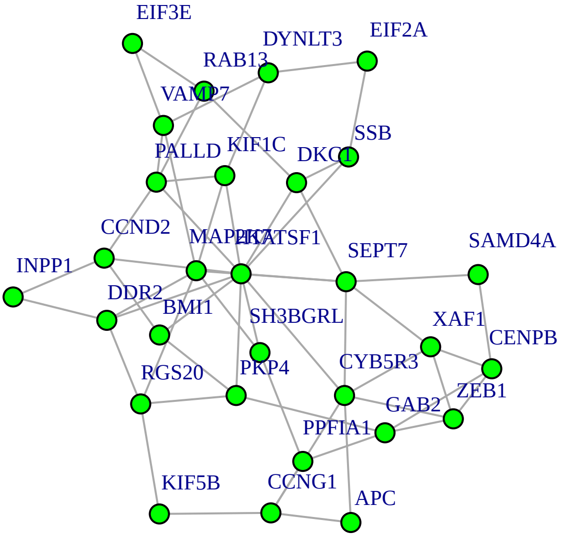

MST2 of the coexpression network for NEG ALL

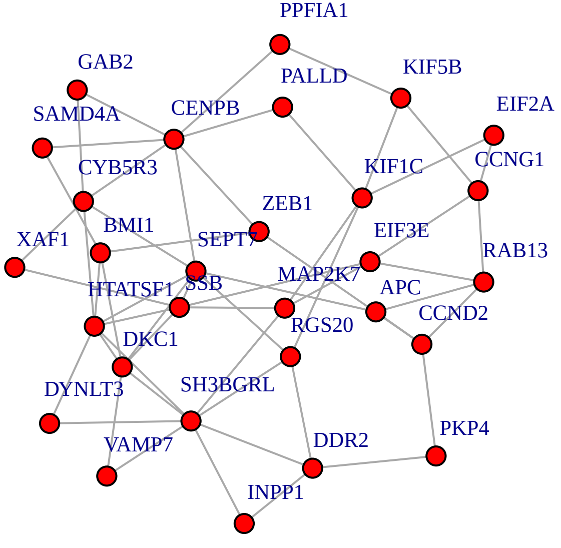

Pathway: VALK_AML_CLUSTER_5

There are 24 genes in this pathway. This pathway was detected by GSNCA

BCR/ABL ALL
Major Gene (BCR/ABL): LILRB3
Weight Factor: 1.344
Major Gene (NEG): TYMP
Weight Factor: 0.907

NEG ALL
Major Gene (NEG): TYMP
Weight Factor: 1.429
Major Gene (BCR/ABL): LILRB3
Weight Factor: 0.704

MST2 of the coexpression network for BCR/ABL ALL

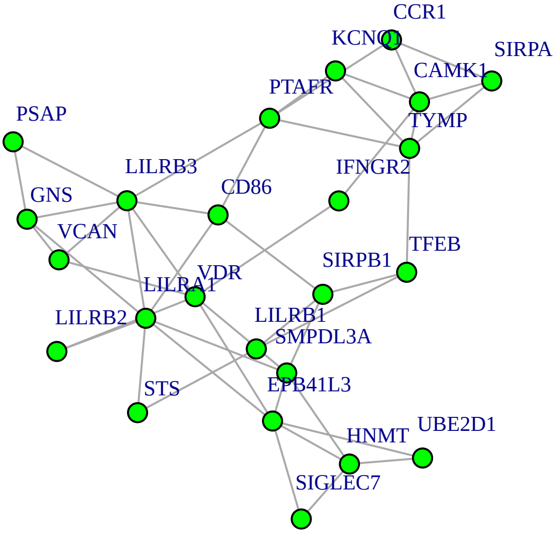

MST2 of the coexpression network for NEG ALL

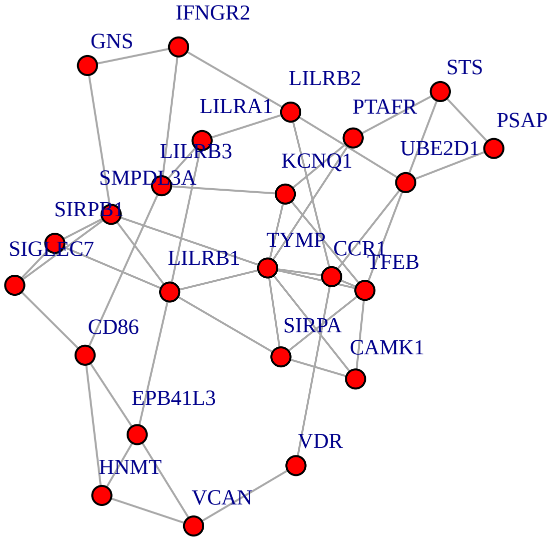

Pathway: VALK_AML_CLUSTER_11

There are 28 genes in this pathway. This pathway was detected by GSNCA

BCR/ABL ALL
Major Gene (BCR/ABL): APP
Weight Factor: 1.504
Major Gene (NEG): CD200
Weight Factor: 0.816

NEG ALL
Major Gene (NEG): CD200
Weight Factor: 1.317
Major Gene (BCR/ABL): APP
Weight Factor: 0.729

MST2 of the coexpression network for BCR/ABL ALL

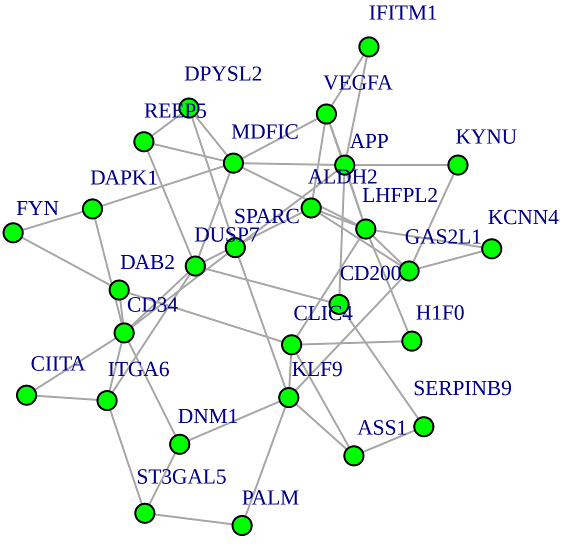

MST2 of the coexpression network for NEG ALL

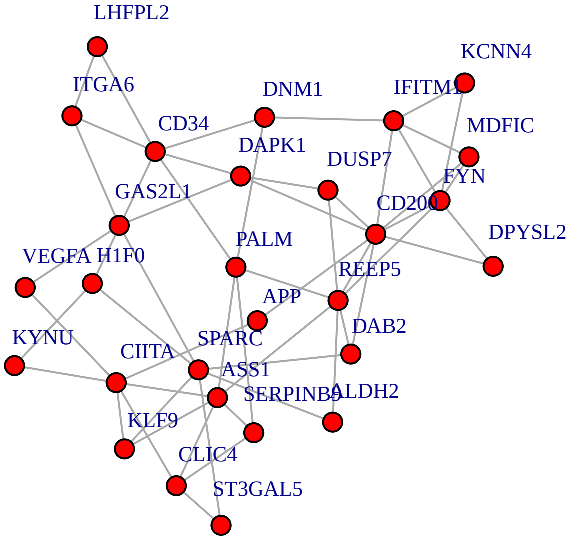

Pathway: VALK_AML_WITH_CEBPA

There are 29 genes in this pathway. This pathway was detected by GSNCA

BCR/ABL ALL
Major Gene (BCR/ABL): CD7
Weight Factor: 1.39
Major Gene (NEG): CAMP
Weight Factor: 1.12

NEG ALL
Major Gene (NEG): CAMP
Weight Factor: 1.537
Major Gene (BCR/ABL): CD7
Weight Factor: 1.435

MST2 of the coexpression network for BCR/ABL ALL

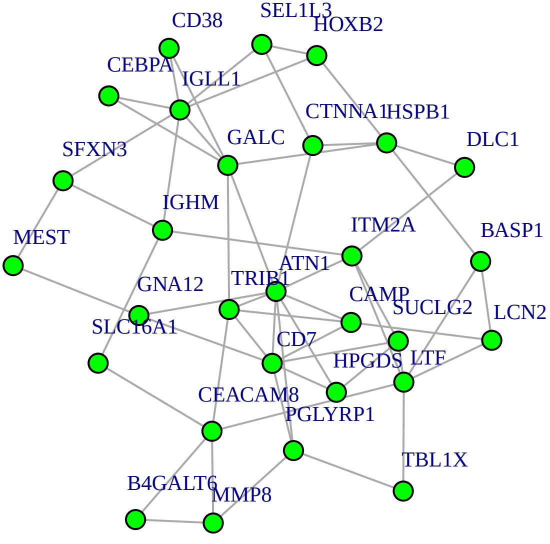

MST2 of the coexpression network for NEG ALL

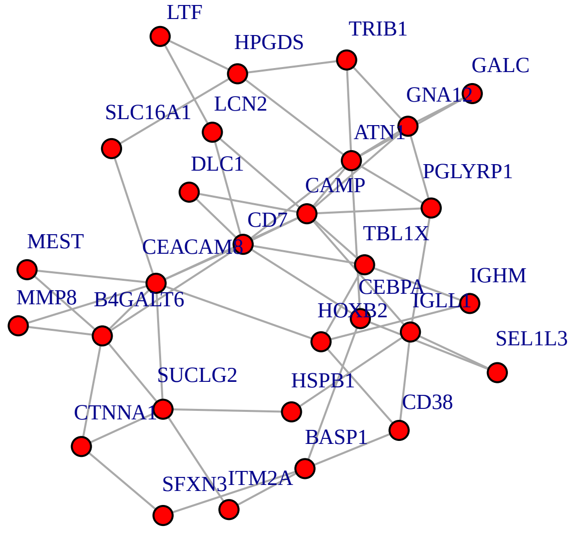

Pathway: ZHAN_MULTIPLE_MYELOMA_LB_UP

There are 17 genes in this pathway. This pathway was detected by GSNCA

BCR/ABL ALL
Major Gene (BCR/ABL): RASGRP1
Weight Factor: 1.289
Major Gene (NEG): CSF2RB
Weight Factor: 1.144

NEG ALL
Major Gene (NEG): CSF2RB
Weight Factor: 1.335
Major Gene (BCR/ABL): RASGRP1
Weight Factor: 0.625

MST2 of the coexpression network for BCR/ABL ALL

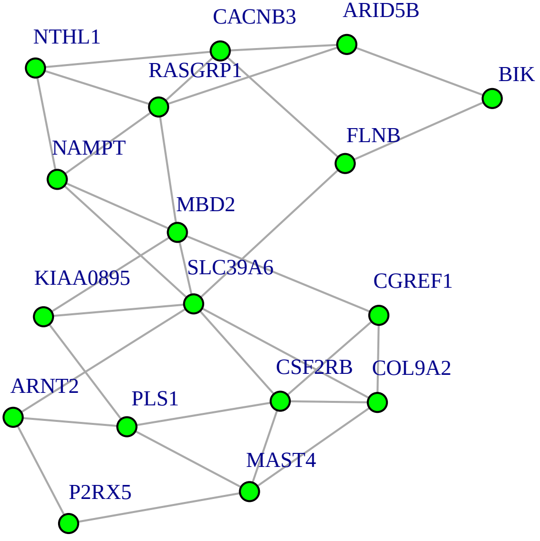

MST2 of the coexpression network for NEG ALL

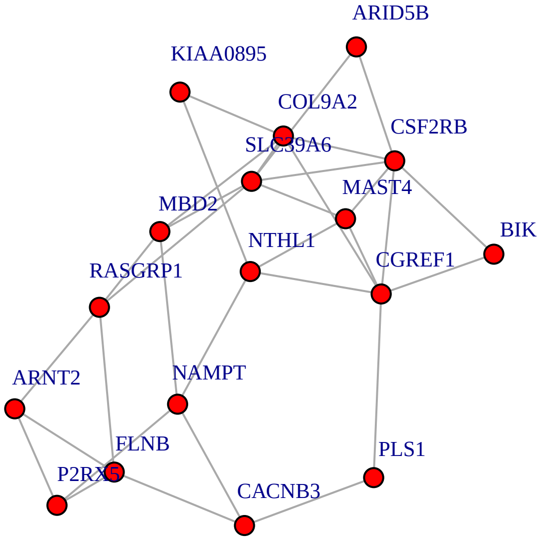

Pathway: URS_ADIPOCYTE_DIFFERENTIATION_DN

There are 27 genes in this pathway. This pathway was detected by GSNCA

BCR/ABL ALL
Major Gene (BCR/ABL): ROM1
Weight Factor: 1.409
Major Gene (NEG): COL6A1
Weight Factor: 1.326

NEG ALL
Major Gene (NEG): COL6A1
Weight Factor: 1.38
Major Gene (BCR/ABL): ROM1
Weight Factor: 1.355

MST2 of the coexpression network for BCR/ABL ALL

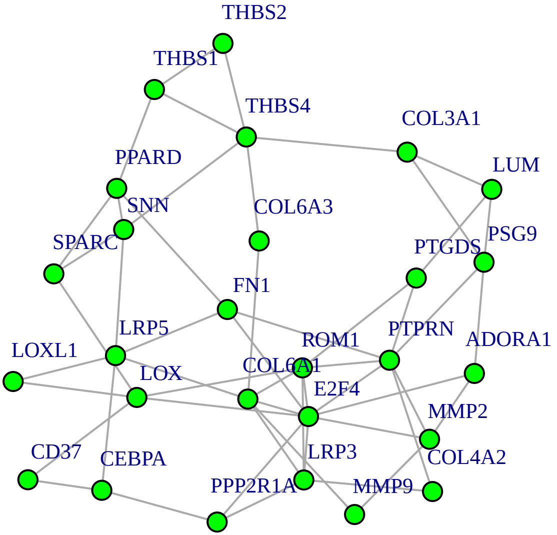

MST2 of the coexpression network for NEG ALL

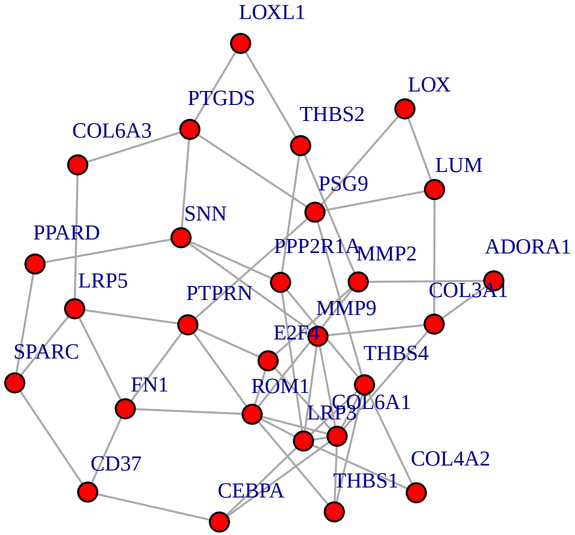

Pathway: JAZAERI_BREAST_CANCER_BRCA1_VS_BRCA2_DN

There are 34 genes in this pathway. This pathway was detected by GSNCA

BCR/ABL ALL
Major Gene (BCR/ABL): BMP6
Weight Factor: 1.386
Major Gene (NEG): PDE6A
Weight Factor: 1.21

NEG ALL
Major Gene (NEG): PDE6A
Weight Factor: 1.454
Major Gene (BCR/ABL): BMP6
Weight Factor: 1.006

MST2 of the coexpression network for BCR/ABL ALL

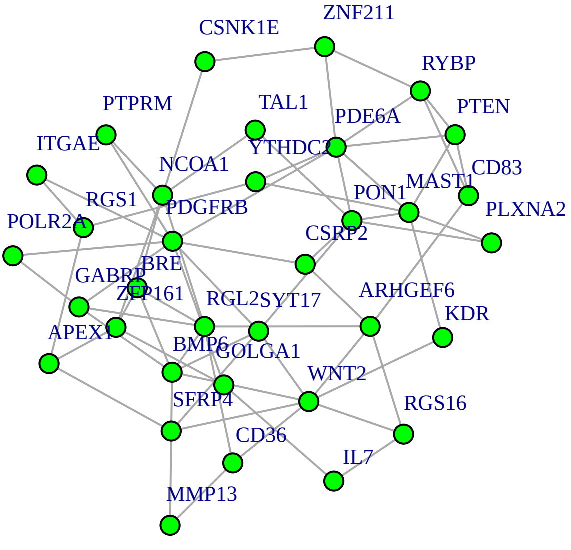

MST2 of the coexpression network for NEG ALL

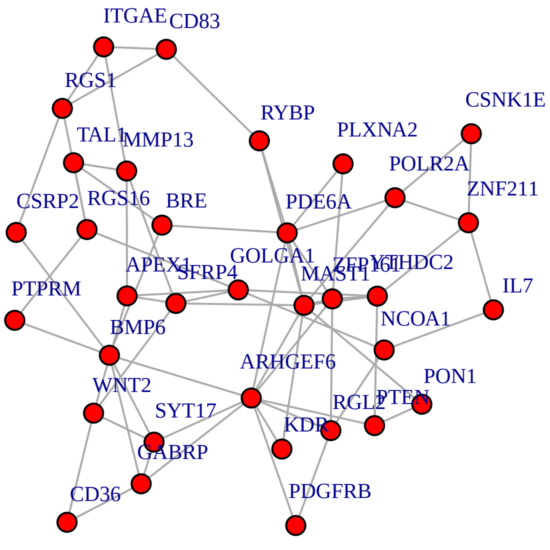

Pathway: XU_GH1_AUTOCRINE_TARGETS_DN

There are 84 genes in this pathway. This pathway was detected by GSNCA

BCR/ABL ALL
Major Gene (BCR/ABL): DYRK1B
Weight Factor: 1.385
Major Gene (NEG): TNFRSF4
Weight Factor: 1.045

NEG ALL
Major Gene (NEG): TNFRSF4
Weight Factor: 1.554
Major Gene (BCR/ABL): DYRK1B
Weight Factor: 1.512

MST2 of the coexpression network for BCR/ABL ALL

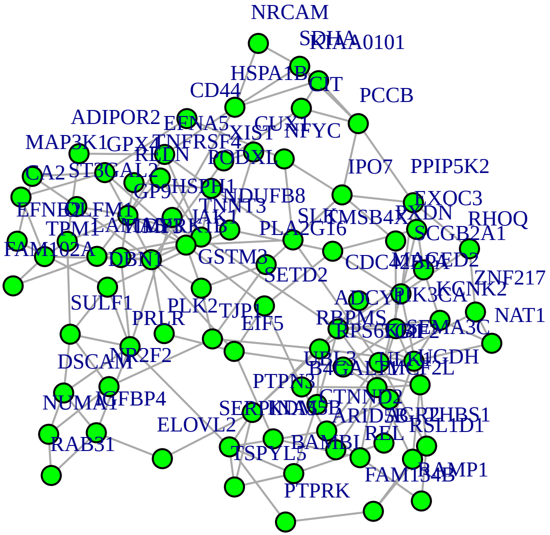

MST2 of the coexpression network for NEG ALL

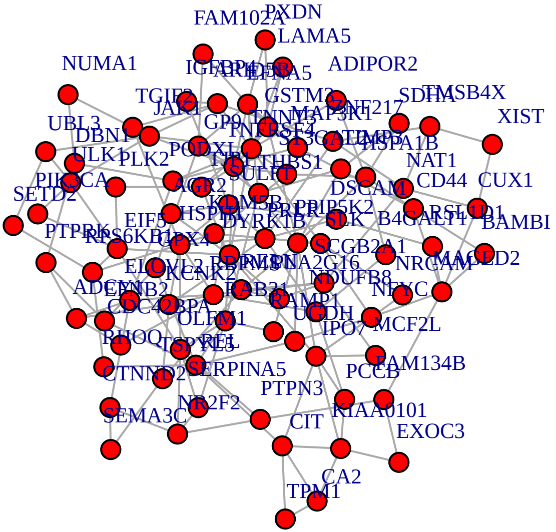

Pathway: MARIADASON_RESPONSE_TO_CURCUMIN_SULINDAC_5

There are 19 genes in this pathway. This pathway was detected by GSNCA

BCR/ABL ALL
Major Gene (BCR/ABL): TARS
Weight Factor: 1.162
Major Gene (NEG): TARS
Weight Factor: 1.162

NEG ALL
Major Gene (NEG): TARS
Weight Factor: 1.372
Major Gene (BCR/ABL): TARS
Weight Factor: 1.372

MST2 of the coexpression network for BCR/ABL ALL

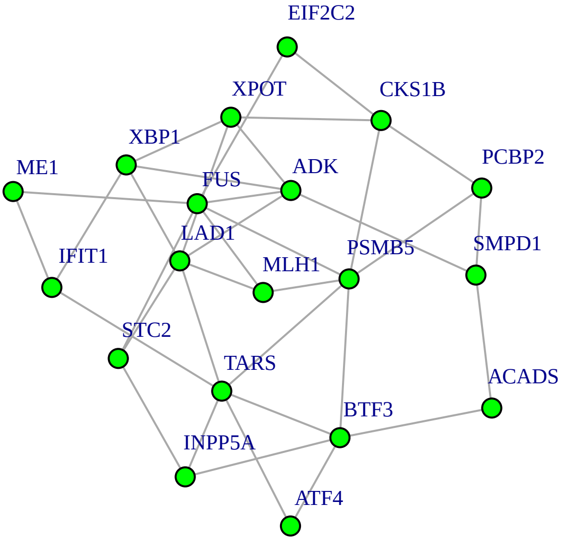

MST2 of the coexpression network for NEG ALL

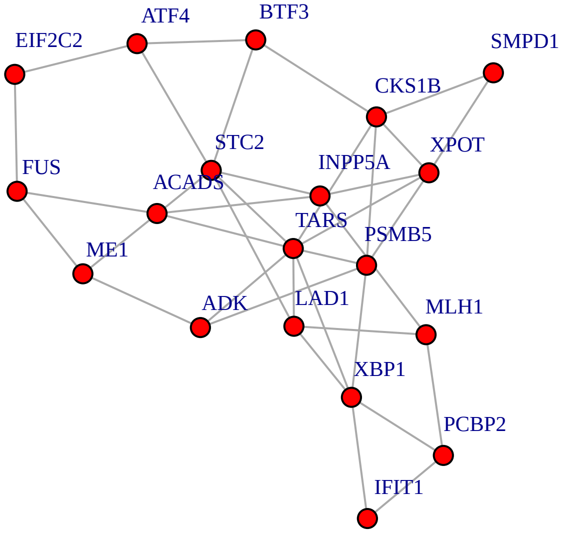

Pathway: CUI_TCF21_TARGETS_UP

There are 29 genes in this pathway. This pathway was detected by GSNCA

BCR/ABL ALL
Major Gene (BCR/ABL): COL6A1
Weight Factor: 1.271
Major Gene (NEG): COL6A2
Weight Factor: 1.249

NEG ALL
Major Gene (NEG): COL6A2
Weight Factor: 1.394
Major Gene (BCR/ABL): COL6A1
Weight Factor: 1.366

MST2 of the coexpression network for BCR/ABL ALL

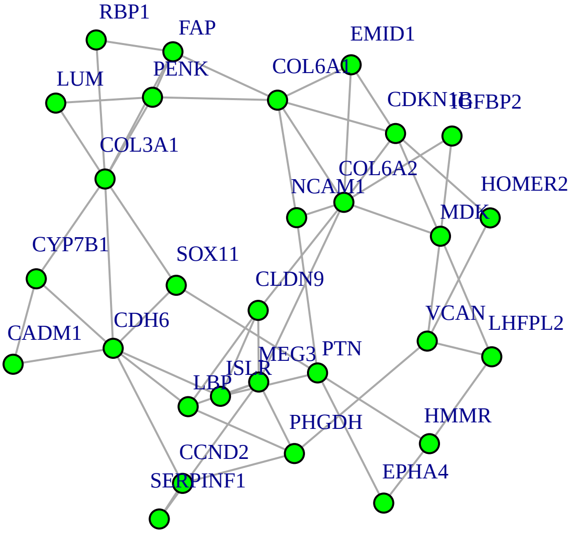

MST2 of the coexpression network for NEG ALL

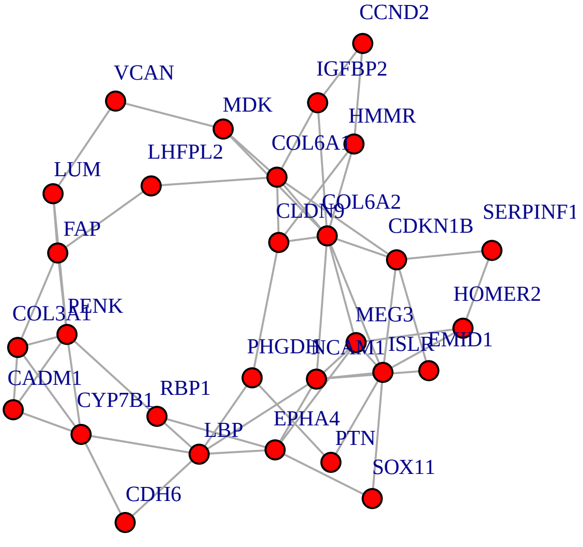

Pathway: ABE_VEGFA_TARGETS_30MIN

There are 20 genes in this pathway. This pathway was detected by GSNCA

BCR/ABL ALL
Major Gene (BCR/ABL): PCDH17
Weight Factor: 1.444
Major Gene (NEG): MYO7A
Weight Factor: 0.812

NEG ALL
Major Gene (NEG): MYO7A
Weight Factor: 1.274
Major Gene (BCR/ABL): PCDH17
Weight Factor: 0.577

MST2 of the coexpression network for BCR/ABL ALL

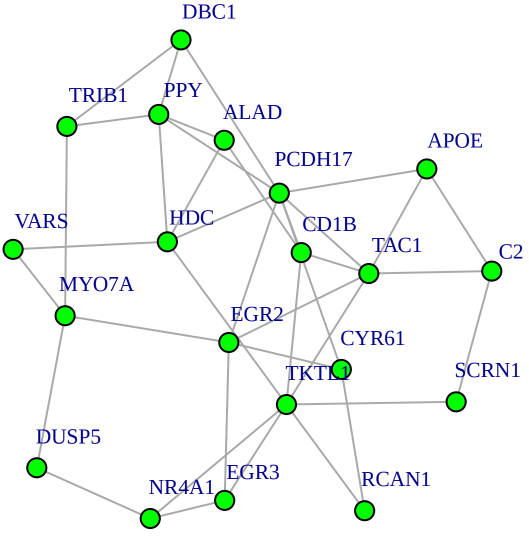

MST2 of the coexpression network for NEG ALL

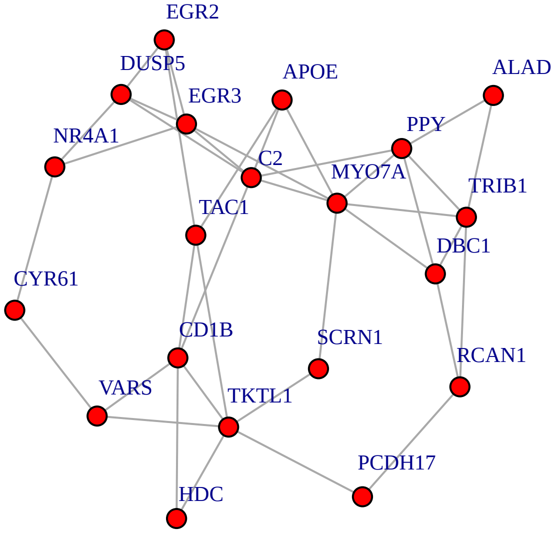

Pathway: KEGG_CITRATE_CYCLE_TCA_CYCLE

There are 28 genes in this pathway. This pathway was detected by GSNCA

BCR/ABL ALL
Major Gene (BCR/ABL): FH
Weight Factor: 1.317
Major Gene (NEG): MDH1
Weight Factor: 1.267

NEG ALL
Major Gene (NEG): MDH1
Weight Factor: 1.426
Major Gene (BCR/ABL): FH
Weight Factor: 1.298

MST2 of the coexpression network for BCR/ABL ALL

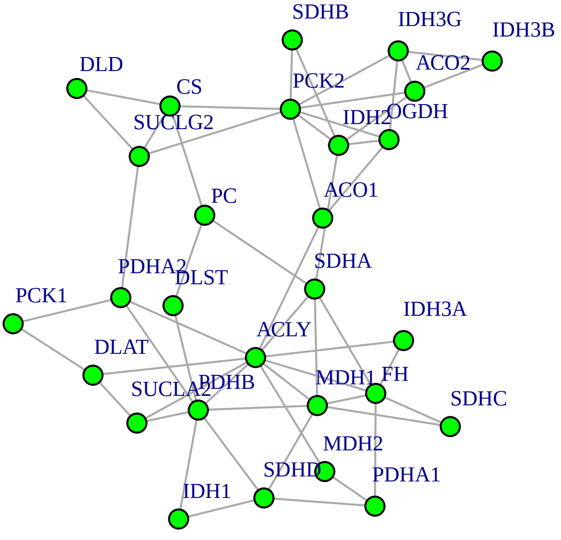

MST2 of the coexpression network for NEG ALL

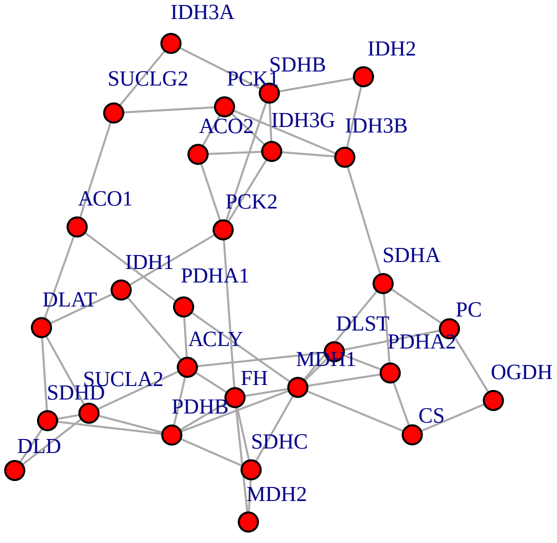

Pathway: KEGG_RNA_POLYMERASE

There are 17 genes in this pathway. This pathway was detected by GSNCA

BCR/ABL ALL
Major Gene (BCR/ABL): POLR2L
Weight Factor: 1.384
Major Gene (NEG): POLR2E
Weight Factor: 1.161

NEG ALL
Major Gene (NEG): POLR2E
Weight Factor: 1.307
Major Gene (BCR/ABL): POLR2L
Weight Factor: 1.15

MST2 of the coexpression network for BCR/ABL ALL

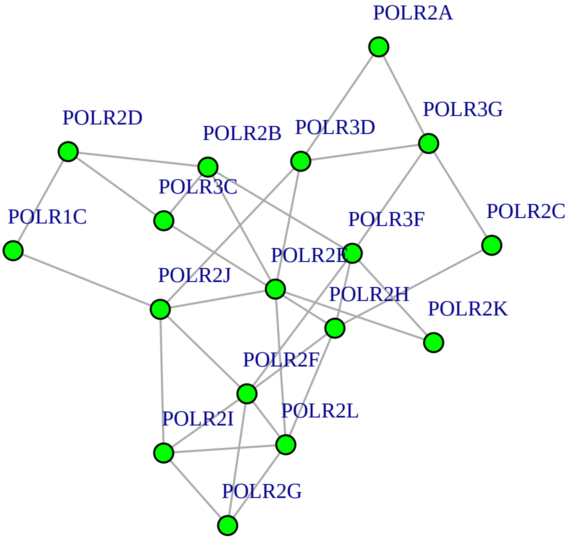

MST2 of the coexpression network for NEG ALL

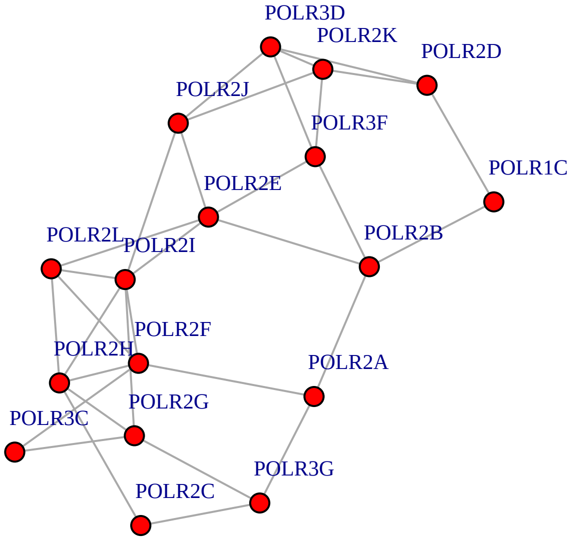

Pathway: KEGG_PROTEASOME

There are 41 genes in this pathway. This pathway was detected by GSNCA

BCR/ABL ALL
Major Gene (BCR/ABL): PSMA4
Weight Factor: 1.251
Major Gene (NEG): PSMB4
Weight Factor: 0.951

NEG ALL
Major Gene (NEG): PSMB4
Weight Factor: 1.305
Major Gene (BCR/ABL): PSMA4
Weight Factor: 1.232

MST2 of the coexpression network for BCR/ABL ALL

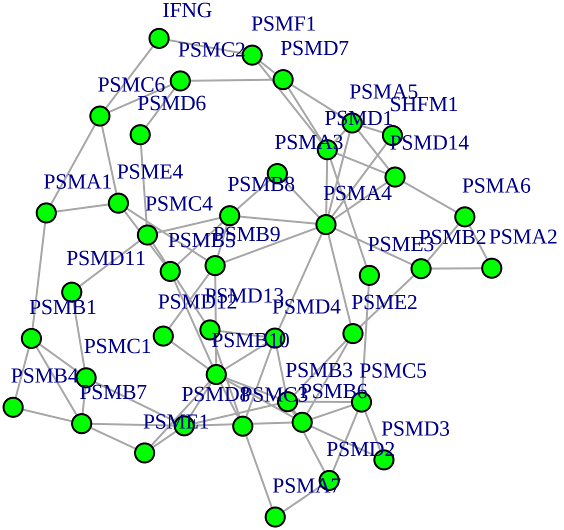

MST2 of the coexpression network for NEG ALL

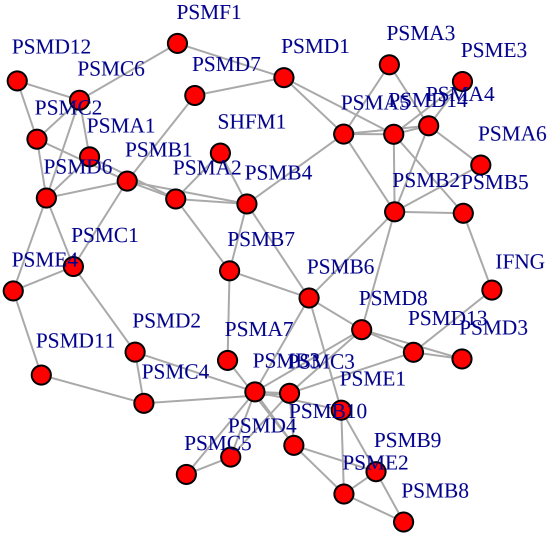

Pathway: KEGG_PROSTATE_CANCER

There are 82 genes in this pathway. This pathway was detected by GSNCA

BCR/ABL ALL
Major Gene (BCR/ABL):   INS
Weight Factor: 1.524
Major Gene (NEG):   KLK3
Weight Factor: 1.511

NEG ALL
Major Gene (NEG):   KLK3
Weight Factor: 1.559
Major Gene (BCR/ABL):   INS
Weight Factor: 1.499

MST2 of the coexpression network for BCR/ABL ALL

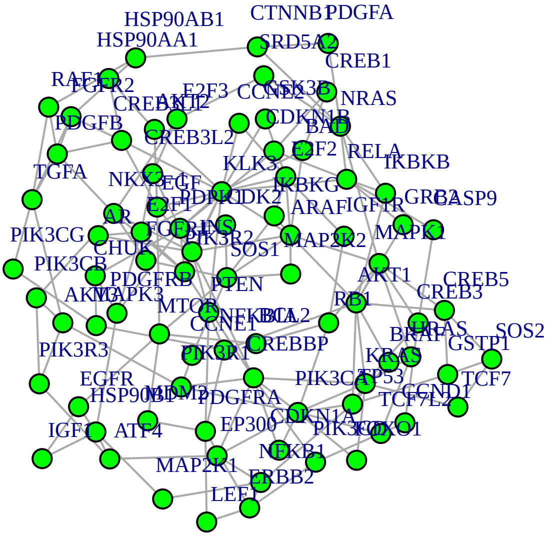

MST2 of the coexpression network for NEG ALL

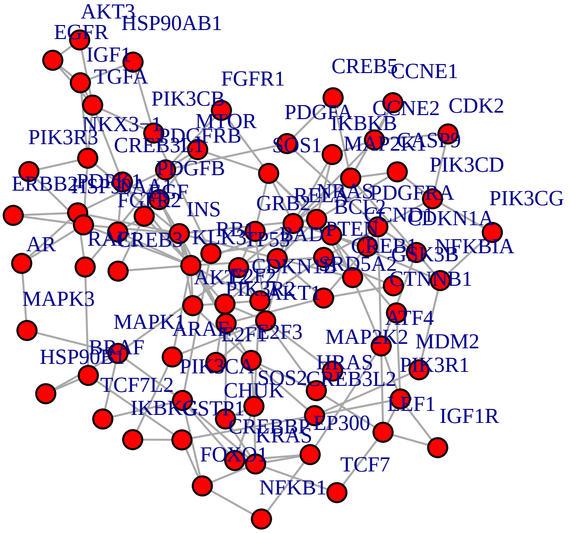

Pathway: KEGG_MELANOMA

There are 58 genes in this pathway. This pathway was detected by GSNCA

BCR/ABL ALL
Major Gene (BCR/ABL): ARAF
Weight Factor: 1.468
Major Gene (NEG): BAD
Weight Factor: 1.231

NEG ALL
Major Gene (NEG): BAD
Weight Factor: 1.47
Major Gene (BCR/ABL): ARAF
Weight Factor: 1.214

MST2 of the coexpression network for BCR/ABL ALL

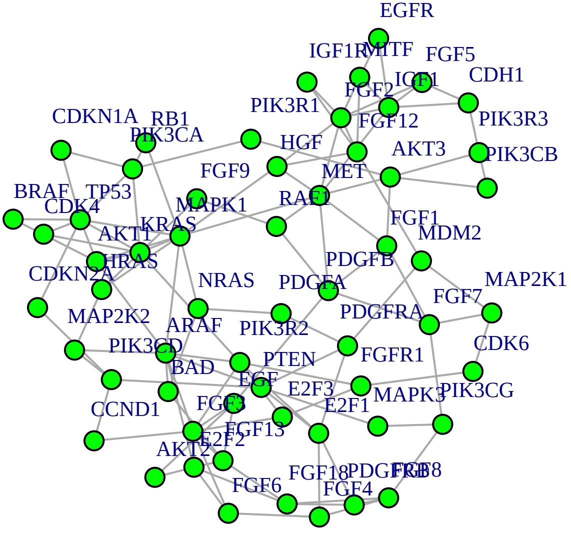

MST2 of the coexpression network for NEG ALL

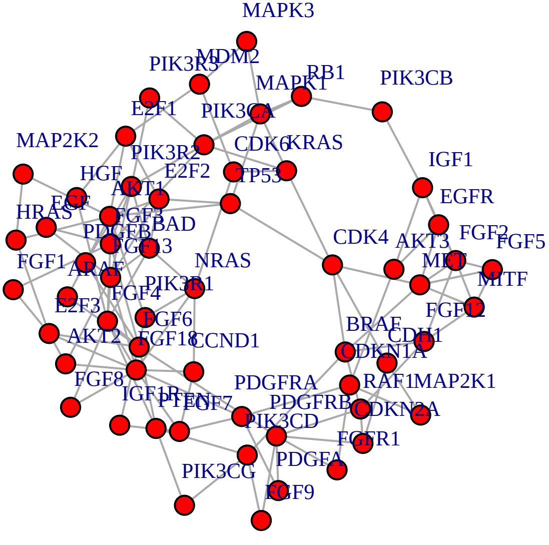

Pathway: KEGG_CHRONIC_MYELOID_LEUKEMIA

There are 70 genes in this pathway. This pathway was detected by GSNCA

BCR/ABL ALL
Major Gene (BCR/ABL): ARAF
Weight Factor: 1.446
Major Gene (NEG): BCL2L1
Weight Factor: 1.406

NEG ALL
Major Gene (NEG): BCL2L1
Weight Factor: 1.469
Major Gene (BCR/ABL): ARAF
Weight Factor: 1.201

MST2 of the coexpression network for BCR/ABL ALL

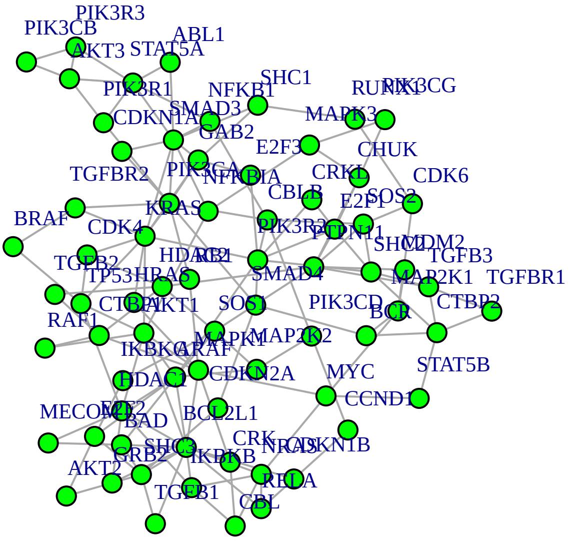

MST2 of the coexpression network for NEG ALL

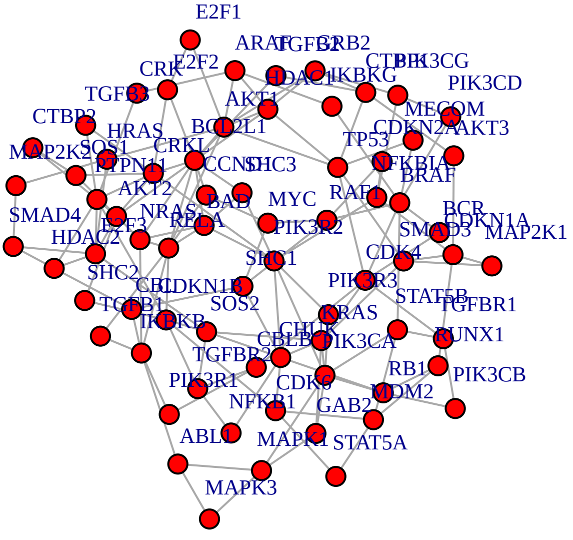

Pathway: BIOCARTA_AKT_PATHWAY

There are 22 genes in this pathway. This pathway was detected by GSNCA

BCR/ABL ALL
Major Gene (BCR/ABL): PDPK1
Weight Factor: 1.359
Major Gene (NEG): PPP2CA
Weight Factor: 1.19

NEG ALL
Major Gene (NEG): PPP2CA
Weight Factor: 1.269
Major Gene (BCR/ABL): PDPK1
Weight Factor: 1.259

MST2 of the coexpression network for BCR/ABL ALL

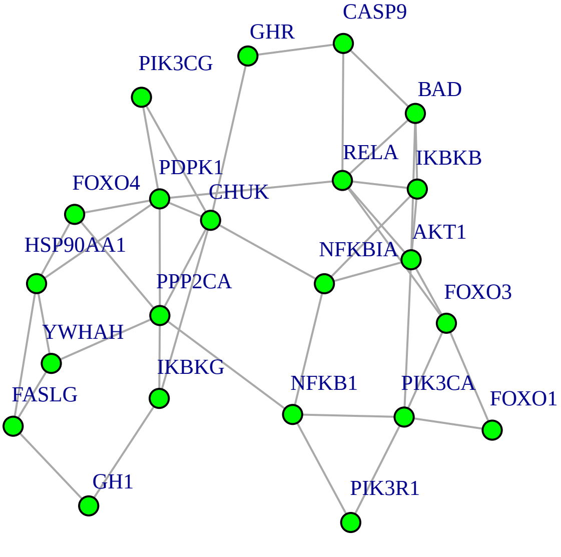

MST2 of the coexpression network for NEG ALL

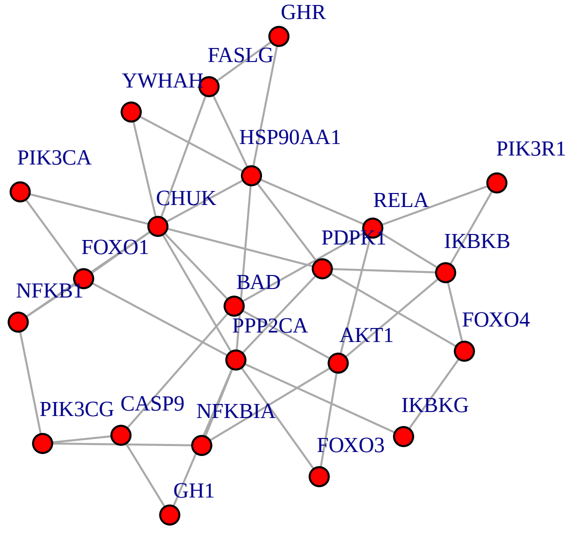

Pathway: BIOCARTA_BCELLSURVIVAL_PATHWAY

There are 16 genes in this pathway. This pathway was detected by GSNCA

BCR/ABL ALL
Major Gene (BCR/ABL): PIK3CA
Weight Factor: 1.483
Major Gene (NEG): ZBTB7A
Weight Factor: 0.961

NEG ALL
Major Gene (NEG): ZBTB7A
Weight Factor: 1.478
Major Gene (BCR/ABL): PIK3CA
Weight Factor: 0.927

MST2 of the coexpression network for BCR/ABL ALL

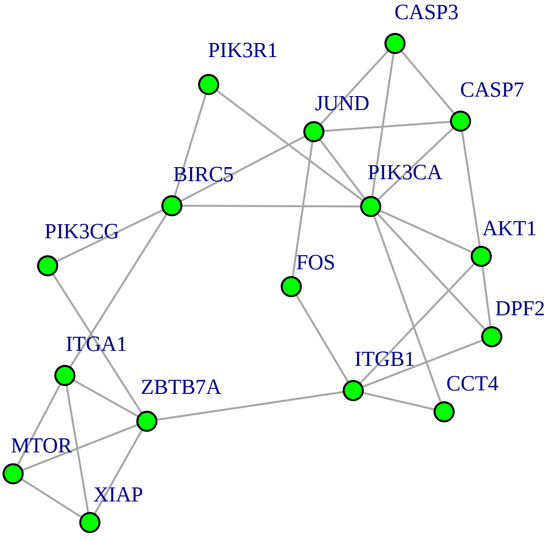

MST2 of the coexpression network for NEG ALL

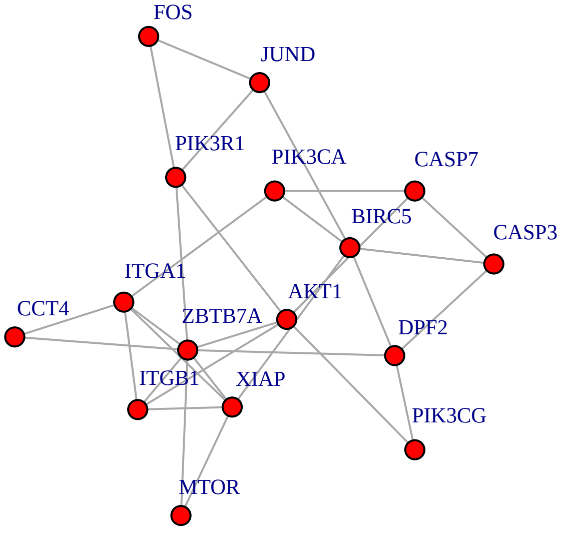

Pathway: BIOCARTA_NTHI_PATHWAY

There are 24 genes in this pathway. This pathway was detected by GSNCA

BCR/ABL ALL
Major Gene (BCR/ABL): SMAD3
Weight Factor: 1.37
Major Gene (NEG): RELA
Weight Factor: 0.749

NEG ALL
Major Gene (NEG): RELA
Weight Factor: 1.318
Major Gene (BCR/ABL): SMAD3
Weight Factor: 0.637

MST2 of the coexpression network for BCR/ABL ALL

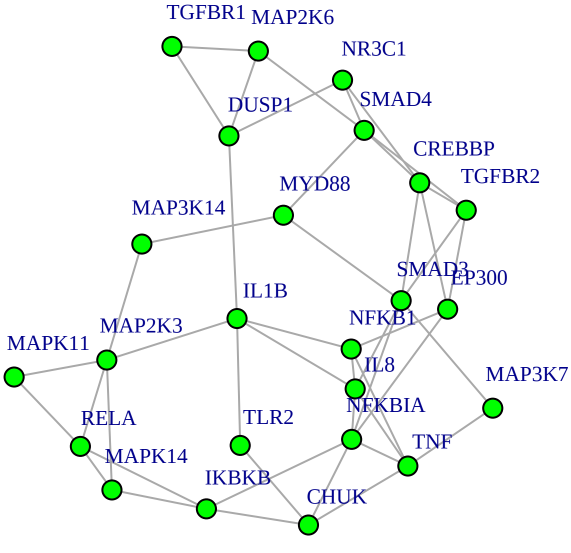

MST2 of the coexpression network for NEG ALL

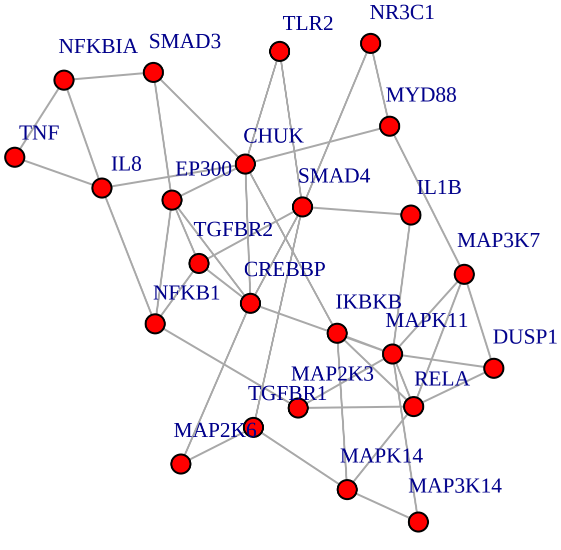

Pathway: BIOCARTA_MITOCHONDRIA_PATHWAY

There are 20 genes in this pathway. This pathway was detected by GSNCA

BCR/ABL ALL
Major Gene (BCR/ABL): CASP8
Weight Factor: 1.341
Major Gene (NEG): ENDOG
Weight Factor: 1.042

NEG ALL
Major Gene (NEG): ENDOG
Weight Factor: 1.399
Major Gene (BCR/ABL): CASP8
Weight Factor: 1.147

MST2 of the coexpression network for BCR/ABL ALL

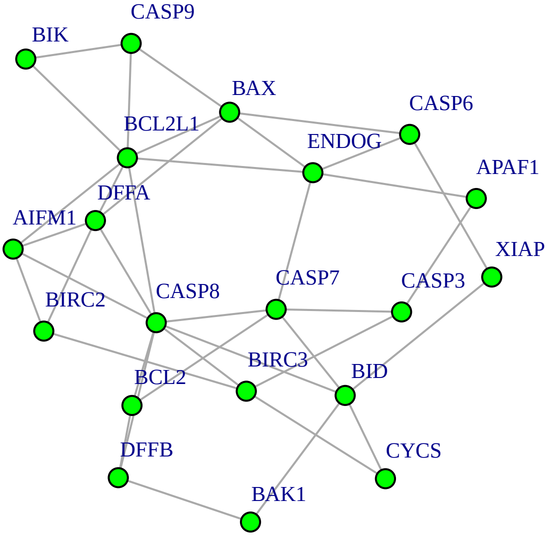

MST2 of the coexpression network for NEG ALL

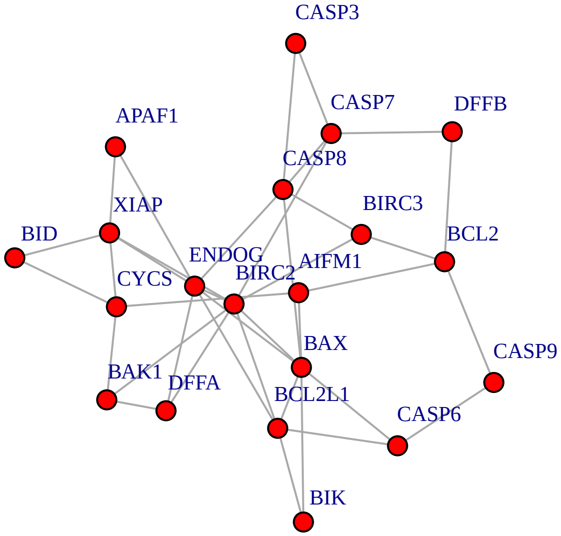

Pathway: BIOCARTA_TEL_PATHWAY

There are 18 genes in this pathway. This pathway was detected by GSNCA

BCR/ABL ALL
Major Gene (BCR/ABL): KRAS
Weight Factor: 1.289
Major Gene (NEG): TEP1
Weight Factor: 0.943

NEG ALL
Major Gene (NEG): TEP1
Weight Factor: 1.419
Major Gene (BCR/ABL): KRAS
Weight Factor: 1.038

MST2 of the coexpression network for BCR/ABL ALL

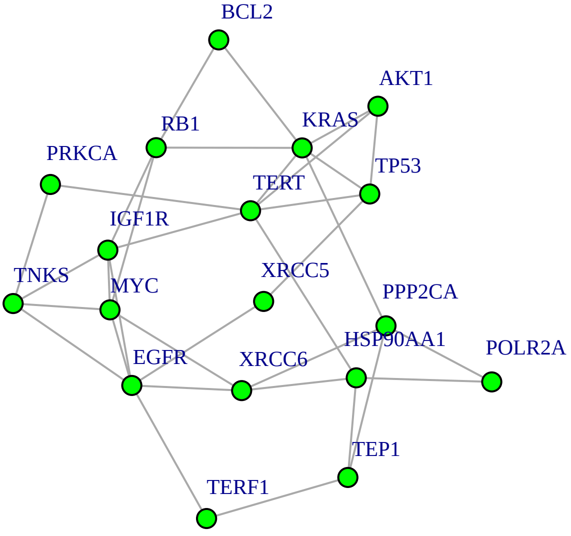

MST2 of the coexpression network for NEG ALL

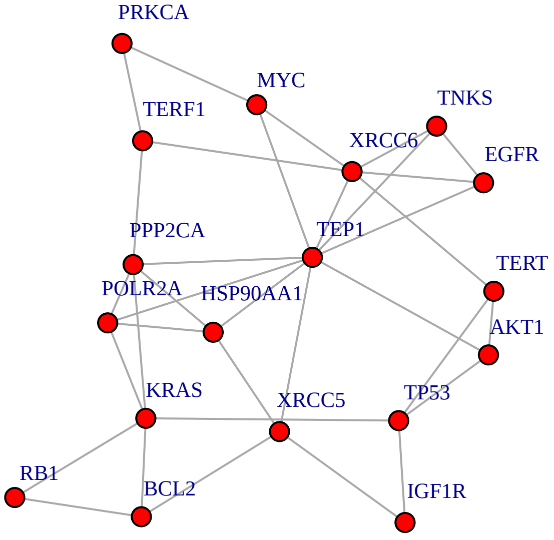

Pathway: BIOCARTA_TNFR2_PATHWAY

There are 18 genes in this pathway. This pathway was detected by GSNCA

BCR/ABL ALL
Major Gene (BCR/ABL): IKBKAP
Weight Factor: 1.343
Major Gene (NEG): IKBKB
Weight Factor: 1.157

NEG ALL
Major Gene (NEG): IKBKB
Weight Factor: 1.383
Major Gene (BCR/ABL): IKBKAP
Weight Factor: 1.147

MST2 of the coexpression network for BCR/ABL ALL

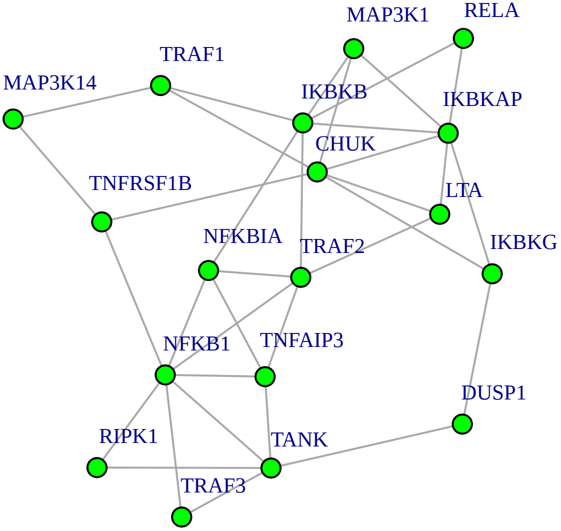

MST2 of the coexpression network for NEG ALL

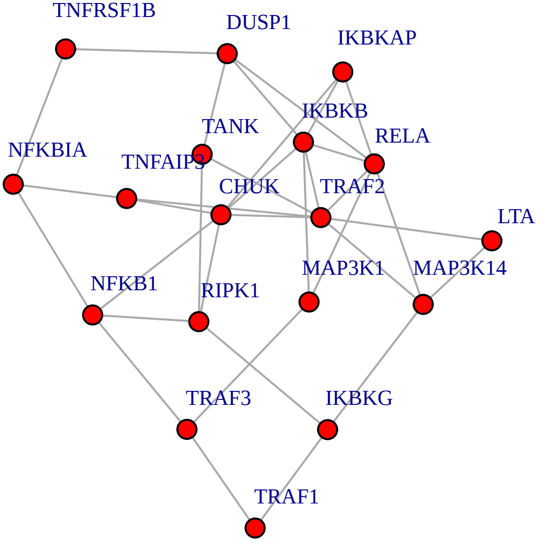

Pathway: BIOCARTA_TOLL_PATHWAY

There are 30 genes in this pathway. This pathway was detected by GSNCA

BCR/ABL ALL
Major Gene (BCR/ABL): RELA
Weight Factor: 1.326
Major Gene (NEG): PGLYRP1
Weight Factor: 1.274

NEG ALL
Major Gene (NEG): PGLYRP1
Weight Factor: 1.496
Major Gene (BCR/ABL): RELA
Weight Factor: 1.451

MST2 of the coexpression network for BCR/ABL ALL

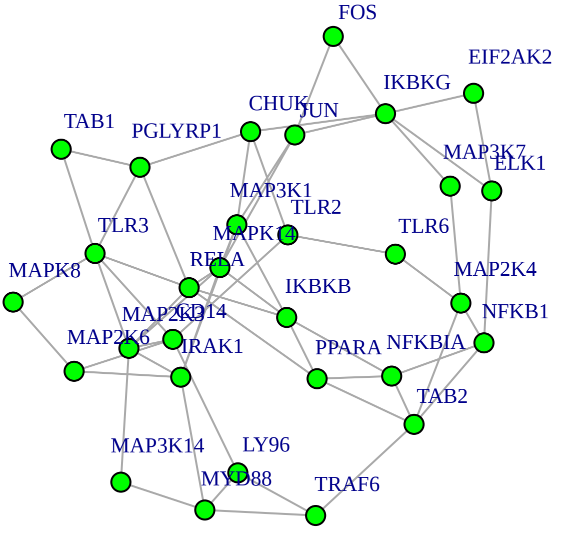

MST2 of the coexpression network for NEG ALL

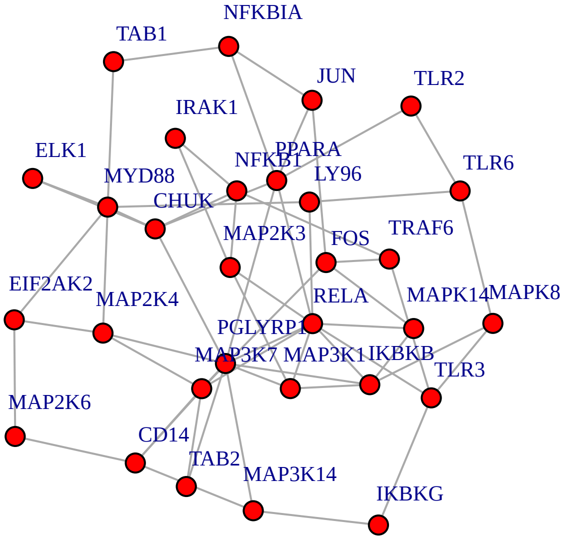

Pathway: REACTOME_AUTODEGRADATION_OF_CDH1_BY_CDH1_APC

There are 51 genes in this pathway. This pathway was detected by GSNCA

BCR/ABL ALL
Major Gene (BCR/ABL): PSMA4
Weight Factor: 1.312
Major Gene (NEG): PSMB4
Weight Factor: 0.989

NEG ALL
Major Gene (NEG): PSMB4
Weight Factor: 1.343
Major Gene (BCR/ABL): PSMA4
Weight Factor: 1.295

MST2 of the coexpression network for BCR/ABL ALL

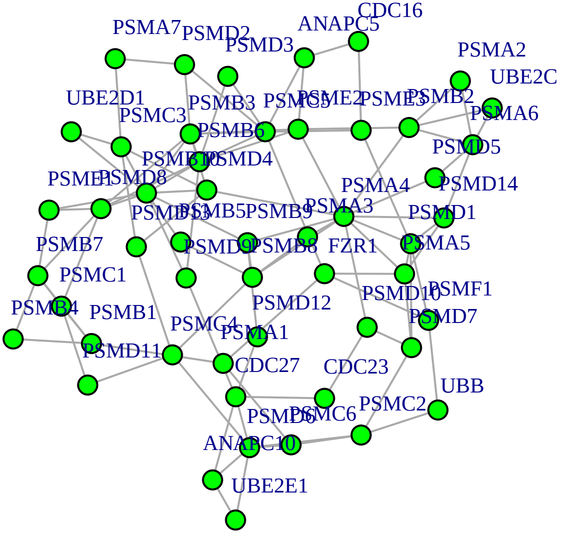

MST2 of the coexpression network for NEG ALL

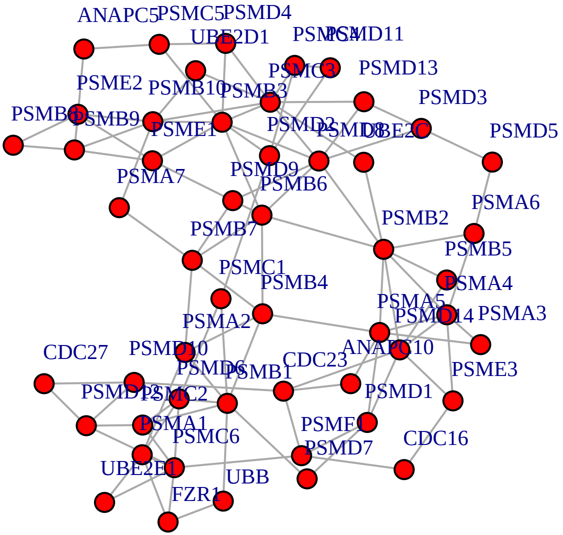

Pathway: REACTOME_CD28_CO_STIMULATION

There are 25 genes in this pathway. This pathway was detected by GSNCA

BCR/ABL ALL
Major Gene (BCR/ABL): MAPKAP1
Weight Factor: 1.53
Major Gene (NEG): MAPKAP1
Weight Factor: 1.53

NEG ALL
Major Gene (NEG): MAPKAP1
Weight Factor: 1.515
Major Gene (BCR/ABL): MAPKAP1
Weight Factor: 1.515

MST2 of the coexpression network for BCR/ABL ALL

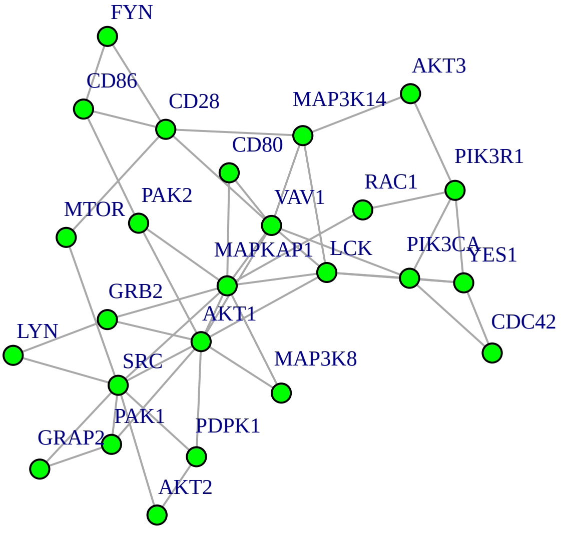

MST2 of the coexpression network for NEG ALL

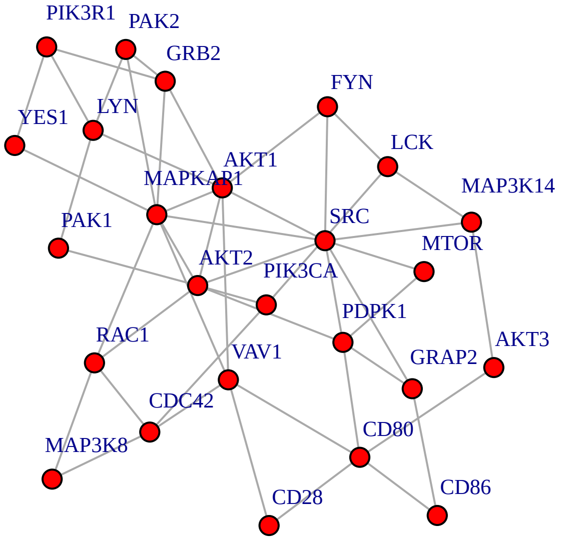

Pathway: REACTOME_CDC20_PHOSPHO_APC_MEDIATED_DEGRADATION_OF_CYCLIN_A

There are 57 genes in this pathway. This pathway was detected by GSNCA

BCR/ABL ALL
Major Gene (BCR/ABL): PSMA4
Weight Factor: 1.34
Major Gene (NEG): PSMA4
Weight Factor: 1.34

NEG ALL
Major Gene (NEG): PSMA4
Weight Factor: 1.321
Major Gene (BCR/ABL): PSMA4
Weight Factor: 1.321

MST2 of the coexpression network for BCR/ABL ALL

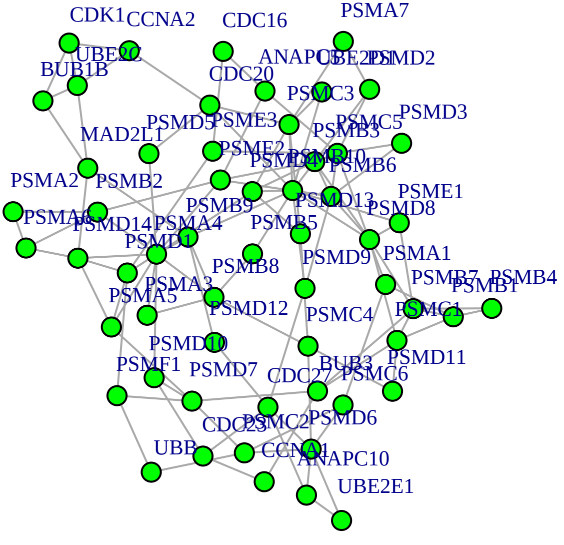

MST2 of the coexpression network for NEG ALL

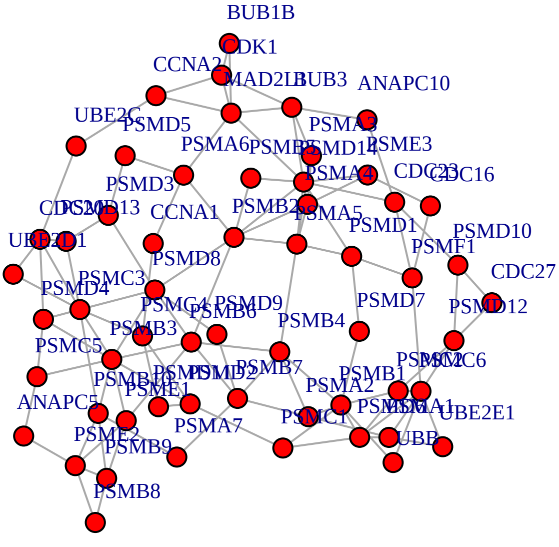

Pathway: REACTOME_CELL_CYCLE_CHECKPOINTS

There are 91 genes in this pathway. This pathway was detected by GSNCA

BCR/ABL ALL
Major Gene (BCR/ABL): PSMA4
Weight Factor: 1.393
Major Gene (NEG): MCM6
Weight Factor: 1.155

NEG ALL
Major Gene (NEG): MCM6
Weight Factor: 1.427
Major Gene (BCR/ABL): PSMA4
Weight Factor: 1.304

MST2 of the coexpression network for BCR/ABL ALL

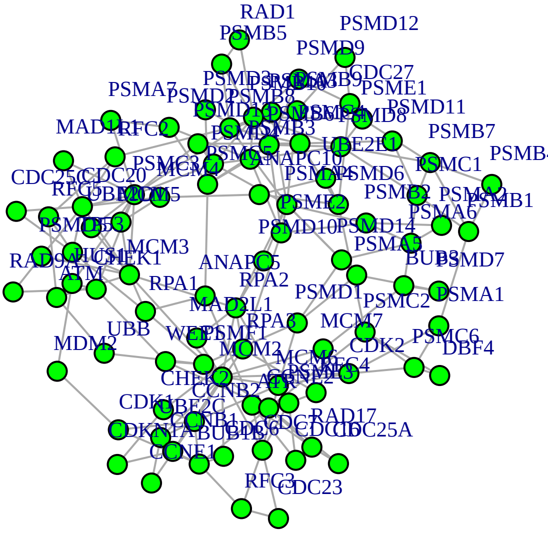

MST2 of the coexpression network for NEG ALL

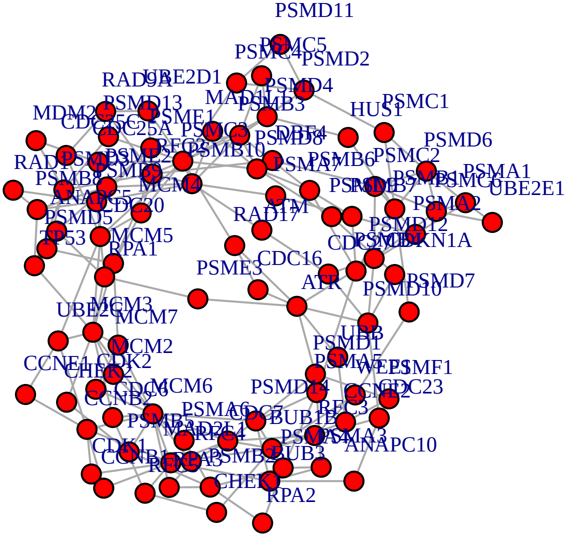

Pathway: REACTOME_CHAPERONIN_MEDIATED_PROTEIN_FOLDING

There are 28 genes in this pathway. This pathway was detected by GSNCA

BCR/ABL ALL
Major Gene (BCR/ABL): CCT3
Weight Factor: 1.458
Major Gene (NEG): CCT5
Weight Factor: 1.397

MST2 of the coexpression network for BCR/ABL ALL

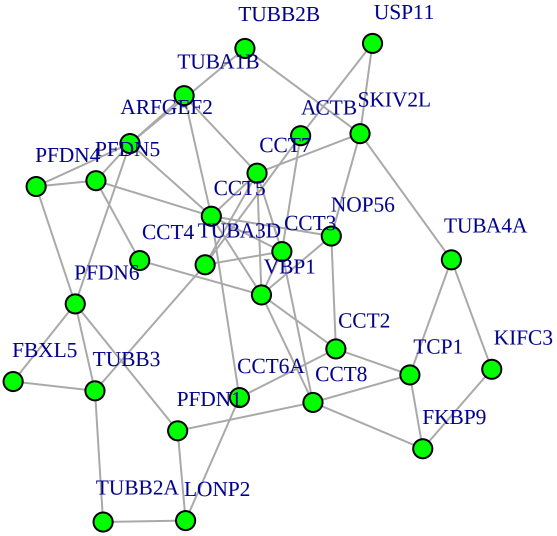

NEG ALL
Major Gene (NEG): CCT5
Weight Factor: 1.36
Major Gene (BCR/ABL): CCT3
Weight Factor: 1.263

MST2 of the coexpression network for NEG ALL

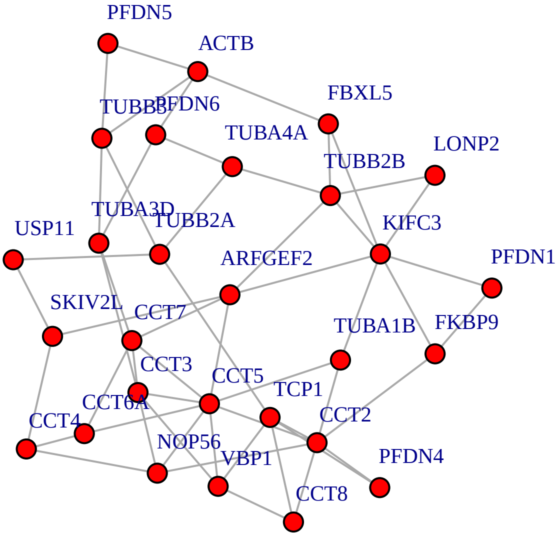

Pathway: REACTOME_CITRIC_ACID_CYCLE

There are 18 genes in this pathway. This pathway was detected by GSNCA

BCR/ABL ALL
Major Gene (BCR/ABL): IDH2
Weight Factor: 1.263
Major Gene (NEG): NNT
Weight Factor: 1.124

NEG ALL
Major Gene (NEG): NNT
Weight Factor: 1.306
Major Gene (BCR/ABL): IDH2
Weight Factor: 0.482

MST2 of the coexpression network for BCR/ABL ALL

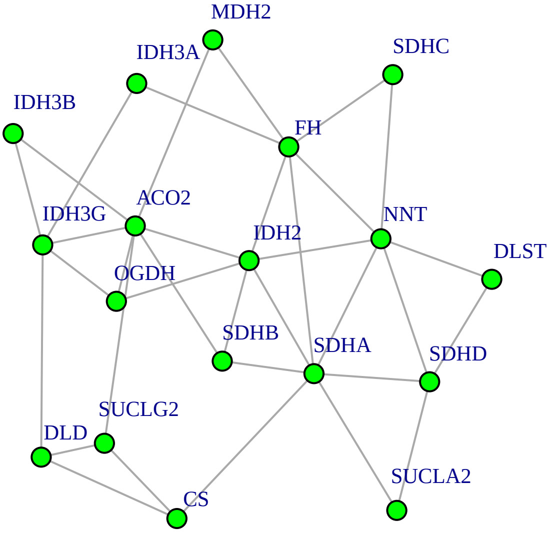

MST2 of the coexpression network for NEG ALL

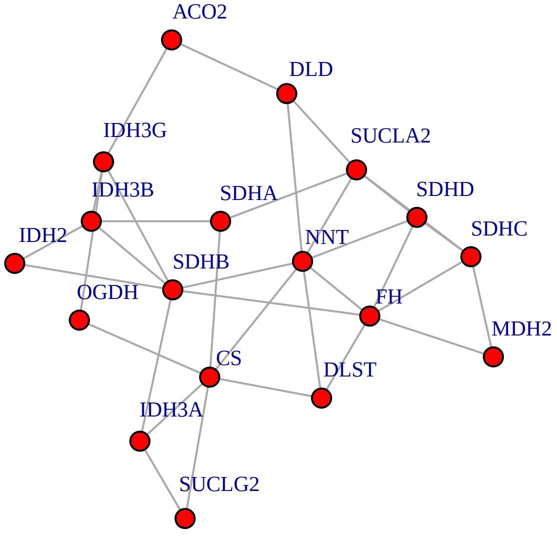

Pathway: REACTOME_CYCLIN_E_ASSOCIATED_EVENTS_DURING_G1_S_TRANSITION_

There are 58 genes in this pathway. This pathway was detected by GSNCA

BCR/ABL ALL
Major Gene (BCR/ABL): PSMB6
Weight Factor: 1.363
Major Gene (NEG): PSMB4
Weight Factor: 1.018

NEG ALL
Major Gene (NEG): PSMB4
Weight Factor: 1.365
Major Gene (BCR/ABL): PSMB6
Weight Factor: 1.214

MST2 of the coexpression network for BCR/ABL ALL

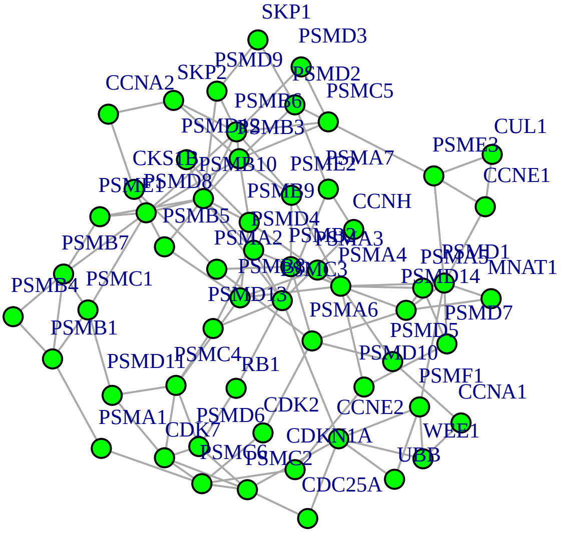

MST2 of the coexpression network for NEG ALL

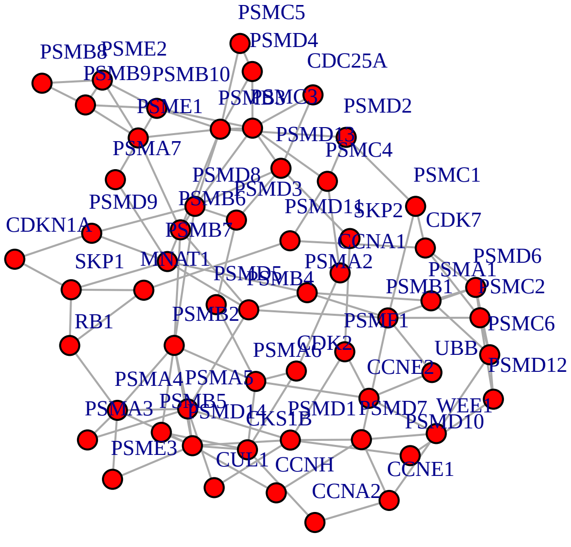

Pathway: REACTOME_DOUBLE_STRAND_BREAK_REPAIR

There are 20 genes in this pathway. This pathway was detected by GSNCA

BCR/ABL ALL
Major Gene (BCR/ABL): RPA2
Weight Factor: 1.342
Major Gene (NEG): RPA2
Weight Factor: 1.342

NEG ALL
Major Gene (NEG): RPA2
Weight Factor: 1.35
Major Gene (BCR/ABL): RPA2
Weight Factor: 1.35

MST2 of the coexpression network for BCR/ABL ALL

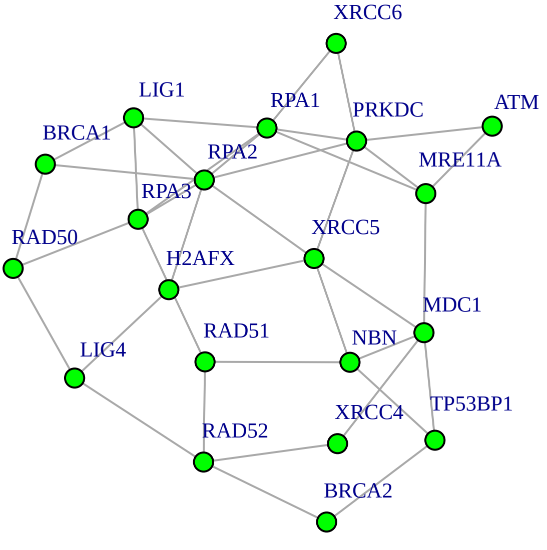

MST2 of the coexpression network for NEG ALL

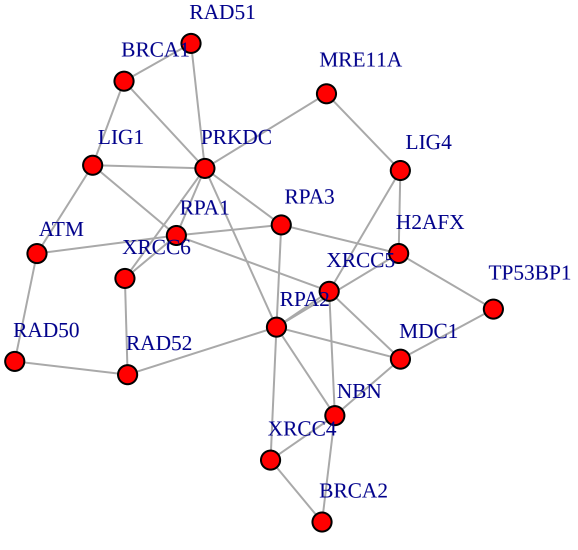

Pathway: REACTOME_GLYCOGEN_BREAKDOWN_GLYCOGENOLYSIS

There are 15 genes in this pathway. This pathway was detected by GSNCA

BCR/ABL ALL
Major Gene (BCR/ABL): AGL
Weight Factor: 1.323
Major Gene (NEG): PHKG1
Weight Factor: 0.885

NEG ALL
Major Gene (NEG): PHKG1
Weight Factor: 1.374
Major Gene (BCR/ABL): AGL
Weight Factor: 1.013

MST2 of the coexpression network for BCR/ABL ALL

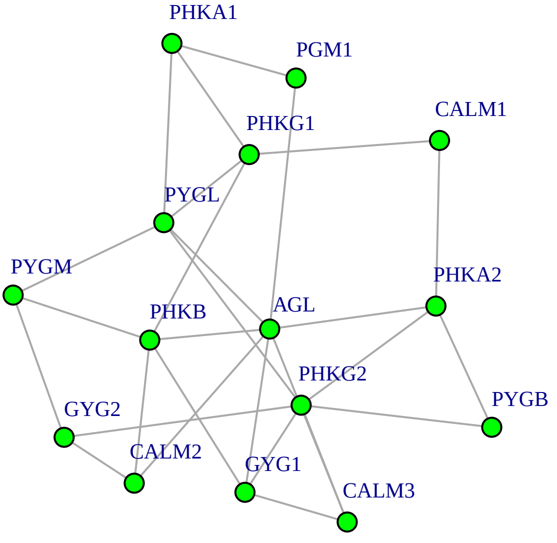

MST2 of the coexpression network for NEG ALL

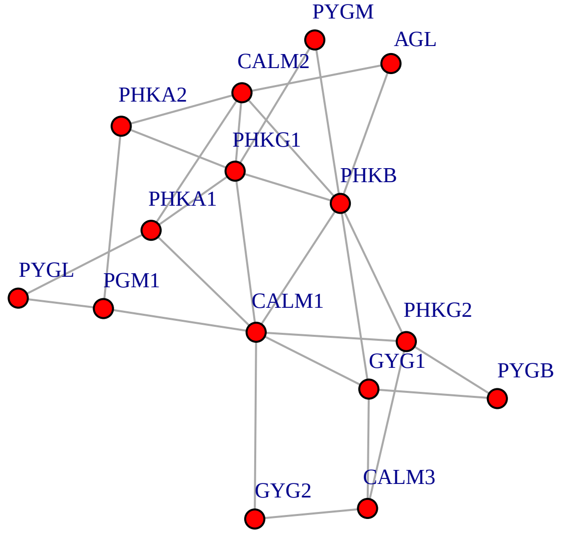

Pathway: REACTOME_HIV_INFECTION

There are 167 genes in this pathway. This pathway was detected by GSNCA

BCR/ABL ALL
Major Gene (BCR/ABL): PSMB10
Weight Factor: 1.51
Major Gene (NEG): NUP205
Weight Factor: 0.846

NEG ALL
Major Gene (NEG): NUP205
Weight Factor: 1.329
Major Gene (BCR/ABL): PSMB10
Weight Factor: 1.133

MST2 of the coexpression network for BCR/ABL ALL

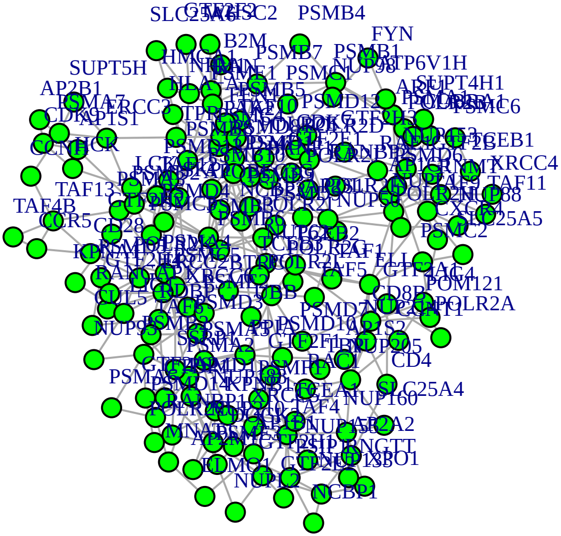

MST2 of the coexpression network for NEG ALL

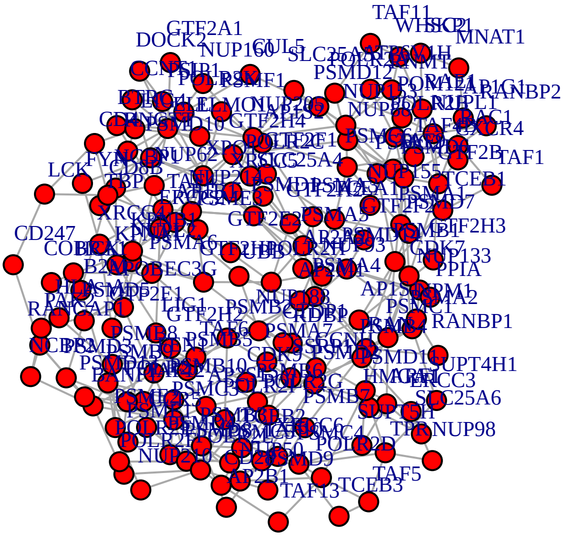

Pathway: REACTOME_HOMOLOGOUS_RECOMBINATION_REPAIR

There are 15 genes in this pathway. This pathway was detected by GSNCA

BCR/ABL ALL
Major Gene (BCR/ABL): RPA2
Weight Factor: 1.327
Major Gene (NEG): RPA2
Weight Factor: 1.327

NEG ALL
Major Gene (NEG): RPA2
Weight Factor: 1.318
Major Gene (BCR/ABL): RPA2
Weight Factor: 1.318

MST2 of the coexpression network for BCR/ABL ALL

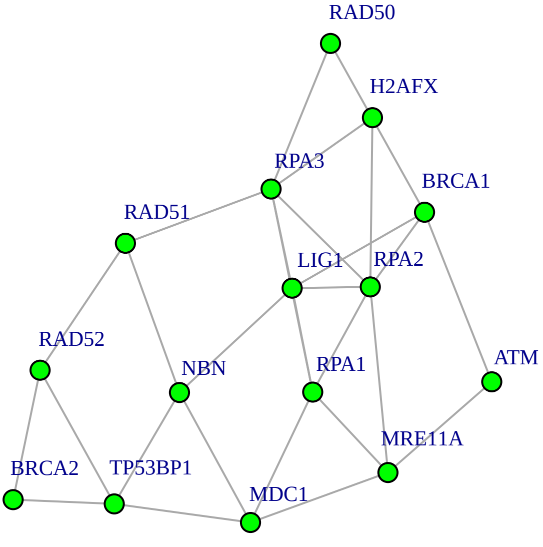

MST2 of the coexpression network for NEG ALL

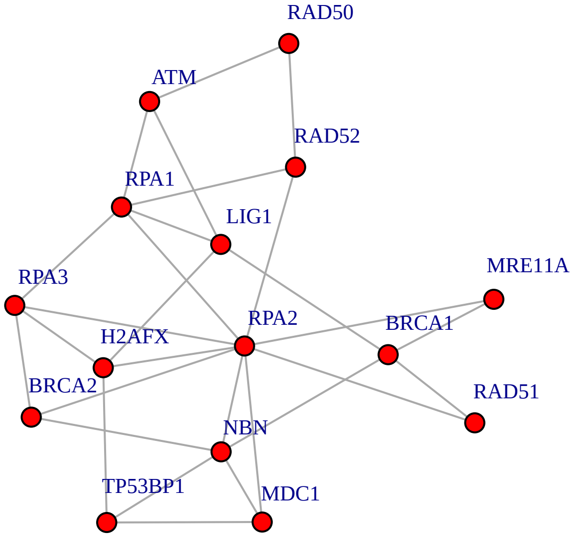

Pathway: REACTOME_HOST_INTERACTIONS_OF_HIV_FACTORS

There are 106 genes in this pathway. This pathway was detected by GSNCA

BCR/ABL ALL
Major Gene (BCR/ABL): PSMB10
Weight Factor: 1.484
Major Gene (NEG): PSMB1
Weight Factor: 1.186

NEG ALL
Major Gene (NEG): PSMB1
Weight Factor: 1.346
Major Gene (BCR/ABL): PSMB10
Weight Factor: 1.064

MST2 of the coexpression network for BCR/ABL ALL

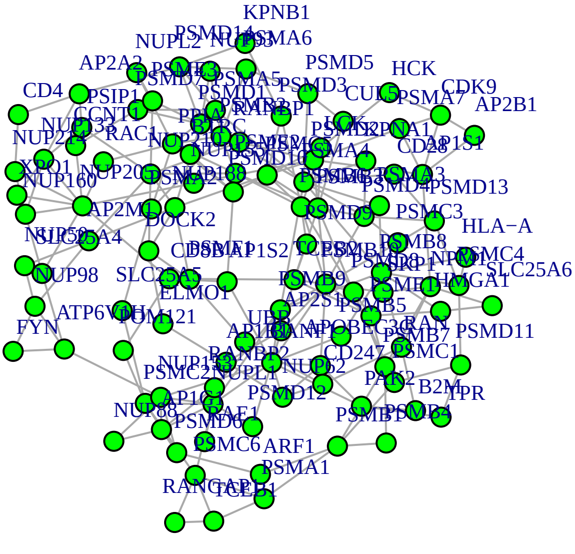

MST2 of the coexpression network for NEG ALL

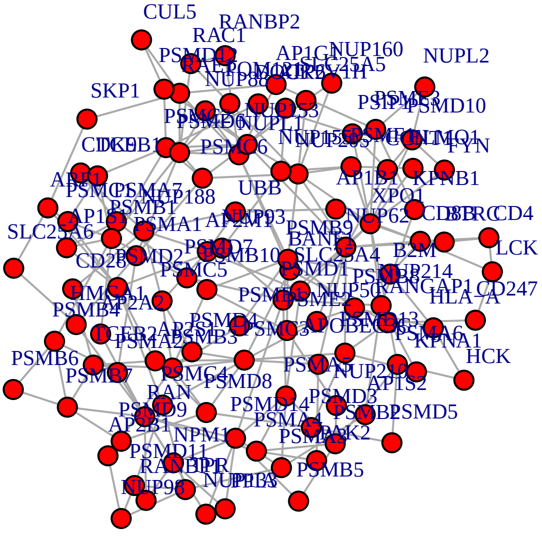

Pathway: REACTOME_PYRUVATE_METABOLISM_AND_TCA_CYCLE

There are 31 genes in this pathway. This pathway was detected by GSNCA

BCR/ABL ALL
Major Gene (BCR/ABL): FH
Weight Factor: 1.321
Major Gene (NEG): PDHB
Weight Factor: 1.01

NEG ALL
Major Gene (NEG): PDHB
Weight Factor: 1.389
Major Gene (BCR/ABL): FH
Weight Factor: 1.315

MST2 of the coexpression network for BCR/ABL ALL

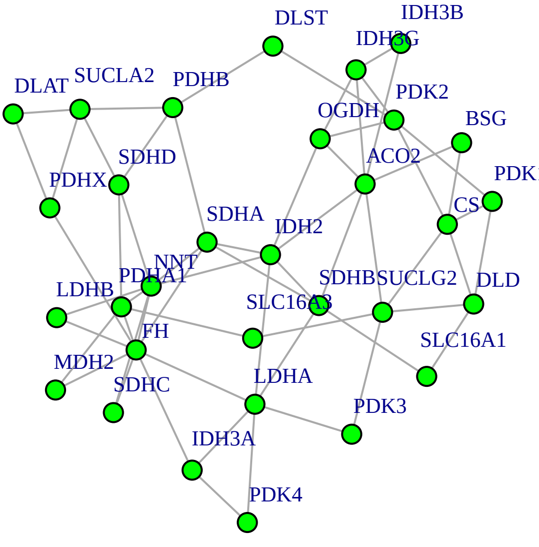

MST2 of the coexpression network for NEG ALL

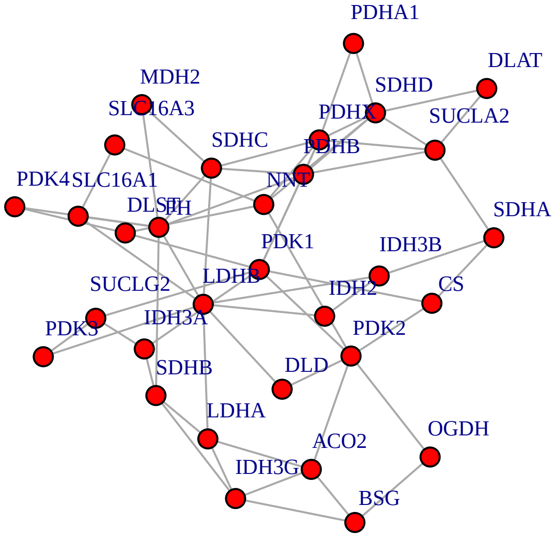

hway: REACTOME_REGULATION_OF_APC_ACTIVATORS_BETWEEN_G1_S_AND_EARLY_ANAPHA

There are 64 genes in this pathway. This pathway was detected by GSNCA

BCR/ABL ALL
Major Gene (BCR/ABL): PSMA4
Weight Factor: 1.381
Major Gene (NEG): PSMA4
Weight Factor: 1.381

NEG ALL
Major Gene (NEG): PSMA4
Weight Factor: 1.334
Major Gene (BCR/ABL): PSMA4
Weight Factor: 1.334

MST2 of the coexpression network for BCR/ABL ALL

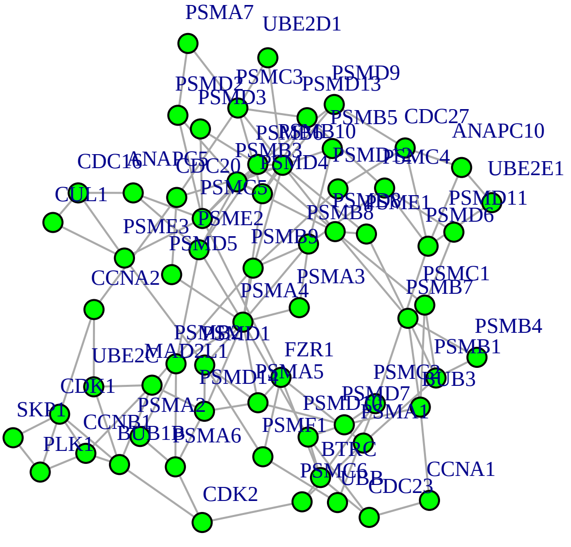

MST2 of the coexpression network for NEG ALL

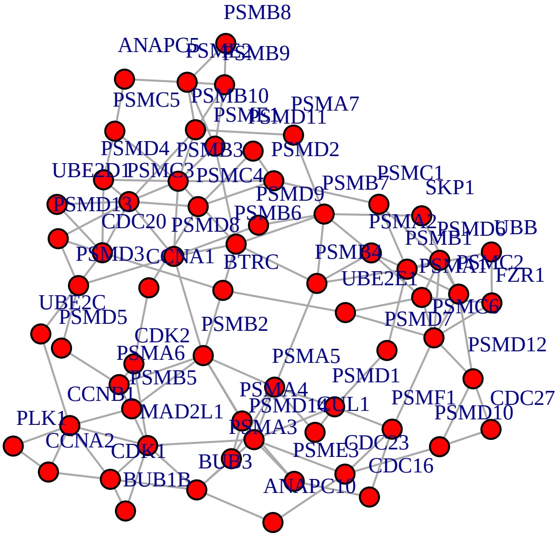

Pathway: REACTOME_SCF_BETA_TRCP_MEDIATED_DEGRADATION_OF_EMI1

There are 47 genes in this pathway. This pathway was detected by GSNCA

BCR/ABL ALL
Major Gene (BCR/ABL): PSMB6
Weight Factor: 1.281
Major Gene (NEG): PSMB4
Weight Factor: 0.967

NEG ALL
Major Gene (NEG): PSMB4
Weight Factor: 1.337
Major Gene (BCR/ABL): PSMB6
Weight Factor: 1.197

MST2 of the coexpression network for BCR/ABL ALL

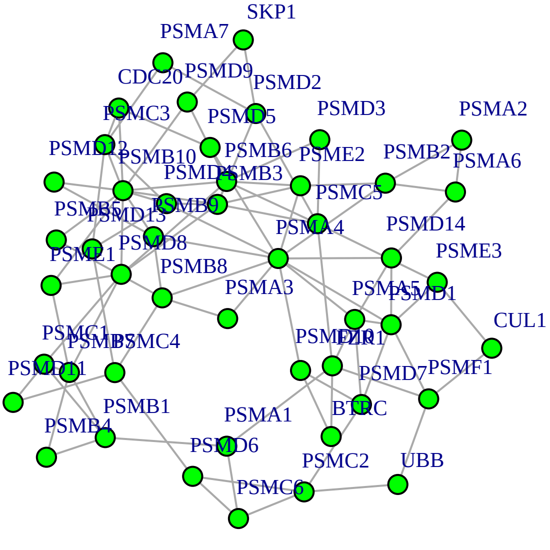

MST2 of the coexpression network for NEG ALL

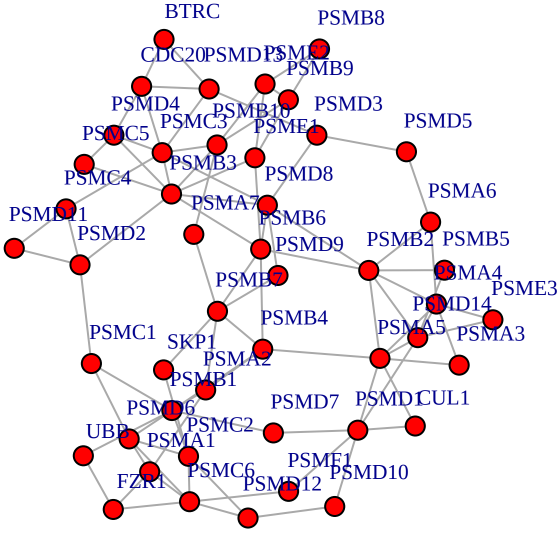

Pathway: REACTOME_SCF_SKP2_MEDIATED_DEGRADATION_OF_P27_P21

There are 52 genes in this pathway. This pathway was detected by GSNCA

BCR/ABL ALL
Major Gene (BCR/ABL): PSMB6
Weight Factor: 1.328
Major Gene (NEG): PSMB4
Weight Factor: 0.996

NEG ALL
Major Gene (NEG): PSMB4
Weight Factor: 1.35
Major Gene (BCR/ABL): PSMB6
Weight Factor: 1.216

MST2 of the coexpression network for BCR/ABL ALL

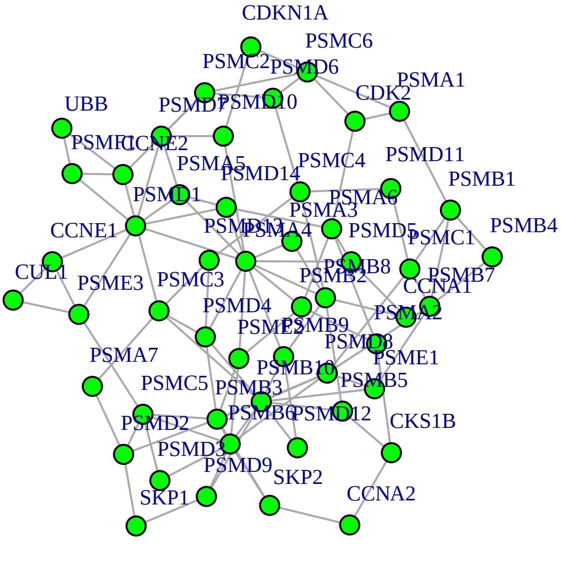

MST2 of the coexpression network for NEG ALL

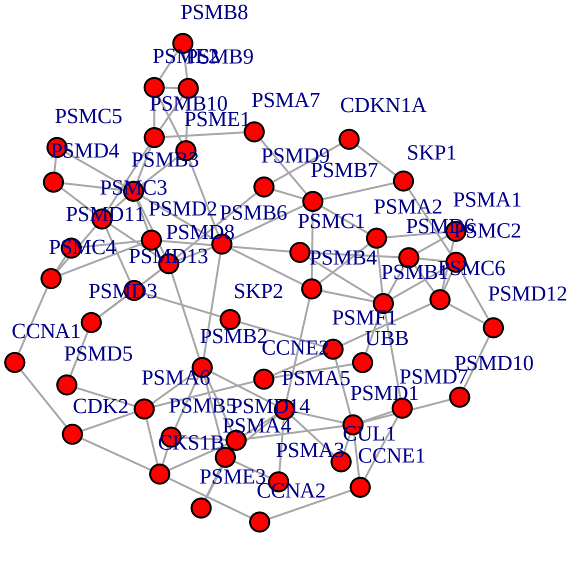

Pathway: REACTOME_SIGNALING_BY_TGF_BETA

There are 15 genes in this pathway. This pathway was detected by GSNCA

BCR/ABL ALL
Major Gene (BCR/ABL): SMAD3
Weight Factor: 1.373
Major Gene (NEG): UBE2D3
Weight Factor: 0.688

NEG ALL
Major Gene (NEG): UBE2D3
Weight Factor: 1.307
Major Gene (BCR/ABL): SMAD3
Weight Factor: 0.755

MST2 of the coexpression network for BCR/ABL ALL

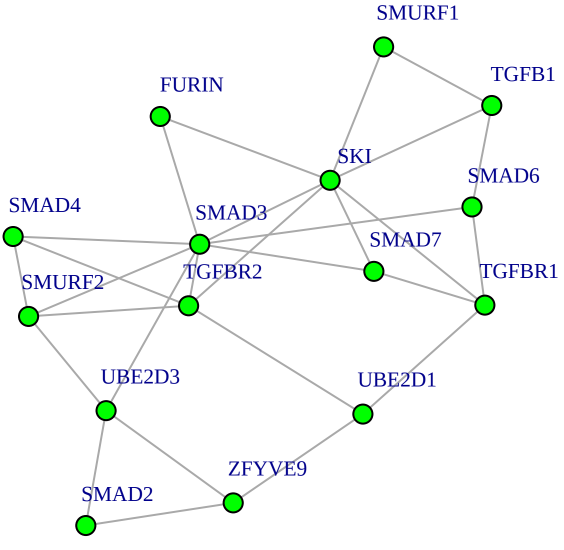

MST2 of the coexpression network for NEG ALL

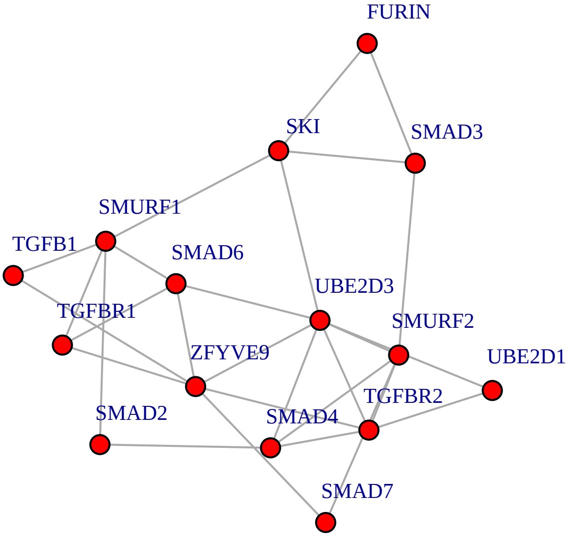

Pathway: REACTOME_SIGNALING_BY_WNT

There are 58 genes in this pathway. This pathway was detected by GSNCA

BCR/ABL ALL
Major Gene (BCR/ABL): PSMB6
Weight Factor: 1.344
Major Gene (NEG): PSMB4
Weight Factor: 1.003

NEG ALL
Major Gene (NEG): PSMB4
Weight Factor: 1.319
Major Gene (BCR/ABL): PSMB6
Weight Factor: 1.166

MST2 of the coexpression network for BCR/ABL ALL

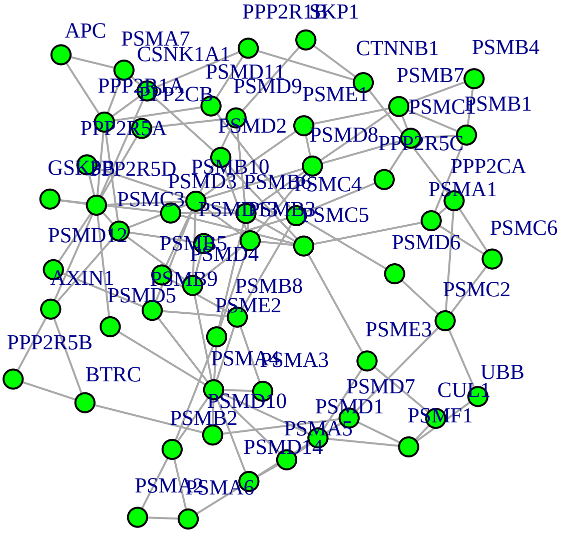

MST2 of the coexpression network for NEG ALL

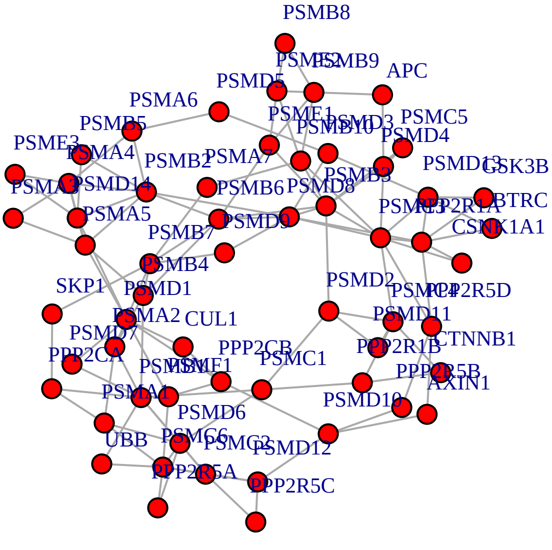

way: REACTOME_ASSOCIATION_OF_TRIC_CCT_WITH_TARGET_PROTEINS_DURING_BIOSYNTH

There are 16 genes in this pathway. This pathway was detected by GSNCA

BCR/ABL ALL
Major Gene (BCR/ABL): CCT3
Weight Factor: 1.348
Major Gene (NEG): CCT5
Weight Factor: 1.327

NEG ALL
Major Gene (NEG): CCT5
Weight Factor: 1.246
Major Gene (BCR/ABL): CCT3
Weight Factor: 1.129

MST2 of the coexpression network for BCR/ABL ALL

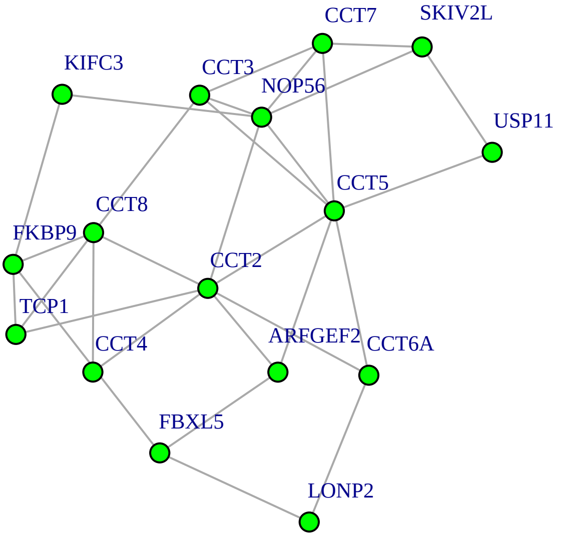

MST2 of the coexpression network for NEG ALL

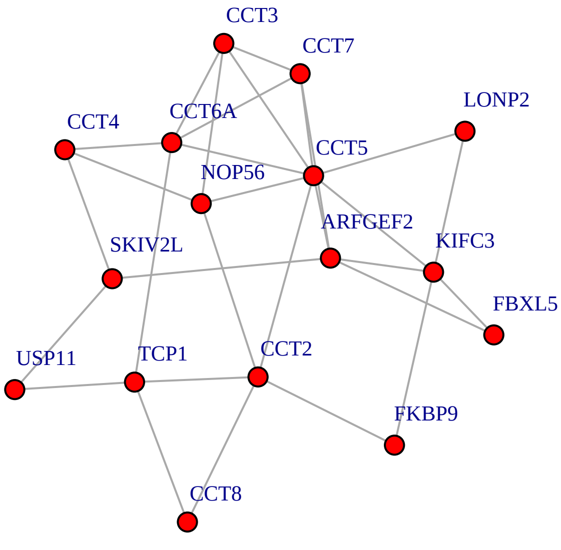

Pathway: NAKAMURA_CANCER_MICROENVIRONMENT_DN

There are 33 genes in this pathway. This pathway was detected by GSCA

BCR/ABL ALL
Major Gene (BCR/ABL): CCNB2
Weight Factor: 1.473
Major Gene (NEG): IARS
Weight Factor: 1.231

NEG ALL
Major Gene (NEG): IARS
Weight Factor: 1.396
Major Gene (BCR/ABL): CCNB2
Weight Factor: 1.351

MST2 of the coexpression network for BCR/ABL ALL

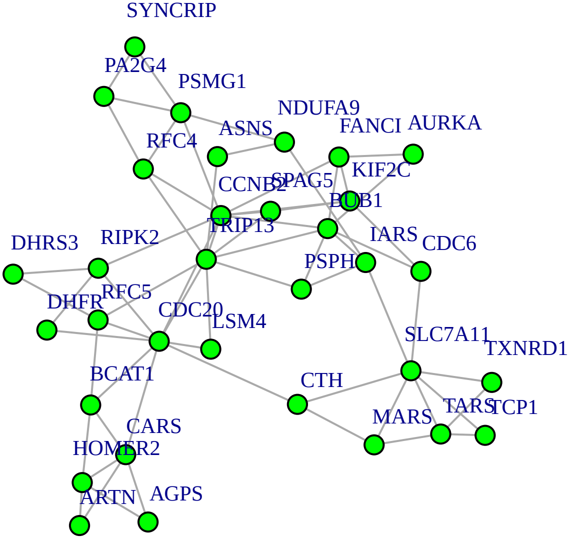

MST2 of the coexpression network for NEG ALL

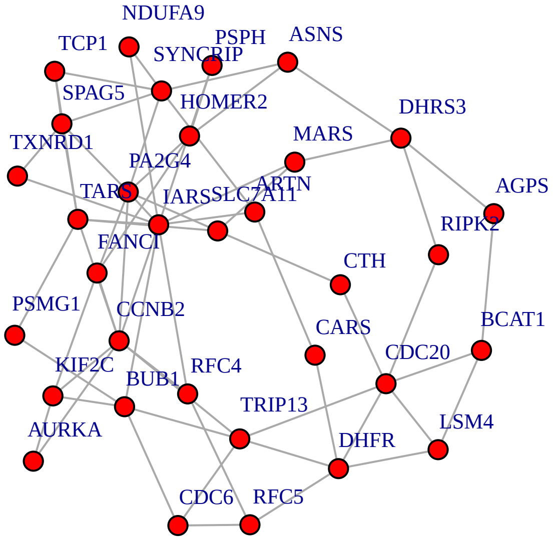

Pathway: KORKOLA_EMBRYONAL_CARCINOMA_UP

There are 24 genes in this pathway. This pathway was detected by GSCA

BCR/ABL ALL
Major Gene (BCR/ABL): ARHGDIB
Weight Factor: 1.398
Major Gene (NEG): EMG1
Weight Factor: 1.034

NEG ALL
Major Gene (NEG): EMG1
Weight Factor: 1.195
Major Gene (BCR/ABL): ARHGDIB
Weight Factor: 1.133

MST2 of the coexpression network for BCR/ABL ALL

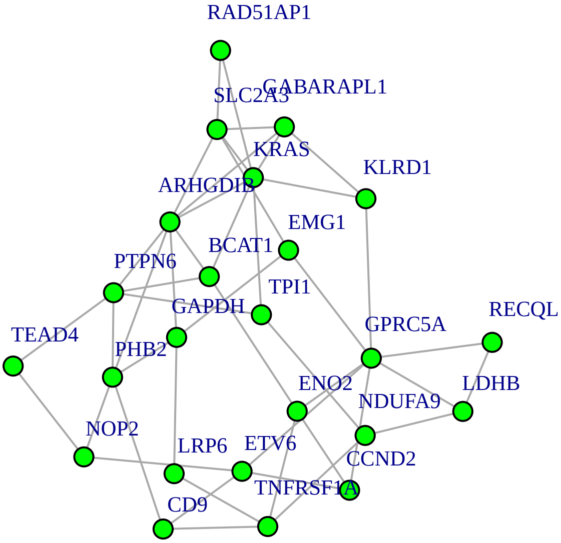

MST2 of the coexpression network for NEG ALL

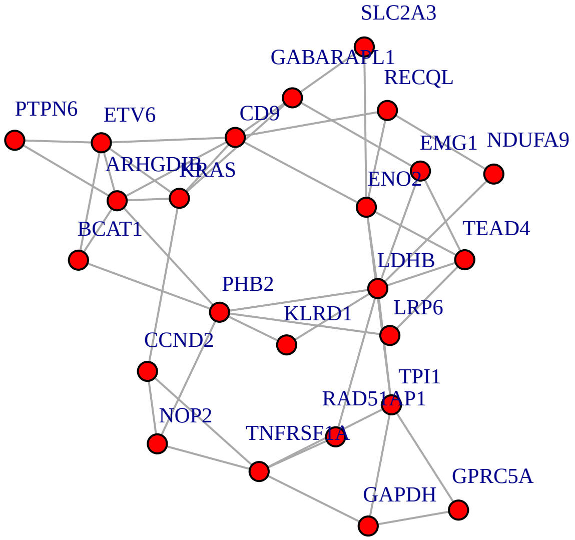

Pathway: KORKOLA_SEMINOMA_UP

There are 28 genes in this pathway. This pathway was detected by GSCA

BCR/ABL ALL
Major Gene (BCR/ABL): ARHGDIB
Weight Factor: 1.294
Major Gene (NEG): ATN1
Weight Factor: 1.193

NEG ALL
Major Gene (NEG): ATN1
Weight Factor: 1.359
Major Gene (BCR/ABL): ARHGDIB
Weight Factor: 1.061

MST2 of the coexpression network for BCR/ABL ALL

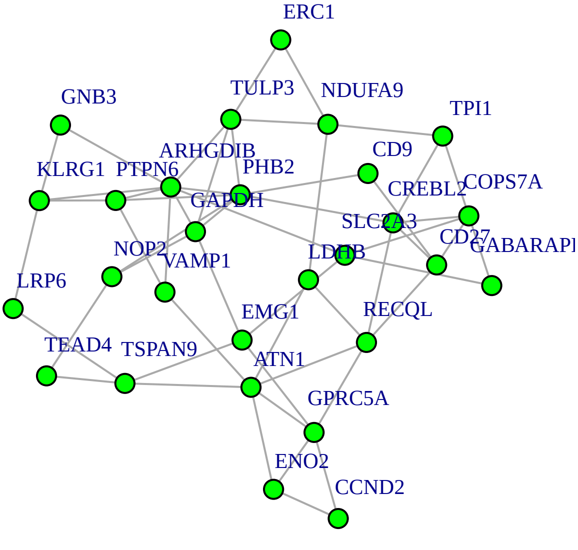

MST2 of the coexpression network for NEG ALL

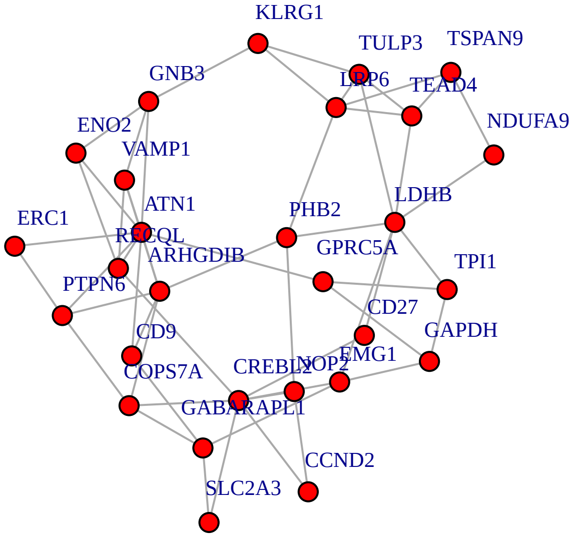

Pathway: HOLLMAN_APOPTOSIS_VIA_CD40_DN

There are 186 genes in this pathway. This pathway was detected by GSCA

BCR/ABL ALL
Major Gene (BCR/ABL): GTF2H5
Weight Factor: 1.423
Major Gene (NEG): CDKN1B
Weight Factor: 1.141

NEG ALL
Major Gene (NEG): CDKN1B
Weight Factor: 1.401
Major Gene (BCR/ABL): GTF2H5
Weight Factor: 1.205

MST2 of the coexpression network for BCR/ABL ALL

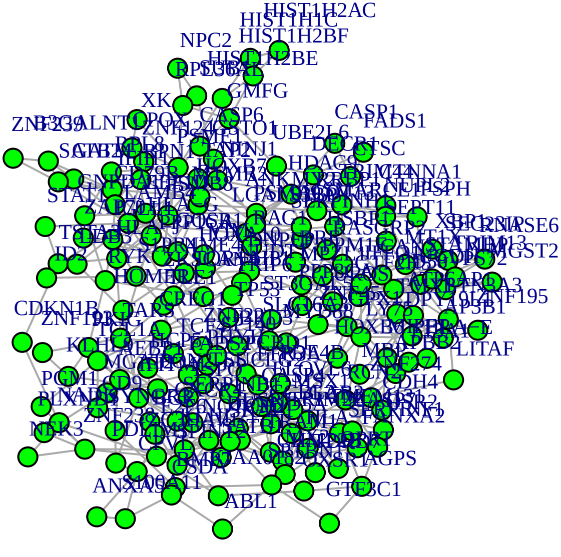

MST2 of the coexpression network for NEG ALL

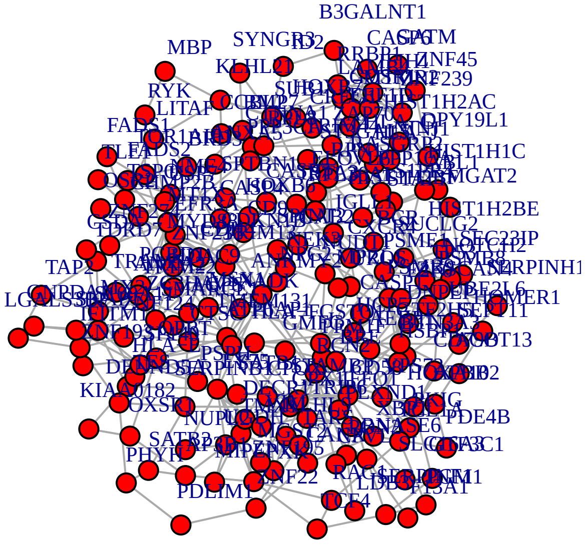

Pathway: TURASHVILI_BREAST_DUCTAL_CARCINOMA_VS_LOBULAR_NORMAL_DN

There are 47 genes in this pathway. This pathway was detected by GSCA

BCR/ABL ALL
Major Gene (BCR/ABL): CX3CL1
Weight Factor: 1.35
Major Gene (NEG): CNN1
Weight Factor: 1.133

NEG ALL
Major Gene (NEG): CNN1
Weight Factor: 1.373
Major Gene (BCR/ABL): CX3CL1
Weight Factor: 1.084

MST2 of the coexpression network for BCR/ABL ALL

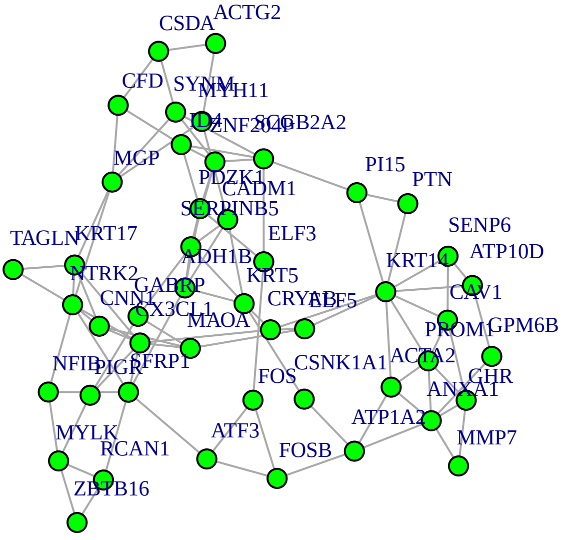

MST2 of the coexpression network for NEG ALL

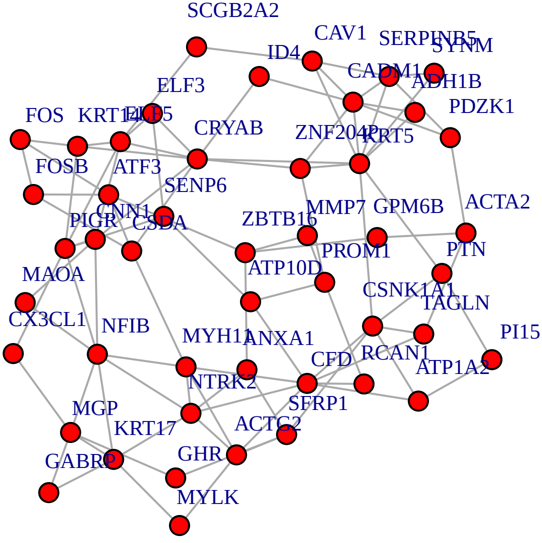

Pathway: DIAZ_CHRONIC_MEYLOGENOUS_LEUKEMIA_DN

There are 93 genes in this pathway. This pathway was detected by GSCA

BCR/ABL ALL
Major Gene (BCR/ABL): RPS12
Weight Factor: 1.362
Major Gene (NEG): RPL32
Weight Factor: 1.093

NEG ALL
Major Gene (NEG): RPL32
Weight Factor: 1.581
Major Gene (BCR/ABL): RPS12
Weight Factor: 1.333

MST2 of the coexpression network for BCR/ABL ALL

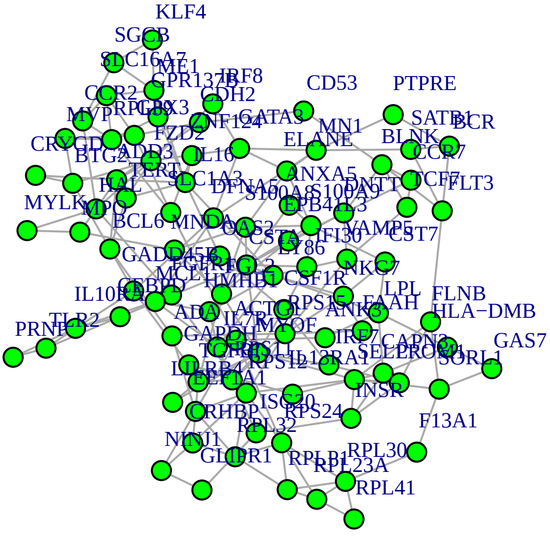

MST2 of the coexpression network for NEG ALL

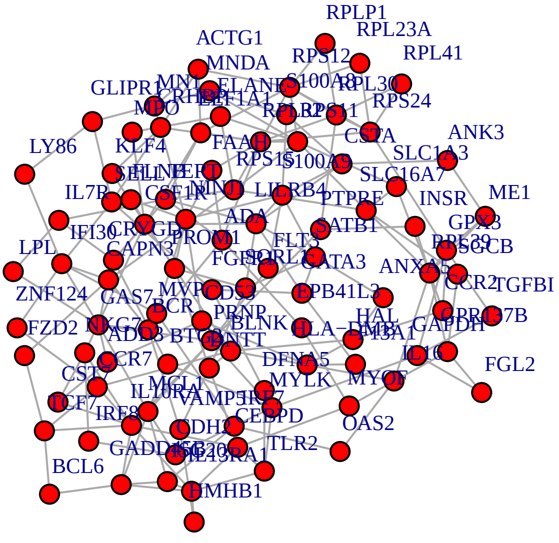

Pathway: CASORELLI_APL_SECONDARY_VS_DE_NOVO_UP

There are 27 genes in this pathway. This pathway was detected by GSCA

BCR/ABL ALL
Major Gene (BCR/ABL): NID1
Weight Factor: 1.275
Major Gene (NEG): TMX4
Weight Factor: 1.059

NEG ALL
Major Gene (NEG): TMX4
Weight Factor: 1.303
Major Gene (BCR/ABL): NID1
Weight Factor: 0.944

MST2 of the coexpression network for BCR/ABL ALL

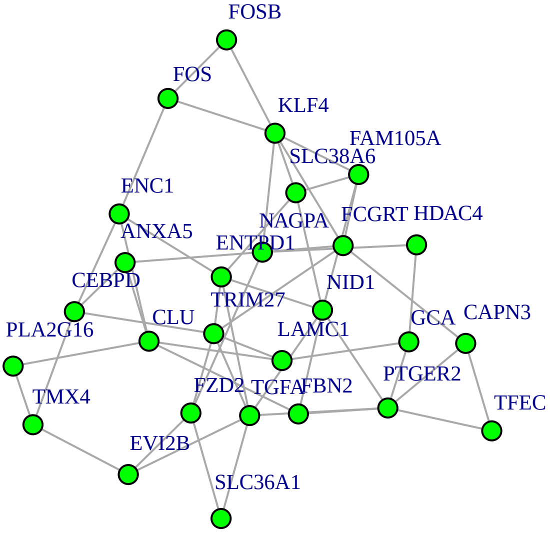

MST2 of the coexpression network for NEG ALL

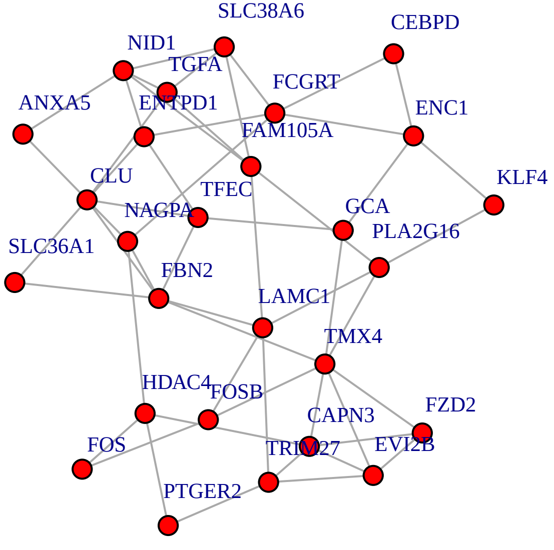

Pathway: GARGALOVIC_RESPONSE_TO_OXIDIZED_PHOSPHOLIPIDS_YELLOW_UP

There are 19 genes in this pathway. This pathway was detected by GSCA

BCR/ABL ALL
Major Gene (BCR/ABL): PHLPP2
Weight Factor: 1.326
Major Gene (NEG): NUP153
Weight Factor: 1.208

NEG ALL
Major Gene (NEG): NUP153
Weight Factor: 1.332
Major Gene (BCR/ABL): PHLPP2
Weight Factor: 0.995

MST2 of the coexpression network for BCR/ABL ALL

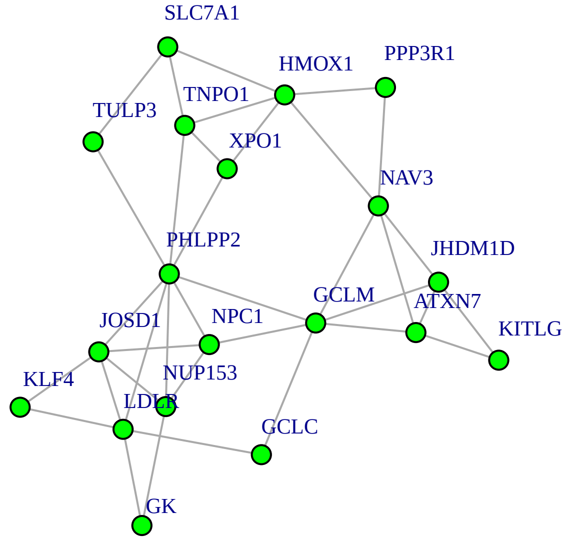

MST2 of the coexpression network for NEG ALL

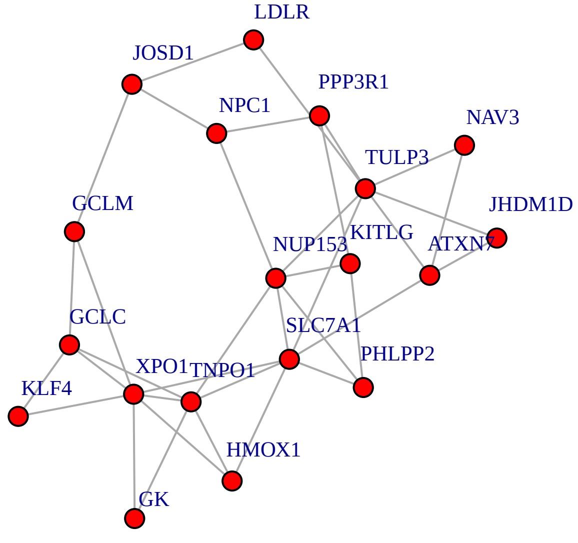

Pathway: TAKEDA_TARGETS_OF_NUP98_HOXA9_FUSION_3D_UP

There are 120 genes in this pathway. This pathway was detected by GSCA

BCR/ABL ALL
Major Gene (BCR/ABL): IFI27
Weight Factor: 1.338
Major Gene (NEG): CELA2A
Weight Factor: 1.273

NEG ALL
Major Gene (NEG): CELA2A
Weight Factor: 1.355
Major Gene (BCR/ABL): IFI27
Weight Factor: 0.958

MST2 of the coexpression network for BCR/ABL ALL

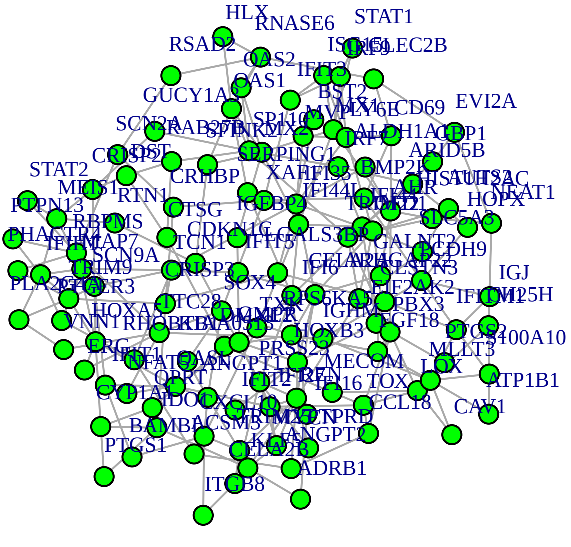

MST2 of the coexpression network for NEG ALL

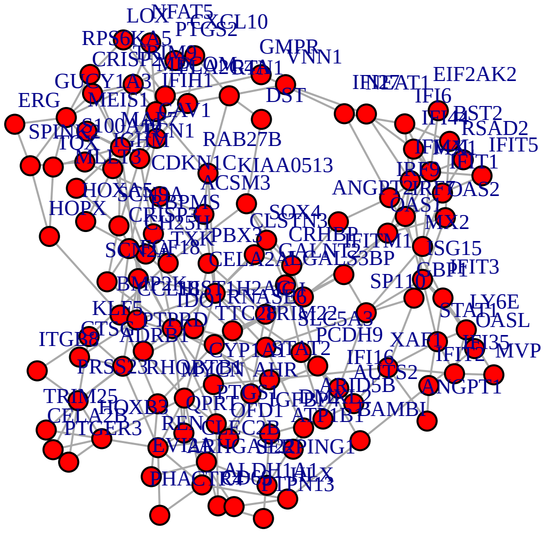

Pathway: TAKEDA_TARGETS_OF_NUP98_HOXA9_FUSION_8D_UP

There are 103 genes in this pathway. This pathway was detected by GSCA

BCR/ABL ALL
Major Gene (BCR/ABL): GAD1
Weight Factor: 1.346
Major Gene (NEG): FGF18
Weight Factor: 0.965

NEG ALL
Major Gene (NEG): FGF18
Weight Factor: 1.348
Major Gene (BCR/ABL): GAD1
Weight Factor: 1.158

MST2 of the coexpression network for BCR/ABL ALL

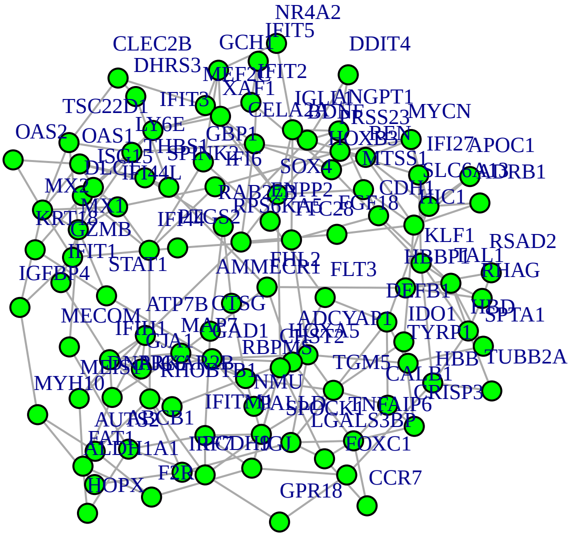

MST2 of the coexpression network for NEG ALL

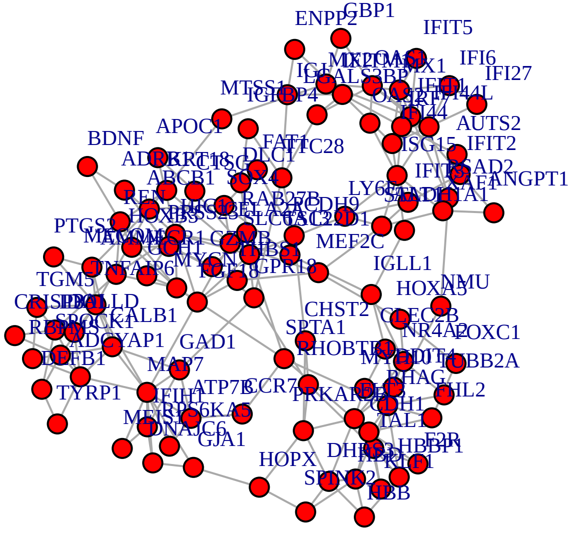

Pathway: TAKEDA_TARGETS_OF_NUP98_HOXA9_FUSION_10D_UP

There are 121 genes in this pathway. This pathway was detected by GSCA

BCR/ABL ALL
Major Gene (BCR/ABL): DEFB1
Weight Factor: 1.343
Major Gene (NEG): OASL
Weight Factor: 1.156

NEG ALL
Major Gene (NEG): OASL
Weight Factor: 1.336
Major Gene (BCR/ABL): DEFB1
Weight Factor: 1.146

MST2 of the coexpression network for BCR/ABL ALL

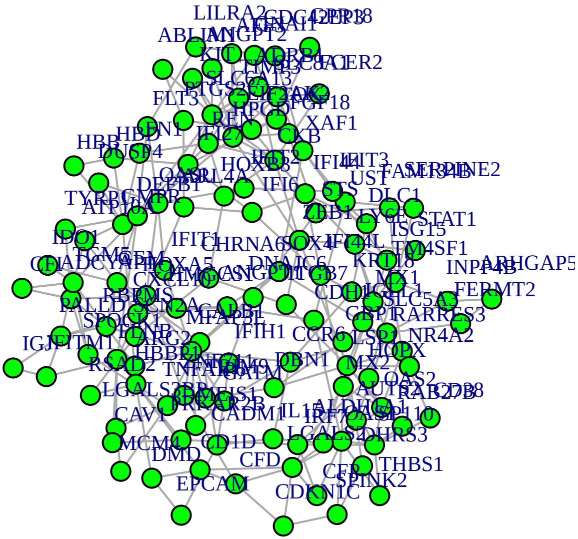

MST2 of the coexpression network for NEG ALL

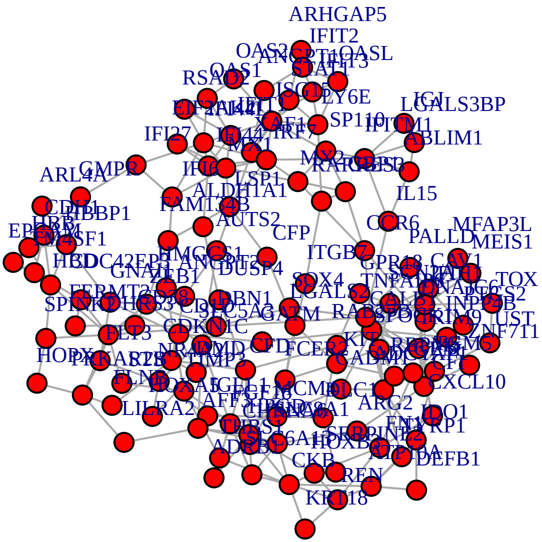

Pathway: TAKEDA_TARGETS_OF_NUP98_HOXA9_FUSION_16D_UP

There are 114 genes in this pathway. This pathway was detected by GSCA

BCR/ABL ALL
Major Gene (BCR/ABL): IFI27
Weight Factor: 1.394
Major Gene (NEG): HPGD
Weight Factor: 1.374

NEG ALL
Major Gene (NEG): HPGD
Weight Factor: 1.453
Major Gene (BCR/ABL): IFI27
Weight Factor: 1.002

MST2 of the coexpression network for BCR/ABL ALL

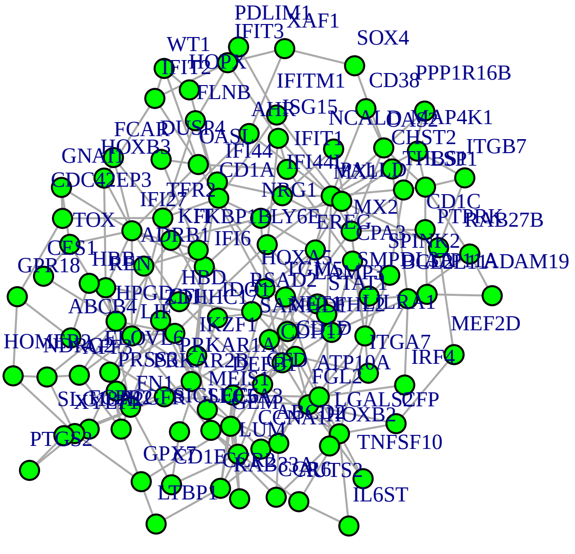

MST2 of the coexpression network for NEG ALL

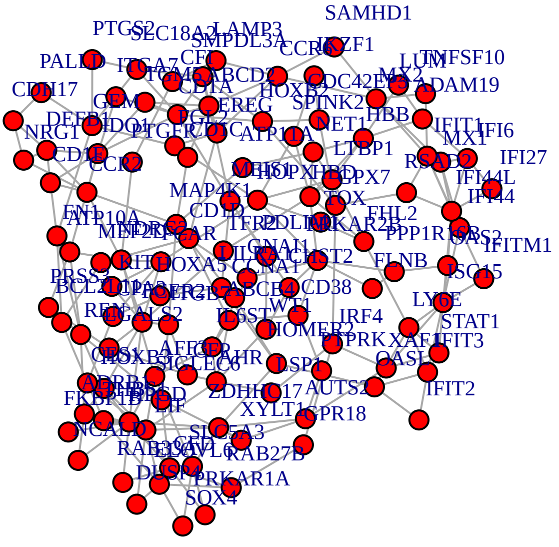

Pathway: BOGNI_TREATMENT_RELATED_MYELOID_LEUKEMIA_DN

There are 28 genes in this pathway. This pathway was detected by GSCA

BCR/ABL ALL
Major Gene (BCR/ABL): MRPL28
Weight Factor: 1.275
Major Gene (NEG): NKTR
Weight Factor: 1.062

NEG ALL
Major Gene (NEG): NKTR
Weight Factor: 1.461
Major Gene (BCR/ABL): MRPL28
Weight Factor: 1.316

MST2 of the coexpression network for BCR/ABL ALL

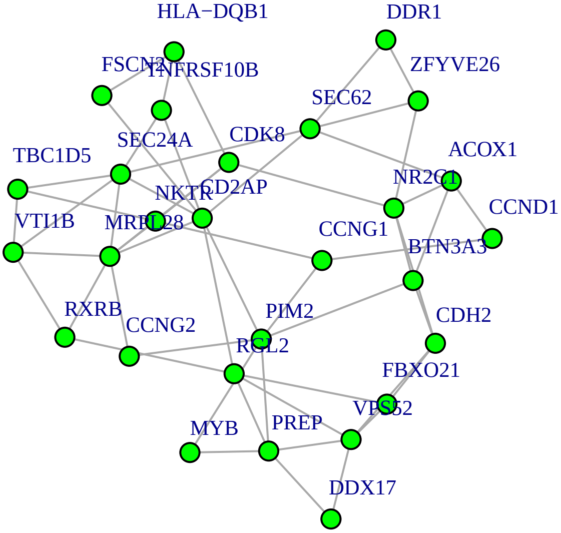

MST2 of the coexpression network for NEG ALL

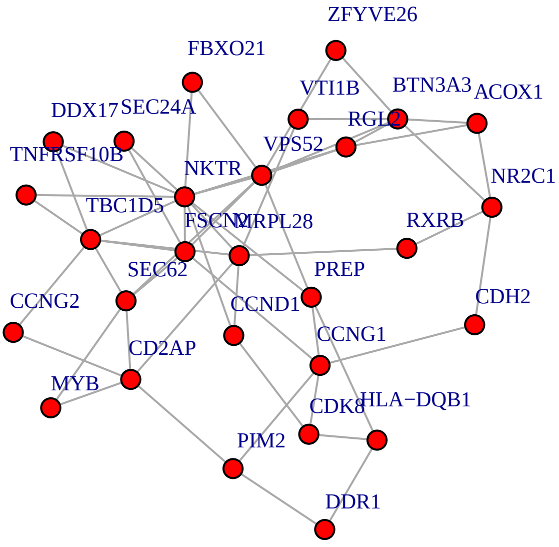

Pathway: HOEBEKE_LYMPHOID_STEM_CELL_DN

There are 69 genes in this pathway. This pathway was detected by GSCA

BCR/ABL ALL
Major Gene (BCR/ABL): SLC39A8
Weight Factor: 1.286
Major Gene (NEG): CAT
Weight Factor: 1.108

NEG ALL
Major Gene (NEG): CAT
Weight Factor: 1.414
Major Gene (BCR/ABL): SLC39A8
Weight Factor: 0.86

MST2 of the coexpression network for BCR/ABL ALL

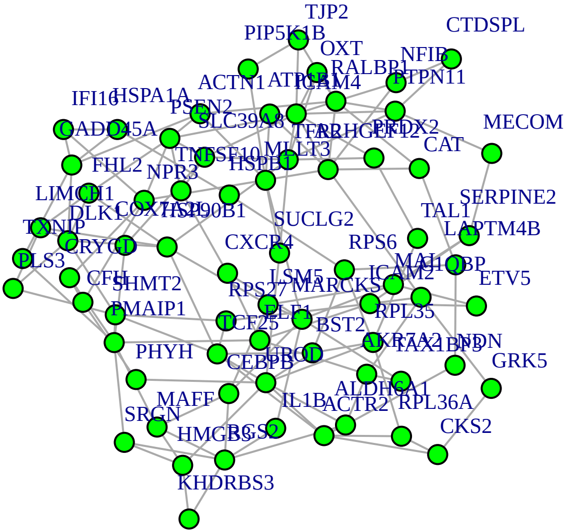

MST2 of the coexpression network for NEG ALL

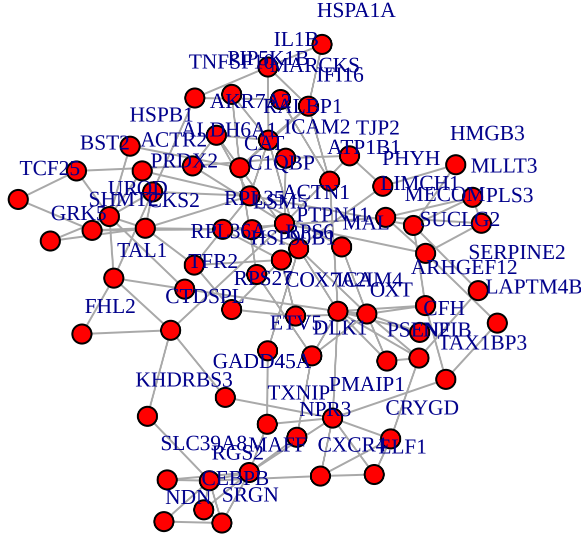

Pathway: AKL_HTLV1_INFECTION_UP

There are 23 genes in this pathway. This pathway was detected by GSCA

BCR/ABL ALL
Major Gene (BCR/ABL): NME1
Weight Factor: 1.523
Major Gene (NEG): NME1
Weight Factor: 1.523

NEG ALL
Major Gene (NEG): NME1
Weight Factor: 1.457
Major Gene (BCR/ABL): NME1
Weight Factor: 1.457

MST2 of the coexpression network for BCR/ABL ALL

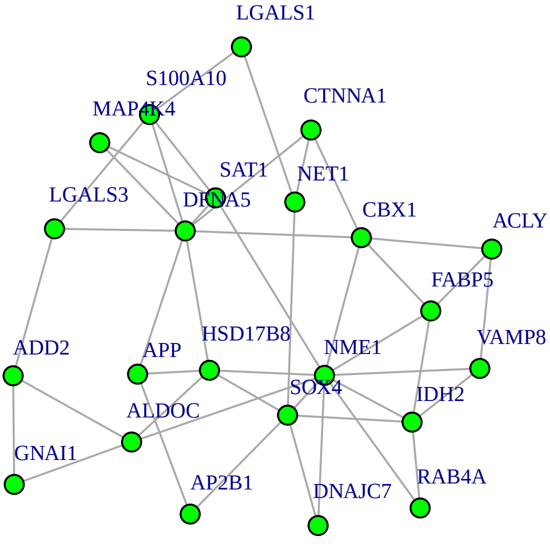

MST2 of the coexpression network for NEG ALL

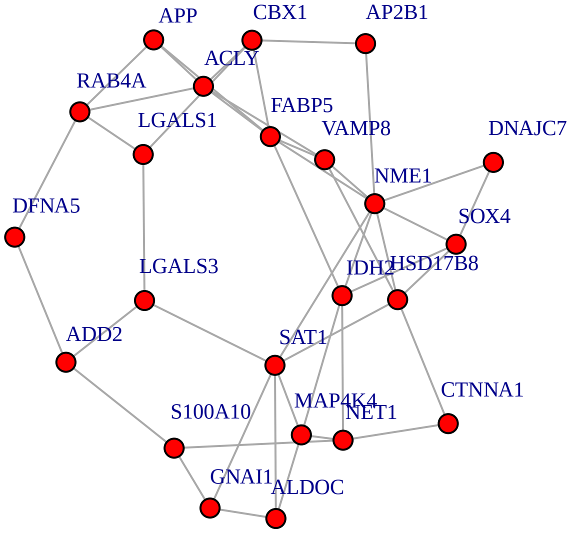

Pathway: RHEIN_ALL_GLUCOCORTICOID_THERAPY_UP

There are 65 genes in this pathway. This pathway was detected by GSCA

BCR/ABL ALL
Major Gene (BCR/ABL): IL8
Weight Factor: 1.468
Major Gene (NEG): CD55
Weight Factor: 1.363

NEG ALL
Major Gene (NEG): CD55
Weight Factor: 1.481
Major Gene (BCR/ABL): IL8
Weight Factor: 1.223

MST2 of the coexpression network for BCR/ABL ALL

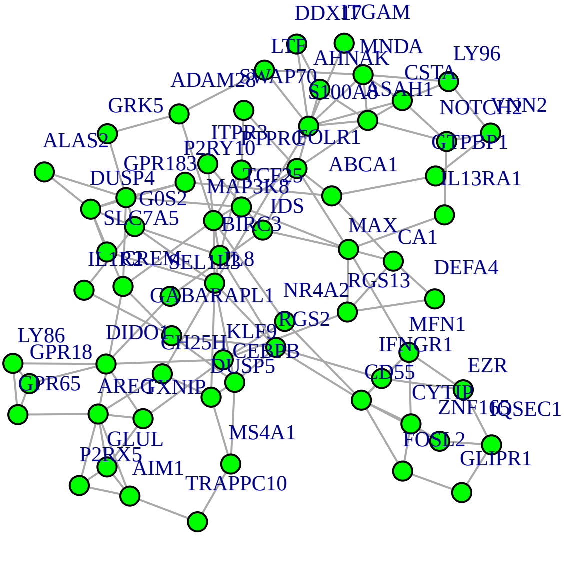

MST2 of the coexpression network for NEG ALL

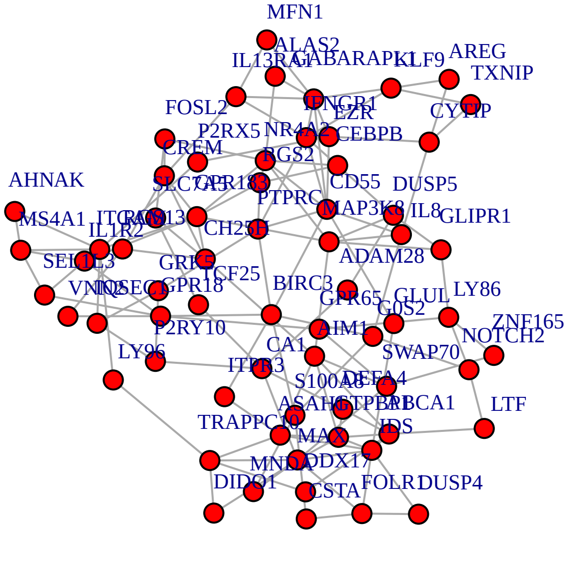

Pathway: MULLIGHAN_MLL_SIGNATURE_1_DN

There are 184 genes in this pathway. This pathway was detected by GSCA

BCR/ABL ALL
Major Gene (BCR/ABL): HYAL2
Weight Factor: 1.548
Major Gene (NEG): NOVA2
Weight Factor: 1.44

NEG ALL
Major Gene (NEG): NOVA2
Weight Factor: 1.596
Major Gene (BCR/ABL): HYAL2
Weight Factor: 1.446

MST2 of the coexpression network for BCR/ABL ALL

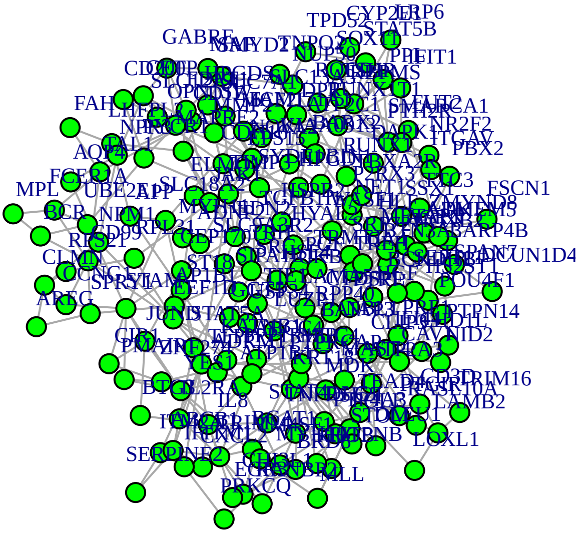

MST2 of the coexpression network for NEG ALL

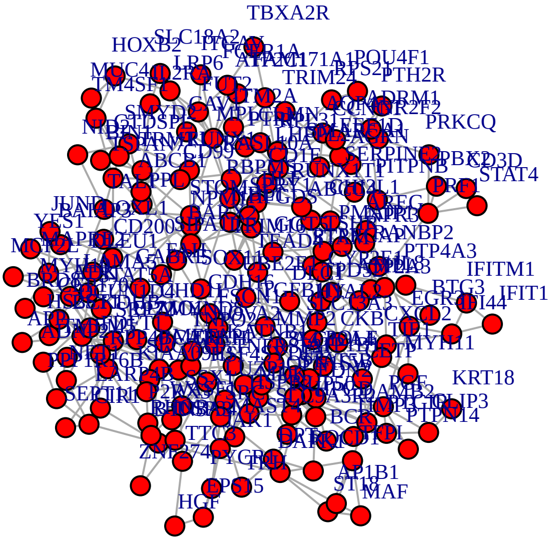

Pathway: MULLIGHAN_MLL_SIGNATURE_2_DN

There are 221 genes in this pathway. This pathway was detected by GSCA

BCR/ABL ALL
Major Gene (BCR/ABL): EDN2
Weight Factor: 1.505
Major Gene (NEG): ASS1
Weight Factor: 1.264

NEG ALL
Major Gene (NEG): ASS1
Weight Factor: 1.579
Major Gene (BCR/ABL): EDN2
Weight Factor: 1.534

MST2 of the coexpression network for BCR/ABL ALL

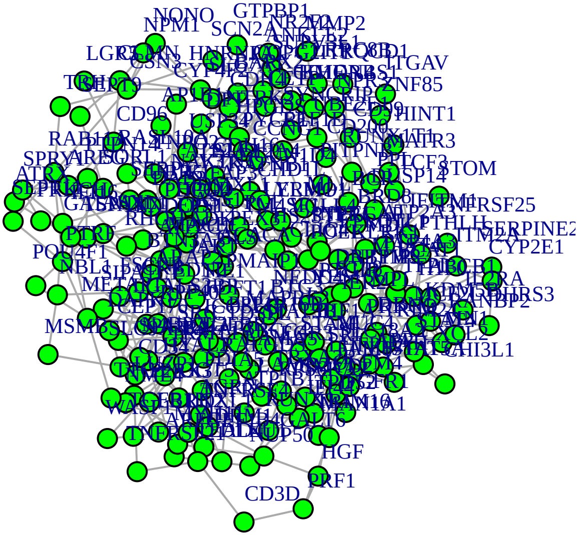

MST2 of the coexpression network for NEG ALL

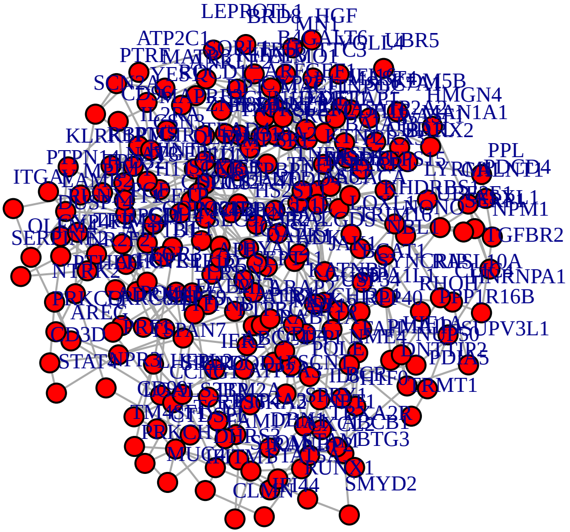

Pathway: TIEN_INTESTINE_PROBIOTICS_6HR_DN

There are 121 genes in this pathway. This pathway was detected by GSCA

BCR/ABL ALL
Major Gene (BCR/ABL): N4BP1
Weight Factor: 1.529
Major Gene (NEG): PICALM
Weight Factor: 1.272

NEG ALL
Major Gene (NEG): PICALM
Weight Factor: 1.57
Major Gene (BCR/ABL): N4BP1
Weight Factor: 1.155

MST2 of the coexpression network for BCR/ABL ALL

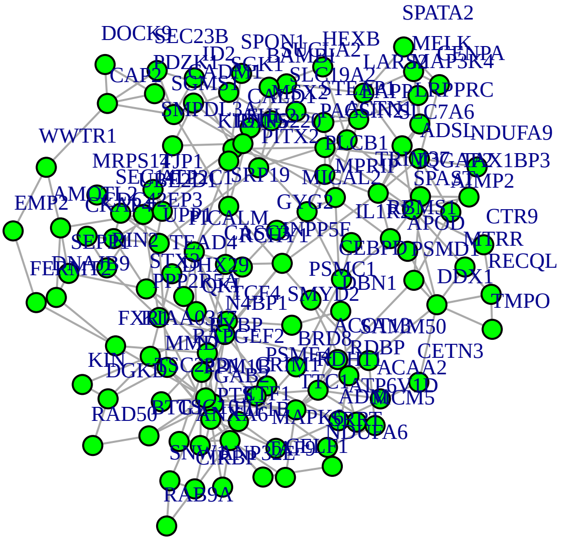

MST2 of the coexpression network for NEG ALL

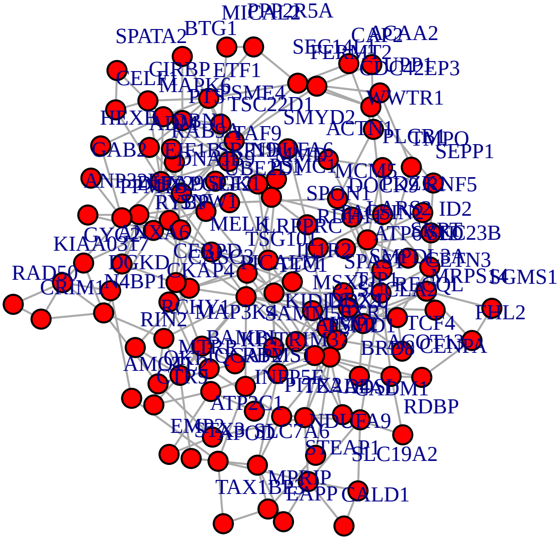

Pathway: ELVIDGE_HIF1A_TARGETS_UP

There are 47 genes in this pathway. This pathway was detected by GSCA

BCR/ABL ALL
Major Gene (BCR/ABL): MRPS12
Weight Factor: 1.372
Major Gene (NEG): CHUK
Weight Factor: 1.182

NEG ALL
Major Gene (NEG): CHUK
Weight Factor: 1.324
Major Gene (BCR/ABL): MRPS12
Weight Factor: 1.047

MST2 of the coexpression network for BCR/ABL ALL

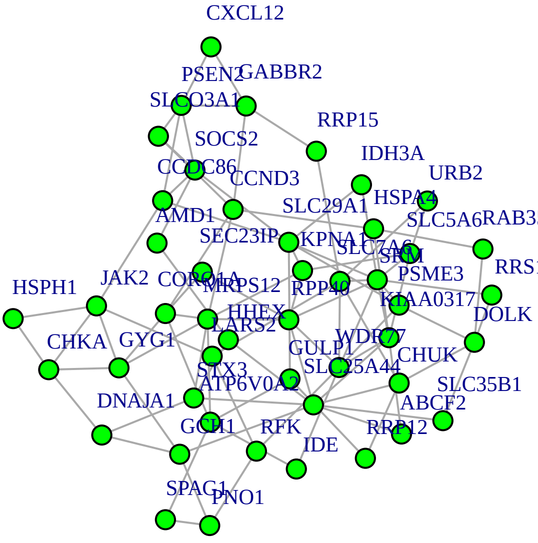

MST2 of the coexpression network for NEG ALL

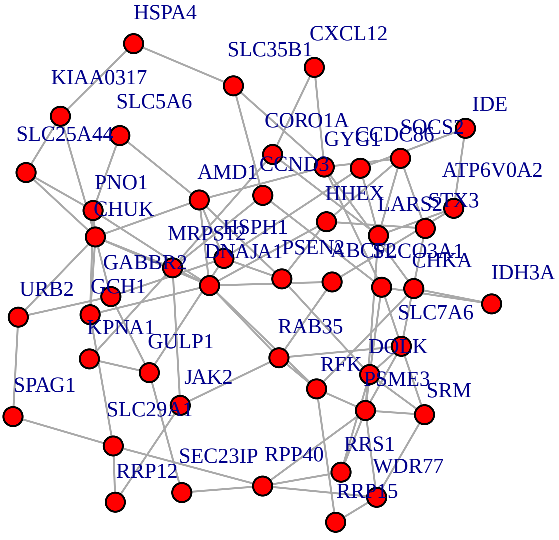

Pathway: ELVIDGE_HIF1A_AND_HIF2A_TARGETS_UP

There are 29 genes in this pathway. This pathway was detected by GSCA

BCR/ABL ALL
Major Gene (BCR/ABL): FLAD1
Weight Factor: 1.425
Major Gene (NEG): CHUK
Weight Factor: 0.968

NEG ALL
Major Gene (NEG): CHUK
Weight Factor: 1.262
Major Gene (BCR/ABL): FLAD1
Weight Factor: 1.109

MST2 of the coexpression network for BCR/ABL ALL

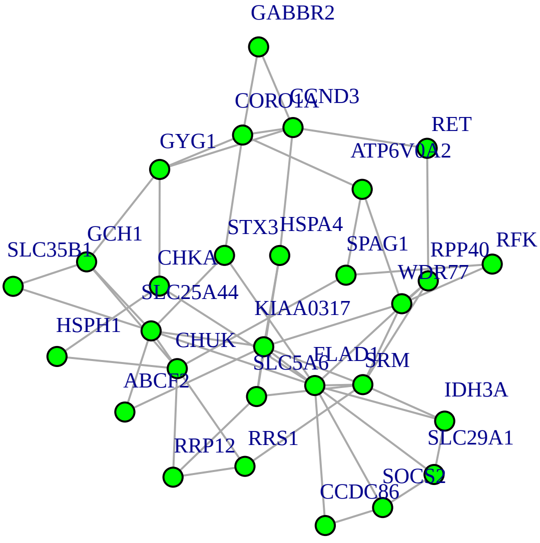

MST2 of the coexpression network for NEG ALL

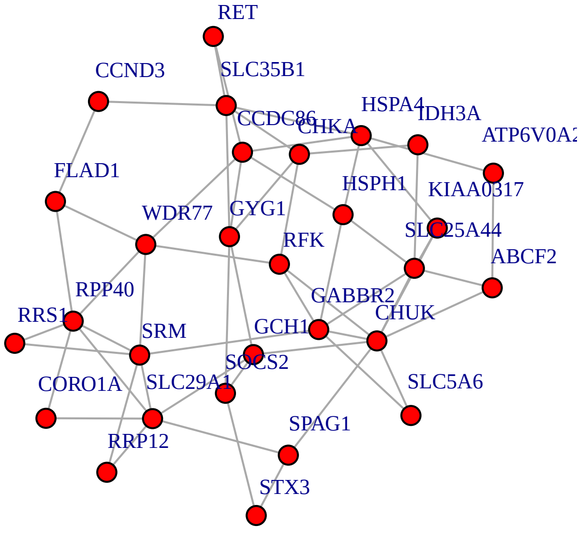

Pathway: JAATINEN_HEMATOPOIETIC_STEM_CELL_DN

There are 168 genes in this pathway. This pathway was detected by GSCA

BCR/ABL ALL
Major Gene (BCR/ABL): FCN1
Weight Factor: 1.534
Major Gene (NEG): FGR
Weight Factor: 1.452

NEG ALL
Major Gene (NEG): FGR
Weight Factor: 1.468
Major Gene (BCR/ABL): FCN1
Weight Factor: 1.183

MST2 of the coexpression network for BCR/ABL ALL

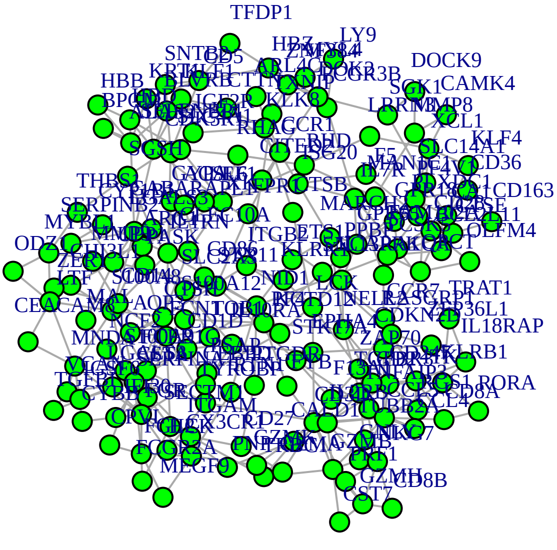

MST2 of the coexpression network for NEG ALL

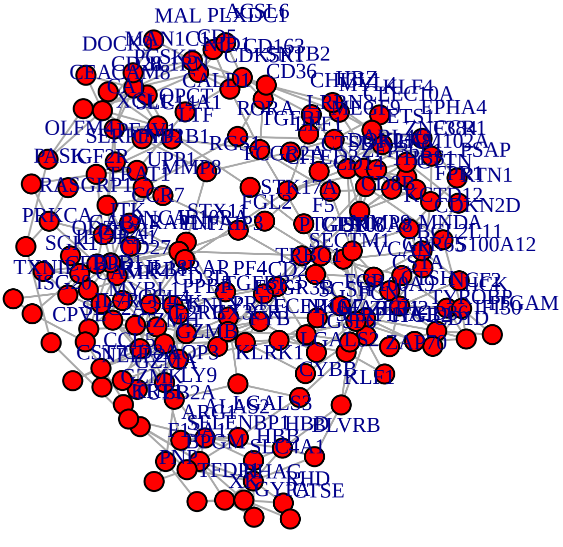

Pathway: RODRIGUES_THYROID_CARCINOMA_DN

There are 53 genes in this pathway. This pathway was detected by GSCA

BCR/ABL ALL
Major Gene (BCR/ABL): ZMYM2
Weight Factor: 1.475
Major Gene (NEG): LIF
Weight Factor: 1.367

NEG ALL
Major Gene (NEG): LIF
Weight Factor: 1.559
Major Gene (BCR/ABL): ZMYM2
Weight Factor: 1.267

MST2 of the coexpression network for BCR/ABL ALL

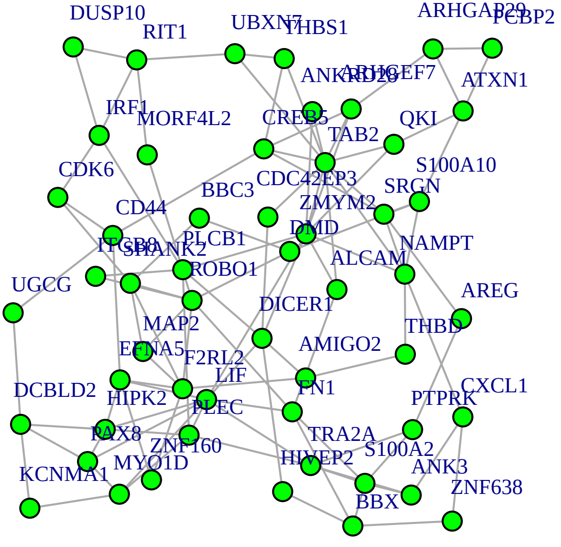

MST2 of the coexpression network for NEG ALL

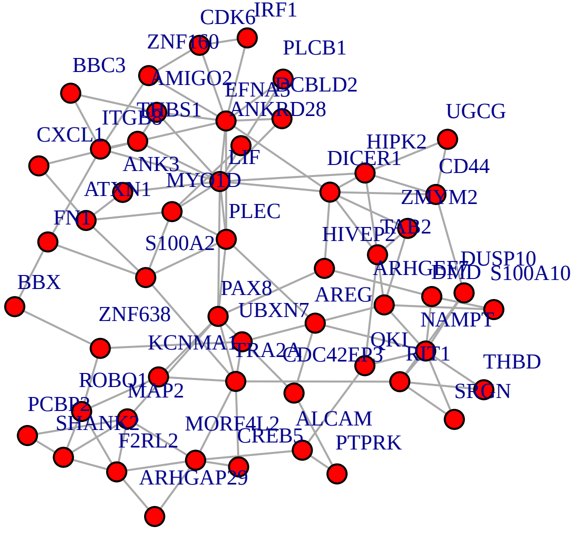

Pathway: HAHTOLA_MYCOSIS_FUNGOIDES_SKIN_UP

There are 131 genes in this pathway. This pathway was detected by GSCA

BCR/ABL ALL
Major Gene (BCR/ABL): WBSCR22
Weight Factor: 1.452
Major Gene (NEG): UPF2
Weight Factor: 1.438

NEG ALL
Major Gene (NEG): UPF2
Weight Factor: 1.624
Major Gene (BCR/ABL): WBSCR22
Weight Factor: 1.52

MST2 of the coexpression network for BCR/ABL ALL

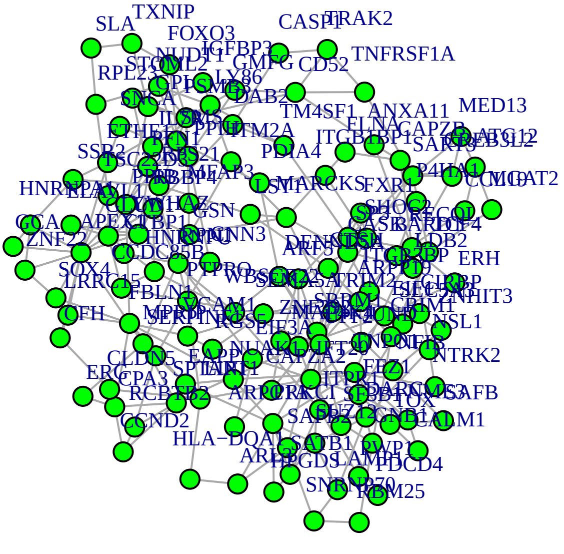

MST2 of the coexpression network for NEG ALL

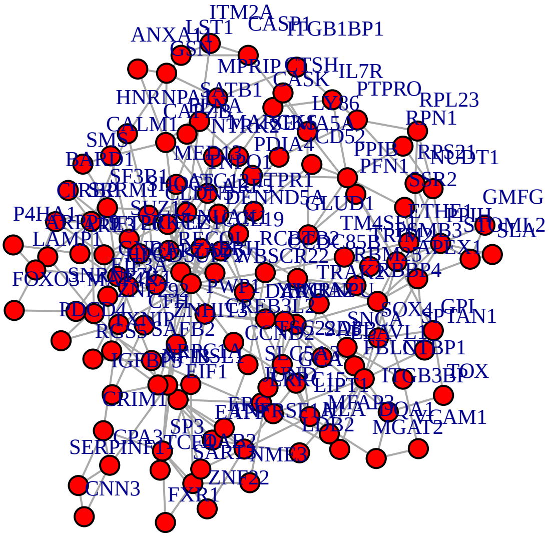

Pathway: GAUSSMANN_MLL_AF4_FUSION_TARGETS_G_DN

There are 20 genes in this pathway. This pathway was detected by GSCA

BCR/ABL ALL
Major Gene (BCR/ABL): WNT4
Weight Factor: 1.352
Major Gene (NEG): ELMO1
Weight Factor: 0.926

NEG ALL
Major Gene (NEG): ELMO1
Weight Factor: 1.307
Major Gene (BCR/ABL): WNT4
Weight Factor: 1.194

MST2 of the coexpression network for BCR/ABL ALL

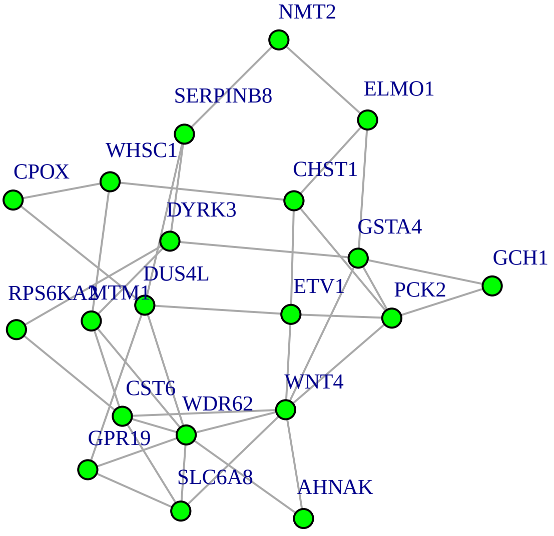

MST2 of the coexpression network for NEG ALL

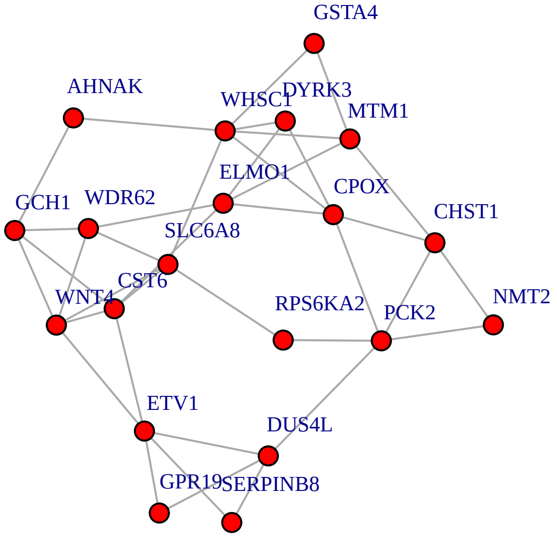

Pathway: OUELLET_OVARIAN_CANCER_INVASIVE_VS_LMP_UP

There are 112 genes in this pathway. This pathway was detected by GSCA

BCR/ABL ALL
Major Gene (BCR/ABL): SNRPD3
Weight Factor: 1.353
Major Gene (NEG): H2AFZ
Weight Factor: 1.253

NEG ALL
Major Gene (NEG): H2AFZ
Weight Factor: 1.366
Major Gene (BCR/ABL): SNRPD3
Weight Factor: 0.981

MST2 of the coexpression network for BCR/ABL ALL

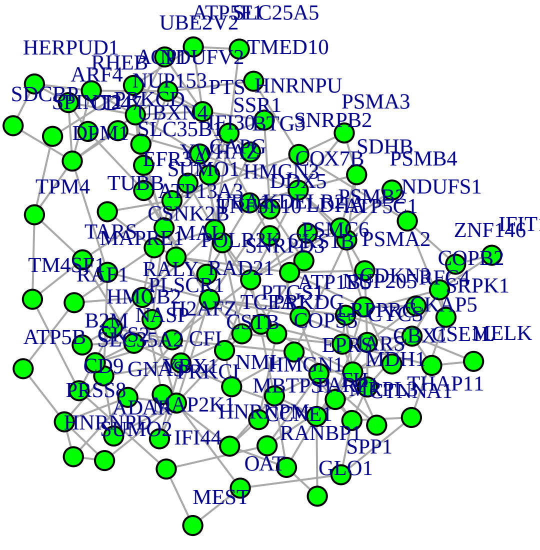

MST2 of the coexpression network for NEG ALL

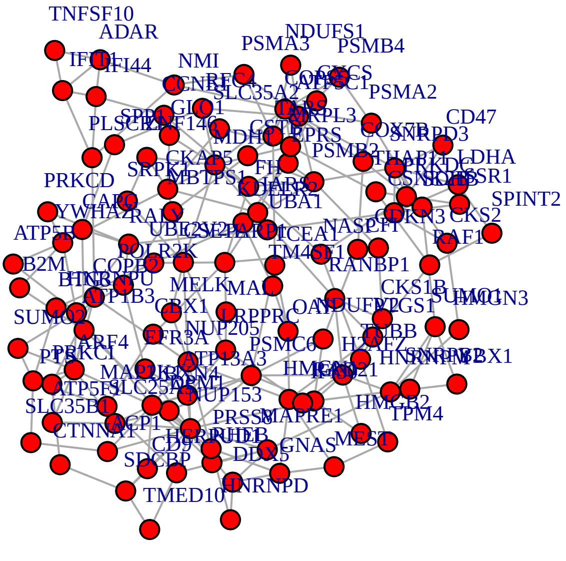

Pathway: MISSIAGLIA_REGULATED_BY_METHYLATION_UP

There are 91 genes in this pathway. This pathway was detected by GSCA

BCR/ABL ALL
Major Gene (BCR/ABL): IGF2
Weight Factor: 1.395
Major Gene (NEG): PRSS8
Weight Factor: 1.25

NEG ALL
Major Gene (NEG): PRSS8
Weight Factor: 1.514
Major Gene (BCR/ABL): IGF2
Weight Factor: 1.483

MST2 of the coexpression network for BCR/ABL ALL

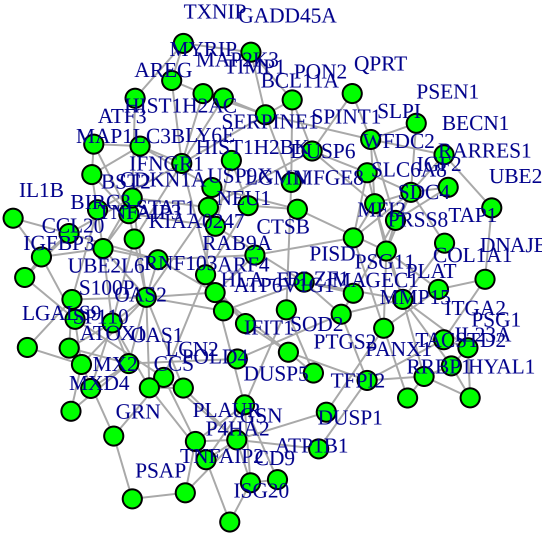

MST2 of the coexpression network for NEG ALL

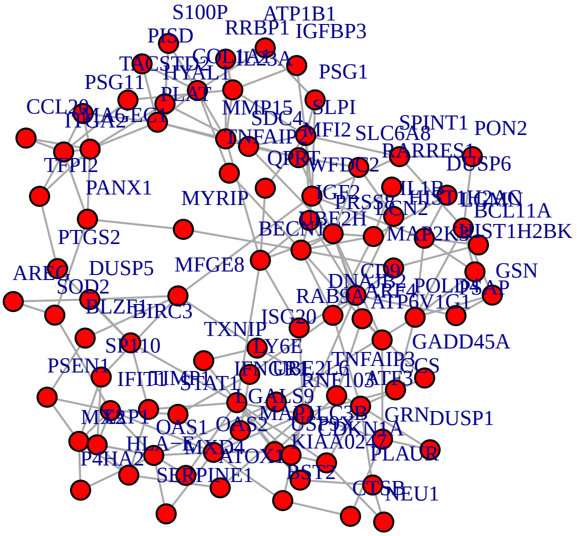

Pathway: DUNNE_TARGETS_OF_AML1_MTG8_FUSION_UP

There are 37 genes in this pathway. This pathway was detected by GSCA

BCR/ABL ALL
Major Gene (BCR/ABL): S100A8
Weight Factor: 1.398
Major Gene (NEG): IL6R
Weight Factor: 0.956

NEG ALL
Major Gene (NEG): IL6R
Weight Factor: 1.406
Major Gene (BCR/ABL): S100A8
Weight Factor: 0.675

MST2 of the coexpression network for BCR/ABL ALL

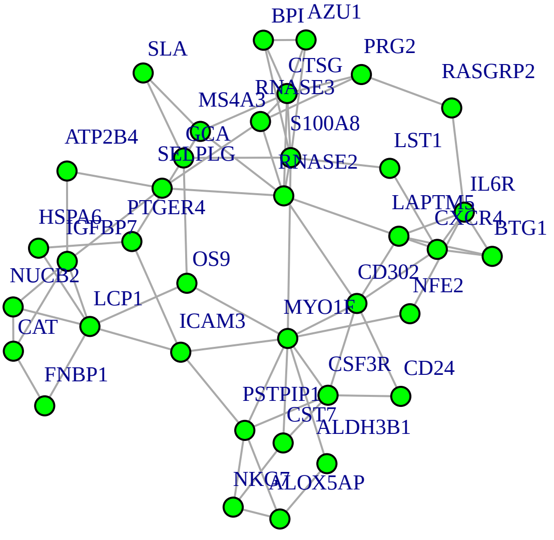

MST2 of the coexpression network for NEG ALL

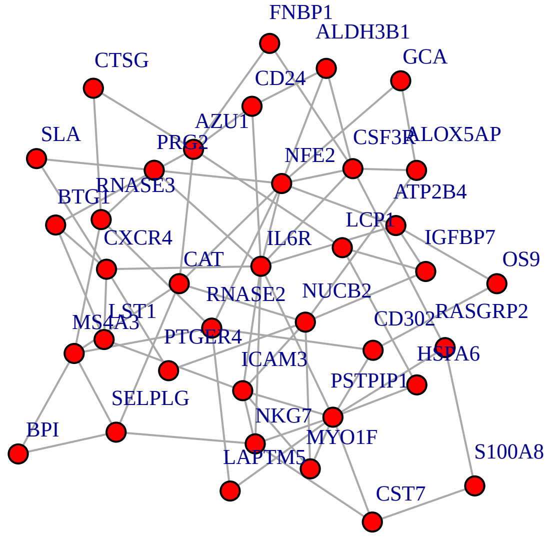

Pathway: TOMIDA_METASTASIS_UP

There are 17 genes in this pathway. This pathway was detected by GSCA

BCR/ABL ALL
Major Gene (BCR/ABL): DAD1
Weight Factor: 1.375
Major Gene (NEG): TUBA3D
Weight Factor: 1.255

NEG ALL
Major Gene (NEG): TUBA3D
Weight Factor: 1.327
Major Gene (BCR/ABL): DAD1
Weight Factor: 1.242

MST2 of the coexpression network for BCR/ABL ALL

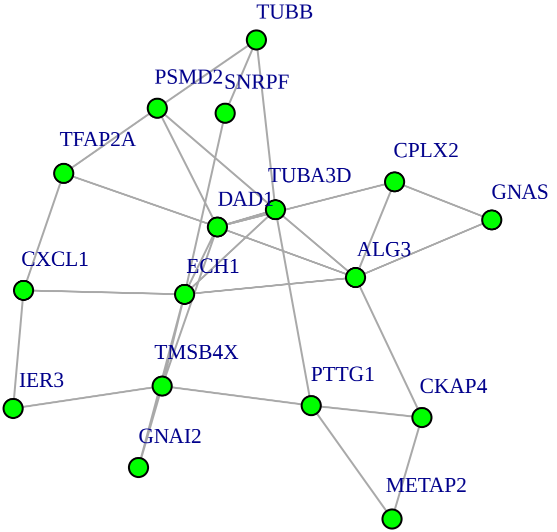

MST2 of the coexpression network for NEG ALL

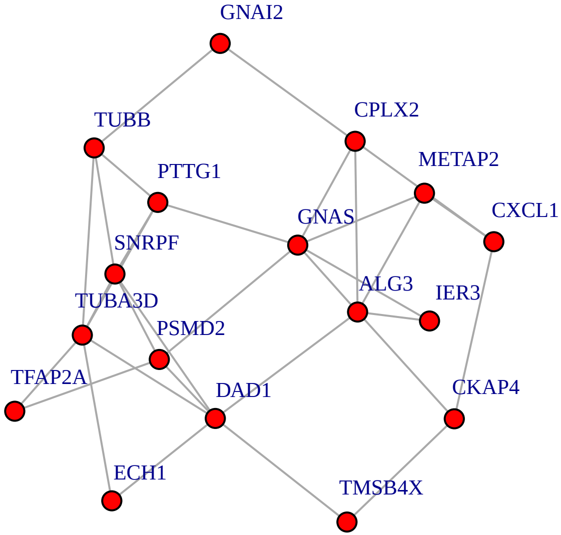

Pathway: HU_ANGIOGENESIS_DN

There are 30 genes in this pathway. This pathway was detected by GSCA

BCR/ABL ALL
Major Gene (BCR/ABL): SOD1
Weight Factor: 1.408
Major Gene (NEG): MDH1
Weight Factor: 1.201

NEG ALL
Major Gene (NEG): MDH1
Weight Factor: 1.328
Major Gene (BCR/ABL): SOD1
Weight Factor: 1.234

MST2 of the coexpression network for BCR/ABL ALL

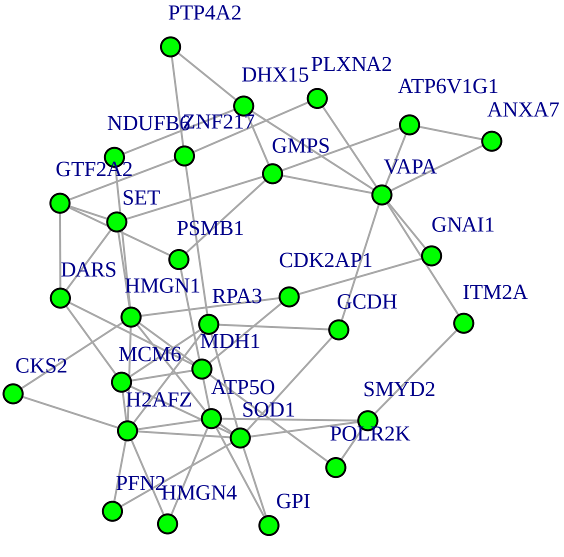

MST2 of the coexpression network for NEG ALL

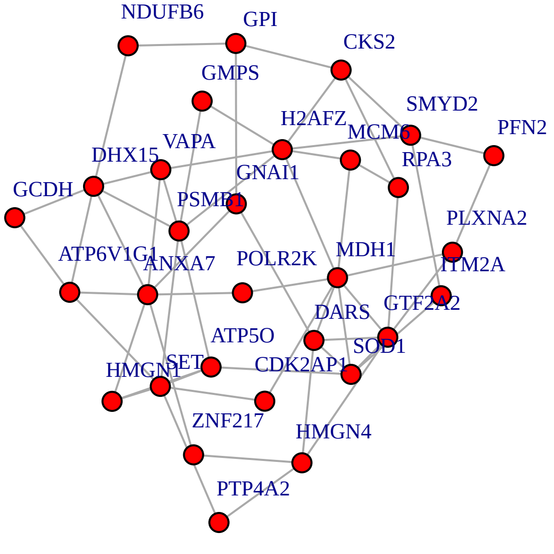

Pathway: APPIERTO_RESPONSE_TO_FENRETINIDE_DN

There are 32 genes in this pathway. This pathway was detected by GSCA

BCR/ABL ALL
Major Gene (BCR/ABL): PGK1
Weight Factor: 1.403
Major Gene (NEG): MDH1
Weight Factor: 1.046

NEG ALL
Major Gene (NEG): MDH1
Weight Factor: 1.452
Major Gene (BCR/ABL): PGK1
Weight Factor: 1.311

MST2 of the coexpression network for BCR/ABL ALL

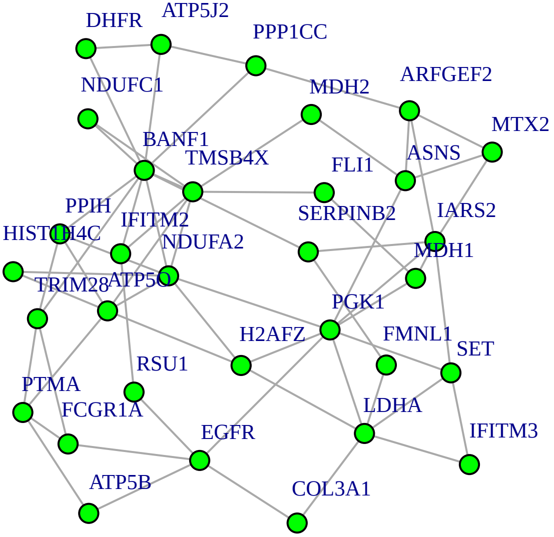

MST2 of the coexpression network for NEG ALL

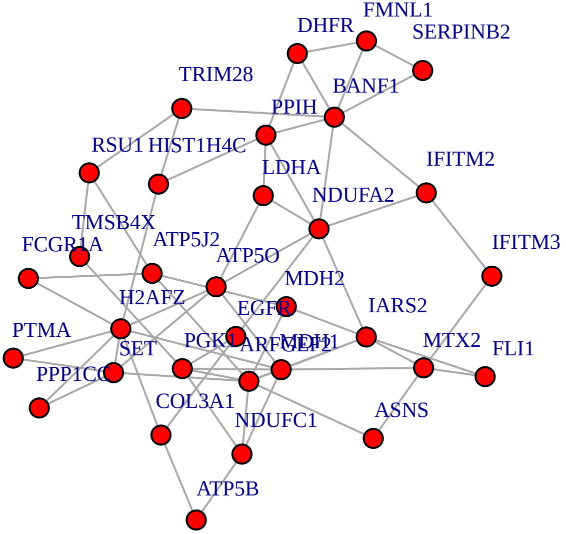

Pathway: LINDGREN_BLADDER_CANCER_CLUSTER_2B

There are 295 genes in this pathway. This pathway was detected by GSCA

BCR/ABL ALL
Major Gene (BCR/ABL): JAK3
Weight Factor: 1.426
Major Gene (NEG): TIMP2
Weight Factor: 1.318

NEG ALL
Major Gene (NEG): TIMP2
Weight Factor: 1.614
Major Gene (BCR/ABL): JAK3
Weight Factor: 1.335

MST2 of the coexpression network for BCR/ABL ALL

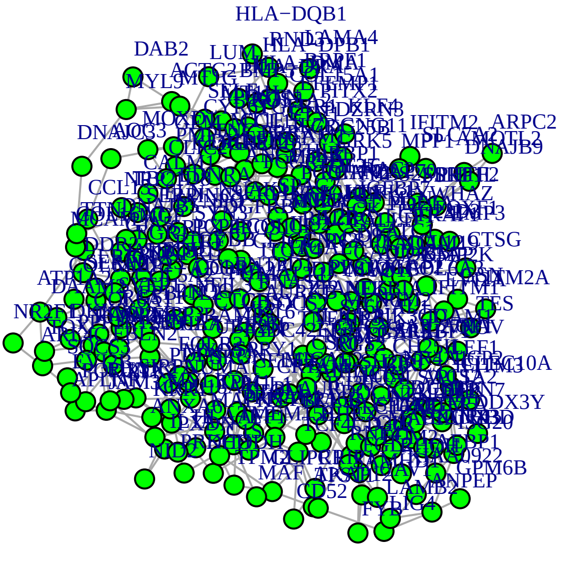

MST2 of the coexpression network for NEG ALL

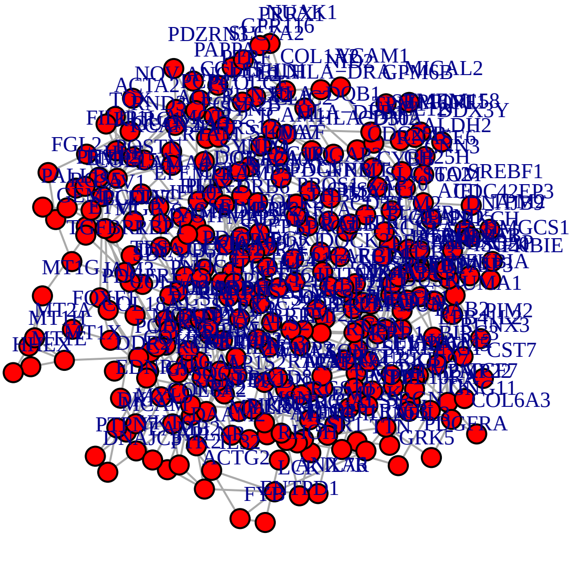

Pathway: EBAUER_TARGETS_OF_PAX3_FOXO1_FUSION_DN

There are 36 genes in this pathway. This pathway was detected by GSCA

BCR/ABL ALL
Major Gene (BCR/ABL): ADRA2A
Weight Factor: 1.269
Major Gene (NEG): ADRA2A
Weight Factor: 1.269

NEG ALL
Major Gene (NEG): ADRA2A
Weight Factor: 1.497
Major Gene (BCR/ABL): ADRA2A
Weight Factor: 1.497

MST2 of the coexpression network for BCR/ABL ALL

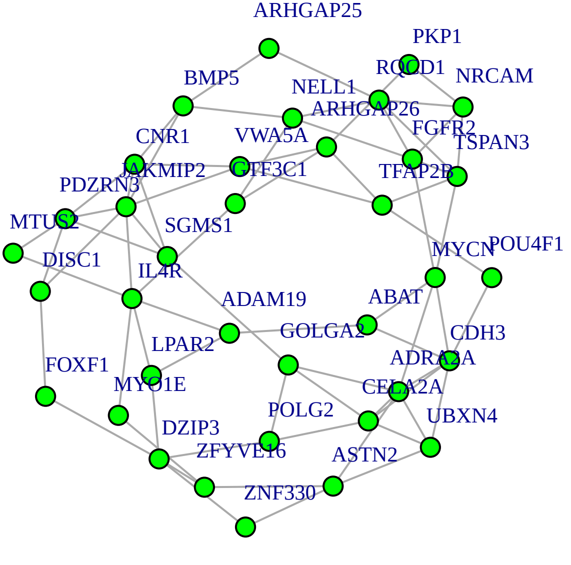

MST2 of the coexpression network for NEG ALL

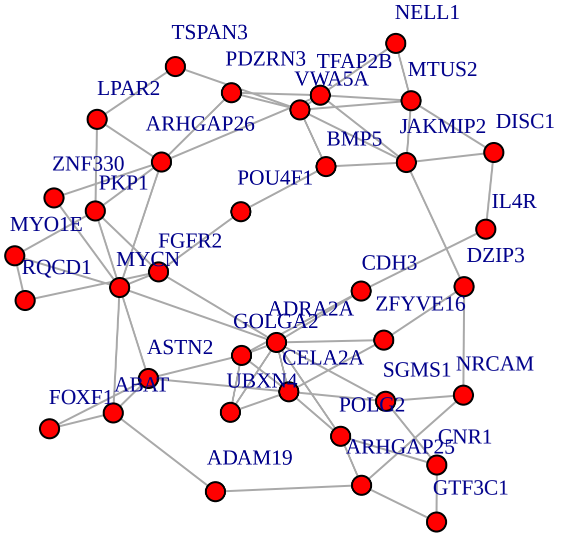

Pathway: MATTIOLI_MGUS_VS_PCL

There are 88 genes in this pathway. This pathway was detected by GSCA

BCR/ABL ALL
Major Gene (BCR/ABL): CCT3
Weight Factor: 1.565
Major Gene (NEG): CCT3
Weight Factor: 1.565

NEG ALL
Major Gene (NEG): CCT3
Weight Factor: 1.426
Major Gene (BCR/ABL): CCT3
Weight Factor: 1.426

MST2 of the coexpression network for BCR/ABL ALL

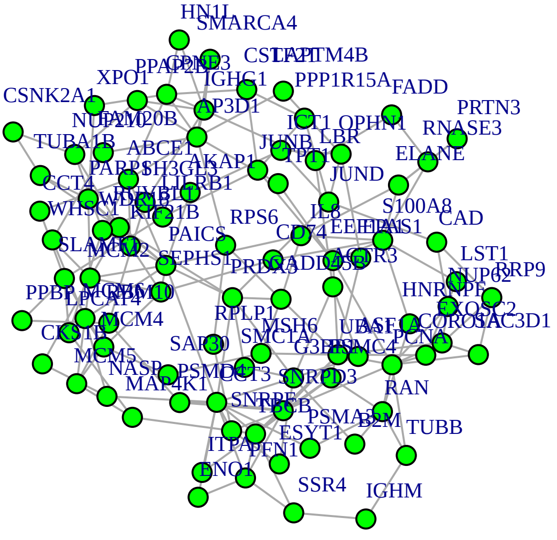

MST2 of the coexpression network for NEG ALL

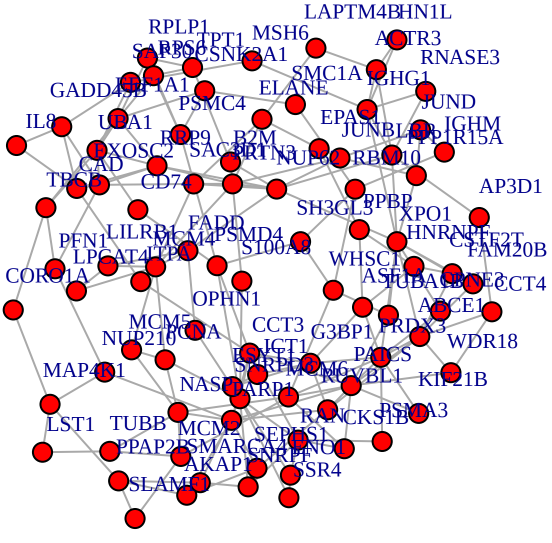

Pathway: MATTIOLI_MULTIPLE_MYELOMA_WITH_14Q32_TRANSLOCATIONS

There are 28 genes in this pathway. This pathway was detected by GSCA

BCR/ABL ALL
Major Gene (BCR/ABL): CCND2
Weight Factor: 1.272
Major Gene (NEG): SORT1
Weight Factor: 0.963

NEG ALL
Major Gene (NEG): SORT1
Weight Factor: 1.438
Major Gene (BCR/ABL): CCND2
Weight Factor: 0.846

MST2 of the coexpression network for BCR/ABL ALL

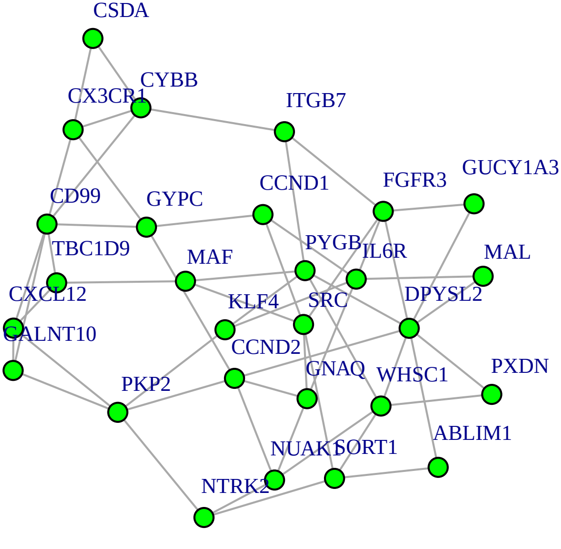

MST2 of the coexpression network for NEG ALL

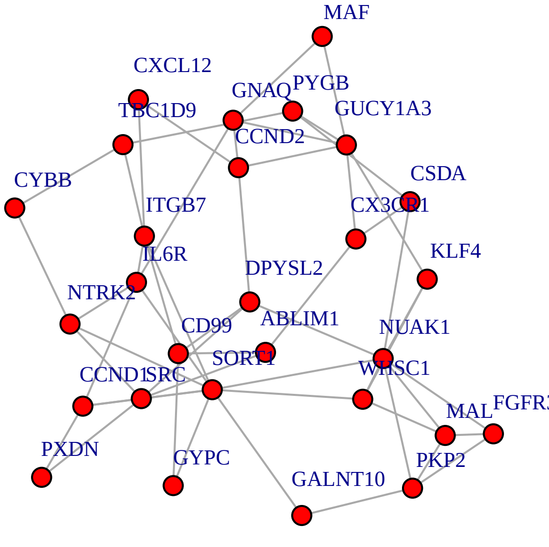

Pathway: FARMER_BREAST_CANCER_CLUSTER_7

There are 16 genes in this pathway. This pathway was detected by GSCA

BCR/ABL ALL
Major Gene (BCR/ABL): TMEM41B
Weight Factor: 1.279
Major Gene (NEG): MSX2
Weight Factor: 1.161

NEG ALL
Major Gene (NEG): MSX2
Weight Factor: 1.321
Major Gene (BCR/ABL): TMEM41B
Weight Factor: 1.211

MST2 of the coexpression network for BCR/ABL ALL

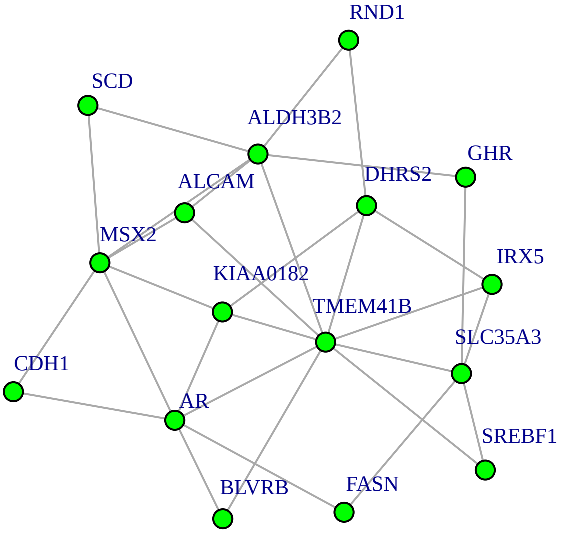

MST2 of the coexpression network for NEG ALL

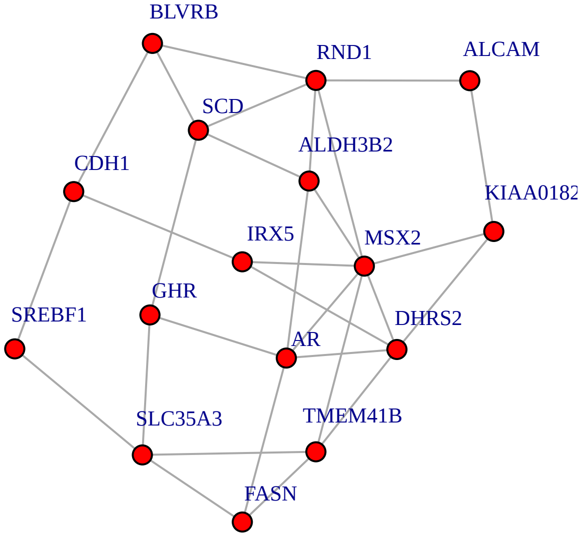

Pathway: LI_CISPLATIN_RESISTANCE_DN

There are 23 genes in this pathway. This pathway was detected by GSCA

BCR/ABL ALL
Major Gene (BCR/ABL): MMP10
Weight Factor: 1.353
Major Gene (NEG): TDO2
Weight Factor: 1.165

NEG ALL
Major Gene (NEG): TDO2
Weight Factor: 1.396
Major Gene (BCR/ABL): MMP10
Weight Factor: 1.08

MST2 of the coexpression network for BCR/ABL ALL

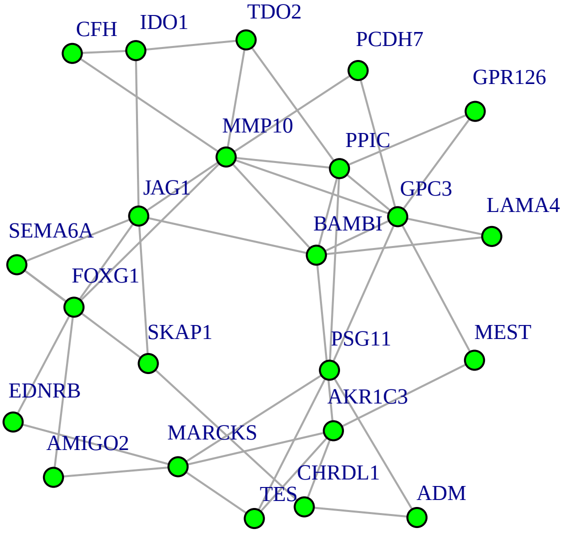

MST2 of the coexpression network for NEG ALL

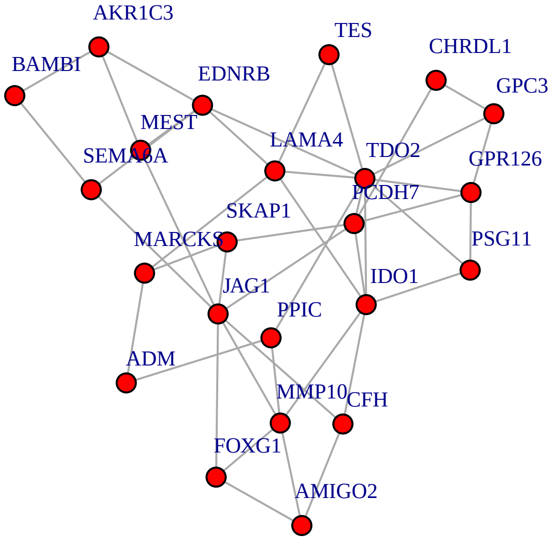

Pathway: XU_HGF_SIGNALING_NOT_VIA_AKT1_48HR_UP

There are 33 genes in this pathway. This pathway was detected by GSCA

BCR/ABL ALL
Major Gene (BCR/ABL): MACF1
Weight Factor: 1.422
Major Gene (NEG): PHLDA1
Weight Factor: 1.276

NEG ALL
Major Gene (NEG): PHLDA1
Weight Factor: 1.534
Major Gene (BCR/ABL): MACF1
Weight Factor: 1.34

MST2 of the coexpression network for BCR/ABL ALL

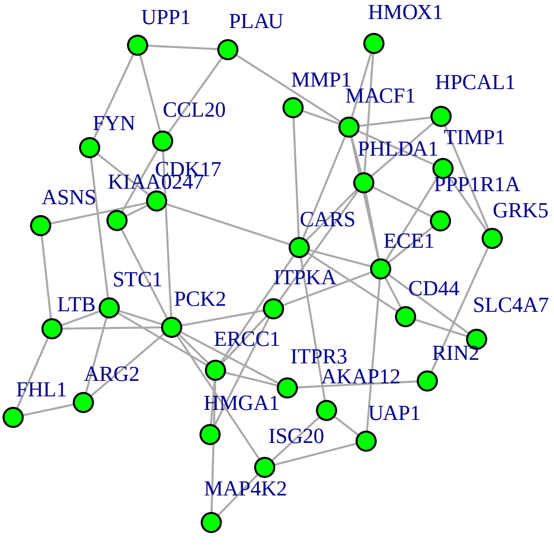

MST2 of the coexpression network for NEG ALL

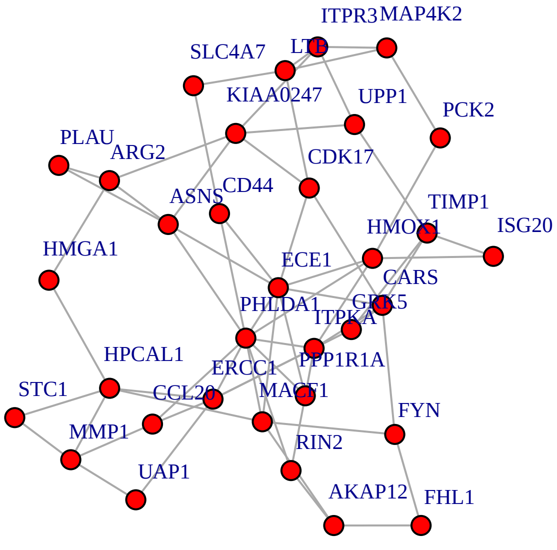

Pathway: DAUER_STAT3_TARGETS_DN

There are 26 genes in this pathway. This pathway was detected by GSCA

BCR/ABL ALL
Major Gene (BCR/ABL): ISG15
Weight Factor: 1.275
Major Gene (NEG): ISG15
Weight Factor: 1.275

NEG ALL
Major Gene (NEG): ISG15
Weight Factor: 1.351
Major Gene (BCR/ABL): ISG15
Weight Factor: 1.351

MST2 of the coexpression network for BCR/ABL ALL

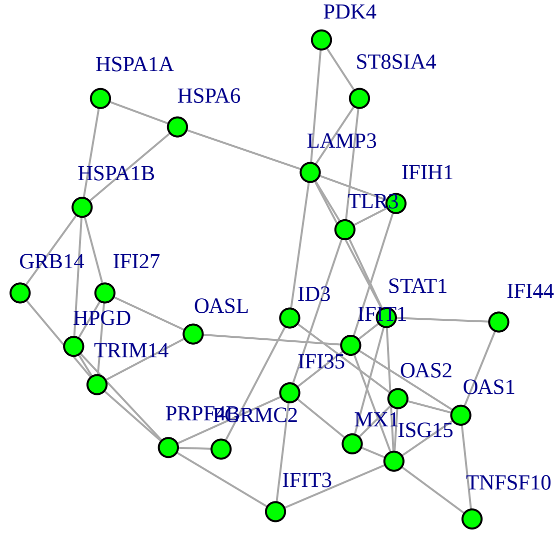

MST2 of the coexpression network for NEG ALL

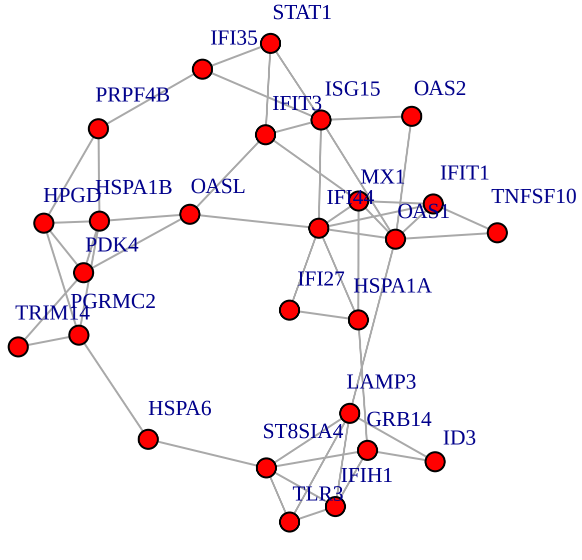

Pathway: WANG_METHYLATED_IN_BREAST_CANCER

There are 31 genes in this pathway. This pathway was detected by GSCA

BCR/ABL ALL
Major Gene (BCR/ABL): FOSL1
Weight Factor: 1.273
Major Gene (NEG): FOSL1
Weight Factor: 1.273

NEG ALL
Major Gene (NEG): FOSL1
Weight Factor: 1.465
Major Gene (BCR/ABL): FOSL1
Weight Factor: 1.465

MST2 of the coexpression network for BCR/ABL ALL

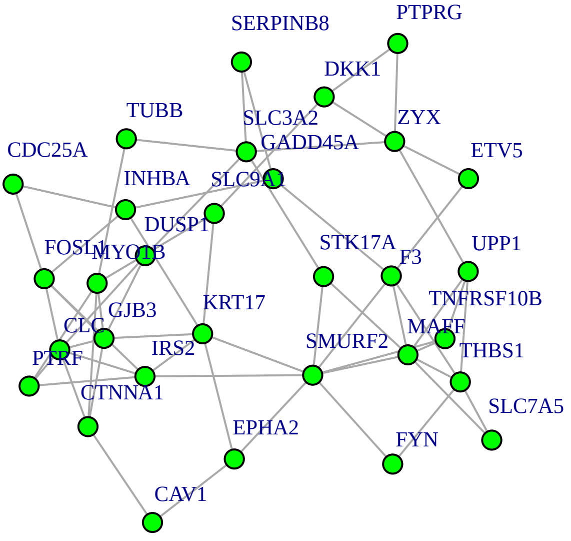

MST2 of the coexpression network for NEG ALL

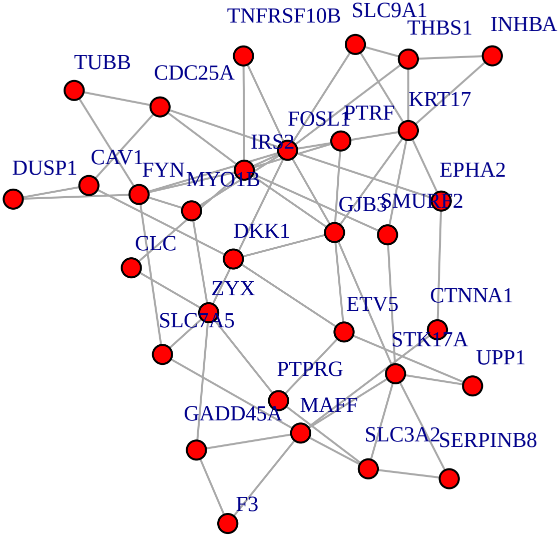

Pathway: DIRMEIER_LMP1_RESPONSE_LATE_DN

There are 25 genes in this pathway. This pathway was detected by GSCA

BCR/ABL ALL
Major Gene (BCR/ABL): RPL29
Weight Factor: 1.399
Major Gene (NEG): RPL29
Weight Factor: 1.399

NEG ALL
Major Gene (NEG): RPL29
Weight Factor: 1.519
Major Gene (BCR/ABL): RPL29
Weight Factor: 1.519

MST2 of the coexpression network for BCR/ABL ALL

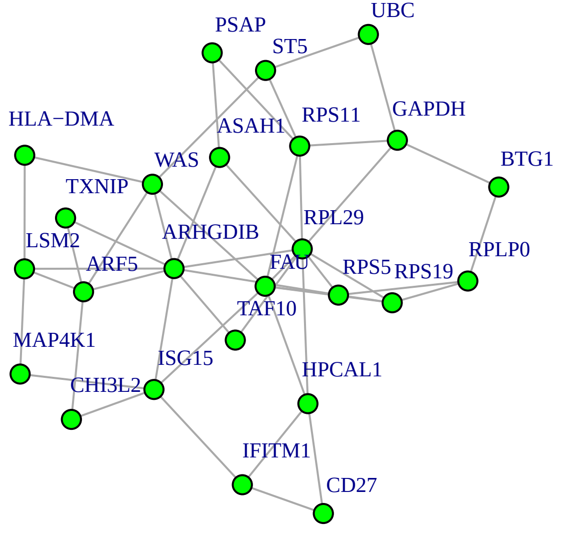

MST2 of the coexpression network for NEG ALL

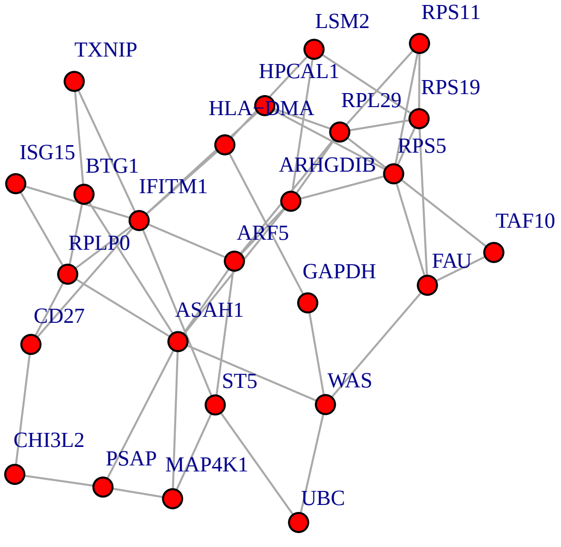

Pathway: RUGO_ENVIRONMENTAL_STRESS_RESPONSE_UP

There are 27 genes in this pathway. This pathway was detected by GSCA

BCR/ABL ALL
Major Gene (BCR/ABL): FGFR4
Weight Factor: 1.37
Major Gene (NEG): FGFR4
Weight Factor: 1.37

NEG ALL
Major Gene (NEG): FGFR4
Weight Factor: 1.497
Major Gene (BCR/ABL): FGFR4
Weight Factor: 1.497

MST2 of the coexpression network for BCR/ABL ALL

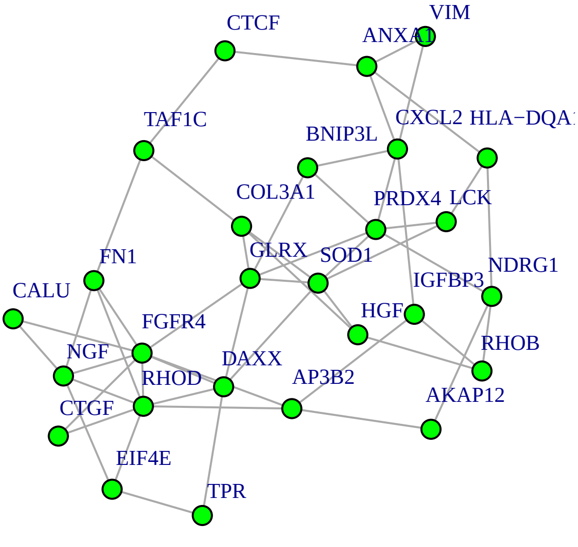

MST2 of the coexpression network for NEG ALL

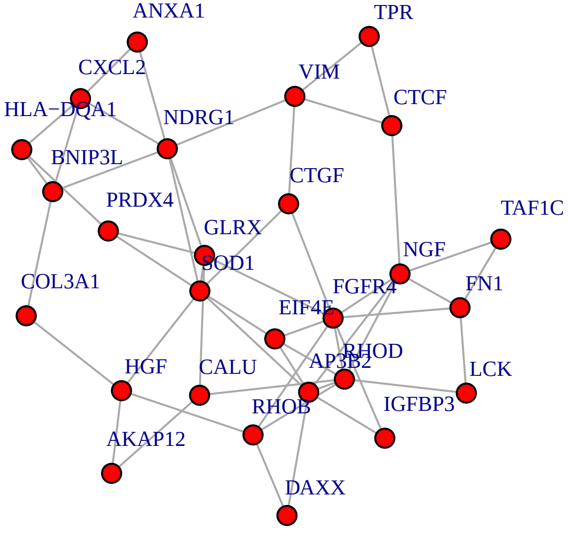

Pathway: WOOD_EBV_EBNA1_TARGETS_DN

There are 41 genes in this pathway. This pathway was detected by GSCA

BCR/ABL ALL
Major Gene (BCR/ABL): WISP2
Weight Factor: 1.373
Major Gene (NEG): WISP2
Weight Factor: 1.373

NEG ALL
Major Gene (NEG): WISP2
Weight Factor: 1.439
Major Gene (BCR/ABL): WISP2
Weight Factor: 1.439

MST2 of the coexpression network for BCR/ABL ALL

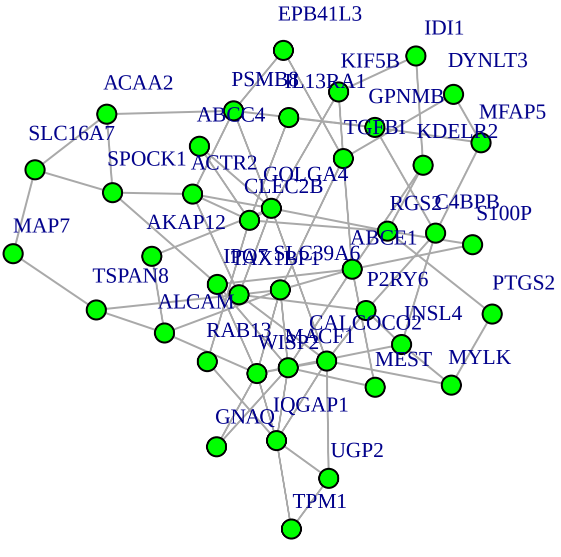

MST2 of the coexpression network for NEG ALL

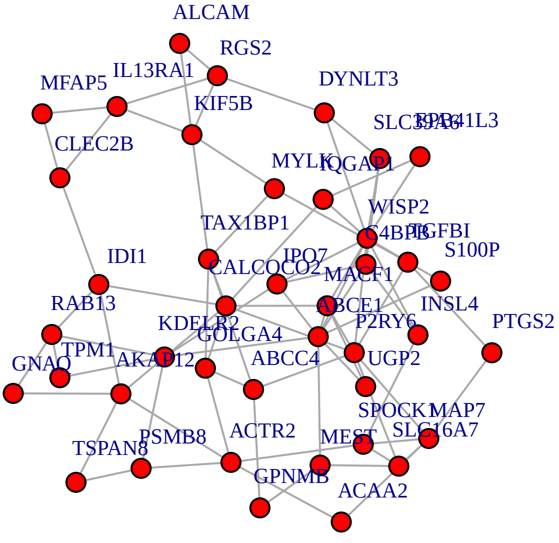

Pathway: GRUETZMANN_PANCREATIC_CANCER_UP

There are 334 genes in this pathway. This pathway was detected by GSCA

BCR/ABL ALL
Major Gene (BCR/ABL): MRPS12
Weight Factor: 1.441
Major Gene (NEG): XPO1
Weight Factor: 0.932

NEG ALL
Major Gene (NEG): XPO1
Weight Factor: 1.533
Major Gene (BCR/ABL): MRPS12
Weight Factor: 1.13

MST2 of the coexpression network for BCR/ABL ALL

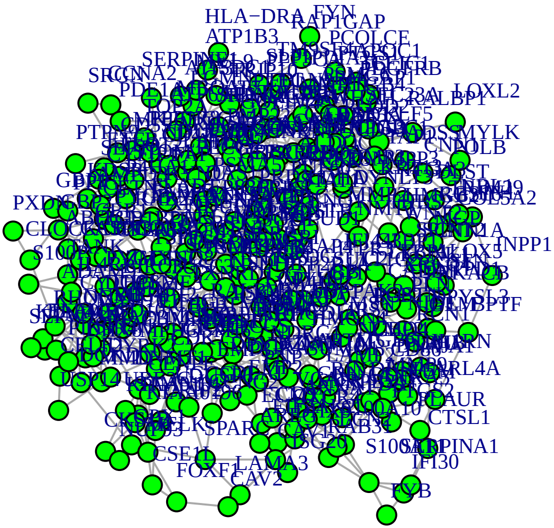

MST2 of the coexpression network for NEG ALL

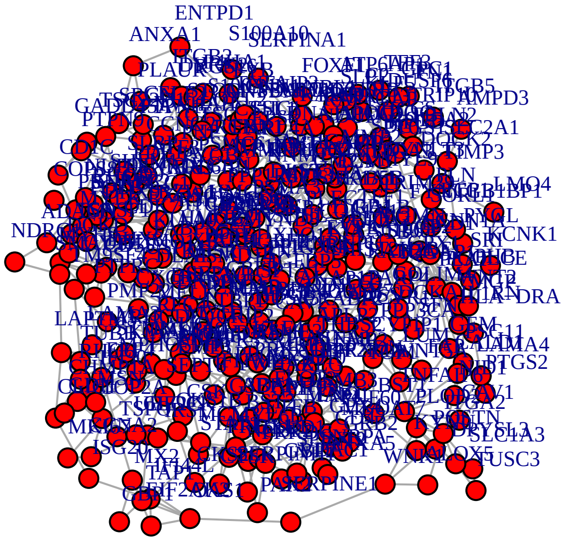

Pathway: WATTEL_AUTONOMOUS_THYROID_ADENOMA_DN

There are 17 genes in this pathway. This pathway was detected by GSCA

BCR/ABL ALL
Major Gene (BCR/ABL): DUSP2
Weight Factor: 1.317
Major Gene (NEG): ATF3
Weight Factor: 1.035

NEG ALL
Major Gene (NEG): ATF3
Weight Factor: 1.358
Major Gene (BCR/ABL): DUSP2
Weight Factor: 1.129

MST2 of the coexpression network for BCR/ABL ALL

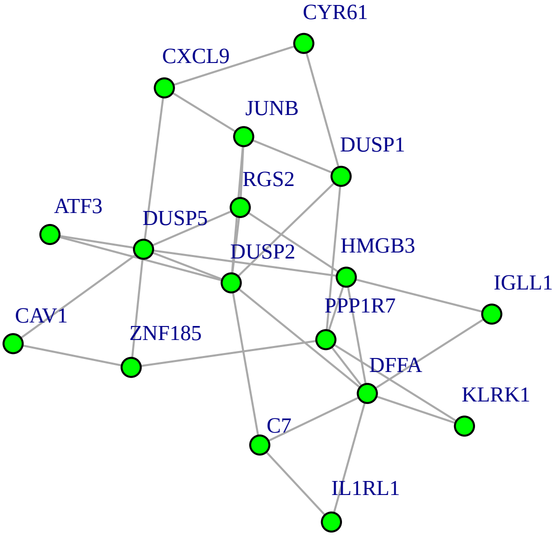

MST2 of the coexpression network for NEG ALL

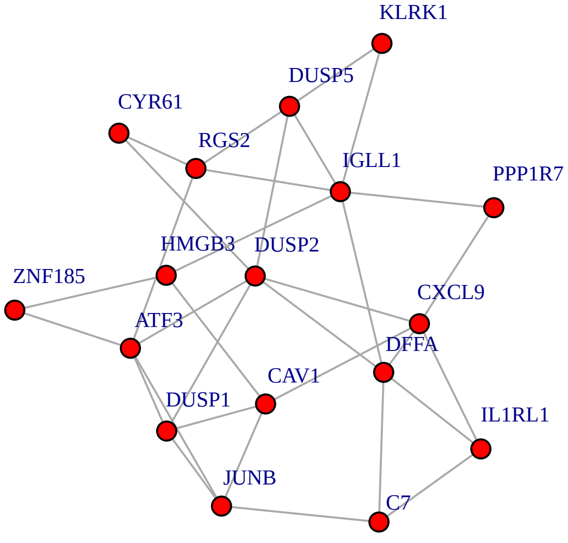

Pathway: OHM_METHYLATED_IN_ADULT_CANCERS

There are 21 genes in this pathway. This pathway was detected by GSCA

BCR/ABL ALL
Major Gene (BCR/ABL): TIMP3
Weight Factor: 1.438
Major Gene (NEG): GATA4
Weight Factor: 1.326

NEG ALL
Major Gene (NEG): GATA4
Weight Factor: 1.433
Major Gene (BCR/ABL): TIMP3
Weight Factor: 1.211

MST2 of the coexpression network for BCR/ABL ALL

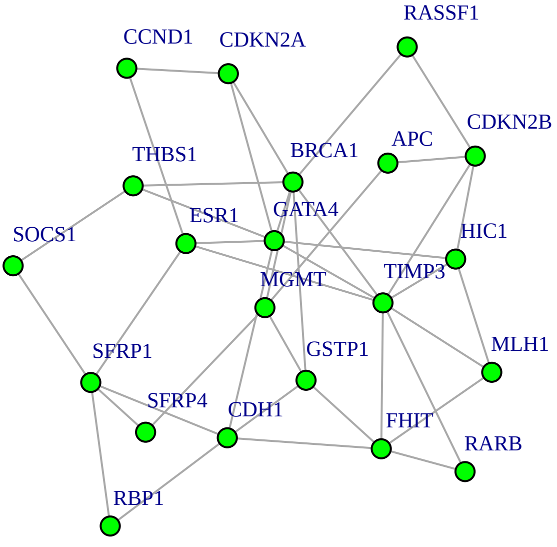

MST2 of the coexpression network for NEG ALL

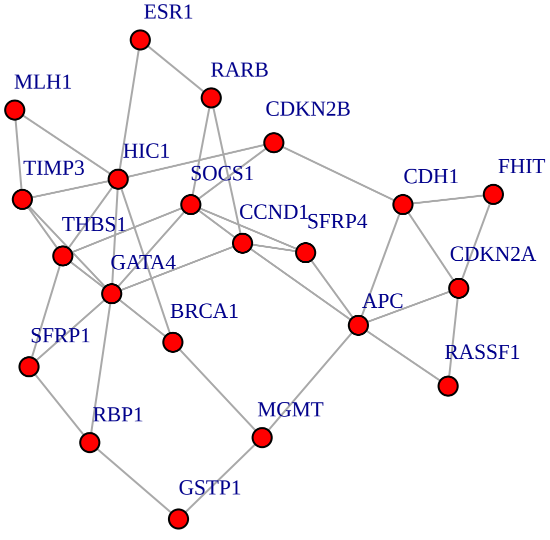

Pathway: ALCALA_APOPTOSIS

There are 70 genes in this pathway. This pathway was detected by GSCA

BCR/ABL ALL
Major Gene (BCR/ABL): RAC2
Weight Factor: 1.449
Major Gene (NEG): MARK2
Weight Factor: 1.191

NEG ALL
Major Gene (NEG): MARK2
Weight Factor: 1.53
Major Gene (BCR/ABL): RAC2
Weight Factor: 1.148

MST2 of the coexpression network for BCR/ABL ALL

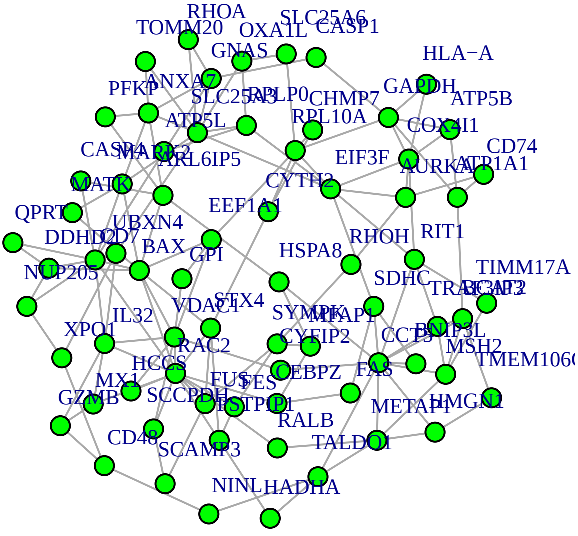

MST2 of the coexpression network for NEG ALL

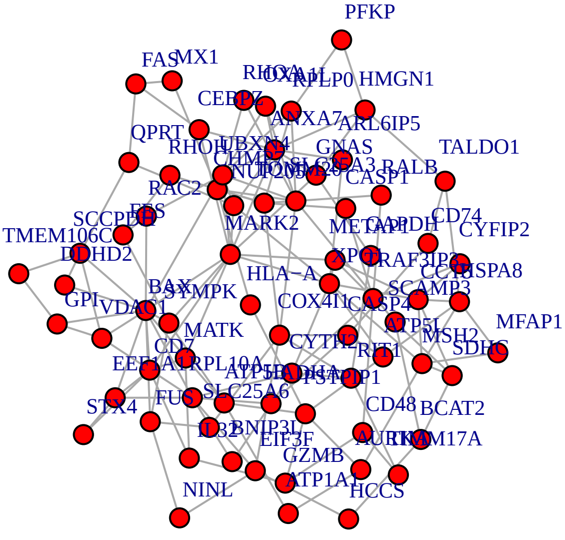

Pathway: LIU_NASOPHARYNGEAL_CARCINOMA

There are 49 genes in this pathway. This pathway was detected by GSCA

BCR/ABL ALL
Major Gene (BCR/ABL): BTF3
Weight Factor: 1.269
Major Gene (NEG): EIF1AX
Weight Factor: 0.946

NEG ALL
Major Gene (NEG): EIF1AX
Weight Factor: 1.451
Major Gene (BCR/ABL): BTF3
Weight Factor: 0.888

MST2 of the coexpression network for BCR/ABL ALL

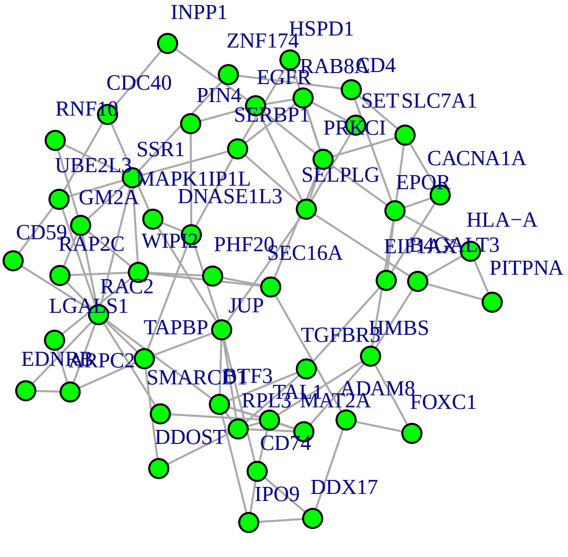

MST2 of the coexpression network for NEG ALL

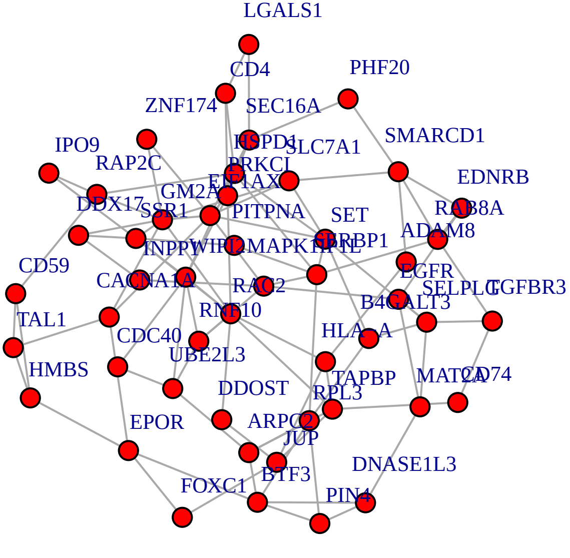

Pathway: LIAO_HAVE_SOX4_BINDING_SITES

There are 23 genes in this pathway. This pathway was detected by GSCA

BCR/ABL ALL
Major Gene (BCR/ABL): PFKFB4
Weight Factor: 1.327
Major Gene (NEG): PFKFB4
Weight Factor: 1.327

NEG ALL
Major Gene (NEG): PFKFB4
Weight Factor: 1.454
Major Gene (BCR/ABL): PFKFB4
Weight Factor: 1.454

MST2 of the coexpression network for BCR/ABL ALL

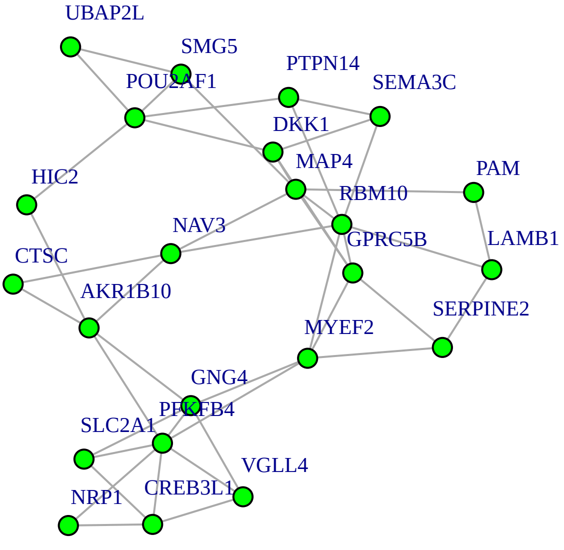

MST2 of the coexpression network for NEG ALL

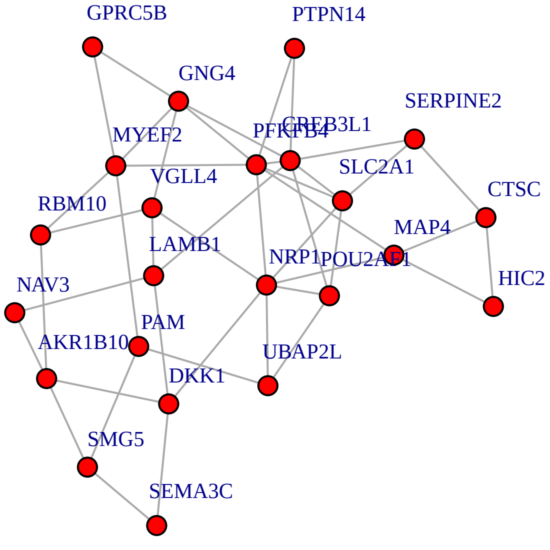

Pathway: CAFFAREL_RESPONSE_TO_THC_24HR_5_UP

There are 21 genes in this pathway. This pathway was detected by GSCA

BCR/ABL ALL
Major Gene (BCR/ABL): PCNA
Weight Factor: 1.443
Major Gene (NEG): IARS
Weight Factor: 1.015

NEG ALL
Major Gene (NEG): IARS
Weight Factor: 1.364
Major Gene (BCR/ABL): PCNA
Weight Factor: 1.242

MST2 of the coexpression network for BCR/ABL ALL

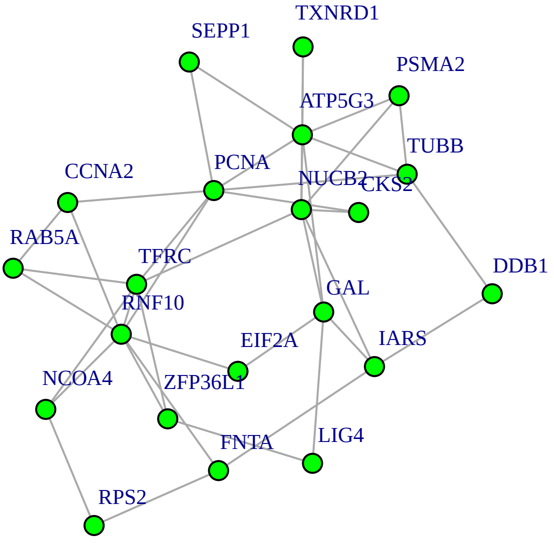

MST2 of the coexpression network for NEG ALL

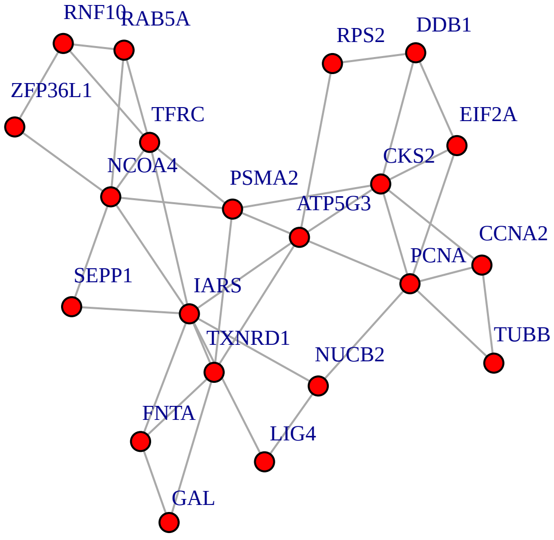

Pathway: BERTUCCI_INVASIVE_CARCINOMA_DUCTAL_VS_LOBULAR_DN

There are 36 genes in this pathway. This pathway was detected by GSCA

BCR/ABL ALL
Major Gene (BCR/ABL): ADAM11
Weight Factor: 1.258
Major Gene (NEG): OMD
Weight Factor: 1.191

NEG ALL
Major Gene (NEG): OMD
Weight Factor: 1.347
Major Gene (BCR/ABL): ADAM11
Weight Factor: 1.345

MST2 of the coexpression network for BCR/ABL ALL

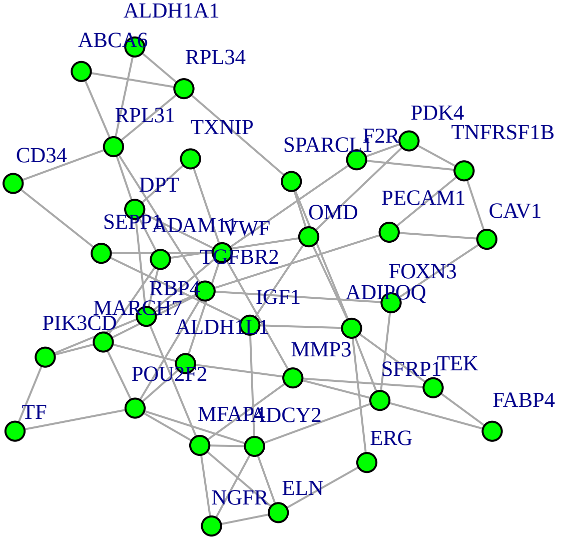

MST2 of the coexpression network for NEG ALL

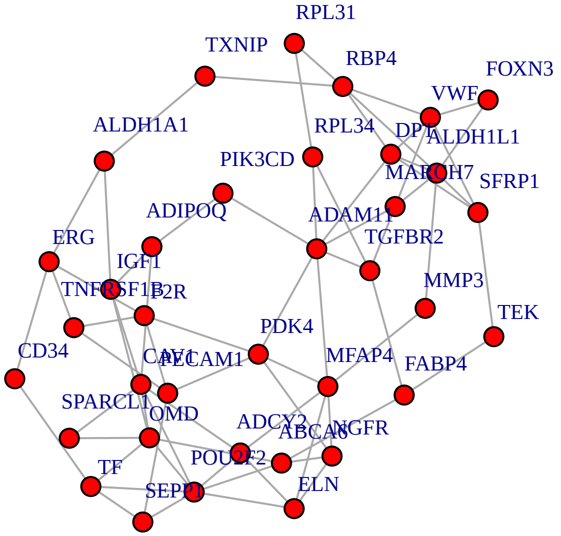

Pathway: FRIDMAN_SENESCENCE_UP

There are 75 genes in this pathway. This pathway was detected by GSCA

BCR/ABL ALL
Major Gene (BCR/ABL): TP53
Weight Factor: 1.42
Major Gene (NEG): RAB5B
Weight Factor: 1.221

NEG ALL
Major Gene (NEG): RAB5B
Weight Factor: 1.527
Major Gene (BCR/ABL): TP53
Weight Factor: 0.995

MST2 of the coexpression network for BCR/ABL ALL

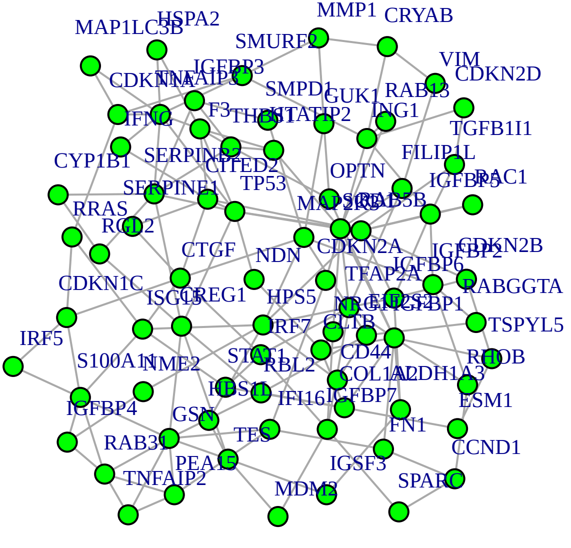

MST2 of the coexpression network for NEG ALL

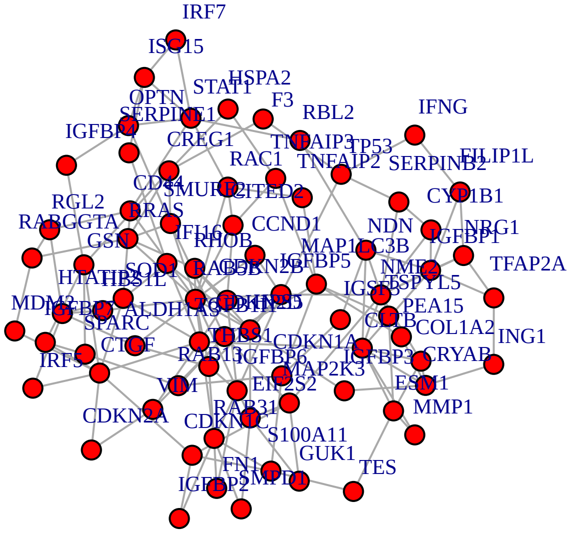

Pathway: SAKAI_TUMOR_INFILTRATING_MONOCYTES_DN

There are 68 genes in this pathway. This pathway was detected by GSCA

BCR/ABL ALL
Major Gene (BCR/ABL): PRDX1
Weight Factor: 1.354
Major Gene (NEG): HNRNPA2B1
Weight Factor: 1.138

NEG ALL
Major Gene (NEG): HNRNPA2B1
Weight Factor: 1.45
Major Gene (BCR/ABL): PRDX1
Weight Factor: 1.13

MST2 of the coexpression network for BCR/ABL ALL

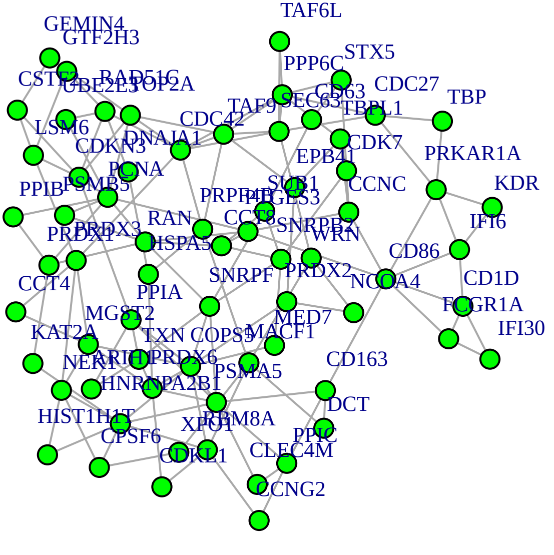

MST2 of the coexpression network for NEG ALL

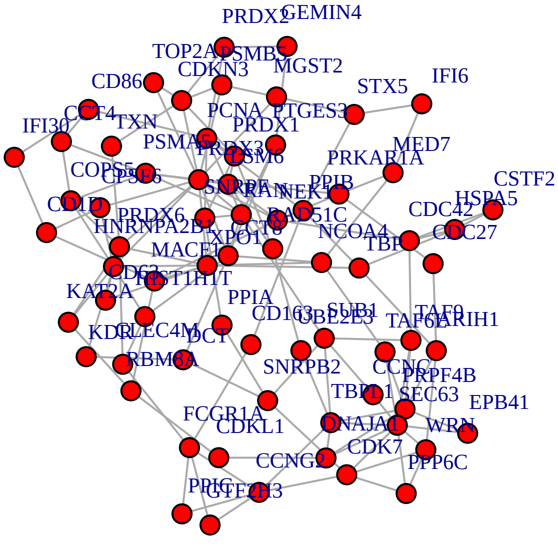

Pathway: MORI_LARGE_PRE_BII_LYMPHOCYTE_DN

There are 41 genes in this pathway. This pathway was detected by GSCA

BCR/ABL ALL
Major Gene (BCR/ABL): CD22
Weight Factor: 1.295
Major Gene (NEG): ADCY7
Weight Factor: 1.067

MST2 of the coexpression network for BCR/ABL ALL

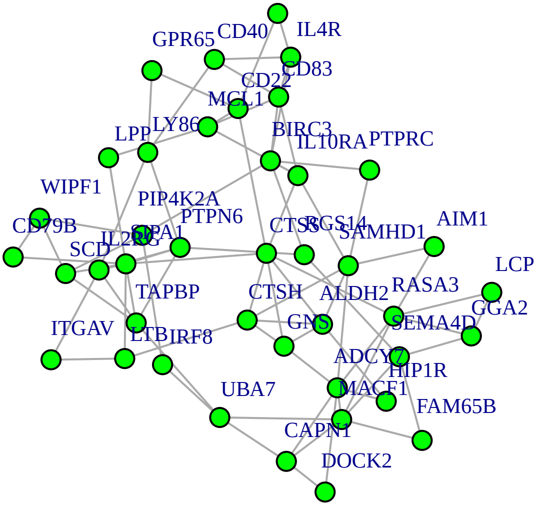

NEG ALL
Major Gene (NEG): ADCY7
Weight Factor: 1.375
Major Gene (BCR/ABL): CD22
Weight Factor: 0.706

MST2 of the coexpression network for NEG ALL

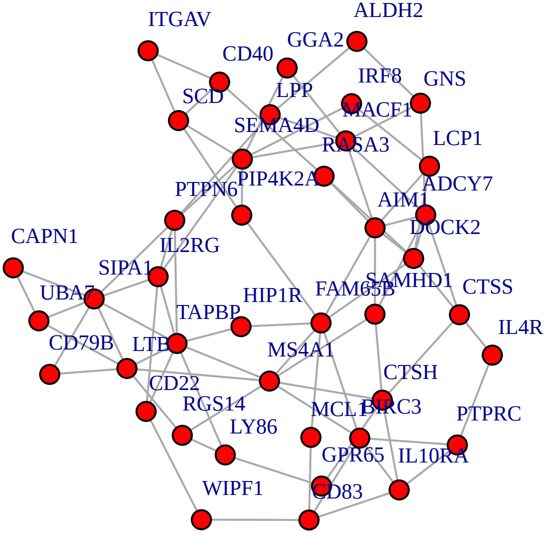

Pathway: MORI_MATURE_B_LYMPHOCYTE_UP

There are 52 genes in this pathway. This pathway was detected by GSCA

BCR/ABL ALL
Major Gene (BCR/ABL): NCF4
Weight Factor: 1.374
Major Gene (NEG): AIM1
Weight Factor: 0.9

NEG ALL
Major Gene (NEG): AIM1
Weight Factor: 1.461
Major Gene (BCR/ABL): NCF4
Weight Factor: 1.105

MST2 of the coexpression network for BCR/ABL ALL

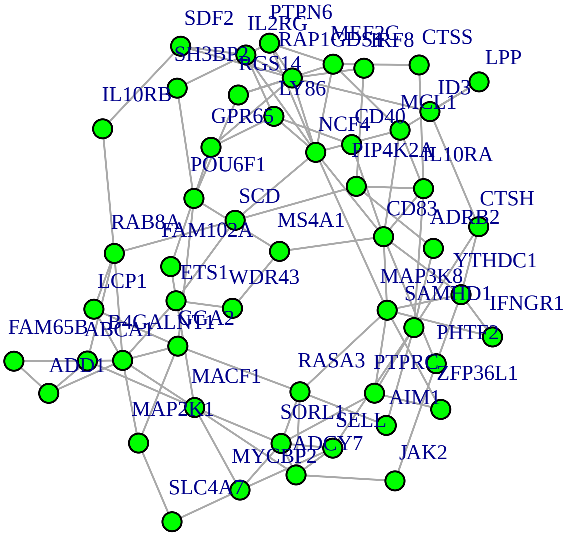

MST2 of the coexpression network for NEG ALL

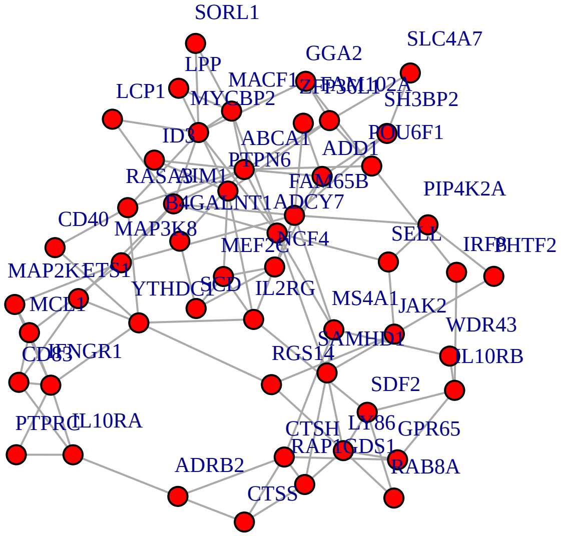

Pathway: COLLIS_PRKDC_SUBSTRATES

There are 16 genes in this pathway. This pathway was detected by GSCA

BCR/ABL ALL
Major Gene (BCR/ABL): IGHG1
Weight Factor: 1.298
Major Gene (NEG): SNRNP200
Weight Factor: 1.133

NEG ALL
Major Gene (NEG): SNRNP200
Weight Factor: 1.356
Major Gene (BCR/ABL): IGHG1
Weight Factor: 1.215

MST2 of the coexpression network for BCR/ABL ALL

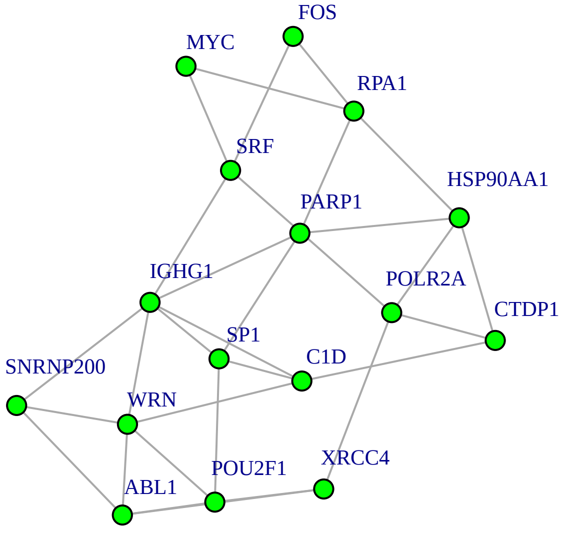

MST2 of the coexpression network for NEG ALL

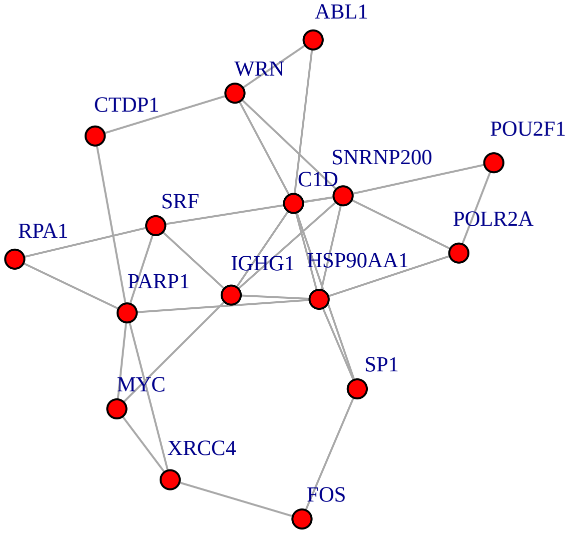

Pathway: DING_LUNG_CANCER_EXPRESSION_BY_COPY_NUMBER

There are 61 genes in this pathway. This pathway was detected by GSCA

BCR/ABL ALL
Major Gene (BCR/ABL): NUP153
Weight Factor: 1.536
Major Gene (NEG): NUP153
Weight Factor: 1.536

NEG ALL
Major Gene (NEG): NUP153
Weight Factor: 1.426
Major Gene (BCR/ABL): NUP153
Weight Factor: 1.426

MST2 of the coexpression network for BCR/ABL ALL

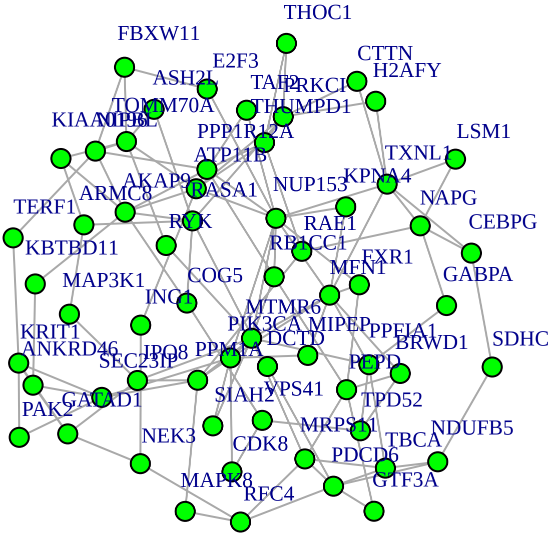

MST2 of the coexpression network for NEG ALL

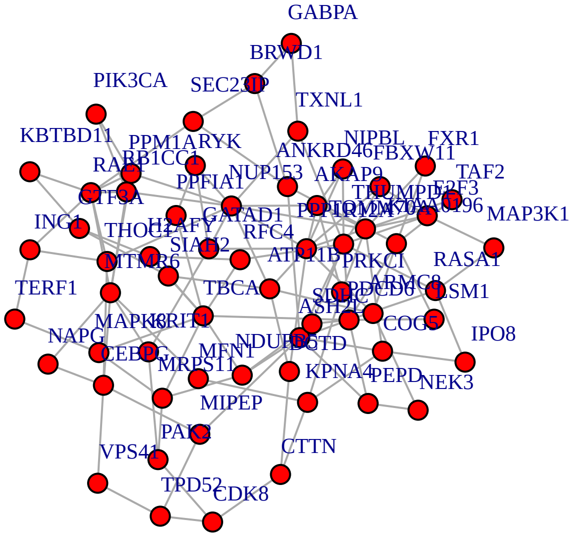

Pathway: ROSS_ACUTE_MYELOID_LEUKEMIA_CBF

There are 59 genes in this pathway. This pathway was detected by GSCA

BCR/ABL ALL
Major Gene (BCR/ABL): HYAL2
Weight Factor: 1.316
Major Gene (NEG): ITGB4
Weight Factor: 1.291

NEG ALL
Major Gene (NEG): ITGB4
Weight Factor: 1.419
Major Gene (BCR/ABL): HYAL2
Weight Factor: 1.366

MST2 of the coexpression network for BCR/ABL ALL

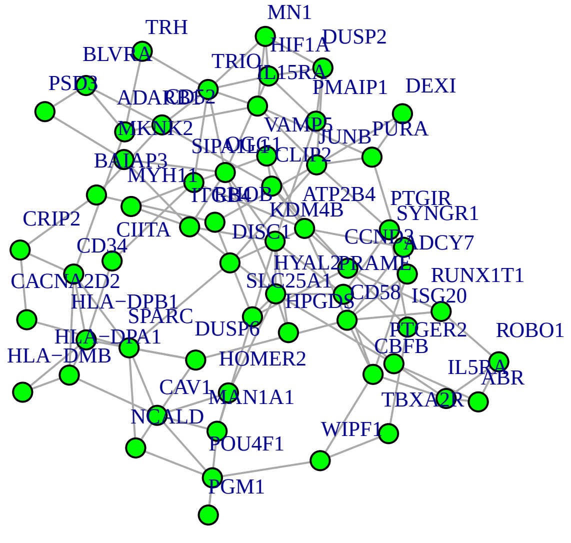

MST2 of the coexpression network for NEG ALL

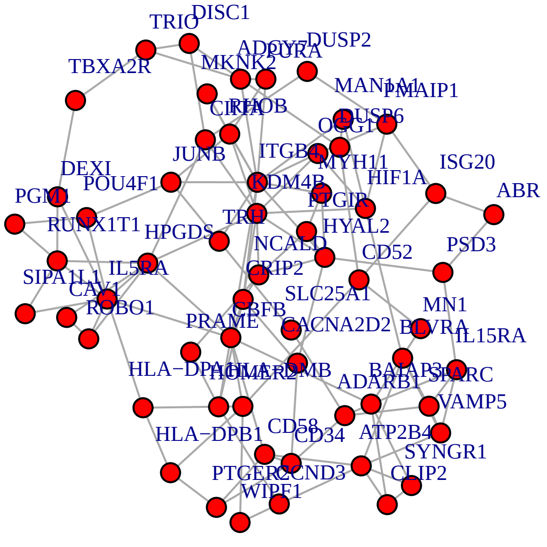

Pathway: SWEET_KRAS_TARGETS_DN

There are 26 genes in this pathway. This pathway was detected by GSCA

BCR/ABL ALL
Major Gene (BCR/ABL): FKBP2
Weight Factor: 1.347
Major Gene (NEG): TGOLN2
Weight Factor: 0.836

NEG ALL
Major Gene (NEG): TGOLN2
Weight Factor: 1.253
Major Gene (BCR/ABL): FKBP2
Weight Factor: 1.199

MST2 of the coexpression network for BCR/ABL ALL

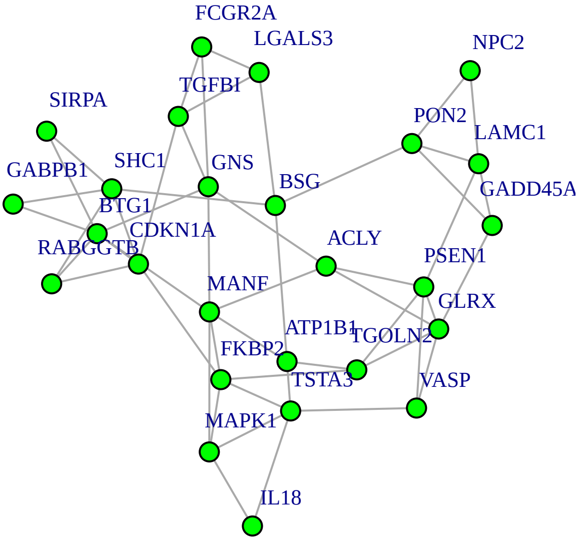

MST2 of the coexpression network for NEG ALL

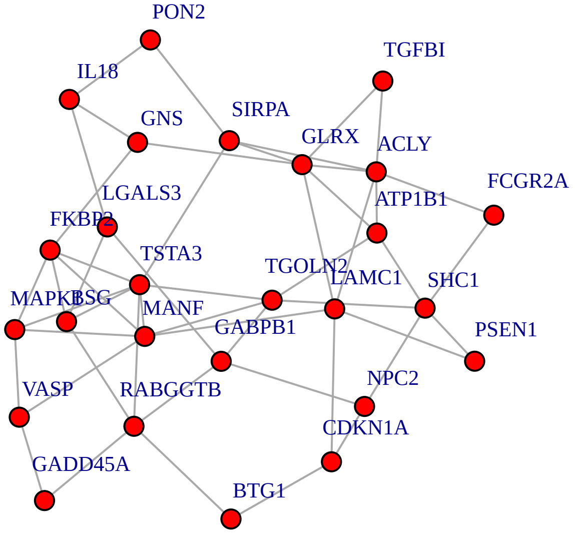

Pathway: ROSS_AML_WITH_CBFB_MYH11_FUSION

There are 44 genes in this pathway. This pathway was detected by GSCA

BCR/ABL ALL
Major Gene (BCR/ABL): SLC7A7
Weight Factor: 1.524
Major Gene (NEG): CLEC10A
Weight Factor: 1.05

NEG ALL
Major Gene (NEG): CLEC10A
Weight Factor: 1.401
Major Gene (BCR/ABL): SLC7A7
Weight Factor: 0.876

MST2 of the coexpression network for BCR/ABL ALL

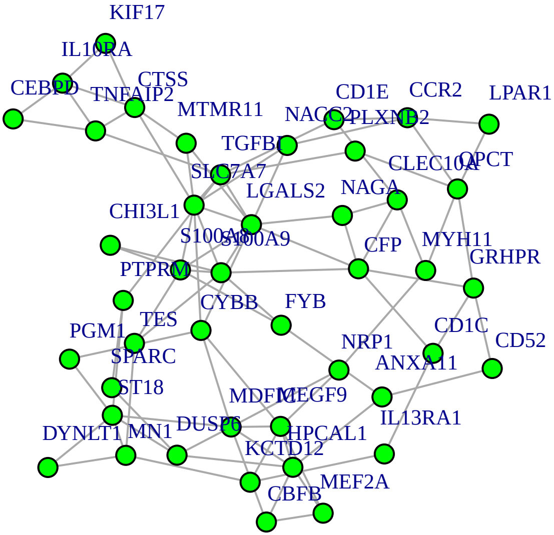

MST2 of the coexpression network for NEG ALL

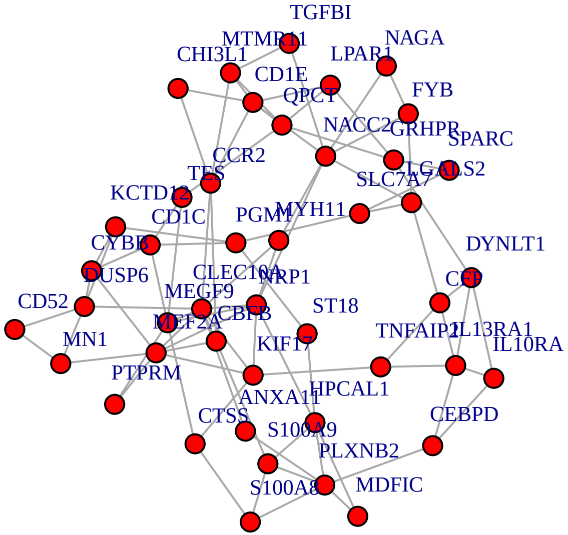

Pathway: GNATENKO_PLATELET_SIGNATURE

There are 31 genes in this pathway. This pathway was detected by GSCA

BCR/ABL ALL
Major Gene (BCR/ABL): ITGA2B
Weight Factor: 1.408
Major Gene (NEG): GNAS
Weight Factor: 0.827

NEG ALL
Major Gene (NEG): GNAS
Weight Factor: 1.298
Major Gene (BCR/ABL): ITGA2B
Weight Factor: 1.154

MST2 of the coexpression network for BCR/ABL ALL

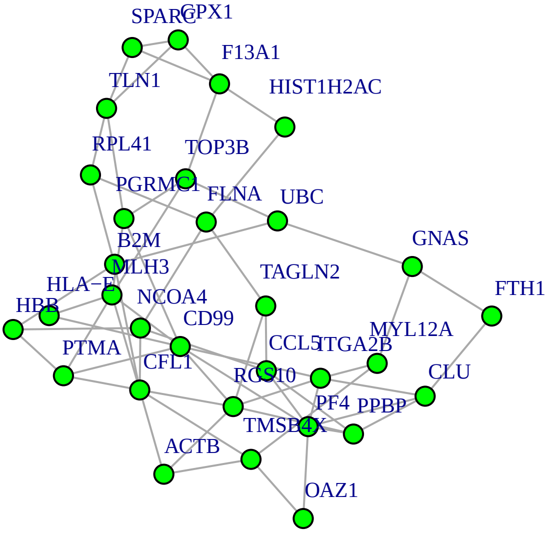

MST2 of the coexpression network for NEG ALL

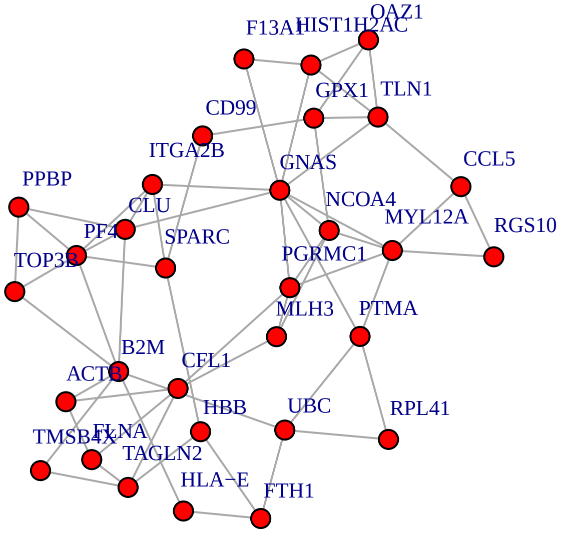

Pathway: DER_IFN_ALPHA_RESPONSE_UP

There are 56 genes in this pathway. This pathway was detected by GSCA

BCR/ABL ALL
Major Gene (BCR/ABL): IFI35
Weight Factor: 1.378
Major Gene (NEG): MX2
Weight Factor: 1.09

MST2 of the coexpression network for BCR/ABL ALL

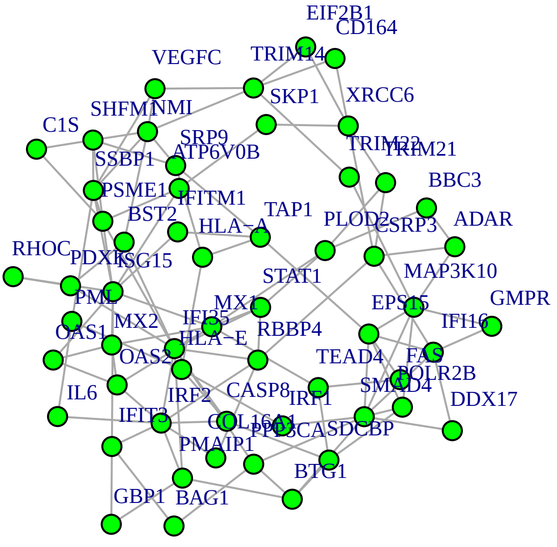

NEG ALL
Major Gene (NEG): MX2
Weight Factor: 1.351
Major Gene (BCR/ABL): IFI35
Weight Factor: 1.258

MST2 of the coexpression network for NEG ALL

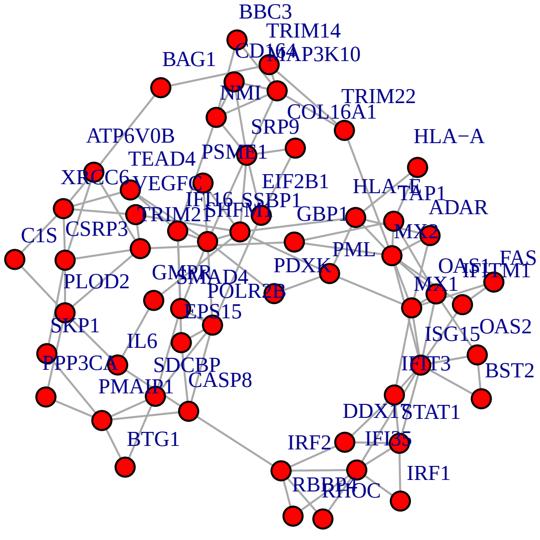

Pathway: KLEIN_PRIMARY_EFFUSION_LYMPHOMA_DN

There are 57 genes in this pathway. This pathway was detected by GSCA

BCR/ABL ALL
Major Gene (BCR/ABL): CAPG
Weight Factor: 1.362
Major Gene (NEG): DEK
Weight Factor: 1.022

NEG ALL
Major Gene (NEG): DEK
Weight Factor: 1.505
Major Gene (BCR/ABL): CAPG
Weight Factor: 0.794

MST2 of the coexpression network for BCR/ABL ALL

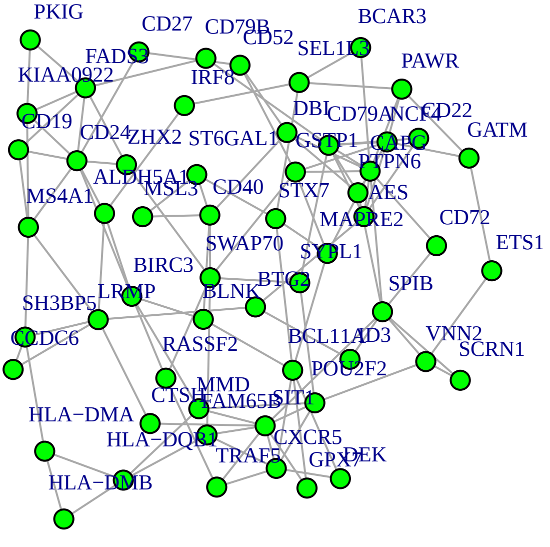

MST2 of the coexpression network for NEG ALL

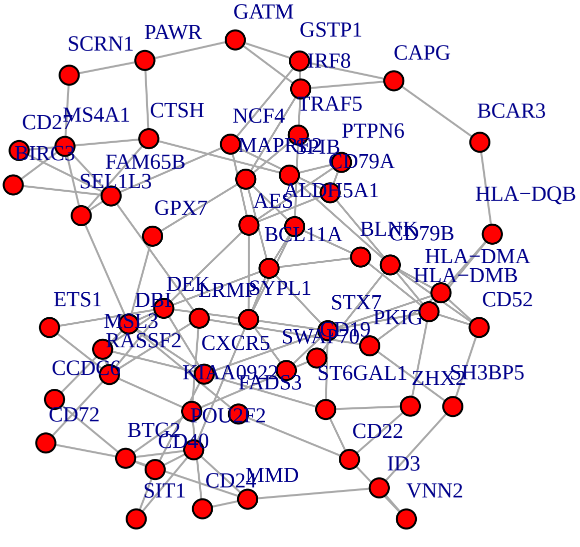

Pathway: TARTE_PLASMA_CELL_VS_B_LYMPHOCYTE_DN

There are 37 genes in this pathway. This pathway was detected by GSCA

BCR/ABL ALL
Major Gene (BCR/ABL): NFKB1
Weight Factor: 1.409
Major Gene (NEG): KIAA0430
Weight Factor: 1.332

NEG ALL
Major Gene (NEG): KIAA0430
Weight Factor: 1.429
Major Gene (BCR/ABL): NFKB1
Weight Factor: 0.962

MST2 of the coexpression network for BCR/ABL ALL

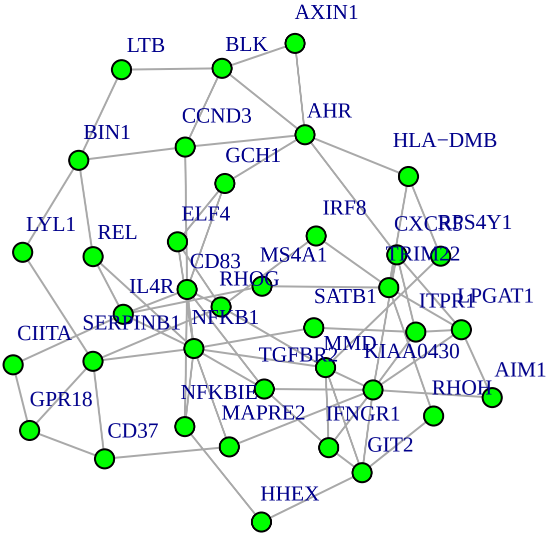

MST2 of the coexpression network for NEG ALL

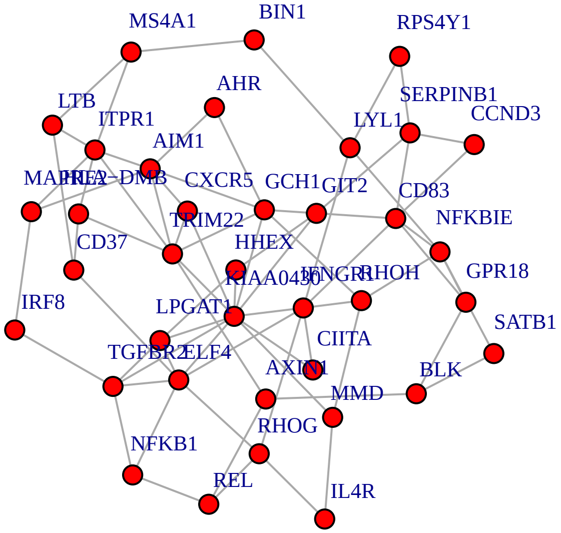

Pathway: FLECHNER_BIOPSY_KIDNEY_TRANSPLANT_REJECTED_VS_OK_UP

There are 86 genes in this pathway. This pathway was detected by GSCA

BCR/ABL ALL
Major Gene (BCR/ABL): TYROBP
Weight Factor: 1.502
Major Gene (NEG): RAB31
Weight Factor: 1.283

NEG ALL
Major Gene (NEG): RAB31
Weight Factor: 1.488
Major Gene (BCR/ABL): TYROBP
Weight Factor: 0.907

MST2 of the coexpression network for BCR/ABL ALL

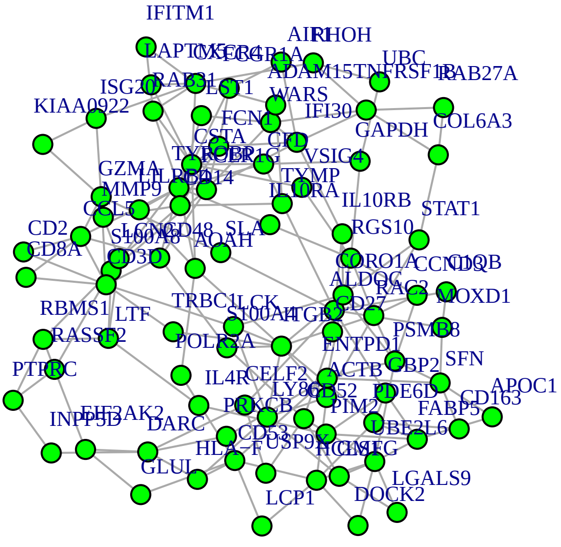

MST2 of the coexpression network for NEG ALL

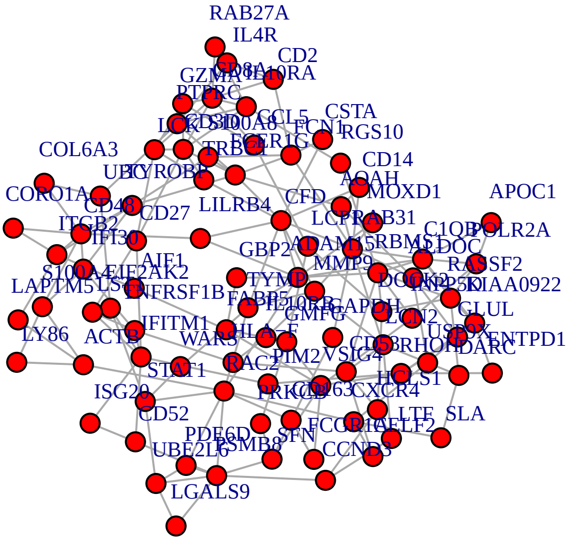

Pathway: WIELAND_UP_BY_HBV_INFECTION

There are 79 genes in this pathway. This pathway was detected by GSCA

BCR/ABL ALL
Major Gene (BCR/ABL): WARS
Weight Factor: 1.311
Major Gene (NEG): PSMB9
Weight Factor: 1.196

NEG ALL
Major Gene (NEG): PSMB9
Weight Factor: 1.405
Major Gene (BCR/ABL): WARS
Weight Factor: 0.768

MST2 of the coexpression network for BCR/ABL ALL

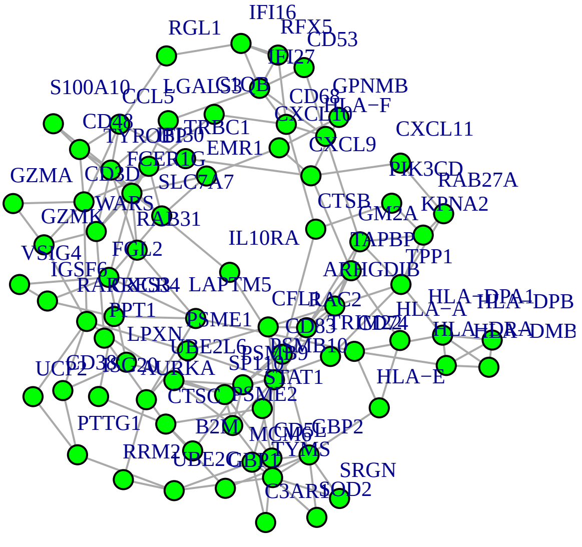

MST2 of the coexpression network for NEG ALL

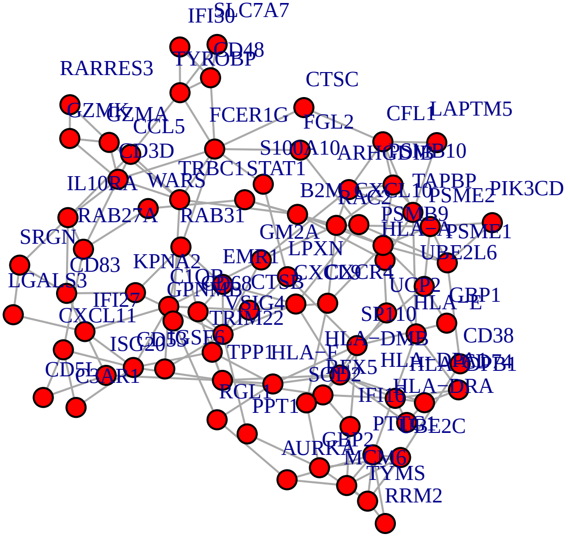

Pathway: DER_IFN_BETA_RESPONSE_UP

There are 81 genes in this pathway. This pathway was detected by GSCA

BCR/ABL ALL
Major Gene (BCR/ABL): IFI35
Weight Factor: 1.331
Major Gene (NEG): PTPN11
Weight Factor: 1.207

NEG ALL
Major Gene (NEG): PTPN11
Weight Factor: 1.426
Major Gene (BCR/ABL): IFI35
Weight Factor: 1.178

MST2 of the coexpression network for BCR/ABL ALL

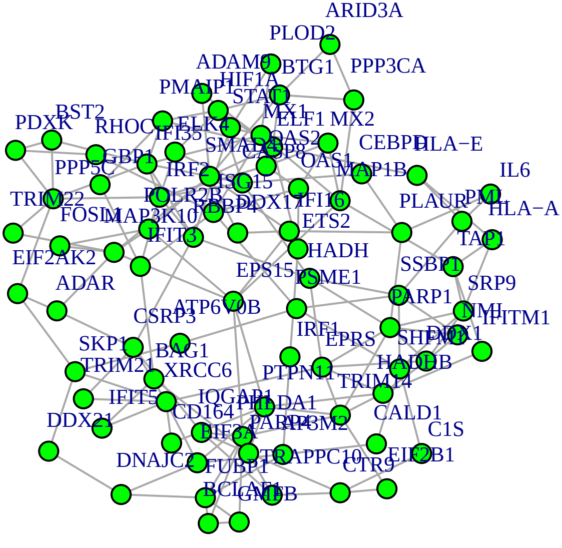

MST2 of the coexpression network for NEG ALL

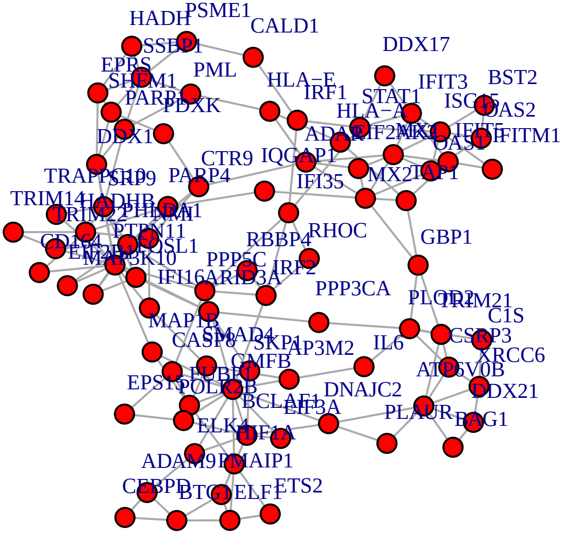

Pathway: FRASOR_RESPONSE_TO_ESTRADIOL_DN

There are 62 genes in this pathway. This pathway was detected by GSCA

BCR/ABL ALL
Major Gene (BCR/ABL): BCAS1
Weight Factor: 1.296
Major Gene (NEG): BMP4
Weight Factor: 1.167

NEG ALL
Major Gene (NEG): BMP4
Weight Factor: 1.325
Major Gene (BCR/ABL): BCAS1
Weight Factor: 1.081

MST2 of the coexpression network for BCR/ABL ALL

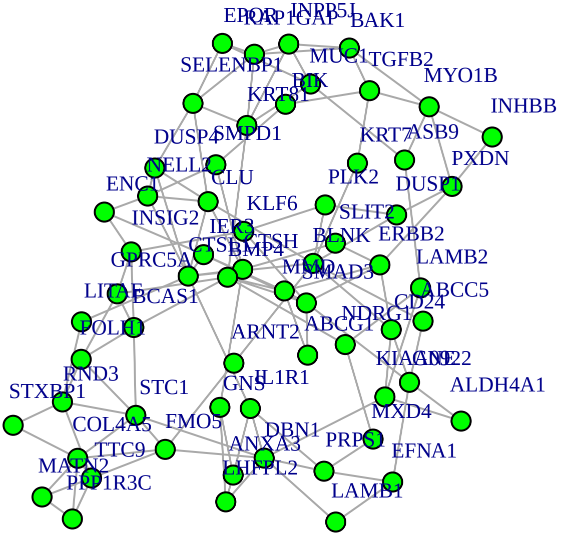

MST2 of the coexpression network for NEG ALL

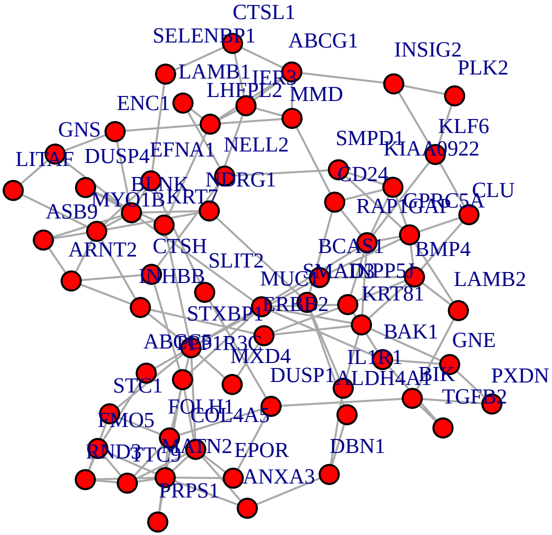

Pathway: TENEDINI_MEGAKARYOCYTE_MARKERS

There are 50 genes in this pathway. This pathway was detected by GSCA

BCR/ABL ALL
Major Gene (BCR/ABL): GATA1
Weight Factor: 1.335
Major Gene (NEG): GP9
Weight Factor: 1.239

NEG ALL
Major Gene (NEG): GP9
Weight Factor: 1.528
Major Gene (BCR/ABL): GATA1
Weight Factor: 0.996

MST2 of the coexpression network for BCR/ABL ALL

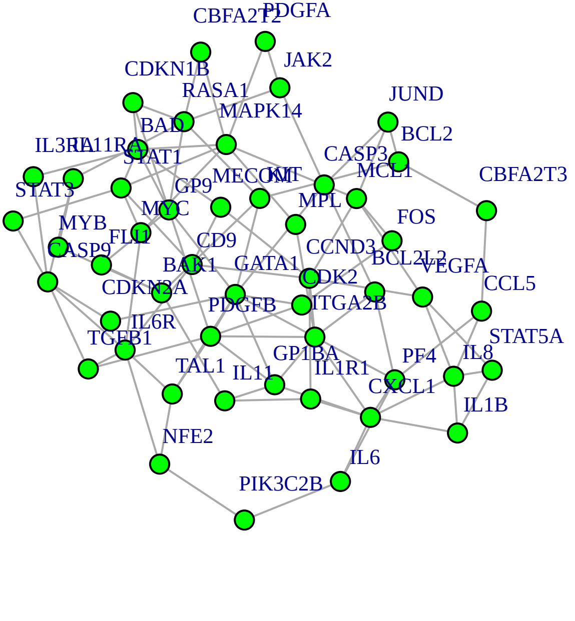

MST2 of the coexpression network for NEG ALL

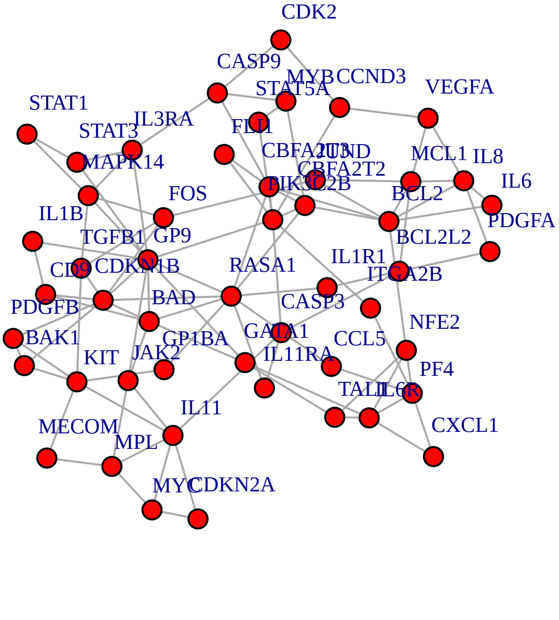

Pathway: ROSS_AML_WITH_MLL_FUSIONS

There are 60 genes in this pathway. This pathway was detected by GSCA

BCR/ABL ALL
Major Gene (BCR/ABL): PLD3
Weight Factor: 1.45
Major Gene (NEG): COL9A2
Weight Factor: 1.219

NEG ALL
Major Gene (NEG): COL9A2
Weight Factor: 1.469
Major Gene (BCR/ABL): PLD3
Weight Factor: 1.187

MST2 of the coexpression network for BCR/ABL ALL

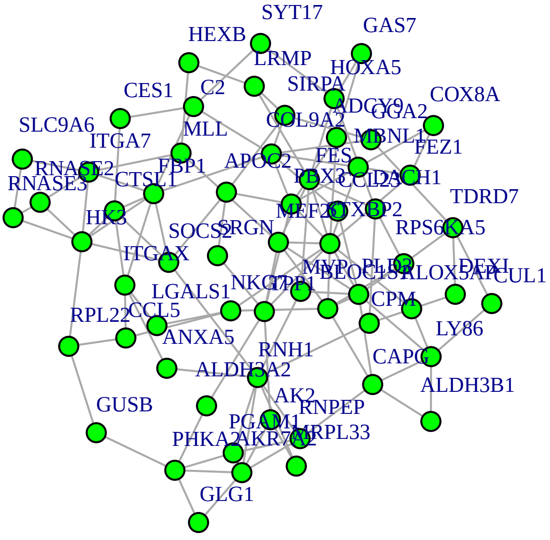

MST2 of the coexpression network for NEG ALL

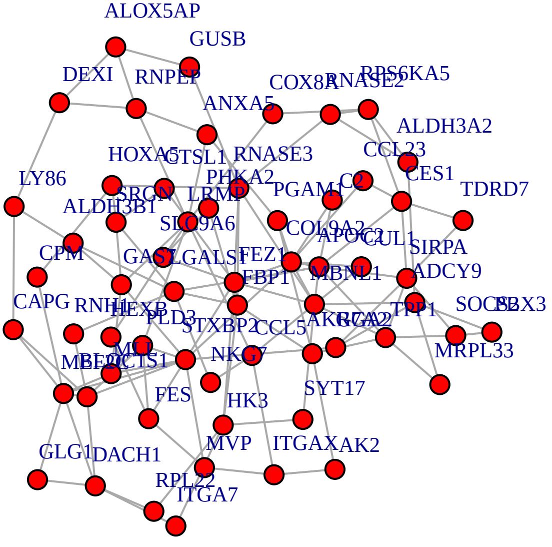

Pathway: CHEOK_RESPONSE_TO_HD_MTX_UP

There are 21 genes in this pathway. This pathway was detected by GSCA

BCR/ABL ALL
Major Gene (BCR/ABL): S100A9
Weight Factor: 1.319
Major Gene (NEG): BAX
Weight Factor: 1.036

NEG ALL
Major Gene (NEG): BAX
Weight Factor: 1.347
Major Gene (BCR/ABL): S100A9
Weight Factor: 1.147

MST2 of the coexpression network for BCR/ABL ALL

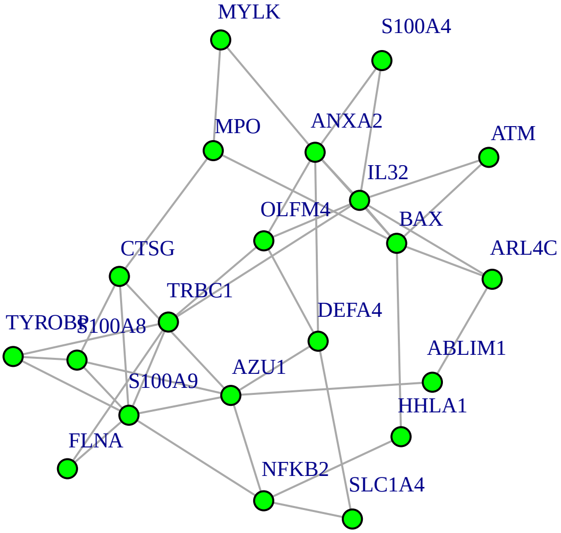

MST2 of the coexpression network for NEG ALL

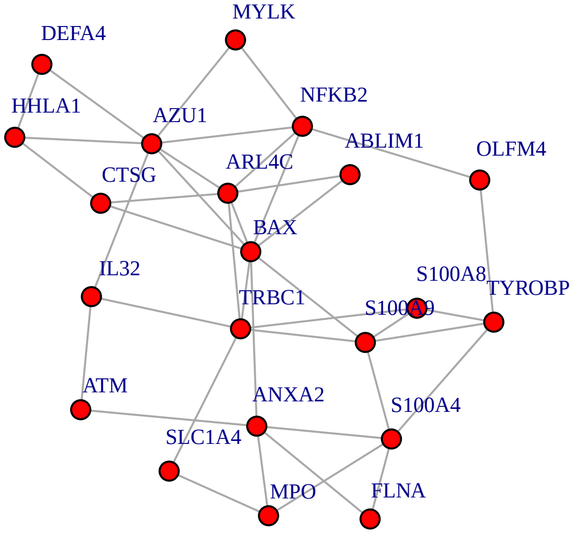

Pathway: WONG_IFNA2_RESISTANCE_DN

There are 15 genes in this pathway. This pathway was detected by GSCA

BCR/ABL ALL
Major Gene (BCR/ABL): LZTR1
Weight Factor: 1.304
Major Gene (NEG): TTR
Weight Factor: 0.873

NEG ALL
Major Gene (NEG): TTR
Weight Factor: 1.307
Major Gene (BCR/ABL): LZTR1
Weight Factor: 1.178

MST2 of the coexpression network for BCR/ABL ALL

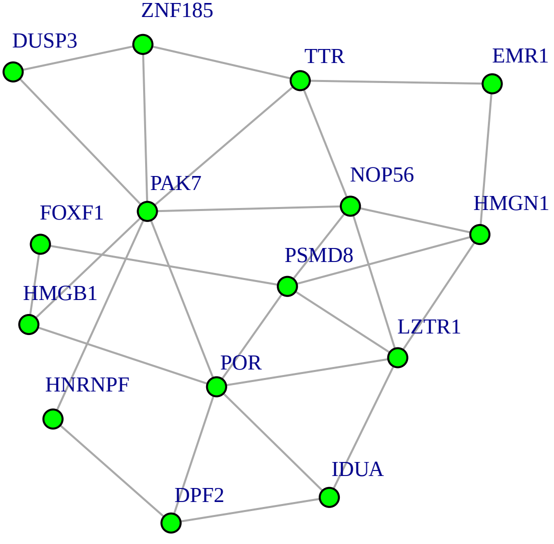

MST2 of the coexpression network for NEG ALL

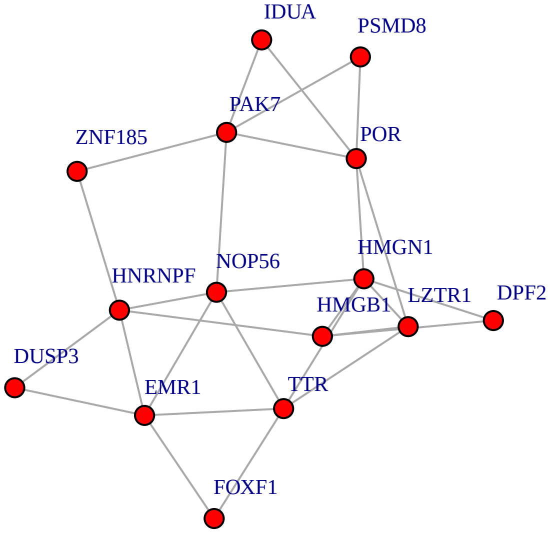

Pathway: ROSS_AML_WITH_AML1_ETO_FUSION

There are 53 genes in this pathway. This pathway was detected by GSCA

BCR/ABL ALL
Major Gene (BCR/ABL): HYAL2
Weight Factor: 1.451
Major Gene (NEG): ARHGAP1
Weight Factor: 1.416

NEG ALL
Major Gene (NEG): ARHGAP1
Weight Factor: 1.495
Major Gene (BCR/ABL): HYAL2
Weight Factor: 1.421

MST2 of the coexpression network for BCR/ABL ALL

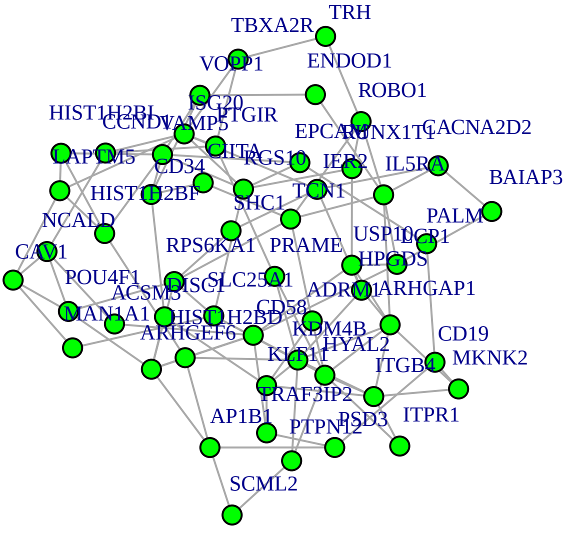

MST2 of the coexpression network for NEG ALL

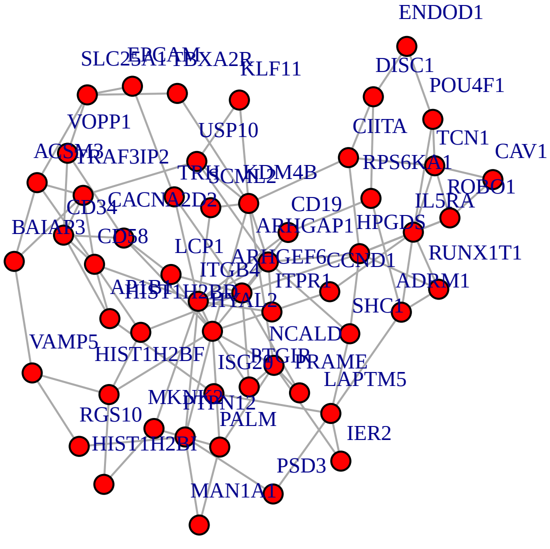

Pathway: HASLINGER_B_CLL_WITH_11Q23_DELETION

There are 19 genes in this pathway. This pathway was detected by GSCA

BCR/ABL ALL
Major Gene (BCR/ABL): UBE4A
Weight Factor: 1.257
Major Gene (NEG): BIRC2
Weight Factor: 1.217

NEG ALL
Major Gene (NEG): BIRC2
Weight Factor: 1.364
Major Gene (BCR/ABL): UBE4A
Weight Factor: 1.308

MST2 of the coexpression network for BCR/ABL ALL

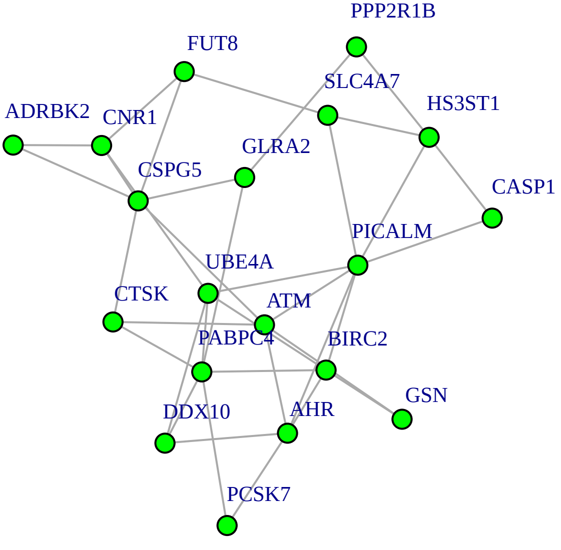

MST2 of the coexpression network for NEG ALL

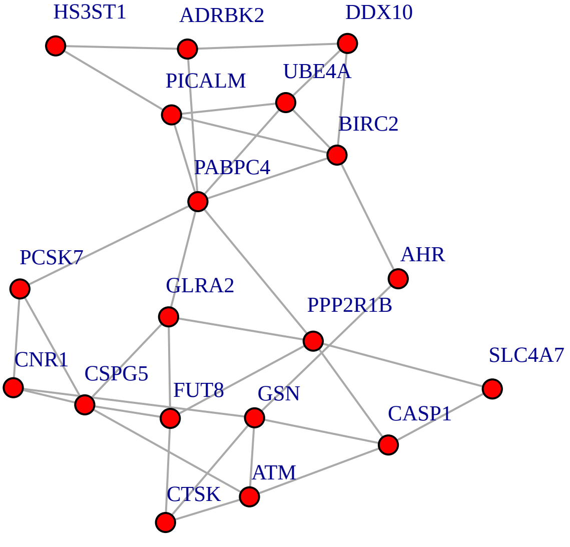

Pathway: HESS_TARGETS_OF_HOXA9_AND_MEIS1_DN

There are 66 genes in this pathway. This pathway was detected by GSCA

BCR/ABL ALL
Major Gene (BCR/ABL): FGR
Weight Factor: 1.366
Major Gene (NEG): PHLDA1
Weight Factor: 1.08

NEG ALL
Major Gene (NEG): PHLDA1
Weight Factor: 1.629
Major Gene (BCR/ABL): FGR
Weight Factor: 1.109

MST2 of the coexpression network for BCR/ABL ALL

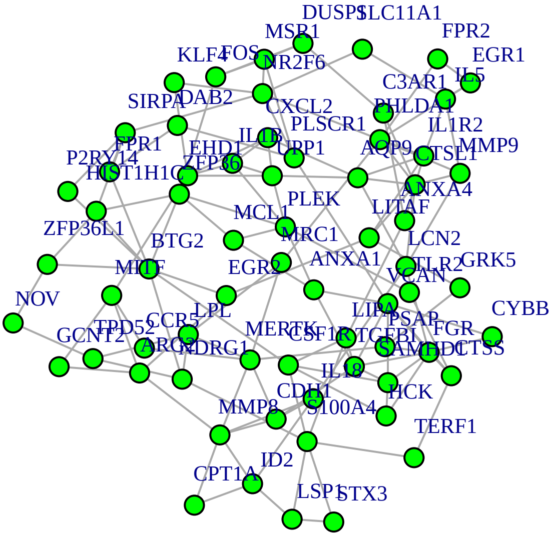

MST2 of the coexpression network for NEG ALL

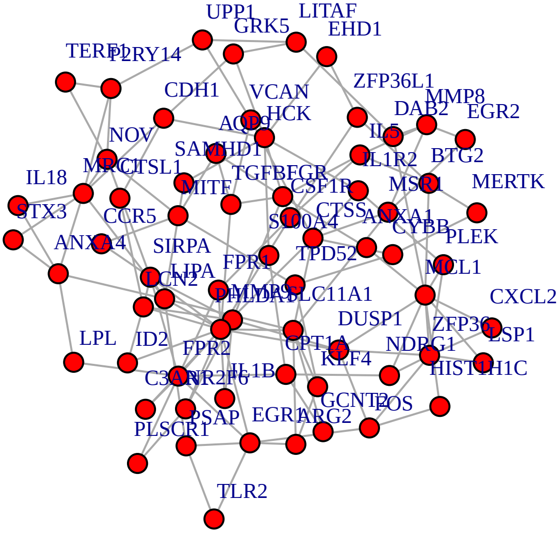

Pathway: FERRANDO_T_ALL_WITH_MLL_ENL_FUSION_DN

There are 74 genes in this pathway. This pathway was detected by GSCA

BCR/ABL ALL
Major Gene (BCR/ABL): MCM2
Weight Factor: 1.465
Major Gene (NEG): HPRT1
Weight Factor: 1.354

NEG ALL
Major Gene (NEG): HPRT1
Weight Factor: 1.477
Major Gene (BCR/ABL): MCM2
Weight Factor: 1.14

MST2 of the coexpression network for BCR/ABL ALL

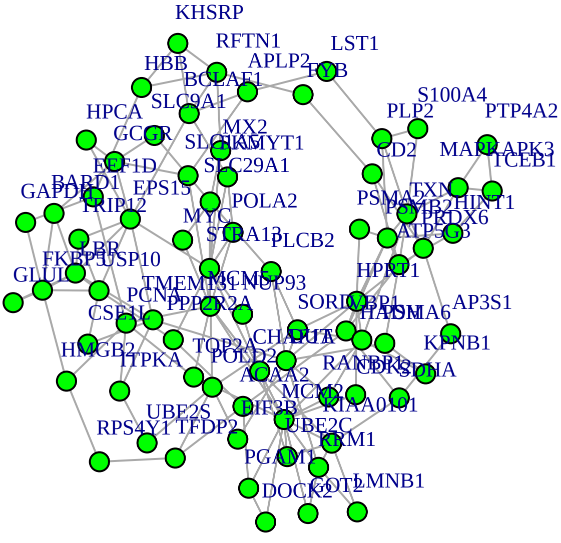

MST2 of the coexpression network for NEG ALL

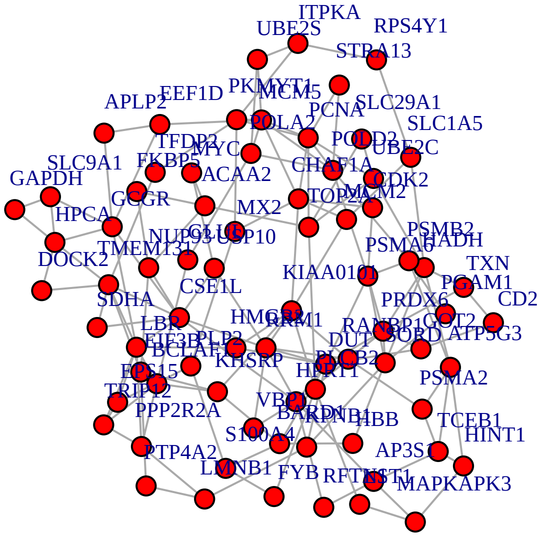

Pathway: HADDAD_B_LYMPHOCYTE_PROGENITOR

There are 214 genes in this pathway. This pathway was detected by GSCA

BCR/ABL ALL
Major Gene (BCR/ABL): ZMYM2
Weight Factor: 1.432
Major Gene (NEG): KIAA1033
Weight Factor: 1.355

NEG ALL
Major Gene (NEG): KIAA1033
Weight Factor: 1.591
Major Gene (BCR/ABL): ZMYM2
Weight Factor: 1.339

MST2 of the coexpression network for BCR/ABL ALL

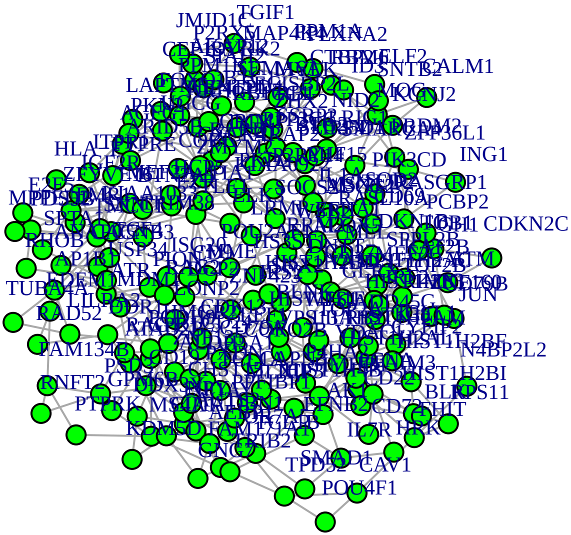

MST2 of the coexpression network for NEG ALL

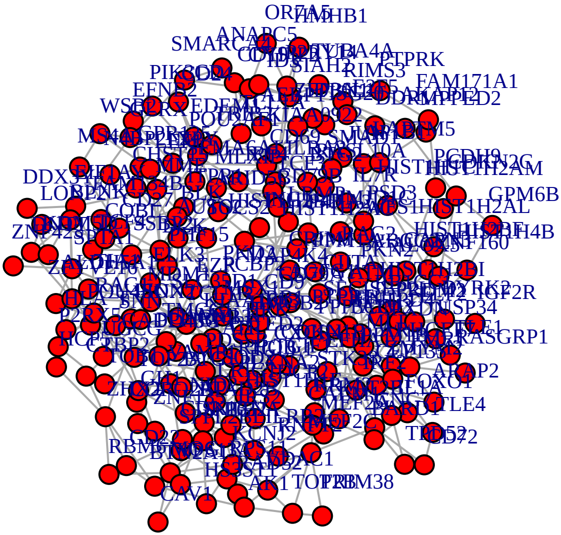

Pathway: VERHAAK_AML_WITH_NPM1_MUTATED_UP

There are 144 genes in this pathway. This pathway was detected by GSCA

BCR/ABL ALL
Major Gene (BCR/ABL): CD14
Weight Factor: 1.5
Major Gene (NEG): PHLDA1
Weight Factor: 1.063

NEG ALL
Major Gene (NEG): PHLDA1
Weight Factor: 1.54
Major Gene (BCR/ABL): CD14
Weight Factor: 1.223

MST2 of the coexpression network for BCR/ABL ALL

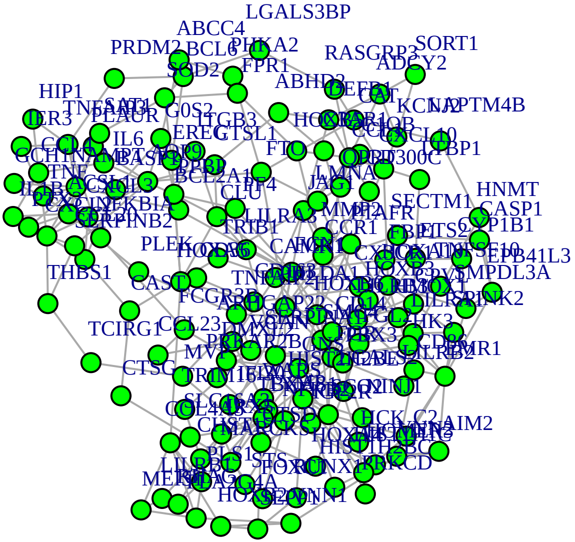

MST2 of the coexpression network for NEG ALL

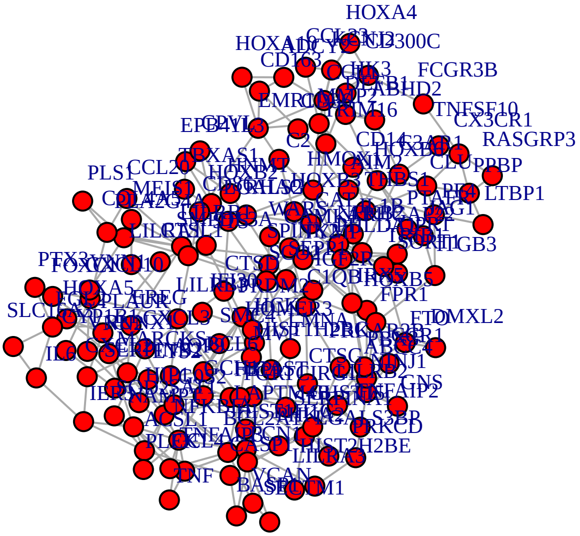

Pathway: FAELT_B_CLL_WITH_VH_REARRANGEMENTS_DN

There are 46 genes in this pathway. This pathway was detected by GSCA

BCR/ABL ALL
Major Gene (BCR/ABL): UBE2D2
Weight Factor: 1.288
Major Gene (NEG): XPO1
Weight Factor: 0.929

NEG ALL
Major Gene (NEG): XPO1
Weight Factor: 1.428
Major Gene (BCR/ABL): UBE2D2
Weight Factor: 1.335

MST2 of the coexpression network for BCR/ABL ALL

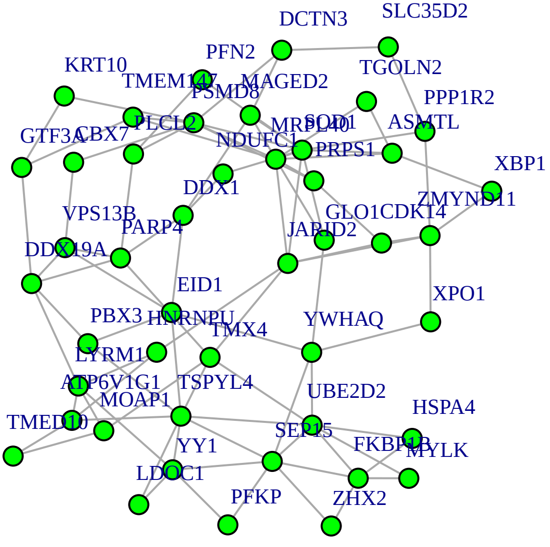

MST2 of the coexpression network for NEG ALL

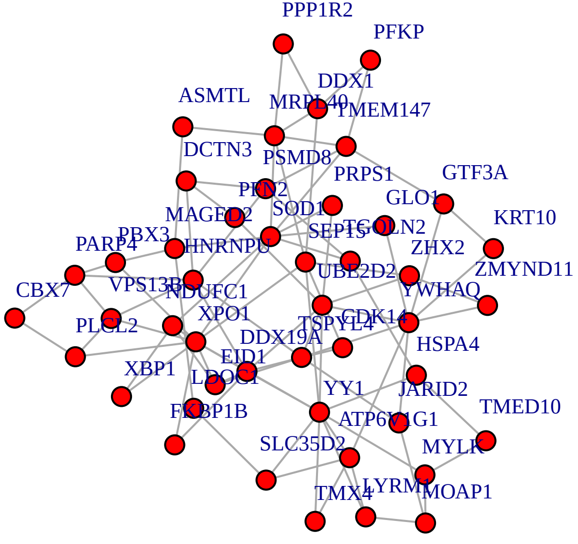

Pathway: CHIARETTI_ACUTE_LYMPHOBLASTIC_LEUKEMIA_ZAP70

There are 65 genes in this pathway. This pathway was detected by GSCA

BCR/ABL ALL
Major Gene (BCR/ABL): SFI1
Weight Factor: 1.486
Major Gene (NEG): GRIN1
Weight Factor: 1.207

NEG ALL
Major Gene (NEG): GRIN1
Weight Factor: 1.337
Major Gene (BCR/ABL): SFI1
Weight Factor: 1.279

MST2 of the coexpression network for BCR/ABL ALL

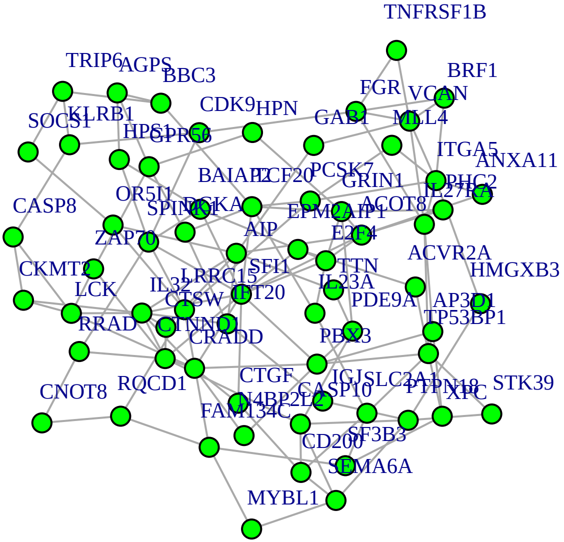

MST2 of the coexpression network for NEG ALL

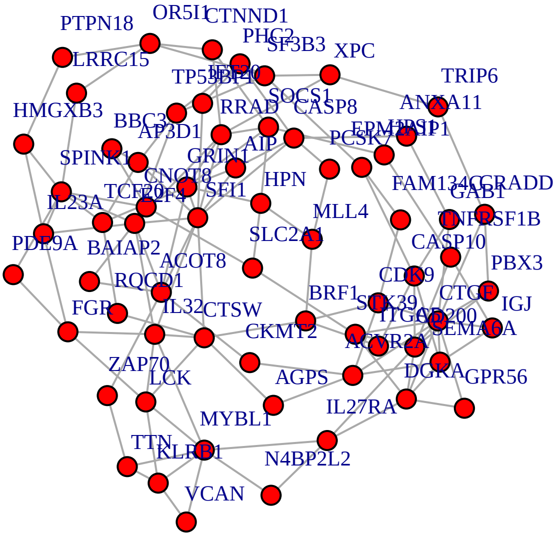

Pathway: BENNETT_SYSTEMIC_LUPUS_ERYTHEMATOSUS

There are 23 genes in this pathway. This pathway was detected by GSCA

BCR/ABL ALL
Major Gene (BCR/ABL): ISG15
Weight Factor: 1.366
Major Gene (NEG): ISG15
Weight Factor: 1.366

NEG ALL
Major Gene (NEG): ISG15
Weight Factor: 1.275
Major Gene (BCR/ABL): ISG15
Weight Factor: 1.275

MST2 of the coexpression network for BCR/ABL ALL

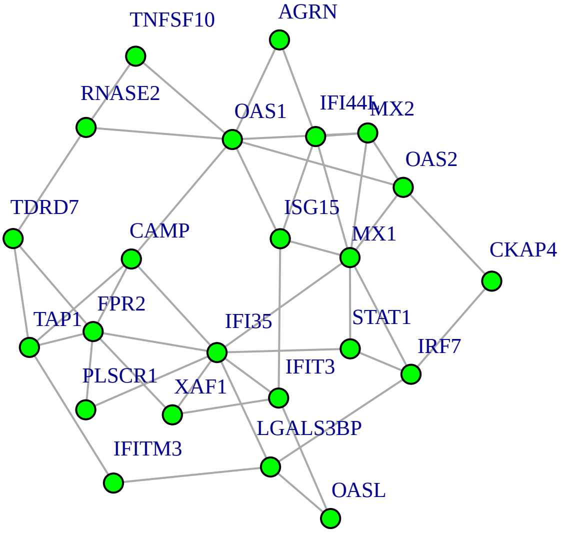

MST2 of the coexpression network for NEG ALL

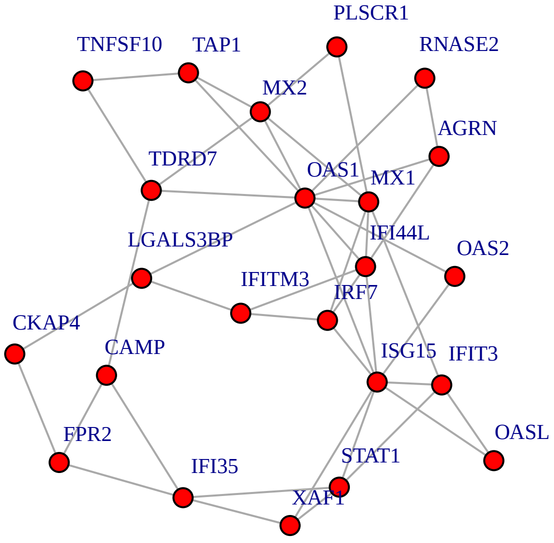

Pathway: HADDAD_T_LYMPHOCYTE_AND_NK_PROGENITOR_DN

There are 56 genes in this pathway. This pathway was detected by GSCA

BCR/ABL ALL
Major Gene (BCR/ABL): TYROBP
Weight Factor: 1.54
Major Gene (NEG): CFD
Weight Factor: 1.341

NEG ALL
Major Gene (NEG): CFD
Weight Factor: 1.53
Major Gene (BCR/ABL): TYROBP
Weight Factor: 1.149

MST2 of the coexpression network for BCR/ABL ALL

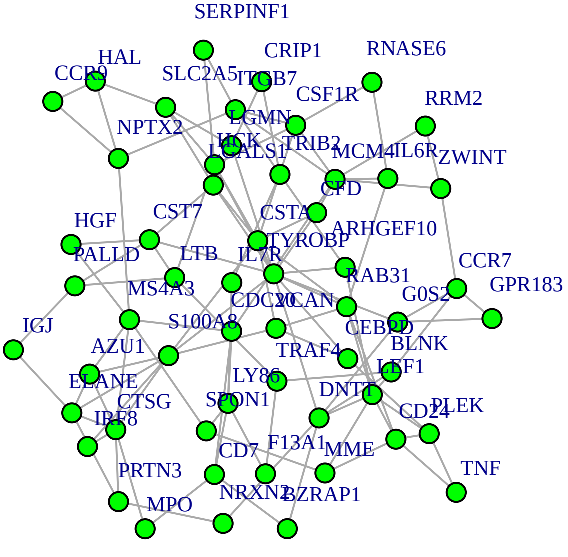

MST2 of the coexpression network for NEG ALL

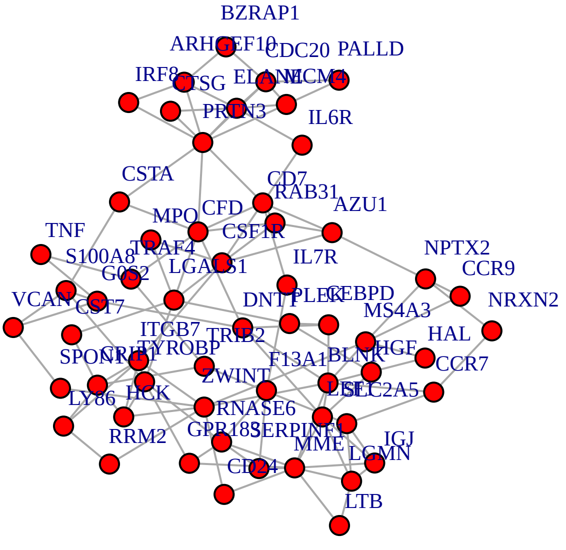

Pathway: RADMACHER_AML_PROGNOSIS

There are 66 genes in this pathway. This pathway was detected by GSCA

BCR/ABL ALL
Major Gene (BCR/ABL): IFI27
Weight Factor: 1.572
Major Gene (NEG): SLC6A8
Weight Factor: 1.451

NEG ALL
Major Gene (NEG): SLC6A8
Weight Factor: 1.472
Major Gene (BCR/ABL): IFI27
Weight Factor: 0.81

MST2 of the coexpression network for BCR/ABL ALL

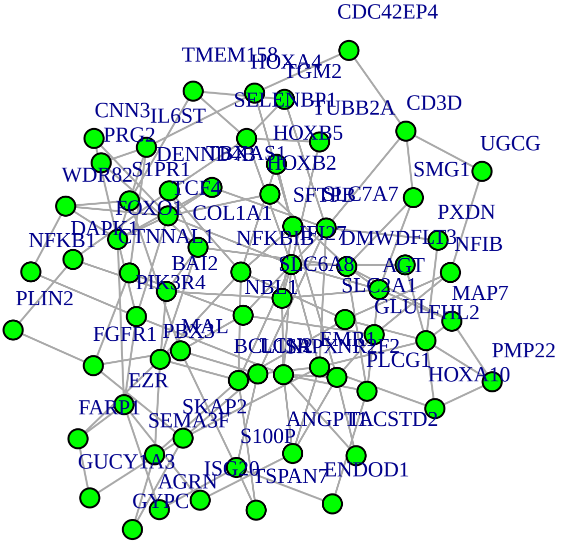

MST2 of the coexpression network for NEG ALL

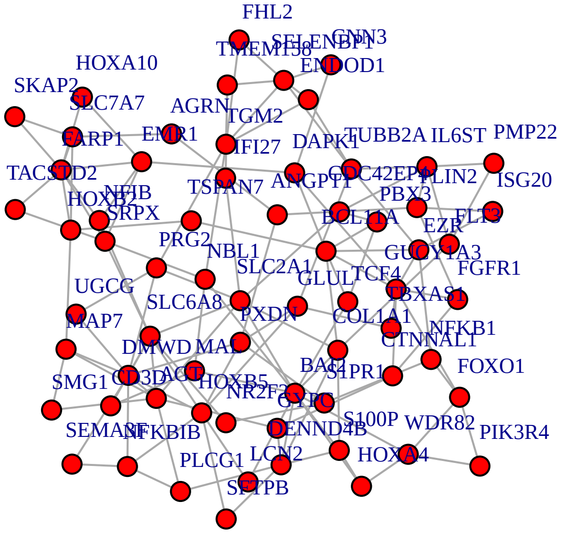

Pathway: VERHAAK_AML_WITH_NPM1_MUTATED_DN

There are 206 genes in this pathway. This pathway was detected by GSCA

BCR/ABL ALL
Major Gene (BCR/ABL): TFPI
Weight Factor: 1.359
Major Gene (NEG): PRODH
Weight Factor: 1.286

NEG ALL
Major Gene (NEG): PRODH
Weight Factor: 1.55
Major Gene (BCR/ABL): TFPI
Weight Factor: 0.725

MST2 of the coexpression network for BCR/ABL ALL

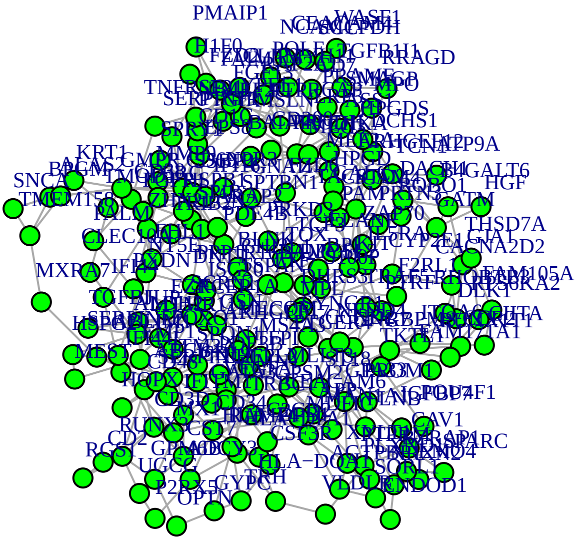

MST2 of the coexpression network for NEG ALL

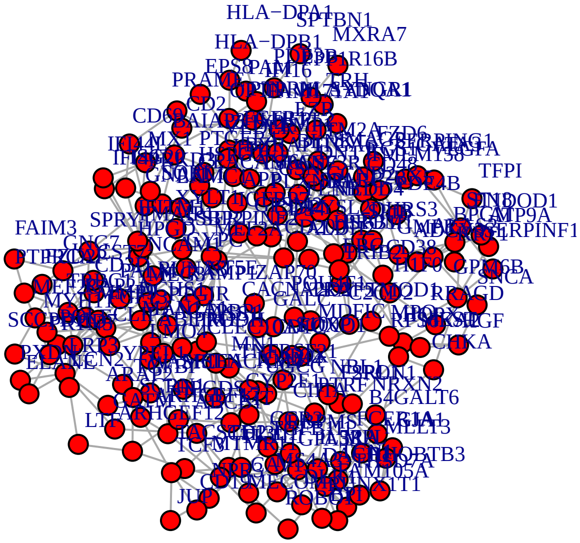

Pathway: MUNSHI_MULTIPLE_MYELOMA_UP

There are 54 genes in this pathway. This pathway was detected by GSCA

BCR/ABL ALL
Major Gene (BCR/ABL): DAD1
Weight Factor: 1.418
Major Gene (NEG): PSMB1
Weight Factor: 0.814

NEG ALL
Major Gene (NEG): PSMB1
Weight Factor: 1.376
Major Gene (BCR/ABL): DAD1
Weight Factor: 1.095

MST2 of the coexpression network for BCR/ABL ALL

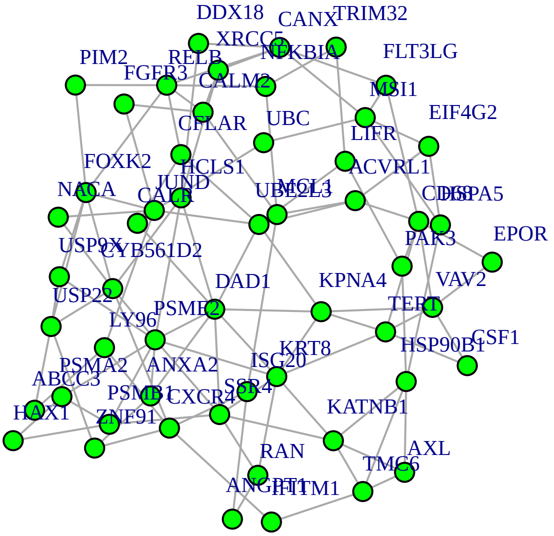

MST2 of the coexpression network for NEG ALL

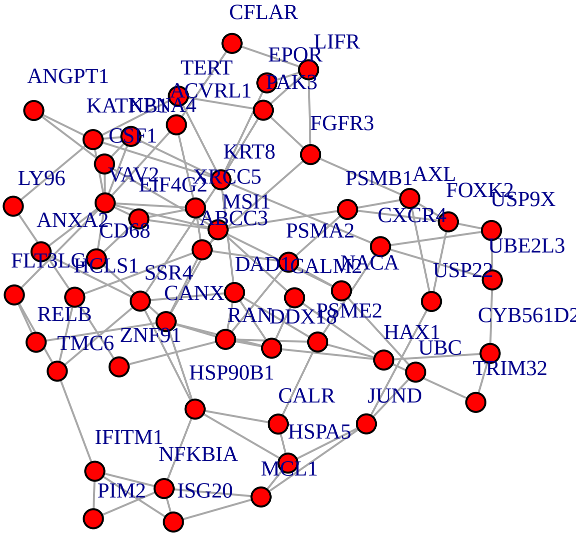

Pathway: FAELT_B_CLL_WITH_VH3_21_DN

There are 48 genes in this pathway. This pathway was detected by GSCA

BCR/ABL ALL
Major Gene (BCR/ABL): SPEN
Weight Factor: 1.484
Major Gene (NEG): MICB
Weight Factor: 1.085

NEG ALL
Major Gene (NEG): MICB
Weight Factor: 1.461
Major Gene (BCR/ABL): SPEN
Weight Factor: 1.332

MST2 of the coexpression network for BCR/ABL ALL

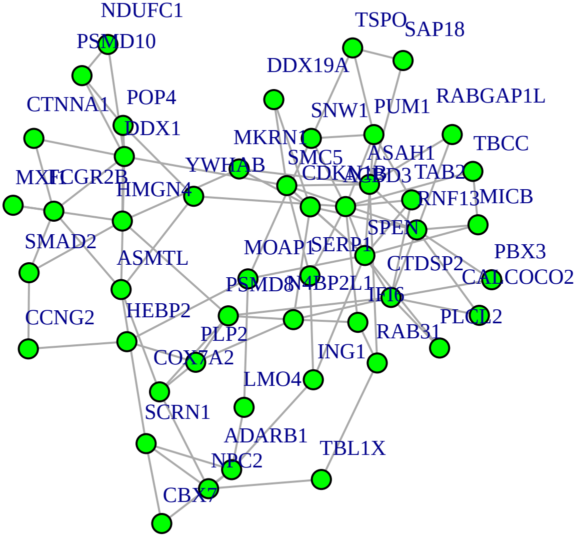

MST2 of the coexpression network for NEG ALL

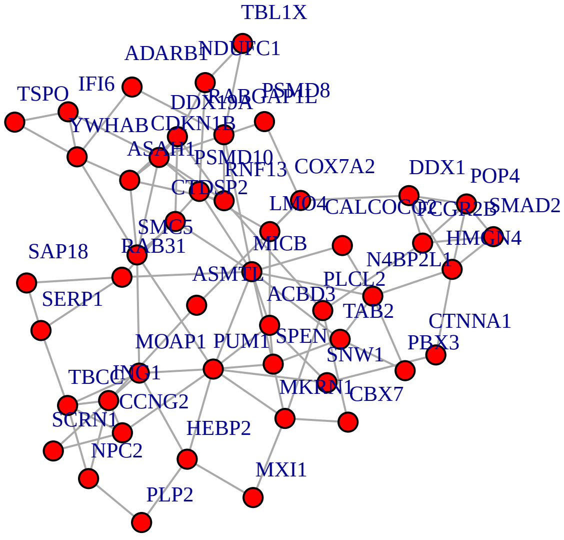

Pathway: GEISS_RESPONSE_TO_DSRNA_UP

There are 30 genes in this pathway. This pathway was detected by GSCA

BCR/ABL ALL
Major Gene (BCR/ABL): TNFAIP6
Weight Factor: 1.378
Major Gene (NEG): BIRC2
Weight Factor: 1.211

NEG ALL
Major Gene (NEG): BIRC2
Weight Factor: 1.374
Major Gene (BCR/ABL): TNFAIP6
Weight Factor: 0.878

MST2 of the coexpression network for BCR/ABL ALL

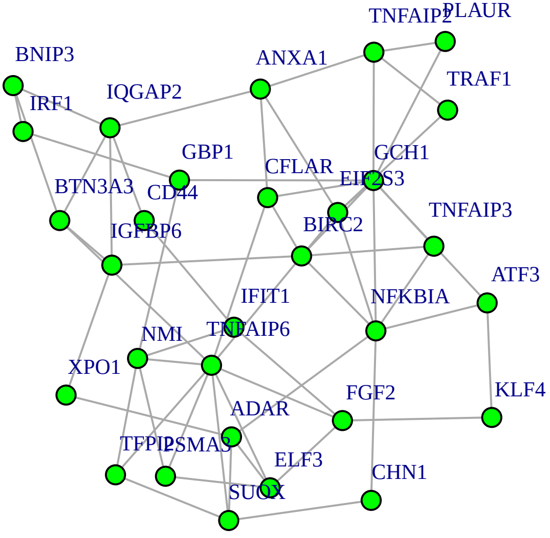

MST2 of the coexpression network for NEG ALL

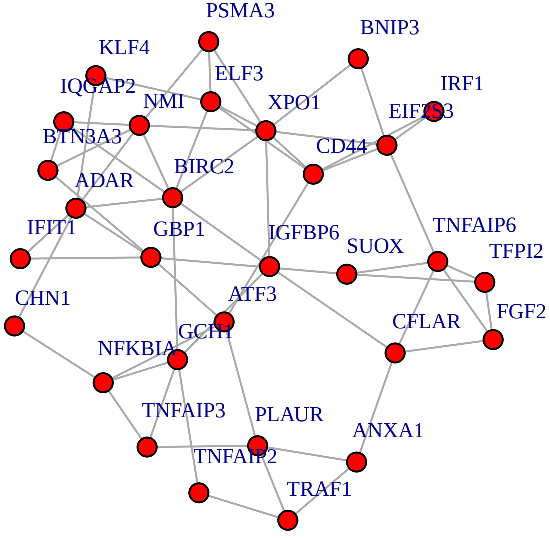

Pathway: CLASPER_LYMPHATIC_VESSELS_DURING_METASTASIS_UP

There are 15 genes in this pathway. This pathway was detected by GSCA

BCR/ABL ALL
Major Gene (BCR/ABL): HOXB5
Weight Factor: 1.246
Major Gene (NEG): TGFB3
Weight Factor: 1.102

NEG ALL
Major Gene (NEG): TGFB3
Weight Factor: 1.394
Major Gene (BCR/ABL): HOXB5
Weight Factor: 1.28

MST2 of the coexpression network for BCR/ABL ALL

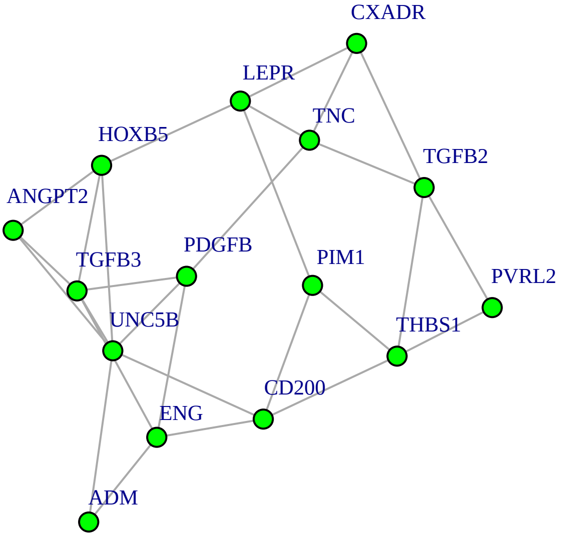

MST2 of the coexpression network for NEG ALL

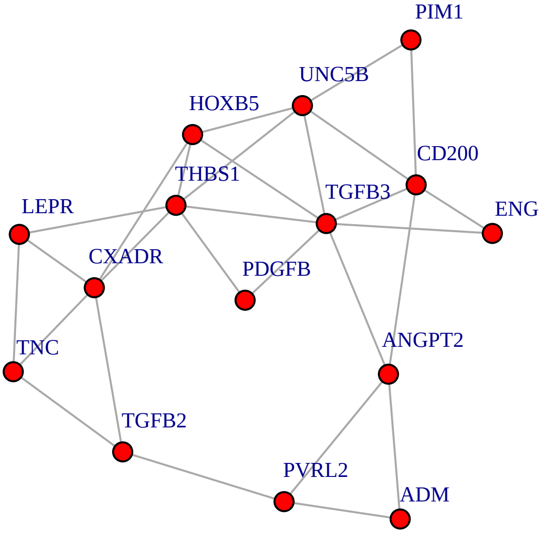

Pathway: KYNG_ENVIRONMENTAL_STRESS_RESPONSE_UP

There are 28 genes in this pathway. This pathway was detected by GSCA

BCR/ABL ALL
Major Gene (BCR/ABL): FGFR4
Weight Factor: 1.381
Major Gene (NEG): FGFR4
Weight Factor: 1.381

NEG ALL
Major Gene (NEG): FGFR4
Weight Factor: 1.505
Major Gene (BCR/ABL): FGFR4
Weight Factor: 1.505

MST2 of the coexpression network for BCR/ABL ALL

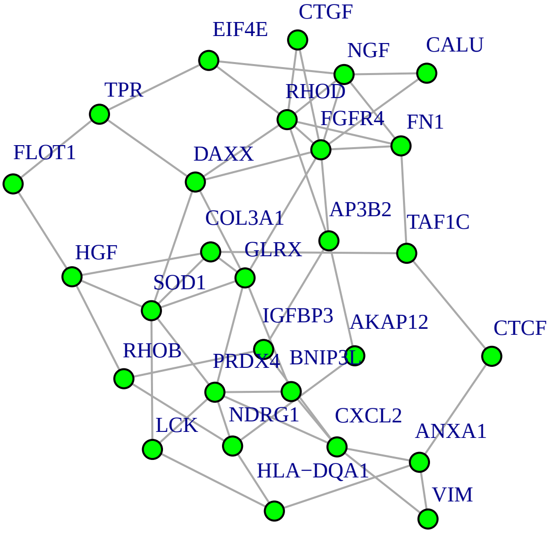

MST2 of the coexpression network for NEG ALL

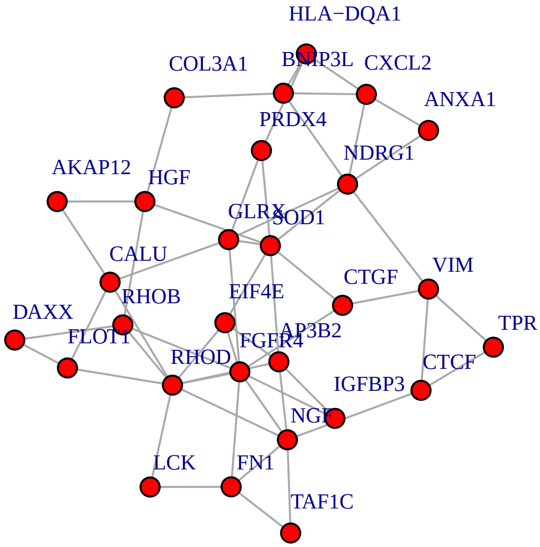

Pathway: COATES_MACROPHAGE_M1_VS_M2_DN

There are 44 genes in this pathway. This pathway was detected by GSCA

BCR/ABL ALL
Major Gene (BCR/ABL): F7
Weight Factor: 1.563
Major Gene (NEG): LBP
Weight Factor: 1.394

NEG ALL
Major Gene (NEG): LBP
Weight Factor: 1.563
Major Gene (BCR/ABL): F7
Weight Factor: 1.545

MST2 of the coexpression network for BCR/ABL ALL

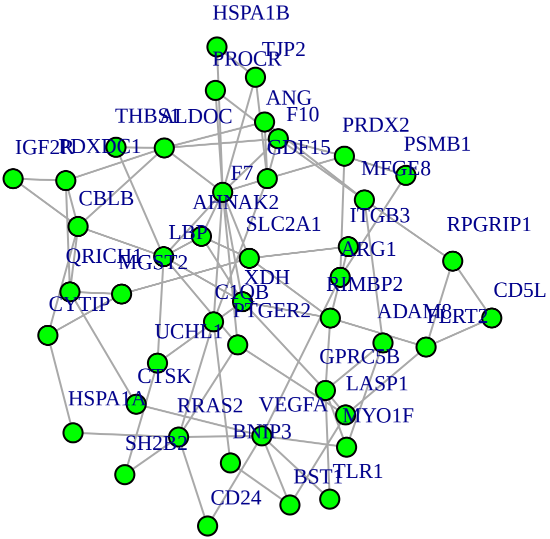

MST2 of the coexpression network for NEG ALL

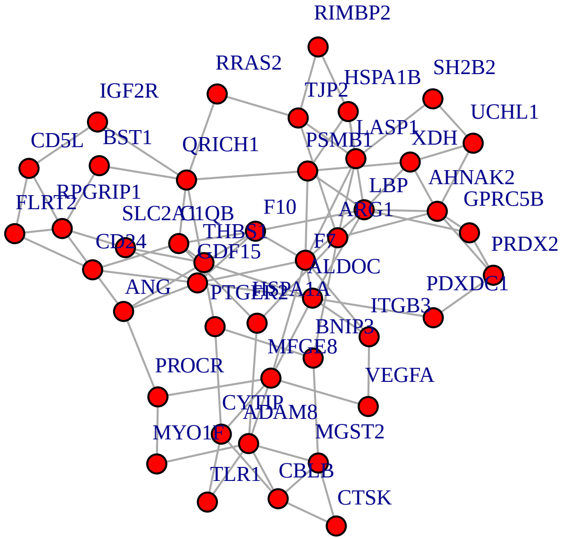

Pathway: WALLACE_PROSTATE_CANCER_RACE_UP

There are 242 genes in this pathway. This pathway was detected by GSCA

BCR/ABL ALL
Major Gene (BCR/ABL): RAC2
Weight Factor: 1.409
Major Gene (NEG): POM121L9P
Weight Factor: 1.242

NEG ALL
Major Gene (NEG): POM121L9P
Weight Factor: 1.45
Major Gene (BCR/ABL): RAC2
Weight Factor: 1.123

MST2 of the coexpression network for BCR/ABL ALL

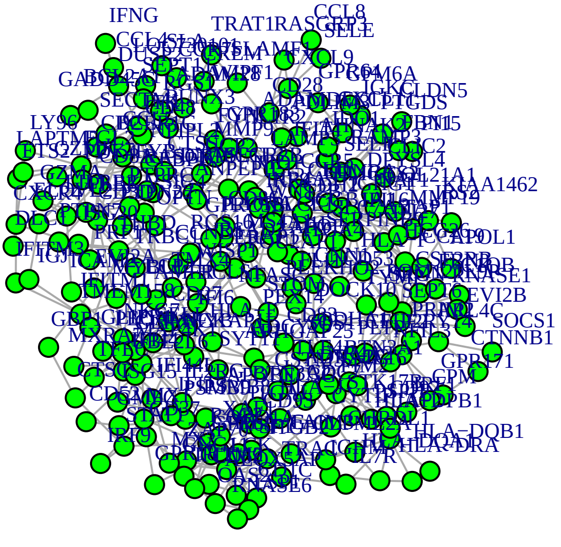

MST2 of the coexpression network for NEG ALL

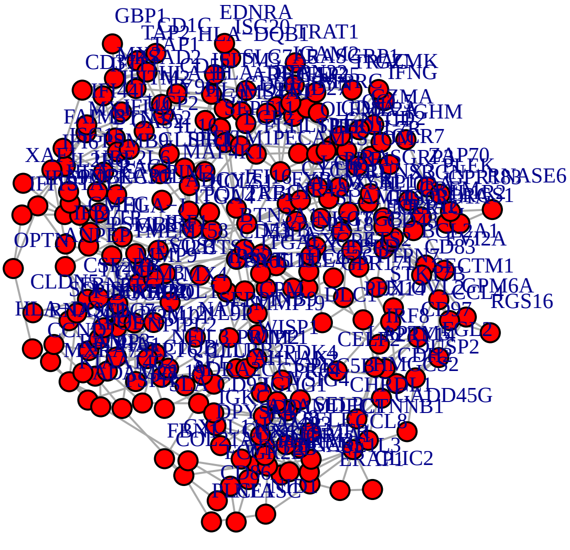

Pathway: OUILLETTE_CLL_13Q14_DELETION_DN

There are 32 genes in this pathway. This pathway was detected by GSCA

BCR/ABL ALL
Major Gene (BCR/ABL): LPIN1
Weight Factor: 1.454
Major Gene (NEG): RNASEH2B
Weight Factor: 0.892

NEG ALL
Major Gene (NEG): RNASEH2B
Weight Factor: 1.424
Major Gene (BCR/ABL): LPIN1
Weight Factor: 1.232

MST2 of the coexpression network for BCR/ABL ALL

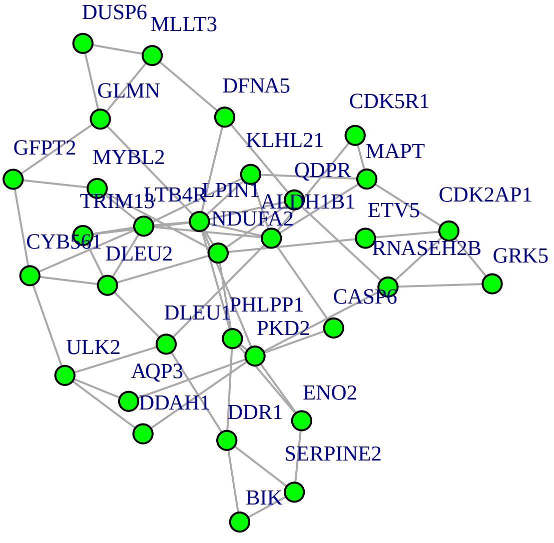

MST2 of the coexpression network for NEG ALL

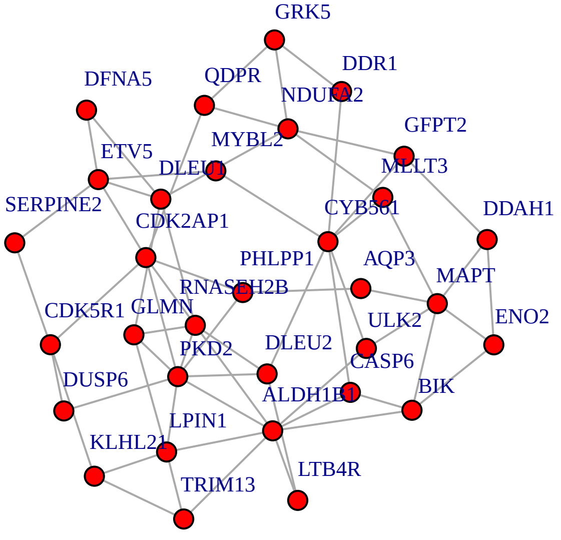

Pathway: FUJII_YBX1_TARGETS_UP

There are 22 genes in this pathway. This pathway was detected by GSCA

BCR/ABL ALL
Major Gene (BCR/ABL): NR5A2
Weight Factor: 1.273
Major Gene (NEG): GPER
Weight Factor: 1.191

NEG ALL
Major Gene (NEG): GPER
Weight Factor: 1.413
Major Gene (BCR/ABL): NR5A2
Weight Factor: 0.852

MST2 of the coexpression network for BCR/ABL ALL

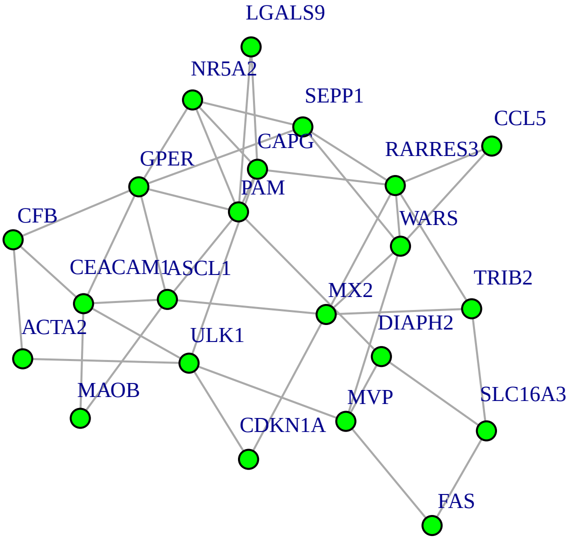

MST2 of the coexpression network for NEG ALL

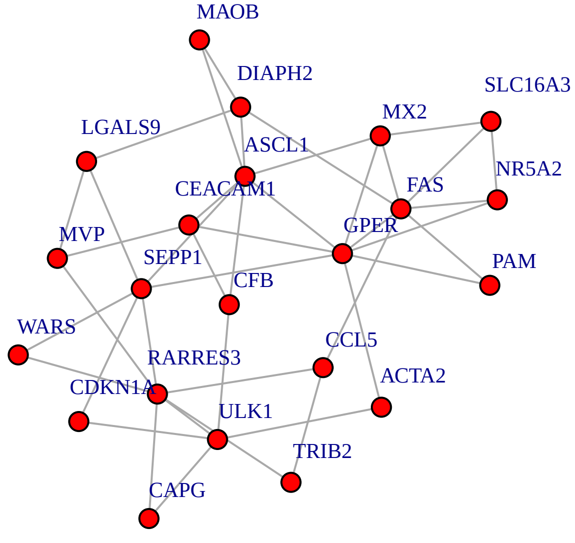

Pathway: HUANG_FOXA2_TARGETS_UP

There are 36 genes in this pathway. This pathway was detected by GSCA

BCR/ABL ALL
Major Gene (BCR/ABL): HPGD
Weight Factor: 1.306
Major Gene (NEG): HPGD
Weight Factor: 1.306

NEG ALL
Major Gene (NEG): HPGD
Weight Factor: 1.399
Major Gene (BCR/ABL): HPGD
Weight Factor: 1.399

MST2 of the coexpression network for BCR/ABL ALL

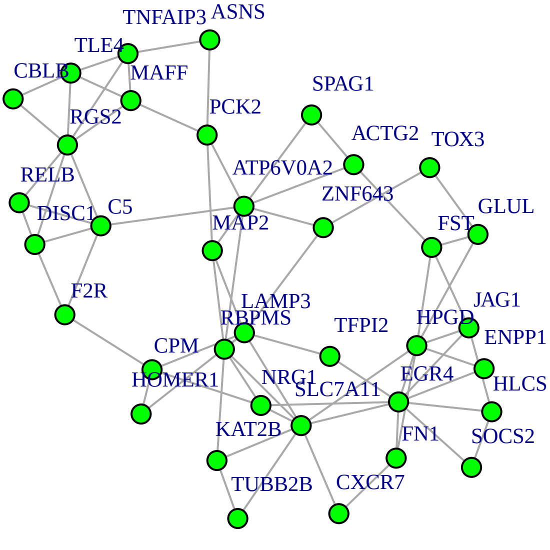

MST2 of the coexpression network for NEG ALL

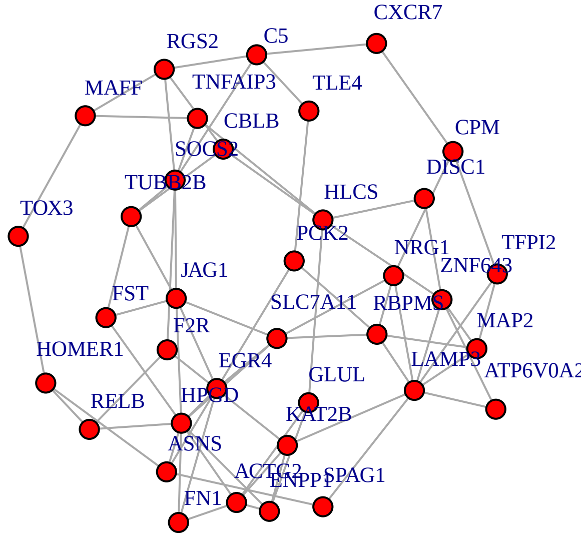

Pathway: SAGIV_CD24_TARGETS_DN

There are 34 genes in this pathway. This pathway was detected by GSCA

BCR/ABL ALL
Major Gene (BCR/ABL): CTTN
Weight Factor: 1.41
Major Gene (NEG): THBS1
Weight Factor: 0.961

MST2 of the coexpression network for BCR/ABL ALL

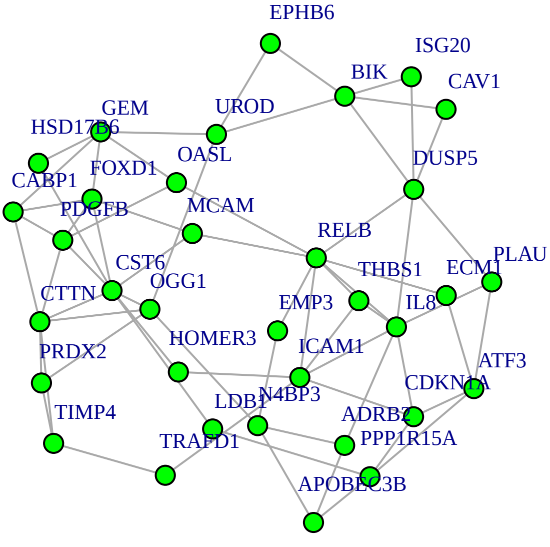

NEG ALL
Major Gene (NEG): THBS1
Weight Factor: 1.334
Major Gene (BCR/ABL): CTTN
Weight Factor: 0.888

MST2 of the coexpression network for NEG ALL

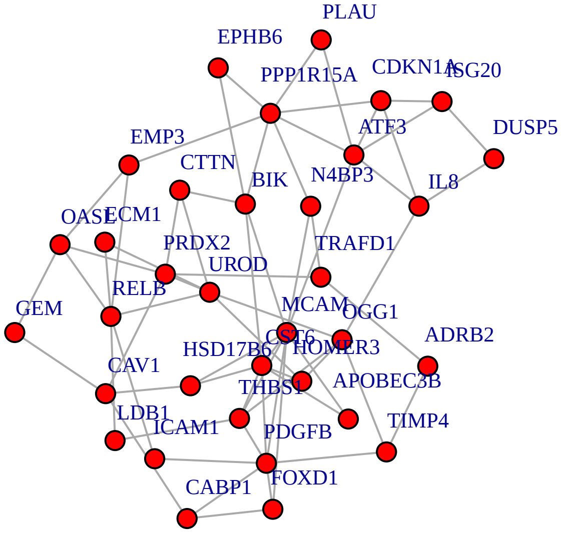

Pathway: MOSERLE_IFNA_RESPONSE

There are 20 genes in this pathway. This pathway was detected by GSCA

BCR/ABL ALL
Major Gene (BCR/ABL): IFI44L
Weight Factor: 1.306
Major Gene (NEG): IFIT1
Weight Factor: 1.236

NEG ALL
Major Gene (NEG): IFIT1
Weight Factor: 1.297
Major Gene (BCR/ABL): IFI44L
Weight Factor: 1.23

MST2 of the coexpression network for BCR/ABL ALL

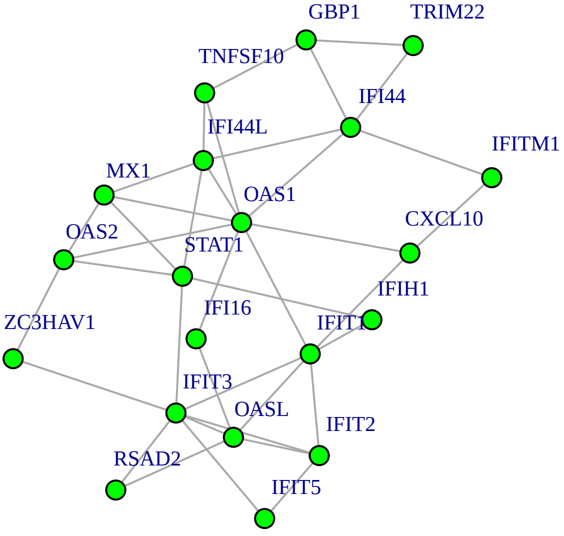

MST2 of the coexpression network for NEG ALL

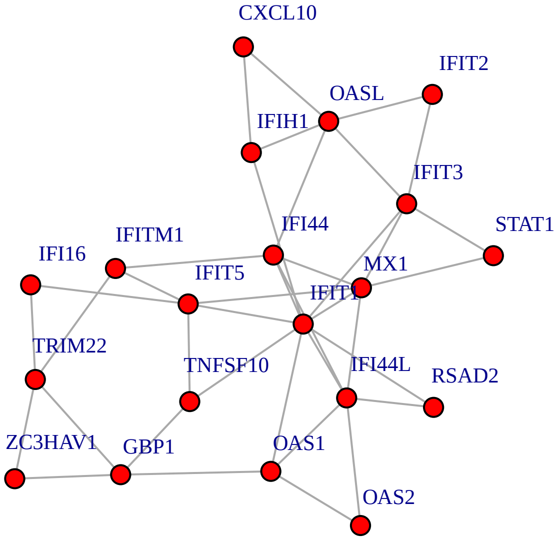

Pathway: BASSO_HAIRY_CELL_LEUKEMIA_DN

There are 80 genes in this pathway. This pathway was detected by GSCA

BCR/ABL ALL
Major Gene (BCR/ABL): SDC3
Weight Factor: 1.275
Major Gene (NEG): GABARAPL2
Weight Factor: 0.649

NEG ALL
Major Gene (NEG): GABARAPL2
Weight Factor: 1.366
Major Gene (BCR/ABL): SDC3
Weight Factor: 1.316

MST2 of the coexpression network for BCR/ABL ALL

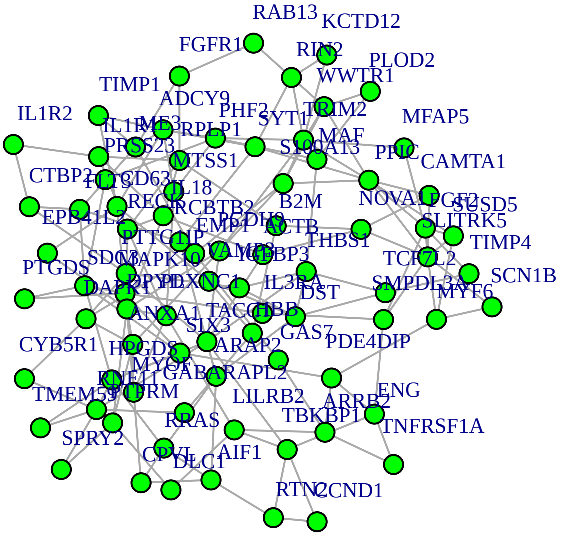

MST2 of the coexpression network for NEG ALL

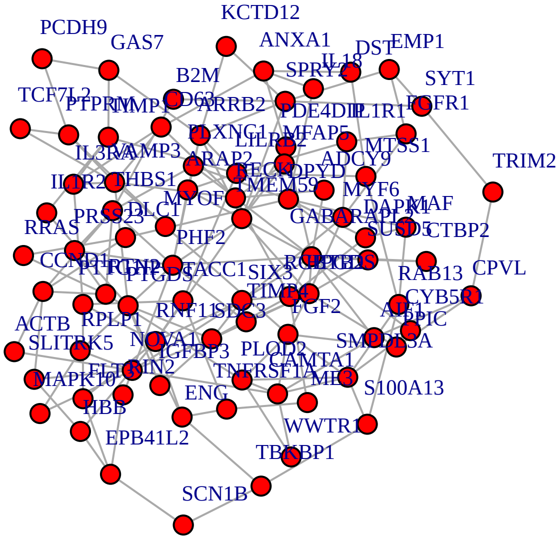

Pathway: BERNARD_PPAPDC1B_TARGETS_UP

There are 17 genes in this pathway. This pathway was detected by GSCA

BCR/ABL ALL
Major Gene (BCR/ABL): SNX13
Weight Factor: 1.321
Major Gene (NEG): WBP4
Weight Factor: 0.979

NEG ALL
Major Gene (NEG): WBP4
Weight Factor: 1.276
Major Gene (BCR/ABL): SNX13
Weight Factor: 1.145

MST2 of the coexpression network for BCR/ABL ALL

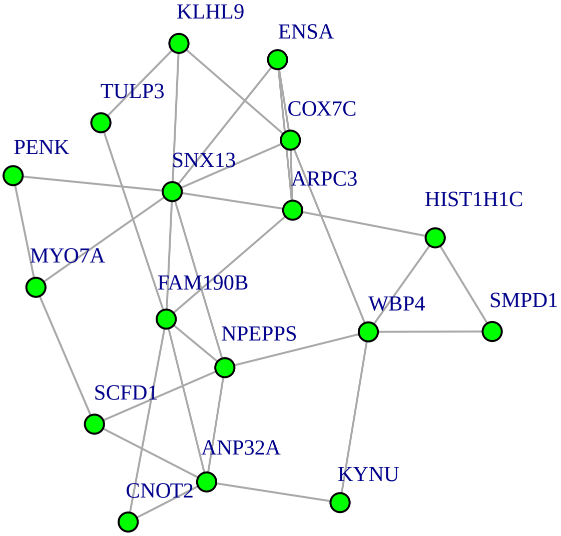

MST2 of the coexpression network for NEG ALL

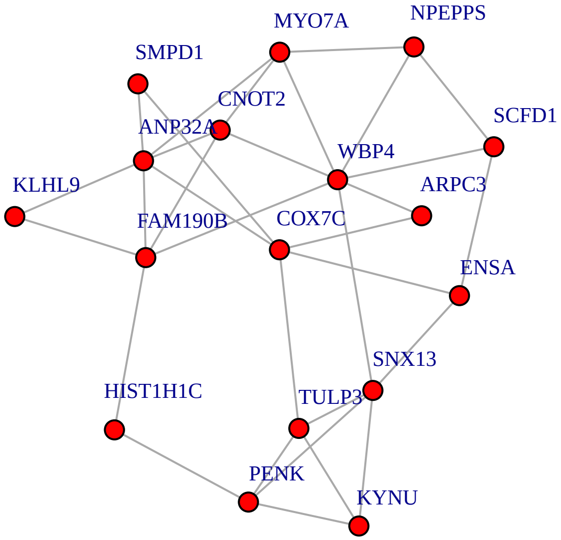

Pathway: BERNARD_PPAPDC1B_TARGETS_DN

There are 33 genes in this pathway. This pathway was detected by GSCA

BCR/ABL ALL
Major Gene (BCR/ABL): ADD3
Weight Factor: 1.357
Major Gene (NEG): RARA
Weight Factor: 1.177

NEG ALL
Major Gene (NEG): RARA
Weight Factor: 1.473
Major Gene (BCR/ABL): ADD3
Weight Factor: 1.035

MST2 of the coexpression network for BCR/ABL ALL

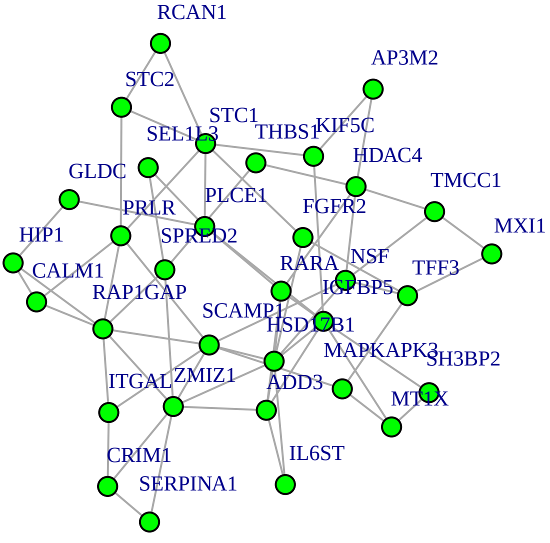

MST2 of the coexpression network for NEG ALL

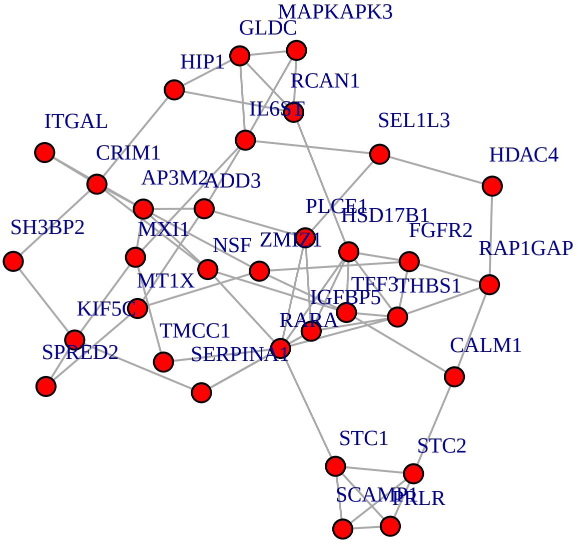

Pathway: BREDEMEYER_RAG_SIGNALING_VIA_ATM_NOT_VIA_NFKB_DN

There are 20 genes in this pathway. This pathway was detected by GSCA

BCR/ABL ALL
Major Gene (BCR/ABL): SLC4A8
Weight Factor: 1.37
Major Gene (NEG): ACADL
Weight Factor: 1.305

NEG ALL
Major Gene (NEG): ACADL
Weight Factor: 1.325
Major Gene (BCR/ABL): SLC4A8
Weight Factor: 1.001

MST2 of the coexpression network for BCR/ABL ALL

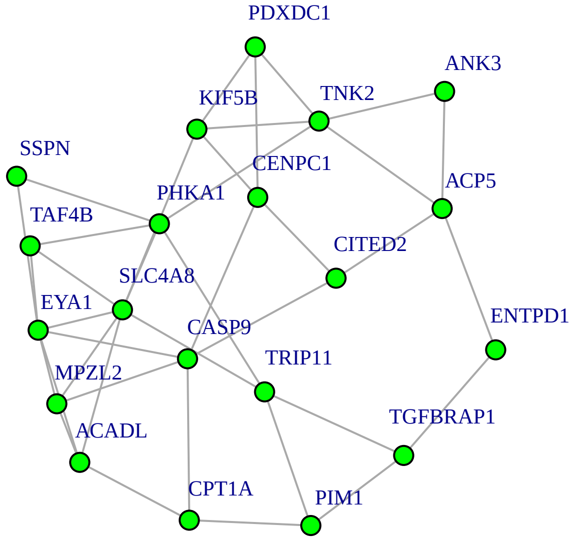

MST2 of the coexpression network for NEG ALL

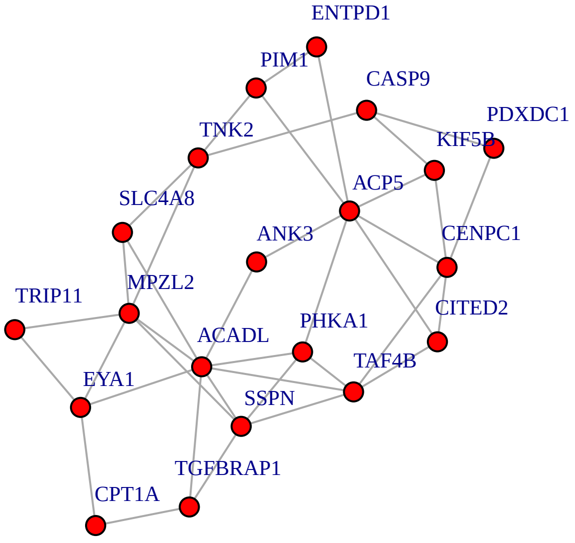

Pathway: HUANG_DASATINIB_RESISTANCE_UP

There are 58 genes in this pathway. This pathway was detected by GSCA

BCR/ABL ALL
Major Gene (BCR/ABL): JAG1
Weight Factor: 1.322
Major Gene (NEG): MET
Weight Factor: 1.233

NEG ALL
Major Gene (NEG): MET
Weight Factor: 1.47
Major Gene (BCR/ABL): JAG1
Weight Factor: 1.219

MST2 of the coexpression network for BCR/ABL ALL

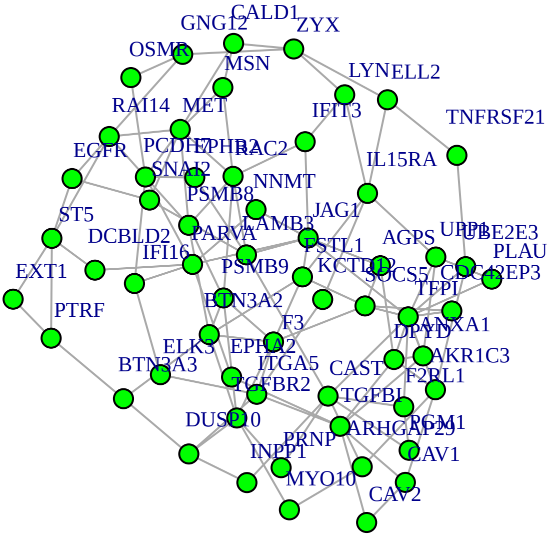

MST2 of the coexpression network for NEG ALL

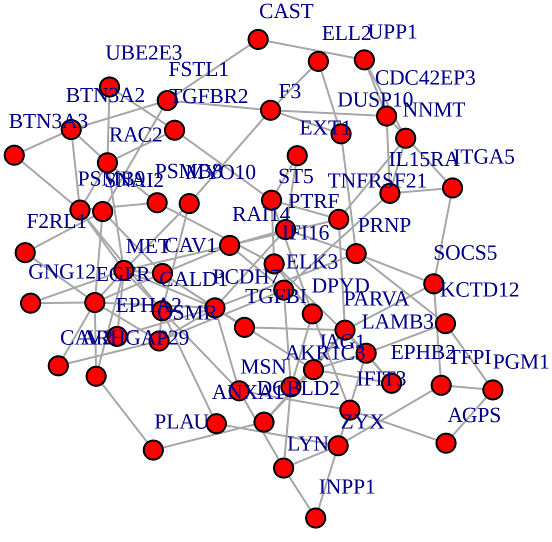

Pathway: TAYLOR_METHYLATED_IN_ACUTE_LYMPHOBLASTIC_LEUKEMIA

There are 38 genes in this pathway. This pathway was detected by GSCA

BCR/ABL ALL
Major Gene (BCR/ABL): PROX1
Weight Factor: 1.372
Major Gene (NEG): FOXD2
Weight Factor: 1.071

NEG ALL
Major Gene (NEG): FOXD2
Weight Factor: 1.439
Major Gene (BCR/ABL): PROX1
Weight Factor: 1.083

MST2 of the coexpression network for BCR/ABL ALL

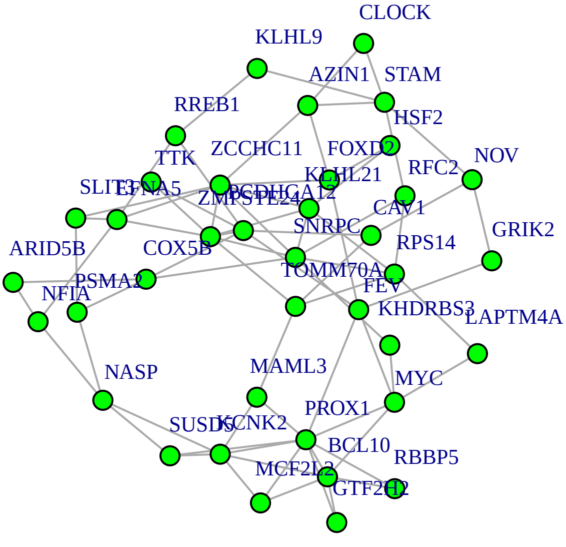

MST2 of the coexpression network for NEG ALL

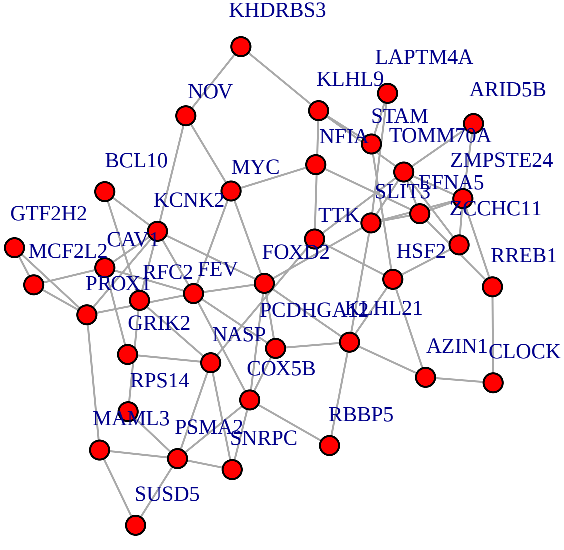

Pathway: ALONSO_METASTASIS_UP

There are 128 genes in this pathway. This pathway was detected by GSCA

BCR/ABL ALL
Major Gene (BCR/ABL): VBP1
Weight Factor: 1.332
Major Gene (NEG): MDH1
Weight Factor: 1.276

NEG ALL
Major Gene (NEG): MDH1
Weight Factor: 1.486
Major Gene (BCR/ABL): VBP1
Weight Factor: 1.445

MST2 of the coexpression network for BCR/ABL ALL

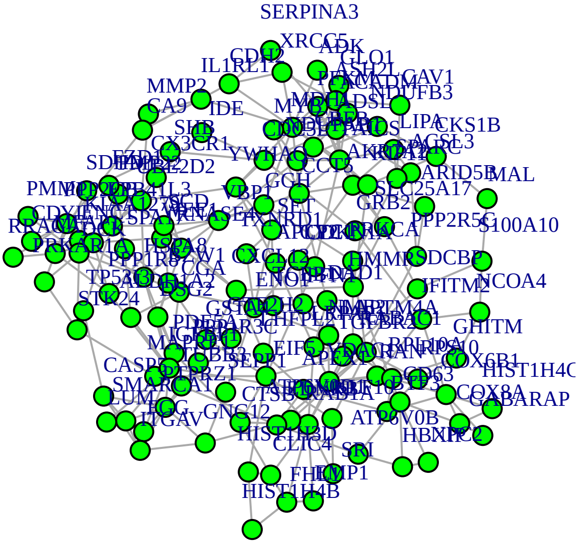

MST2 of the coexpression network for NEG ALL

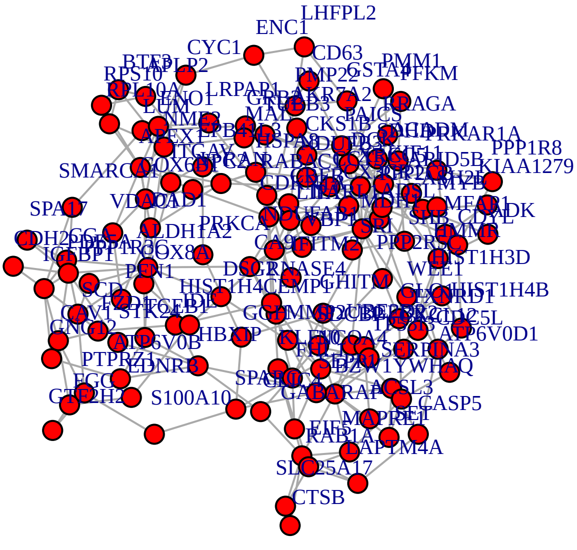

Pathway: TSAI_RESPONSE_TO_RADIATION_THERAPY

There are 29 genes in this pathway. This pathway was detected by GSCA

BCR/ABL ALL
Major Gene (BCR/ABL): TSC22D1
Weight Factor: 1.335
Major Gene (NEG): COL6A2
Weight Factor: 1.279

NEG ALL
Major Gene (NEG): COL6A2
Weight Factor: 1.576
Major Gene (BCR/ABL): TSC22D1
Weight Factor: 1.013

MST2 of the coexpression network for BCR/ABL ALL

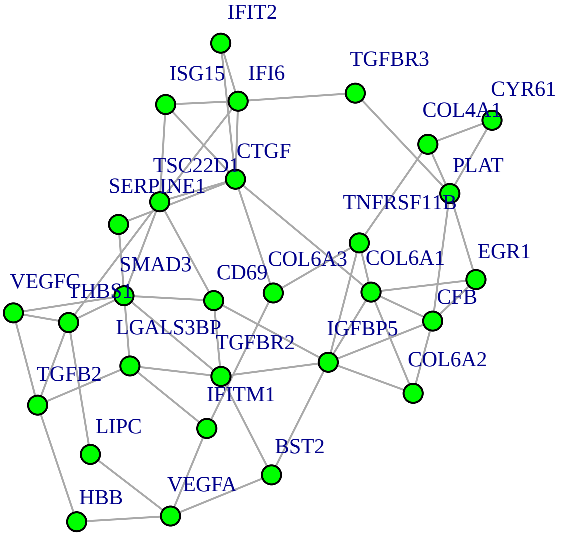

MST2 of the coexpression network for NEG ALL

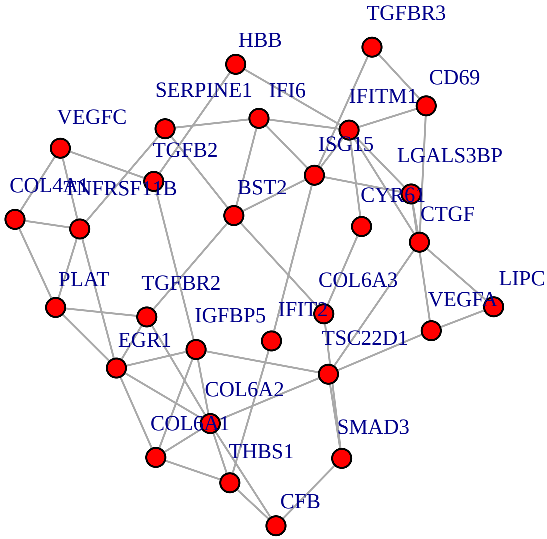

Pathway: DAVIES_MULTIPLE_MYELOMA_VS_MGUS_DN

There are 21 genes in this pathway. This pathway was detected by GSCA

BCR/ABL ALL
Major Gene (BCR/ABL): LASP1
Weight Factor: 1.305
Major Gene (NEG): CAP1
Weight Factor: 1.286

NEG ALL
Major Gene (NEG): CAP1
Weight Factor: 1.428
Major Gene (BCR/ABL): LASP1
Weight Factor: 1.199

MST2 of the coexpression network for BCR/ABL ALL

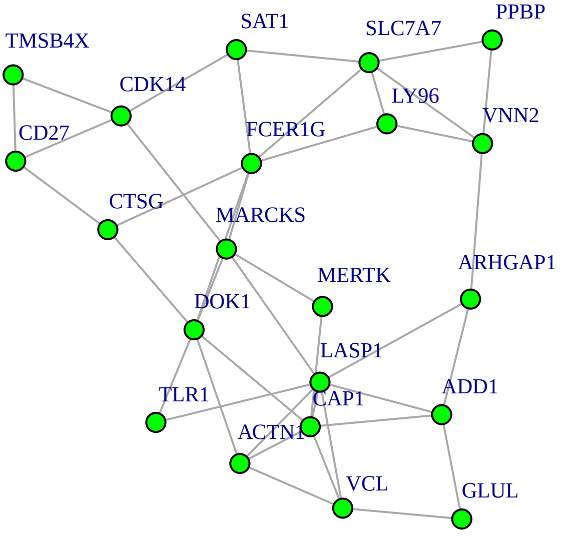

MST2 of the coexpression network for NEG ALL

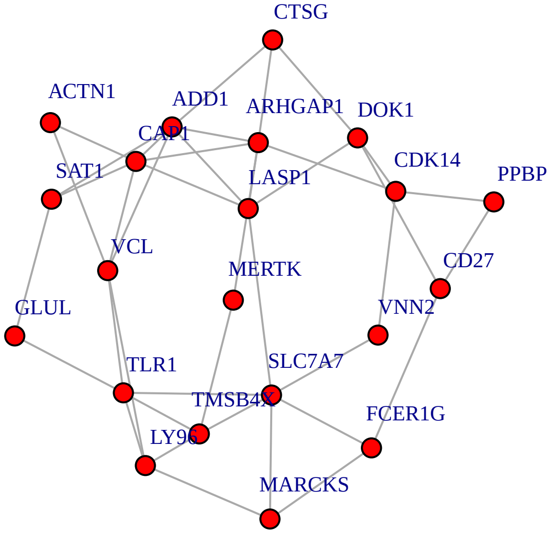

Pathway: FERRARI_RESPONSE_TO_FENRETINIDE_UP

There are 16 genes in this pathway. This pathway was detected by GSCA

BCR/ABL ALL
Major Gene (BCR/ABL): CXCL2
Weight Factor: 1.306
Major Gene (NEG): DDIT4
Weight Factor: 0.946

NEG ALL
Major Gene (NEG): DDIT4
Weight Factor: 1.334
Major Gene (BCR/ABL): CXCL2
Weight Factor: 1.028

MST2 of the coexpression network for BCR/ABL ALL

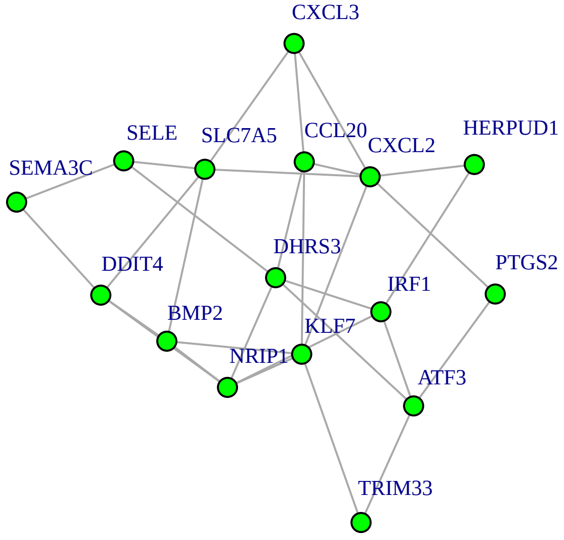

MST2 of the coexpression network for NEG ALL

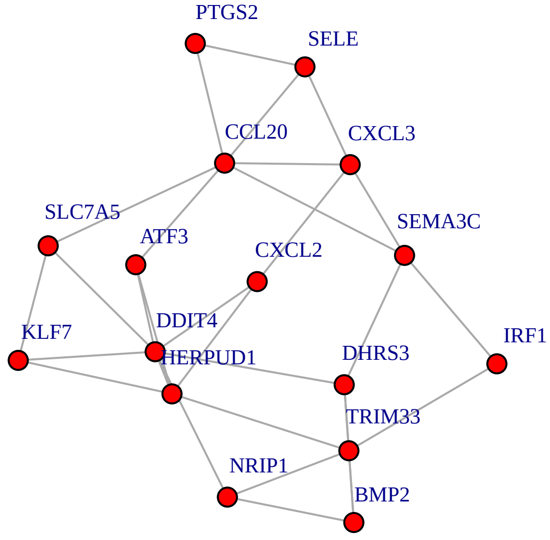

Pathway: TOOKER_GEMCITABINE_RESISTANCE_UP

There are 64 genes in this pathway. This pathway was detected by GSCA

BCR/ABL ALL
Major Gene (BCR/ABL): AATF
Weight Factor: 1.321
Major Gene (NEG): ASS1
Weight Factor: 0.967

NEG ALL
Major Gene (NEG): ASS1
Weight Factor: 1.4
Major Gene (BCR/ABL): AATF
Weight Factor: 1.021

MST2 of the coexpression network for BCR/ABL ALL

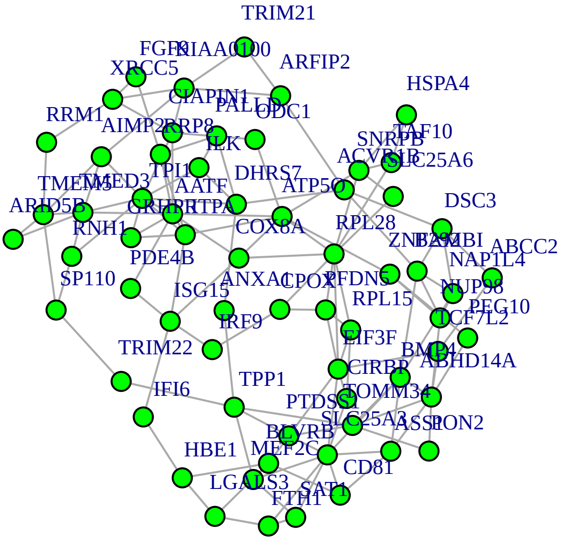

MST2 of the coexpression network for NEG ALL

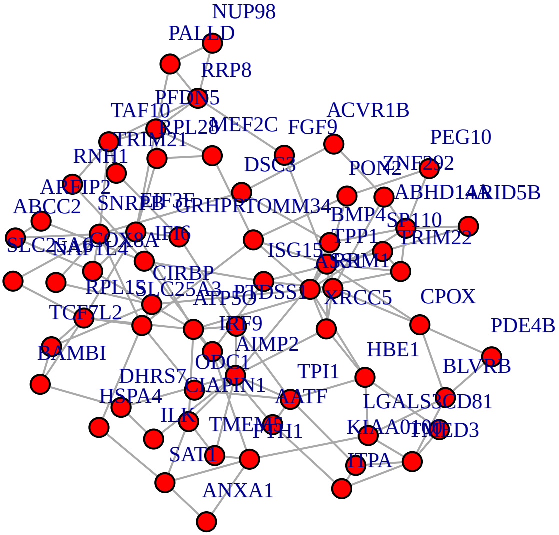

Pathway: TOOKER_RESPONSE_TO_BEXAROTENE_DN

There are 64 genes in this pathway. This pathway was detected by GSCA

BCR/ABL ALL
Major Gene (BCR/ABL): AATF
Weight Factor: 1.321
Major Gene (NEG): ASS1
Weight Factor: 0.967

NEG ALL
Major Gene (NEG): ASS1
Weight Factor: 1.4
Major Gene (BCR/ABL): AATF
Weight Factor: 1.021

MST2 of the coexpression network for BCR/ABL ALL

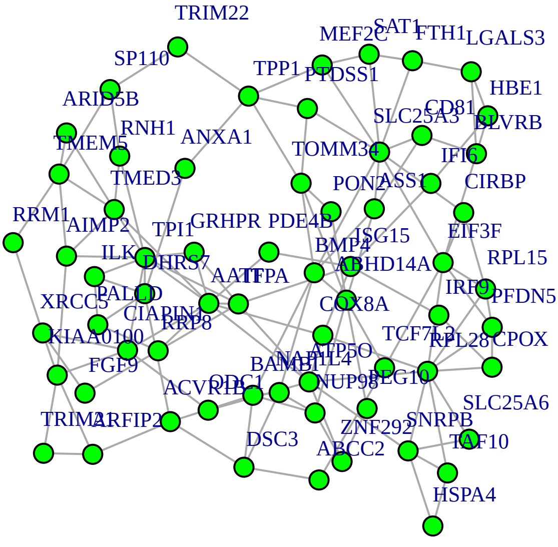

MST2 of the coexpression network for NEG ALL

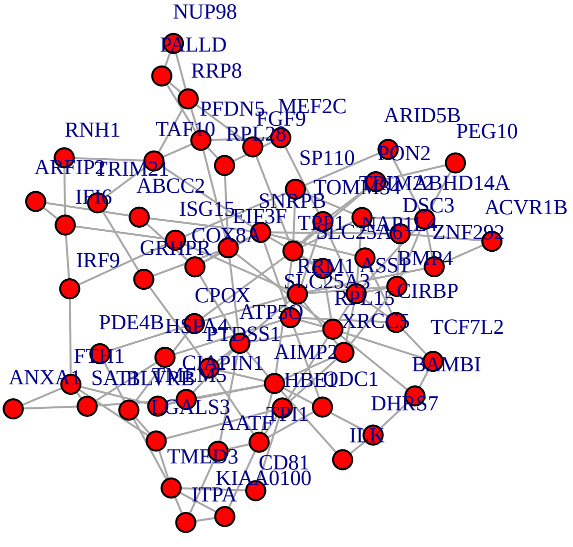

Pathway: ICHIBA_GRAFT_VERSUS_HOST_DISEASE_D7_UP

There are 86 genes in this pathway. This pathway was detected by GSCA

BCR/ABL ALL
Major Gene (BCR/ABL): PSMB10
Weight Factor: 1.375
Major Gene (NEG): CCL7
Weight Factor: 1.039

NEG ALL
Major Gene (NEG): CCL7
Weight Factor: 1.409
Major Gene (BCR/ABL): PSMB10
Weight Factor: 1.038

MST2 of the coexpression network for BCR/ABL ALL

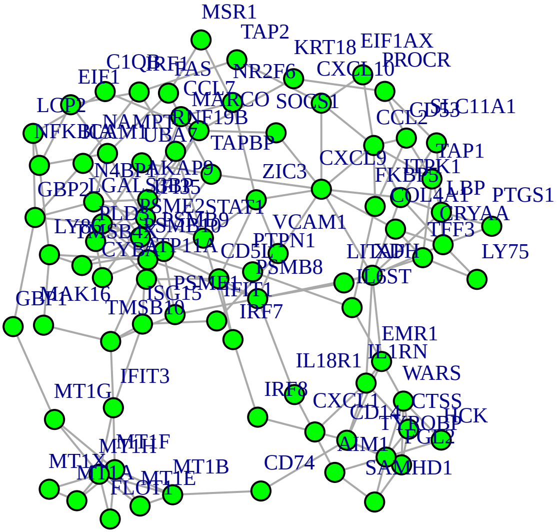

MST2 of the coexpression network for NEG ALL

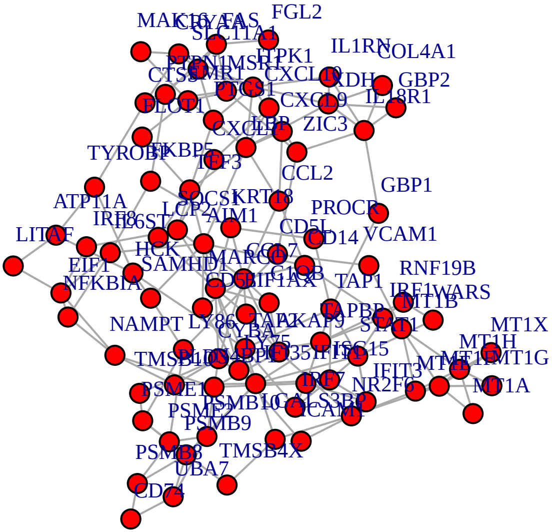

Pathway: LINDSTEDT_DENDRITIC_CELL_MATURATION_D

There are 57 genes in this pathway. This pathway was detected by GSCA

BCR/ABL ALL
Major Gene (BCR/ABL): CCR1
Weight Factor: 1.318
Major Gene (NEG): SYPL1
Weight Factor: 1.24

NEG ALL
Major Gene (NEG): SYPL1
Weight Factor: 1.502
Major Gene (BCR/ABL): CCR1
Weight Factor: 1.101

MST2 of the coexpression network for BCR/ABL ALL

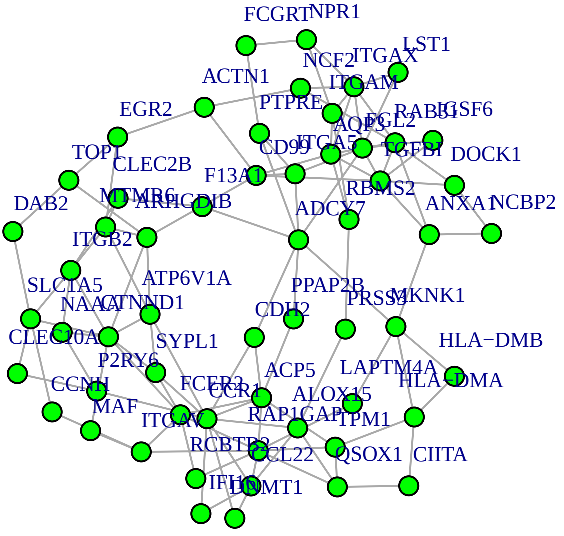

MST2 of the coexpression network for NEG ALL

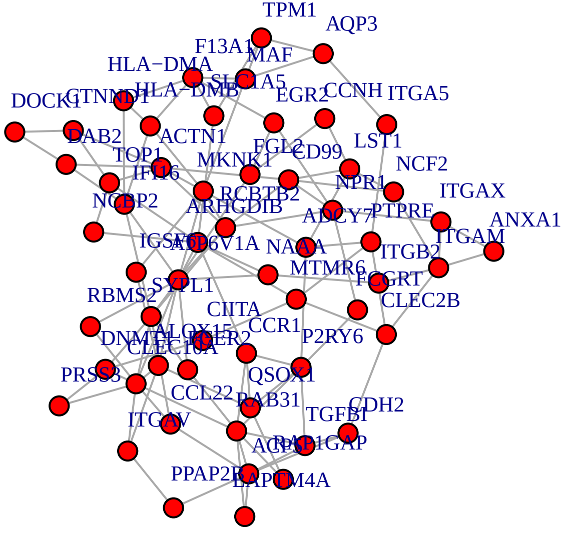

Pathway: NUTT_GBM_VS_AO_GLIOMA_DN

There are 42 genes in this pathway. This pathway was detected by GSCA

BCR/ABL ALL
Major Gene (BCR/ABL): RPL24
Weight Factor: 1.417
Major Gene (NEG): EIF3E
Weight Factor: 1.036

NEG ALL
Major Gene (NEG): EIF3E
Weight Factor: 1.466
Major Gene (BCR/ABL): RPL24
Weight Factor: 1.37

MST2 of the coexpression network for BCR/ABL ALL

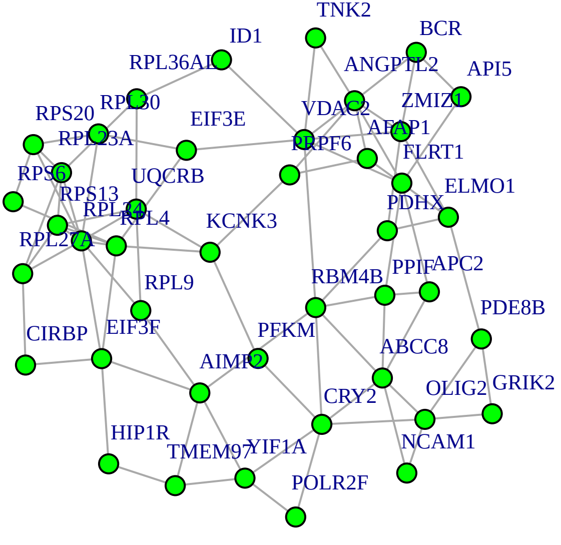

MST2 of the coexpression network for NEG ALL

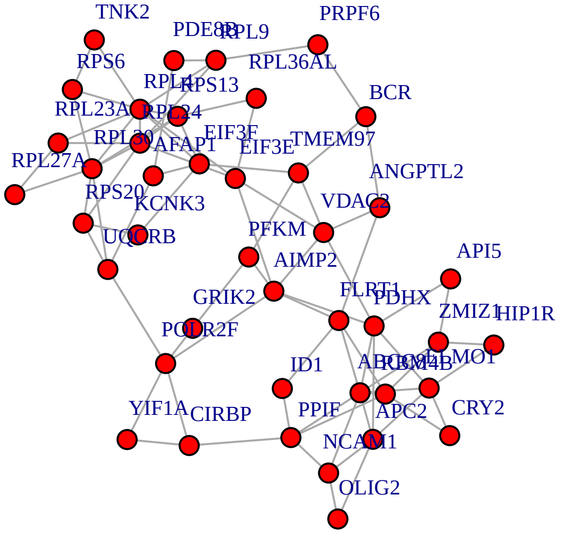

Pathway: ZHANG_INTERFERON_RESPONSE

There are 19 genes in this pathway. This pathway was detected by GSCA

BCR/ABL ALL
Major Gene (BCR/ABL): ISG15
Weight Factor: 1.312
Major Gene (NEG): ISG15
Weight Factor: 1.312

NEG ALL
Major Gene (NEG): ISG15
Weight Factor: 1.359
Major Gene (BCR/ABL): ISG15
Weight Factor: 1.359

MST2 of the coexpression network for BCR/ABL ALL

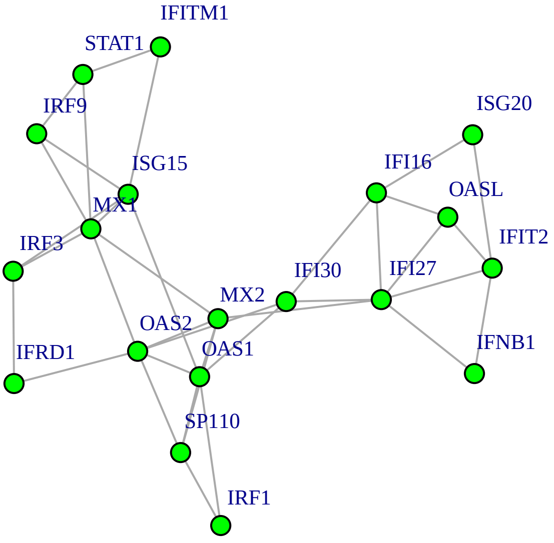

MST2 of the coexpression network for NEG ALL

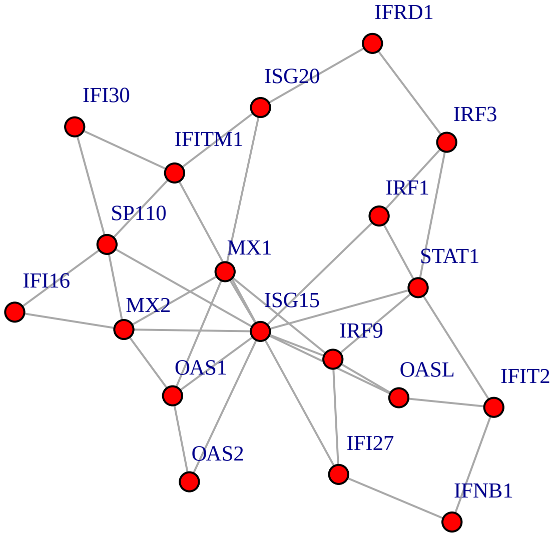

Pathway: RUTELLA_RESPONSE_TO_CSF2RB_AND_IL4_DN

There are 259 genes in this pathway. This pathway was detected by GSCA

BCR/ABL ALL
Major Gene (BCR/ABL): CNPY3
Weight Factor: 1.397
Major Gene (NEG): TIMP2
Weight Factor: 1.265

NEG ALL
Major Gene (NEG): TIMP2
Weight Factor: 1.598
Major Gene (BCR/ABL): CNPY3
Weight Factor: 1.399

MST2 of the coexpression network for BCR/ABL ALL

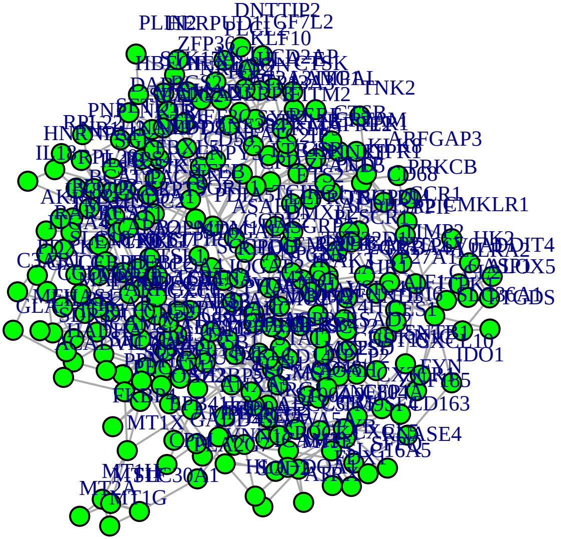

MST2 of the coexpression network for NEG ALL

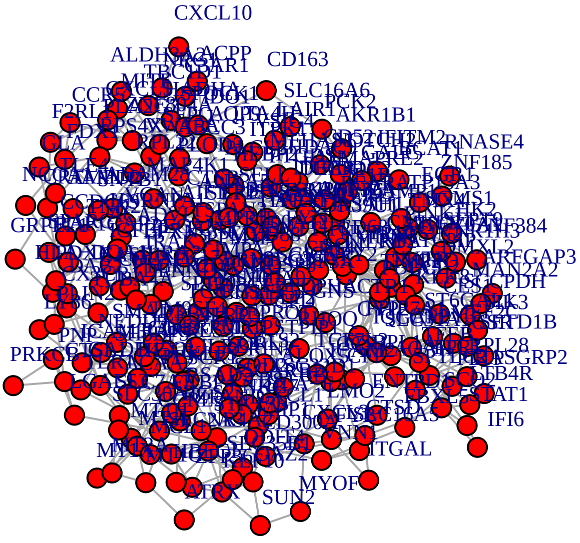

Pathway: RUTELLA_RESPONSE_TO_HGF_DN

There are 187 genes in this pathway. This pathway was detected by GSCA

BCR/ABL ALL
Major Gene (BCR/ABL): CNPY3
Weight Factor: 1.31
Major Gene (NEG): RABGAP1
Weight Factor: 1.21

NEG ALL
Major Gene (NEG): RABGAP1
Weight Factor: 1.437
Major Gene (BCR/ABL): CNPY3
Weight Factor: 1.373

MST2 of the coexpression network for BCR/ABL ALL

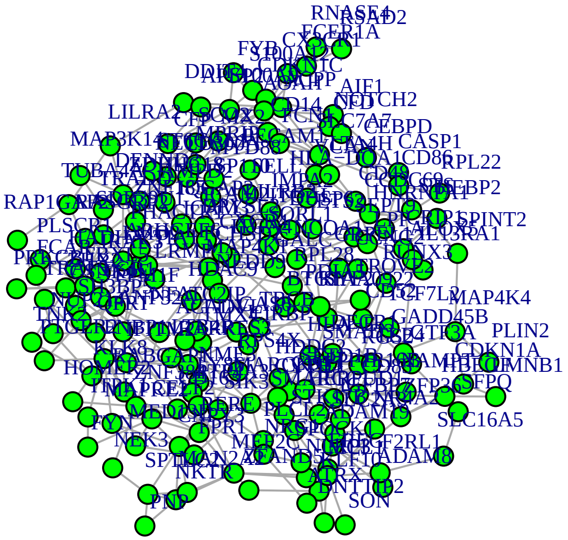

MST2 of the coexpression network for NEG ALL

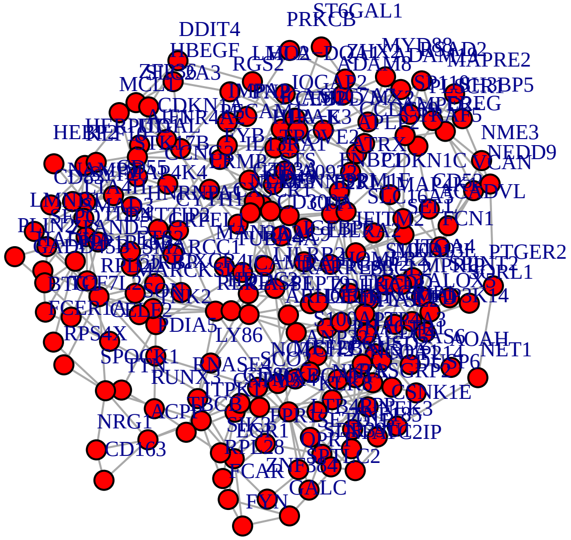

Pathway: VANTVEER_BREAST_CANCER_BRCA1_DN

There are 22 genes in this pathway. This pathway was detected by GSCA

BCR/ABL ALL
Major Gene (BCR/ABL): HDGFRP3
Weight Factor: 1.358
Major Gene (NEG): HSD11B2
Weight Factor: 0.999

NEG ALL
Major Gene (NEG): HSD11B2
Weight Factor: 1.354
Major Gene (BCR/ABL): HDGFRP3
Weight Factor: 1.074

MST2 of the coexpression network for BCR/ABL ALL

MST2 of the coexpression network for NEG ALL

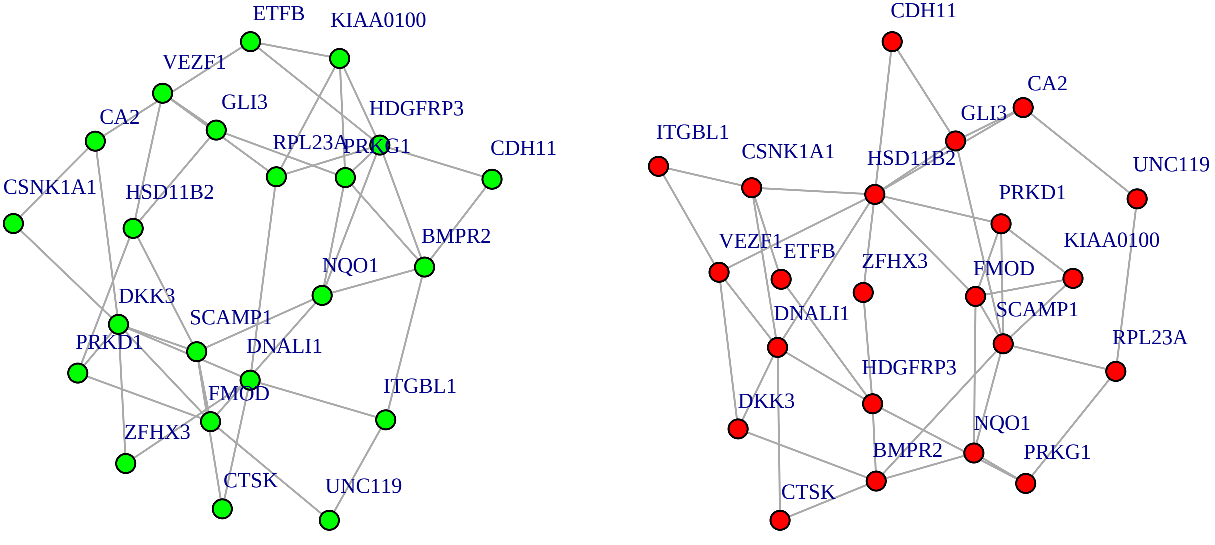

Pathway: HOFMANN_MYELODYSPLASTIC_SYNDROM_RISK_DN

There are 21 genes in this pathway. This pathway was detected by GSCA

BCR/ABL ALL
Major Gene (BCR/ABL): MTMR11
Weight Factor: 1.236
Major Gene (NEG): DLEC1
Weight Factor: 1.16

NEG ALL
Major Gene (NEG): DLEC1
Weight Factor: 1.419
Major Gene (BCR/ABL): MTMR11
Weight Factor: 1.066

MST2 of the coexpression network for BCR/ABL ALL

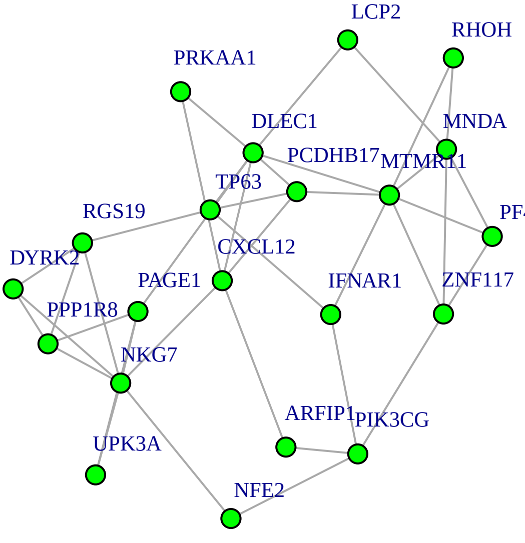

MST2 of the coexpression network for NEG ALL

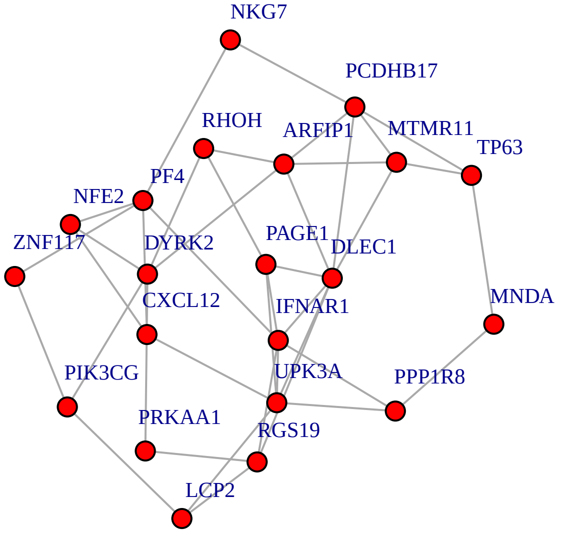

Pathway: LEE_RECENT_THYMIC_EMIGRANT

There are 87 genes in this pathway. This pathway was detected by GSCA

BCR/ABL ALL
Major Gene (BCR/ABL): LYPLA1
Weight Factor: 1.376
Major Gene (NEG): HDAC2
Weight Factor: 1.281

NEG ALL
Major Gene (NEG): HDAC2
Weight Factor: 1.507
Major Gene (BCR/ABL): LYPLA1
Weight Factor: 1.363

MST2 of the coexpression network for BCR/ABL ALL

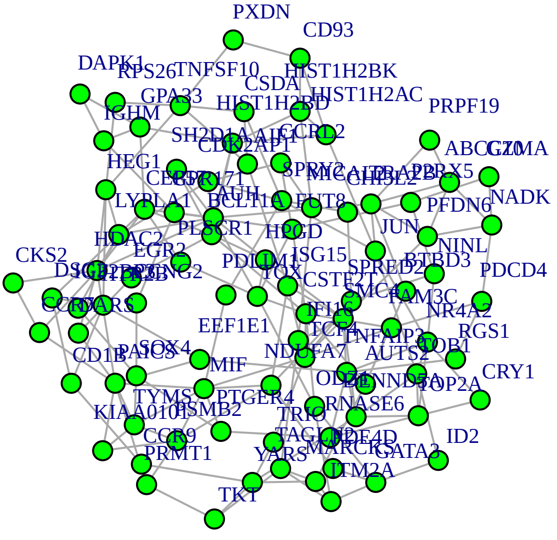

MST2 of the coexpression network for NEG ALL

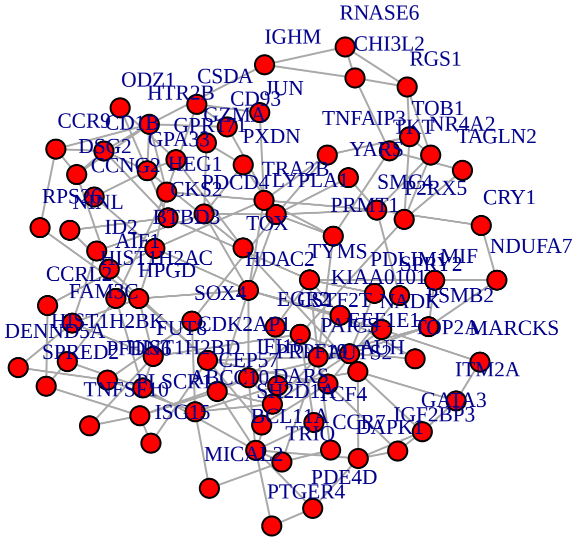

Pathway: SHAFFER_IRF4_TARGETS_IN_MYELOMA_VS_MATURE_B_LYMPHOCYTE

There are 86 genes in this pathway. This pathway was detected by GSCA

BCR/ABL ALL
Major Gene (BCR/ABL): YES1
Weight Factor: 1.445
Major Gene (NEG): PABPC4
Weight Factor: 1.016

NEG ALL
Major Gene (NEG): PABPC4
Weight Factor: 1.46
Major Gene (BCR/ABL): YES1
Weight Factor: 0.58

MST2 of the coexpression network for BCR/ABL ALL

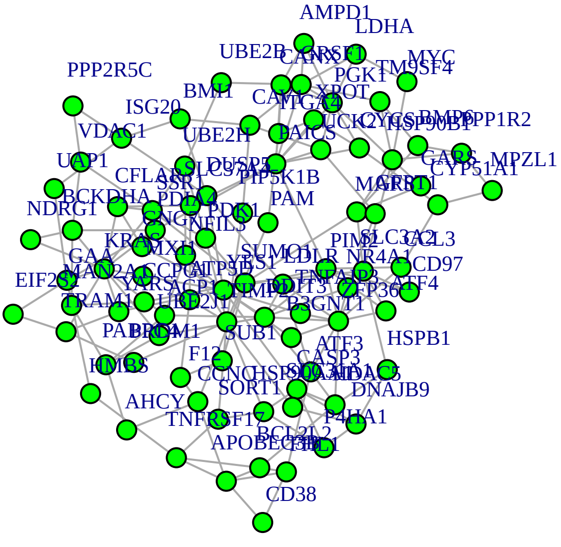

MST2 of the coexpression network for NEG ALL

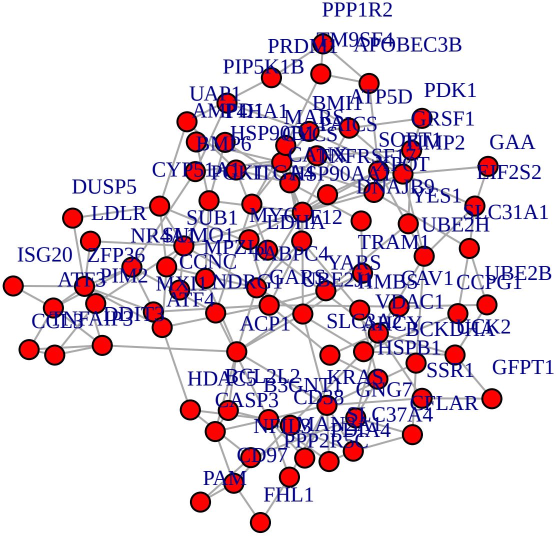

Pathway: SHAFFER_IRF4_TARGETS_IN_ACTIVATED_DENDRITIC_CELL

There are 50 genes in this pathway. This pathway was detected by GSCA

BCR/ABL ALL
Major Gene (BCR/ABL): PAICS
Weight Factor: 1.571
Major Gene (NEG): PAICS
Weight Factor: 1.571

MST2 of the coexpression network for BCR/ABL ALL

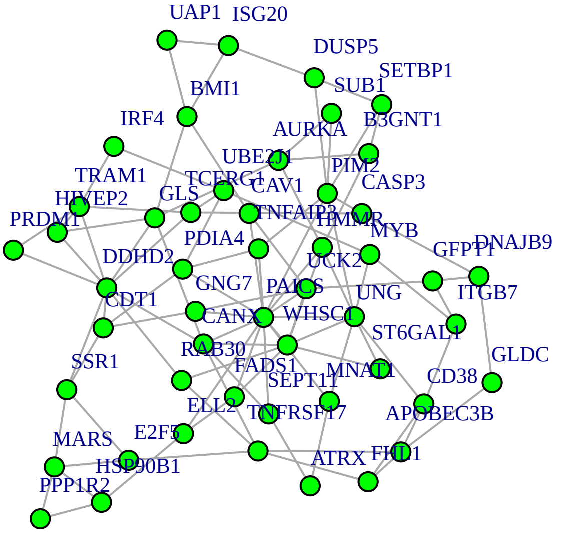

NEG ALL
Major Gene (NEG): PAICS
Weight Factor: 1.357
Major Gene (BCR/ABL): PAICS
Weight Factor: 1.357

MST2 of the coexpression network for NEG ALL

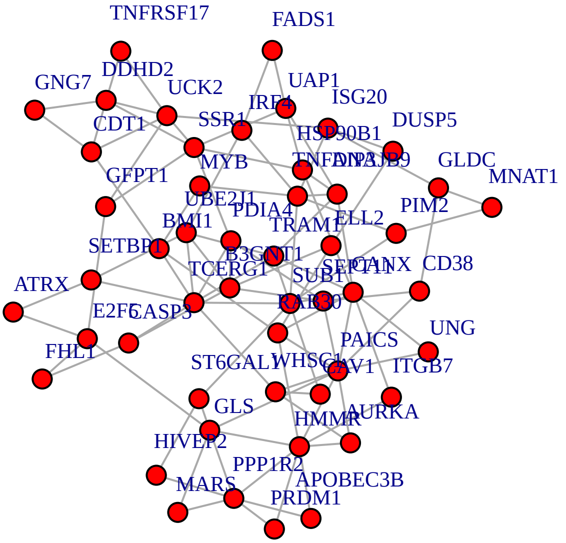

Pathway: FERRANDO_HOX11_NEIGHBORS

There are 15 genes in this pathway. This pathway was detected by GSCA

BCR/ABL ALL
Major Gene (BCR/ABL): MTHFD1
Weight Factor: 1.334
Major Gene (NEG): MCM4
Weight Factor: 1.223

NEG ALL
Major Gene (NEG): MCM4
Weight Factor: 1.281
Major Gene (BCR/ABL): MTHFD1
Weight Factor: 1.04

MST2 of the coexpression network for BCR/ABL ALL

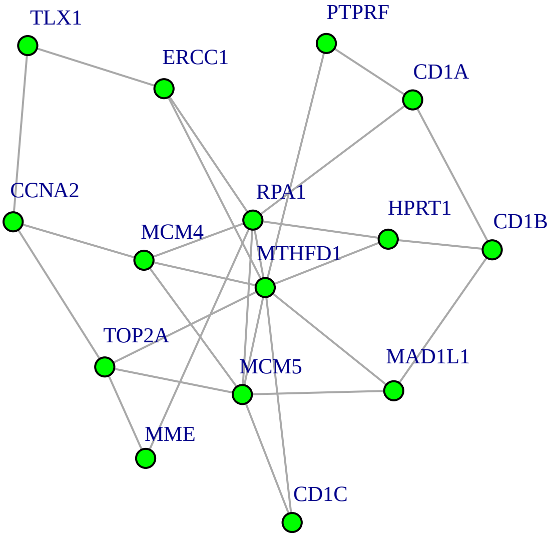

MST2 of the coexpression network for NEG ALL

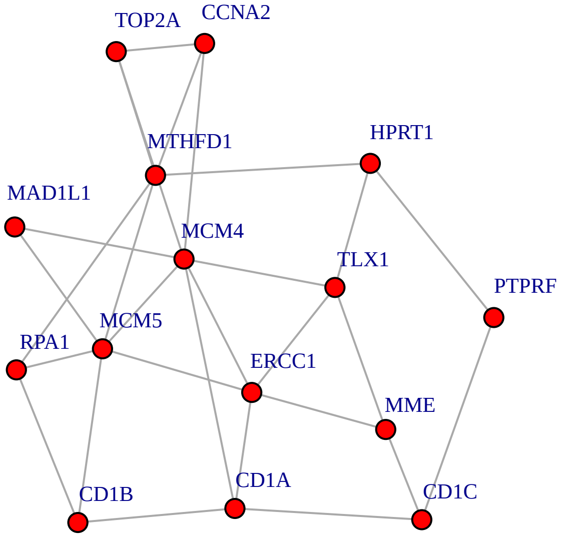

Pathway: CROONQUIST_IL6_DEPRIVATION_UP

There are 16 genes in this pathway. This pathway was detected by GSCA

BCR/ABL ALL
Major Gene (BCR/ABL): FCGRT
Weight Factor: 1.316
Major Gene (NEG): PDE1A
Weight Factor: 1.312

NEG ALL
Major Gene (NEG): PDE1A
Weight Factor: 1.475
Major Gene (BCR/ABL): FCGRT
Weight Factor: 0.896

MST2 of the coexpression network for BCR/ABL ALL

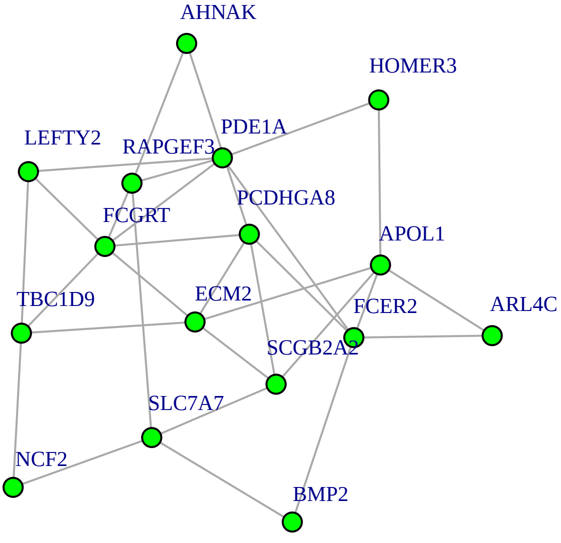

MST2 of the coexpression network for NEG ALL

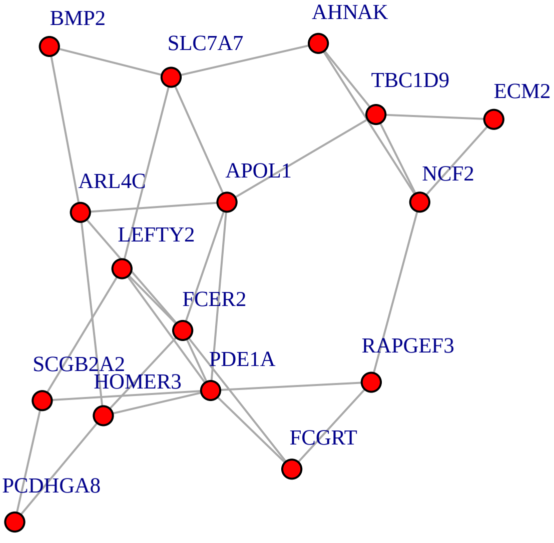

Pathway: ZHAN_LATE_DIFFERENTIATION_GENES_UP

There are 23 genes in this pathway. This pathway was detected by GSCA

BCR/ABL ALL
Major Gene (BCR/ABL): MCFD2
Weight Factor: 1.311
Major Gene (NEG): MCFD2
Weight Factor: 1.311

NEG ALL
Major Gene (NEG): MCFD2
Weight Factor: 1.405
Major Gene (BCR/ABL): MCFD2
Weight Factor: 1.405

MST2 of the coexpression network for BCR/ABL ALL

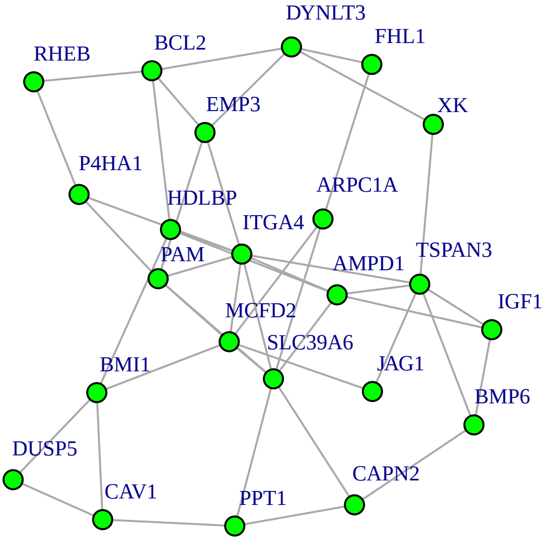

MST2 of the coexpression network for NEG ALL

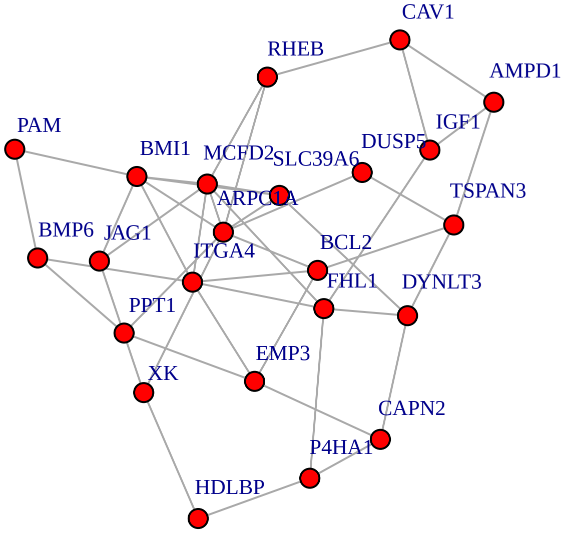

Pathway: ZHAN_V1_LATE_DIFFERENTIATION_GENES_UP

There are 30 genes in this pathway. This pathway was detected by GSCA

BCR/ABL ALL
Major Gene (BCR/ABL): TCN2
Weight Factor: 1.215
Major Gene (NEG): TIMP2
Weight Factor: 0.921

NEG ALL
Major Gene (NEG): TIMP2
Weight Factor: 1.461
Major Gene (BCR/ABL): TCN2
Weight Factor: 1.263

MST2 of the coexpression network for BCR/ABL ALL

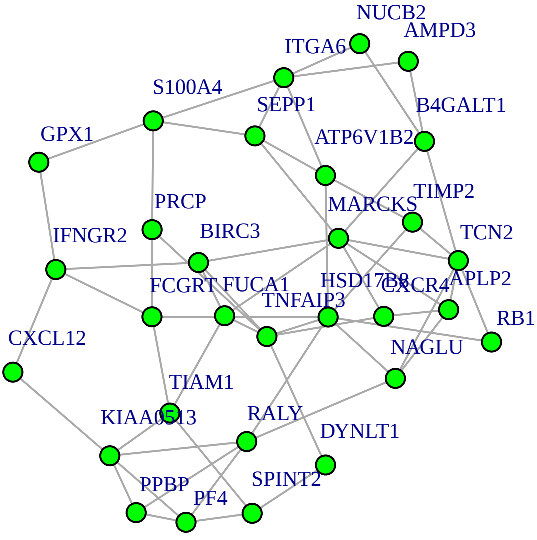

MST2 of the coexpression network for NEG ALL

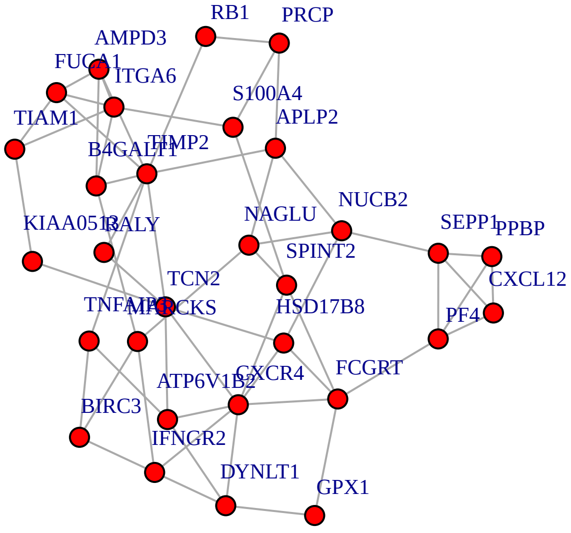

Pathway: VALK_AML_CLUSTER_2

There are 25 genes in this pathway. This pathway was detected by GSCA

BCR/ABL ALL
Major Gene (BCR/ABL): JAG1
Weight Factor: 1.309
Major Gene (NEG): TRIM16
Weight Factor: 0.782

NEG ALL
Major Gene (NEG): TRIM16
Weight Factor: 1.334
Major Gene (BCR/ABL): JAG1
Weight Factor: 1.129

MST2 of the coexpression network for BCR/ABL ALL

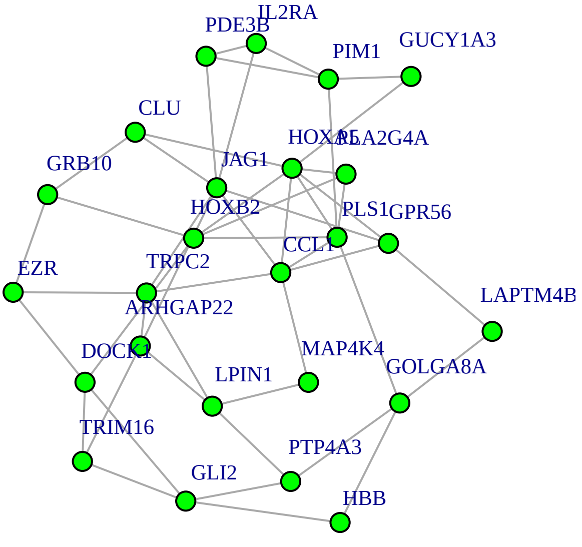

MST2 of the coexpression network for NEG ALL

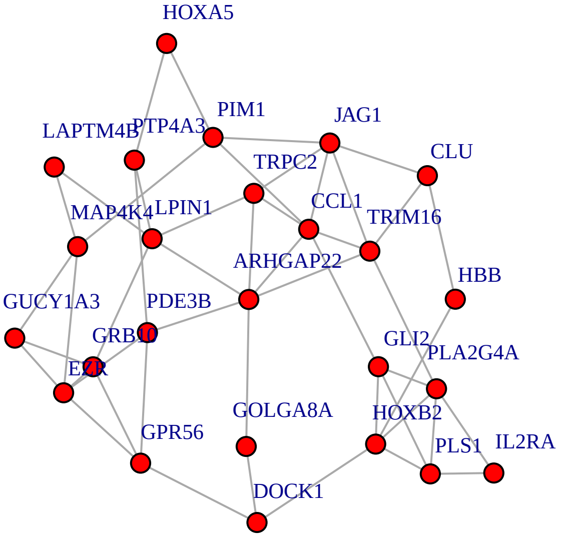

Pathway: VALK_AML_CLUSTER_4

There are 19 genes in this pathway. This pathway was detected by GSCA

BCR/ABL ALL
Major Gene (BCR/ABL): FZD6
Weight Factor: 1.334
Major Gene (NEG): B4GALT6
Weight Factor: 0.828

NEG ALL
Major Gene (NEG): B4GALT6
Weight Factor: 1.256
Major Gene (BCR/ABL): FZD6
Weight Factor: 0.805

MST2 of the coexpression network for BCR/ABL ALL

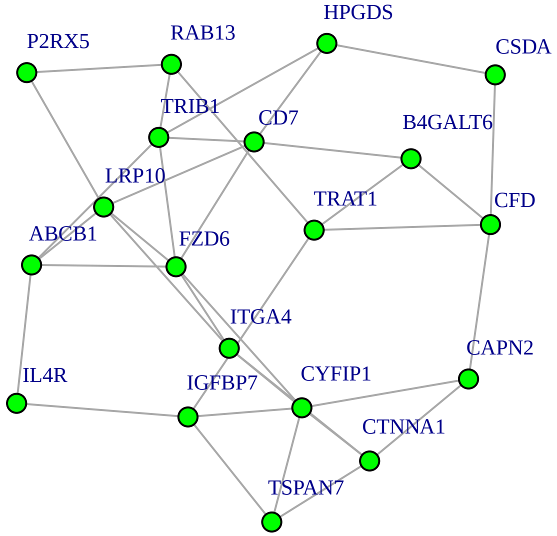

MST2 of the coexpression network for NEG ALL

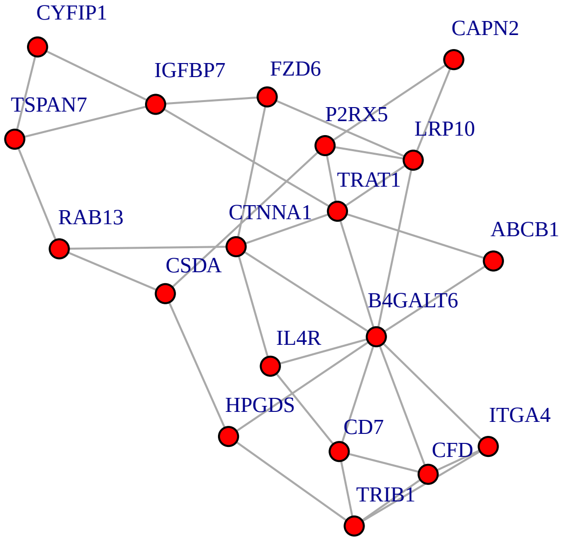

Pathway: VALK_AML_CLUSTER_9

There are 29 genes in this pathway. This pathway was detected by GSCA

BCR/ABL ALL
Major Gene (BCR/ABL): ICAM4
Weight Factor: 1.293
Major Gene (NEG): ICAM4
Weight Factor: 1.293

NEG ALL
Major Gene (NEG): ICAM4
Weight Factor: 1.515
Major Gene (BCR/ABL): ICAM4
Weight Factor: 1.515

MST2 of the coexpression network for BCR/ABL ALL

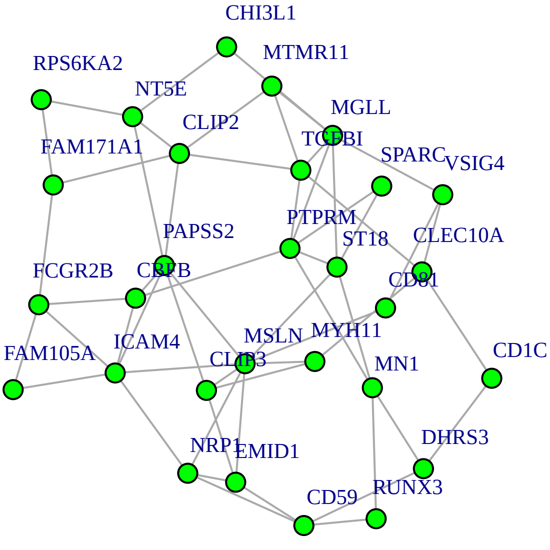

MST2 of the coexpression network for NEG ALL

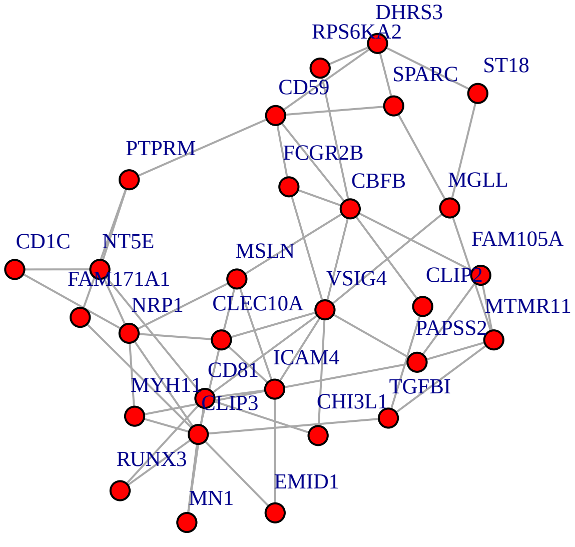

Pathway: VALK_AML_CLUSTER_10

There are 30 genes in this pathway. This pathway was detected by GSCA

BCR/ABL ALL
Major Gene (BCR/ABL): ARHGEF17
Weight Factor: 1.27
Major Gene (NEG): SPTBN1
Weight Factor: 1.219

NEG ALL
Major Gene (NEG): SPTBN1
Weight Factor: 1.403
Major Gene (BCR/ABL): ARHGEF17
Weight Factor: 1.037

MST2 of the coexpression network for BCR/ABL ALL

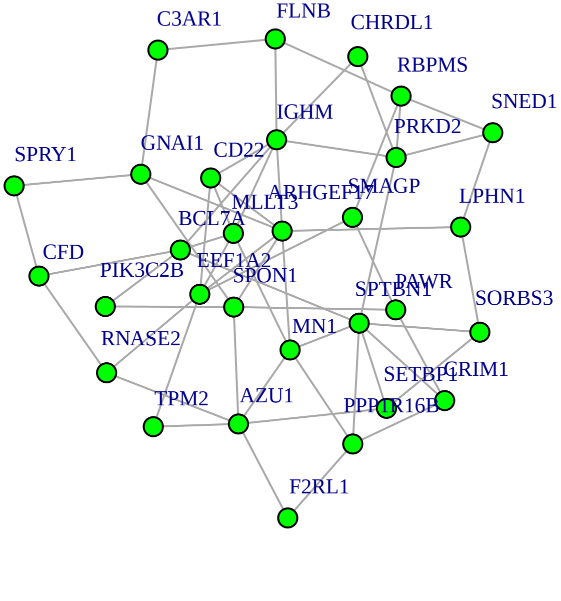

MST2 of the coexpression network for NEG ALL

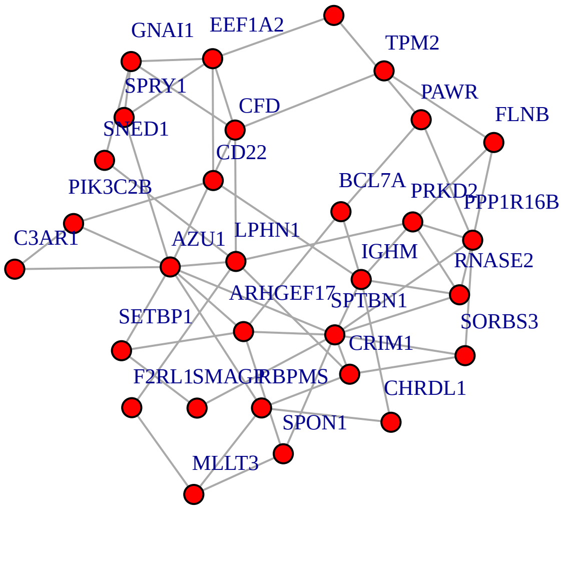

Pathway: VALK_AML_CLUSTER_13

There are 24 genes in this pathway. This pathway was detected by GSCA

BCR/ABL ALL
Major Gene (BCR/ABL): THSD7A
Weight Factor: 1.271
Major Gene (NEG): ADRA2C
Weight Factor: 1.066

NEG ALL
Major Gene (NEG): ADRA2C
Weight Factor: 1.288
Major Gene (BCR/ABL): THSD7A
Weight Factor: 0.967

MST2 of the coexpression network for BCR/ABL ALL

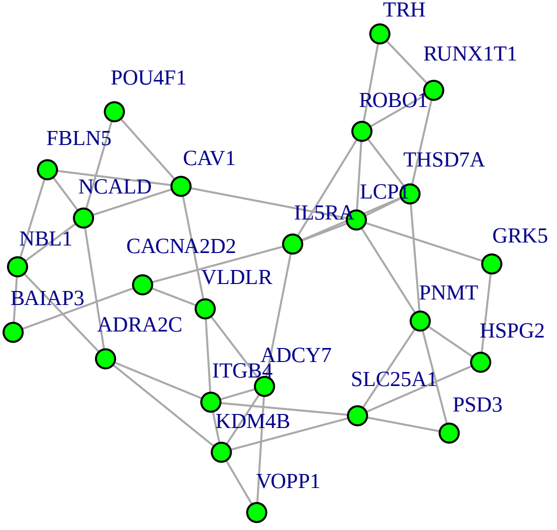

MST2 of the coexpression network for NEG ALL

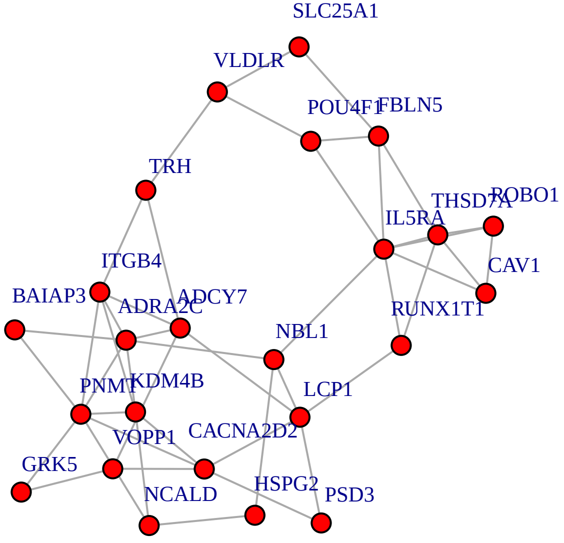

Pathway: VALK_AML_WITH_EVI1

There are 22 genes in this pathway. This pathway was detected by GSCA

BCR/ABL ALL
Major Gene (BCR/ABL): DMXL2
Weight Factor: 1.293
Major Gene (NEG): SPTBN1
Weight Factor: 1.109

NEG ALL
Major Gene (NEG): SPTBN1
Weight Factor: 1.359
Major Gene (BCR/ABL): DMXL2
Weight Factor: 0.806

MST2 of the coexpression network for BCR/ABL ALL

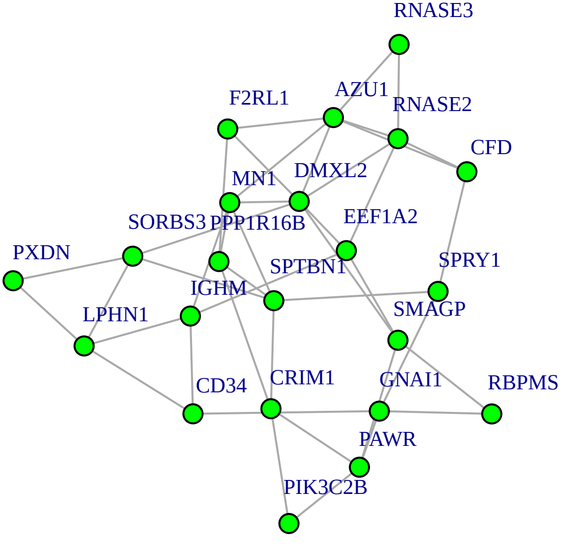

MST2 of the coexpression network for NEG ALL

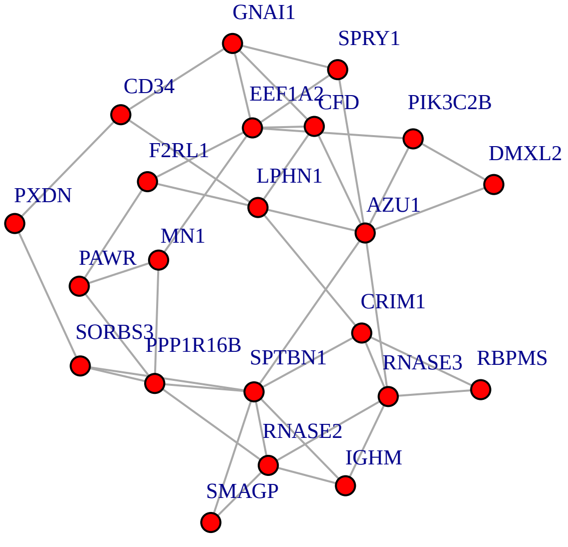

Pathway: JISON_SICKLE_CELL_DISEASE_UP

There are 175 genes in this pathway. This pathway was detected by GSCA

BCR/ABL ALL
Major Gene (BCR/ABL): BLOC1S1
Weight Factor: 1.436
Major Gene (NEG): AZU1
Weight Factor: 0.689

NEG ALL
Major Gene (NEG): AZU1
Weight Factor: 1.391
Major Gene (BCR/ABL): BLOC1S1
Weight Factor: 1.256

MST2 of the coexpression network for BCR/ABL ALL

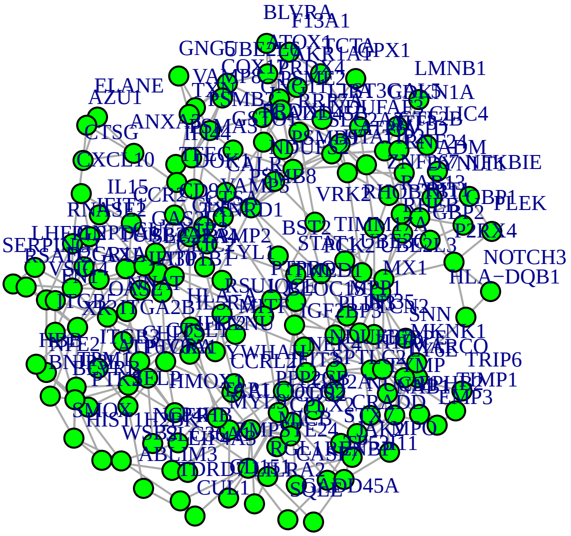

MST2 of the coexpression network for NEG ALL

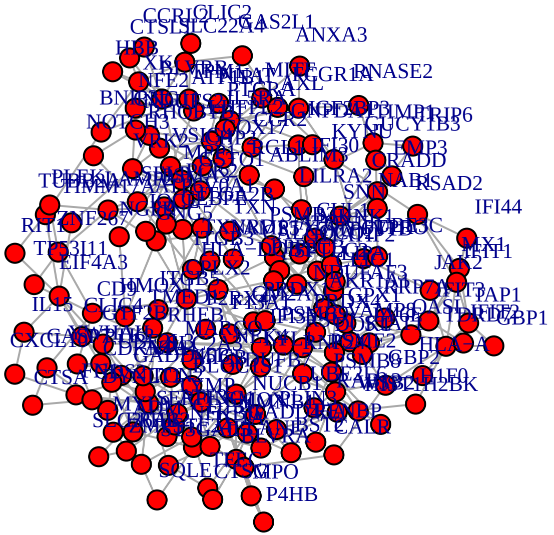

Pathway: CHIANG_LIVER_CANCER_SUBCLASS_UNANNOTATED_DN

There are 111 genes in this pathway. This pathway was detected by GSCA

BCR/ABL ALL
Major Gene (BCR/ABL): CDK4
Weight Factor: 1.428
Major Gene (NEG): HAT1
Weight Factor: 0.953

NEG ALL
Major Gene (NEG): HAT1
Weight Factor: 1.426
Major Gene (BCR/ABL): CDK4
Weight Factor: 1.202

MST2 of the coexpression network for BCR/ABL ALL

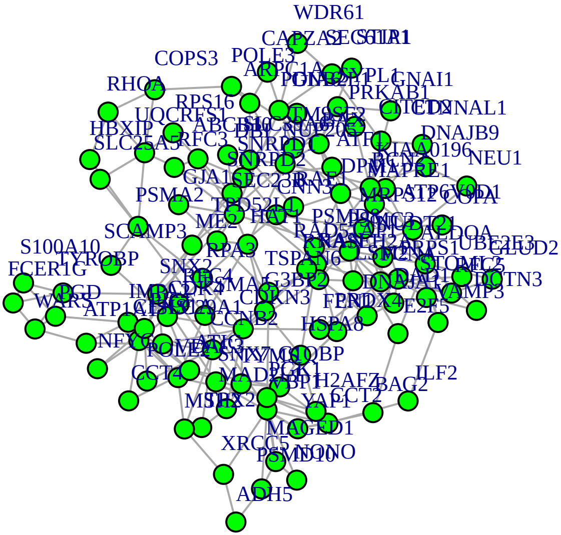

MST2 of the coexpression network for NEG ALL

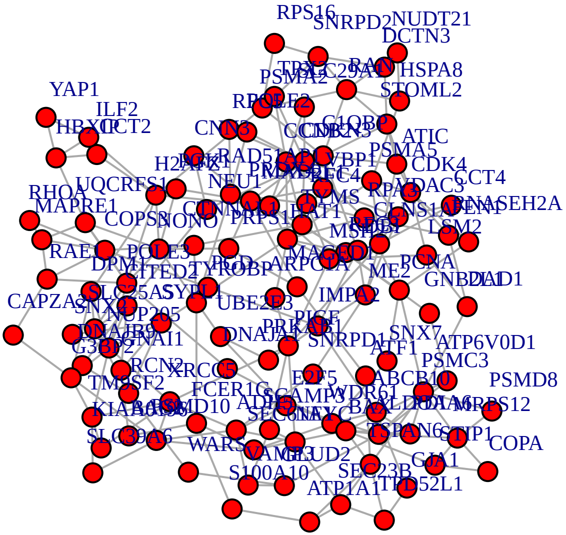

Pathway: HOSHIDA_LIVER_CANCER_LATE_RECURRENCE_UP

There are 53 genes in this pathway. This pathway was detected by GSCA

BCR/ABL ALL
Major Gene (BCR/ABL): MMP14
Weight Factor: 1.345
Major Gene (NEG): SLC8A1
Weight Factor: 1.248

NEG ALL
Major Gene (NEG): SLC8A1
Weight Factor: 1.567
Major Gene (BCR/ABL): MMP14
Weight Factor: 1.531

MST2 of the coexpression network for BCR/ABL ALL

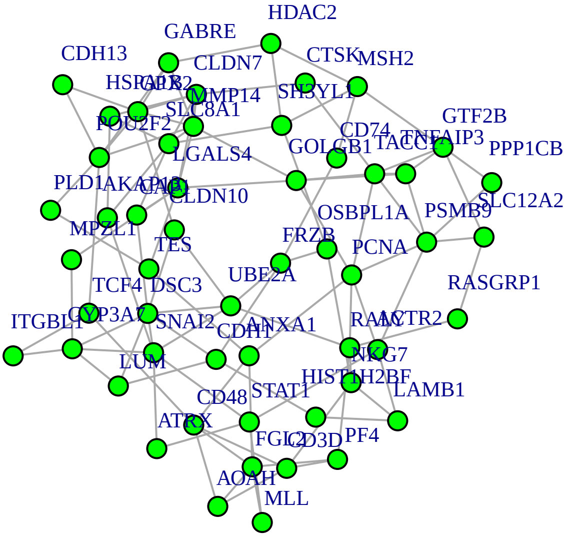

MST2 of the coexpression network for NEG ALL

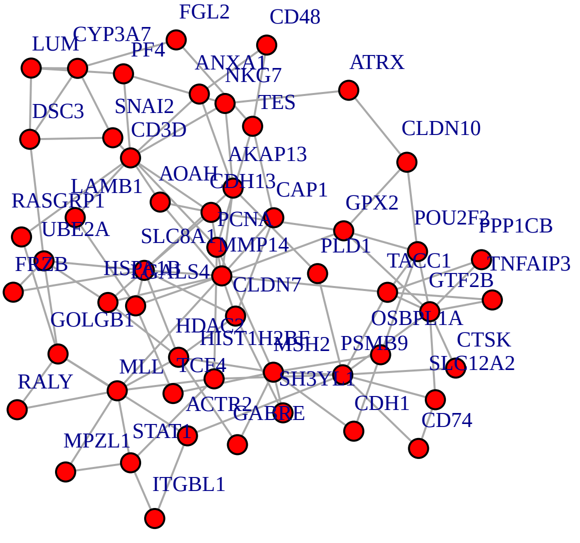

Pathway: HOSHIDA_LIVER_CANCER_SUBCLASS_S1

There are 228 genes in this pathway. This pathway was detected by GSCA

BCR/ABL ALL
Major Gene (BCR/ABL): ARHGDIB
Weight Factor: 1.363
Major Gene (NEG): TIMP2
Weight Factor: 1.236

NEG ALL
Major Gene (NEG): TIMP2
Weight Factor: 1.594
Major Gene (BCR/ABL): ARHGDIB
Weight Factor: 1.287

MST2 of the coexpression network for BCR/ABL ALL

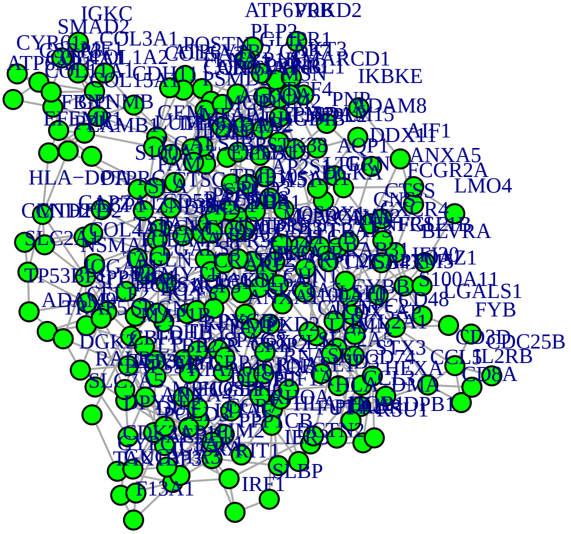

MST2 of the coexpression network for NEG ALL

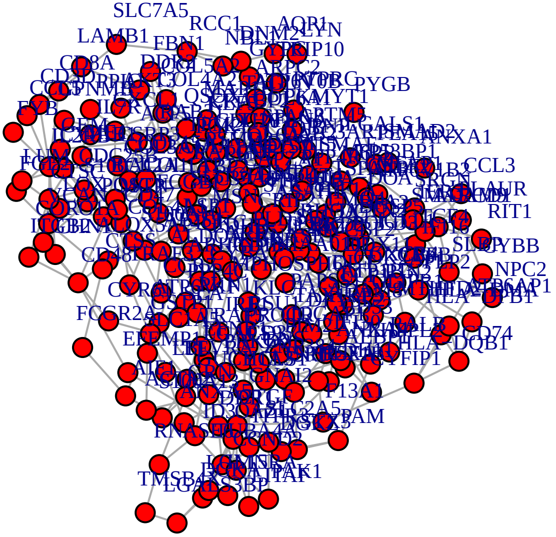

Pathway: GUTIERREZ_CHRONIC_LYMPHOCYTIC_LEUKEMIA_DN

There are 45 genes in this pathway. This pathway was detected by GSCA

BCR/ABL ALL
Major Gene (BCR/ABL): APOBEC3G
Weight Factor: 1.458
Major Gene (NEG): COL9A2
Weight Factor: 1.135

NEG ALL
Major Gene (NEG): COL9A2
Weight Factor: 1.501
Major Gene (BCR/ABL): APOBEC3G
Weight Factor: 1.126

MST2 of the coexpression network for BCR/ABL ALL

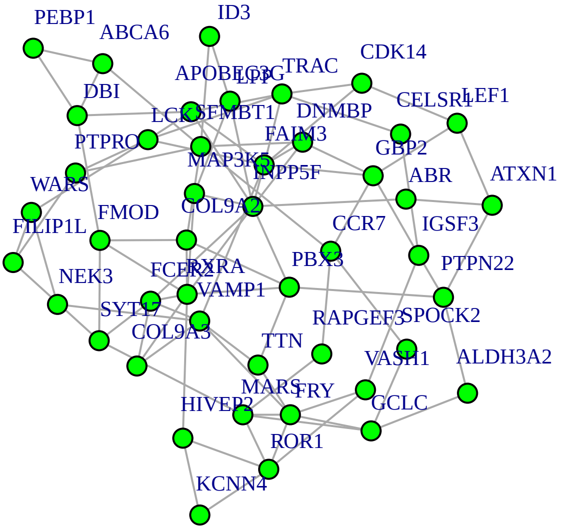

MST2 of the coexpression network for NEG ALL

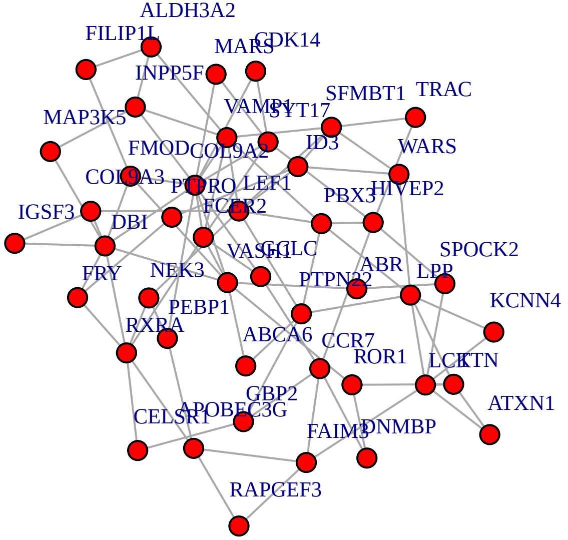

Pathway: GUTIERREZ_MULTIPLE_MYELOMA_DN

There are 29 genes in this pathway. This pathway was detected by GSCA

BCR/ABL ALL
Major Gene (BCR/ABL): WBSCR22
Weight Factor: 1.36
Major Gene (NEG): WBSCR22
Weight Factor: 1.36

NEG ALL
Major Gene (NEG): WBSCR22
Weight Factor: 1.339
Major Gene (BCR/ABL): WBSCR22
Weight Factor: 1.339

MST2 of the coexpression network for BCR/ABL ALL

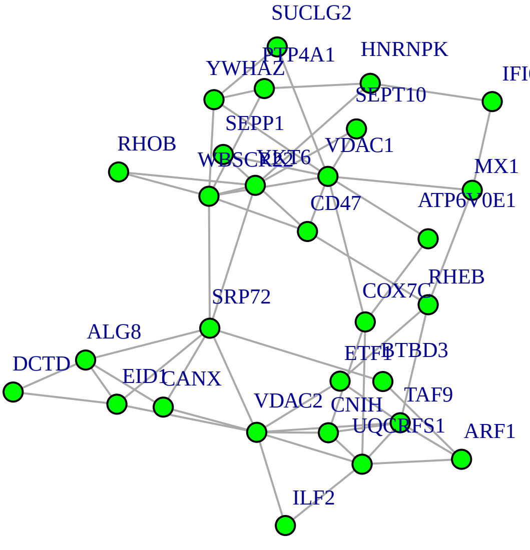

MST2 of the coexpression network for NEG ALL

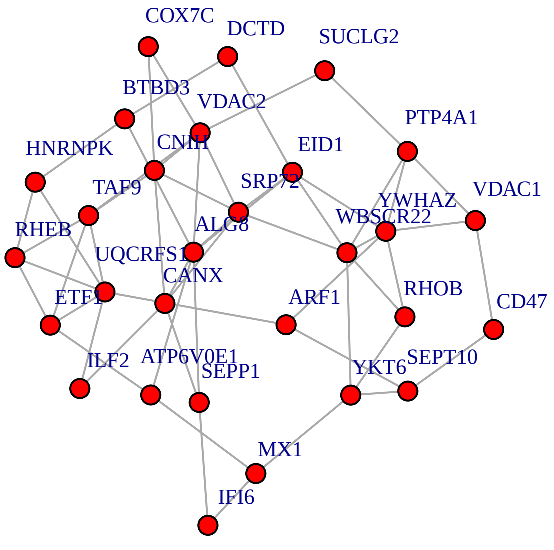

Pathway: NAKAYAMA_SOFT_TISSUE_TUMORS_PCA1_UP

There are 58 genes in this pathway. This pathway was detected by GSCA

BCR/ABL ALL
Major Gene (BCR/ABL): CSF1R
Weight Factor: 1.261
Major Gene (NEG): WISP2
Weight Factor: 1.009

NEG ALL
Major Gene (NEG): WISP2
Weight Factor: 1.431
Major Gene (BCR/ABL): CSF1R
Weight Factor: 1.194

MST2 of the coexpression network for BCR/ABL ALL

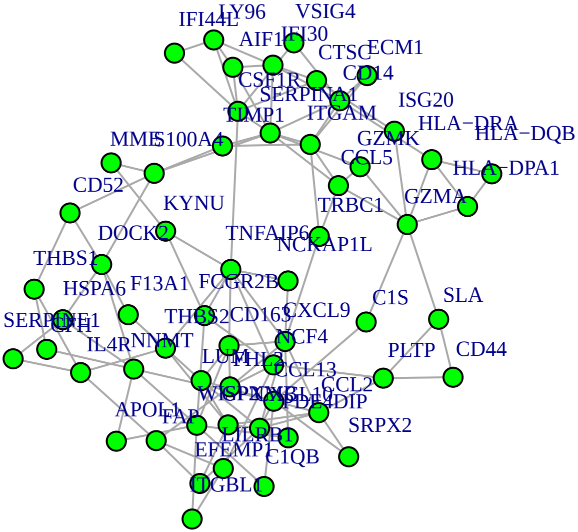

MST2 of the coexpression network for NEG ALL

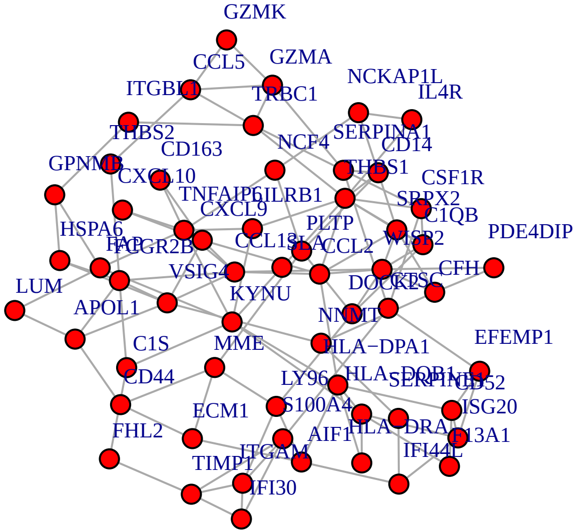

Pathway: YAO_TEMPORAL_RESPONSE_TO_PROGESTERONE_CLUSTER_13

There are 103 genes in this pathway. This pathway was detected by GSCA

BCR/ABL ALL
Major Gene (BCR/ABL): NDUFA2
Weight Factor: 1.527
Major Gene (NEG): PARK7
Weight Factor: 1.402

NEG ALL
Major Gene (NEG): PARK7
Weight Factor: 1.403
Major Gene (BCR/ABL): NDUFA2
Weight Factor: 1.393

MST2 of the coexpression network for BCR/ABL ALL

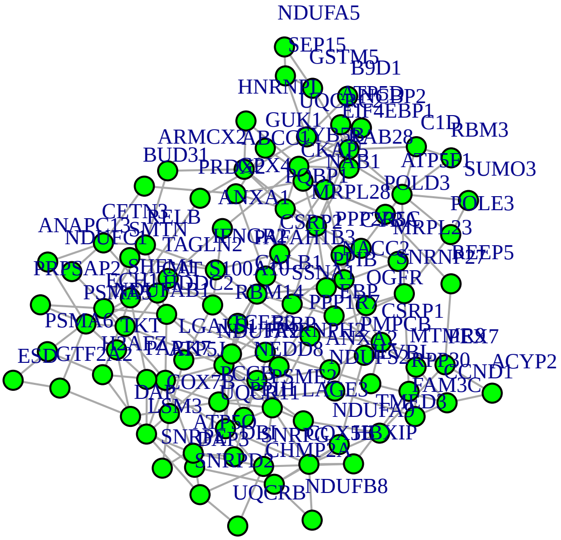

MST2 of the coexpression network for NEG ALL

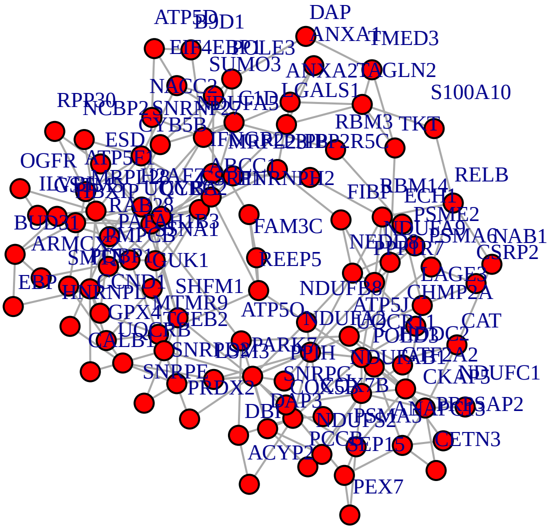

Pathway: SASSON_RESPONSE_TO_FORSKOLIN_UP

There are 68 genes in this pathway. This pathway was detected by GSCA

BCR/ABL ALL
Major Gene (BCR/ABL): PSMC3
Weight Factor: 1.355
Major Gene (NEG): GNAS
Weight Factor: 1.152

NEG ALL
Major Gene (NEG): GNAS
Weight Factor: 1.481
Major Gene (BCR/ABL): PSMC3
Weight Factor: 1.053

MST2 of the coexpression network for BCR/ABL ALL

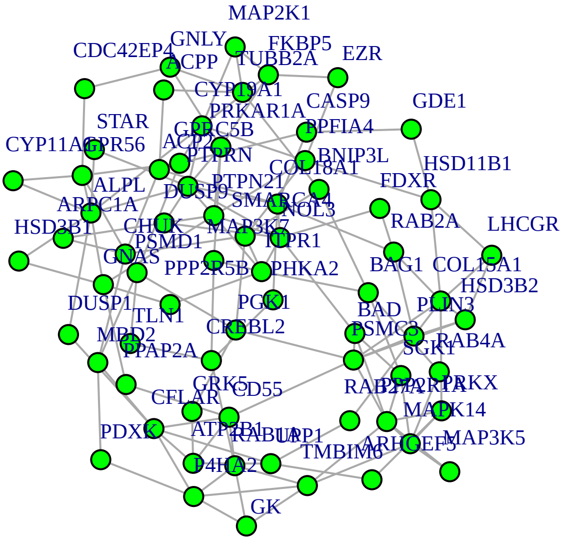

MST2 of the coexpression network for NEG ALL

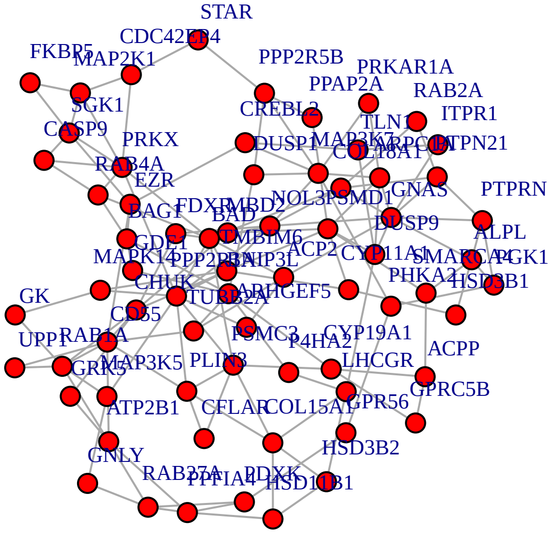

Pathway: RAGHAVACHARI_PLATELET_SPECIFIC_GENES

There are 63 genes in this pathway. This pathway was detected by GSCA

BCR/ABL ALL
Major Gene (BCR/ABL): ITGA2B
Weight Factor: 1.319
Major Gene (NEG): GNAS
Weight Factor: 1.175

NEG ALL
Major Gene (NEG): GNAS
Weight Factor: 1.541
Major Gene (BCR/ABL): ITGA2B
Weight Factor: 0.934

MST2 of the coexpression network for BCR/ABL ALL

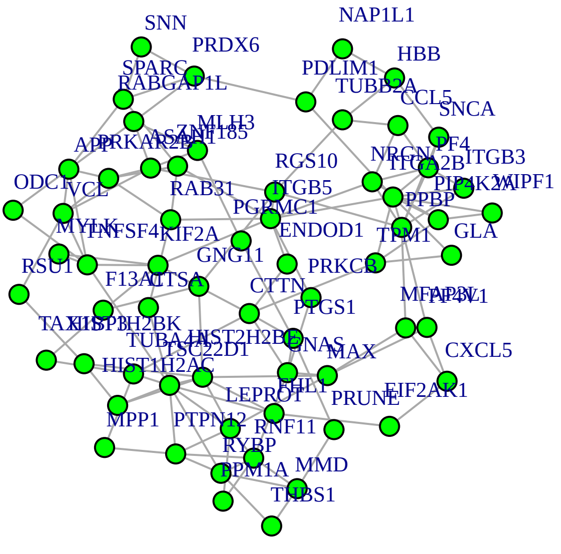

MST2 of the coexpression network for NEG ALL

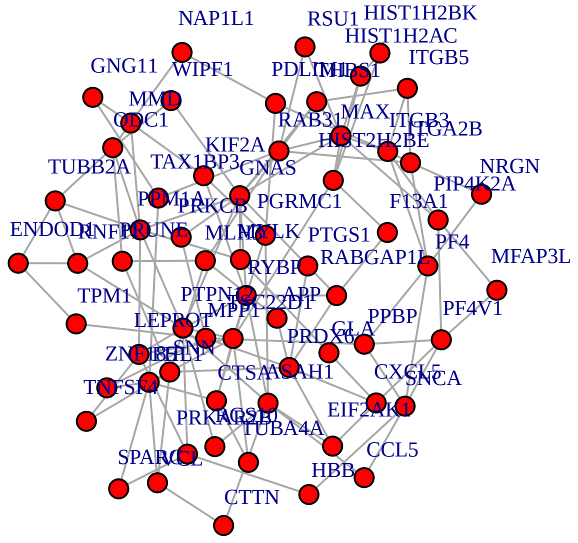

Pathway: RODWELL_AGING_KIDNEY_DN

There are 84 genes in this pathway. This pathway was detected by GSCA

BCR/ABL ALL
Major Gene (BCR/ABL): ADCY1
Weight Factor: 1.294
Major Gene (NEG): LRPPRC
Weight Factor: 1.145

NEG ALL
Major Gene (NEG): LRPPRC
Weight Factor: 1.416
Major Gene (BCR/ABL): ADCY1
Weight Factor: 0.891

MST2 of the coexpression network for BCR/ABL ALL

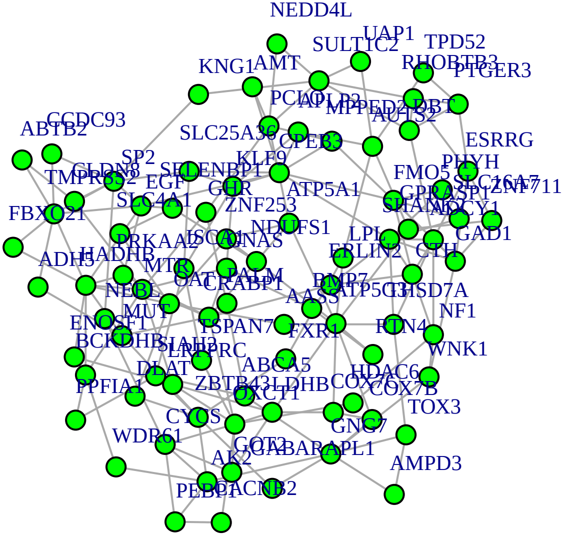

MST2 of the coexpression network for NEG ALL

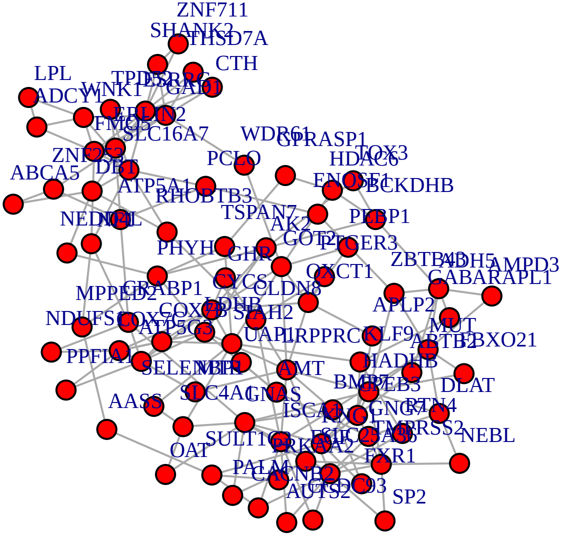

Pathway: KAYO_CALORIE_RESTRICTION_MUSCLE_DN

There are 66 genes in this pathway. This pathway was detected by GSCA

BCR/ABL ALL
Major Gene (BCR/ABL): HADHB
Weight Factor: 1.4
Major Gene (NEG): MDH1
Weight Factor: 1.375

MST2 of the coexpression network for BCR/ABL ALL

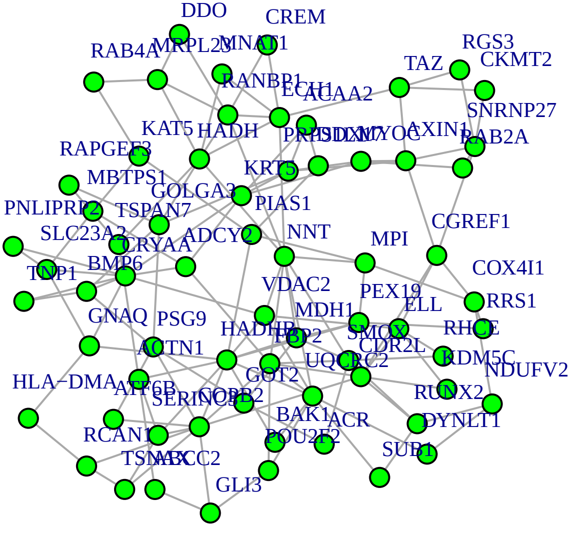

NEG ALL
Major Gene (NEG): MDH1
Weight Factor: 1.481
Major Gene (BCR/ABL): HADHB
Weight Factor: 1.401

MST2 of the coexpression network for NEG ALL

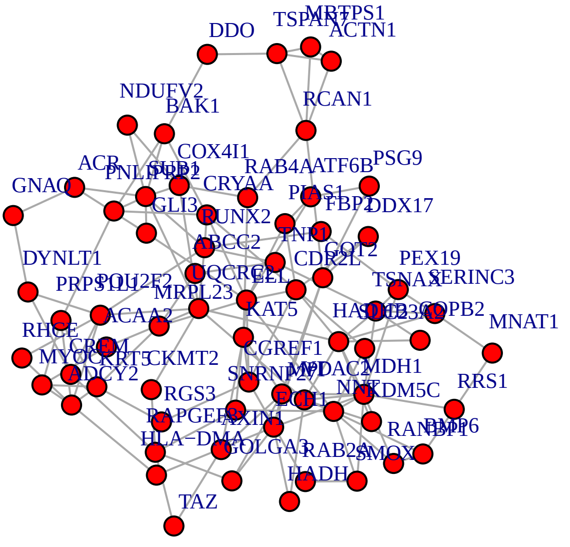

Pathway: LIANG_SILENCED_BY_METHYLATION_UP

There are 18 genes in this pathway. This pathway was detected by GSCA

BCR/ABL ALL
Major Gene (BCR/ABL): TIMP3
Weight Factor: 1.416
Major Gene (NEG): ASAH1
Weight Factor: 0.882

NEG ALL
Major Gene (NEG): ASAH1
Weight Factor: 1.46
Major Gene (BCR/ABL): TIMP3
Weight Factor: 1.367

MST2 of the coexpression network for BCR/ABL ALL

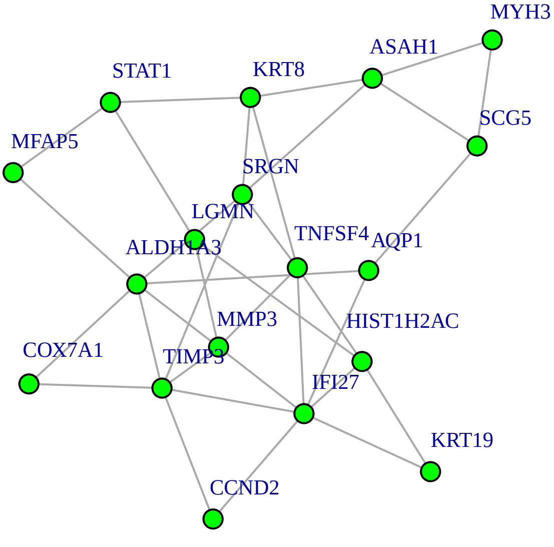

MST2 of the coexpression network for NEG ALL

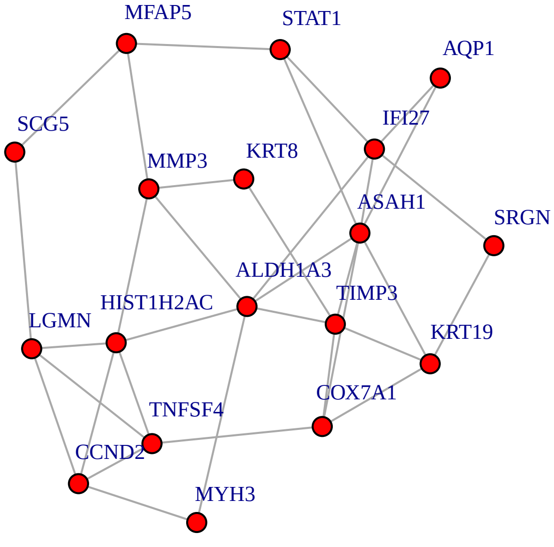

Pathway: KAAB_FAILED_HEART_ATRIUM_DN

There are 138 genes in this pathway. This pathway was detected by GSCA

BCR/ABL ALL
Major Gene (BCR/ABL): MCM3
Weight Factor: 1.468
Major Gene (NEG): PGK1
Weight Factor: 1.321

NEG ALL
Major Gene (NEG): PGK1
Weight Factor: 1.389
Major Gene (BCR/ABL): MCM3
Weight Factor: 1.129

MST2 of the coexpression network for BCR/ABL ALL

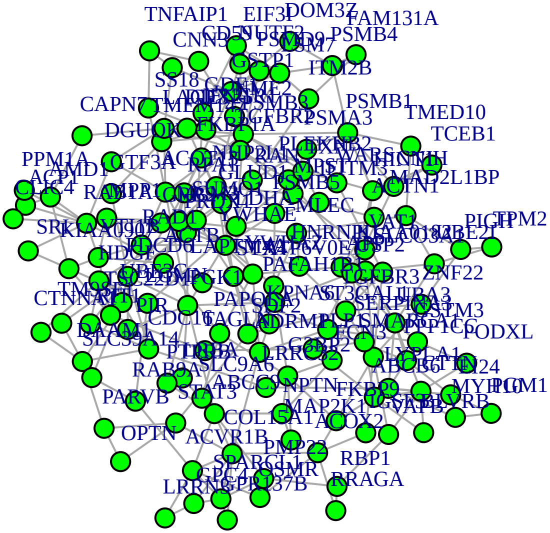

MST2 of the coexpression network for NEG ALL

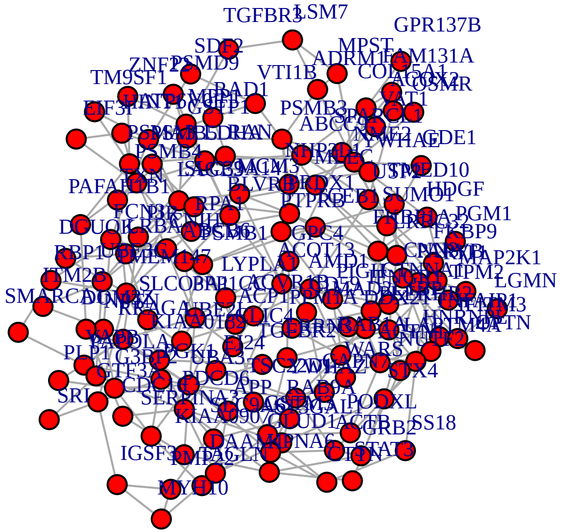

Pathway: IVANOVA_HEMATOPOIESIS_INTERMEDIATE_PROGENITOR

There are 24 genes in this pathway. This pathway was detected by GSCA

BCR/ABL ALL
Major Gene (BCR/ABL): BID
Weight Factor: 1.211
Major Gene (NEG): CTSZ
Weight Factor: 0.903

NEG ALL
Major Gene (NEG): CTSZ
Weight Factor: 1.31
Major Gene (BCR/ABL): BID
Weight Factor: 0.82

MST2 of the coexpression network for BCR/ABL ALL

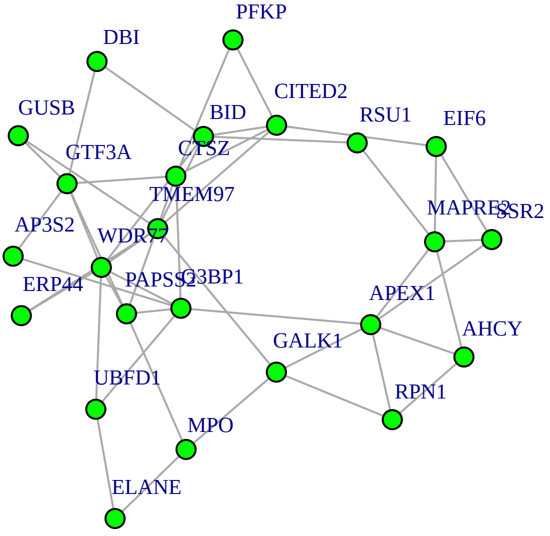

MST2 of the coexpression network for NEG ALL

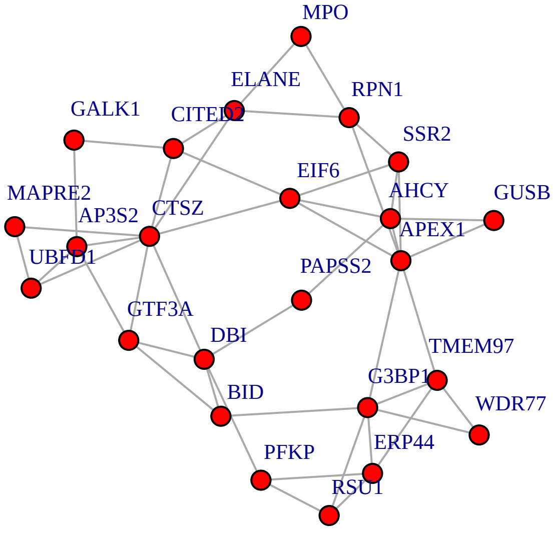

Pathway: IVANOVA_HEMATOPOIESIS_MATURE_CELL

There are 78 genes in this pathway. This pathway was detected by GSCA

BCR/ABL ALL
Major Gene (BCR/ABL): EPS15
Weight Factor: 1.394
Major Gene (NEG): XPO7
Weight Factor: 0.984

NEG ALL
Major Gene (NEG): XPO7
Weight Factor: 1.45
Major Gene (BCR/ABL): EPS15
Weight Factor: 1.177

MST2 of the coexpression network for BCR/ABL ALL

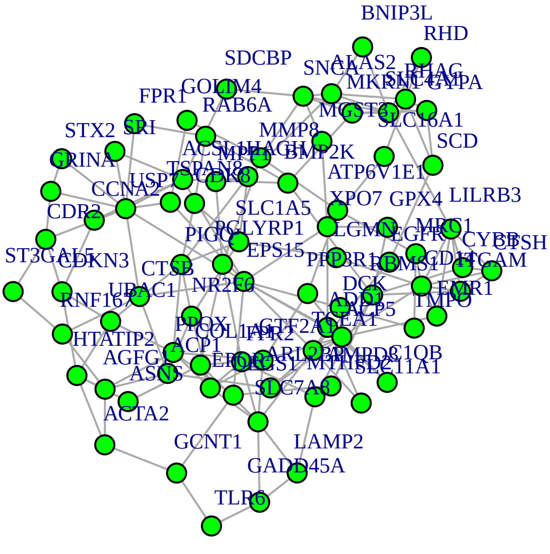

MST2 of the coexpression network for NEG ALL

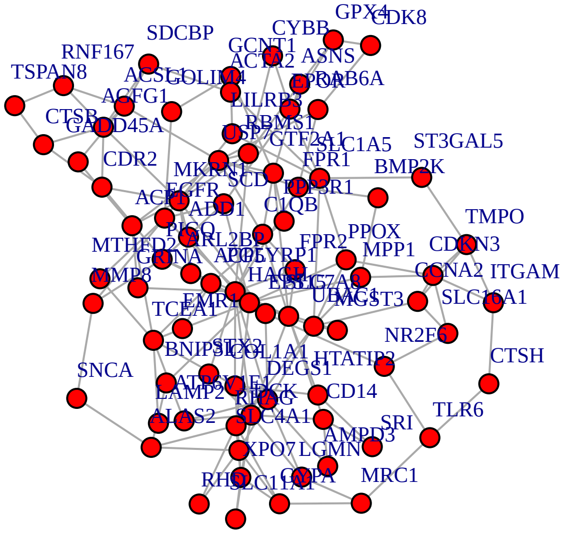

Pathway: DASU_IL6_SIGNALING_SCAR_DN

There are 16 genes in this pathway. This pathway was detected by GSCA

BCR/ABL ALL
Major Gene (BCR/ABL): HMOX1
Weight Factor: 1.2
Major Gene (NEG): HMOX1
Weight Factor: 1.2

NEG ALL
Major Gene (NEG): HMOX1
Weight Factor: 1.372
Major Gene (BCR/ABL): HMOX1
Weight Factor: 1.372

MST2 of the coexpression network for BCR/ABL ALL

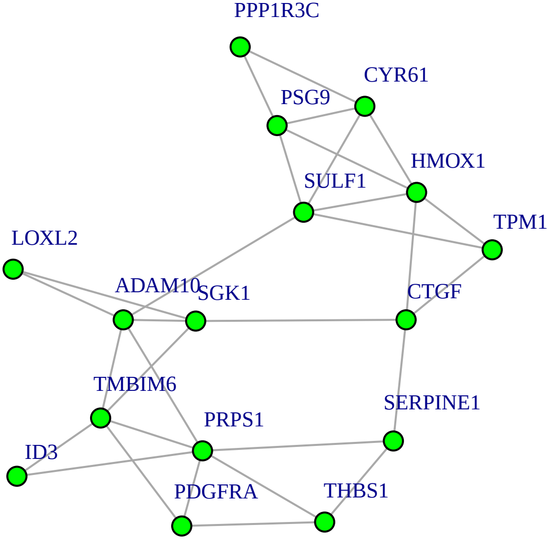

MST2 of the coexpression network for NEG ALL

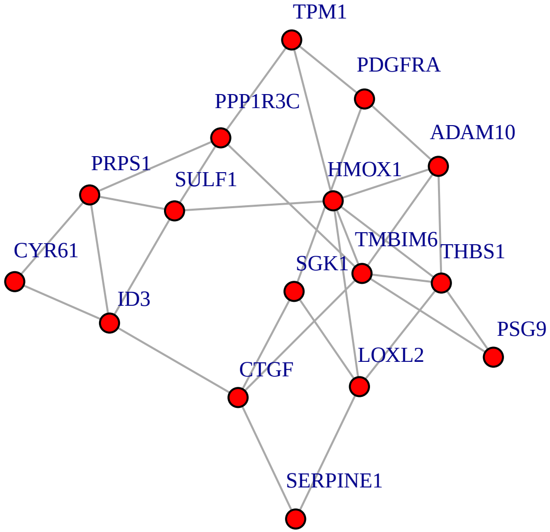

Pathway: MMS_MOUSE_LYMPH_HIGH_4HRS_UP

There are 29 genes in this pathway. This pathway was detected by GSCA

BCR/ABL ALL
Major Gene (BCR/ABL): VBP1
Weight Factor: 1.303
Major Gene (NEG): ATP6V1A
Weight Factor: 1.162

NEG ALL
Major Gene (NEG): ATP6V1A
Weight Factor: 1.384
Major Gene (BCR/ABL): VBP1
Weight Factor: 1.328

MST2 of the coexpression network for BCR/ABL ALL

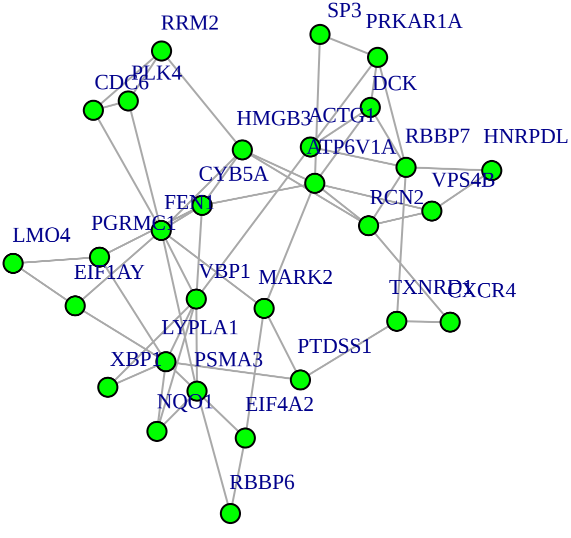

MST2 of the coexpression network for NEG ALL

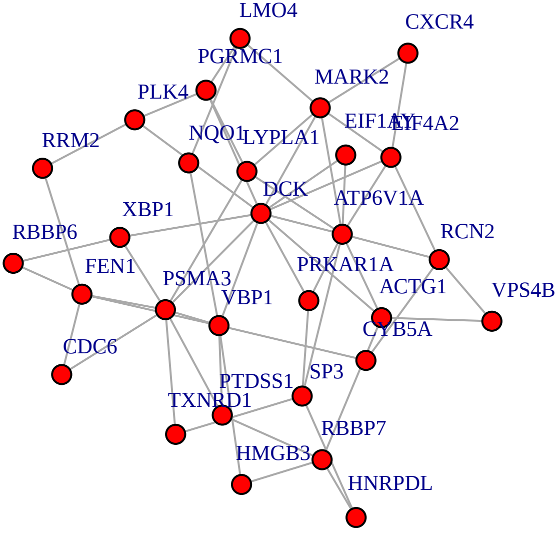

Pathway: BACOLOD_RESISTANCE_TO_ALKYLATING_AGENTS_UP

There are 18 genes in this pathway. This pathway was detected by GSCA

BCR/ABL ALL
Major Gene (BCR/ABL): IGF1
Weight Factor: 1.344
Major Gene (NEG): ARSF
Weight Factor: 1.121

NEG ALL
Major Gene (NEG): ARSF
Weight Factor: 1.183
Major Gene (BCR/ABL): IGF1
Weight Factor: 1.169

MST2 of the coexpression network for BCR/ABL ALL

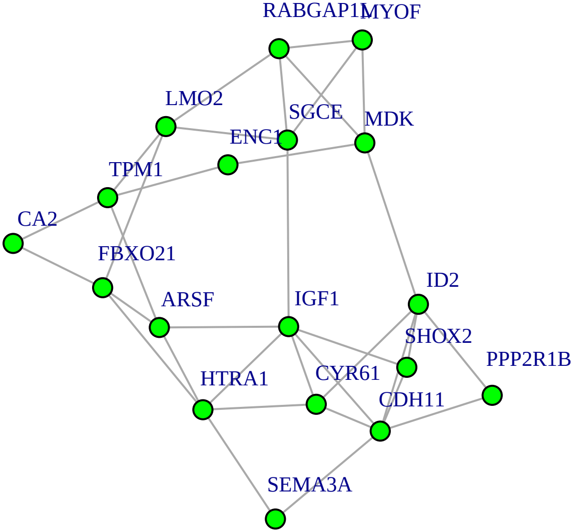

MST2 of the coexpression network for NEG ALL

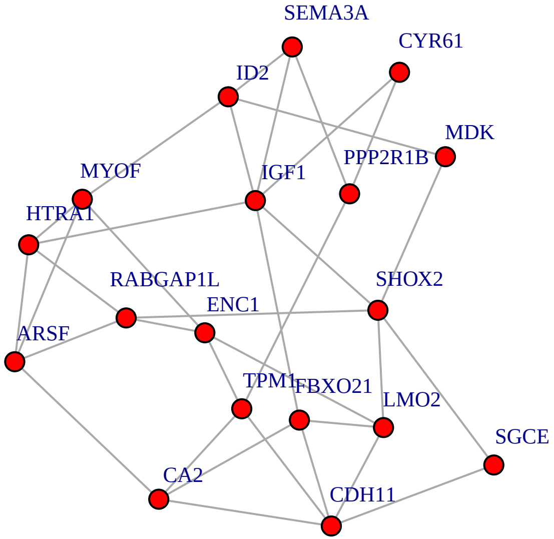

Pathway: GENTILE_UV_RESPONSE_CLUSTER_D7

There are 31 genes in this pathway. This pathway was detected by GSCA

BCR/ABL ALL
Major Gene (BCR/ABL): CTDSP2
Weight Factor: 1.338
Major Gene (NEG): MORC3
Weight Factor: 1.178

NEG ALL
Major Gene (NEG): MORC3
Weight Factor: 1.384
Major Gene (BCR/ABL): CTDSP2
Weight Factor: 1.258

MST2 of the coexpression network for BCR/ABL ALL

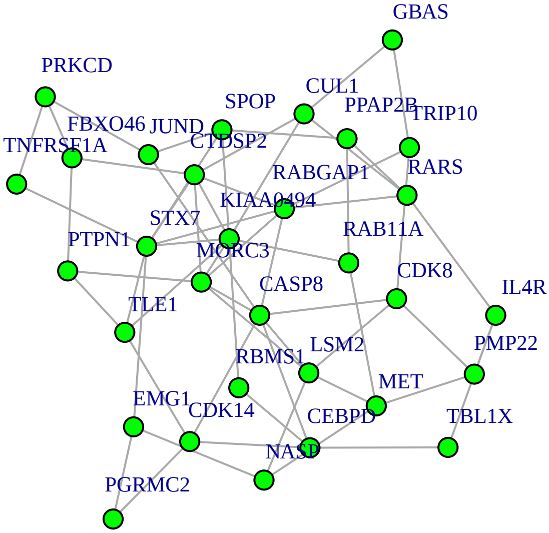

MST2 of the coexpression network for NEG ALL

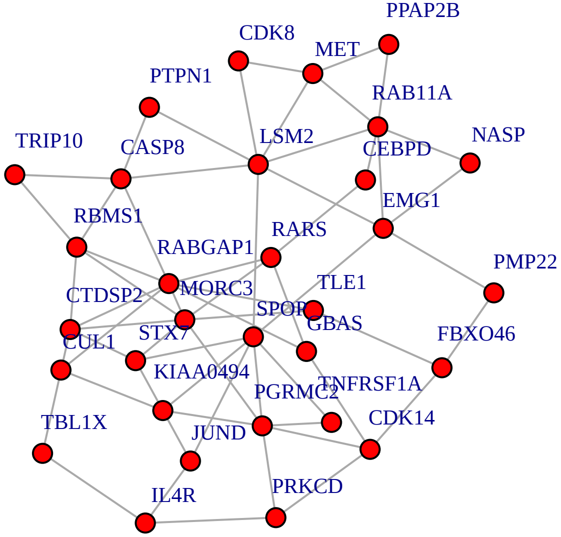

Pathway: ZUCCHI_METASTASIS_UP

There are 18 genes in this pathway. This pathway was detected by GSCA

BCR/ABL ALL
Major Gene (BCR/ABL): H2AFZ
Weight Factor: 1.367
Major Gene (NEG): H2AFZ
Weight Factor: 1.367

NEG ALL
Major Gene (NEG): H2AFZ
Weight Factor: 1.382
Major Gene (BCR/ABL): H2AFZ
Weight Factor: 1.382

MST2 of the coexpression network for BCR/ABL ALL

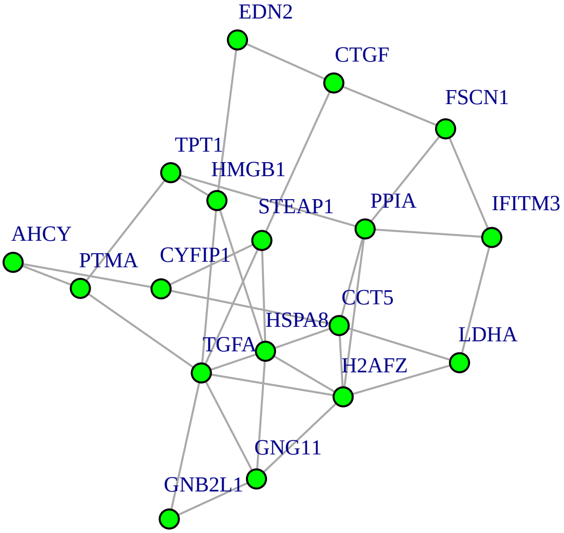

MST2 of the coexpression network for NEG ALL

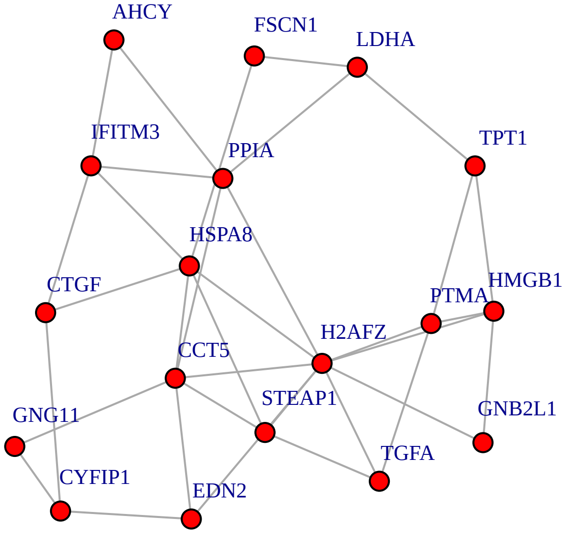

Pathway: ZHANG_TARGETS_OF_EWSR1_FLI1_FUSION

There are 85 genes in this pathway. This pathway was detected by GSCA

BCR/ABL ALL
Major Gene (BCR/ABL): FNBP1
Weight Factor: 1.4
Major Gene (NEG): JAK1
Weight Factor: 1.37

NEG ALL
Major Gene (NEG): JAK1
Weight Factor: 1.587
Major Gene (BCR/ABL): FNBP1
Weight Factor: 1.088

MST2 of the coexpression network for BCR/ABL ALL

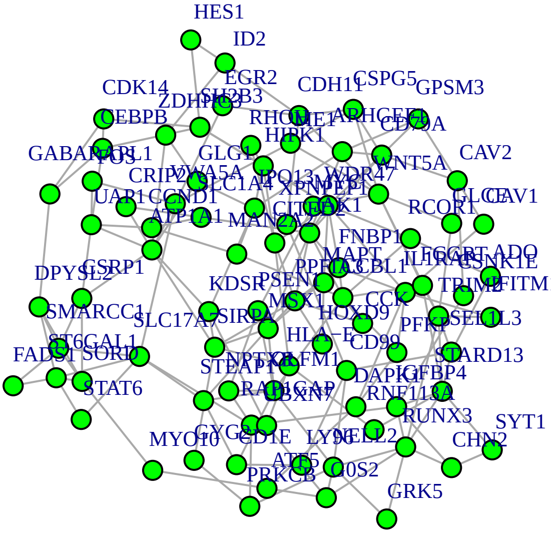

MST2 of the coexpression network for NEG ALL

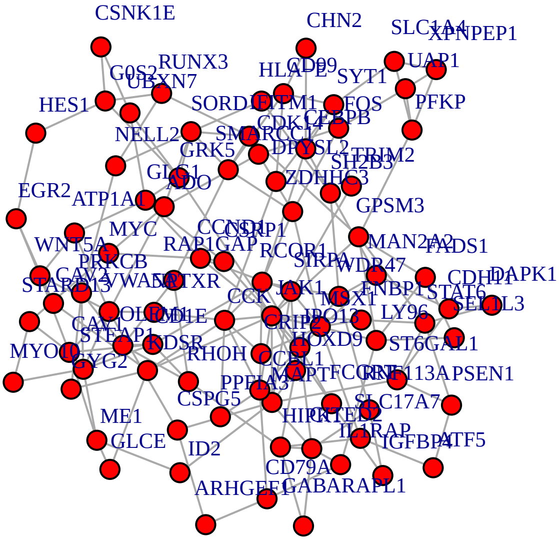

Pathway: KEGG_PATHOGENIC_ESCHERICHIA_COLI_INFECTION

There are 40 genes in this pathway. This pathway was detected by GSCA

BCR/ABL ALL
Major Gene (BCR/ABL): ROCK1
Weight Factor: 1.321
Major Gene (NEG): NCK2
Weight Factor: 1.015

NEG ALL
Major Gene (NEG): NCK2
Weight Factor: 1.395
Major Gene (BCR/ABL): ROCK1
Weight Factor: 0.985

MST2 of the coexpression network for BCR/ABL ALL

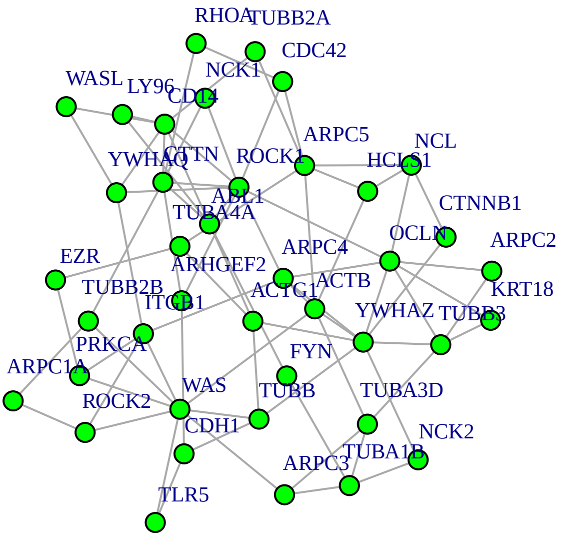

MST2 of the coexpression network for NEG ALL

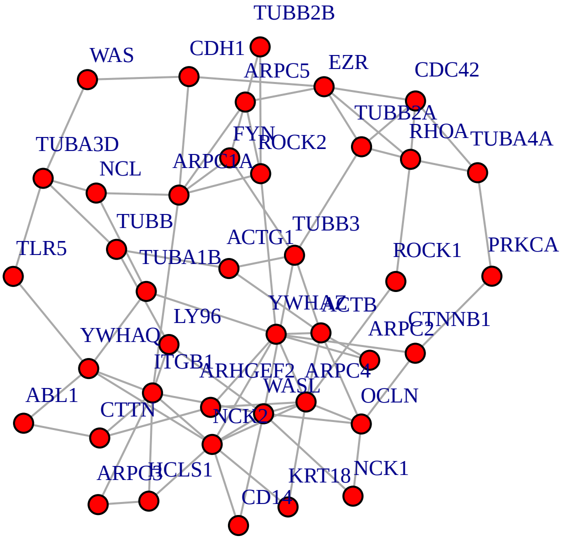

Pathway: BIOCARTA_ECM_PATHWAY

There are 24 genes in this pathway. This pathway was detected by GSCA

BCR/ABL ALL
Major Gene (BCR/ABL): ROCK1
Weight Factor: 1.49
Major Gene (NEG): ITGB1
Weight Factor: 0.851

NEG ALL
Major Gene (NEG): ITGB1
Weight Factor: 1.374
Major Gene (BCR/ABL): ROCK1
Weight Factor: 1.204

MST2 of the coexpression network for BCR/ABL ALL

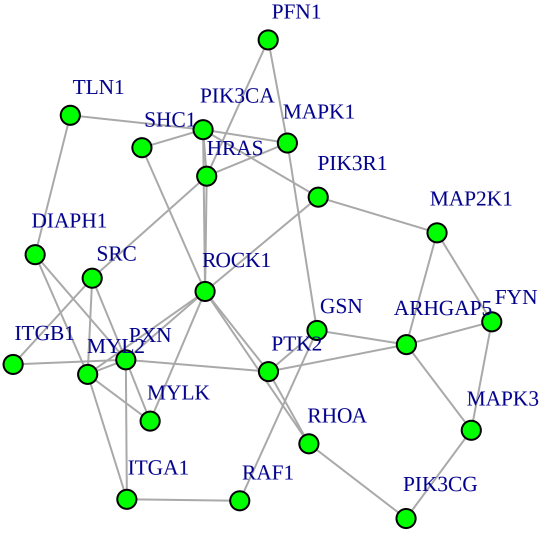

MST2 of the coexpression network for NEG ALL

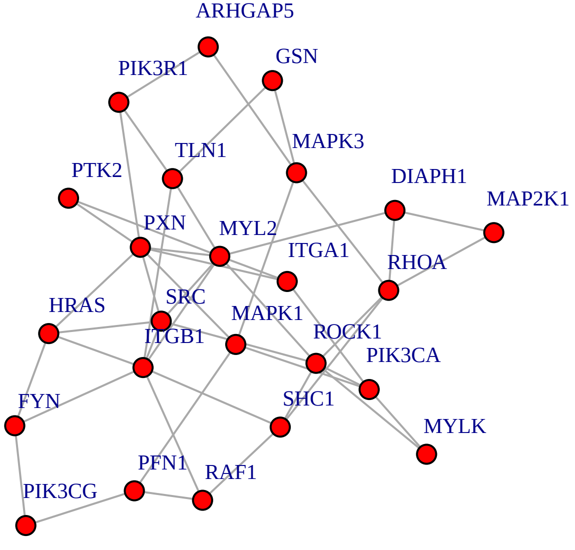

Pathway: BIOCARTA_MYOSIN_PATHWAY

There are 29 genes in this pathway. This pathway was detected by GSCA

BCR/ABL ALL
Major Gene (BCR/ABL): ROCK1
Weight Factor: 1.452
Major Gene (NEG): ARHGEF16
Weight Factor: 1.302

NEG ALL
Major Gene (NEG): ARHGEF16
Weight Factor: 1.384
Major Gene (BCR/ABL): ROCK1
Weight Factor: 1.236

MST2 of the coexpression network for BCR/ABL ALL

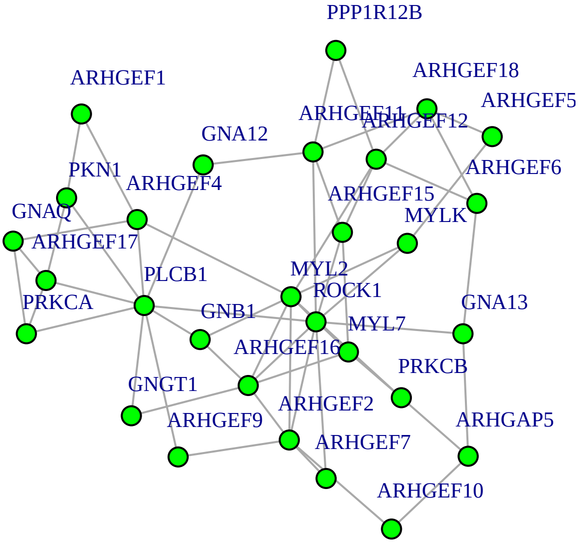

MST2 of the coexpression network for NEG ALL

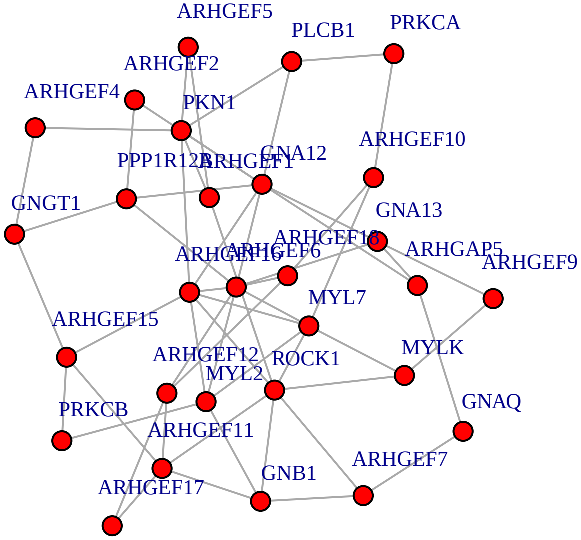

Pathway: BIOCARTA_LONGEVITY_PATHWAY

There are 15 genes in this pathway. This pathway was detected by GSCA

BCR/ABL ALL
Major Gene (BCR/ABL): HRAS
Weight Factor: 1.321
Major Gene (NEG): SOD1
Weight Factor: 1.257

NEG ALL
Major Gene (NEG): SOD1
Weight Factor: 1.392
Major Gene (BCR/ABL): HRAS
Weight Factor: 1.163

MST2 of the coexpression network for BCR/ABL ALL

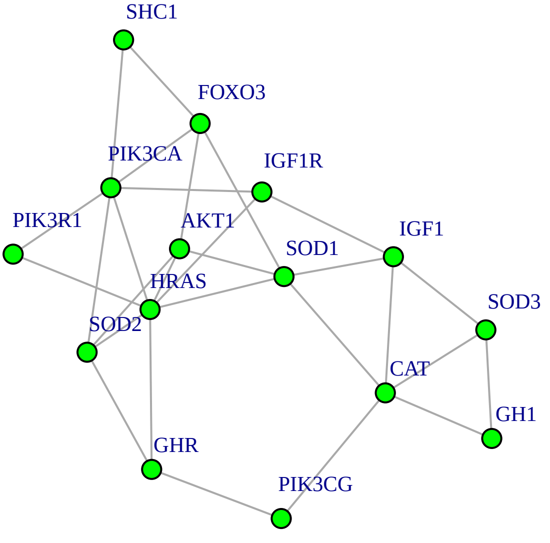

MST2 of the coexpression network for NEG ALL

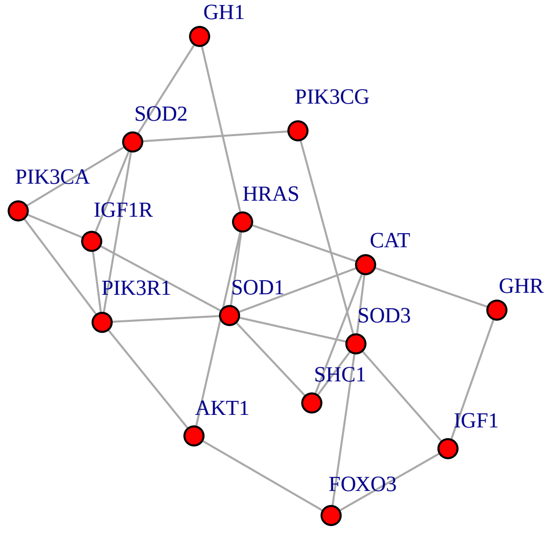

Pathway: REACTOME_CELL_SURFACE_INTERACTIONS_AT_THE_VASCULAR_WALL

There are 81 genes in this pathway. This pathway was detected by GSCA

BCR/ABL ALL
Major Gene (BCR/ABL): PPIL2
Weight Factor: 1.449
Major Gene (NEG): PTPN11
Weight Factor: 1.253

NEG ALL
Major Gene (NEG): PTPN11
Weight Factor: 1.599
Major Gene (BCR/ABL): PPIL2
Weight Factor: 1.532

MST2 of the coexpression network for BCR/ABL ALL

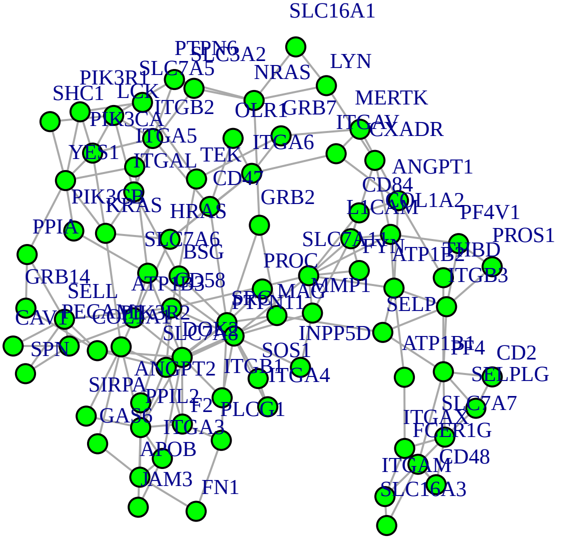

MST2 of the coexpression network for NEG ALL

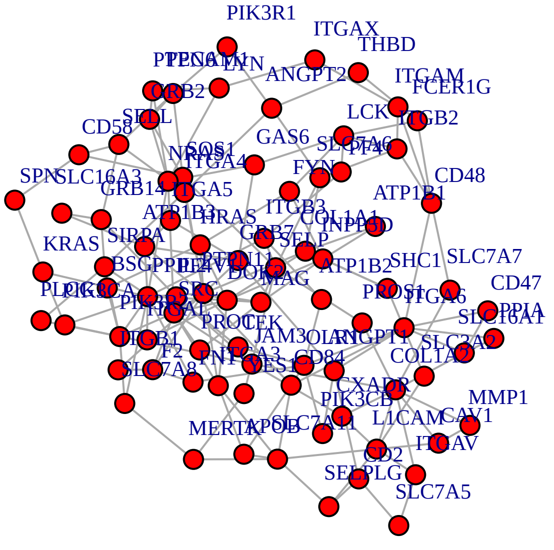

Pathway: REACTOME_CYTOSOLIC_TRNA_AMINOACYLATION

There are 20 genes in this pathway. This pathway was detected by GSCA

BCR/ABL ALL
Major Gene (BCR/ABL): IARS
Weight Factor: 1.273
Major Gene (NEG): IARS
Weight Factor: 1.273

NEG ALL
Major Gene (NEG): IARS
Weight Factor: 1.303
Major Gene (BCR/ABL): IARS
Weight Factor: 1.303

MST2 of the coexpression network for BCR/ABL ALL

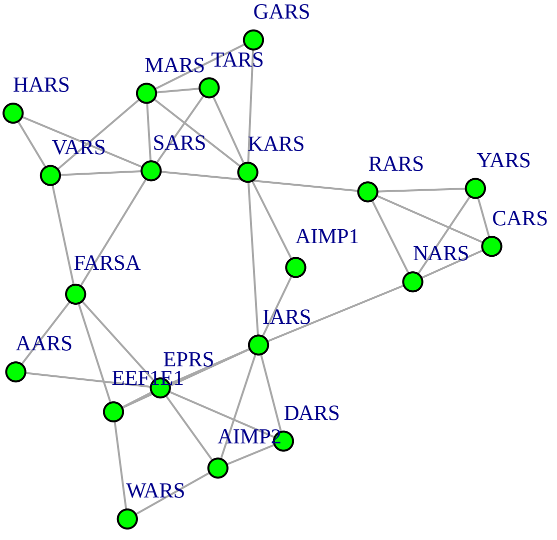

MST2 of the coexpression network for NEG ALL

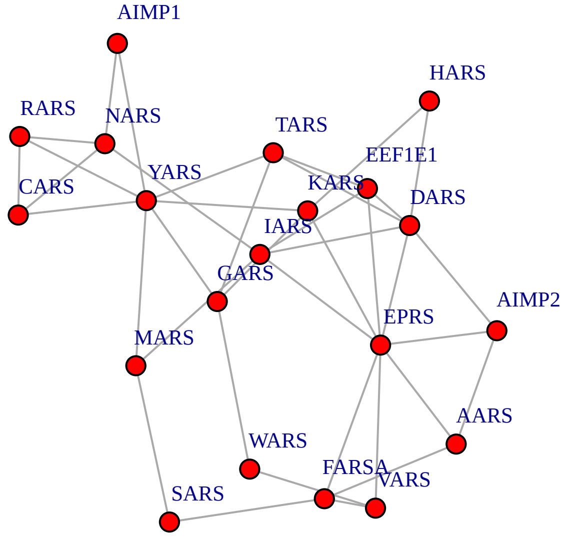

_MEDIATES_DOWN_MODULATION_OF_CELL_SURFACE_RECEPTORS_BY_RECRUITING_THEM_T

There are 16 genes in this pathway. This pathway was detected by GSCA

BCR/ABL ALL
Major Gene (BCR/ABL): AP2S1
Weight Factor: 1.305
Major Gene (NEG): CD8B
Weight Factor: 1.194

NEG ALL
Major Gene (NEG): CD8B
Weight Factor: 1.282
Major Gene (BCR/ABL): AP2S1
Weight Factor: 1.122

MST2 of the coexpression network for BCR/ABL ALL

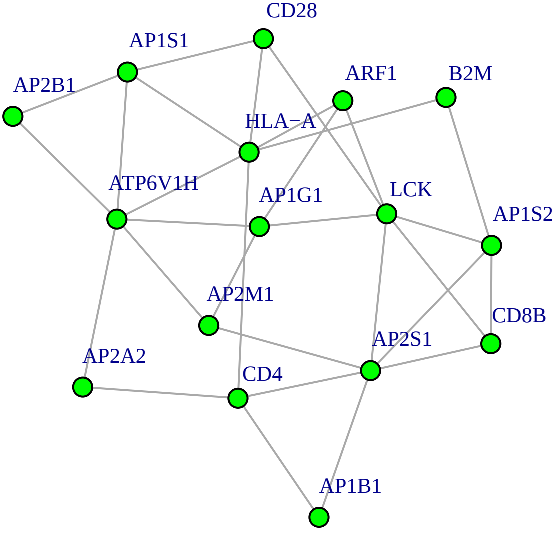

MST2 of the coexpression network for NEG ALL

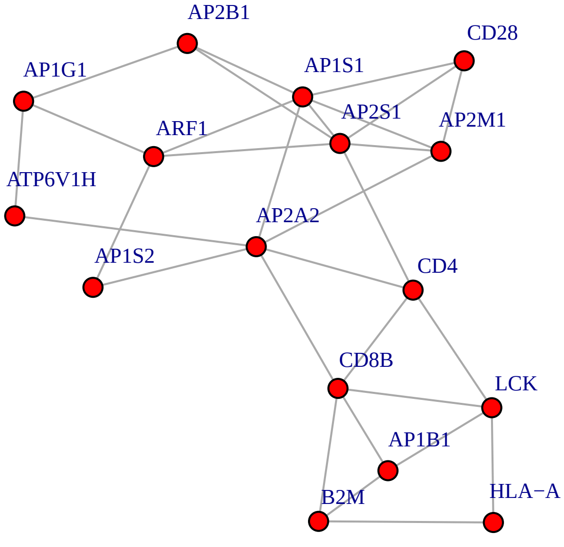

athway: REACTOME_THE_ROLE_OF_NEF_IN_HIV1_REPLICATION_AND_DISEASE_PATHOGENESI

There are 23 genes in this pathway. This pathway was detected by GSCA

BCR/ABL ALL
Major Gene (BCR/ABL): AP2S1
Weight Factor: 1.272
Major Gene (NEG): CD8B
Weight Factor: 1.198

NEG ALL
Major Gene (NEG): CD8B
Weight Factor: 1.399
Major Gene (BCR/ABL): AP2S1
Weight Factor: 1.024

MST2 of the coexpression network for BCR/ABL ALL

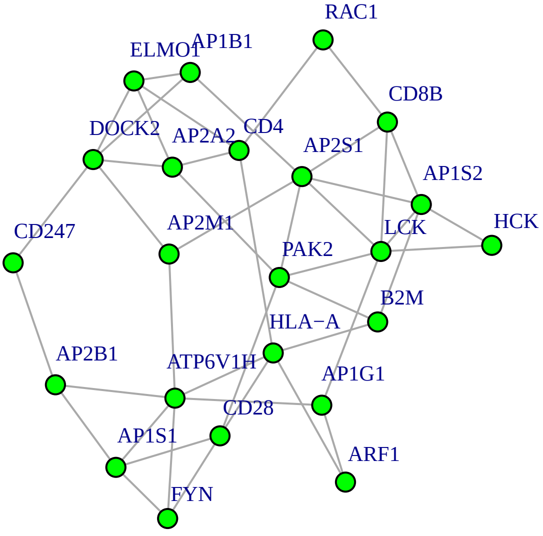

MST2 of the coexpression network for NEG ALL

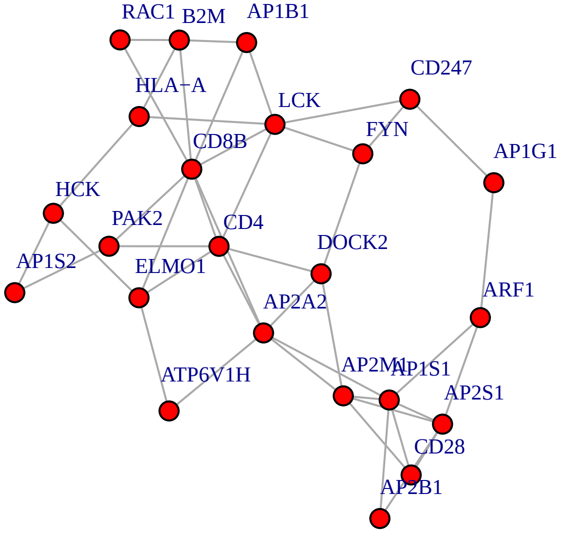

Pathway: REACTOME_TIE2_SIGNALING

There are 17 genes in this pathway. This pathway was detected by GSCA

BCR/ABL ALL
Major Gene (BCR/ABL): PIK3R2
Weight Factor: 1.336
Major Gene (NEG): PTPN11
Weight Factor: 0.886

NEG ALL
Major Gene (NEG): PTPN11
Weight Factor: 1.4
Major Gene (BCR/ABL): PIK3R2
Weight Factor: 1.29

MST2 of the coexpression network for BCR/ABL ALL

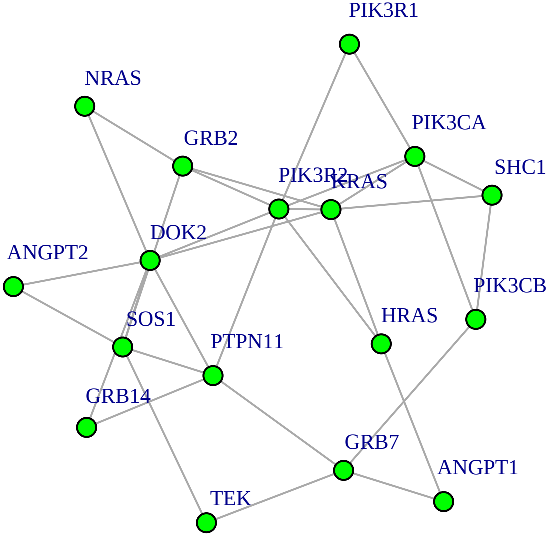

MST2 of the coexpression network for NEG ALL

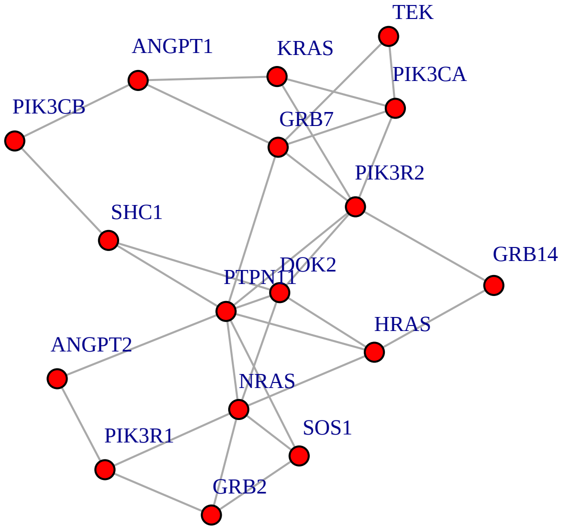

Pathway: REACTOME_TOLL_LIKE_RECEPTOR_4_CASCADE

There are 17 genes in this pathway. This pathway was detected by GSCA

BCR/ABL ALL
Major Gene (BCR/ABL): LY86
Weight Factor: 1.37
Major Gene (NEG): TLR1
Weight Factor: 0.863

NEG ALL
Major Gene (NEG): TLR1
Weight Factor: 1.293
Major Gene (BCR/ABL): LY86
Weight Factor: 1.036

MST2 of the coexpression network for BCR/ABL ALL

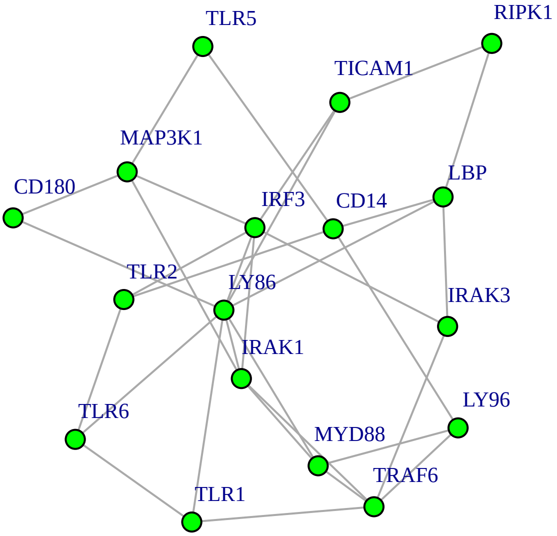

MST2 of the coexpression network for NEG ALL

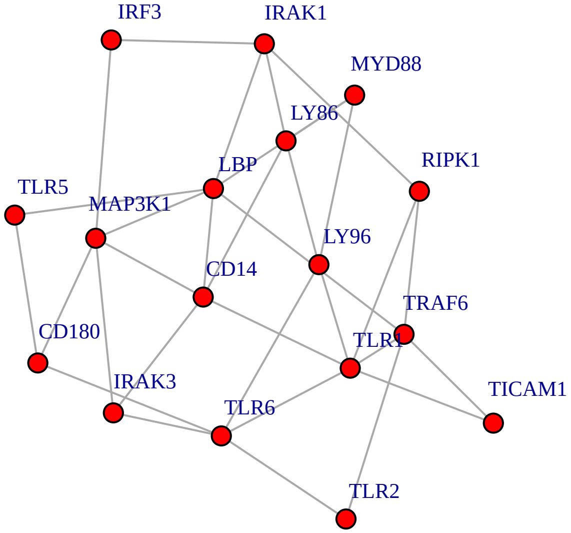

Pathway: SIG_REGULATION_OF_THE_ACTIN_CYTOSKELETON_BY_RHO_GTPASES

There are 31 genes in this pathway. This pathway was detected by GSCA

BCR/ABL ALL
Major Gene (BCR/ABL): GDI2
Weight Factor: 1.461
Major Gene (NEG): LIMK1
Weight Factor: 0.938

NEG ALL
Major Gene (NEG): LIMK1
Weight Factor: 1.388
Major Gene (BCR/ABL): GDI2
Weight Factor: 1.306

MST2 of the coexpression network for BCR/ABL ALL

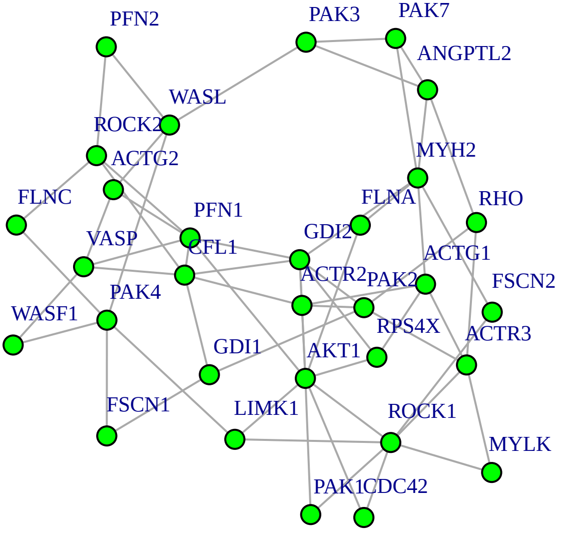

MST2 of the coexpression network for NEG ALL

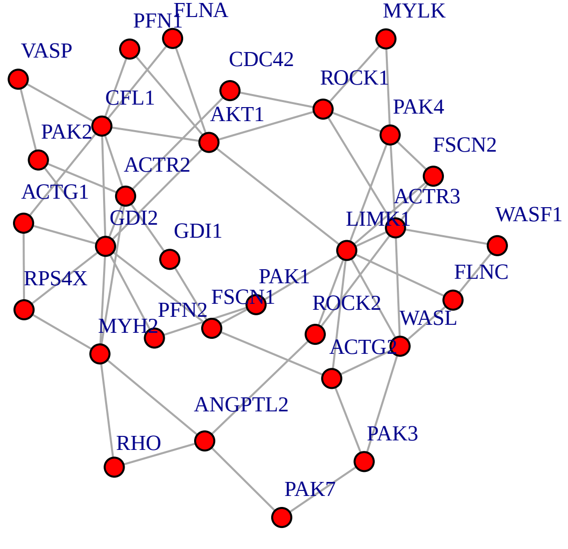

Supplement: Supplementary Data [file supp_btt687_Supplementary_Document_2.pdf]
